# Distal Ionic Substrate–Catalyst Interactions Enable Long-Range Stereocontrol: Access to Remote Quaternary Stereocenters through a Desymmetrizing Suzuki–Miyaura Reaction

**DOI:** 10.1021/jacs.1c12345

**Published:** 2022-01-03

**Authors:** Yazhou Lou, Junqiang Wei, Mingfeng Li, Ye Zhu

**Affiliations:** Department of Chemistry, Faculty of Science, National University of Singapore, 3 Science Drive 3, Singapore 117543

## Abstract

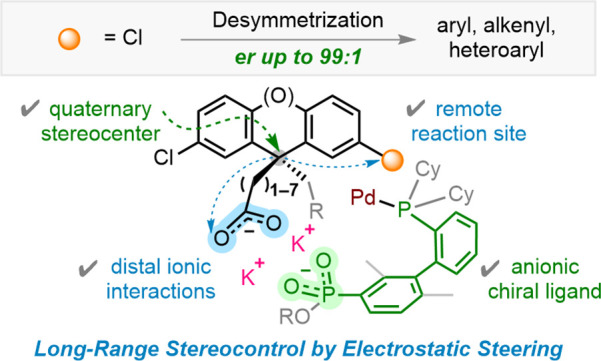

Spatial distancing of a substrate’s reactive group and nonreactive catalyst-binding group from its pro-stereogenic element presents substantial hurdles in asymmetric catalysis. In this context, we report a desymmetrizing Suzuki–Miyaura reaction that establishes chirality at a remote quaternary carbon. The anionic, chiral catalyst exerts stereocontrol through electrostatic steering of substrates, even as the substrate’s reactive group and charged catalyst-binding group become increasingly distanced. This study demonstrates that precise long-range stereocontrol is achievable by engaging ionic substrate–ligand interactions at a distal position.

The remarkable ability of enzymes to utilize attractive noncovalent interactions with distant, nonreactive groups of substrates to accelerate reactions and modulate selectivity has been regarded as a fundamental distinction from small-molecule catalysts.^1^ In recent years, substantial advances, particularly by Phipps and co-workers,^2,3^ have been achieved in harnessing distal ionic substrate–ligand interactions to control regio- and site-selectivity of transition-metal-catalyzed transformations (Scheme 1a, top).^4^ By contrast, integrating distal ionic interactions represents a compelling, yet undeveloped enantiocontrol strategy in transition-metal catalysis (Scheme 1a, bottom).^5^ In a prominent work, Miller and co-workers accomplished remote desymmetrization^6^ through asymmetric Ullmann coupling (Scheme 1b).^7^ Mechanistic investigation revealed an exquisite preorganization through proximal trifluoroacetamide anion–Cu binding and a distal Cs^+^ bridge between the substrate’s nonreacting enantiotopic arene and the peptide ligand’s terminal carboxylate.^7c^ Notwithstanding, chiral ligand scaffolds bearing nonligating charged groups (mostly tethered chirotopic ionic groups to date^8^) are uncommon, and the general effects of ion–ion interaction’s low directionality on long-range enantioinduction have not been studied.^9^ The broad potential of asymmetric transition-metal catalysis directed by distal ionic interactions has remained underexploited.

**Scheme 1:**
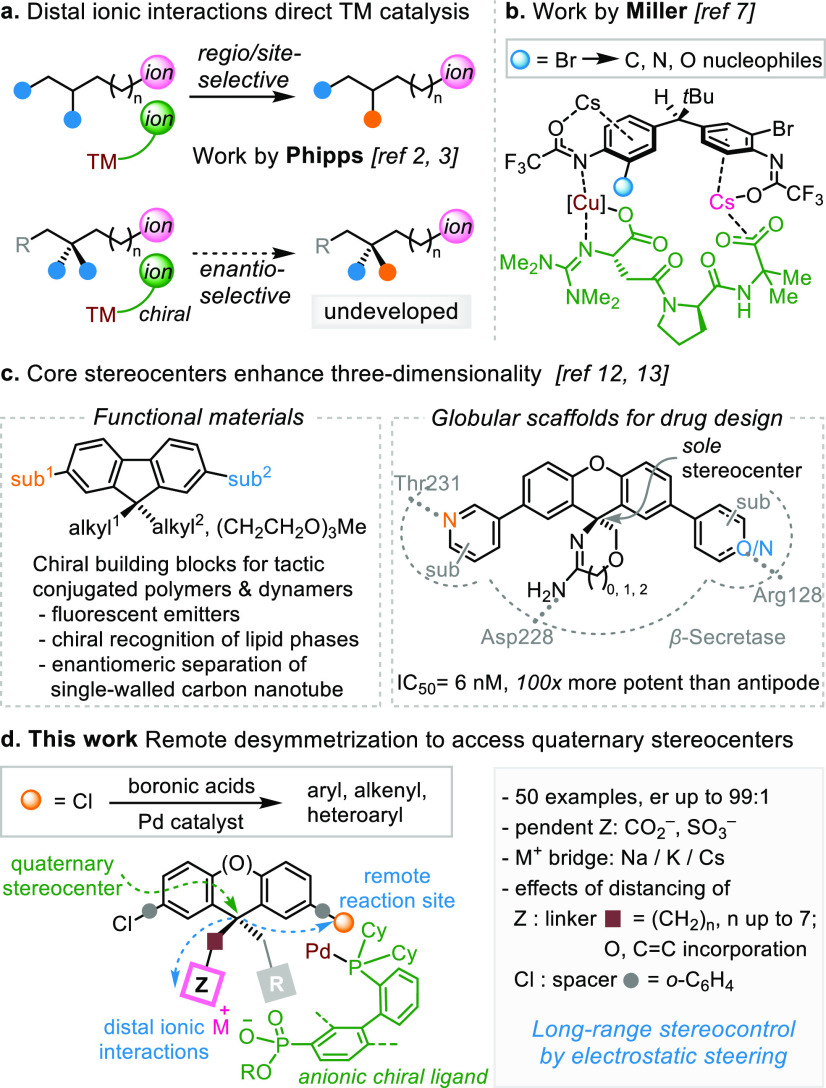
Desymmetrization Strategy for Remote Quaternary Stereocenters Directed by Distal Ionic Interactions

In pursue of such an enantiocontrol strategy, we targeted an untapped class of stereocenters through a transformation that allows us to rigorously test its viability. To date, remote desymmetrization to trisubstituted stereocenters has been made possible by only a handful of ingenious catalysts,^6,7,10^ and creation of remote quaternary carbon stereocenters has remained elusive.^11^ Quaternary stereocenters embedded in fluorenes^12^ and xanthenes^13^ possess distinctive ability to project chirality to distant loci of three-dimensional dispositions, an appealing feature for functional materials and pharmaceuticals^14^ (Scheme 1c). However, these enantio-enriched molecules are accessible only through chiral chromatography.^15^ We envisaged that Pd-catalyzed desymmetrizing Suzuki–Miyaura reaction^16^ of bis(chloroaryl)methane derivatives could furnish this class of core quaternary stereocenters (Scheme 1d).

Establishing quaternary stereocenters bearing sterically similar geminal substituents poses a major obstacle in catalytic desymmetrization,^17^ and distant reactive groups may conceivably exacerbate the challenge. We were drawn to the design principle by Phipps and co-workers using cation bridges between anionic substrates and sulfonated dialkylbiaryl phosphines^18^ in site-selective cross-coupling of dichloroarenes.^3^ We surmised that a novel anionic dialkylbiphenyl phosphine–Pd catalyst could interact with the charged substituent (Z^–^ M^+^) of the substrate preferentially (Scheme 1d). Furthermore, we reasoned that integrating the catalyst’s axial chirality^19,20^—spatial arrangement of Pd and phosphonate—into stereocontrol relay from ionic group Z to C–Cl bonds could be a viable approach to long-range asymmetric induction. As such, the effects of spatial distancing of ionic group Z and C–Cl bonds could be elucidated through judicious variation of the substrates. Here, we report that catalyst-controlled electrostatic steering of substrates led to realization of an enantioselective desymmetrizing Suzuki–Miyaura reaction that establishes chirality at remote quaternary stereocenters.

We commenced our study by synthesizing 3′-phosphonate dialkylbiphenyl phosphines (Scheme 2). Racemic **L1**, readily prepared from RuPhos,^21^ was converted to **L2** as separable atropo-diastereomers in three steps. Upon desulfinylation, the axial chirality of **L2** was preserved in the resulting individual enantiomers of **L1** by a methyl “atropo-tag”. Subsequent phosphonylation and hydrolysis afforded enantioenriched **L4**. Besides, **L5** (depicted in Table 1) was prepared following an analogous synthetic route starting from SPhos.^22^

**Scheme 2:**
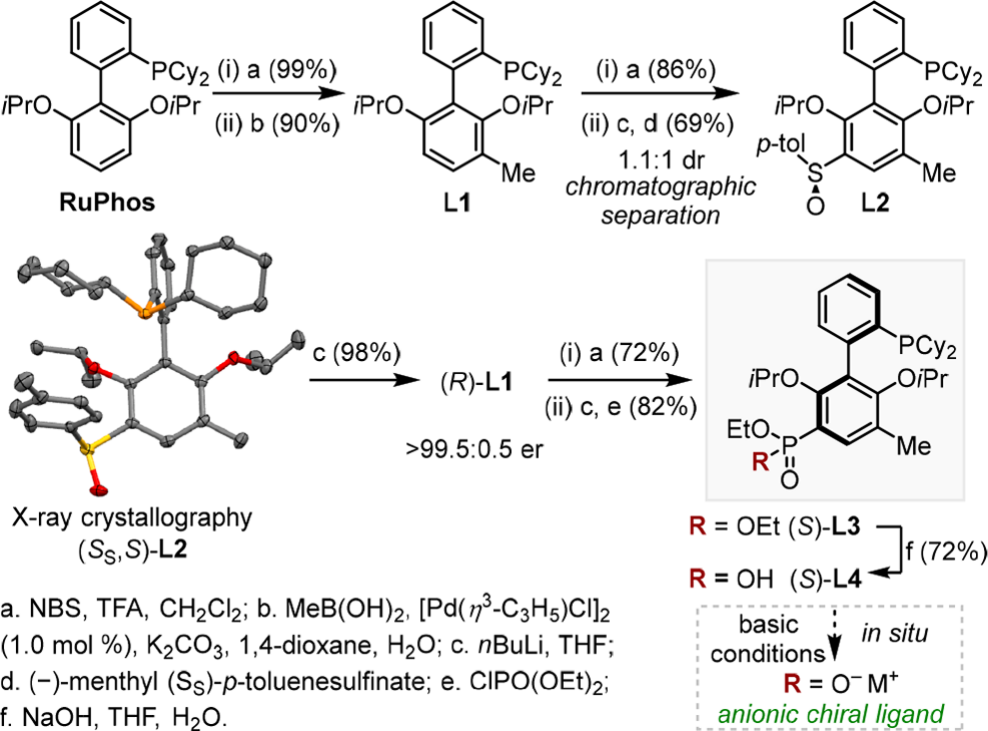
Synthesis of Anionic, Axially Chiral Ligands

**Table 1:**
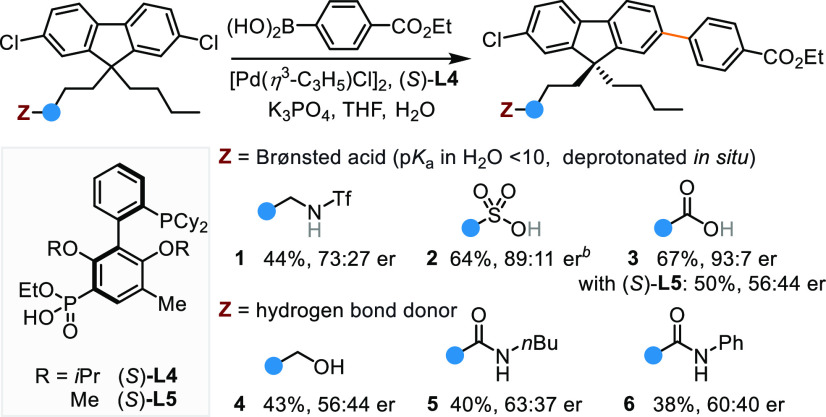
Effect of Pendent Group on Pd-Catalyzed Desymmetrizing Suzuki–Miyaura Reaction^a^

a Reaction conditions: substrate (0.1 mmol), aryl boronic acid (0.1 mmol), [Pd(η^3^-C_3_H_5_)Cl]_2_ (1.0 mol%), (*S*)-**L4** (2.2 mol%), K_3_PO_4_ (3 equiv), THF (9.5 mL/mmol), H_2_O (0.5 mL/mmol), 60 °C, 18 h. Isolated yields reported. Enantiomeric ratios (er) were determined by chiral high-performance liquid chromatography.

b Isolated as ethyl ester. Tf = trifluoromethanesulfonyl.

The nature of substrate’s catalyst-binding group is anticipated to influence the stereochemical outcome of desymmetrization if distal substrate–ligand interactions are operating. Therefore, we evaluated a range of Brønsted acidic groups^3^ (Table 1). Each group is separated from fluorene C9 by four rotatable bonds. This way, differences between their steric effects imposed on the pro-stereogenic center are minimized. Using (*S*)-**L4** as ligand, the substrate bearing a distal triflamide underwent the desymmetrizing Suzuki–Miyaura reaction, affording the product in an encouraging 44% yield with 73:27 er (**1**). Replacing the triflamide with sulfo group (**2**) and carboxyl group (**3**) led to markedly improved results. By contrast, pendent hydrogen bond donors (**4**–**6**) resulted in comparably low enantioselectivity.

Subsequently, we focused our efforts on reaction optimization (Tables S1–S5 in the Supporting Information (SI)). The model reaction gave merely 56:44 er using SPhos-derived (*S*)-**L5** as ligand (Table 1, **3**). Investigating solvent effect using (*S*)-**L4**, we found that the enantioselectivity diminished in DMF (66:34 er). This observation is consistent with a participating cation bridge, which is disrupted by strong solvation of cations in polar aprotic solvents.^7c^ To probe the effects of cations, we surveyed alkali-metal hydroxides and carbonates as exogenous base. Similar results were observed using Na, K, and Cs bases irrespective of the counteranions (96:4–97:3 er), while Li bases were inferior. The reaction remained enantioselective using Bu_4_NOH as base (91:9 er), suggesting that stereocontrol is attainable in the organic phase. Finally, a 2-MeTHF–aqueous K_3_PO_4_ system was identified as the optimal reaction media.

We next studied the effects of distancing the ionic pendent group (Table 2a, **3** and **7**–**12**). Initially, we anticipated a steep drop in enantioselectivity once the distance between C–Cl bond and the distal carboxylate exceeds the span of catalyst. The entropic penalty incurred could obliterate the energetic differentiation of desymmetrization. Surprisingly, the catalyst system adapted well to changes in length of (CH_2_)_*n*_ (*n* = 1–7) linking the carboxyl group (32–67% yield, 82.5:17.5–96:4 er). Notably, desymmetrization was achieved even when the carboxylate was placed eight C–C bonds away from the quaternary carbon (**12**, 86.5:13.5 er). The results also substantiate the attractive nature of substrate–ligand interactions involving the distal carboxylate. Repulsive forces unlikely play the dominant role, because they can be easily avoided by shifting the carboxylate away without affecting the catalysis at the Pd center.

**Table 2:**
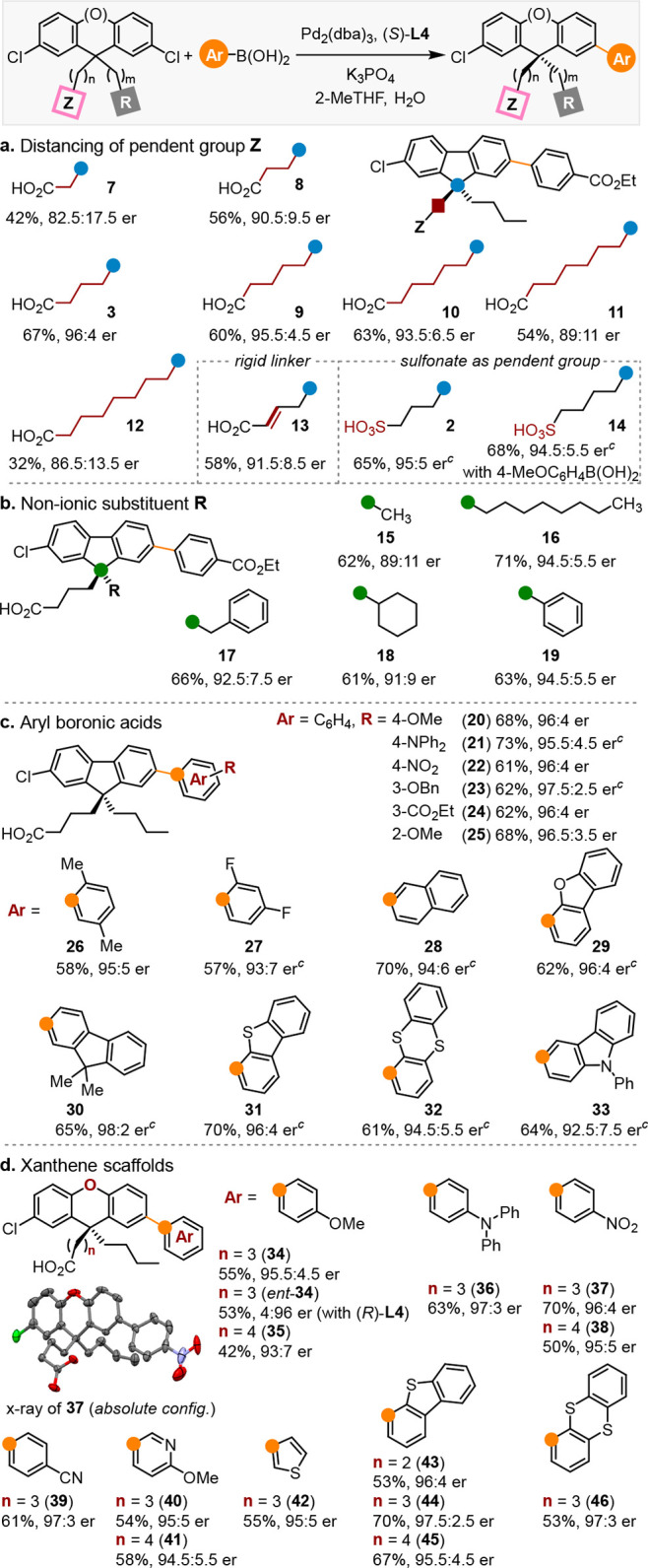
Substrate Scope of Pd-Catalyzed Remote Desymmetrization to Quaternary Stereocenters^a^^,^^b^

a The absolute configurations of products were assigned by analogy to **37**.

b Standard reaction conditions: substrate (0.25 mmol), aryl boronic acid (0.30 mmol), Pd_2_(dba)_3_ (1.0 mol%), (*S*)-**L4** (2.2 mol%), K_3_PO_4_ (10 equiv), 2-MeTHF (20 mL/mmol), H_2_O (1.6 mL/mmol), 60 °C, 18 h. Isolated yields reported.

c Isolated as ethyl ester. dba = dibenzylideneacetone.

Furthermore, increasing the conformational rigidity by incorporating a double bond into the linker only led to marginal decrease in enantioselectivity (Table 2a, **13**, 91.5:8.5 er).

The ability to direct the catalyst is not unique to carboxylate, which presumably serves as a diffuse negative charge occupying the distal end of C9 substituent. Recently, fluorenes bearing pendent sulfonate have emerged as prominent components of conjugated polyelectrolytes.^23^ Investigation of sulfo group in the desymmetrization reactions revealed that it functioned equally well, affording the products in up to 95:5 er (Table 2a, **2** and **14**).

Besides the catalyst’s effectiveness in long-range stereocontrol, we were excited by its ability to construct quaternary stereocenters bearing sterically similar geminal substituents. Gratifyingly, variations in the non-ionic C9 substituents including alkyl, benzyl, and phenyl groups, were well tolerated (Table 2b, **15**–**19**, 61–71% yield, 89:11–94.5:5.5 er). Clearly, the size difference between the geminal substituents is not the main determinant of enantioselectivity.

The transformation is compatible with a broad spectrum of arylboronic acids (Table 2c). Substituents at the *para*- (**20**–**22**), *meta*- (**23** and **24**), and *ortho*- (**25**–**27**) positions, irrespective of electronic properties, had an insignificant influence on the enantioselectivity (57–73% yield, 93:7–97.5:2.5 er). Additionally, a wide range of polycyclic aromatics commonly employed in π-conjugated materials can be installed in 61–70% yield, 92.5:7.5–98:2 er (**28**–**33**).

The remote desymmetrization strategy is also applicable to accessing enantioenriched xanthenes (Table 2d). Specifically, dichloroxanthenes participated in the transformation with various electron-rich aryl (**34**–**36**), electron-deficient aryl (**37**–**39**), heteroaryl (**40**–**42**), and polycyclic aryl (**43**–**46**) boronic acids, affording the products in 42–70% yield, 93:7–97.5:2.5 er.

Intrigued by the catalyst’s ability in exerting long-range stereocontrol, we further evaluated its adaptability to distancing the reactive group and to altering the catalyst-binding substituent. First, we placed the C–Cl bonds farther apart (Scheme 3a). Despite the substantial structural change in the substrates, the catalyst remained capable of imparting asymmetric induction (**47** and **48**, up to 89.5:10.5 er). Next, we studied the stereochemical outcome of incorporating an oxygen atom adjacent to the pro-stereogenic carbon, which possibly provides additional interaction with the K^+^ bridge (Scheme 3b). Indeed, the remote desymmetrization reactions proceeded in up to 99:1 er (**49** and **50**).

**Scheme 3:**
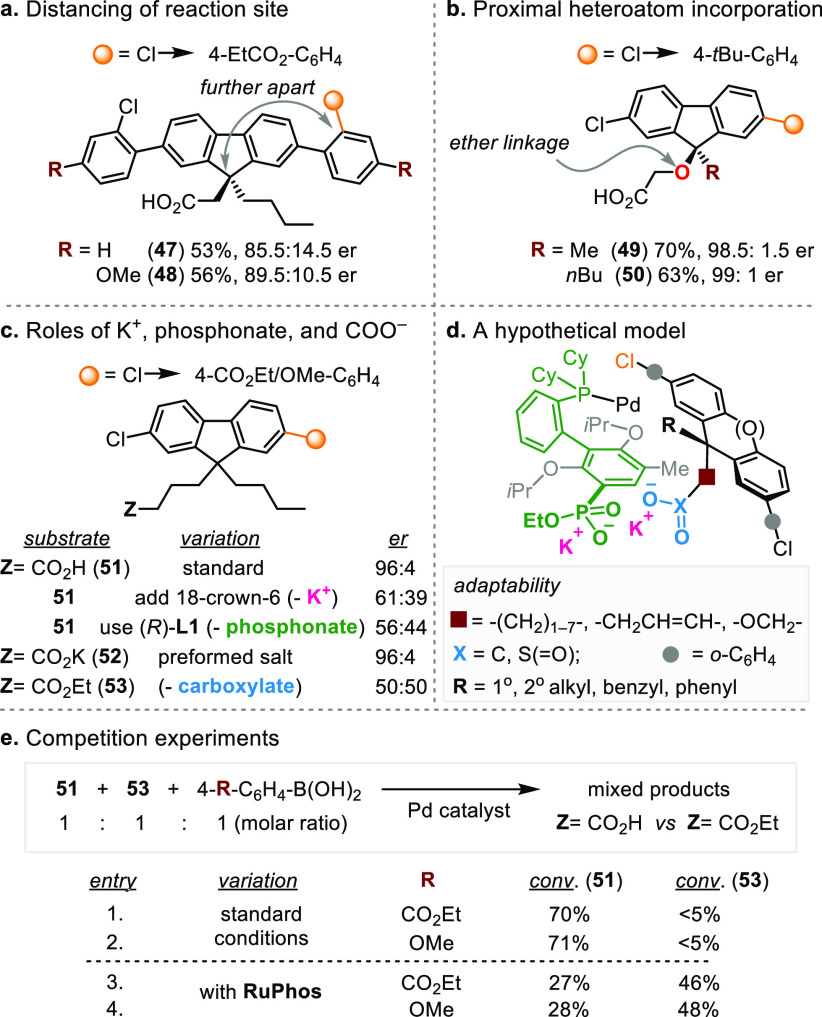
Substrate Scope Expansion and Control Experiments to Probe the Effect of Spatial Distancing and the Role of Distal Ionic Interactions

Based on the results of control experiments, we concluded that K^+^, phosphonate of (*S*)-**L4**, and carboxylate of substrate contribute collectively to the ionic substrate–ligand interactions (Scheme 3c). Encapsulation of K^+^ by 18-crown-6 led to diminished enantioselectivity (61:39 er), and reduction in er paralleled the quantity of added 18-crown-6 (SI). The critical role of ligand’s phosphonate was evidenced by the negligible enantioinduction by truncated ligand (*R*)-**L1** (56:44 er). In comparison, the reaction using (*S*)-**L3** gave 83:17 er. The ion–dipole interaction between K^+^ and P=O of (*S*)-**L3** is inferior to the ion–ion interaction between K^+^ and P–O^–^ of (*S*)-**L4** in asymmetric induction. In contrast to the preformed carboxylate salt (**52**), racemic product was obtained from corresponding ethyl ester (**53**), which lacks the key ion–ion interactions with K^+^.

The oxidative addition step^24^ is plausibly selectivity-determining, while other steps in the catalytic cycle could contribute to the enantioselectivity.^25^ On the basis of the absolute configurations of (*S*)-**L4** and **37**, we hypothesized a model^26^ to illustrate the putative distal ionic interactions (Scheme 3d). Unlike enzymes’ large and deep binding clefts that confer substrate specificity, Pd–(*S*)-**L4**, which carries a diffuse negative charge at an unshielded phosphonate, preserves distal ionic interactions when it adapts to substrates’ structural diversity in pendent groups and linkers, non-ionic substituents (R), and distanced C–Cl bonds.

Nature utilizes long-range electrostatic attractions to significantly accelerate biochemical processes that require precise orientations of biomolecules.^27^ We postulated that the Pd-catalyzed remote desymmetrization follows the same principle of electrostatic steering of charged substrates.^28^ To elucidate this phenomenon, we carried out competition experiments between carboxylate acid **51** and ethyl ester **53** (Scheme 3e). Under the standard conditions, **51** reacted predominantly regardless of the electronic property of aryl boronic acids (entries 1 and 2). Such selectivity is catalyst-controlled, as competition experiments using RuPhos slightly favored **53** (entries 3 and 4). The observations, coupled with the noticeable difference between their enantioselectivities (Scheme 3c), indicate that compared with **53**, the ionic interactions arising from distal carboxylate of **51** lead to a preferential increase in the rate of selectivity-determining step at one of the enantiotopic reaction sites.

The desymmetrization strategy offers efficient access to core quaternary stereocenters that project substituents to widely spaced positions (Scheme 4). As an illustration, **3** underwent Pd-catalyzed C–B, C–C, and C–N bond formation reactions (Scheme 4a), furnishing combinations of functionalities at two distant sites (**54**–**56**). Moreover, the sequential desymmetrizing cross-coupling is enantiodivergent (Scheme 4b). Starting from **57**, the stereochemical outcome was precisely controlled by the choreography of heteroaryl and alkenyl boronic acids (**58** and **59**), where both enantiomers of **60** were synthesized using (*S*)-**L4** as ligand. Subsequent transformations afforded spirocycle **61** as a β-secretase inhibitor^13^ analog.

**Scheme 4:**
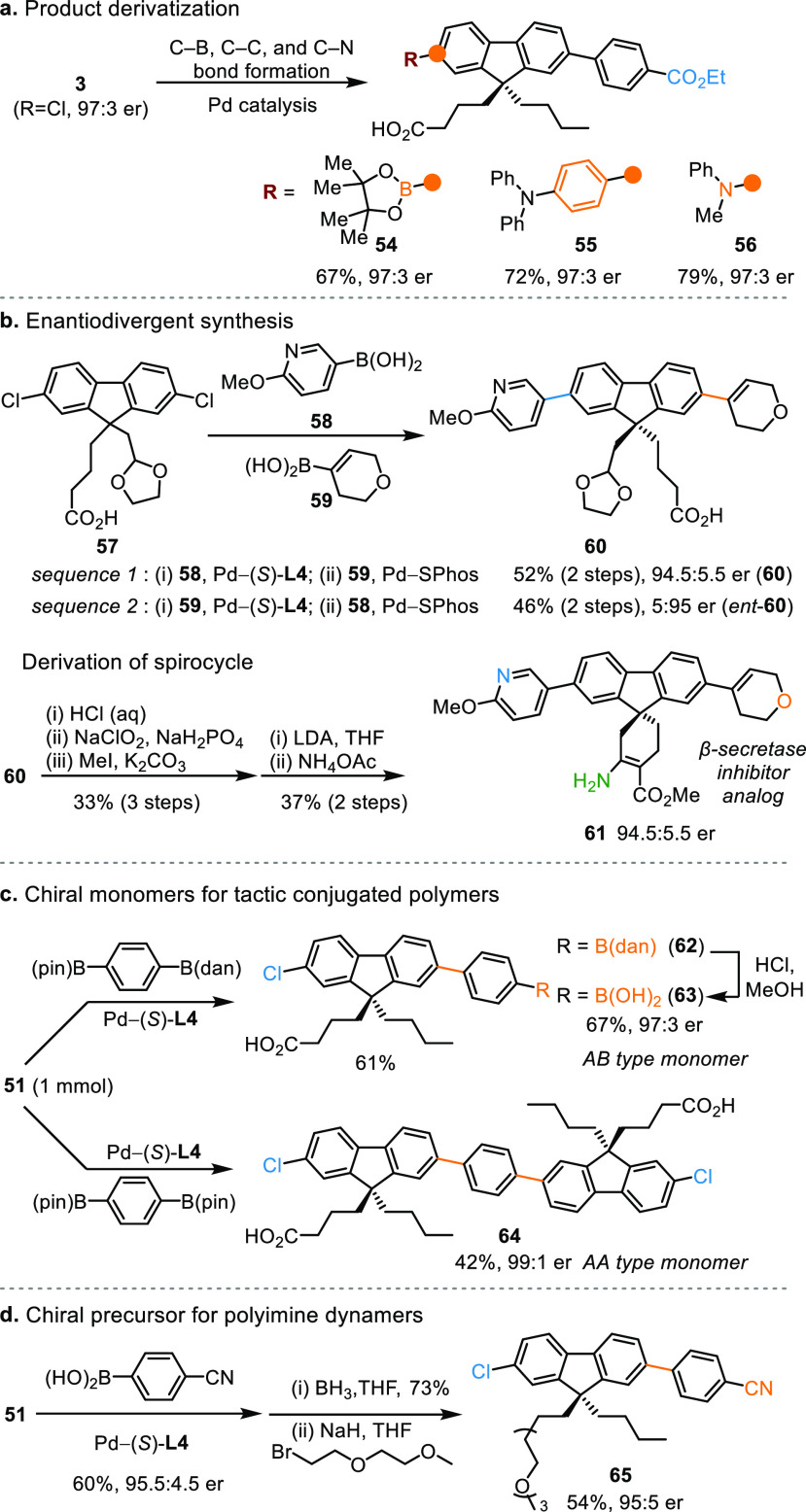
Synthetic Applications of Pd-Catalyzed Desymmetrizing Suzuki–Miyaura Reaction

As a practical feature, the remote desymmetrization can be readily adopted to construct chiral building blocks of fluorene-based materials without rerouting existing syntheses. For example, desymmetrization of **51** with 4-B(dan) phenylboronic pinacol ester (dan = naphthalene-1,8-diaminato) proceeded smoothly on a 1 mmol scale using 1 mol% Pd–(*S*)-**L4**, affording AB-type monomer^12d^**63** in 97:3 er upon deprotection of coupling product **62**. Notably, we also succeeded in synthesizing enantioenriched (99:1 er) AA-type monomer **64** in one step using 1,4-phenylenediboronic pinacol ester as bis-coupling partner (Scheme 4c). Additionally, the pendent carboxyl group can be readily converted to other functionalities, such as ethylene glycol chain of a chiral precursor for polyimine dynamers^12c^ (Scheme 4d, **65**).

In summary, we have realized a desymmetrizing Suzuki–Miyaura reaction that establishes chirality at a remote quaternary carbon. The anionic catalyst’s ability to transmit asymmetry across large distances enables facile access to enantioenriched molecules that project chirality to widely spaced loci. We have demonstrated that by engaging distal ionic substrate–catalyst interactions, it is possible to surmount the hurdle in asymmetric catalysis arising from spatial distancing of substrate’s reactive group and catalyst-binding group. We anticipate that pursuing this strategy could stimulate rational design of catalysts capable of long-range asymmetric induction to create chirality that would be difficult to construct using conventional methods.

## Supporting Information Available

Experimental details and characterization data (PDF)
