# Supplementary material for: Distal Ionic Substrate–Catalyst Interactions Enable Long-Range Stereocontrol: Access to Remote Quaternary Stereocenters through a Desymmetrizing Suzuki–Miyaura Reaction
Source: J Am Chem Soc. 2022 Jan 3;144(1):123–9. doi: 10.1021/jacs.1c12345 (PMC9549467; doi:10.1021/jacs.1c12345)

Distal Ionic Substrate–Catalyst Interactions Enable Long-Range Stereocontrol: Access to Remote Quaternary Stereocenters through a Desymmetrizing Suzuki–Miyaura Reaction

Yazhou Lou, Junqiang Wei, Mingfeng Li, Ye Zhu*

Department of Chemistry, Faculty of Science, National University of Singapore, 3 Science Drive 3, Singapore 117543
*Correspondence to: chmzhu@nus.edu.sg

Supporting Information

Table of Contents

List of acronyms and abbreviations 1

General information 3

Preparation of ligands 4

Preparation of substrates 11

Optimization of reaction conditions (Tables S1–S5) 33

General procedure of catalytic desymmetrization reaction 38

Characterization data of desymmetrization products 39

Control experiments 63

Synthetic applications 66

References 82

NMR spectra 83

HPLC traces 203

X-ray crystallography data 270

List of acronyms and abbreviations

**Ac**	acetyl
**Bu**	butyl
**COD**	1,5-cyclooctadiene
**Cy**	cyclohexyl
**dan**	naphthalene-1,8-diaminato
**dba**	dibenzylideneacetone
**DCC**	dicyclohexylcarbodiimide
**DCM**	dichloromethane
**DME**	1,2-dimethoxyethane
**DMF**	*N,N*-dimethylformamide
**Et**	ethyl
**HMDS**	bis(trimethylsilyl)amide
**Me**	methyl
**2-MeTHF**	2-methyltetrahydrofuran
**NBS**	*N*-bromosuccinimide
**NCS**	*N*-chlorosuccinimide
**Ph**	phenyl
**pin**	pinacolato
**Pr**	propyl
**RuPhos**	2-dicyclohexylphosphino-2′,6′-diisopropoxybiphenyl
**SPhos**	2-dicyclohexylphosphino-2′,6′-dimethoxybiphenyl
**TBAOH**	tetrabutylammonium hydroxide
**TFA**	trifluoroacetic acid
**THF**	tetrahydrofuran

**Tf**	trifluoromethylsulfonyl
**TMS**	trimethylsilyl
**Tol**	methylphenyl

General information

Materials: Commercially available reagents and solvents were used as received. Commercial dry solvents (Aldrich Sure/Seal™) were sparged with nitrogen before used in catalytic reactions. Solvents used for column chromatography were analytical grade.

Methods: Unless otherwise noted, all experiments were set up under an atmosphere of nitrogen in a glovebox or using standard Schlenk techniques. Reactions were monitored by thin layer chromatography (TLC), gas chromatography (GC), or nuclear magnetic resonance (NMR) analysis. Flash column chromatography was performed using Tsingdao silica gel (60, particle size 300-400 mesh). Yields refer to isolated yields after flash column chromatography purification.

Characterization: Products were characterized by means of nuclear magnetic resonance (NMR), mass spectrometry (MS), high performance liquid chromatography (HPLC), and optical rotation. NMR spectra were recorded on a Bruker DPX 400 spectrometer at 300 MHz for ^{1}H NMR, 101 MHz for ^{13}C NMR and 162 MHz for ^{31}P NMR or on a Bruker DPX 500 spectrometer at 500 MHz for ^{1}H NMR, 126 MHz for ^{13}C NMR, 202 MHz for ^{31}P NMR and 471 MHz for ^{19}F NMR in $CDCl_3$ with tetramethylsilane as internal standard. Chemical shifts were reported relative to tetramethylsilane (0 ppm) for ^{1}H NMR and relative to $CDCl_3$ (77.0 ppm) for ^{13}C NMR. ^{19}F spectra were calibrated from external standard ($CFCl_3$: 0 ppm). ^{31}P spectra were calibrated from external standard (85 wt% phosphoric acid: 0 ppm). NMR data are reported as: chemical shift (parts per million, ppm), multiplicity (s = singlet, d = doublet, t = triplet, q = quartet, m = multiplet), coupling constant (Hz), and integration. HPLC analysis was performed on a Shimadzu *i*-series HPLC system equipped with photodiode array (PDA) detector and Chiralcel and Chiralpak columns (0.46 cmØ× 25 cm). The wavelength that is an apex in the spectrum was selected for analysis of the enantiomeric ratio (er) of each compound. Optical rotation ($[\alpha]_D^T$, deg•cm^{3}•g^{-1}•dm^{-1}) was measured on a Jasco DIP-1000 Digital Polarimeter at λ=589 nm in the given solvent at the indicated concentration (*c*, g/100 mL) and temperature (T, °C). X-ray diffraction was performed on Bruker D8 Venture single crystal X-ray diffractometer.

Preparation of ligands

1. Synthetic procedures and characterization data for (*S*)-**L4**

(3'-Bromo-2',6'-diisopropoxy-[1,1'-biphenyl]-2-yl)dicyclohexylphosphane

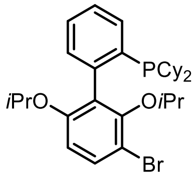

SI-L1

TFA (27.4 g, 241.1 mmol) was added to a solution of RuPhos (15.0 g, 32.1 mmol) in 36 mL DCM, then NBS (5.72 g, 32.1 mmol) was added in ten minutes at 0 °C. After addition was completed, the resulting reaction mixture was stirred at this temperature for 30-45 min. The reaction was quenched with ice water and neutralized with $NaHCO_3$ to pH 9-10.and extracted with DCM three times. The combined organic phases were washed with water and brine, dried over Na_2SO_4, and concentrated in *vacuo* to give the desired product as a white solid which was directly used in the next step without further purification (17.4 g, 99% yield).

Dicyclohexyl(2',6'-diisopropoxy-3'-methyl-[1,1'-biphenyl]-2-yl)phosphane

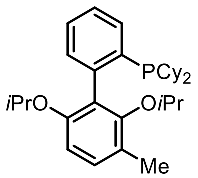

L1

To **SI-L1** (10.9 g, 20.0 mmol), $MeB(OH)_2$ (3.59 g, 60.0 mmol), and $[Pd(allyl)Cl]_2$ (73.1 mg, 0.2 mmol) were added 110 mL dioxane, followed by 2M K_2CO_3 (8.27 g, 60 mmol). Then the resulting reaction mixture was warmed to 90 °C and stirred for 24 h. The reaction was cooled to room temperature and extracted with ethyl acetate (20 mL X 3) for three times. The combined organic phases were washed with brine, dried over Na_2SO_4 and concentrated in *vacuo*. The crude materials were purified by flash column chromatography to give the desired product (8.6 g**,** 90% yield) as a colorless oil.

^{1}H NMR (500 MHz, $CDCl_3$) δ 7.56 (d, *J* = 4.7 Hz, 1H), 7.31 (dd, *J* = 13.4, 9.1 Hz, 2H), 7.26 – 7.20 (m, 1H), 7.09 (d, *J* = 8.3 Hz, 1H), 6.61 (d, *J* = 8.4 Hz, 1H), 4.40 – 4.30 (m, 1H), 3.78 – 3.69 (m, 1H), 2.24 (s, 3H), 1.85-1.75 (m, 2H), 1.71 – 1.54 (m, 12H), 1.32-1.24 (m, 8H), 1.13 (dd, *J* = 6.0, 6.0 Hz, 6H), 0.97 (d, *J* = 6.1 Hz, 3H), 0.76 (d, *J* = 6.1 Hz, 3H). ^{13}C NMR (126 MHz, $CDCl_3$) δ 154.3, 143.7 (d, *J* = 30.8 Hz), 137.0 (d, *J* = 18.7 Hz), 132.1 (d, *J* = 5.9 Hz), 131.9 (d, *J* = 3.9 Hz), 129.7, 127.1, 125.7, 123.1, 108.1, 74.1, 70.0, 34.8 (d, *J* = 15.0 Hz), 33.1 (d, *J* = 13.9 Hz), 30.7 (d, *J* = 15.2 Hz), 30.2 – 28.6 (m), 28.6 – 27.0 (m), 26.6 (d, *J* = 7.6 Hz), 22.7, 22.4 (d, *J* = 3.9 Hz), 22.1, 17.0. ^{31}P NMR (202 MHz, $CDCl_3$) δ -8.91.

(3'-Bromo-2',6'-diisopropoxy-5'-methyl-[1,1'-biphenyl]-2-yl)dicyclohexylphosphane

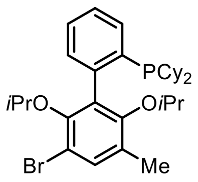

SI-L2

To a solution of **L1** (8.18 g, 17.0 mmol) in 20 mL DCM was added TFA (14.55 g, 127.6 mmol) at 0 °C, then NBS (3.02 g, 17.0 mmol) was added to the solution portionwise in 5 minutes. After addition was completed, the reaction mixture was stirred for 0.5-1h until the reaction was completed. The reaction was quenched with ice water and neutralized with $NaHCO_3$ to pH 9-10 and extracted with DCM (15 mL X 3) for three times. The combined organic phases were washed with water and brine, dried over Na_2SO_4 and concentrated in *vacuo*. The crude product was dissolved in toluene and MeOH was added. The solution was stirred at room temperature for 30 minutes and a white solid precipitated and filtered to give the desired product (8.2 g, 86% yield) as a white solid which was directly used in next step without further purification.

^{1}H NMR (500 MHz, $CDCl_3$) δ 7.64-7.62 (m, 1H), 7.39-7.37 (m, 4H), 3.92-3.88 (m, 1H), 3.73-3.69 (m, 1H), 2.25 (s, 3H), 2.13-2.11 (m, 2H), 1.99-1.97 (m, 2H), 1.80-1.78 (m, 2H), 1.69-1.67 (m, 4H), 1.60-1.58 (m, 2H), 1.35 – 1.16 (m, 8H), 1.10-1.06 (m, 2H), 0.94 (d, *J* = 6.1 Hz, 6H), 0.87 (d, *J* = 5.9 Hz, 6H). ^{13}C NMR (126 MHz, $CDCl_3$) δ 154.1 , 151.4 , 142.3 (d, *J* = 32.1 Hz), 138.4 , 133.4 , 132.6 (d, *J* = 7.0 Hz), 132.2 (d, *J* = 5.9 Hz), 131.7 , 128.1 , 127.5 , 126.9 , 112.0 , 75.8 , 74.9 , 33.8 (dd, *J* = 14.4, 5.8 Hz), 31.7 (dd, *J* = 19.2, 12.5 Hz), 27.7 (dd, *J* = 13.0, 4.9 Hz) , 26.5 , 22.6 , 22.5 (d, *J* = 2.7 Hz), 22.4, 16.8. ^{31}P NMR (202 MHz, $CDCl_3$) δ -8.01. MS (m/z, ESI): Calcd. for Chemical Formula:$C_{31}H_{45}BrO_2P^+$[M+H]$^+$: 559.2335, Found: 559.2333.

(*S*$_S$)-(3'-((l1-oxidanyl)(*p*-tolyl)-l3-sulfanyl)-2',6'-diisopropoxy-5'-methyl-[1,1'-biphenyl]-2-yl)dicyclohexylphosphane

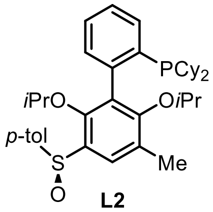

L2

To a solution of **SI-L2** (5.59 g, 10.0 mmol) in anhydrous 28 mL THF was added *n*BuLi (5.5 mL, 2.0 mol/L, 11.0 mmol) as a cyclohexane solution dropwise at -78 °C. After addition was completed, the reaction mixture was stirred at this temperature for 1h. Then this reaction solution was transferred to a solution of (−)-menthyl (*S*$_S$)-*p*-toluenesulfinate (3.82 g, 13.0 mmol) in anhydrous toluene at 0 °C and the resulting reaction mixture was stirred at room temperature overnight. The reaction was quenched with saturated NH_4Cl solution and extracted with ethyl acetate for three times. The combined organic phases were washed with brine, dried over Na_2SO_4 and concentrated in *vacuo*. Based on analysis of the NMR

spectra, the crude material contained two products (1.1:1 dr). The crude material was purified by flash column chromatography with hexane and ethyl acetate (10:1-3:1) to give the two diastereomers.

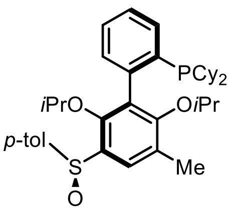

(S_S, *S*)-**L2**

(S_S , *S*)-**L2**: White solid, 2.0 g, 32% yield. $[\alpha]_D^{20}$ = -149.0 (*c* 0.005 $CHCl_3$). ^{1}H NMR (500 MHz, $CDCl_3$) δ 7.65-7.59 (m, 4H), 7.37-7.36 (m, 3H), 7.26-7.24 (m, 2H), 3.88-3.86 (m, 1H), 3.84 – 3.74 (m, 1H), 2.38 (s, 3H), 2.30 (s, 3H), 1.85-1.83 (m, 3H), 1.76-1.71 (m, 4H), 1.68 – 1.56 (m, 4H), 1.37-1.32 (m, 2H), 1.18-1.13 (m, 10H), 1.01 – 0.81 (m, 11H). ^{13}C NMR (126 MHz, $CDCl_3$) δ 157.3 , 151.3 , 142.7 , 142.2 , 141.9 , 140.6 , 138.2 (d, *J* = 19.8 Hz), 132.8 , 132.4 , 131.9 (d, *J* = 5.7 Hz), 129.7 , 128.5 , 127.6 , 127.1 , 125.8 , 125.6 , 75.6 , 74.9 , 34.3 (d, *J* = 15.4 Hz), 34.0 (d, *J* = 15.2 Hz), 31.4 (d, *J* = 18.6 Hz), 30.4 (d, *J* = 14.3 Hz), 29.9 (d, *J* = 15.5 Hz), 28.5 (d, *J* = 8.9 Hz), 27.8 (d, *J* = 12.8 Hz), 27.3 (dd, J = 28.1, 9.9 Hz), 26.5 (d, J = 10.2 Hz), 22.8, 22.6, 22.4, 22.0, 21.4, 17.4. ^{31}P NMR (202 MHz, CDCl3) δ -8.40. MS (m/z, ESI): Calcd. for Chemical Formula:$C_{31}H_{45}O_2P^+$[M+Na]$^+$: 641.3189, Found: 641.3188.

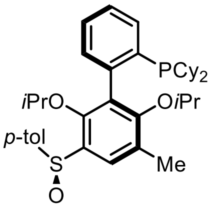

(S_S, *R*)-**L2**

(S_S , *R*)-**L2** : White solid, 2.3g , 37% yield. $[\alpha]_D^{20}$ = -195.0 (*c* 0.005 $CHCl_3$). ^{1}H NMR (500 MHz, $CDCl_3$) δ 7.76 (d, *J* = 8.8 Hz, 1H), 7.67 (d, *J* = 8.2 Hz, 2H), 7.51 (d, *J* = 5.6 Hz, 1H), 7.33 (p, *J* = 6.7 Hz, 2H), 7.23 (t, *J* = 6.4 Hz, 3H), 6.83 (d, *J* = 8.9 Hz, 1H), 4.55 – 4.48 (m, 1H), 3.72 – 3.64 (m, 1H), 2.38 (s, 3H), 1.90-1.86 (m, 2H), 1.78 – 1.39 (m, 12H), 1.25 – 0.98 (m, 17H), 0.85 (s, 3H). ^{13}C NMR (126 MHz, $CDCl_3$) δ 158.6 , 152.9 , 143.3 , 142.1 , 141.9 , 140.3 , 137.0 , 136.8 , 132.4 , 131.5 (d, *J* = 5.3 Hz), 129.7 , 129.4 , 127.6 , 126.5 , 126.1 (d, *J* = 3.4 Hz), 124.4 , 108.0 , 75.1 , 69.9 , 35.3 (d, *J* = 16.2 Hz), 32.8 (d, *J* = 14.6 Hz), 30.5 – 29.4 (m), 28.7 (d, *J* = 6.4 Hz), 27.5 (d, *J* = 6.4 Hz), 27.4, 27.1 (t, *J* = 3.4 Hz), 26.5 (d, *J* = 19.4 Hz), 22.7 , 22.1, 21.9 (d, *J* = 14.6 Hz), 21.4. ^{31}P NMR (202 MHz, $CDCl_3$) δ -8.82. MS (m/z, ESI): Calcd. for Chemical Formula:$C_{31}H_{45}O_2P^+$[M+Na]$^+$: 641.3189, Found: 641.3188.

(*R*)-dicyclohexyl(2',6'-diisopropoxy-3'-methyl-[1,1'-biphenyl]-2-yl)phosphane

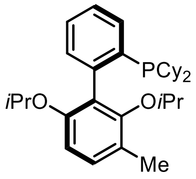

(*R*)-**L1**

Under N_2 atmosphere, *n*BuLi (9.9 mL, 2.0 mol/L, 19.8 mmol) as a cyclohexane solution was added to (S_S , *S*)-**L2** (2.0 g, 3.2 mmol) in anhydrous 20.0 mL THF at -78 °C. Then the resulting reaction mixture was stirred at room temperature for 1-2 h and MeOH (1.0 mL) was added to this reaction mixture at -78 °C. The reaction was quenched with saturated NH_4Cl solution and extracted with ethyl acetate (10 mL X 3) three times. The combined organic phases were washed with brine, dried over Na_2SO_4, and concentrated in *vacuo*. The crude materials were purified by flash column chromatography to give the product a colorless oil. 1.5 g, 98% yield. $[\alpha]_D^{20}$ = -18.36 (*c* 0.050 $CHCl_3$) for >99.5: 0.5 er. The NMR spectra are consistent with racemic **L1**.

HPLC analysis of this compound: Daicel Chiralpak IA, hexane/iso-propanol = 95: 5, 1.0 mL/min, λ = 296 nm, retention time: 8.37 min (major) and 9.31 min (minor).

(*S*)-(3'-bromo-2',6'-diisopropoxy-5'-methyl-[1,1'-biphenyl]-2-yl)dicyclohexylphosphane

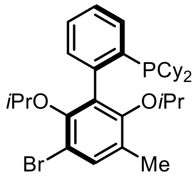

(*S*)-**SI-L2**

(*R*)-**L1** (1.5 g, 3.1 mmol) was dissolved in 4 mL DCM and TFA (2.81 g, 24.6 mmol) was added at 0 °C. After stirring for 5 min, NBS (0.59 g, 3.2 mmol) was added portion-wise at 0 °C. After addition was completed, the reaction mixture was stirred for 0.5-1h until the reaction was completed. The reaction was quenched with ice water and neutralized with $NaHCO_3$ to pH 9-10 and extracted with DCM (8 mL X 3) for three times. The combined organic phases were washed with water and brine, dried over Na_2SO_4, and concentrated in *vacuo*. The crude product was dissolved in 1.0 mL toluene and 8.0 mL MeOH was added. The solution was stirred at room temperature for 30 minutes and a white solid precipitated and filtrated to give the desired product 1.24 g in 72% yield as a white solid which was directly used in the next step without further purification. $[\alpha]_D^{20}$ = +6.00 (*c* 0.050 $CHCl_3$). The NMR spectra are consistent with racemic **SI-L2**.

Diethyl (*S*)-(2'-(dicyclohexylphosphanyl)-2,6-diisopropoxy-5-methyl-[1,1'-biphenyl]-3-yl)phosphonate

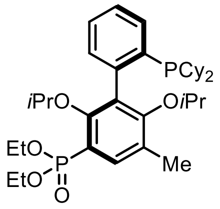

(*S*)-**L3**

Under N_2 atmosphere, to a solution of (*S*)-**SI-L2** (1.2 g, 2.1 mmol) in anhydrous 3.0 mL THF was added *n*BuLi (1.2 mL, 2.0 mol/L, 2.35 mmol) dropwise as a cyclohexane solution at -78 °C. The reaction mixture was stirred at this temperature for 1 h and ethyl chlorophosphate (0.44 g, 2.6 mmol) was added dropwise. The reaction was quenched with saturated NH_4Cl solution and extracted with ethyl acetate (8 mL X 3) for three times. The combined organic phases were washed with brine, dried over Na_2SO_4 and concentrated in *vacuo*. The crude materials were purified by flash column chromatography with hexane and ethyl acetate (10:1-3:1) to give the desired products (*S*)-**L3** 1.08 g in 82% yield as a colorless oil.

$[\alpha]_D^{20}$ = +22.00 (*c* 0.100 $CHCl_3$). ^1H NMR (500 MHz, $CDCl_3$) δ 7.67-7.61 (m, 2H), 7.34 (d, *J* = 2.7 Hz, 3H), 4.24 – 4.15 (m, 3H), 4.12-4.10 (m, 1H), 3.86 – 3.76 (m, 1H), 3.73-3.70 (m, 1H), 2.25 (s, 3H), 2.02 – 1.93 (m, 2H), 1.88-1.84 (m, 2H), 1.76-1.74 (m, 2H), 1.69 – 1.60 (m, 5H), 1.48 (d, *J* = 12.8 Hz, 1H), 1.38 (t, *J* = 7.0 Hz, 3H), 1.34 (t, *J* = 7.1 Hz, 3H), 1.26-1.18 (m, 10H), 0.97 (dd, *J* = 5.8, 3.0 Hz, 6H), 0.90 (d, *J* = 6.1 Hz, 3H), 0.87 (d, *J* = 6.1 Hz, 3H). ^{13}C NMR (126 MHz, $CDCl_3$) δ 158.7 , 156.8 , 142.5 , 138.3 (d, *J* = 19.4 Hz), 135.8 (d, *J* = 7.6 Hz), 132.3 , 131.9 (d, *J* = 5.9 Hz), 131.0 , 127.6 , 126.9 , 126.1 (d, *J* = 15.3 Hz), 117.5 , 74.8 , 74.0 , 61.8 (d, *J* = 5.7 Hz), 61.6 (d, *J* = 5.3 Hz), 34.3 (d, *J* = 15.4 Hz), 33.9 (d, *J* = 15.0 Hz), 31.3 (dd, *J* = 33.6, 17.2 Hz), 29.3 (d, *J* = 12.1 Hz), 29.0 (d, *J* = 11.0 Hz), 27.6 (dd, *J* = 14.2, 5.4 Hz), 27.3 (t, *J* = 5.4 Hz), 26.5 (d, *J* = 9.2 Hz), 22.5 (t, *J* = 12.1 Hz), 22.4, 21.6 , 17.1 , 16.5 (dd, *J* = 11.3, 6.9 Hz). ^{31}P NMR (202 MHz, $CDCl_3$) δ 19.27, -8.29. HRMS (m/z, ESI): Calcd. for Chemical Formula: $C_{35}H_{55}O_5P_2^+$ $[M+H]^+$: 617.3525, Found: 617.3531.

Ethyl hydrogen ((*S*)-2'-(dicyclohexylphosphanyl)-2,6-diisopropoxy-5-methyl-[1,1'-biphenyl]-3-yl)phosphonate

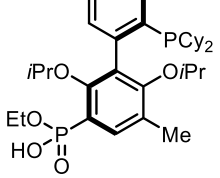

(*S*)-**L4**

Under N_2 atmosphere, (*S*)-**L3** (0.5 g, 0.81 mmol) was dissolved in 1.0 mL THF and 2.0 mL EtOH, followed by addition of 2M NaOH (65 mg, 1.62 mmol).[1] The resulting reaction mixture was warmed to 75 °C and stirred for 3-4 days. After the reaction was completed, removal of the organic phase was performed under reduced pressure and water was added and extracted with ethyl ether and hexane (5.0 mL, 4:1 v/v) for three times. The water phase was neutralized with 1 M HCl to pH 2-3 and extracted with ethyl acetate (5 mL X 3) for three times. The combined organic phases were washed with

water and brine, dried over Na_2SO_4 and concentrated in *vacuo* to give the desired ligands without further purification. 0.34 g in 72% yield as a white solid.

$[\alpha]_D^{20}$ = +44.00 (*c* 0.010 $CHCl_3$). ^{1}H NMR (500 MHz, $CDCl_3$) δ 7.71 (d, *J* = 15.3 Hz, 1H), 7.65 – 7.60 (m, 1H), 7.37 (td, *J* = 6.8, 3.5 Hz, 3H), 6.30 (s, 1H), 4.20 – 4.09 (m, 2H), 3.82-3.78 (m, 1H), 3.73-3.70 (m, 1H), 2.26 (s, 3H), 1.20-1.18 (m, 2H), 1.89 (s, 1H), 1.84 – 1.58 (m, 8H), 1.50 – 1.45 (m, 1H), 1.37-1.33 (m, 3H), 1.31 – 1.14 (m, 10H), 1.00 (d, *J* = 6.1 Hz, 3H), 0.96 (d, *J* = 6.1 Hz, 3H), 0.92 (d, *J* = 6.2 Hz, 3H), 0.86 (d, *J* = 6.2 Hz, 3H). ^{13}C NMR (126 MHz, $CDCl_3$) δ 158.5 , 156.5 , 142.6 , 142.4 , 135.4 , 132.1 (d, *J* = 2.6 Hz), 132.0 (d, *J* = 6.5 Hz), 130.7 , 127.7 , 127.0 , 126.2 (d, *J* = 15.7 Hz), 118.3 , 116.8 , 74.9 , 74.3 , 61.7 (d, *J* = 5.7 Hz), 33.9 (dd, *J* = 32.6, 14.4 Hz), 31.8 (d, *J* = 19.4 Hz), 31.0 (d, *J* = 16.1 Hz), 29.7 , 29.3 (d, *J* = 12.2 Hz), 28.6 (d, *J* = 8.5 Hz), 27.9 – 26.8 (m), 26.3 (d, *J* = 7.8 Hz), 22.48 (dd, *J* = 19.9, 10.3 Hz), 22.2 , 21.5 , 17.0 , 16.4 (d, *J* = 7.2 Hz). ^{31}P NMR (202 MHz, $CDCl_3$) δ 20.58, -8.87. HRMS (m/z, ESI): Calcd. for Chemical Formula: $C_{33}H_{51}O_5P_2^+$ $[M+H]^+$: 589.3212, Found: 589.3215.

2. (*S*)-**L5** was prepared starting from SPhos following an analogues route.

Diethyl (*S*)-(2'-(dicyclohexylphosphanyl)-2,6-dimethoxy-5-methyl-[1,1'-biphenyl]-3-yl)phosphonate

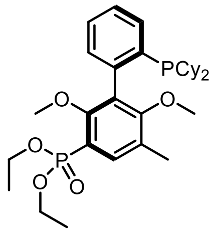

(*S*)-**SI-L3**

Prepared from SPhos following an analogous synthetic route. White solid, $[\alpha]_D^{20}$ = +24.00 (*c* 0.010 $CHCl_3$) for 98.5:1.5 er. ^{1}H NMR (500 MHz, $CDCl_3$) δ 7.67 (d, *J* = 14.4 Hz, 1H), 7.65 – 7.59 (m, 1H), 7.39 (dd, *J* = 5.4, 3.4 Hz, 2H), 7.36 – 7.31 (m, 1H), 4.23 – 4.09 (m, 4H), 3.45 (s, 3H), 3.33 (s, 3H), 2.30 (s, 3H), 1.93 – 1.81 (m, 2H), 1.79 – 1.60 (m, 8H), 1.55-1.53 (m, 2H), 1.40 – 1.32 (m, 6H), 1.29 – 1.00 (m, 10H). ^{13}C NMR (126 MHz, $CDCl_3$) δ 160.9 , 159.1 , 141.5 (d, *J* = 32.1 Hz), 137.5 (d, *J* = 19.9 Hz), 136.0 (d, *J* = 7.8 Hz), 132.7 , 131.5 (d, *J* = 5.9 Hz), 127.7 , 126.9 , 126.3 (d, *J* = 15.4 Hz), 117.4 , 115.9, 62.1 (d, *J* = 5.5 Hz), 61.9 (d, *J* = 4.5 Hz), 61.0, 60.0, 35.3 (d, *J* = 14.8 Hz), 34.4 (d, *J* = 14.3 Hz), 30.6 – 29.6 (m), 28.0 – 27.1 (m), 26.5 (d, *J* = 11.9 Hz), 16.5 (dd, *J* = 14.9, 8.7 Hz). ^{31}P NMR (202 MHz, $CDCl_3$) δ 17.85, -8.62. HRMS (m/z, ESI): Calcd. for Chemical Formula: $C_{31}H_{47}O_5P_2^+$ $[M+H]^+$: 561.2899, Found: 561.2885.

HPLC analysis of this compound: Daicel Chiralpak IH, hexane/iso-propanol = 90: 10, 1.0 mL/min, λ = 272 nm, retention time: 12.27 min (major) and 11.89 min (minor).

Ethyl hydrogen ((*S*)-2'-(dicyclohexylphosphanyl)-2,6-dimethoxy-5-methyl-[1,1'-biphenyl]-3-yl)phosphonate

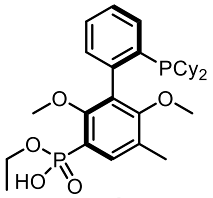

(*S*)-**L5**

Prepared from (*S*)-**SI-L3** in 64% yield. White solid, $[\alpha]_D^{20}$ = +46.00 (*c* 0.020 $CHCl_3$). ^{1}H NMR (500 MHz, $CDCl_3$) δ 7.71 (d, *J* = 14.8 Hz, 1H), 7.67 – 7.61 (m, 1H), 7.40 (dd, *J* = 9.2, 4.2 Hz, 2H), 7.39 – 7.35 (m, 1H), 5.26 (s, 1H), 4.18 – 4.10 (m, 2H), 3.43 (s, 3H), 3.35 (s, 3H), 2.29 (s, 3H), 1.88 – 1.60 (m, 10H), 1.58-1.56 (m, 2H), 1.35 (t, *J* = 7.1 Hz, 3H), 1.27 – 1.08 (m, 10H). ^{13}C NMR (126 MHz, $CDCl_3$) δ 160.7, 159.0, 141.5, 141.3, 135.7 (d, *J* = 8.0 Hz), 132.6, 131.7 (d, *J* = 6.1 Hz), 130.6, 127.9, 127.0, 126.3 (d, *J* = 15.6 Hz), 118.5, 116.9, 62.0 (d, *J* = 4.4 Hz), 61.1, 59.9, 35.0 (d, *J* = 12.5 Hz), 34.4 (d, *J* = 11.9 Hz), 30.3 (t, *J* = 12.6 Hz), 29.69 (t, *J* = 12.8 Hz), 27.90 – 27.08 (m), 26.4 (d, *J* = 3.6 Hz), 16.4 (d, *J* = 7.8 Hz), 16.3. ^{31}P NMR (202 MHz, $CDCl_3$) δ 19.56, -7.71. HRMS (m/z, ESI): Calcd. for Chemical Formula: $C_{29}H_{43}O_5P_2^+$ $[M+H]^+$: 533.2586, Found: 533.2588.

Preparation of substrates

1. Unless otherwise noted, the general procedure for synthesis of fluorene substrates was described below. [2]

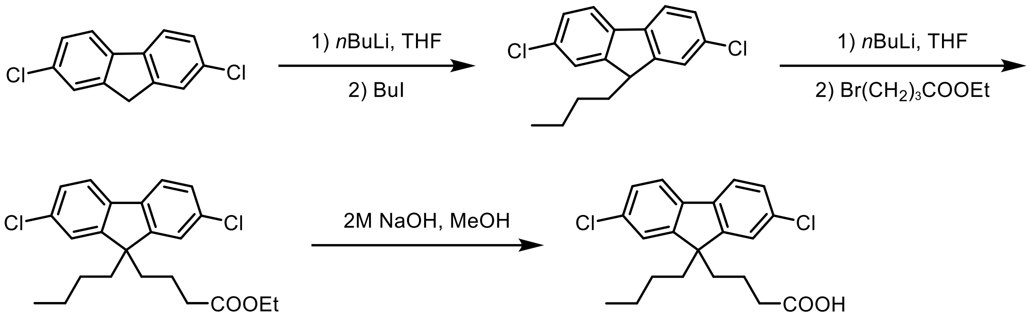

9-butyl-2,7-dichloro-9H-fluorene

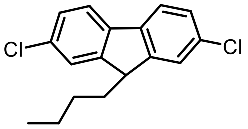

Under N_2 atmosphere, 2,7-dichlorofluorene (6.5 g, 27.6 mmol) was dissolved in anhydrous 30 mL THF, then *n*BuLi (14.5 mL, 2.0 mol/L, 29.0 mmol) as a cyclohexane solution was added dropwise at -78 ^oC. After addition was completed, the resulting reaction mixture was stirred for 40 min at room temperature. 1-iodobutane (6.1 g, 33.1 mmol) was added in one portion at -78 ^oC and stirring at this temperature for 20 min. Then the reaction mixture was warmed to room temperature and stirring for 15 h. The reaction was quenched with saturated NH_4Cl solution and extracted with ethyl acetate (20 X 3 mL) three times. The combined organic phases were washed with brine, dried over Na_2SO_4, and concentrated in *vacuo*. The crude materials were purified by flash column chromatography to give the desired product 7.41 g in 92 % yield as a white solid.

ethyl 4-(9-butyl-2,7-dichloro-9H-fluoren-9-yl)butanoate (53)

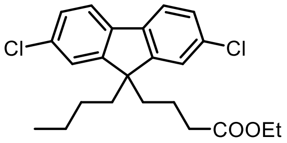

Under N_2 atmosphere, 9-butyl-2,7-dichloro-9H-fluorene (2.6 g, 8.9 mmol) was dissolved in anhydrous 20 mL THF and *n*BuLi (4.9 mL, 2.0 mol/L, 9.8 mmol) as a cyclohexane solution was added dropwise at -78 ^oC. After addition was completed, the reaction mixture was stirring for 30 min at room temperature and ethyl 4-bromobutanoate (2.09 g, 10.7 mmol) was

added in one portion. The resulting reaction mixture was warmed to room temperature and stirred overnight. The reaction was quenched with saturated NH_4Cl solution and extracted with ethyl acetate (15 mL X 3) three times. The combined organic phases were washed with brine, dried over Na_2SO_4, and concentrated in *vacuo*. The crude materials were purified by flash column chromatography to give the desired product 3.13 g in 86 % yield as a white solid.

^{1}H NMR (500 MHz, $CDCl_3$) δ 7.59 (d, *J* = 8.0 Hz, 2H), 7.33 (dd, *J* = 8.0, 1.9 Hz, 2H), 7.31 (d, *J* = 1.7 Hz, 2H), 4.06 (q, *J* = 7.1 Hz, 2H), 2.08 (t, *J* = 7.4 Hz, 2H), 2.03 – 1.98 (m, 2H), 1.97 – 1.92 (m, 2H), 1.22 (t, *J* = 7.1 Hz, 3H), 1.13-1.09 (m, 2H), 0.99 – 0.93 (m, 2H), 0.71 (t, *J* = 7.4 Hz, 3H), 0.62 – 0.56 (m, 2H). ^{13}C NMR (126 MHz, $CDCl_3$) δ 173.2, 151.7, 138.6, 133.3, 127.6, 123.3, 120.9, 60.3, 55.3, 40.1, 39.38, 34.3, 25.7, 22.9, 19.3, 14.2, 13.8. MS (m/z, ESI): Calcd. for Chemical Formula: $C_{23}H_{25}Cl_2O_2^-$ [M+Na]$^-$: 427.1202, Found: 427.1201.

4-(9-butyl-2,7-dichloro-9H-fluoren-9-yl)butanoic acid (51)

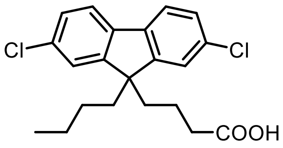

Ethyl 4-(9-butyl-2,7-dichloro-9H-fluoren-9-yl)butanoate (1.5 g, 3.7 mmol) was dissolved in 5.0 mL THF and 5.0 mL MeOH, and 8M NaOH (0.6 g, 14.8 mmol, 1.8 mL) was added. The reaction mixtures were stirred at room temperature for 2 h and solvents were removed under reduced pressure. Water was added and the aqueous solution was acidified to pH 2-3 with 1M HCl solution. The reaction was extracted with ethyl acetate (10 mL X 3) three times. The combined organic phases were washed with brine, dried over Na_2SO_4, and concentrated in *vacuo*. The crude materials were purified by flash column chromatography to give the desired product. The product was recrystallized in ethyl acetate and hexane to give the desired products **51** 0.97 g in 69% yield as a white solid.

^{1}H NMR (500 MHz, $CDCl_3$) δ 7.59 (d, *J* = 8.0 Hz, 2H), 7.33 (dd, *J* = 8.0, 1.9 Hz, 2H), 7.31 (d, *J* = 1.6 Hz, 2H), 2.14-2.11 (m, 2H), 2.04 – 1.99 (m, 2H), 1.97 – 1.92 (m, 2H), 1.14-1.10 (m, 2H), 0.96-0.92 (m, 2H), 0.71 (t, *J* = 7.4 Hz, 3H), 0.62 – 0.55 (m, 2H). ^{13}C NMR (126 MHz, $CDCl_3$) δ 178.4, 151.6, 138.6, 133.4, 127.6, 123.3, 120.9, 55.3, 40.0, 39.3, 33.8, 25.7, 22.9, 19.1, 13.8. MS (m/z, ESI): Calcd. for Chemical Formula: $C_{21}H_{21}Cl_2O_2$ $^-$[M-H]$^-$:375.0924, Found: 375.0930.

2-(9-butyl-2,7-dichloro-9H-fluoren-9-yl)acetic acid

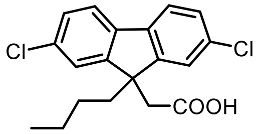

Prepared from 9-butyl-2,7-dichloro-9H-fluorene in two steps, 68% yield, white solid. ^{1}H NMR (500 MHz, $CDCl_3$) δ 7.61 – 7.56 (m, 2H), 7.38 (d, *J* = 1.6 Hz, 2H), 7.35 (dd, *J* = 8.1, 1.9 Hz, 2H), 2.90 (s, 2H), 2.07 – 2.01 (m, 2H), 1.11 (dt, *J* = 14.7, 7.4 Hz, 2H), 0.71 (t, *J* = 7.4 Hz, 3H), 0.61 – 0.54 (m, 2H). ^{13}C NMR (126 MHz, $CDCl_3$) δ 174.5, 150.5, 138.3, 133.3, 128.0, 123.6, 120.9, 52.4, 43.1, 38.8, 25.6, 22.7, 13.7. MS (m/z, ESI): Calcd. for Chemical Formula: $C_{19}H_{17}Cl_2O_2^-$ [M-H]$^-$: 347.0611, Found:347.0613.

3-(9-butyl-2,7-dichloro-9H-fluoren-9-yl)propanoic acid

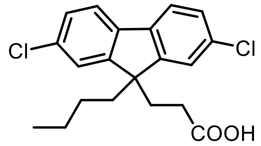

Prepared from 9-butyl-2,7-dichloro-9H-fluorene in two steps, 76% yield, white solid. ^{1}H NMR (300 MHz, $CDCl_3$) δ 7.62 (d, *J* = 8.0 Hz, 2H), 7.42 – 7.31 (m, 4H), 2.42 – 2.27 (m, 2H), 2.05 – 1.94 (m, 2H), 1.68 – 1.53 (m, 2H), 1.16-1.12 (m, 2H), 0.73 (t, *J* = 7.4 Hz, 3H), 0.68 – 0.48 (m, 2H). ^{13}C NMR (75 MHz, $CDCl_3$) δ 179.2, 150.5, 138.6, 133.5, 127.9, 123.3, 120.9, 54.6, 39.8, 34.2, 28.6, 25.7, 22.8, 13.7. MS (m/z, ESI): Calcd. for Chemical Formula: $C_{20}H_{19}Cl_2O_2^-$ [M-H]$^-$:361.0768, Found: 361.0761.

(*E*)-4-(9-butyl-2,7-dichloro-9H-fluoren-9-yl)but-2-enoic acid

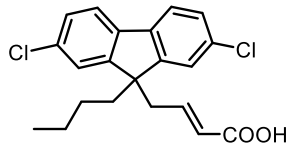

Prepared from 9-butyl-2,7-dichloro-9H-fluorene in two steps, 46% yield, white solid. ^{1}H NMR (500 MHz, $CDCl_3$) δ 7.59 (d, *J* = 8.0 Hz, 2H), 7.34 (dd, *J* = 8.1, 1.9 Hz, 2H), 7.32 (d, *J* = 1.6 Hz, 2H), 6.32 (dt, *J* = 15.3, 7.6 Hz, 1H), 5.59 (d, *J* = 15.5 Hz, 1H), 2.83-2.79 (m, 2H), 2.03 – 1.96 (m, 2H), 1.15-1.11 (m, 2H), 0.72 (t, *J* = 7.4 Hz, 3H), 0.64 – 0.56 (m, 2H). ^{13}C NMR (126 MHz, $CDCl_3$) δ 170.2, 150.5, 146.0, 138.3, 133.5, 128.0, 123.4, 121.1, 54.8, 42.7, 39.1, 25.7, 22.8, 13.7. MS (m/z, ESI): Calcd. for Chemical Formula: $C_{21}H_{19}Cl_2O_2^-$ [M-H]$^-$: 373.0768, Found: 373.0766.

5-(9-butyl-2,7-dichloro-9H-fluoren-9-yl)pentanoic acid

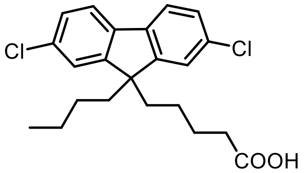

Prepared from 9-butyl-2,7-dichloro-9H-fluorene in two steps, 64% yield, white solid. ^{1}H NMR (500 MHz, $CDCl_3$) δ 7.59 (d, *J* = 8.0 Hz, 2H), 7.35 – 7.29 (m, 4H), 2.18 – 2.09 (m, 2H), 1.99-1.93 (m, 4H), 1.48 – 1.39 (m, 2H), 1.13-1.09 (m, 2H), 0.71 (t, *J* = 7.4 Hz, 3H), 0.68 – 0.62 (m, 2H), 0.62 – 0.55 (m, 2H). ^{13}C NMR (126 MHz, $CDCl_3$) δ 179.5, 151.9, 138.6, 133.3, 127.5, 123.2,

120.8, 55.4, 40.0, 39.8, 33.6, 25.8, 24.8, 23.2, 22.9, 13.8. MS (m/z, ESI): Calcd. for Chemical Formula: $C_{22}H_{23}Cl_2O_2^-$ [M-H]$^-$: 389.1081, Found: 389.1076.

6-(9-butyl-2,7-dichloro-9H-fluoren-9-yl)hexanoic acid

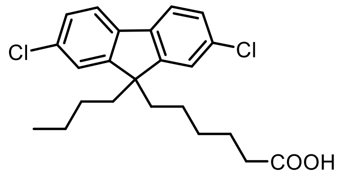

Prepared from 9-butyl-2,7-dichloro-9H-fluorene in two steps, 56% yield, white solid. ^{1}H NMR (300 MHz, $CDCl_3$) δ 7.61 (d, *J* = 8.0 Hz, 2H), 7.36-7.30 (m, 4H), 2.20 (t, *J* = 7.4 Hz, 2H), 2.00-1.94 (m, 4H), 1.51 – 1.37 (m, 2H), 1.23 – 1.08 (m, 4H), 0.73 (t, *J* = 7.3 Hz, 3H), 0.65-0.60 (m, 4H). ^{13}C NMR (75 MHz, $CDCl_3$) δ 179.5, 152.0, 138.5, 133.2, 127.3, 123.1, 120.7, 55.4, 39.9, 33.7, 29.2, 25.7, 24.2, 23.3, 22.9, 13.7. MS (m/z, ESI): Calcd. for Chemical Formula: $C_{23}H_{25}Cl_2O_2^-$ [M-H]$^-$: 403.1237, Found: 403.1242.

7-(9-butyl-2,7-dichloro-9H-fluoren-9-yl)heptanoic acid

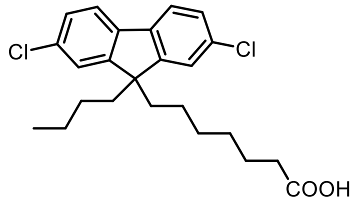

Prepared from 9-butyl-2,7-dichloro-9H-fluorene in two steps, 61% yield, white solid. ^{1}H NMR (500 MHz, $CDCl_3$) δ 7.59 (d, *J* = 8.0 Hz, 2H), 7.33 (dd, *J* = 8.0, 1.9 Hz, 2H), 7.30 (d, *J* = 1.6 Hz, 2H), 2.25 (t, *J* = 7.5 Hz, 2H), 1.97 – 1.91 (m, 4H), 1.50 – 1.43 (m, 2H), 1.15 – 1.07 (m, 6H), 0.71 (t, *J* = 7.4 Hz, 3H), 0.64 – 0.56 (m, 4H). ^{13}C NMR (126 MHz, $CDCl_3$) δ 179.4, 152.2, 138.6, 133.2, 127.4, 123.2, 120.8, 55.5, 40.2, 40.0, 29.5, 28.7, 25.8, 24.5, 23.5, 22.9, 13.8. MS (m/z, ESI): Calcd. for Chemical Formula: $C_{24}H_{27}Cl_2O_2$ [M-H]: 417.1394, Found: 417.1395.

8-(9-butyl-2,7-dichloro-9H-fluoren-9-yl)octanoic acid

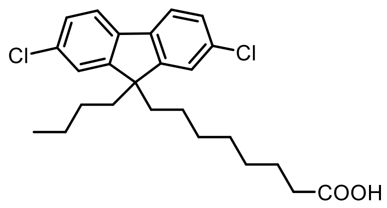

Prepared from 9-butyl-2,7-dichloro-9H-fluorene in two steps, 66% yield, light green oil. ^{1}H NMR (500 MHz, $CDCl_3$) δ 7.62 – 7.54 (m, 2H), 7.33 (dd, *J* = 8.0, 1.9 Hz, 2H), 7.30 (d, *J* = 1.5 Hz, 2H), 2.28 (t, *J* = 7.5 Hz, 2H), 1.97 – 1.90 (m, 4H), 1.56 – 1.51 (m, 2H), 1.21 – 1.15 (m, 2H), 1.13 – 1.05 (m, 6H), 0.71 (t, *J* = 7.4 Hz, 3H), 0.62-0.58 (m, 4H). ^{13}C NMR (126 MHz, $CDCl_3$) δ 179.1, 152.3, 138.6, 133.2, 127.3, 123.2, 120.7, 55.5, 40.2, 40.1, 33.9, 33.8, 32.7, 28.9, 28.8_1, 28.8_2, 28.4, 27.9, 25.8, 24.5, 23.6, 22.9, 13.8. MS (m/z, ESI): Calcd. for Chemical Formula: $C_{25}H_{29}Cl_2O_2^-$ [M-H]$^-$: 431.1550, Found: 431.1552.

4-(2,7-dichloro-9-methyl-9H-fluoren-9-yl)butanoic acid

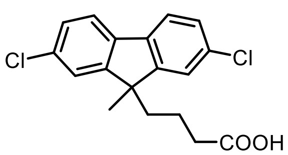

Prepared from 2,7-dichlorofluorene in three steps, 48% yield, white solid. ^{1}H NMR (500 MHz, $CDCl_3$) δ 7.60 (d, *J* = 7.9 Hz, 2H), 7.36 – 7.32 (m, 4H), 2.15 (t, *J* = 7.4 Hz, 2H), 2.05 – 2.00 (m, 2H), 1.47 (s, 3H), 1.02-0.95 (m, 2H). ^{13}C NMR (126 MHz, $CDCl_3$) δ 178.1, 152.9, 137.7, 133.4, 127.7, 123.3, 121.1, 51.0, 39.5, 33.7, 26.6, 19.5. MS (m/z, ESI): Calcd. for Chemical Formula: $C_{18}H_{15}Cl_2O_2^-$ [M-H]$^-$: 333.0455, Found: 333.0453.

4-(2,7-dichloro-9-octyl-9H-fluoren-9-yl)butanoic acid

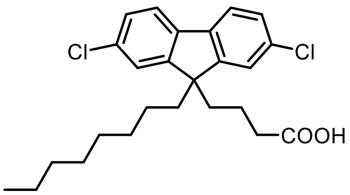

Prepared from 2,7-dichlorofluorene in three steps, 36% yield, white solid. ^{1}H NMR (500 MHz, $CDCl_3$) δ 7.59 (d, *J* = 8.1 Hz, 2H), 7.33 (dd, *J* = 8.1, 1.9 Hz, 2H), 7.30 (d, *J* = 1.6 Hz, 2H), 2.12 (t, *J* = 7.4 Hz, 2H), 2.04 – 1.99 (m, 2H), 1.96 – 1.91 (m, 2H), 1.26 – 1.20 (m, 2H), 1.17 – 1.04 (m, 8H), 0.96-0.92 (m, 2H), 0.85 (t, *J* = 7.2 Hz, 3H), 0.62-0.58 (m, 2H). ^{13}C NMR (126 MHz, $CDCl_3$) δ 178.2, 151.6, 138.6, 133.4, 127.6, 123.3, 120.9, 55.3, 40.2, 39.3, 33.7, 31.8, 29.8, 29.2, 29.1 23.5, 22.6, 19.1, 14.1. MS (m/z, ESI): Calcd. for Chemical Formula: $C_{25}H_{29}Cl_2O_2^-$ [M-H]$^-$: 431.1550, Found: 431.1547.

4-(9-benzyl-2,7-dichloro-9H-fluoren-9-yl)butanoic acid

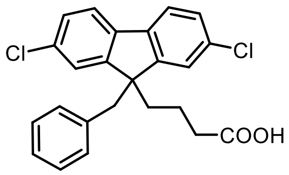

Prepared from 2,7-dichlorofluorene in three steps, 62% yield, white solid. ^{1}H NMR (500 MHz, $CDCl_3$) δ 7.47 – 7.41 (m, 2H), 7.31 – 7.26 (m, 4H), 7.09 – 6.98 (m, 3H), 6.69 – 6.62 (m, 2H), 3.14 (s, 2H), 2.22-2.17 (m, 4H), 1.01 – 0.92 (m, 2H). ^{13}C NMR (126 MHz, $CDCl_3$) δ 179.0, 150.4, 138.5, 136.0, 133.0, 130.2, 127.8, 127.4, 126.3, 124.1, 120.9, 56.1, 46.7, 37.7, 33.8, 19.1. MS (m/z, ESI): Calcd. for Chemical Formula: $C_{24}H_{19}Cl_2O_2^-$ [M-H]$^-$: 409.0768, Found: 409.0769.

4-(2,7-dichloro-9-phenyl-9H-fluoren-9-yl)butanoic acid

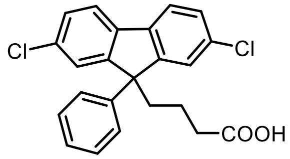

Prepared from 2,7-dichloro-9-phenyl-9H-fluorene[3] in two steps, 42% yield, white solid. ^{1}H NMR (500 MHz, $CDCl_3$) δ 7.66 – 7.64 (m, 2H), 7.35 (dd, *J* = 8.1, 1.9 Hz, 2H), 7.28 – 7.21 (m, 3H), 7.18 (d, *J* = 1.6 Hz, 2H), 7.15 – 7.11 (m, 2H), 2.63 – 2.45 (m, 2H), 2.26 (t, *J* = 7.3 Hz, 2H), 1.11-1.05 (m, 2H). ^{13}C NMR (126 MHz, $CDCl_3$) δ 178.3, 152.9, 143.0, 138.2, 133.7, 128.7, 128.0, 127.0, 126.4, 124.7, 121.1, 58.7, 36.7, 33.7, 19.2. MS (m/z, ESI): Calcd. for Chemical Formula: $C_{23}H_{17}Cl_2O_2^-$ [M-H]$^-$: 395.0611, Found: 395.0605.

2. Substrates bearing sulfo group were prepared following the procedure shown below. [4]

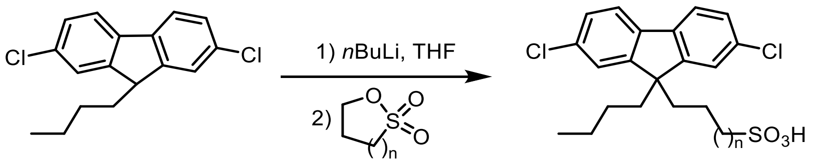

3-(9-butyl-2,7-dichloro-9H-fluoren-9-yl)propane-1-sulfonic acid

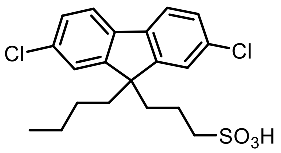

Under N_2 atmosphere, 9-butyl-2,7-dichloro-9H-fluorene (0.5 g, 1.7 mmol) was dissolved in anhydrous 1.5 mL THF and *n*BuLi (0.95 mL, 2.0 mol/L, 1.9 mmol) as a cyclohexane solution was added dropwise at -78 °C. After addition was completed, the reaction mixture was stirring for 30 min at room temperature and ethyl 1,2-oxathiolane 2,2-dioxide (0.25 g, 2.1 mmol) was added in one portion. The resulting reaction mixture was warmed to room temperature and stirred overnight. Water was added and the aqueous solution was acidified to pH 2-3 with 1M HCl solution and extracted with ethyl acetate (10 mL X 3) three times. The combined organic phases were washed with brine, dried over Na_2SO_4, and concentrated in *vacuo*. The crude materials were purified by flash column chromatography to give the desired product 0.59 g in 83% yield as a white solid.

^{1}H NMR (500 MHz, $CDCl_3$) δ 7.49 (d, *J* = 8.1 Hz, 2H), 7.35-7.32 (m, 2H), 7.27 – 7.20 (m, 2H), 2.50-2.48 (m, 2H), 1.96-1.94 (m, 4H), 1.13-1.07 (m, 4H), 0.72 (t, *J* = 7.3 Hz, 3H), 0.57-0.53 (m, 2H). ^{13}C NMR (126 MHz, $CDCl_3$) δ 151.7 , 138.5 , 133.3 , 127.6 , 123.4 , 120.9 , 55.2 , 51.0 , 39.7 , 38.7 , 25.7 , 23.0 , 19.1 , 13.9. MS (m/z, ESI): Calcd. for Chemical Formula: $C_{20}H_{21}Cl_2O_3S^-$ [M-H]$^-$: 411.0594, Found: 411.0599.

4-(9-butyl-2,7-dichloro-9H-fluoren-9-yl)butane-1-sulfonic acid

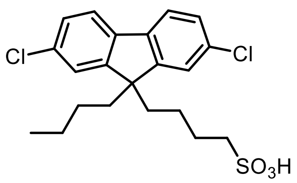

The compound was prepared using a similar procedure to 3-(9-butyl-2,7-dichloro-9H-fluoren-9-yl)propane-1-sulfonic acid. 0.63 g, 86% yield, white solid. ^1H NMR (500 MHz, $CDCl_3$) δ 7.53 (d, *J* = 8.0 Hz, 2H), 7.36 – 7.24 (m, 4H), 2.52-2.51 (m, 2H), 1.91-1.87 (m, 4H), 1.43-1.41 (m, 2H), 1.12-1.08 (m, 2H), 0.70 (t, *J* = 7.3 Hz, 3H), 0.59-0.56 (m, 4H). ^{13}C NMR (126 MHz, $CDCl_3$) δ 151.9, 138.6, 133.3, 127.5, 123.3, 120.8, 55.4, 40.1, 39.4, 25.8, 24.2, 23.0, 22.6, 13.9. MS (m/z, ESI): Calcd. for Chemical Formula: $C_{21}H_{23}Cl_2O_3S^-$ [M-H]$^-$: 425.0750, Found: 425.0754.

3. Substrate bearing triflimide was prepared following the synthetic route shown below.

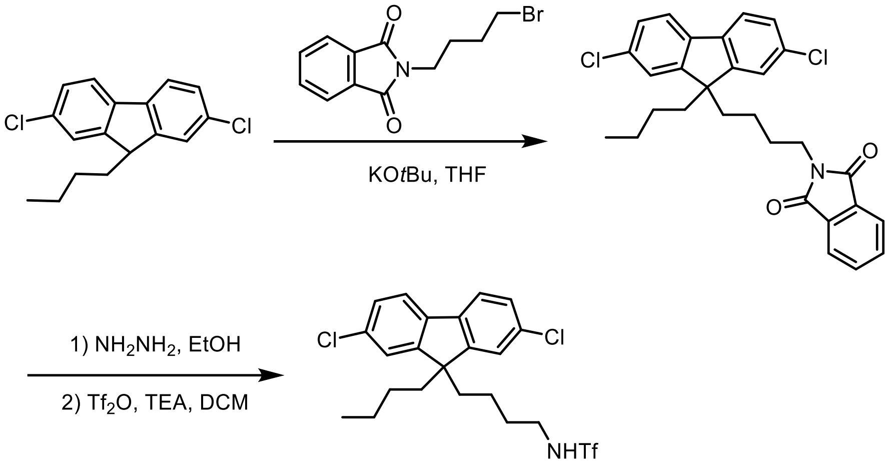

Under N_2 atmosphere, 9-butyl-2,7-dichloro-9H-fluorene (1.0 g, 3.43 mmol) was dissolved in anhydrous 6 mL THF and *t*BuOK (0.36 g, 3.8 mmol) was added dropwise at 0 °C. After addition was completed, the reaction mixture was stirring for 30 min at room temperature and 2-(4-bromobutyl)isoindoline-1,3-dione (1.07 g, 3.8 mmol) was added in one portion at 0 °C.[5] The resulting reaction mixture was warmed to room temperature and stirred overnight. The reaction was quenched with saturated NH_4Cl solution and extracted with ethyl acetate (15 mL X 3) three times. The combined organic phases were washed with brine, dried over Na_2SO_4, and concentrated in *vacuo*. The crude materials were purified by flash column chromatography to give the desired product 1.33 g in 79 % yield as a white solid.

The compound prepared from the previous step (1.33 g, 2.7 mmol) was dissolved in 8 mL EtOH and NH_2NH_2-H_2O (0.81 g, 16.2 mmol) was added. The resulting reaction mixture was heated at 70 °C for 8 h. Then solid was removed and the organic phase was purified by flash column chromatography to give the desired product 0.61 g in 62% yield.

At 0 °C, to a mixture of primary amine prepared in the previous step (0.2 g, 0.55 mmol) and triethylamine (0.17 g, 1.66 mmol) in anhydrous 1.5 mL DCM. Tf_2O (0.234 g, 0.83 mmol) was added dropwise to the reaction mixture. Water was added to quench the reaction. The reaction was extracted with DCM (5 mL X 3) for three times. The combined organic phases were washed with brine, dried over Na_2SO_4, and concentrated in *vacuo*. The crude materials were purified by flash column chromatography to give the desired product. The product was recrystallized in ethyl acetate and hexane to give the desired products 0.170 g in 62% yield as a white solid.

N-(4-(9-butyl-2,7-dichloro-9H-fluoren-9-yl)butyl)-1,1,1-trifluoromethanesulfonamide

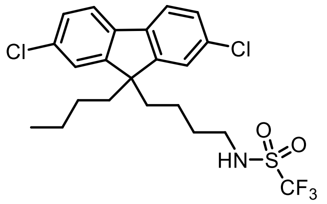

Prepared from 9-butyl-2,7-dichloro-9H-fluorene in four steps, 34% yield, white solid. ^{1}H NMR (300 MHz, $CDCl_3$) δ 7.63 (dd, *J* = 7.9, 2.6 Hz, 2H), 7.39-7.33 (m, 4H), 4.92 (s, 1H), 3.10-3.06 (m, 2H), 2.10-1.88 (m, 4H), 1.42-1.40 (m, 2H), 1.16-1.14 (m, 2H), 0.80-0.49 (m, 7H). ^{13}C NMR (75 MHz, $CDCl_3$) δ 151.6, 138.6, 133.4, 127.6, 123.1, 120.9, 119.6 (q, *J* = 321.3 Hz), 55.3, 44.0, 39.9, 39.5, 30.2, 25.7, 22.8, 20.4, 13.7. ^{19}F NMR (471 MHz, $CDCl_3$) -77.39 ppm. MS (m/z, ESI): Calcd. for Chemical Formula: $C_{22}H_{23}Cl_2F_3O_2S^-$ $[M-H]^-$: 492.0784, Found: 492.0784.

4. Preparation of substrate bearing amides.

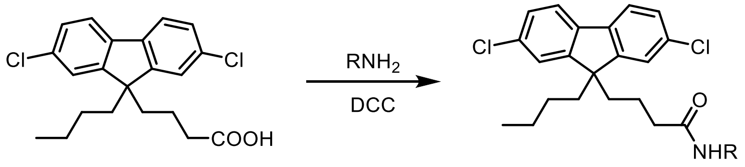

N-butyl-4-(9-butyl-2,7-dichloro-9H-fluoren-9-yl)butanamide

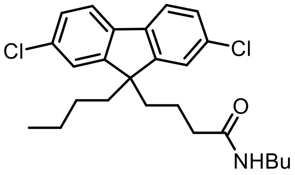

This compound was synthesized by the condensation of 4-(9-butyl-2,7-dichloro-9H-fluoren-9-yl)butanoic acid **51** with $BuNH_2$ using DCC according previous literature,[6] one step, 56% yield, white solid. ^1H NMR (500 MHz, $CDCl_3$) δ 7.59 (d, *J* = 8.1 Hz, 2H), 7.33 (dd, *J* = 8.0, 1.9 Hz, 2H), 7. 31 (d, *J* = 1.6 Hz, 2H), 5.18 (s, 1H), 3.19-3.15 (m, 2H), 2.03 – 1.98 (m, 2H), 1.96 – 1.92 (m, 4H), 1.46 – 1.40 (m, 2H), 1.34-1.29 (m, 2H), 1.13-1.08 (m, 2H), 0.99-0.95 (m, 2H), 0.93-0.88 (m, 3H), 0.70 (t, *J* = 7.4 Hz, 3H), 0.59-0.54 (m, 2H). ^{13}C NMR (126 MHz, $CDCl_3$) δ 172.2, 151.8, 138.6, 133.3, 127.6, 123.3, 120.9, 77.3, 77.0, 76.8, 55.3, 40.0, 39.6, 39.2, 36.7, 31.7, 25.7, 22.9, 20.3, 20.1, 13.8, 13.7. MS (m/z, ESI): Calcd. for Chemical Formula: $C_{25}H_{30}Cl_2NO^-$ $[M-H]^-$: 430.1710, Found: 430.1710.

4-(9-butyl-2,7-dichloro-9H-fluoren-9-yl)-N-phenylbutanamide

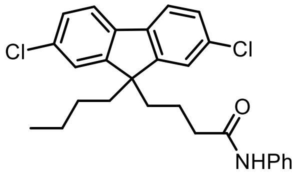

This compound was synthesized by the condensation of 4-(9-butyl-2,7-dichloro-9H-fluoren-9-yl)butanoic acid **51** with $PhNH_2$ using DCC according previous literature.[6] One step, 52% yield, white solid. ^1H NMR (500 MHz, $CDCl_3$) δ 7.60 (d, *J* = 8.1 Hz, 2H), 7.44 (d, *J* = 7.9 Hz, 2H), 7.36 – 7.29 (m, 6H), 7.10 (t, *J* = 7.4 Hz, 1H), 6.93 (s, 1H), 2.12 (t, *J* = 7.5 Hz, 2H), 2.09 – 2.04 (m, 2H), 1.98 – 1.94 (m, 2H), 1.13 – 1.04 (m, 4H), 0.71 (t, *J* = 7.4 Hz, 3H), 0.62 – 0.55 (m, 2H). ^{13}C NMR (126 MHz, $CDCl_3$) δ 151.7, 138.7, 133.4, 129.0, 127.7, 124.2, 123.3, 120.9, 119.7, 40.0, 39.5, 37.7, 25.7, 22.9, 20.1, 13.8. MS (m/z, ESI): Calcd. for Chemical Formula: $C_{27}H_{26}Cl_2NO^-$ $[M-H]^-$: 450.1397, Found: 450.1395.

5. Preparation of substrate bearing alcohol.

4-(9-butyl-2,7-dichloro-9H-fluoren-9-yl)butan-1-ol

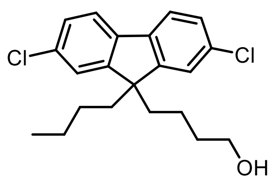

This compound was prepared by reduction of 4-(9-butyl-2,7-dichloro-9H-fluoren-9-yl)butanoic acid using lithium triethylborohydride (1.0 mol/L) in THF according previous literature.[7] 92% yield, white solid. ^1H NMR (500 MHz, $CDCl_3$) δ 7.59 (d, *J* = 8.0 Hz, 2H), 7.33 (dd, *J* = 8.0, 1.9 Hz, 2H), 7.31 (d, *J* = 1.8 Hz, 2H), 3.87 (t, *J* = 6.8 Hz, 2H), 2.01-1.98 (m, 2H), 1.97 – 1.93 (m, 2H), 1.48 – 1.37 (m, 2H), 1.14-1.10 (m, 2H), 0.71 (t, *J* = 10.0 Hz, 3H), 0.69-0.63 (m, 2H), 0.63 – 0.55 (m, 2H). ^{13}C

NMR (126 MHz, $CDCl_3$) δ 151.9, 138.6, 133.3, 127.5, 123.2, 120.8, 63.7, 55.5, 40.0, 39.6, 28.6, 25.8, 22.9, 20.9, 19.9, 13.8. MS (m/z, ESI): Calcd. for Chemical Formula: $C_{21}H_{23}Cl_2O^-$ $[M-H]^-$: 361.1, Found: 361.2.

6. Preparation of substrate bearing cyclohexyl group.

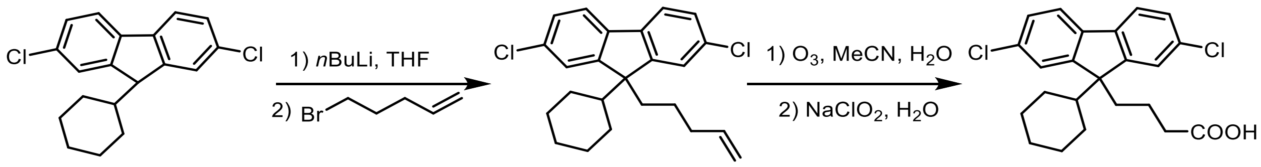

The procedure to prepare 2,7-dichloro-9-cyclohexyl-9H-fluorene is same as the synthesis of 9-butyl-2,7-dichloro-9H-fluorene. Under N_2 atmosphere, 2,7-dichloro-9-cyclohexyl-9H-fluorene (0.8 g, 2.5 mmol) was dissolved in anhydrous 8 mL THF and *n*BuLi (1.4 mL, 2.8 mmol, 2.0 mmol/L) was added dropwise at -78 °C. After addition was completed, the reaction mixture was stirring for 30 min at room temperature and 5-bromopent-1-ene (0.45 g, 3.0 mmol) was added in one portion at -78 °C and stirred for 30 min. The resulting reaction mixture was warmed to room temperature and stirred overnight. The reaction was quenched with saturated NH_4Cl solution and extracted with ethyl acetate (15 mL X 3) three times. The combined organic phases were washed with brine, dried over Na_2SO_4, and concentrated in *vacuo*. The crude materials were purified by flash column chromatography to give the desired product 0.5 g in 51% yield as a white solid. (The following steps are performed according to the synthetic route of xanthene substrates.)

4-(2,7-dichloro-9-cyclohexyl-9H-fluoren-9-yl)butanoic acid

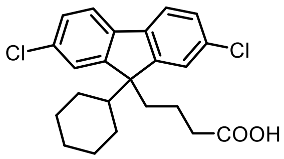

The procedure to this compound is similar to the synthesis of Xanthene-type substrates, starting from 2,7-dichloro-9H-fluorene. Three steps, 18% yield, white solids. ^{1}H NMR (500 MHz, $CDCl_3$) δ 7.57 (dd, *J* = 7.3, 1.4 Hz, 2H), 7.35-7.33 (m, 3H), 7.32 (d, *J* = 1.9 Hz, 1H), 2.14 (t, *J* = 7.3 Hz, 2H), 2.12 – 2.07 (m, 2H), 1.87-1.83 (m, 1H), 1.67 – 1.56 (m, 4H), 1.52-1.48 (m, 2H), 1.21 – 1.10 (m, 2H), 0.87-0.83 (m, 2H), 0.80-0.76 (m, 2H). ^{13}C NMR (126 MHz, $CDCl_3$) δ 177.9, 151.3, 139.0, 133.2, 127.5, 123.9, 120.6, 58.6, 47.6, 35.6, 33.8, 27.7, 26.8, 26.3, 19.1. MS (m/z, ESI): Calcd. for Chemical Formula:

$C_{23}H_{23}Cl_2O_2^-$ [M-H]$^-$: 401.1081, Found: 401.1082.

7. The general procedure for the synthesis of xanthene substrates is shown below.[8]

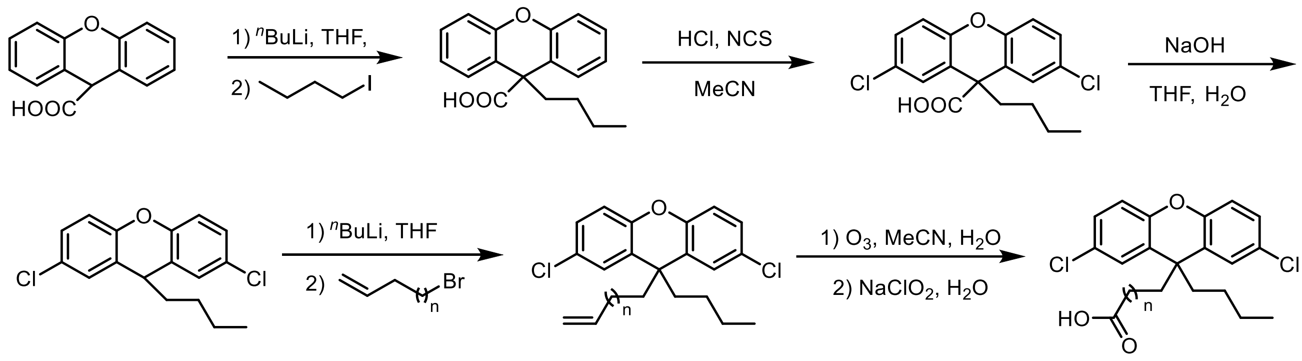

9-butyl-9H-xanthene-9-carboxylic acid

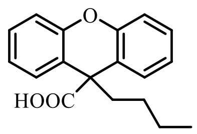

Under N_2 atmosphere, 9H-xanthene-9-carboxylic acid (4.52 g, 20 mmol) was dissolved in anhydrous THF (100 mL), then *n*BuLi (2 M, 21 mL, 42 mmol) as a cyclohexane solution was added dropwise at -78 ^{o}C. After addition was completed, the resulting reaction mixture was stirred for 30 min at room temperature. 1-iodobutane (4.42 g, 24 mmol) was added in one portion at -78 ^{o}C and stirring at this temperature for 20 min. Then the reaction mixture was warmed to room temperature and stirring overnight. The reaction was quenched with saturated NH_4Cl solution and extracted with ethyl acetate three times. The combined organic phases were washed with brine, dried over Na_2SO_4, and concentrated in *vacuo*. The crude mixture was purified by column chromatography on silica gel (hexane: acetone = 10: 1) to afford the target compound (4.12 g, 73%) as a white solid.

^{1}H NMR (500 MHz, $CDCl_3$) δ 7.30 – 7.26 (m, 2H), 7.25 – 7.23 (m, 2H), 7.11 – 7.06 (m, 4H), 2.29 – 2.23 (m, 2H), 1.12 – 1.19 (m, 2H), 0.82 – 0.76 (m, 2H), 0.70 (t, *J* = 7.3 Hz, 3H). ^{13}C NMR (126 MHz, $CDCl_3$) δ 177.6, 150.8, 129.0, 127.3, 123.5, 121.0, 116.9, 49.8, 40.2, 26.1, 22.8, 13.9.

9-butyl-2,7-dichloro-9H-xanthene-9-carboxylic acid

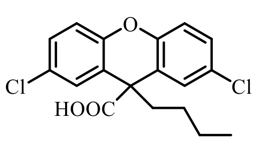

9-butyl-9H-xanthene-9-carboxylic acid (4.12 g, 14.6 mmol) was dissolved in MeCN (100 mL), then conc. HCl (10 mL, 8.3 mmol) was added. After that, NCS (4.31 g, 32.1 mmol) was added into the reaction mixture at 0 °C. Then the reaction mixture was warmed to room temperature and stirring for 12 h. The reaction was quenched with saturated $NaHCO_3$ solution and extracted with ethyl acetate three times. The combined organic phases were washed with brine, dried over Na_2SO_4, and concentrated in *vacuo*. The crude mixture was purified by column chromatography on silica gel (hexane: acetone = 10: 1) to afford the target compound (4.77 g, 93%) as a white solid.

^{1}H NMR (400 MHz, $CDCl_3$) δ 7.26 (d, *J* = 2.3 Hz, 2H), 7.24 (d, *J* = 1.7 Hz, 2H), 7.05 – 7.03 (m, 1H), 7.03 – 7.01 (m, 1H), 2.28 – 2.19 (m, 2H), 1.16 – 1.21 (m, 2H), 0.88 – 0.67 (m, 5H). ^{13}C NMR (126 MHz, $CDCl_3$) δ 177.4, 149.2, 129.5, 128.6, 127.1, 121.9, 118.4, 50.1, 39.9, 26.2, 22.7, 13.9.

9-butyl-2,7-dichloro-9H-xanthene

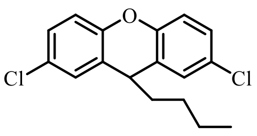

9-butyl-2,7-dichloro-9H-xanthene-9-carboxylic acid (1.97 g, 5.6 mmol) was dissolved in THF-H_2O co-solvents (8: 1, 45 mL), then NaOH (449 mg, 11.2 mmol) was added. After that, the reaction mixture was heated to 60 °C and stirring for overnight. The reaction was quenched with 1 N HCl solution and extracted with ethyl acetate three times. The combined organic phases were washed with brine, dried over Na_2SO_4, and concentrated in *vacuo*. The crude mixture was purified by column chromatography on silica gel (hexane) to afford the target compound (1.63 g, 95%) as a white solid.

^{1}H NMR (500 MHz, $CDCl_3$) δ 7.17 (d, *J* = 8.3 Hz, 4H), 7.02 – 6.97 (m, 2H), 3.92 (t, *J* = 5.9 Hz, 1H), 1.74 – 1.67 (m, 2H), 1.26 – 1.18 (m, 2H), 1.07 – 1.13 (m, 2H), 0.80 (t, *J* = 7.3 Hz, 3H). ^{13}C NMR (126 MHz, $CDCl_3$) δ 150.7, 128.3, 128.2, 127.8, 126.7, 117.8, 40.4, 39.0, 27.4, 22.7, 14.0.

9-butyl-2,7-dichloro-9-(pent-3-en-1-yl)-9H-xanthene

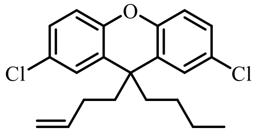

Under N_2 atmosphere, 9-butyl-2,7-dichloro-9H-xanthene (1.09 g, 3.6 mmol) was dissolved in anhydrous THF (18 mL) and *n*BuLi (2 M, 2.16 mL, 4.32 mmol) as a cyclohexane solution was added dropwise at -78 °C. After addition was completed, the reaction mixture was stirring for 30 min at room temperature and 4-bromopent-1-ene (730 mg, 5.4 mmol) was added dropwise at -

78 °C. The resulting reaction mixture was warmed to room temperature and stirred overnight. The reaction was quenched with saturated NH_4Cl solution and extracted with ethyl acetate three times. The combined organic phases were washed with brine, dried over Na_2SO_4, and concentrated in *vacuo*. The crude mixture was purified by column chromatography on silica gel (hexane) to afford the target compound (909 mg, 70%) as a white solid.

^{1}H NMR (400 MHz, $CDCl_3$) δ 7.23 (d, *J* = 2.5 Hz, 2H), 7.18 – 7.13 (m, 2H), 6.94 (d, *J* = 8.7 Hz, 2H), 5.67 – 5.54 (m, 1H), 4.88 – 4.77 (m, 2H), 2.00 – 1.94 (m, 2H), 1.91 – 1.86 (m, 2H), 1.65 – 1.58 (m, 2H), 1.13 – 1.18 (m, 2H), 0.87 – 0.78 (m, 2H), 0.73 (t, *J* = 7.3 Hz, 3H). ^{13}C NMR (126 MHz, $CDCl_3$) δ 150.0, 137.9, 128.3, 127.8, 126.3, 126.0, 117.6, 114.5, 45.0, 44.4, 42.7, 29.1, 26.9, 22.8, 13.8.

9-butyl-2,7-dichloro-9-(pent-4-en-1-yl)-9H-xanthene

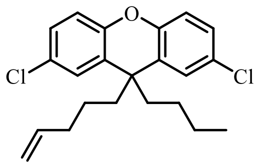

Under N_2 atmosphere, 9-butyl-2,7-dichloro-9H-xanthene (1.86 g, 6.06 mmol) was dissolved in anhydrous THF (30 mL) and *n*BuLi (2 M, 3.64 mL, 7.27 mmol) as a cyclohexane solution was added dropwise at -78 °C. After addition was completed, the reaction mixture was stirring for 30 min at room temperature and 5-bromopent-1-ene (1.35 g, 9.09 mmol) was added dropwise at -78 °C. The resulting reaction mixture was warmed to room temperature and stirred overnight. The reaction was quenched with saturated NH_4Cl solution and extracted with ethyl acetate three times. The combined organic phases were washed with brine, dried over Na_2SO_4, and concentrated in *vacuo*. The crude mixture was purified by column chromatography on silica gel (hexane) to afford the target compound (1.8 g, 79%).

^{1}H NMR (500 MHz, $CDCl_3$) δ 7.21 (d, *J* = 2.5 Hz, 2H), 7.17 – 7.13 (m, 2H), 6.93 (d, *J* = 8.7 Hz, 2H), 5.59 (ddt, *J* = 15.7, 11.5, 6.6 Hz, 1H), 4.91 – 4.85 (m, 2H), 1.91 – 1.83 (m, 6H), 1.12 – 1.17 (m, 2H), 0.99 – 0.91 (m, 2H), 0.85 – 0.78 (m, 2H), 0.73 (t, *J* = 7.4 Hz, 3H). ^{13}C NMR (126 MHz, $CDCl_3$) δ 150.1, 138.4, 128.4, 127.9, 126.7, 126.1, 117.7, 114.9, 45.3, 44.8, 42.9, 33.8, 27.0, 24.1, 23.0, 14.0.

9-butyl-2,7-dichloro-9-(pent-5-en-1-yl)-9H-xanthene

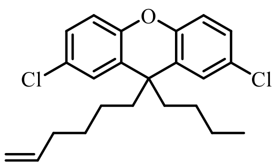

Under N_2 atmosphere, 9-butyl-2,7-dichloro-9H-xanthene (1.34 g, 4.4 mmol) was dissolved in anhydrous THF (22 mL) and *n*BuLi (2 M, 2.64 mL, 5.28 mmol) as a cyclohexane solution was added dropwise at -78 °C. After addition was completed, the reaction mixture was stirring for 30 min at room temperature and 6-bromopent-1-ene (1.08 g, 6.6 mmol) was added dropwise at -78 °C. The resulting reaction mixture was warmed to room temperature and stirred overnight. The reaction was quenched with saturated NH_4Cl solution and extracted with ethyl acetate three times. The combined organic phases were washed with brine, dried over Na_2SO_4, and concentrated in *vacuo*. The crude mixture was purified by column chromatography on silica gel (hexane) to afford the target compound (1.29 g, 75%) as a white solid.

^{1}H NMR (500 MHz, $CDCl_3$) δ 7.21 (d, *J* = 2.5 Hz, 2H), 7.16 (d, *J* = 2.5 Hz, 1H), 7.14 (d, *J* = 2.5 Hz, 1H), 6.94 (d, *J* = 8.7 Hz, 2H), 5.65 (ddt, *J* = 16.9, 10.2, 6.6 Hz, 1H), 4.92 – 4.82 (m, 2H), 1.91 – 1.83 (m, 6H), 1.26 – 1.19 (m, 2H), 1.13 – 1.17 (m, 2H), 0.89 – 0.79 (m, 4H), 0.73 (t, *J* = 7.4 Hz, 3H). ^{13}C NMR (126 MHz, $CDCl_3$) δ 150.1, 138.7, 128.3, 127.8, 126.8, 126.1, 117.7, 114.5, 45.4, 45.2, 43.0, 33.4, 29.0, 27.1, 24.3, 23.0, 14.0.

3-(9-butyl-2,7-dichloro-9H-xanthen-9-yl)propanoic acid

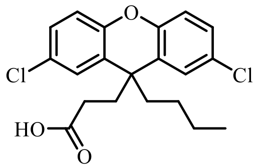

9-(but-3-en-1-yl)-9-butyl-2,7-dichloro-9H-xanthene (682 mg, 1.89 mmol) was dissolved in MeCN-H_2O co-solvents (9: 1, 20 mL).The solution was cooled to 0 °C and sparged with ozone, meanwhile, reaction completeness was confirmed by TLC. An aqueous solution of $NaClO_2$ (80 wt%, 684, 7.56 mmol) in H_2O (8 mL) was added portion-wise to the reaction while maintaining an internal reaction temp <15 °C. The cold bath was removed from the reaction and the mixture stirred overnight at room temperature. The reaction was quenched with a saturated $Na_2S_2O_3$ solution at 0 °C and the reaction mixture was allowed to stir for 4 h at room temperature, then it was extracted with ethyl acetate three times. The combined organic phases were washed with brine, dried over Na_2SO_4, and concentrated in *vacuo*. The crude mixture was purified by column chromatography on silica gel (hexane: acetone = 10: 1) to afford the target compound (444 mg, 62%) as a white solid.

^{1}H NMR (500 MHz, $CDCl_3$) δ7.23 (d, *J* = 2.5 Hz, 2H), 7.18 (d, *J* = 2.4 Hz, 1H), 7.16 (d, *J* = 2.4 Hz, 1H), 6.95 (d, *J* = 8.7 Hz, 2H), 2.27 – 2.20 (m, 2H), 1.95 – 1.88 (m, 4H), 1.13 – 1.18 (m, 2H), 0.84 – 0.78 (m, 2H), 0.73 (t, *J* = 7.3 Hz, 3H).

^{13}C NMR (126 MHz, $CDCl_3$) δ 177.7, 150.1, 128.7, 128.4, 126.0, 125.3, 118.0, 45.1, 42.3, 39.6, 29.9, 27.1, 22.9, 13.9. HRMS (m/z, ESI): Calcd. for Chemical Formula: $C_{20}H_{19}Cl_2O_3^-$[M-H]$^-$: 377.0717, Found: 377.0714.

4-(9-butyl-2,7-dichloro-9H-xanthen-9-yl)butanoic acid

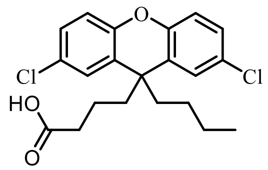

9-butyl-2,7-dichloro-9-(pent-4-en-1-yl)-9H-xanthene (1.8 g, 4.8 mmol) was dissolved in MeCN-H_2O co-solvents (9: 1, 50 mL).The solution was cooled to 0 °C and sparged with ozone, meanwhile, reaction completeness was confirmed by TLC. An aqueous solution of $NaClO_2$ (80 wt%, 1.74 g, 19.2 mmol, 4 eq.) in H_2O (20 mL) was added portion-wise to the reaction while maintaining an internal reaction temp <15 °C. The cold bath was removed from the reaction and the mixture stirred overnight at room temperature. The reaction was quenched with a saturated $Na_2S_2O_3$ solution at 0 °C and the reaction mixture was allowed to stir for 2 h at room temperature, then it was extracted with ethyl acetate three times. The combined organic phases were washed with brine, dried over Na_2SO_4, and concentrated in *vacuo*. The crude mixture was purified by column chromatography on silica gel (hexane: acetone = 10: 1) to afford the target compound (1.23 g, 65%) as a white solid.

^1H NMR (500 MHz, $CDCl_3$) δ 7.22 (d, *J* = 2.5 Hz, 2H), 7.16 (dd, *J* = 8.7, 2.5 Hz, 2H), 6.94 (d, *J* = 8.7 Hz, 2H), 2.20 (t, *J* = 7.3 Hz, 2H), 1.96 – 1.92 (m, 2H), 1.87 – 1.83 (m, 2H), 1.23 – 1.17 (m, 2H), 1.16 – 1.11 (m, 2H), 0.84 – 0.78 (m, 2H), 0.72 (t, *J* = 7.4 Hz, 3H). ^{13}C NMR (126 MHz, $CDCl_3$) δ 177.3, 150.1, 128.5, 128.1, 126.2, 126.1, 117.9, 45.5, 44.2, 42.8, 33.5, 27.0, 22.9, 20.2, 14.0. HRMS (m/z, ESI): Calcd. for Chemical Formula: $C_{21}H_{21}Cl_2O_3^-$[M-H]$^-$: 391.0873, Found: 391.0871.

5-(9-butyl-2,7-dichloro-9H-xanthen-9-yl)pentanoic acid

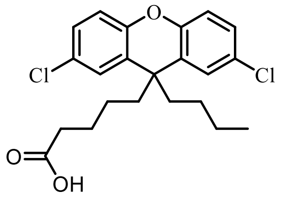

9-butyl-2,7-dichloro-9-(hex-5-en-1-yl)-9H-xanthene (1.29 g, 3.32 mmol) was dissolved in MeCN-H_2O co-solvents (9: 1, 35 mL).The solution was cooled to 0 °C and sparged with ozone, meanwhile, reaction completeness was confirmed by TLC. An aqueous solution of $NaClO_2$ (80 wt%, 1.2 g, 13.28 mmol) in H_2O (14 mL) was added portion-wise to the reaction while maintaining an internal reaction temp <15 °C. The cold bath was removed from the reaction and the mixture stirred overnight at room temperature. The reaction was quenched with a saturated

$Na_2S_2O_3$ solution at 0 °C and the reaction mixture was allowed to stir for 2 h at room temperature, then it was extracted with ethyl acetate three times. The combined organic phases were washed with brine, dried over Na_2SO_4, and concentrated in *vacuo*. The crude mixture was purified by column chromatography on silica gel (hexane: acetone = 10: 1) to afford the target compound (865 mg, 64%) as a white solid.

^1H NMR (500 MHz, $CDCl_3$) δ 7.20 (d, *J* = 2.5 Hz, 2H), 7.17 – 7.13 (m, 2H), 6.93 (dd, *J* = 8.7, 1.0 Hz, 2H), 2.17 (t, *J* = 7.7 Hz, 2H), 1.90 – 1.84 (m, 4H), 1.51 – 1.44 (m, 2H), 1.12 – 1.17 (m, 2H), 0.92 – 0.86 (m, 2H), 0.84 – 0.77 (m, 2H), 0.73 (t, *J* = 7.3 Hz, 3H). ^{13}C NMR (126 MHz, $CDCl_3$) δ 178.2, 150.1, 128.4, 128.0, 126.5, 126.0, 117.8, 45.3, 45.0, 42.9, 33.6, 27.0, 24.9, 24.4, 23.0, 14.0. HRMS (m/z, ESI): Calcd. for Chemical Formula: $C_{22}H_{23}Cl_2O_3^-$[M-H]$^-$: 405.1030, Found: 405.1028.

8. Substrates bearing extra distant chloroarenes were prepared following the scheme shown below.

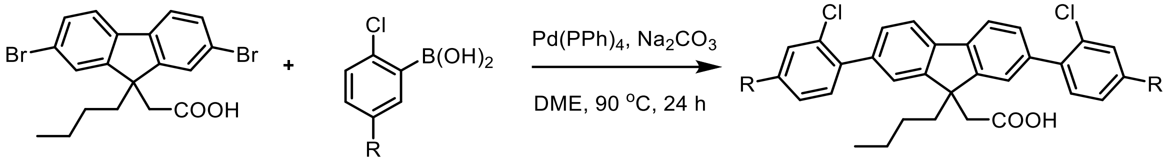

2-(2,7-dibromo-9-butyl-9H-fluoren-9-yl)acetic acid

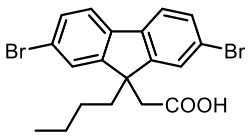

Prepared from 2,7-dibromofluorene in three steps, 52% yield, white solid. ^1H NMR (500 MHz, $CDCl_3$) δ 7.57 – 7.45 (m, 6H), 2.90 (s, 2H), 2.06 – 1.99 (m, 2H), 1.12 (dt, *J* = 14.7, 7.4 Hz, 2H), 0.71 (t, *J* = 7.4 Hz, 3H), 0.62-0.58 (m, 2H). ^{13}C NMR (126 MHz, $CDCl_3$) δ 174.5, 150.7, 138.8, 130.9, 126.5, 121.5, 121.4, 52.4, 43.1, 38.8, 25.6, 22.7, 13.8. MS (m/z, ESI): Calcd. for Chemical Formula: $C_{19}H_{17}Br_2O_2^-$ [M-H]$^-$: 434.9601, Found: 434.9598.

2-(9-butyl-2,7-bis(2-chlorophenyl)-9H-fluoren-9-yl)acetic acid

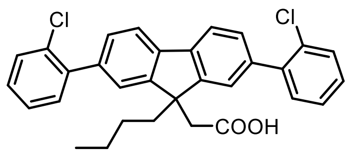

Under N_2 atmosphere, to a mixture of 2-(2,7-dibromo-9-butyl-9H-fluoren-9-yl)acetic acid (0.4 g, 0.9 mmol), (4-chlorophenyl)boronic acid (0.31 g, 2.0 mmol) and $Pd(PPh_3)_4$ were added 2.8 mL DME and followed by 2M Na_2CO_3.[9] The resulting reaction mixture was heated to 90 °C and stirred for 24 h. Water was added and the aqueous solution was acidified to pH 2-3 with 1M HCl solution and extracted with ethyl acetate (10 mL X 3) three times. The combined organic phases were washed with brine, dried over Na_2SO_4, and concentrated in *vacuo*. The crude materials were purified by flash column chromatography to give the desired product 0.42 g in 91% yield as a light white solid.

^{1}H NMR (500 MHz, $CDCl_3$) δ 7.80 (d, *J* = 7.8 Hz, 2H), 7.58 (d, *J* = 1.1 Hz, 2H), 7.51 (dd, *J* = 7.8, 1.3 Hz, 2H), 7.46-7.41 (m, 4H), 7.37 – 7.30 (m, 4H), 2.96 (s, 2H), 2.23 – 2.17 (m, 2H), 1.16-1.11 (m, 2H), 0.78 – 0.70 (m, 5H). ^{13}C NMR (126 MHz, $CDCl_3$) δ 174.3, 149.0, 140.7, 139.7, 138.4, 132.6, 131.5, 130.1, 128.9, 128.5, 126.9, 124.7, 119.7, 52.4, 43.4, 38.4, 25.9, 22.8, 13.8. MS (m/z, ESI): Calcd. for Chemical Formula: $C_{31}H_{25}Cl_2O_2^-$ [M-H]$^-$: 499.1237, Found: 499.1232.

2-(9-butyl-2,7-bis(2-chloro-4-methoxyphenyl)-9H-fluoren-9-yl)acetic acid

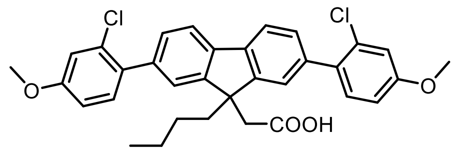

This compound was prepared using a similar method to 2-(9-butyl-2,7-bis(2-chlorophenyl)-9H-fluoren-9-yl)acetic acid. 91% yield, white solid. ^{1}H NMR (500 MHz, $CDCl_3$) δ 7.77 (d, *J* = 7.8 Hz, 2H), 7.54 (d, *J* = 0.9 Hz, 2H), 7.42 (dd, *J* = 7.8, 1.5 Hz, 2H), 7.34 (d, *J* = 8.5 Hz, 2H), 7.05 (d, *J* = 2.6 Hz, 2H), 6.90 (dd, *J* = 8.5, 2.6 Hz, 2H), 3.86 (s, 6H), 2.93 (s, 2H), 2.24 – 2.15 (m, 2H), 1.16-1.11 (m, 2H), 0.79 – 0.69 (m, 5H). ^{13}C NMR (126 MHz, $CDCl_3$) δ 159.3, 149.0, 139.4, 138.1, 133.2, 133.1, 132.0, 129.0, 124.8, 119.6, 115.2, 113.1, 55.6, 52.3, 43.6, 38.3, 25.9, 22.9, 13.8. MS (m/z, ESI): Calcd. for Chemical Formula: $C_{33}H_{29}Cl_2O_4^-$ [M-H]$^-$: 559.1448, Found: 559.1444.

9. Substrates bearing heteroatom adjacent to C9.

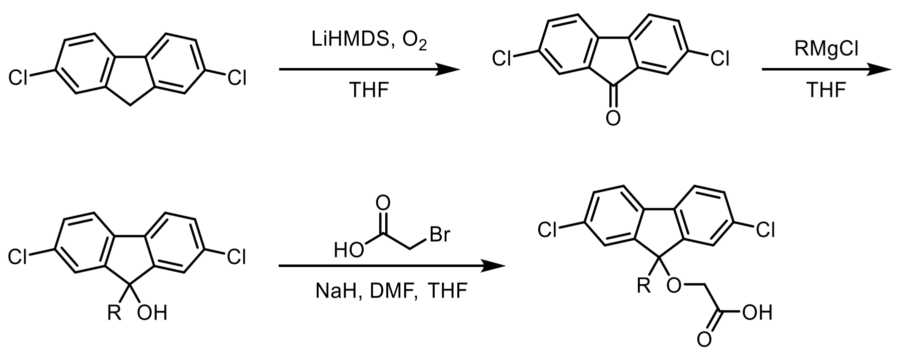

2,7-dichloro-9H-fluoren-9-one

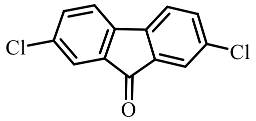

Under O_2 atmosphere, 2,7-dichloro-9H-fluorene (4.68 g, 20 mmol) was dissolved in anhydrous THF (80 mL), then LiHMDS (5.01 g, 30 mmol.) in anhydrous THF (20 mL) was added dropwise at 0 °C.[10] And, the resulting reaction mixture was stirred for 30 min at the same temperature. After that, the reaction mixture was allowed to warm to 60 °C and stirring at this temperature overnight. Then the reaction mixture was warmed to room temperature and quenched with saturated NH_4Cl solution and extracted with ethyl acetate three times. The combined organic phases were washed with brine, dried over Na_2SO_4, and concentrated in *vacuo*. The crude mixture was purified by column chromatography on silica gel (hexane: ethyl acetate = 20: 1) to afford the target compound (3.98 g, 80% yield) as a yellow solid.

^1H NMR (500 MHz, $CDCl_3$) δ 7.63 (d, *J* = 1.8 Hz, 2H), 7.47 (dd, *J* = 7.9, 1.9 Hz, 2H), 7.45 – 7.43 (m, 2H).

2,7-dichloro-9-methyl-9H-fluoren-9-ol

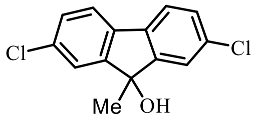

Under N_2 atmosphere, 2,7-dichloro-9H-fluoren-9-one (2.12 g, 8.5 mmol) was dissolved in anhydrous THF (50 mL), then MeMgCl (2 M, 12.75 mL, 25.5 mmol.) was added dropwise at 0 °C. And, the resulting reaction mixture was stirred for 30 min at the same temperature. After that, the reaction mixture was allowed to warm to room temperature and stirring at this temperature for 12 h. Then the reaction mixture was quenched with saturated NH_4Cl solution and extracted with ethyl acetate three times. The combined organic phases were washed with brine, dried over Na_2SO_4, and concentrated in *vacuo*. The crude mixture was purified by column chromatography on silica gel (hexane: ethyl acetate = 15: 1) to afford the target compound (1.85 g, 82% yield) as a yellow solid.

^1H NMR (500 MHz, $CDCl_3$) δ 7.53 – 7.48 (m, 4H), 7.34 (dd, *J* = 8.1, 2.0 Hz, 2H), 1.70 (s, 3H). ^{13}C NMR (126 MHz,

$CDCl_3$) δ 151.5, 136.4, 134.2, 129.5, 124.2, 121.3, 26.3.

9-butyl-2,7-dichloro-9H-fluoren-9-ol

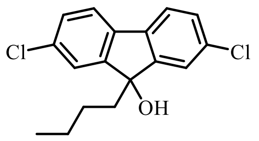

Under N_2 atmosphere, 2,7-dichloro-9H-fluoren-9-one (2.12 g, 8.5 mmol) was dissolved in anhydrous THF (40 mL), then *n*-BuMgCl (2 M, 12.75 mL, 25.5 mmol.) was added dropwise at 0 °C. And, the resulting reaction mixture was stirred for 30 min at the same temperature. After that, the reaction mixture was allowed to warm to room temperature and stirring at this temperature for 12 h. Then the reaction mixture was quenched with saturated NH_4Cl solution and extracted with ethyl acetate three times. The combined organic phases were washed with brine, dried over Na_2SO_4, and concentrated in *vacuo*. The crude mixture was purified by column chromatography on silica gel (hexane: ethyl acetate = 15: 1) to afford the target compound (1.85 g, 71%) as a yellow solid.

^1H NMR (500 MHz, $CDCl_3$) δ 7.51 – 7.45 (m, 4H), 7.35 – 7.31 (m, 2H), 2.13 – 2.03 (m, 3H), 1.24 – 1.15 (m, 2H), 0.89 – 0.81 (m, 2H), 0.77 (t, *J* = 7.4 Hz, 3H). ^{13}C NMR (126 MHz, $CDCl_3$) δ 150.6, 137.2, 134.2, 129.4, 124.3, 121.2, 82.5, 39.4, 26.0, 22.9, 13.9.

2-((2,7-dichloro-9-methyl-9H-fluoren-9-yl)oxy)acetic acid

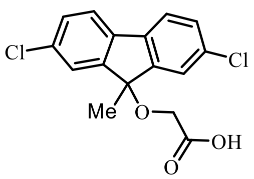

Under N_2 atmosphere, NaH (60 % dispersion in mineral oil, 360 mg, 9 mmol) was suspended in anhydrous THF (10 mL) and the mixture was allowed to cool to 0 °C. Next, methyl bromoacetate (626 mg, 4.5 mmol) in anhydrous THF (2 mL) was added dropwise into the reaction mixture at 0 °C, and the solution was allowed to warm to room temperature and stir for 1 h. 2,7-dichloro-9-methyl-9H-fluoren-9-ol (921mg, 3 mmol) anhydrous DMF (3 mL) was added dropwise at 0 °C.[11] And, the resulting reaction mixture was stirred for 30 min at the same temperature. After that, the reaction mixture was allowed to warm to room temperature and stirring at this temperature overnight. Then the reaction mixture was quenched with saturated NH_4Cl solution and extracted with ethyl acetate three times. The combined organic phases were washed with brine, dried over Na_2SO_4, and concentrated in *vacuo*. The crude mixture was purified by column chromatography on silica gel (hexane: acetone = 5: 1) to afford the target compound (700 mg, 62%) as a yellow solid.

^1H NMR (500 MHz, $CDCl_3$) δ 7.54 (dd, *J* = 8.1, 0.6 Hz, 2H), 7.46 (dd, *J* = 2.0, 0.5 Hz, 2H), 7.39 (dd, *J* = 8.1, 1.9 Hz,

2H), 3.47 (s, 2H), 1.76 (s, 3H). ^{13}C NMR (126 MHz, $CDCl_3$) δ 173.2, 146.9, 137.5, 134.6, 130.2, 124.6, 121.5, 85.2, 61.8, 26.1. HRMS (m/z, ESI): Calcd. for Chemical Formula: $C_{16}H_{11}Cl_2O_3^-$ [M-H]$^-$: 321.0091, Found: 321.0088.

2-((9-butyl-2,7-dichloro-9H-fluoren-9-yl)oxy)acetic acid

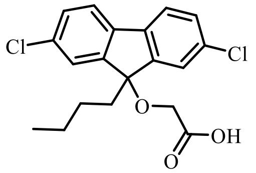

Under N_2 atmosphere, NaH (60 % dispersion in mineral oil, 72 mg, 3 equiv) was suspended in anhydrous THF (5 mL) and the mixture was allowed to cool to 0 °C. Next, methyl bromoacetate (209 mg, 1.5 mmol) in anhydrous THF (2 mL) was added dropwise into the reaction mixture at 0 °C, and the solution was allowed to warm to room temperature and stir for 1 h. 9-butyl-2,7-dichloro-9H-fluoren-9-ol (307 mg, 1 mmol) anhydrous DMF (1.5 mL) was added dropwise at 0 °C. And, the resulting reaction mixture was stirred for 30 min at the same temperature. After that, the reaction mixture was allowed to warm to room temperature and stirring at this temperature overnight. Then the reaction mixture was quenched with saturated NH_4Cl solution and extracted with ethyl acetate three times. The combined organic phases were washed with brine, dried over Na_2SO_4, and concentrated in *vacuo*. The crude mixture was purified by column chromatography on silica gel (hexane: acetone = 5: 1) to afford the target compound (281 mg, 77%) as a yellow solid.

^1H NMR (500 MHz, $CDCl_3$) δ 7.57 – 7.51 (m, 2H), 7.46 – 7.35 (m, 4H), 3.48 (s, 2H), 2.24 – 2.12 (m, 2H), 1.19 – 1.16 (m, 2H), 0.86 – 0.68 (m, 5H). ^{13}C NMR (126 MHz, $CDCl_3$) δ 172.6, 145.9, 138.3, 134.6, 130.2, 124.7, 121.4, 88.6, 61.6, 39.1, 25.5, 22.8, 13.9. HRMS (m/z, ESI): Calcd. for Chemical Formula: $C_{19}H_{17}Cl_2O_3^-$ [M-H]$^-$: 363.0560, Found: 363.0563.

10. Substrate for synthetic applications.

4-(9-((1,3-dioxolan-2-yl)methyl)-2,7-dichloro-9H-fluoren-9-yl)butanoic acid (57)

The substrate was prepared following the general procedure for synthesis of fluorene substrates described in Part 1.

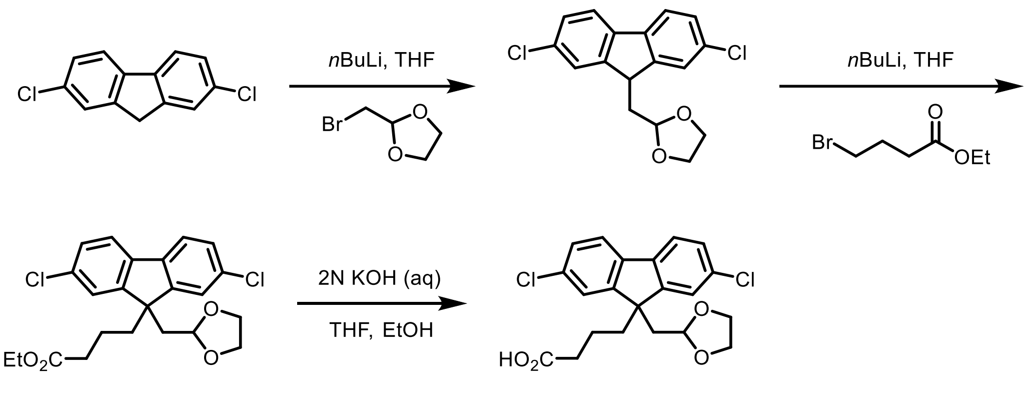

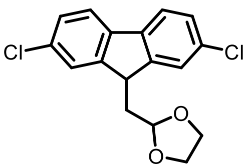

Under N_2 atmosphere, 2,7-dichloro-9H-fluorene (1.7 g, 5 mmol) was dissolved in anhydrous THF (20 mL). A solution of *n*BuLi in cyclohexane (2 M, 3.0 mL, 6.0 mmol) was added dropwise at -78 ^oC. Then, the reaction mixture was stirring for 30 min at room temperature, and cooled back to -78 ^oC. 2-(bromomethyl)-1,3-dioxolane (1.09 g, 6.5 mmol) was added dropwise at -78 ^oC. The resulting reaction mixture was warmed to room temperature and stirred overnight. The reaction was quenched with saturated NH_4Cl solution and extracted with ethyl acetate three times. The combined organic phases were washed with brine, dried over Na_2SO_4, and concentrated in *vacuo*. The crude product was purified by column chromatography on silica gel (hexane) to afford a light-yellow solid (788 mg, 49%).

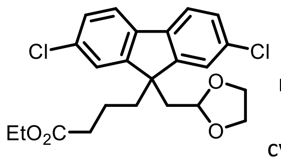

Under N_2 atmosphere, 2-((2,7-dichloro-9H-fluoren-9-yl)methyl)-1,3-dioxolane (788 mg, 2.46 mmol) was dissolved in anhydrous THF (10 mL). A solution of *n*BuLi in cyclohexane (2 M, 1.48 mL, 2.95 mmol) was added dropwise at -78 ^oC. Then, the reaction mixture was stirring for 30 min at room temperature, and cooled back to -78 ^oC. Ethyl 4-bromobutyrate (600 mg, 3.08 mmol) was added dropwise at -78 ^oC. The resulting reaction mixture was warmed to room temperature and stirred overnight. The reaction was quenched with saturated NH_4Cl solution and extracted with ethyl acetate three times. The combined organic phases were washed with brine, dried over Na_2SO_4, and concentrated in *vacuo*. The crude product was purified by column chromatography on silica gel (hexane : acetone = 100:1) to afford a light-yellow solid (448 mg, 46%).

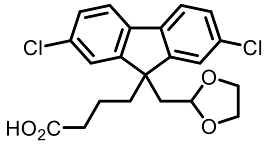

Ethyl 4-(9-((1,3-dioxolan-2-yl)methyl)-2,7-dichloro-9H-fluoren-9-yl)butanoate (1.0 g, 2.3 mmol) was dissolved in 3.0 mL THF and 3.0 mL EtOH, and 2 M KOH (11.5 mmol, 23 mL) was added. The reaction mixtures were stirred at reflux overnight and solvents were removed under reduced pressure. Water was added and the aqueous solution was acidified to pH 2-3 with 1 M HCl solution. The reaction was extracted with ethyl acetate (10 mL X 3) three times. The combined organic phases were washed with brine, dried over Na_2SO_4, and concentrated in *vacuo*. The crude materials were purified by flash column chromatography (hexane : acetone = 5:1) to give the desired product (0.85 g, 91%) yield as a white solid. ^1H NMR (500 MHz, $CDCl_3$) δ 7.57 (d, *J* = 8.1 Hz, 2H), 7.35 (d, *J* = 1.9 Hz, 2H), 7.32 (dd, *J* = 8.1, 1.8 Hz, 2H), 4.27 (t, *J* = 4.8 Hz, 1H), 3.76 – 3.70 (m, 2H), 3.59 – 3.50 (m, 2H), 2.29 (d, J = 4.8 Hz, 2H), 2.11 (t, J = 7.4 Hz, 2H), 2.07 – 2.00 (m, 2H), 0.92 – 0.85 (m, 2H). ^{13}C NMR (101 MHz, $CDCl_3$) δ 177.0, 150.7, 138.4, 133.4, 128.0, 124.1, 121.1, 102.0, 64.6, 52.8, 43.7, 33.5, 18.8. MS (m/z, ESI): Calcd. for Chemical Formula: $C_{21}H_{19}Cl_2O_4^-$ $[M-H]^-$: 405.0666, Found: 405.0667.

Optimization of reaction conditions (Tables S1–S5)

Table S1. Pd sources [a]

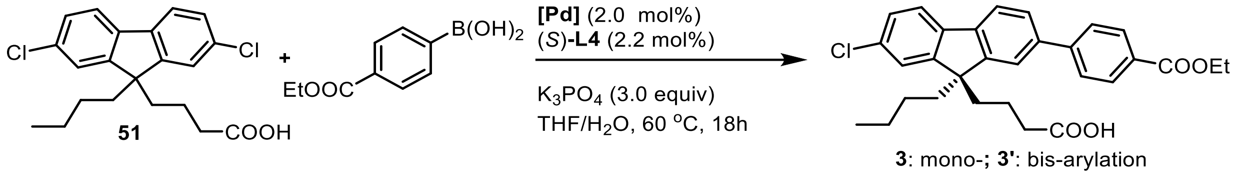

entry	Pd source	**51** (%)[b]	**3** (%)[b]	**3** (er)[c]	**3'** (%)[b]
1	$[Pd(allyl)Cl]_2$	9	76	93:7	15
2	$Pd(CH_2TMS)_2(COD)$	8	75	94:6	17
3	$Pd(OAc)_2$	5	79	77:23	16
4	$Pd_2(dba)_3$	4	79	94:6	17
5	$[Pd(2\text{-}Butenyl)Cl]_2$	6	78	83:17	16

[a] Unless otherwise stated, the reactions were performed with substrate (0.1 mmol), aryl boronic acid (0.1 mmol), [Pd] (2.0 mol%), (*S*)-**L4** (2.2 mol%), K_3PO_4 (0.3 mmol) in 0.95 mL THF and 0.05 mL H_2O at 60 °C for 18 h. [b] The ratio of starting material, mono and bis were determined by their corresponding ^1H NMR of crude reaction mixture. [c] Determined using chiral chromatography.

Table S2. Solvents [a]

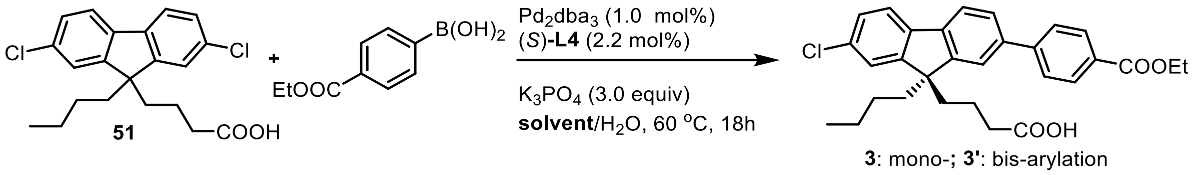

entry	solvent	**51** (%)[b]	**3** (%)[b]	**3** (er)[c]	**3’** (%)[b]
1	THF	4	79	94:6	17
2	Ethyl acetate	13	70	94:6	17
3	Dimethylformamide	33	52	66:34	17
4	Dimethoxyethane	13	68	93:7	19
5	Toluene	18	64	91:9	18
6	2-MeTHF	14	73	94:6	13

[a] Unless otherwise stated, the reactions were performed with **51** (0.1 mmol), aryl boronic acid (0.1 mmol), $Pd_2(dba)_3$ (1.0 mol%), (*S*)-**L4** (2.2 mol%), K_3PO_4 (0.3 mmol) in 0.95 mL THF and 0.05 mL H_2O at 60 °C for 18 h. [b] The ratio of starting material, mono and bis were determined by their corresponding ^1H NMR of crude reaction mixture. [c] Determined using chiral chromatography.

Table S3. Metal to ligand ratio, catalyst loading, temperature, quantity of base and boronic acid [a]

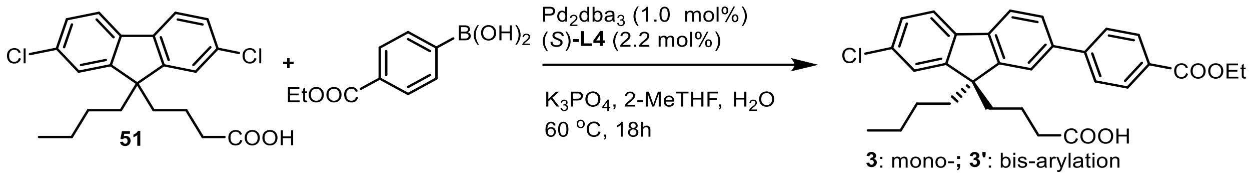

entry	condition changes	**51** (%)[b]	**3** (%)[b]	**3** (er)[c]	**3’** (%)[b]
1	--	14	73	94:6	13
2	45 °C	>90%	<10%	n.d.	n.d.
3	75 °C	12	73	93:7	15
4	1.0 mol% Pd_2bda_3, 4.4 mol% (*S*)-**L4**	10	73	93.5:6.5	17
5	2.0 mol% Pd_2bda_3, 4.4 mol% (*S*)-**L4**	9	73	93.5:6.5	18
6	10 equiv K_3PO_4, 0.165 mL H_2O	8	74	93.5:6.5	18
7	1.1 equiv aryl boronic acid, 10 eq K_3PO_4, 0.165 mL H_2O	7	74	95:5	19
8	1.2 equiv aryl boronic acid, 10 eq K_3PO_4, 0.165 mL H_2O	6	74	96:4	20

[a] Unless otherwise stated, the reactions were performed with **51** (0.1 mmol), aryl boronic acid (0.1 mmol), $Pd_2(dba)_3$ (1.0 mol%), (*S*)-**L4** (2.2 mol%), K_3PO_4 (0.3 mmol) in 0.95 mL THF and 0.05 mL H_2O at 60 °C for 18 h. [b] The ratio of starting material, mono and bis were determined by their corresponding ^1H NMR of crude reaction mixture. [c] Determined using chiral chromatography.

Table S4. Concentration [a]

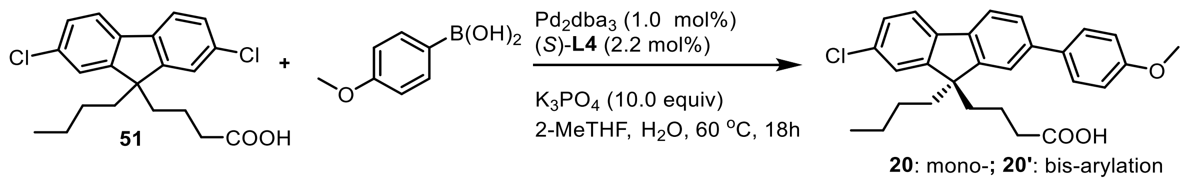

entry	Condition changes	**51** (%)[b]	**20** (%)[b]	**20** (er)[c]	**20'** (%)[b]
1	--	10	72	94:6	18
2	0.5 mL 2-MeTHF	7	75	96:4	18
3	2.0 mL 2-MeTHF	6	80	96:4	14
4	6.0 mL 2-MeTHF	8	80	97:3	12
5	6.0 mL 2-MeTHF, 1.0 mL H_2O	5	69	95:5	26

[a] Unless otherwise stated, the reactions were performed with **51** (0.1 mmol), arylboronic acid (0.12 mmol), $Pd_2(dba)_3$ (1.0 mol%), (*S*)-**L4** (2.2 mol%), K_3PO_4 (1.0 mmol) in 0.95 mL 2-MeTHF and 0.165 mL H_2O at 60 °C for 18 h. [b] The ratio of starting material, mono and bis were determined by their corresponding ^{1}H NMR of crude reaction mixture. [c] Determined using chiral chromatography.

Table S5. Bases [a]

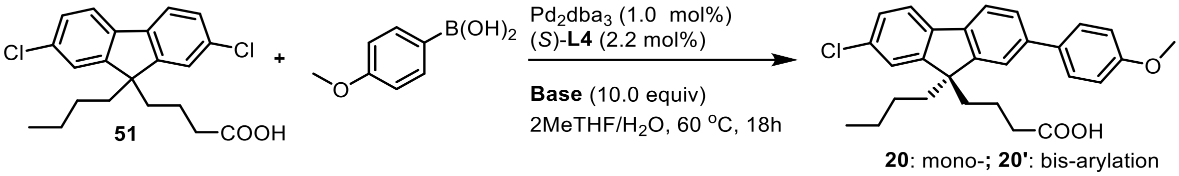

entry	base	**51** (%)[b]	**20** (%)[b]	**20** (er)[c]	**20'** (%)[b]
1	K_3PO_4	6	80	96:4	14
2	Li_2CO_3	72	26	75:25	2
3	Na_2CO_3	13	71	96:4	16
4	K_2CO_3	10	73	96:4	18
5	Cs_2CO_3	9	70	96:4	21
6	LiOH	61	36	86:13	3
7	NaOH	7	74	97:3	19
8	KOH	7	73	97:3	20
9	CsOH	6	70	97:3	24
10	TBAOH	26	54	91:9	20

[a] Unless otherwise stated, the reactions were performed with **51** (0.1 mmol), arylboronic acid (0.12 mmol), $Pd_2(dba)_3$ (1.0 mol%), (*S*)-**L4** (2.2 mol%), K_3PO_4 (1.0 mmol) in 2.0 mL 2-MeTHF and 0.165 mL H_2O at 60 °C for 18 h. [b] The ratio of starting material, mono and bis were determined by their corresponding ^1H NMR of crude reaction mixture. [c] Determined using chiral chromatography.

General procedure of catalytic desymmetrization reaction

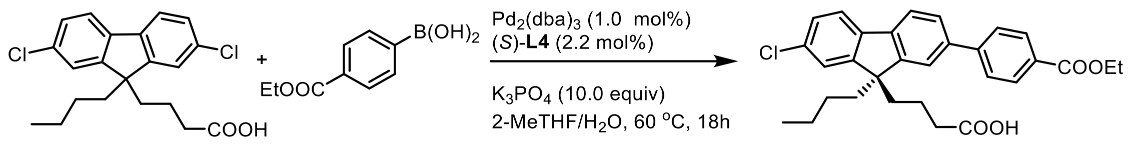

Under N_2 atmosphere, to a mixture of Pd_2dba_3 (2.3 mg, 0.0025 mmol) and (*S*)-**L4** (3.2 mg, 0.0055 mmol) was added 0.5 mL 2-MeTHF, and the mixture was stirred at room temperature for 20 min. The resulting metal-ligand complex solution was added to a reaction flask containing 4.5 mL 2-MeTHF solution of 4-(9-butyl-2,7-dichloro-9H-fluoren-9-yl)butanoic acid (94.4 mg, 0.25 mmol) and (4-(ethoxycarbonyl)phenyl)boronic acid (58.2 mg, 0.3 mmol), and K_3PO_4 (530.7 mg, 2.5 mmol), followed by addition of 0.41 mL H_2O. Then the resulting reaction mixture was stirred at 60 °C for 18h. The reaction was then quenched with water, neutralized to pH 3-5 with 1 M HCl and extracted with ethyl acetate (4 mL) for three times. The combined organic phases were washed with brine, dried over Na_2SO_4 and concentrated in *vacuo*. The crude materials were purified by flash column chromatography with hexane and ethyl acetate as the eluents to give the desired products.

Characterization data of desymmetrization products

(*R*)-ethyl 4-(9-butyl-7-chloro-9-(4-((trifluoromethyl)sulfonamido)butyl)-9H-fluoren-2-yl)benzoate (1)

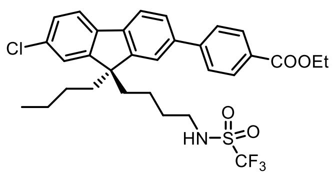

The reaction was carried out under conditions in Table 1.

White solid, 26.8 mg, 44% yield, $[\alpha]_D^{20}$ = +1.67 (*c* 0.100 $CHCl_3$) for 73:27 er. ^{1}H NMR (500 MHz, $CDCl_3$) δ 8.23 – 8.07 (m, 2H), 7.75 (ddd, *J* = 9.0, 8.5, 4.8 Hz, 3H), 7.69 – 7.63 (m, 2H), 7.58 (d, *J* = 1.2 Hz, 1H), 7.37 (dd, *J* = 8.0, 1.9 Hz, 1H), 7.35 (d, *J* = 1.7 Hz, 1H), 4.65 (s, 1H), 4.44 (q, *J* = 7.1 Hz, 2H), 3.09-3.06 (m, 2H), 2.14 – 1.97 (m, 4H), 1.47 – 1.37 (m, 5H), 1.14-1.10 (m, 2H), 0.76 – 0.59 (m, 7H). ^{13}C NMR (126 MHz, $CDCl_3$) δ 166.6, 152.2, 150.6, 145.5, 140.2, 139.5, 139.1, 133.4, 130.1, 129.3, 127.6, 127.0, 126.7, 123.2, 121.4, 121.1, 120.3, 119.6 (q, *J* = 321.4 Hz), 61.1, 55.3, 44.0, 40.1, 39.7, 30.5, 25.8, 22.9, 20.6, 14.4, 13.8. ^{19}F NMR (471 MHz, $CDCl_3$) δ -77.40. HRMS (m/z, ESI): Calcd. for Chemical Formula: $C_{31}H_{32}ClF_3NO_4S^-$ $[M-H]^-$: 606.1698, Found: 606.1700. HPLC analysis of the reaction product: Daicel Chiralpak IB, hexane/*iso*-propanol = 99: 1, 1.0 mL/min, λ = 321 nm, retention time: 24.47 min (major) and 27.09 min (minor).

(*R*)-ethyl 4-(9-butyl-7-chloro-9-(4-hydroxybutyl)-9H-fluoren-2-yl)benzoate (4)

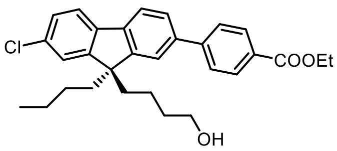

The reaction was carried out under conditions in Table 1.

White solid, 51.0 mg, 43% yield, $[\alpha]_D^{20}$ = +1.10 (*c* 0.100 $CHCl_3$) for 56:44 er. ^{1}H NMR (500 MHz, $CDCl_3$) δ 8.16 (d, *J* = 8.5 Hz, 2H), 7.77-7.73 (m, 3H), 7.69 – 7.62 (m, 2H), 7.58 (d, *J* = 0.9 Hz, 1H), 7.40 – 7.32 (m, 2H), 4.44 (q, *J* = 7.1 Hz, 2H), 3.88 (t, *J* = 6.8 Hz, 2H), 2.11 – 1.99 (m, 4H), 1.92 (s, 1H), 1.47-1.42 (m, 5H), 1.15-1.11 (m, 2H), 0.76 – 0.62 (m, 7H). ^{13}C NMR (126 MHz, $CDCl_3$) δ 171.1, 166.5, 152.5, 150.9, 145.7, 140.2, 139.3, 139.1, 133.3, 130.1, 129.2, 127.4, 127.0, 126.6, 123.3, 121.5, 121.0, 120.3, 63.8, 61.0, 55.4, 40.1, 39.7, 28.6, 25.9, 23.0, 20.9, 20.0, 14.4, 13.8. MS (m/z, ESI): Calcd. for Chemical Formula: $C_{30}H_{33}ClO_3Na^+$ $[M+Na]^+$: 499.2, Found: 499.3. HPLC analysis of the reaction product: Daicel Chiralpak IA, hexane/*iso*-propanol = 99: 1, 1.0 mL/min, λ= 324 nm, retention time: 7.04 min (major) and 7.74 min (minor).

(*R*)-ethyl 4-(9-butyl-9-(4-(butylamino)-4-oxobutyl)-7-chloro-9H-fluoren-2-yl)benzoate (5)

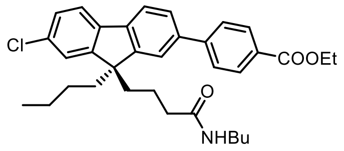

The reaction was carried out under conditions in Table 1.

White solid, 54.3 mg, 40% yield, $[\alpha]_D^{20}$ = -1.80 (*c* 0.040 $CHCl_3$) for 63:37 er. ^{1}H NMR (500 MHz, $CDCl_3$) δ 8.18 – 8.13 (m, 2H), 7.78 – 7.71 (m, 3H), 7.68 – 7.62 (m, 2H), 7.58 (d, *J* = 1.3 Hz, 1H), 7.36 (dd, *J* = 7.6, 1.5 Hz, 2H), 5.15 (s, 1H), 4.44 (q, *J* = 7.1 Hz, 2H), 3.18 – 3.11 (m, 2H), 2.11 – 1.98 (m, 4H), 1.94 (t, *J* = 7.5 Hz, 2H), 1.45 (t, *J* = 7.1 Hz, 3H), 1.41 – 1.36 (m, 2H), 1.14-1.10 (m, 2H), 1.04-1.01 (m, 2H), 0.93 – 0.86 (m, 3H), 0.71 (t, *J* = 7.3 Hz, 3H), 0.67 – 0.61 (m, 2H). ^{13}C NMR (126 MHz, $CDCl_3$) δ 172.3, 166.6, 152.4, 150.7, 140.2, 139.4, 139.1, 130.1, 129.2, 127.5, 127.1, 126.6, 123.3, 121.6, 121.1, 120.3, 76.9, 61.0, 55.3, 40.1, 39.7, 39.1, 36.8, 31.7, 25.9, 22.9, 20.4, 20.0, 14.4, 13.8, 13.7. HRMS (m/z, ESI): Calcd. for Chemical Formula: $C_{34}H_{39}ClNO_3^-$ $[M-H]^-$: 544.2633, Found: 544.2624. HPLC analysis of the reaction product: Daicel Chiralpak IA, hexane/*iso*-propanol = 96: 4, 1.0 mL/min, λ= 330 nm, retention time: 19.73 min (minor) and 20.89 min (major).

(*R*)-ethyl 4-(9-butyl-7-chloro-9-(4-oxo-4-(phenylamino)butyl)-9H-fluoren-2-yl)benzoate (6)

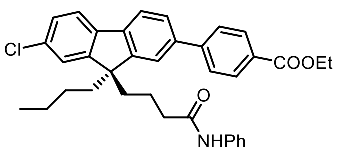

The reaction was carried out under conditions in Table 1.

White solid, 53.3 mg, 38% yield, $[\alpha]_D^{20}$ = -4.00 (*c* 0.100 $CHCl_3$) for 60:40 er. ^{1}H NMR (500 MHz, $CDCl_3$) δ 8.18 – 8.09 (m, 2H), 7.77 (d, *J* = 7.9 Hz, 1H), 7.75 – 7.70 (m, 2H), 7.69 – 7.62 (m, 2H), 7.60 (s, 1H), 7.40 (d, *J* = 7.9 Hz, 2H), 7.36 (dd, *J* = 6.1, 2.3 Hz, 2H), 7.26 (d, *J* = 8.3 Hz, 2H), 7.08 (t, *J* = 7.4 Hz, 1H), 6.92 (s, 1H), 4.44 (q, *J* = 7.1 Hz, 2H), 2.14-2.11 (m, 4H), 2.06 – 1.96 (m, 2H), 1.46 (t, *J* = 7.1 Hz, 3H), 1.13-1.00 (m, 4H), 0.73 – 0.69 (m, 3H), 0.67-0.64 (m, 2H). ^{13}C NMR (126 MHz, $CDCl_3$) δ 170.6, 166.6, 152.3, 150.6, 145.6, 140.2, 139.4, 139.2, 137.7, 133.4, 130.1, 129.2, 129.0, 127.6, 127.1, 126.7, 124.2, 123.3, 121.6, 121.1, 120.3, 119.6, 61.0, 55.3, 40.1, 39.5, 37.7, 25.8, 22.9, 20.2, 14.4, 13.8. HRMS (m/z, ESI): Calcd. for Chemical Formula: $C_{36}H_{35}ClNO_3^-$ $[M-H]^-$: 564.2311, Found: 564.2308. HPLC analysis of the reaction product: Daicel Chiralpak IA, hexane/*iso*-propanol = 96: 4, 1.0 mL/min, λ= 329 nm, retention time: 22.32 min (minor) and 35.64 min (major).

(*R*)-2-(9-butyl-2-chloro-7-(4-(ethoxycarbonyl)phenyl)-9H-fluoren-9-yl)acetic acid (7)

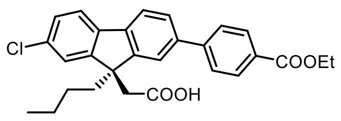

White solid, 48.3 mg, 42% yield, $[\alpha]_D^{20}$ = -0.30 (*c* 0.100 $CHCl_3$) for 82.5:17.5 er. ^1H NMR (500 MHz, $CDCl_3$) δ 8.16 – 8.11 (m, 2H), 7.75 (dd, *J* = 7.9, 0.4 Hz, 1H), 7.72 – 7.68 (m, 2H), 7.68 – 7.62 (m, 3H), 7.44 (d, *J* = 1.8 Hz, 1H), 7.37 (dd, *J* = 8.1, 1.9 Hz, 1H), 4.44 (q, *J* = 7.1 Hz, 2H), 3.02 – 2.92 (m, 2H), 2.15-2.11 (m, 2H), 1.45 (t, *J* = 7.1 Hz, 3H), 1.16-1.12 (m, 2H), 0.71 (t, *J* = 7.4 Hz, 3H), 0.68 – 0.60 (m, 2H). ^{13}C NMR (126 MHz, $CDCl_3$) δ 174.1, 166.6, 151.2, 149.5, 145.5, 139.8, 139.4, 138.8, 133.3, 130.1, 129.2, 128.0, 127.1, 127.0, 123.7, 122.0, 121.1, 120.4, 61.0, 52.4, 43.3, 38.8, 25.7, 22.8, 14.4, 13.8. HRMS (m/z, ESI): Calcd. for Chemical Formula: $C_{28}H_{26}ClO_4^-$ $[M-H]^-$: 461.1525, Found: 461.1530. HPLC analysis of the reaction product: Daicel Chiralpak IA, hexane/iso-propanol = 90: 10, 1.0 mL/min, λ= 331 nm, retention time: 6.16 min (major) and 10.84 min (minor).

(*R*)-3-(9-butyl-2-chloro-7-(4-(ethoxycarbonyl)phenyl)-9H-fluoren-9-yl)propanoic acid (8)

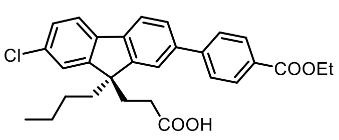

White solid, 66.3 mg, 56% yield, $[\alpha]_D^{20}$ = +5.30 (*c* 0.100 $CHCl_3$) for 90.5:9.5 er. ^1H NMR (500 MHz, $CDCl_3$) δ 8.17 – 8.12 (m, 2H), 7.76 (d, *J* = 7.9 Hz, 1H), 7.73 – 7.69 (m, 2H), 7.67 (d, *J* = 8.0 Hz, 1H), 7.64 (dd, *J* = 7.9, 1.6 Hz, 1H), 7.58 (d, *J* = 1.3 Hz, 1H), 7.39 – 7.32 (m, 2H), 4.44 (q, *J* = 7.1 Hz, 2H), 2.45 – 2.32 (m, 2H), 2.08 – 1.99 (m, 2H), 1.62 (t, *J* = 8.1 Hz, 2H), 1.45 (t, *J* = 7.1 Hz, 3H), 1.15-1.10 (m, 2H), 0.73 – 0.63 (m, 5H). ^{13}C NMR (126 MHz, $CDCl_3$) δ 166.5, 151.2, 149.6, 145.4, 140.2, 139.6, 139.2, 133.5, 130.1, 127.9, 127.0, 127.0, 123.4, 121.6, 121.2, 120.4, 61.1, 54.6, 40.0, 34.4, 28.6, 25.9, 22.9, 14.4, 13.8. HRMS (m/z, ESI): Calcd. for Chemical Formula: $C_{29}H_{28}ClO_4^-$ $[M-H]^-$: 475.1682, Found: 475.1678. HPLC analysis of the reaction product: Daicel Chiralpak IA, hexane/*iso*-propanol = 90: 10, 1.0 mL/min, λ= 320 nm, retention time: 5.58 min (major) and 6.17 min (minor).

(*R*)-4-(9-butyl-2-chloro-7-(4-(ethoxycarbonyl)phenyl)-9H-fluoren-9-yl)butanoic acid (3)

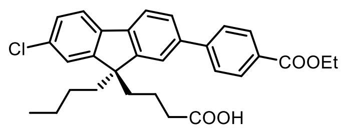

White solid, 82.4 mg, 67% yield, $[\alpha]_D^{20}$ = +1.20 (*c* 0.080 $CHCl_3$) for 96:4 er. ^1H NMR (500 MHz, $CDCl_3$) δ 8.15 (d, *J* = 8.2 Hz, 2H), 7.76 (d, *J* = 7.9 Hz, 1H), 7.73 (d, *J* = 8.2 Hz, 2H), 7.67 (d, *J* = 8.7 Hz, 1H), 7.63 (dd, *J* = 7.9, 1.3 Hz, 1H), 7.58 (d, *J* = 1.5 Hz, 1H), 7.36 (dd, *J* = 6.9, 1.6 Hz, 2H), 4.44 (q, *J* = 7.1 Hz, 2H), 2.16 – 1.99 (m, 6H), 1.44 (t, *J* = 7.2 Hz, 3H), 1.14-1.09

(m, 2H), 1.02-0.98 (m, 2H), 0.71 (t, *J* = 7.3 Hz, 3H), 0.64 (t, *J* = 8.0 Hz, 2H). ^{13}C NMR (126 MHz, $CDCl_3$) δ 166.6, 152.2, 150.6, 145.7, 140.1, 139.4, 139.1, 133.3, 130.1, 127.6, 127.1, 126.7, 123.3, 121.6, 121.1, 120.3, 61.0, 55.2, 40.2, 39.4, 33.5, 25.8, 22.9, 19.1, 14.4, 13.8. HRMS (m/z, ESI): Calcd. for Chemical Formula: $C_{30}H_{30}ClO_4^-$ [M-H]$^-$: 489.1837, Found: 489.1839. HPLC analysis of the reaction product: Daicel Chiralpak IA, hexane/*iso*-propanol = 90: 10, 1.0 mL/min, λ= 272 nm, retention time: 4.87 min (major) and 6.05 min (minor).

(*R*)-5-(9-butyl-2-chloro-7-(4-(ethoxycarbonyl)phenyl)-9H-fluoren-9-yl)pentanoic acid (9)

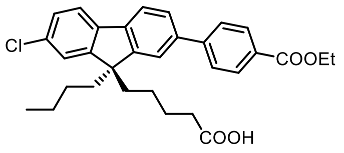

White solid, 75.9 mg, 60% yield, $[\alpha]_D^{20}$ = +12.4 (*c* 0.100 $CHCl_3$) for 95.5:4.5 er. ^{1}H NMR (300 MHz, $CDCl_3$) δ 8.17 (d, *J* = 8.3 Hz, 2H), 7.77-7.74 (m, 1H), 7.74 – 7.72 (m, 2H), 7.67-7.62 (m, 2H), 7.5 (s, 1H), 7.37 (dd, *J* = 6.5, 1.8 Hz, 2H), 4.45 (q, *J* = 7.1 Hz, 2H), 2.15-2.00 (m, 6H), 1.51 – 1.36 (m, 5H), 1.15-1.12 (m, 2H), 0.94 – 0.54 (m, 7H). ^{13}C NMR (75 MHz, $CDCl_3$) δ 178.6, 166.6, 152.5, 150.9, 145.7, 140.2, 139.3, 139.1, 133.3, 130.1, 129.2, 127.4, 127.0, 126.6, 123.3, 121.5, 120.9, 120.2, 61.0, 55.3, 40.1, 39.9, 33.5, 33.4, 25.9, 24.9, 23.3, 23.0, 14.4, 13.8. HRMS (m/z, ESI): Calcd. for Chemical Formula: $C_{31}H_{32}ClO_4^-$ [M-H]$^-$: 503.1995, Found: 503.1993. HPLC analysis of the reaction product: Daicel Chiralpak IA, hexane/*iso*-propanol = 90: 10, 1.0 mL/min, λ= 320 nm, retention time: 14.54 min (major) and 16.07 min (minor).

(*R*)-6-(9-butyl-2-chloro-7-(4-(ethoxycarbonyl)phenyl)-9H-fluoren-9-yl)hexanoic acid (10)

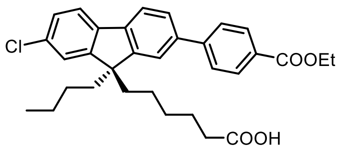

White solid, 81.5 mg, 63% yield, $[\alpha]_D^{20}$ = +6.90 (*c* 0.100 $CHCl_3$) for 93.5:6.5 er. ^{1}H NMR (500 MHz, $CDCl_3$) δ 8.16 (d, *J* = 8.3 Hz, 2H), 7.75 (dd, *J* = 10.7, 8.2 Hz, 3H), 7.68 – 7.61 (m, 2H), 7.57 (s, 1H), 7.38 – 7.31 (m, 2H), 4.44 (q, *J* = 7.1 Hz, 2H), 2.17 (t, *J* = 7.5 Hz, 2H), 2.04-2.00 (m, 4H), 1.47 – 1.39 (m, 5H), 1.18 – 1.08 (m, 4H), 0.74 – 0.62 (m, 7H). ^{13}C NMR (126 MHz, $CDCl_3$) δ 178.4, 166.6, 152.7, 151.1, 145.7, 140.2, 139.3, 139.1, 133.2, 130.1, 129.2, 127.4, 127.0, 126.5, 123.3, 121.5, 120.9, 120.2, 61.0, 55.4, 40.1, 33.6, 29.7, 29.3, 25.9, 24.3, 23.4, 23.0, 14.4, 13.8. HRMS (m/z, ESI): Calcd. for Chemical Formula: $C_{32}H_{34}ClO_4^-$ [M-H]$^-$: 517.2151, Found: 517.2150. HPLC analysis of the reaction product: Daicel Chiralpak IA, hexane/*iso*-propanol = 98: 2, 1.0 mL/min, λ= 324 nm, retention time: 13.09 min (minor) and 15.24 min (major).

(*R*)-7-(9-butyl-2-chloro-7-(4-(ethoxycarbonyl)phenyl)-9H-fluoren-9-yl)heptanoic acid (11)

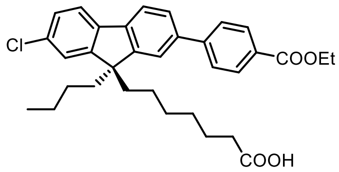

White solid, 72.6 mg, 54% yield, $[\alpha]_D^{20}$ = +9.30 (*c* 0.100 $CHCl_3$) for 89:11 er. ^{1}H NMR (500 MHz, $CDCl_3$) δ 8.16 (d, *J* = 8.3 Hz, 2H), 7.75 (t, *J* = 8.6 Hz, 3H), 7.69 – 7.60 (m, 2H), 7.57 (d, *J* = 1.1 Hz, 1H), 7.35 (dd, *J* = 6.4, 1.9 Hz, 2H), 4.44 (q, *J* = 7.1 Hz, 2H), 2.23 (t, *J* = 7.5 Hz, 2H), 2.06 – 1.96 (m, 4H), 1.49 – 1.42 (m, 5H), 1.16 – 1.07 (m, 6H), 0.73-0.60 (m, 7H). ^{13}C NMR (126 MHz, $CDCl_3$) δ 178.6, 166.6, 152.8, 151.2, 145.7, 140.2, 139.2, 139.1, 133.2, 130.1, 129.2, 127.3, 127.0, 126.5, 123.3, 121.5, 120.9, 120.2, 64.7, 61.0, 55.5, 40.2, 40.2, 33.7, 29.5, 28.7, 25.9, 24.5, 23.6, 23.0, 14.4, 13.8. HRMS (m/z, ESI): Calcd. for Chemical Formula: $C_{33}H_{36}ClO_4^-$ [M-H]$^-$: 531.2308, Found: 531.2303. HPLC analysis of the reaction product: Daicel Chiralpak IA, hexane/*iso*-propanol = 98: 2, 1.0 mL/min, λ= 319 nm, retention time: 12.50 min (minor) and 14.31 min (major).

(*R*)-8-(9-butyl-2-chloro-7-(4-(ethoxycarbonyl)phenyl)-9H-fluoren-9-yl)octanoic acid (12)

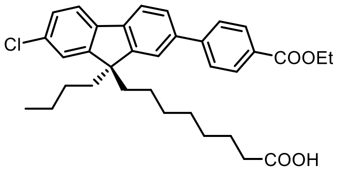

White solid, 43.6 mg, 32% yield, $[\alpha]_D^{20}$ = +2.40 (*c* 0.080 $CHCl_3$) for 86.5:13.5 er. ^{1}H NMR (500 MHz, $CDCl_3$) δ 8.18 – 8.13 (m, 2H), 7.78 – 7.71 (m, 3H), 7.68 – 7.61 (m, 2H), 7.57 (d, *J* = 1.2 Hz, 1H), 7.35 (dd, *J* = 6.6, 1.9 Hz, 2H), 4.44 (q, *J* = 7.1 Hz, 2H), 2.26 (t, *J* = 7.5 Hz, 2H), 2.05 – 1.97 (m, 4H), 1.54 – 1.49 (m, 2H), 1.45 (t, *J* = 8.8 Hz, 3H), 1.19-1.08 (m, 8H), 0.71 (t, *J* = 7.4 Hz, 3H), 0.69 – 0.62 (m, 4H). ^{13}C NMR (126 MHz, $CDCl_3$) δ 166.6, 152.9, 151.2, 145.8, 140.2, 139.2, 139.1, 133.2, 130.1, 129.2, 127.3, 127.0, 126.4, 123.3, 121.5, 120.9, 120.2, 61.0, 55.5, 40.2, 40.1, 33.6, 28.9, 28.8, 25.9, 24.5, 23.7, 23.0, 14.4, 13.8. HRMS (m/z, ESI): Calcd. for Chemical Formula: $C_{34}H_{38}ClO_4^-$ [M-H]$^-$: 545.2464, Found: 545.2465. HPLC analysis of the reaction product: Daicel Chiralpak IH, hexane/*iso*-propanol = 97: 3, 1.0 mL/min, λ= 336 nm, retention time: 12.00 min (major) and 14.12 min (minor).

(*R,E*)-4-(9-butyl-2-chloro-7-(4-(ethoxycarbonyl)phenyl)-9H-fluoren-9-yl)but-2-enoic acid (13)

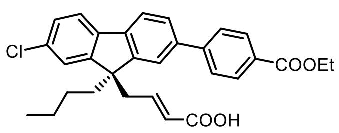

White solid, 70.7 mg, 58% yield, $[\alpha]_D^{20}$ = +57.80 (*c* 0.100 $CHCl_3$) for 91.5:8.5 er. ^{1}H NMR (500 MHz, $CDCl_3$) δ 8.15 (dd, *J* = 6.5, 4.8 Hz, 2H), 7.76 (d, *J* = 7.8 Hz, 1H), 7.73 – 7.69 (m, 2H), 7.68 – 7.63 (m, 2H), 7.57 (d, *J* = 1.1 Hz, 1H), 7.39 – 7.34 (m, 2H), 6.44 (dt, *J* = 15.3, 7.5 Hz, 1H), 5.63 (d, *J* = 15.5 Hz, 1H), 4.44 (q, *J* = 7.1 Hz, 2H), 2.89-2.85 (m, 2H), 2.12 – 2.03

(m, 2H), 1.45 (t, *J* = 7.1 Hz, 3H), 1.14-1.11 (m, 2H), 0.72 (t, *J* = 10.0 Hz, 3H), 0.69 – 0.62 (m, 2H). ^{13}C NMR (126 MHz, $CDCl_3$) δ 170.0, 166.5, 151.1, 149.4, 146.5, 145.5, 139.8, 139.5, 138.8, 133.4, 130.1, 129.3, 128.0, 127.1, 127.1, 123.4, 123.2, 121.8, 121.3, 120.6, 61.0, 54.7, 42.8, 39.1, 25.8, 22.9, 14.4, 13.8. HRMS (m/z, ESI): Calcd. for Chemical Formula: $C_{30}H_{28}ClO_4^-$ $[M-H]^-$: 487.1682, Found: 487.1676. HPLC analysis of the reaction product: Daicel Chiralpak IH, hexane/*iso*-propanol = 95: 5, 1.0 mL/min, λ= 339 nm, retention time: 10.05 min (minor) and 12.69 min (major).

(*R*)-diethyl 4,4'-(9-butyl-9-(3-(ethoxysulfonyl)propyl)-9H-fluorene-2,7-diyl)dibenzoate (2)

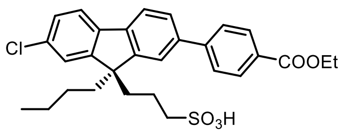

After the reaction was completed, the reaction mixture was neutralized with 1 M HCl to pH 2-3, extracted with ethyl acetate, dried with Na_2SO_4, the organic phase was removed under reduced pressure.

The product was then converted to the corresponding ethyl sulfonate using triethyl orthoacetate[12] for isolation, characterization, and HPLC analysis. White solid, 90.5 mg, 65% yield, $[\alpha]_D^{20}$ = +9.667 (*c* 0.030 $CHCl_3$) for 95:5 er. ^{1}H NMR (500 MHz, $CDCl_3$) δ 8.19 – 8.13 (m, 2H), 7.77 (d, *J* = 7.9 Hz, 1H), 7.75 – 7.71 (m, 2H), 7.70 – 7.63 (m, 2H), 7.59 (d, *J* = 1.2 Hz, 1H), 7.40 – 7.34 (m, 2H), 4.44 (q, *J* = 7.1 Hz, 2H), 4.13 – 4.05 (m, 2H), 2.88 – 2.81 (m, 2H), 2.30 – 2.18 (m, 2H), 2.06-2.03 (m, 2H), 1.45 (t, *J* = 7.1 Hz, 3H), 1.25-1.20 (m, 5H), 1.15-1.12 (m, 2H), 0.71 (t, *J* = 7.4 Hz, 3H), 0.67-0.63 (m, 2H). ^{13}C NMR (126 MHz, $CDCl_3$) δ 151.5, 149.9, 145.5, 139.6, 139.1, 139.1, 133.5, 130.2, 127.8, 127.1, 127.0, 123.2, 121.5, 121.2, 120.5, 66.0, 61.1, 55.1, 50.4, 40.1, 38.3, 25.8, 22.9, 18.4, 15.0, 14.4, 13.8. HRMS (m/z, ESI): Calcd. for Chemical Formula: $C_{31}H_{36}ClO_5SNa^+$$[M+Na]^+$: 577.1786, Found: 577.1779. HPLC analysis of the esterification product: Daicel Chiralpak IA, hexane/*iso*-propanol = 97: 3, 1.0 mL/min, λ= 322 nm, retention time: 14.97 min (major) and 17.49 min (minor).

Ethyl (*R*)-4-(9-butyl-2-chloro-7-(4-methoxyphenyl)-9H-fluoren-9-yl)butane-1-sulfonate (14)

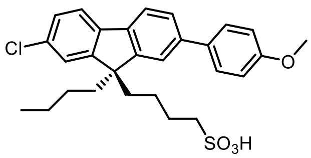

After the reaction was completed, the reaction mixture was neutralized with 1 M HCl to pH 2-3, extracted with ethyl acetate, dried with Na_2SO_4, the organic phase was removed under reduced pressure.

The product was then converted to the corresponding ethyl sulfonate using triethyl orthoacetate[12] for isolation, characterization, and HPLC analysis. White solid, 89.0 mg, 68% yield, $[\alpha]_D^{20}$ = +14.00 (*c* 0.020 $CHCl_3$) for 94.5:5.5 er.

^{1}H NMR (500 MHz, $CDCl_3$) δ 7.72 (d, *J* = 7.7 Hz, 1H), 7.64 (d, *J* = 8.0 Hz, 1H), 7.63 – 7.59 (m, 2H), 7.57 (dd, *J* = 7.9, 1.7 Hz, 1H), 7.49 (d, *J* = 1.2 Hz, 1H), 7.35 (dd, *J* = 8.0, 1.9 Hz, 1H), 7.32 (d, *J* = 1.6 Hz, 1H), 7.06 – 7.01 (m, 2H), 4.17 (q, *J* = 7.1 Hz, 2H), 3.90 (s, 3H), 2.87-2.83 (m, 2H), 2.09 – 2.00 (m, 4H), 1.69-1.62 (m, 2H), 1.32 (t, *J* = 7.1 Hz, 3H), 1.14-1.08 (m, 2H), 0.80 – 0.64 (m, 7H). ^{13}C NMR (126 MHz, $CDCl_3$) δ 159.3, 152.0, 150.3, 140.4, 139.5, 138.7, 133.8, 132.9, 128.2, 127.5, 126.0, 123.1, 120.9, 120.7, 120.1, 114.3, 65.8, 55.4, 55.1, 50.2, 40.2, 39.8, 25.8, 23.7, 23.0, 22.6, 15.1, 13.8. MS (m/z, ESI): Calcd. for Chemical Formula: $C_{30}H_{34}ClO_4S^-$ [M-H]$^-$: 497.1559, Found: 497.1568. HPLC analysis of the esterification product: Daicel Chiralpak IA, hexane/*iso*-propanol = 97: 3, 1.0 mL/min, λ= 325 nm, retention time: 8.07 min (minor) and 9.58 min (major).

(*R*)-4-(2-chloro-7-(4-(ethoxycarbonyl)phenyl)-9-methyl-9H-fluoren-9-yl)butanoic acid (15)

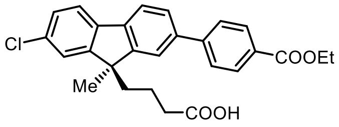

White solid, 69.2 mg, 62% yield, $[\alpha]_D^{20}$ = +37.70 (*c* 0.100 $CHCl_3$) for 89:11 er. ^{1}H NMR (500 MHz, $CDCl_3$) δ 8.16 – 8.11 (m, 2H), 7.77 (dd, *J* = 7.7, 0.8 Hz, 1H), 7.74 – 7.69 (m, 2H), 7.67 (d, *J* = 7.9 Hz, 1H), 7.65 – 7.60 (m, 2H), 7.39 (d, *J* = 1.6 Hz, 1H), 7.36 (dd, *J* = 8.0, 1.9 Hz, 1H), 4.43 (q, *J* = 7.1 Hz, 2H), 2.17 – 2.06 (m, 4H), 1.53 (s, 3H), 1.44 (t, *J* = 7.1 Hz, 3H), 1.07-1.03 (m, 2H). ^{13}C NMR (126 MHz, $CDCl_3$) δ 177.8, 166.6, 153.5, 152.0, 145.6, 139.5, 139.2, 138.1, 133.4, 130.1, 129.2, 127.6, 127.0, 126.8, 123.3, 121.6, 121.2, 120.5, 61.0, 51.0, 39.6, 33.6, 26.8, 19.6, 14.4. MS (m/z, ESI): Calcd. for Chemical Formula: $C_{27}H_{24}ClO_4^-$ [M-H]$^-$: 447.1369, Found: 447.1364. HPLC analysis of the reaction product: Daicel Chiralpak IA, hexane/*iso*-propanol = 90: 10, 1.0 mL/min, λ= 315 nm, retention time: 6.12 min (major) and 9.40 min (minor).

(*R*)-4-(2-chloro-7-(4-(ethoxycarbonyl)phenyl)-9-octyl-9H-fluoren-9-yl)butanoic acid (16)

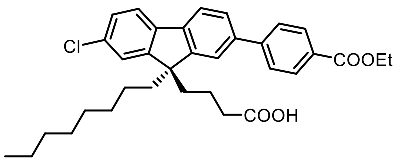

White solid, 96.8 mg, 71% yield, $[\alpha]_D^{20}$ = -4.60 (*c* 0.100 $CHCl_3$) for 94.5:5.5 er. ^{1}H NMR (500 MHz, $CDCl_3$) δ 8.16 – 8.11 (m, 2H), 7.75 (d, *J* = 7.9 Hz, 1H), 7.74 – 7.70 (m, 2H), 7.66 (d, *J* = 8.7 Hz, 1H), 7.62 (dd, *J* = 7.9, 1.6 Hz, 1H), 7.57 (d, *J* = 1.2 Hz, 1H), 7.35 (dd, *J* = 6.7, 1.9 Hz, 2H), 4.43 (q, *J* = 7.1 Hz, 2H), 2.14 (t, *J* = 7.3 Hz, 2H), 2.10 – 1.93 (m, 4H), 1.44 (t, *J* = 7.1 Hz, 3H), 1.24 – 1.18 (m, 2H), 1.15 – 0.96 (m, 10H), 0.82 (t, *J* = 7.2 Hz, 3H), 0.70 – 0.63 (m, 2H). ^{13}C NMR (126 MHz, $CDCl_3$) δ 178.0, 166.6, 152.2, 150.6, 145.7, 140.1, 139.4, 139.1, 133.3, 130.1, 129.2, 127.5, 127.0,

126.7, 123.3, 121.6, 121.0, 120.3, 61.0, 55.3, 40.3, 39.3, 33.7, 31.7, 29.8, 29.1_1, 29.1_2, 23.6, 22.6, 19.1, 14.4, 14.1. HRMS (m/z, ESI): Calcd. for Chemical Formula: $C_{34}H_{38}ClO_4^-$[M-H]$^-$: 545.2464, Found: 545.2461. HPLC analysis of the reaction product: Daicel Chiralpak IA, hexane/*iso*-propanol = 90: 10, 1.0 mL/min, λ= 330 nm, retention time: 4.33 min (major) and 5.11 min (minor).

(*S*)-4-(9-benzyl-2-chloro-7-(4-(ethoxycarbonyl)phenyl)-9H-fluoren-9-yl)butanoic acid (17)

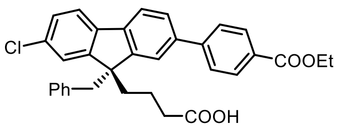

White solid, 86.4 mg, 66% yield, $[\alpha]_D^{20}$ = -56.70 (*c* 0.100 $CHCl_3$) for 92.5:7.5 er. ^1H NMR (500 MHz, $CDCl_3$) δ 8.17 – 8.10 (m, 2H), 7.70 – 7.65 (m, 2H), 7.63 (d, *J* = 7.7 Hz, 1H), 7.59 (dd, *J* = 7.9, 1.6 Hz, 1H), 7.54 (dd, *J* = 7.5, 1.1 Hz, 1H), 7.44 (d, *J* = 1.2 Hz, 1H), 7.32 (dd, *J* = 7.9, 1.5 Hz, 2H), 7.14 – 7.08 (m, 1H), 7.08 – 7.02 (m, 2H), 6.77 – 6.69 (m, 2H), 4.44 (q, *J* = 7.1 Hz, 2H), 3.16 (AB, 2H), 2.32 – 2.20 (m, 2H), 2.16 (t, *J* = 7.3 Hz, 2H), 1.45 (t, *J* = 7.1 Hz, 3H), 1.03 – 0.95 (m, 2H). ^{13}C NMR (126 MHz, $CDCl_3$) δ 177.8, 166.6, 151.3, 149.3, 145.6, 139.9, 138.8, 136.6, 133.0, 130.4, 130.1, 129.1, 127.7, 127.4, 127.0, 126.8, 126.3, 124.1, 122.7, 121.1, 120.3, , 61.0, 55.9, 46.9, 37.5, 33.6, 19.2, 14.4. HRMS (m/z, ESI): Calcd. for Chemical Formula: $C_{33}H_{28}ClO_4^-$ [M-H]$^-$: 523.1682, Found: 523.1677. HPLC analysis of the reaction product: Daicel Chiralpak IA, hexane/*iso*-propanol = 90: 10, 1.0 mL/min, λ= 330 nm, retention time: 6.76 min (major) and 15.04 min (minor).

(*S*)-4-(2-chloro-9-cyclohexyl-7-(4-(ethoxycarbonyl)phenyl)-9H-fluoren-9-yl)butanoic acid (18)

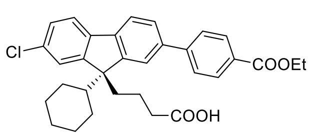

White solid, 79.6 mg, 61% yield, $[\alpha]_D^{20}$ = -5.80 (*c* 0.100 $CHCl_3$) for 91:9 er. ^1H NMR (500 MHz, $CDCl_3$) δ 8.16 – 8.12 (m, 2H), 7.74-7.71 (m, 3H), 7.66 – 7.59 (m, 3H), 7.38 – 7.33 (m, 2H), 4.43 (q, *J* = 7.1 Hz, 2H), 2.25 – 2.10 (m, 4H), 1.94 – 1.87 (m, 1H), 1.68 – 1.51 (m, 6H), 1.44 (t, *J* = 7.1 Hz, 3H), 1.19 – 1.11 (m, 2H), 1.00 – 0.84 (m, 4H). ^{13}C NMR (126 MHz, $CDCl_3$) δ 177.4, 166.6, 151.9, 150.3, 145.7, 140.5, 139.5, 139.1, 133.1, 130.1, 129.1, 127.4, 127.1, 126.6, 123.9, 122.3, 120.8, 120.0, 61.0, 58.6, 47.7, 35.6, 33.7, 27.9, 27.8, 26.9, 26.4, 19.2, 14.4. HRMS (m/z, ESI): Calcd. for Chemical Formula: $C_{32}H_{32}ClO_4^-$ [M-H]$^-$: 515.1995, Found: 515.1989. HPLC analysis of the reaction product: Daicel Chiralpak IA, hexane/*iso*-propanol = 95: 5, 1.0 mL/min, λ= 321 nm, retention time: 7.63 min (major) and 9.39 min (minor).

(*S*)-4-(2-chloro-7-(4-(ethoxycarbonyl)phenyl)-9-phenyl-9H-fluoren-9-yl)butanoic acid (19)

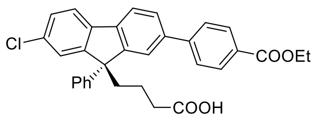

White solid, 80.1 mg, 63% yield, $[\alpha]_D^{20}$ = -55.20 (*c* 0.100 $CHCl_3$) for 94.5:5.5 er. ^{1}H NMR (500 MHz, $CDCl_3$) δ 8.08 (d, *J* = 8.5 Hz, 2H), 7.81 (d, *J* = 7.9 Hz, 1H), 7.72 (d, *J* = 8.1 Hz, 1H), 7.66 – 7.61 (m, 3H), 7.46 (d, *J* = 1.2 Hz, 1H), 7.36 (dd, *J* = 8.1, 1.9 Hz, 1H), 7.27 – 7.18 (m, 6H), 4.41 (q, *J* = 7.1 Hz, 2H), 2.63-2.53 (m, 2H), 2.26 (t, *J* = 7.2 Hz, 2H), 1.42 (t, *J* = 7.1 Hz, 3H), 1.17 – 1.10 (m, 2H). ^{13}C NMR (126 MHz, $CDCl_3$) δ 178.7, 166.5, 153.5, 151.9, 145.4, 143.6, 139.8, 138.7, 133.7, 130.0, 129.2, 128.6, 127.9, 127.0, 126.9, 126.5, 124.7, 123.1, 121.2, 120.5, 61.0, 58.8, 36.9, 33.8, 19.2, 14.4. HRMS (m/z, ESI): Calcd. for Chemical Formula: $C_{32}H_{26}ClO_4^-$ $[M-H]^-$: 509.1525, Found: 509.1522. HPLC analysis of the reaction product: Daicel Chiralpak IA, hexane/*iso*-propanol = 95: 5, 1.0 mL/min, λ= 340 nm, retention time: 12.33 min (major) and 17.25 min (minor).

(*R*)-4-(9-butyl-2-chloro-7-(4-methoxyphenyl)-9H-fluoren-9-yl)butanoic acid (20)

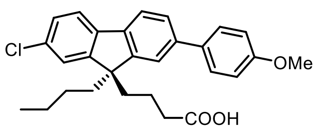

White solid, 76.2 mg, 68% yield, $[\alpha]_D^{20}$ = +5.00 (*c* 0.100 $CHCl_3$) for 96:4 er. ^{1}H NMR (500 MHz, $CDCl_3$) δ 7.71 (d, *J* = 7.9 Hz, 1H), 7.63 (d, *J* = 8.7 Hz, 1H), 7.62 – 7.58 (m, 2H), 7.55 (dd, *J* = 7.9, 1.7 Hz, 1H), 7.50 (d, *J* = 1.2 Hz, 1H), 7.34 (dd, *J* = 6.6, 1.9 Hz, 2H), 7.04 – 7.00 (m, 2H), 3.89 (s, 3H), 2.12 (t, *J* = 7.4 Hz, 2H), 2.08 – 1.93 (m, 4H), 1.11 (dd, *J* = 14.8, 7.4 Hz, 2H), 1.05 – 0.98 (m, 2H), 0.70 (t, *J* = 7.4 Hz, 3H), 0.68 – 0.61 (m, 2H). ^{13}C NMR (126 MHz, $CDCl_3$) δ 177.3, 159.2, 152.0, 150.4, 140.3, 139.5, 138.7, 133.9, 132.8, 128.2, 127.4, 126.0, 123.2, 121.1, 120.7, 120.1, 114.3, 55.4, 55.1, 40.2, 39.5, 33.7, 25.8, 23.0, 19.2, 13.8. HRMS (m/z, ESI): Calcd. for Chemical Formula: $C_{28}H_{28}ClO_3^-$ $[M-H]^-$: 447.1732, Found: 447.1727. HPLC analysis of the reaction product: Daicel Chiralpak IH, hexane/*iso*-propanol = 99: 1, 1.0 mL/min, λ= 327 nm, retention time: 19.36 min (minor) and 21.70 min (major).

(*R*)-4-(9-butyl-2-chloro-7-(4-(diphenylamino)phenyl)-9H-fluoren-9-yl)butanoic acid (21)

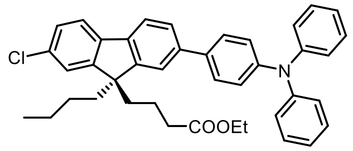

After the reaction was completed, the reaction mixture was neutralized with 1 M HCl to pH 2-3 and the crude product was dissolved in EtOH, then 10 μL conc. H_2SO_4 was added and the reaction mixture was stirred at 65 °C for 10 h. The esterification product was obtained as a white solid, 111.8 mg, 73% yield, $[\alpha]_D^{20}$ = +5.10 (*c* 0.100 $CHCl_3$) for 95.5:4.5 er. ^{1}H NMR (500 MHz, $CDCl_3$) δ 7.73 (d, *J* = 7.9 Hz, 1H), 7.65 (d, *J* = 8.6 Hz, 1H), 7.62 – 7.52 (m, 4H), 7.39 –

7.28 (m, 6H), 7.25 – 7.15 (m, 6H), 7.08 (dd, *J* = 10.6, 4.0 Hz, 2H), 4.12 (q, *J* = 7.1 Hz, 2H), 2.12 – 2.00 (m, 6H), 1.24 – 1.17 (m, 3H), 1.13-1.12 (m, 2H), 1.05-1.02 (m, 2H), 0.73-0.65 (m, 5H). ^{13}C NMR (126 MHz, $CDCl_3$) δ 173.3, 152.2, 150.5, 147.7, 147.3, 140.0, 139.5, 138.9, 135.3, 132.8, 129.3, 127.8, 127.4, 125.9, 124.4, 124.0, 123.2, 123.0, 120.9, 120.8, 120.2, 60.2, 55.2, 40.2, 39.6, 34.4, 25.9, 23.0, 19.5, 14.2, 13.8. HRMS (m/z, ESI): Calcd. for Chemical Formula: $C_{41}H_{41}ClNO_2^+$[M+H]$^+$: 614.2767, Found: 614.2782.

A sample of the ester was hydrolyzed to provide the parent carboxylic acid for the HPLC analysis of the reaction product: Daicel Chiralpak IA, hexane/*iso*-propanol = 99.4: 0.6, 1.0 mL/min, λ= 348 nm, retention time: 9.98 min (major) and 10.26 min (minor).

(*R*)-4-(9-butyl-2-chloro-7-(4-nitrophenyl)-9H-fluoren-9-yl)butanoic acid (22)

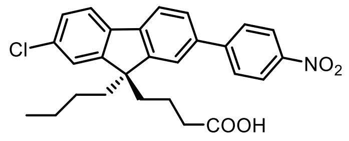

Light yellow solid, 71.0 mg, 61% yield, $[\alpha]_D^{20}$ = +7.00 (*c* 0.100 $CHCl_3$) for 96:4 er. ^{1}H NMR (500 MHz, $CDCl_3$) δ 8.30 – 8.26 (m, 2H), 7.79 – 7.75 (m, 3H), 7.69 (d, *J* = 8.4 Hz, 1H), 7.60 (dd, *J* = 7.9, 1.6 Hz, 1H), 7.57 (d, *J* = 1.4 Hz, 1H), 7.39 – 7.35 (m, 2H), 2.17 – 1.99 (m, 6H), 1.13-1.09 (m, 2H), 1.01-1.97 (m, 2H), 0.70 (t, *J* = 7.3 Hz, 3H), 0.67 – 0.61 (m, 2H). ^{13}C NMR (126 MHz, $CDCl_3$) δ 179.0, 152.1, 150.8, 147.7, 146.9, 141.0, 138.8, 137.9, 133.7, 127.8, 127.7, 126.8, 124.1, 123.3, 121.7, 121.3, 120.5, 76.8, 55.3, 40.3, 39.2, 33.7, 25.8, 22.9, 19.0, 13.8. HRMS (m/z, ESI): Calcd. for Chemical Formula: $C_{27}H_{25}ClNO_4^-$ [M-H]$^-$: 462.1478, Found: 462.1474. HPLC analysis of the reaction product: Daicel Chiralpak IA, hexane/*iso*-propanol = 95: 5, 1.0 mL/min, λ= 340 nm, retention time: 8.67 min (major) and 9.84 min (minor).

(*R*)-4-(2-(3-(benzyloxy)phenyl)-9-butyl-7-chloro-9H-fluoren-9-yl)butanoic acid (23)

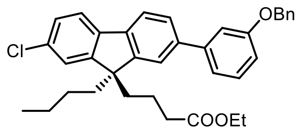

After the reaction was completed, the reaction mixture was neutralized with 1 M HCl to pH 2-3 and the crude product was dissolved in EtOH, then 10 μL conc. H_2SO_4 was added and the reaction mixture was stirred at 65 ^{o}C for 10 h. The esterification product was obtained as a white solid, 85.6 mg, 62% yield, $[\alpha]_D^{20}$ = +3.60 (*c* 0.100 $CHCl_3$) for 97.5:2.5 er. ^{1}H NMR (500 MHz, $CDCl_3$) δ 7.74 (d, *J* = 7.9 Hz, 1H), 7.68 – 7.64 (m, 1H), 7.60 (d, *J* = 7.9 Hz, 1H), 7.56 – 7.49 (m, 3H), 7.46-7.30 (m, 8H), 7.02 (dd, *J* = 8.2, 1.8 Hz, 1H), 5.19 (s, 2H), 4.10 – 3.98 (m, 2H), 2.14 – 1.98 (m, 6H), 1.19 (t, *J* = 7.1 Hz, 3H), 1.14-1.10 (m, 2H), 1.07 – 0.98 (m, 2H), 0.72 (t, *J* = 7.3 Hz, 3H), 0.68-0.65 (m, 2H). ^{13}C NMR (126 MHz, $CDCl_3$) δ 173.3, 159.2, 152.3, 150.5, 142.9, 140.3, 139.5,

139.4, 137.0, 133.0, 129.9, 128.7, 128.1, 127.6, 127.4, 126.4, 123.3, 121.6, 120.9, 120.1, 120.0, 114.2, 113.3, 70.2, 60.2, 55.2, 40.2, 39.5, 34.3, 25.9, 23.0, 19.5, 14.2, 13.8. HRMS (m/z, ESI): Calcd. for Chemical Formula: $C_{36}H_{37}ClNaO_3^+$ $[M+Na]^+$: 575.2323, Found: 575.2322. HPLC analysis: Daicel Chiralpak IB, hexane/*iso*-propanol = 99: 1, 1.0 mL/min, λ= 318 nm, retention time: 7.00 min (minor) and 8.29 min (major).

(*R*)-4-(9-butyl-2-chloro-7-(3-(ethoxycarbonyl)phenyl)-9H-fluoren-9-yl)butanoic acid (24)

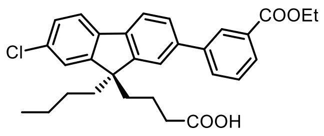

White solid, 75.7 mg, 62% yield, $[\alpha]_D^{20}$ = -9.60 (*c* 0.100 $CHCl_3$) for 96:4 er. ^{1}H NMR (500 MHz, $CDCl_3$) δ 8.34 (t, *J* = 1.6 Hz, 1H), 8.08 – 8.02 (m, 1H), 7.85 (ddd, *J* = 7.7, 1.9, 1.2 Hz, 1H), 7.76 (d, *J* = 7.9 Hz, 1H), 7.64 (ddd, *J* = 9.5, 7.5, 1.6 Hz, 2H), 7.58 – 7.51 (m, 2H), 7.35 (dd, *J* = 7.4, 1.7 Hz, 2H), 4.45 (q, *J* = 7.1 Hz, 2H), 2.15 – 1.97 (m, 6H), 1.45 (t, *J* = 7.1 Hz, 3H), 1.12-1.07 (m, 2H), 1.03 – 0.96 (m, 2H), 0.71 (t, *J* = 7.4 Hz, 3H), 0.66-0.62 (m, 2H). ^{13}C NMR (126 MHz, $CDCl_3$) δ 177.7, 166.8, 152.1, 150.5, 141.7, 139.7, 139.6, 139.2, 133.2, 131.6, 131.0, 128.9, 128.3, 128.3, 127.5, 126.5, 123.3, 121.6, 121.0, 120.3, 61.2, 55.2, 40.2, 39.4, 33.7, 25.8, 22.9, 19.2, 14.4, 13.8. HRMS (m/z, ESI): Calcd. for Chemical Formula: $C_{30}H_{30}ClO_4^-$$[M-H]^-$: 489.1838, Found: 489.1836. HPLC analysis of the reaction product: Daicel Chiralpak IH, hexane/*iso*-propanol = 95: 5, 1.0 mL/min, λ= 316 nm, retention time: 6.59 min (minor) and 7.19 min (major).

(*R*)-4-(9-butyl-2-chloro-7-(2-methoxyphenyl)-9H-fluoren-9-yl)butanoic acid (25)

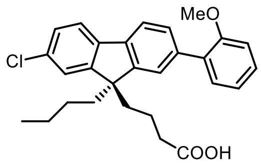

White solid, 76.0 mg, 68% yield, $[\alpha]_D^{20}$ = +140.0 (*c* 0.005 $CHCl_3$) for 96.5:3.5 er. ^{1}H NMR (500 MHz, $CDCl_3$) δ 7.74 – 7.69 (m, 1H), 7.64 (d, *J* = 7.6 Hz, 1H), 7.54 (dd, *J* = 4.0, 2.3 Hz, 2H), 7.41 (d, *J* = 7.5 Hz, 1H), 7.38 – 7.31 (m, 3H), 7.08 (t, *J* = 7.5 Hz, 1H), 7.03 (d, *J* = 8.2 Hz, 1H), 3.83 (s, 3H), 2.14 – 1.92 (m, 6H), 1.18 – 1.04 (m, 6H), 0.75-0.71 (m, 5H). ^{13}C NMR (126 MHz, $CDCl_3$) δ 178.6, 156.6, 152.4, 149.3, 139.6, 138.7, 137.8, 132.8, 130.9, 130.9, 128.6, 128.6, 127.3, 124.4, 123.3, 121.0, 120.7, 119.4, 111.5, 55.7, 40.0, 39.4, 34.2, 25.9, 23.0, 19.4, 13.8. HRMS (m/z, ESI): Calcd. for Chemical Formula: $C_{28}H_{29}ClNaO_3^+$ $[M+Na]^+$: 471.1697, Found: 471.1695. HPLC analysis of the reaction product: Daicel Chiralpak IA, hexane/*iso*-propanol = 99: 1, 1.0 mL/min, λ= 324 nm, retention time: 13.88 min (major) and 15.32 min (minor).

Ethyl (*R*)-4-(9-butyl-2-chloro-7-(2,5-dimethylphenyl)-9H-fluoren-9-yl)butanoate (26)

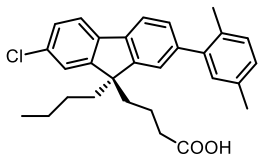

White solid, 64.6 mg, 58% yield, $[\alpha]_D^{20}$ = -1.30 (*c* 0.100 $CHCl_3$) for 95:5 er. ^1H NMR (500 MHz, $CDCl_3$) δ 7.71 (d, *J* = 7.7 Hz, 1H), 7.65 (d, *J* = 7.5 Hz, 1H), 7.33 (dd, *J* = 15.0, 7.6 Hz, 3H), 7.20 (d, *J* = 7.7 Hz, 1H), 7.15 – 7.08 (m, 2H), 2.39 (s, 3H), 2.26 (s, 3H), 2.13-2.08 (m, 2H), 2.06-2.02 (m, 2H), 2.00-1.94 (m, 2H), 1.13-1.08 (m, 2H), 1.06 – 0.97 (m, 2H), 0.74 – 0.62 (m, 5H). ^{13}C NMR (126 MHz, $CDCl_3$) δ 152.0, 149.5, 141.9, 141.4, 139.6, 138.6, 135.3, 132.9, 132.2, 130.5, 130.4, 128.3, 128.0, 127.4, 123.8, 123.2, 120.7, 119.5, 55.1, 40.1, 39.5, 33.8, 25.9, 22.9, 20.9, 20.0, 19.2, 13.8. HRMS (m/z, ESI): Calcd. for Chemical Formula: $C_{29}H_{30}ClO_2^-$ $[M-H]^-$: 445.1940, Found: 445.1939. HPLC analysis of the reaction product: Daicel Chiralpak IA, hexane/*iso*-propanol = 99: 1, 1.0 mL/min, λ= 320 nm, retention time: 9.86 min (major) and 11.75 min (minor).

Ethyl (*R*)-4-(9-butyl-2-chloro-7-(2,4-difluorophenyl)-9H-fluoren-9-yl)butanoate (27)

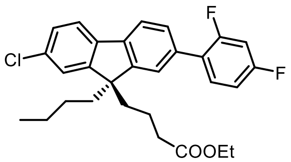

After the reaction was completed, the reaction mixture was neutralized with 1 M HCl to pH 2-3 and the crude product was dissolved in EtOH, then 10μL conc. H_2SO_4 was added and the reaction mixture was stirred at 65 °C for 10 h. The esterification product was obtained as a white solid, 68.6 mg, 57% yield, $[\alpha]_D^{20}$ = -0.40 (*c* 0.100 $CHCl_3$) for 93:7 er. ^1H NMR (500 MHz, $CDCl_3$) δ 7.74 (dd, *J* = 7.9, 0.5 Hz, 1H), 7.67 – 7.63 (m, 1H), 7.52 – 7.46 (m, 3H), 7.35 (dd, *J* = 6.6, 1.9 Hz, 2H), 7.03 – 6.92 (m, 2H), 4.09 – 4.00 (m, 2H), 2.10 – 1.96 (m, 6H), 1.19 (t, *J* = 7.1 Hz, 3H), 1.1-1.09 (m, 2H), 1.06 – 0.98 (m, 2H), 0.72 (t, *J* = 7.4 Hz, 3H), 0.69 – 0.62 (m, 2H). ^{13}C NMR (126 MHz, $CDCl_3$) δ 173.3, 152.3, 150.1, 139.6, 139.2, 134.1, 133.2, 131.5 (dd, *J* = 9.4, 4.9 Hz), 128.1 (d, *J* = 2.3 Hz), 127.5, 123.5 (d, *J* = 2.8 Hz), 123.3, 121.0, 119.9, 111.6 (dd, *J* = 21.1, 3.5 Hz), 104.4 (t, *J* = 25.9 Hz), 104.2, 104.2, 60.2, 55.2, 40.0, 39.5, 34.3, 25.8, 22.9, 19.5, 14.2, 13.8. ^{19}F NMR (471 MHz, $CDCl_3$) δ -111.53 (d, *J* = 7.3 Hz), -113.09 (d, *J* = 7.3 Hz). HRMS (m/z, ESI): Calcd. for Chemical Formula: $C_{29}H_{29}ClF_2NaO_2^+$ $[M+Na]^+$: 505.1716, Found: 505.1708.

A sample of the ester was hydrolyzed to provide the parent carboxylic acid for the HPLC analysis of the reaction product: Daicel Chiralpak IA, hexane/*iso*-propanol = 99: 1, 1.0 mL/min, λ= 317 nm, retention time: 12.33 min (major) and 13.17 min (minor).

Ethyl (*R*)-4-(9-butyl-2-chloro-7-(naphthalen-2-yl)-9H-fluoren-9-yl)butanoate (28)

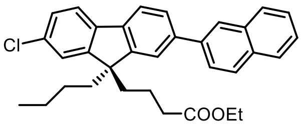

After the reaction was completed, the reaction mixture was neutralized with 1 M HCl to pH 2-3 and the crude product was dissolved in EtOH, then 10μL conc. H_2SO_4 was added and the reaction mixture was stirred at 65 °C for 10 h. The esterification product was obtained as a white solid, 86.5 mg, 70% yield, $[\alpha]_D^{20}$ = +7.60 (*c* 0.100 $CHCl_3$) for 94:6 er. ^{1}H NMR (500 MHz, $CDCl_3$) δ 8.13 (d, *J* = 1.3 Hz, 1H), 7.96 (t, *J* = 7.1 Hz, 2H), 7.91 (d, *J* = 7.8 Hz, 1H), 7.84 (dd, *J* = 8.5, 1.8 Hz, 1H), 7.79 (d, *J* = 7.9 Hz, 1H), 7.74 (dd, *J* = 7.9, 1.6 Hz, 1H), 7.70 – 7.66 (m, 2H), 7.58 – 7.49 (m, 2H), 7.37-7.36 (m, 2H), 4.05 (q, *J* = 7.1 Hz, 2H), 2.17 – 1.98 (m, 6H), 1.19 (t, *J* = 7.1 Hz, 3 H), 1.15-1.13 (m, 2H), 1.09 – 1.00 (m, 2H), 0.76 – 0.65 (m, 5H). ^{13}C NMR (126 MHz, $CDCl_3$) δ 173.3, 152.3, 150.6, 140.5, 139.4, 138.7, 133.7, 133.0, 132.7, 128.5, 128.2, 127.7, 127.4, 126.7, 126.4, 126.0, 125.8, 125.7, 123.3, 121.8, 120.9, 120.3, 60.2, 55.3, 40.2, 39.6, 34.4, 25.9, 23.0, 19.5, 14.2, 13.8. HRMS (m/z, ESI): Calcd. for Chemical Formula: $C_{33}H_{33}ClNaO_2^+$ [M+Na]$^+$: 519.2061, Found: 519.2062.

A sample of the ester was hydrolyzed to provide the parent carboxylic acid for HPLC analysis of the initial reaction product: Daicel Chiralpak IA, hexane/*iso*-propanol = 97: 3, 1.0 mL/min, λ= 321 nm, retention time: 7.63 min (major) and 8.47 min (minor).

Ethyl (*R*)-4-(9-butyl-2-chloro-7-(dibenzo[b,d]furan-4-yl)-9H-fluoren-9-yl)butanoate (29)

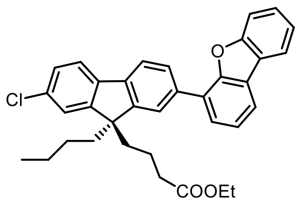

After the reaction was completed, the reaction mixture was neutralized with 1 M HCl to pH 2-3 and the crude product was dissolved in EtOH, then 10μL conc. H_2SO_4 was added and the reaction mixture was stirred at 65 °C for 10 h. The esterification product was obtained as a white solid, 83.0 mg, 62% yield, $[\alpha]_D^{20}$ = +16.00 (*c* 0.100 $CHCl_3$) for 96:4 er. ^{1}H NMR (500 MHz, $CDCl_3$) δ 8.05 – 8.01 (m, 1H), 8.00 – 7.93 (m, 2H), 7.90 (s, 1H), 7.85 (d, *J* = 7.9 Hz, 1H), 7.72 – 7.68 (m, 2H), 7.64 (d, *J* = 8.2 Hz, 1H), 7.50 (dt, *J* = 15.2, 7.4 Hz, 2H), 7.43 – 7.35 (m, 3H), 4.05 (q, *J* = 7.1 Hz, 2H), 2.17 – 1.99 (m, 6H), 1.19-1.12 (m, 7H), 0.78-0.75 (m, 5H). ^{13}C NMR (126 MHz, $CDCl_3$) δ 173.3, 156.2, 153.4, 152.5, 150.2, 139.7, 139.4, 135.6, 133.1, 128.0, 127.5, 127.3, 126.8, 126.2, 125.0, 124.2, 123.4, 123.3_1, 123.3_2, 122.8, 121.0, 120.7, 120.0, 119.7, 111.9, 60.2, 55.2, 40.1, 39.5, 34.5, 25.9, 23.0, 19.6, 14.2, 13.8. HRMS (m/z, ESI): Calcd. for Chemical Formula: $C_{35}H_{33}ClNaO_3^+$ [M+Na]$^+$: 559.2010, Found: 559.2004.

A sample of the ester was hydrolyzed to provide the parent carboxylic acid for the HPLC analysis of the reaction product: Daicel Chiralpak IA, hexane/*iso*-propanol = 97: 3, 1.0 mL/min, λ= 324 nm, retention time: 7.59 min (major) and 8.18 min (minor).

Ethyl (*R*)-4-(9-butyl-7-chloro-9',9'-dimethyl-9H,9'H-[2,2'-bifluoren]-9-yl)butanoate (30)

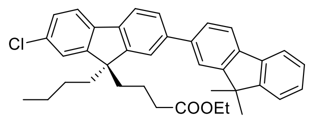

White solid, 91.5 mg, 65% yield, $[\alpha]_D^{20}$ = +3.60 (*c* 0.100 $CHCl_3$) for 98:2 er. ^1H NMR (500 MHz, $CDCl_3$) δ 7.83 (d, *J* = 7.7 Hz, 1H), 7.81 – 7.78 (m, 1H), 7.76 (d, *J* = 7.8 Hz, 1H), 7.73 (d, *J* = 1.2 Hz, 1H), 7.69 – 7.65 (m, 3H), 7.61 (d, *J* = 1.2 Hz, 1H), 7.51 – 7.48 (m, 1H), 7.42 – 7.34 (m, 4H), 4.09 – 4.02 (m, 2H), 2.15 – 2.00 (m, 6H), 1.60 (s, 6H), 1.19 (t, *J* = 7.1 Hz, 3H), 1.16-1.11 (m, 2H), 1.07-1.01 (m, 2H), 0.74 – 0.65 (m, 5H). ^{13}C NMR (126 MHz, $CDCl_3$) δ 173.3, 154.3, 153.9, 152.3, 150.5, 141.0, 140.6, 139.5, 139.2, 138.9, 138.5, 132.9, 127.4, 127.3, 127.1, 126.5, 126.3, 123.3, 122.6, 121.5, 121.4, 120.8, 120.3, 120.1, 120.1, 60.2, 55.2, 47.0, 40.3, 39.6, 34.4, 27.3, 25.9, 23.0, 19.5, 14.2, 13.8. HRMS (m/z, ESI): Calcd. for Chemical Formula: $C_{38}H_{39}ClNaO_2^+$ $[M+Na]^+$: 585.2531, Found: 585.2525.

A sample of the ester was hydrolyzed to provide the parent carboxylic acid for the HPLC analysis of the reaction product: Daicel Chiralpak IA, hexane/*iso*-propanol = 99: 1, 1.0 mL/min, λ= 330 nm, retention time: 22.16 min (major) and 24.66 min (minor).

Ethyl (*R*)-4-(9-butyl-2-chloro-7-(dibenzo[b,d]thiophen-4-yl)-9H-fluoren-9-yl)butanoate (31)

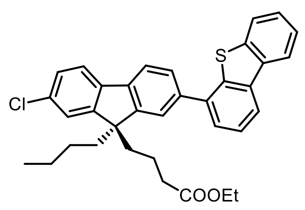

After the reaction was completed, the reaction mixture was neutralized with 1 M HCl to pH 2-3 and the crude product was dissolved in EtOH, then 10µL conc. H_2SO_4 was added and the reaction mixture was stirred at 65 °C for 10 h. The esterification product was obtained as a white solid, 96.4 mg, 70% yield, $[\alpha]_D^{20}$ = +20.50 (*c* 0.100 $CHCl_3$) for 96:4 er. ^1H NMR (500 MHz, $CDCl_3$) δ 8.25 – 8.17 (m, 2H), 7.87 (dd, *J* = 5.7, 3.3 Hz, 1H), 7.83 (d, *J* = 7.8 Hz, 1H), 7.79 (s, 1H), 7.74 – 7.67 (m, 2H), 7.64 – 7.58 (m, 2H), 7.54 – 7.47 (m, 2H), 7.40 – 7.35 (m, 2H), 4.06 (q, *J* = 7.1 Hz, 2H), 2.13-2.07 (m, 6H), 1.21 – 1.08 (m, 7H), 0.80 – 0.70 (m, 5H). ^{13}C NMR (126 MHz, $CDCl_3$) δ 173.3, 152.4, 150.4, 139.9, 139.8, 139.6, 139.3, 138.8, 137.3, 136.3, 135.8, 133.2, 127.6, 127.5, 126.9, 125.2, 124.4, 123.3, 122.8, 122.6, 121.8, 121.0, 120.5, 120.3, 60.2, 55.3, 40.1, 39.5, 34.5, 25.9, 23.0, 19.6, 14.2, 13.9. HRMS (m/z, ESI): Calcd. for

Chemical Formula: $C_{35}H_{33}ClNaO_3S$ [M+Na]$^+$: 575.1782, Found: 575.1781.

A sample of the ester was hydrolyzed to provide the parent carboxylic acid for the HPLC analysis of the reaction product: Daicel Chiralpak IA, hexane/*iso*-propanol = 97: 3, 1.0 mL/min, λ= 320 nm, retention time: 8.37 min (major) and 9.64 min (minor).

Ethyl (*R*)-4-(9-butyl-2-chloro-7-(thianthren-1-yl)-9H-fluoren-9-yl)butanoate (32)

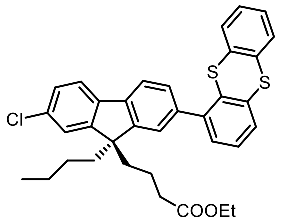

After the reaction was completed, the reaction mixture was neutralized with 1 M HCl to pH 2-3 and the crude product was dissolved in EtOH, then 10μL conc. H_2SO_4 was added and the reaction mixture was stirred at 65 °C for 10 h. The esterification product was obtained as a white solid, 89.0 mg, 61% yield, $[\alpha]_D^{20}$ = +7.80 (*c* 0.100 $CHCl_3$) for 94.5:5.5 er. ^{1}H NMR (500 MHz, $CDCl_3$) δ 7.80 – 7.77 (m, 1H), 7.71 – 7.67 (m, 1H), 7.56 (dd, *J* = 7.6, 1.5 Hz, 1H), 7.53 (dd, *J* = 7.7, 1.2 Hz, 1H), 7.42 (d, *J* = 1.1 Hz, 1H), 7.41 – 7.36 (m, 4H), 7.35-7.32 (m, 2H), 7.26 (td, *J* = 7.5, 1.4 Hz, 1H), 7.19 (td, *J* = 7.5, 1.4 Hz, 1H), 4.05 (q, *J* = 7.1 Hz, 2H), 2.14 – 2.00 (m, 6H), 1.20 – 1.05 (m, 7H), 0.81 – 0.69 (m, 5H). ^{13}C NMR (126 MHz, $CDCl_3$) δ 173.3, 152.4, 149.6, 142.8, 139.7, 139.4, 139.3, 136.4, 136.1, 135.4, 135.2, 133.2, 129.2, 128.8, 128.6, 128.3, 128.3, 127.8, 127.6, 127.5, 127.1, 124.4, 123.3, 121.0, 119.7, 60.2, 55.2, 40.1, 39.6, 34.5, 25.9, 23.1, 19.6, 14.2, 13.9. HRMS (m/z, ESI): Calcd. for Chemical Formula: $C_{35}H_{34}ClO_2S_2^+$ [M+H]$^+$: 585.5275, Found: 585.5309.

A sample of the ester was hydrolyzed to provide the parent carboxylic acid for the HPLC analysis of the reaction product: Daicel Chiralpak IA, hexane/*iso*-propanol = 97: 3, 1.0 mL/min, λ= 320 nm, retention time: 7.08 min (major) and 8.06 min (minor).

Ethyl (*R*)-4-(9-butyl-2-chloro-7-(9-phenyl-9H-carbazol-2-yl)-9H-fluoren-9-yl)butanoate (33)

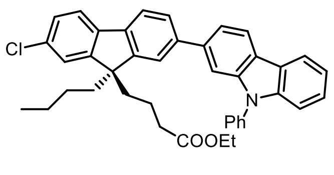

After the reaction was completed, the reaction mixture was neutralized with 1 M HCl to pH 2-3 and the crude product was dissolved in EtOH, then 10 μL conc. H_2SO_4 was added and the reaction mixture was stirred at 65 °C for 10 h. The esterification product was obtained as a white solid, 97.5 mg, 64% yield, $[\alpha]_D^{20}$ = +4.6 (*c* 0.100 $CHCl_3$) for 92.5:7.5 er. ^{1}H NMR (500 MHz, $CDCl_3$) δ 8.47 (s, 1H), 8.29 (d, *J* = 7.7 Hz, 1H), 7.81 – 7.73 (m,

3H), 7.72 – 7.63 (m, 6H), 7.53 (dd, J = 9.2, 4.6 Hz, 2H), 7.48 (d, J = 3.7 Hz, 2H), 7.38-7.36 (m, 3H), 4.08 (q, J = 7.1 Hz, 2H), 2.20 – 2.01 (m, 6H), 1.23 – 1.07 (m, 7H), 0.76-0.73 (m, 5H). ^{13}C NMR (126 MHz, $CDCl_3$) δ 173.3, 152.3, 150.6, 141.5, 140.4, 139.6, 138.6, 137.7, 133.7, 132.8, 130.0, 127.6, 127.6, 127.4, 127.1, 126.6, 126.2, 125.6, 124.0, 123.5, 123.3, 121.7, 120.8, 120.5, 120.2, 120.1, 118.9, 110.1, 110.0, 60.2, 55.2, 40.3, 39.6, 34.4, 25.9, 23.0, 19.5, 14.2, 13.9. HRMS (m/z, ESI): Calcd. for Chemical Formula: $C_{41}H_{38}ClNNaO_2^+$ [M+Na]$^+$: 634.2483, Found: 634.2478.

A sample of the ester was hydrolyzed to provide the parent carboxylic acid for the HPLC analysis of the reaction product: Daicel Chiralpak IA, hexane/*iso*-propanol = 97: 3, 1.0 mL/min, λ= 330 nm, retention time: 10.78 min (major) and 11.99 min (minor).

(*R*)-4-(9-butyl-2-chloro-7-(4-methoxyphenyl)-9H-xanthen-9-yl)butanoic acid (34)

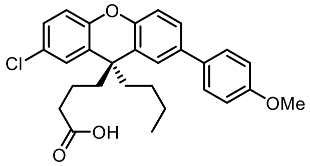

White solid, 64.2 mg, 55% yield, $[\alpha]_D^{20}$ = -3.80 (c 0.50 $CHCl_3$) for 95.5: 4.5 er. ^{1}H NMR (500 MHz, $CDCl_3$) δ 7.50 – 7.46 (m, 2H), 7.41 (d, J = 2.2 Hz, 1H), 7.37 (dd, J = 8.4, 2.2 Hz, 1H), 7.25 (d, J = 2.5 Hz, 1H), 7.16 (dd, J = 8.7, 2.4 Hz, 1H), 7.04 (d, J = 8.4 Hz, 1H), 6.99 – 6.95 (m, 3H), 3.85 (s, 3H), 2.19 (td, J = 7.2, 2.0 Hz, 2H), 2.12 – 2.06 (m, 1H), 1.99 – 1.93 (m, 2H), 1.90 – 1.84 (m, 1H), 1.31 – 1.21 (m, 2H), 1.15 – 1.11 (m, 2H), 0.90 – 0.82 (m, 2H), 0.71 (t, J = 7.3 Hz, 3H). ^{13}C NMR (126 MHz, $CDCl_3$) δ 178.1, 159.1, 150.6, 150.5, 136.4, 133.6, 128.1, 128.0, 127.8, 126.8, 126.4, 126.2, 124.4, 117.9, 116.6, 114.4, 55.5, 45.6, 44.1, 42.7, 33.7, 27.0, 23.0, 20.2, 14.0. HRMS (m/z, ESI): Calcd. for Chemical Formula $C_{28}H_{28}ClO_4^-$ [M-H]$^-$:463.1682, Found: 463.1668. HPLC analysis of the reaction product: Daicel Chiralpak IA, hexane/*iso*-propanol = 95: 5, 1.0 mL/min, λ= 273 nm, retention time: 5.84 min (major) and 8.55 min (minor).

(*S*)-4-(9-butyl-2-chloro-7-(4-methoxyphenyl)-9H-xanthen-9-yl)butanoic acid (*ent*-34)

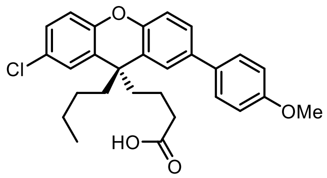

The reaction was carried out using (*R*)-**L4** as the ligand. White solid, 61.0 mg, 53% yield, $[\alpha]_D^{20}$ = +5.83 (c 1.00 $CHCl_3$) for 4: 96 er. HPLC analysis of the reaction product: Daicel Chiralpak IA, hexane/*iso*-propanol = 95: 5, 1.0 mL/min, λ= 273 nm, retention time: 7.87 min (major) and 5.87 min (minor).

(*R*)-5-(9-butyl-2-chloro-7-(4-methoxyphenyl)-9H-xanthen-9-yl)pentanoic acid (35)

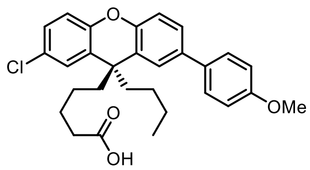

White solid, 50 mg, 42% yield, $[\alpha]_D^{20}$ = -10.00 (*c* 0.040 $CHCl_3$) for 93: 7 er. ^{1}H NMR (500 MHz, $CDCl_3$) δ 7.48 (dt, *J* = 8.7, 2.2 Hz, 2H), 7.43 – 7.34 (m, 2H), 7.25 – 7.22 (m, 1H), 7.15 (dd, *J* = 8.7, 2.1 Hz, 1H), 7.06 – 7.02 (m, 1H), 7.01 – 6.94 (m, 3H), 3.85 (s, 3H), 2.14 (t, *J* = 7.6 Hz, 2H), 2.01 – 1.86 (m, 4H), 1.47 (q, *J* = 7.8 Hz, 2H), 1.17 – 1.12 (m, 2H), 0.96 – 0.83 (m, 4H), 0.72(t, *J* = 7.4 Hz, 3H). ^{13}C NMR (126 MHz, $CDCl_3$) δ 178.4, 159.1, 150.6, 150.5, 136.3, 133.6, 128.1, 128.0, 127.7, 127.2, 126.2, 126.1, 124.7, 124.3, 117.8, 116.6, 114.4, 55.5, 45.3, 45.1, 42.8, 33.6, 27.1, 25.0, 24.5, 23.0, 14.0. HRMS (m/z, ESI): Calcd. for Chemical Formula $C_{29}H_{30}ClO_4^-$ [M-H]$^-$: 477.1838, Found: 477.1841. HPLC analysis of the reaction product: Daicel Chiralpak IA, hexane/*iso*-propanol = 99: 1, 1.0 mL/min, λ= 273 nm, retention time: 22.29 min (major) and 20.59 min (minor).

(*R*)-4-(9-butyl-2-chloro-7-(4-(diphenylamino)phenyl)-9H-xanthen-9-yl)butanoic acid (36)

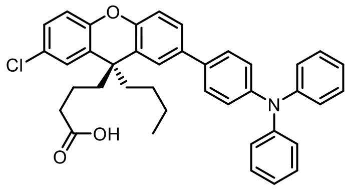

White solid, 95 mg, 63% yield, $[\alpha]_D^{20}$ = +2.25 (*c* 0.400 $CHCl_3$) for 97: 3 er. ^{1}H NMR (500 MHz, $CDCl_3$) δ 7.48 – 7.42 (m, 3H), 7.40 (dd, *J* = 8.4, 2.2 Hz, 1H), 7.31 – 7.22 (m, 6H), 7.14 (td, *J* = 9.9, 8.6, 5.4 Hz, 6H), 7.06 – 6.99 (m, 3H), 6.97 (d, *J* = 8.6 Hz, 1H), 2.16 – 2.06 (m, 3H), 1.98 – 1.81 (m, 3H), 1.25 – 1.09 (m, 4H), 0.90 – 0.84 (m, 2H), 0.71 (t, *J* = 7.3 Hz, 3H). ^{13}C NMR (126 MHz, $CDCl_3$) δ 178.1, 150.7, 150.5, 147.8, 147.1, 136.2, 134.9, 129.4, 128.1, 127.9, 127.6, 126.8, 126.3, 126.1, 124.5, 124.3, 124.2, 123.0, 117.9, 116.7, 45.8, 44.0, 42.7, 33.6, 27.0, 23.0, 20.2, 14.0. HRMS (m/z, ESI): Calcd. for Chemical Formula $C_{39}H_{35}ClNO_3^-$ [M-H]$^-$: 600.2311, Found: 600.2312. HPLC analysis of the reaction product: Daicel Chiralpak IA, hexane/*iso*-propanol = 97: 3, 1.0 mL/min, λ= 321 nm, retention time: 5.83 min (major) and 6.30 min (minor).

(*R*)-4-(9-butyl-2-chloro-7-(4-nitrophenyl)-9H-xanthen-9-yl)butanoic acid (37)

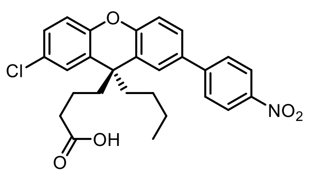

Yellow solid, 84 mg, 70% yield, $[\alpha]_D^{20}$ = -7.00 (*c* 0.100 $CHCl_3$) for 96: 4 er. ^{1}H NMR (500 MHz, $CDCl_3$) δ 8.24 (d, *J* = 8.5 Hz, 2H), 7.68 (d, *J* = 8.7 Hz, 2H), 7.53 (d, *J* = 2.2 Hz, 1H), 7.44 (dd, *J* = 8.5, 2.1 Hz, 1H), 7.28 – 7.25 (m, 1H), 7.18 (dd, *J* = 8.7, 2.4 Hz, 1H), 7.11 (d, *J* = 8.4 Hz, 1H), 7.00 (d, *J* = 8.7 Hz, 1H), 2.32 – 2.13 (m, 3H), 2.05 – 1.96 (m, 1H), 1.91 – 1.89 (m, 2H), 1.33 (d,

J = 14.2 Hz, 2H), 1.13 (dd, *J* = 7.3, 3.1 Hz, 2H), 0.88 – 0.84 (m, 2H), 0.71 (t, *J* = 7.3 Hz, 3H). ^{13}C NMR (126 MHz, $CDCl_3$) δ 179.2, 152.3, 150.2, 147.2, 146.9, 134.1, 128.6, 128.1, 127.5, 127.0, 126.6, 126.1, 125.6, 125.2, 124.3, 118.0, 117.2, 46.2, 43.6, 42.8, 33.6, 26.9, 22.9, 20.1, 13.9.HRMS (m/z, ESI): Calcd. for Chemical Formula $C_{27}H_{25}ClNO_5^-$ [M-H]$^-$: 478.1427, Found: 478.1432. HPLC analysis of the reaction product: Daicel Chiralpak IA, hexane/*iso*-propanol = 95: 5, 1.0 mL/min, λ= 332 nm, retention time: 8.38 min (major) and 16.58 min (minor).

(*R*)-5-(9-butyl-2-chloro-7-(4-nitrophenyl)-9H-xanthen-9-yl)pentanoic acid (38)

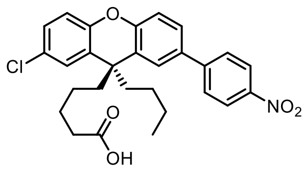

Yellow solid, 62 mg, 50% yield, [α]$_D^{20}$ = -1.00 (*c* 0.100 $CHCl_3$) for 95: 5 er. ^{1}H NMR (500 MHz, $CDCl_3$) δ 8.30 (d, *J* = 8.7 Hz, 2H), 7.70 (d, *J* = 8.7 Hz, 2H), 7.51 – 7.45 (m, 2H), 7.25 (s, 1H), 7.18 (dd, *J* = 8.6, 2.4 Hz, 1H), 7.11 (d, *J* = 8.4 Hz, 1H), 6.99 (d, *J* = 8.6 Hz, 1H), 2.16 (td, *J* = 7.6, 5.1 Hz, 2H), 2.04 – 1.93 (m, 4H), 1.51 – 1.47 (m, 2H), 1.18 – 1.13 (m, 2H), 0.98 – 0.92 (m, 2H), 0.88 – 0.85 (m, 2H), 0.73 (t, *J* = 7.3 Hz, 3H).^{13}C NMR (126 MHz, $CDCl_3$) δ 176.6, 152.3, 150.1, 147.3, 147.0, 134.2, 130.9, 128.6, 128.0, 127.5, 127.0, 126.9, 126.7, 126.1, 125.6, 125.3, 124.4, 117.9, 117.3, 45.5, 45.0, 42.8, 33.2, 27.1, 24.9, 24.5, 23.0, 14.0. HRMS (m/z, ESI): Calcd. for Chemical Formula $C_{28}H_{27}ClNO_5^-$ [M-H]$^-$: 492.1583, Found: 492.1587. HPLC analysis of the reaction product: Daicel Chiralpak IA, hexane/*iso*-propanol = 99: 1, 1.0 mL/min, λ= 331 nm, retention time: 45.37 min (major) and 49.80 min (minor).

(*R*)-4-(9-butyl-2-chloro-7-(4-cyanophenyl)-9H-xanthen-9-yl)butanoic acid (39)

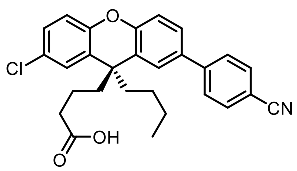

White solid, 70 mg, 61% yield, [α]$_D^{20}$ = -5.00 (*c* 0.200 $CHCl_3$) for 97: 3 er. ^{1}H NMR (500 MHz, $CDCl_3$) δ 7.72 – 7.68 (m, 2H), 7.67 – 7.63 (m, 2H), 7.49 (d, *J* = 2.2 Hz, 1H), 7.44 (dd, *J* = 8.5, 2.2 Hz, 1H), 7.27 (s, 1H), 7.18 (dd, *J* = 8.6, 2.4 Hz, 1H), 7.11 (d, *J* = 8.5 Hz, 1H), 6.99 (d, *J* = 8.7 Hz, 1H), 2.27 – 2.21 (m, 2H), 2.18 – 2.10 (m, 1H), 2.02 – 1.85 (m, 3H), 1.30 – 1.20 (m, 2H), 1.15 – 1.09 (m, 2H), 0.89 – 0.81 (m, 2H), 0.71 (t, *J* = 7.3 Hz, 3H). ^{13}C NMR (126 MHz, $CDCl_3$) δ 178.9, 152.0, 150.2, 145.3, 134.6, 132.8, 128.5, 128.0, 127.5, 126.8, 126.6, 126.1, 125.3, 125.1, 119.2, 117.9, 117.2, 110.6, 46.0, 43.8, 42.7, 33.6, 26.9, 22.9, 20.1, 13.9. HRMS (m/z, ESI): Calcd. for Chemical Formula $C_{28}H_{25}ClNO_3^-$ [M-H]$^-$: 458.1528, Found: 458.1531. HPLC analysis of the reaction product: Daicel Chiralpak IA, hexane/*iso*-propanol = 95: 5, 1.0 mL/min, λ= 319 nm, retention time: 8.05 min (major) and 10.89 min (minor).

(*R*)-4-(9-butyl-2-chloro-7-(6-methoxypyridin-3-yl)-9H-xanthen-9-yl)butanoic acid (40)

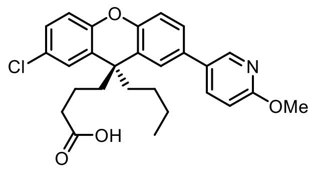

White solid, 63 mg, 54% yield, [α] $_D^{20}$ = -13.00 (*c* 0.100 $CHCl_3$) for 95: 5 er. ^1H NMR (500 MHz, $CDCl_3$) δ 8.37 (d, *J* = 2.5 Hz, 1H), 7.77 (dd, *J* = 8.6, 2.5 Hz, 1H), 7.39 (d, *J* = 2.2 Hz, 1H), 7.34 (dd, *J* = 8.5, 2.2 Hz, 1H), 7.25 (d, *J* = 2.4 Hz, 1H), 7.16 (dd, *J* = 8.7, 2.5 Hz, 1H), 7.07 (d, *J* = 8.4 Hz, 1H), 6.97 (d, *J* = 8.6 Hz, 1H), 6.80 (d, *J* = 8.5 Hz, 1H), 3.97 (s, 3H), 2.25 – 2.16 (m, 2H), 2.12 – 2.04 (m, 1H), 2.01 – 1.83 (m, 3H), 1.30 – 1.21 (m, 2H), 1.15 – 1.10 (m, 2H), 0.86 (q, *J* = 8.4, 8.0 Hz, 2H), 0.71 (t, *J* = 7.3 Hz, 3H). ^{13}C NMR (101 MHz, $CDCl_3$) δ 178.2, 163.5, 151.1, 150.4, 144.7, 137.7, 133.3, 130.1, 128.3, 127.9, 126.8, 126.3, 126.1, 124.9, 124.6, 117.9, 117.0, 110.9, 53.9, 45.7, 44.0, 42.7, 33.8, 27.0, 23.0, 20.3, 14.0. HRMS (m/z, ESI): Calcd. for Chemical Formula $C_{27}H_{27}ClNO_4^-$ $[M-H]^-$: 464.1634, Found: 464.1633. HPLC analysis of the reaction product: Daicel Chiralpak IA, hexane/*iso*-propanol = 99: 1, 1.0 mL/min, λ= 271 nm, retention time: 22.07 min (major) and 24.07 min (minor).

(*R*)-5-(9-butyl-2-chloro-7-(6-methoxypyridin-3-yl)-9H-xanthen-9-yl)pentanoic acid (41)

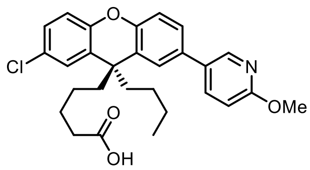

White solid, 70 mg, 58% yield, [α] $_D^{20}$ = -7.00 (*c* 0.100 $CHCl_3$) for 94.5: 5.5 er. ^1H NMR (400 MHz, $CDCl_3$) δ 8.36 (d, *J* = 2.5 Hz, 1H), 7.76 (dd, *J* = 8.6, 2.5 Hz, 1H), 7.37 (d, *J* = 2.2 Hz, 1H), 7.34 (dd, *J* = 8.4, 2.2 Hz, 1H), 7.24 (d, *J* = 2.5 Hz, 1H), 7.16 (dd, *J* = 8.6, 2.5 Hz, 1H), 7.06 (d, *J* = 8.4 Hz, 1H), 6.96 (d, *J* = 8.7 Hz, 1H), 6.83 (d, *J* = 8.6 Hz, 1H), 3.98 (s, 3H), 2.15 (dd, *J* = 8.4, 6.8 Hz, 2H), 2.03 – 1.85 (m, 4H), 1.50 – 1.42 (m, 2H), 1.17 – 1.11 (m, 2H), 0.96 – 0.90 (m, 2H), 0.88 – 0.83 (m, 2H), 0.72 (t, *J* = 7.3 Hz, 3H). ^{13}C NMR (126 MHz, $CDCl_3$) δ 178.3, 163.5, 151.1, 150.4, 144.6, 137.7, 133.2, 130.1, 128.2, 127.8, 127.1, 126.3, 126.1, 125.2, 124.4, 117.8, 116.9, 111.0, 53.9, 45.4, 44.9, 42.8, 33.6, 27.1, 24.9, 24.4, 23.0, 14.0. HRMS (m/z, ESI): Calcd. for Chemical Formula $C_{28}H_{29}ClNO_4^-$ $[M-H]^-$: 478.1791, Found: 478.1800. HPLC analysis of the reaction product: Daicel Chiralpak IA, hexane/*iso*-propanol = 99: 1, 1.0 mL/min, λ= 272 nm, retention time:23.35 min (major) and 20.51 min (minor).

(*R*)-4-(9-butyl-2-chloro-7-(thiophen-3-yl)-9H-xanthen-9-yl)butanoic acid (42)

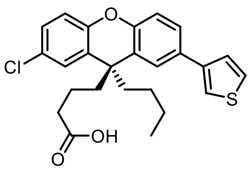

White solid, 60 mg, 55% yield, $[\alpha]_D^{20}$ = -0.40 (*c* 1.000 $CHCl_3$) for 95: 5 er. ^{1}H NMR (500 MHz, $CDCl_3$) δ 7.48 (d, *J* = 2.1 Hz, 1H), 7.43 (dd, *J* = 8.4, 2.1 Hz, 1H), 7.38 (t, *J* = 2.1 Hz, 1H), 7.35 (d, *J* = 2.2 Hz, 2H), 7.25 (d, *J* = 2.4 Hz, 1H), 7.16 (dd, *J* = 8.7, 2.4 Hz, 1H), 7.03 (d, *J* = 8.5 Hz, 1H), 6.97 (d, *J* = 8.6 Hz, 1H), 2.20 (td, *J* = 7.2, 4.7 Hz, 2H), 2.14 – 2.05 (m, 1H), 1.97 -1.93 (m, 2H), 1.89 – 1.82 (m, 1H), 1.31 – 1.20 (m, 2H), 1.14 – 1.11 (m, 2H), 0.90 – 0.82 (m, 2H), 0.71 (t, *J* = 7.3 Hz, 3H). ^{13}C NMR (126 MHz, $CDCl_3$) δ 179.0, 150.8, 150.4, 142.1, 131.6, 128.2, 128.1, 127.9, 126.8, 126.4, 126.4, 126.2, 126.1, 124.5, 124.2, 119.7, 117.9, 116.7, 45.9, 43.9, 42.7, 33.8, 27.0, 23.0, 20.2, 14.0. HRMS (m/z, ESI): Calcd. for Chemical Formula $C_{25}H_{24}ClO_3S^-$ $[M-H]^-$: 439.1140, Found: 439.1148. HPLC analysis of the reaction product: Daicel Chiralpak IA, hexane/*iso*-propanol = 99: 1, 1.0 mL/min, λ= 272 nm, retention time: 16.93 min (major) and 18.82 min (minor).

(*R*)-3-(9-butyl-2-chloro-7-(dibenzo[b,d]thiophen-4-yl)-9H-xanthen-9-yl)propanoic acid (43)

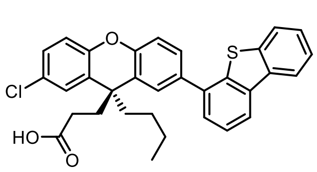

White solid, 70 mg, 53% yield, $[\alpha]_D^{20}$ = +85.00 (*c* 0.040 $CHCl_3$) for 96: 4 er. ^{1}H NMR (500 MHz, $CDCl_3$) δ 8.18 – 8.13 (m, 2H), 7.81 – 7.77 (m, 1H), 7.75 (d, *J* = 2.0 Hz, 1H), 7.57 – 7.50 (m, 2H), 7.49 – 7.41 (m, 3H), 7.29 (d, *J* = 2.3 Hz, 1H), 7.20 (dd, *J* = 8.7, 2.4 Hz, 1H), 7.16 (dd, *J* = 8.4, 1.6 Hz, 1H), 7.02 (dd, *J* = 8.7, 1.6 Hz, 1H), 2.39 – 2.24 (m, 2H), 2.10 – 1.95 (m, 4H), 1.21 (dd, *J* = 8.4, 6.4 Hz, 2H), 1.04 – 0.92 (m, 2H), 0.78 (t, *J* = 7.4 Hz, 3H). ^{13}C NMR (126 MHz, $CDCl_3$) δ 178.5, 151.2, 150.4, 139.6, 138.8, 136.5, 136.2, 135.9, 128.6, 128.4, 128.3, 127.0, 126.6, 126.1_1, 126.1_2, 126.0, 125.3, 124.6, 123.7, 122.8, 121.9, 120.6, 118.1, 117.1, 45.2, 42.2, 39.8, 30.2, 27.2, 23.1, 14.1. HRMS (m/z, ESI): Calcd. for Chemical Formula $C_{32}H_{26}ClO_3S^-$ $[M-H]^-$:525.1297, Found: 525.1290. HPLC analysis of the reaction product: Daicel Chiralpak IA, hexane/*iso*-propanol = 99: 1, 1.0 mL/min, λ= 332 nm, retention time: 27.84 min (major) and 36.43 min (minor).

(*R*)-4-(9-butyl-2-chloro-7-(dibenzo[b,d]thiophen-4-yl)-9H-xanthen-9-yl)butanoic acid (44)

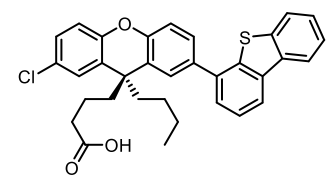

White solid, 95 mg, 70% yield, $[\alpha]_D^{20}$ = -14.00 (*c* 0.100 $CHCl_3$) for 97.5: 2.5 er. ^1H NMR (500 MHz, $CDCl_3$) δ 8.20 – 8.16 (m, 1H), 8.14 (dd, *J* = 7.8, 1.2 Hz, 1H), 7.84 – 7.80 (m, 1H), 7.73 (d, *J* = 2.2 Hz, 1H), 7.55 (t, *J* = 7.6 Hz, 1H), 7.53 – 7.48 (m, 2H), 7.47 – 7.41 (m, 2H), 7.27 (d, *J* = 2.5 Hz, 1H), 7.18 (dd, *J* = 8.7, 2.5 Hz, 1H), 7.15 (d, *J* = 8.4 Hz, 1H), 7.01 (d, *J* = 8.7 Hz, 1H), 2.22 (t, *J* = 7.4 Hz, 2H), 2.08 – 1.95 (m, 3H), 1.92 – 1.86 (m, 1H), 1.39 – 1.27 (m, 2H), 1.19 (p, *J* = 7.4 Hz, 2H), 1.02 – 0.96 (m, 1H), 0.93 – 0.86 (m, 1H), 0.77 (t, *J* = 7.4 Hz, 3H). ^{13}C NMR (126 MHz, $CDCl_3$) δ 178.1, 151.3, 150.4, 139.6, 138.7, 136.7, 136.5, 136.0, 135.9, 128.3, 128.1, 127.9, 127.0, 126.9, 126.7, 126.2, 126.1, 125.3, 124.6, 124.5, 122.8, 121.9, 120.5, 117.9, 116.9, 45.6, 44.4, 42.8, 33.9, 27.1, 23.1, 20.4, 14.1. HRMS (m/z, ESI): Calcd. for Chemical Formula $C_{33}H_{28}ClO_3S^-$[M-H]$^-$:539.1453, Found: 539.1443.

HPLC analysis of the reaction product: Daicel Chiralpak IA, hexane/*iso*-propanol = 95: 5, 1.0 mL/min, λ= 332 nm, retention time: 5.63 min (major) and 6.22 min (minor).

(*R*)-5-(9-butyl-2-chloro-7-(dibenzo[b,d]thiophen-4-yl)-9H-xanthen-9-yl)pentanoic acid (45)

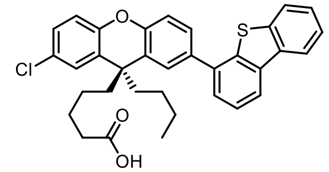

White solid, 93 mg, 67% yield, $[\alpha]_D^{20}$ = -16.00 (*c* 0.100 $CHCl_3$) for 95.5: 4.5 er. ^1H NMR (400 MHz, $CDCl_3$) δ 8.20 – 8.13 (m, 2H), 7.85 – 7.80 (m, 1H), 7.74 (d, *J* = 2.2 Hz, 1H), 7.57 (t, *J* = 7.6 Hz, 1H), 7.51 – 7.44 (m, 4H), 7.27 (d, *J* = 2.5 Hz, 1H), 7.20 – 7.17 (m, 1H), 7.14 (d, *J* = 8.4 Hz, 1H), 7.00 (d, *J* = 8.7 Hz, 1H), 2.23 – 2.16 (m, 2H), 2.03 – 1.86 (m, 4H), 1.57 – 1.49 (m, 2H), 1.21 (d, *J* = 7.4 Hz, 2H), 1.03 – 0.85 (m, 4H), 0.78 (t, *J* = 7.3 Hz, 3H). ^{13}C NMR (126 MHz, $CDCl_3$) δ 178.9, 151.2, 150.4, 139.6, 138.8, 136.7, 136.5, 136.0, 135.9, 128.3, 128.0, 127.8, 127.2, 127.0, 126.6, 126.1_1, 126.1_2, 125.3, 124.8, 124.6, 122.8, 121.9, 120.5, 117.8, 116.9, 45.4, 45.2, 42.8, 33.8, 27.2, 25.1, 24.6, 23.1, 14.1. HRMS (m/z, ESI): Calcd. for Chemical Formula $C_{34}H_{30}ClO_3S^-$ [M-H]$^-$:553.1610, Found: 553.1599. HPLC analysis of the reaction product: Daicel Chiralpak IA, hexane/*iso*-propanol = 99: 1, 1.0 mL/min, λ= 332 nm, retention time: 21.52 min (major) and 20.12 min (minor).

(*R*)-4-(9-butyl-2-chloro-7-(thianthren-1-yl)-9H-xanthen-9-yl)butanoic acid (46)

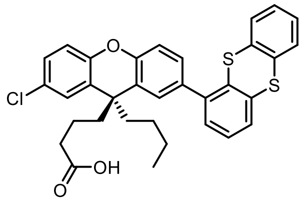

White solid, 76 mg, 53% yield, $[\alpha]_D^{20}$ = +23.00 (*c* 0.100 $CHCl_3$) for 97: 3 er. ^1H NMR (500 MHz, $CDCl_3$) δ 7.51 (dd, *J* = 7.3, 1.8 Hz, 1H), 7.48 (dd, *J* = 7.8, 1.4 Hz, 1H), 7.31 (dd, *J* = 6.9, 2.0 Hz, 1H), 7.29 – 7.25 (m, 4H), 7.23 – 7.17 (m, 3H), 7.13 (td, *J* = 7.5, 1.4 Hz, 1H), 7.10 (d, *J* = 8.3 Hz, 1H), 7.01 (d, *J* = 8.7 Hz, 1H), 2.21 (t, *J* = 7.5 Hz, 2H), 2.09 – 1.85 (m, 4H), 1.43 – 1.33 (m, 1H), 1.29 (d, *J* = 4.7 Hz, 1H), 1.18 (p, *J* = 7.3 Hz, 2H), 1.03 – 0.98 (m, 1H), 0.93 – 0.87 (m, 1H), 0.76 (t, *J* = 7.3 Hz, 3H). ^{13}C NMR (126 MHz, $CDCl_3$) δ 178.9, 151.1, 150.4, 142.1, 136.5, 136.0, 135.4, 135.3, 129.2, 128.8, 128.7, 128.4, 128.3, 127.9, 127.7, 127.2, 126.8, 126.2, 123.7, 117.9, 116.3, 45.7, 44.4, 42.6, 34.0, 27.1, 23.0, 20.4, 14.1. HRMS (m/z, ESI): Calcd. for Chemical Formula $C_{33}H_{28}ClO_3S^-$ $[M-H]^-$: 571.1174, Found: 571.1166. HPLC analysis of the reaction product: Daicel Chiralpak IA, hexane/*iso*-propanol = 99: 1, 1.0 mL/min, λ= 262 nm, retention time: 18.98 min (major) and 21.52 min (minor).

Ethyl (*R*)-2'-(9-butyl-7-(2-chlorophenyl)-9-(2-ethoxy-2-oxoethyl)-9H-fluoren-2-yl)-[1,1'-biphenyl]-4-carboxylate (47)

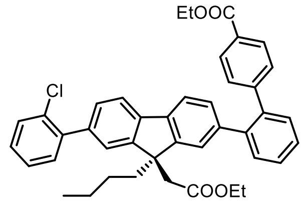

The reaction mixture was neutralized with 1 M HCl to pH 2-3 and the crude product was dissolved in EtOH, then 10μL conc. H_2SO_4 was added and the reaction mixture was stirred at 65 °C for 10 h. The esterification product was obtained as a white solid, 84.6 mg, 53% yield, $[\alpha]_D^{20}$ = -12.20 (*c* 0.100 $CHCl_3$) for 85.5:14.5 er. ^1H NMR (500 MHz, $CDCl_3$) δ 7.90 (d, *J* = 8.5 Hz, 2H), 7.75 – 7.71 (m, 1H), 7.65 (d, *J* = 7.8 Hz, 1H), 7.58 – 7.54 (m, 1H), 7.48 (ddd, *J* = 6.8, 4.9, 1.2 Hz, 4H), 7.42 (dt, *J* = 7.5, 1.6 Hz, 2H), 7.35 (td, *J* = 7.4, 1.4 Hz, 1H), 7.31 – 7.29 (m, 2H), 7.25 (dd, *J* = 7.8, 1.6 Hz, 1H), 7.13 (d, *J* = 1.1 Hz, 1H), 4.36 – 4.31 (m, 2H), 3.77 (q, *J* = 7.1 Hz, 2H), 2.77 (br, 2H), 2.02 – 1.94 (m, 1H), 1.80-1.76 (m, 1H), 1.36 (t, *J* = 7.1 Hz, 3H), 1.02-0.98 (m, 2H), 0.85 (t, *J* = 7.1 Hz, 3H), 0.69 (t, *J* = 7.1 Hz, 3H), 0.59-0.54 (m, 2H). ^{13}C NMR (126 MHz, $CDCl_3$) δ 170.2, 166.4, 149.4, 149.0, 146.5, 141.0, 140.8, 140.2, 139.9, 139.6, 139.0, 138.1, 131.4, 130.8, 130.5, 130.0, 129.9, 129.2, 129.0, 128.7, 128.5, 128.4, 128.1, 127.6, 126.8, 125.1, 124.7, 119.7, 119.4, 60.9, 59.9, 52.5, 44.0, 38.9, 25.8, 22.7, 14.3, 13.8_1, 13.8_2. MS (m/z, ESI): Calcd. for Chemical Formula: $C_{42}H_{39}ClNaO_4^+$ $[M+Na]^+$: 665.2429, Found: 665.2423. HPLC analysis of the reaction product: Daicel Chiralpak IA, hexane/*iso*-propanol = 95: 5, 1.0 mL/min, λ= 330 nm, retention time: 8.06 min (major) and 8.89 min (minor).

Ethyl (*R*)-2'-(9-butyl-7-(2-chloro-4-methoxyphenyl)-9-(2-ethoxy-2-oxoethyl)-9H-fluoren-2-yl)-5'-methoxy-[1,1'-biphenyl]-4-carboxylate (48)

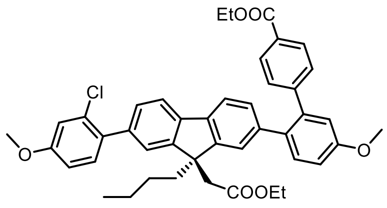

After the reaction was completed, the reaction mixture was neutralized with 1 M HCl to pH 2-3 and the crude product was dissolved in EtOH, then 10μL conc. H_2SO_4 was added and the reaction mixture was stirred at 65 °C for 10 h. The esterification product was obtained as a white solid, 97.9 mg, 56% yield, $[\alpha]_D^{20}$ = -35.10 (*c* 0.100 $CHCl_3$) for 89.5:10.5 er. ^{1}H NMR (500 MHz, $CDCl_3$) δ 7.93 – 7.86 (m, 2H), 7.69 (d, *J* = 7.8 Hz, 1H), 7.61 (d, *J* = 7.8 Hz, 1H), 7.48 (d, *J* = 8.4 Hz, 1H), 7.44 (d, *J* = 1.1 Hz, 1H), 7.38 (dd, *J* = 7.8, 1.5 Hz, 1H), 7.32 (d, *J* = 8.5 Hz, 1H), 7.31 – 7.28 (m, 2H), 7.20 (dd, *J* = 7.8, 1.5 Hz, 1H), 7.08 – 7.02 (m, 3H), 7.00 (d, *J* = 2.7 Hz, 1H), 6.91 (dd, *J* = 8.5, 2.6 Hz, 1H), 4.37 – 4.30 (m, 2H), 3.92 (s, 3H), 3.87 (s, 3H), 3.77 (q, *J* = 7.1 Hz, 2H), 2.74 (br, 2H), 1.99 – 1.93 (m, 1H), 1.79 – 1.73 (m, 1H), 1.35 (d, *J* = 7.1 Hz, 3H), 1.00 (dd, *J* = 13.9, 7.0 Hz, 2H), 0.85 (t, *J* = 7.1 Hz, 3H), 0.69 (t, *J* = 7.3 Hz, 3H), 0.55 (dt, *J* = 19.8, 7.3 Hz, 2H). ^{13}C NMR (126 MHz, $CDCl_3$) δ 170.2, 166.4, 159.3, 159.0, 149.3, 148.9, 146.5, 140.8, 139.8, 139.6, 138.7, 137.8, 133.7, 133.3, 133.1, 131.9_1, 131.9_2, 129.9, 129.2, 129.0, 128.7, 128.6, 125.2, 124.8, 119.6, 119.3, 115.9, 115.2, 113.6, 113.1, 60.9, 59.8, 55.6, 55.5, 52.4, 44.1, 38.9, 25.8, 22.7, 14.3, 13.8_1, 13.8_2. MS (m/z, ESI): Calcd. for Chemical Formula: $C_{44}H_{43}ClNaO_6^+$ $[M+Na]^+$: 725.2640, Found: 725.2638. HPLC analysis of the reaction product: Daicel Chiralpak IA, hexane/*iso*-propanol = 95: 5, 1.0 mL/min, λ= 330 nm, retention time: 13.94 min (minor) and 15.36 min (major).

(*R*)-2-((2-(4-(tert-butyl)phenyl)-7-chloro-9-methyl-9H-fluoren-9-yl)oxy)acetic acid (49)

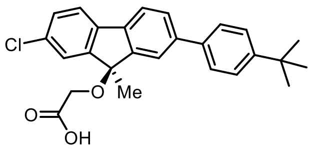

White solid, 74 mg, 70% yield, $[\alpha]_D^{20}$ = -18.33 (*c* 0.06 $CHCl_3$) for 98.5: 1.5 er. ^{1}H NMR (400 MHz, $CDCl_3$) δ 7.71 – 7.63 (m, 3H), 7.60 – 7.54 (m, 3H), 7.51 – 7.47 (m, 3H), 7.39 (dd, *J* = 8.1, 2.0 Hz, 1H), 3.50 (s, 2H), 1.82 (s, 3H), 1.37 (s, 9H). ^{13}C NMR (126 MHz, $CDCl_3$) δ 170.6, 151.1, 147.1, 145.4, 141.9, 138.4, 137.8, 137.7, 134.2, 130.2, 128.9, 126.9, 126.1, 124.4, 122.4, 121.5, 120.8, 85.5, 61.8, 31.5, 26.2. HRMS (m/z, ESI): Calcd. for Chemical Formula $C_{26}H_{24}ClO_3^-$ $[M-H]^-$: 419.1419, Found: 419.1413. HPLC analysis of the reaction product: Daicel Chiralpak IA, hexane/*iso*-propanol = 97: 3, 1.0 mL/min, λ= 304 nm, retention time: 15.88 min (major) and 13.73 min (minor).

(*R*)-2-((9-butyl-2-(4-(tert-butyl)phenyl)-7-chloro-9H-fluoren-9-yl)oxy)acetic acid (50)

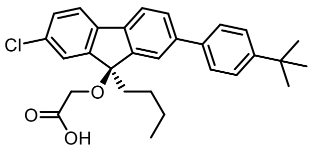

White solid, 72 mg, 63% yield, $[\alpha]_D^{20}$ = + 55.00 (*c* 0.1 $CHCl_3$) for 99: 1 er. ^1H NMR (500 MHz, $CDCl_3$) δ 7.66 (s, 3H), 7.60 – 7.55 (m, 3H), 7.51 – 7.48 (m, 2H), 7.44 (dd, *J* = 1.9, 0.5 Hz, 1H), 7.39 (dd, *J* = 8.1, 2.0 Hz, 1H), 3.52 (s, 2H), 2.28 – 2.20 (m, 2H), 1.37 (s, 9H), 1.21 – 1.14 (m, 2H), 0.89 – 0.84 (m, 2H), 0.74 (t, *J* = 7.4 Hz, 3H). ^{13}C NMR (126 MHz, $CDCl_3$) δ 171.8, 151.1, 146.2, 144.5, 141.6, 139.2, 138.7, 137.7, 134.1, 130.1, 128.7, 126.9, 126.1, 124.5, 122.6, 121.3, 120.7, 88.8, 61.6, 39.2, 34.7, 31.5, 25.7, 22.9, 13.9. HRMS (m/z, ESI): Calcd. for Chemical Formula $C_{29}H_{30}ClO_3^-$ [M-H]$^-$: 461.1889, Found: 461.1888. HPLC analysis of the reaction product: Daicel Chiralpak IA, hexane/*iso*-propanol = 95: 5, 1.0 mL/min, λ= 304 nm, retention time: 9.15 min (major) and 10.18 min (minor).

Control experiments

1. The role of K^+

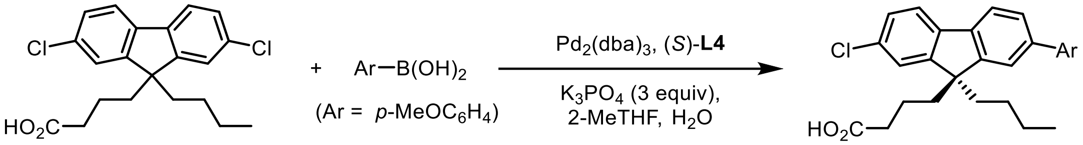

condition:
1. no additive : 94% conv., 96:4 er
2. 1.0 equiv 18-crown-6: 89% conv., 81:19 er
3. 3.0 equiv 18-crown-6: 88% conv., 78:22 er
4. 9.0 equiv 18-crown-6: 84% conv., 61:39 er

Procedure: To the mixture of 4-(9-butyl-2,7-dichloro-9H-fluoren-9-yl)butanoic acid (0.1 mmol), K_3PO_4 (3.0 equiv) and 18-crown-6 were added 1.5 mL 2-MeTHF and the resulting reaction mixture was stirring for 20 min, followed by addition of *p*-MeOPhB$(OH)_2$ (1.2 equiv), (*S*)-**L4** and Pd_2dba_3 complex solution (1.0 mol%), and water following the general procedure. Conversion was determined by analysis of the ^{1}H NMR spectrum of crude reaction mixture.

2. The roles of the phosphonate group of ligand and the carboxylate group of the substrate.

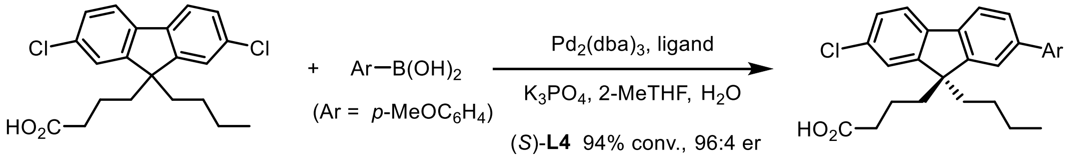

(*S*)-**L4** 94% conv., 96:4 er
(*R*)-**L1** 87% conv., 56:44 er
(*S*)-**L3**: 90% conv., 83:17 er

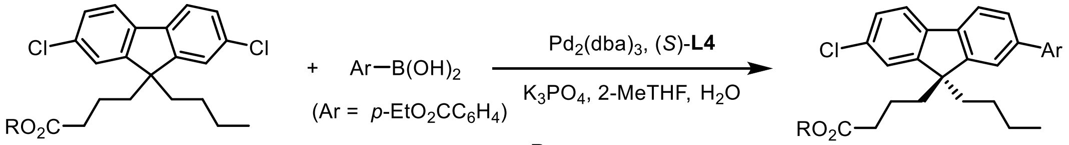

R =
H: 67% yield, 96:4 er
K: 61% yield, 96:4 er
Et: 42% yield, 50:50 er

Procedure: Potassium salt of 4-(9-butyl-2,7-dichloro-9H-fluoren-9-yl)butanoic acid was prepared by stirring of 4-(9-butyl-2,7-dichloro-9H-fluoren-9-yl)butanoic acid (151 mg, 0.4 mmol) and *t*BuOK (45.2 mg, 0.4 mmol) in 2.0 mL THF at 60 ^{o}C for 1h, followed by removal of the solvent under reduced pressure to give the desired product as a white solid.

The reactions were carried out on 0.1 mmol scale following the general procedure. Conversions were determined by analysis of ^{1}H NMR spectra of crude reaction mixtures. Yields were calculated based on the weights of products isolated using column chromatography.

3. Competition experiments

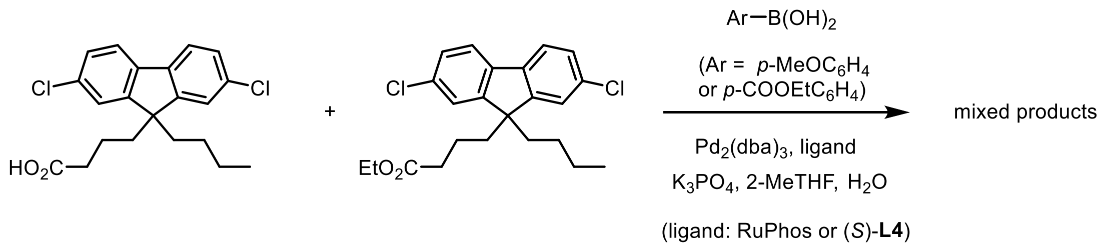

Procedure: To a mixture of 4-(9-butyl-2,7-dichloro-9H-fluoren-9-yl)butanoic acid (37.3 mg, 0.1 mmol), ethyl 4-(9-butyl-2,7-dichloro-9H-fluoren-9-yl)butanoate (40.5 mg, 0.1 mmol), and K_3PO_4 (63.8 mg, 0.3 mmol) were added 3 mL 2-MeTHF and 50 μL H_2O. The resulting reaction mixture was stirring for 20 min, followed by addition of *p*-$MeOC_6H_4B(OH)_2$ (15.2 mg, 0.1 mmol) or *p*-$COOEtC_6H_4B(OH)_2$ (19.4 mg, 0.1 mmol) , then 1.0 mL 2-MeTHF solution of (*S*)-**L4** (1.3 mg, 0.022 mmol) or RuPhos (1.03 mg, 0.0022 mmol) and Pd_2dba_3 (0.9 mg, 0.001 mmol) complex was added. The resulting reaction mixture was stirred at 60 ^{o}C for 20 h. Conversion of each substrate was determined by analysis of the ^{1}H NMR spectra of the resulting mixtures.

Characterization data of the coupling product of ethyl ester **53** under the optimized reaction conditions.

Ethyl (R)-4-(9-butyl-7-chloro-9-(4-ethoxy-4-oxobutyl)-9H-fluoren-2-yl)benzoate (coupling product of **53)**

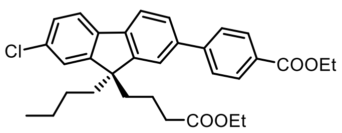

White solid, 21.7 mg, 42% yield. ^{1}H NMR (500 MHz, $CDCl_3$) δ 8.18 – 8.13 (m, 2H), 7.77 – 7.73 (m, 3H), 7.65 (ddd, *J* = 14.5, 7.5, 1.7 Hz, 2H), 7.59 (d, *J* = 1.2 Hz, 1H), 7.36 (dd, *J* = 7.1, 1.8 Hz, 2H), 4.44 (q, *J* = 7.1 Hz, 2H), 4.04 (q, *J* = 7.1, 2H), 2.13 – 1.96 (m, 6H), 1.45 (t, *J* = 7.1 Hz, 3H), 1.18 (t, *J* = 7.1 Hz, 3H), 1.14-1.12 (m, 2H), 1.05 – 0.98 (m, 2H), 0.71 (t, *J* = 7.4 Hz, 3H), 0.69 – 0.62 (m, 2H). ^{13}C NMR (126 MHz, $CDCl_3$) δ 173.2, 166.6, 152.3, 150.7, 145.7, 140.2, 139.3, 139.1, 133.3, 130.1, 129.2, 127.5, 127.1, 126.6, 123.3, 121.6, 121.0, 120.3, 61.0, 60.2, 55.3, 40.2, 39.5, 34.3, 25.8, 22.9, 19.4, 14.4, 14.2, 13.8. MS (m/z, ESI): Calcd. for Chemical Formula: Chemical Formula: $C_{32}H_{35}ClO_4^+$ $[M+H]^+$: 519.2302, Found: 519.2304.

HPLC analysis of the product: Daicel Chiralpak IA, hexane/*iso*-propanol = 99: 1, 1.0 mL/min, λ= 330 nm, retention time: 7.16 min and 7.54 min. 50:50 er.

Synthetic applications

(*S*)-4-(9-butyl-2-(4-(ethoxycarbonyl)phenyl)-7-(4,4,5,5-tetramethyl-1,3,2-dioxaborolan-2-yl)-9H-fluoren-9-yl)butanoic acid (54)

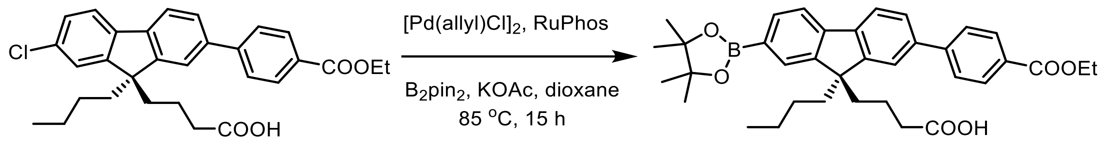

Under N_2 atmosphere, to a mixture of $[Pd(allyl)Cl]_2$ (3.6 mg, 0.01 mmol) and RuPhos (10.3 mg, 0.022 mmol) was added 0.5 mL dioxane and the resulting solution was stirred for 20 min before use. To a mixture of (*R*)-4-(9-butyl-2-chloro-7-(4-(ethoxycarbonyl)phenyl)-9H-fluoren-9-yl)butanoic acid **3** (49.1 mg, 0.1 mmol), B_2pin_2 (38.0 mg, 0.15 mmol) and KOAc (19.6 mg, 0.2 mmol) was added 1.0 mL dioxane and followed with 0.1 mL metal-ligand complex solution.[13] The resulting reaction mixture was stirred at 85 °C for 15 h. After the reaction was completed, the reaction was quenched with water, neutralized to pH 3-5 with 1 M HCl and extracted with ethyl acetate (3 mL X 3) for three times. The combined organic phases were washed with brine, dried over Na_2SO_4 and concentrated in *vacuo*. The crude materials were purified by flash column chromatography with hexane and ethyl acetate (6:1-1:1) to give the desired products as a white solid 39.0 mg in 67% yield. $[\alpha]_D^{20}$ = +9.50 (*c* 0.020 $CHCl_3$) for 97:3 er. ^1H NMR (500 MHz, $CDCl_3$) δ 8.14 (d, *J* = 8.4 Hz, 2H), 7.85 (dd, *J* = 7.5, 0.8 Hz, 1H), 7.81 (d, *J* = 7.9 Hz, 1H), 7.75 (dd, *J* = 16.9, 8.6 Hz, 4H), 7.63 (dd, *J* = 7.9, 1.6 Hz, 1H), 7.59 (d, *J* = 1.2 Hz, 1H), 4.43 (q, *J* = 7.1 Hz, 2H), 2.12 (dt, *J* = 20.0, 7.3 Hz, 4H), 2.07 – 2.03 (m, 2H), 1.44 (t, *J* = 7.1 Hz, 3H), 1.41 (s, 12H), 1.09 (dd, *J* = 14.1, 7.1 Hz, 2H), 0.98 (dd, *J* = 13.1, 7.2 Hz, 2H), 0.70 – 0.59 (m, 5H). ^{13}C NMR (126 MHz, $CDCl_3$) δ 177.4, 166.6, 151.4, 149.5, 145.9, 143.5, 141.1, 139.5, 134.1, 130.1, 129.1, 128.8, 127.1, 126.5, 121.7, 120.7, 119.4, 83.8, 61.0, 55.0, 40.2, 39.4, 33.7, 25.8, 24.96, 24.9, 23.0, 19.2, 14.4, 13.8. HRMS (m/z, ESI): Calcd. for Chemical Formula: $C_{36}H_{44}BO_6^+$ $[M+H]^+$: 583.3014, Found: 583.2954. HPLC analysis of the reaction product: Daicel Chiralpak IH, hexane/*iso*-propanol = 95: 5, 1.0 mL/min, λ = 338 nm, retention time: 26.09 min (major) and 40.06 min (minor).

(*R*)-4-(9-butyl-2-(4-(diphenylamino)phenyl)-7-(4-(ethoxycarbonyl)phenyl)-9H-fluoren-9-yl)butanoic acid (55)

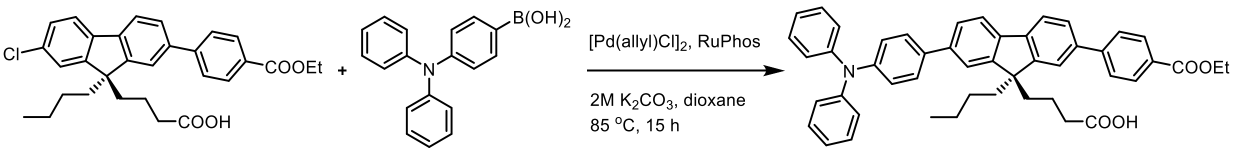

Under N_2 atmosphere, to a mixture of $[Pd(allyl)Cl]_2$ (3.6 mg, 0.01 mmol) and RuPhos (10.3 mg, 0.022 mmol) was added 0.5 mL dioxane and the resulting solution was stirred for 20 min before use. To a mixture of (R)-4-(9-butyl-2-chloro-7-(4-(ethoxycarbonyl)phenyl)-9H-fluoren-9-yl)butanoic acid **3** (49.1 mg, 0.1 mmol), and (4-(diphenylamino)phenyl)boronic acid (31.8 mg, 0.11 mg) was added 1.0 mL dioxane and followed with 0.1 mL metal-ligand complex solution, then 0.15 mL 2M K_2CO_3 (41.4 mg, 0.3 mmol) was added. The resulting reaction mixture was stirred at 85 °C for 15 h. After the reaction was completed, the reaction was quenched with water, neutralized to pH 3-5 with 1 M HCl and extracted with ethyl acetate (3 mL X 3) for three times. The combined organic phases were washed with brine, dried over Na_2SO_4 and concentrated in *vacuo*. The crude materials were purified by flash column chromatography with hexane and ethyl acetate (6:1-1:1) to give the desired products 50.2 mg in 72% yield as a white solid, $[\alpha]_D^{20}$ = +27.00 (*c* 0.020 $CHCl_3$) for 97:3 er. ^1H NMR (500 MHz, $CDCl_3$) δ 8.19 – 8.11 (m, 2H), 7.79 (dd, *J* = 7.7, 6.6 Hz, 2H), 7.76 – 7.71 (m, 2H), 7.63 (dd, *J* = 7.9, 1.6 Hz, 1H), 7.58 (ddd, *J* = 6.6, 5.9, 4.3 Hz, 5H), 7.32 – 7.28 (m, 4H), 7.23 – 7.12 (m, 6H), 7.09 – 7.02 (m, 2H), 4.43 (q, *J* = 7.1 Hz, 2H), 2.17 – 2.09 (m, 4H), 2.08 – 2.03 (m, 2H), 1.44 (t, *J* = 7.1 Hz, 3H), 1.11 (dd, *J* = 14.8, 7.4 Hz, 2H), 1.08 – 1.02 (m, 2H), 0.75 – 0.66 (m, 5H). ^{13}C NMR (126 MHz, $CDCl_3$) δ 166.6, 151.0, 151.0, 147.7, 147.2, 145.9, 141.0, 140.0, 139.3, 138.9, 135.4, 130.1, 129.3, 129.0, 127.8, 127.0, 126.5, 125.9, 124.4, 124.0, 123.0, 121.6, 121.0, 120.3, 120.2, 61.0, 55.0, 40.4, 39.5, 33.7, 25.9, 23.0, 19.3, 14.4, 13.8. HRMS (m/z, ESI): Calcd. for Chemical Formula: $C_{48}H_{46}NO_4^+$ $[M+H]^+$: 700.3785, Found: 700.3738. HPLC analysis of the reaction product: Daicel Chiralpak IH, hexane/*iso*-propanol =94: 6,1.0mL/min, λ = 350 nm, retention time:7.66 min (major) and 32.51 min (minor).

(*R*)-4-(9-butyl-2-(4-(ethoxycarbonyl)phenyl)-7-(methyl(phenyl)amino)-9H-fluoren-9-yl)butanoic acid (56)

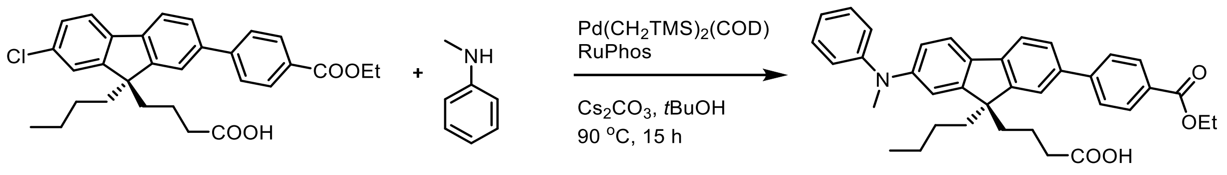

Under N_2 atmosphere, to a mixture of $Pd(CH_2TMS)_2(COD)$ (9.5 mg, 0.025 mmol) and RuPhos (16.5 mg, 0.035 mmol) was added 0.5 mL dioxane and the resulting solution was stirred for 20 min before use. Then 0.1 mL metal-ligand complex solution was added to a 1.0 mL *t*BuOH solution of (R)-4-(9-butyl-2-chloro-7-(4-(ethoxycarbonyl)phenyl)-9H-fluoren-9-yl)butanoic acid **3** (49.1 mg, 0.1 mmol), methylaniline (12.9 mg, 0.12 mmol) and Cs_2CO_3 (48.9 mg, 0.15 mmol).[14] Then the resulting reaction mixture was stirred at 90 °C for 15 h. The reaction was quenched with water, neutralized to pH 1~2 with 1 M HCl and extracted with ethyl acetate (3 mL X 3) for three times. The combined organic phases were washed with brine, dried over Na_2SO_4 and concentrated in *vacuo*. Then the crude product was purified by flash column chromatography with hexane and ethyl acetate (6:1-1:1) to give the desired products 44.1 mg in 79% yield as a light-yellow solid, $[\alpha]_D^{20}$ = +4.00 (*c* 0.030 $CHCl_3$) for 97:3 er. ^{1}H NMR (500 MHz, $CDCl_3$) δ 8.14-8.13 (m, 2H), 7.72 (dt, *J* = 16.8, 5.9 Hz, 3H), 7.65 – 7.58 (m, 2H), 7.55 (s, 1H), 7.34 – 7.28 (m, 2H), 7.16 – 6.86 (m, 5H), 4.43 (q, *J* = 7.1 Hz, 2H), 3.41 (s, 3H), 2.13 – 1.94 (m, 6H), 1.44 (t, *J* = 7.1 Hz, 3H), 1.11-1.06 (m, 4H), 0.74 – 0.67 (m, 5H). ^{13}C NMR (126 MHz, $CDCl_3$) δ 178.1, 166.7, 151.7, 150.5, 150.2, 149.2, 148.8, 146.0, 141.4, 137.8, 134.1, 130.1, 129.2, 128.8, 127.5, 127.2, 127.0, 126.9, 126.5, 122.9, 121.5, 121.4, 121.2, 120.7, 120.3, 120.1, 119.7, 119.4, 115.3, 60.9, 54.9, 40.6, 40.2, 39.5, 33.9, 25.9, 23.0, 19.3, 19.2, 14.4, 13.8. MS (m/z, ESI): Calcd. for Chemical Formula: $C_{37}H_{38}NO_4^+$ $[M+H]^+$: 560.2806, Found: 560.2803. HPLC analysis of the reaction product: Daicel Chiralpak IH, hexane/iso-propanol =95: 5,1.0mL/min, λ = 346 nm, retention time:8.42 min (major) and 11.72 min (minor).

(*S*)-4-(9-((1,3-dioxolan-2-yl)methyl)-2-chloro-7-(6-methoxypyridin-3-yl)-9H-fluoren-9-yl)butanoic acid (precursor to **60**)

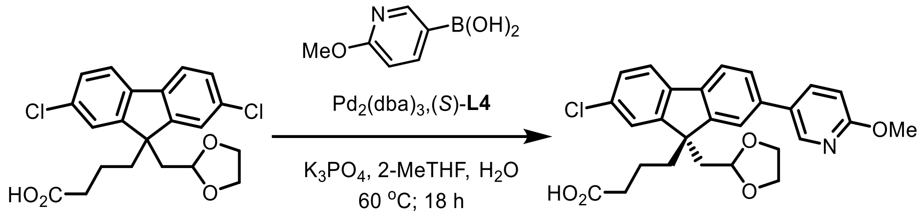

Under N_2 atmosphere, to a mixture of Pd_2dba_3 (4.6 mg, 0.005 mmol) and (*S*)-**L4** (5.9 mg, 0.01 mmol) was added 1 mL 2-MeTHF and the resulting solution was stirred for 20 min before use. Then the metal-ligand complex solution was added to a 9 mL 2-MeTHF solution of 2-Methoxy-5-pyridineboronic acid (92 mg, 0.6 mmol), 4-(9-((1,3-dioxolan-2-yl)methyl)-2,7-dichloro-9H-fluoren-9-yl)butanoic acid (203 mg, 0.5 mmol) and K_3PO_4 (1.06 g, 5 mmol), followed by 0.82 mL H_2O. Then the resulting reaction mixture was stirred at 60 °C for 18 h. The reaction was quenched with water, neutralized to pH 3-5 with 1 M HCl and extracted with ethyl acetate (30 mL X 3) for three times. The combined organic phases were washed with brine, dried over Na_2SO_4 and concentrated in *vacuo*. The crude materials were purified by flash column chromatography with hexane and acetone(20:1-3:1) to give the desired products 136 mg in 57% yield as a white solid. ^{1}H NMR (400 MHz, $CDCl_3$) δ 8.5 – 8.4 (m, 1H), 7.9 (dd, *J* = 8.6, 2.6 Hz, 1H), 7.7 (dd, *J* = 7.8, 0.7 Hz, 1H), 7.6 (d, *J* = 8.1 Hz, 1H), 7.5 – 7.5 (m, 2H), 7.4 (d, *J* = 1.9 Hz, 1H), 7.3 (dd, *J* = 8.1, 1.9 Hz, 1H), 6.8 (dd, J = 8.7, 0.7 Hz, 1H), 4.3 (t, J = 4.7 Hz, 1H), 4.0 (s, 3H), 3.8 – 3.7 (m, 2H), 3.6 – 3.5 (m, 2H), 2.4 – 2.3 (m, 2H), 2.2 – 2.0 (m, 4H), 1.0 – 0.9 (m, 2H). ^{13}C NMR (101 MHz, $CDCl_3$) δ 177.7, 163.5, 151.2, 149.8, 144.8, 139.2, 138.9, 138.1, 137.0, 133.2, 130.6, 127.9, 126.3, 124.1, 122.1, 121.1, 120.5, 111.0, 102.2, 64.6, 64.6, 54.1, 52.7, 43.8, 39.4, 33.7, 18.9. $[\alpha]_D^{20}$ = -8.45 (*c* 1.0 $CHCl_3$) for 95:5 er. HRMS (m/z, ESI): Calcd. for Chemical Formula: $C_{27}H_{25}ClNO_5^-$ $[M-H]^-$: 478.1427, Found: 478.1425. HPLC analysis of the reaction product: Daicel Chiralpak IA, hexane/*iso*-propanol = 80: 20, 1.0 mL/min, λ= 322 nm, retention time: 7.10 min (major) and 5.88 min (minor).

(*R*)-4-(9-((1,3-dioxolan-2-yl)methyl)-2-(3,6-dihydro-2H-pyran-4-yl)-7-(6-methoxypyridin-3-yl)-9H-fluoren-9-yl)butanoic acid (60)

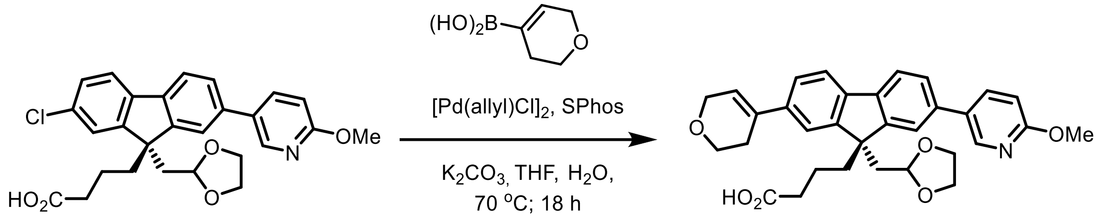

Under N_2 atmosphere, to a mixture of $[Pd(allyl)Cl]_2$ (4.8 mg, 0.013 mmol) and SPhos (10.6 mg, 0.026 mmol) was added 1 mL THF and the resulting solution was stirred for 20 min before use. Then the metal-ligand complex solution was added to a 4 mL THF solution of 3,6-Dihydro-2H-pyran-4-boronic acid pinacol ester (109 mg, 0.52 mmol), (*S*)-4-(9-((1,3-dioxolan-2-yl)methyl)-2-chloro-7-(6-methoxypyridin-3-yl)-9H-fluoren-9-yl)butanoic acid (126 mg, 0.26 mmol) and K_2CO_3 (179 Mg, 1.3 mmol), followed by 0.5 mL H_2O. Then the resulting reaction mixture was stirred at 70 °C for 18 h. The reaction was quenched with water, neutralized to pH 3-5 with 1 M HCl and extracted with ethyl acetate (30 mL X 3) for three times. The combined organic phases were washed with brine, dried over Na_2SO_4 and concentrated in *vacuo*. The crude materials were purified by flash column chromatography with hexane and acetone(20:1-1:1) to give the desired products 125 mg in 91% yield as a white solid. ^{1}H NMR (400 MHz, $CDCl_3$) δ 8.45 (dd, *J* = 2.6, 0.8 Hz, 1H), 7.87 (dd, *J* = 8.6, 2.5 Hz, 1H), 7.73 (d, *J* = 8.0 Hz, 1H), 7.67 (d, *J* = 7.9 Hz, 1H), 7.54 – 7.47 (m, 2H), 7.45 – 7.38 (m, 2H), 6.84 (dd, *J* = 8.6, 0.7 Hz, 1H), 6.18 – 6.20 (m, 1H), 4.39 – 4.33 (m, 3H), 3.99 (s, 3H), 3.96 (t, *J* = 5.4 Hz, 2H), 3.77 – 3.70 (m, 2H), 3.58 – 3.51 (m, 2H), 2.57 – 2.60 (m, 2H), 2.35 (d, *J* = 4.8 Hz, 2H), 2.06 – 2.14 (m, 4H), 0.92 – 0.98 (m, 2H). ^{13}C NMR (101 MHz, $CDCl_3$) δ 177.8, 163.4, 150.2, 149.6, 144.8, 139.9, 139.6, 139.6, 138.1, 136.5, 134.4, 130.8, 126.2, 124.2, 122.5, 122.1, 120.4, 120.0, 119.8, 110.9, 102.4, 66.0, 64.6, 64.5, 54.0, 52.5, 44.0, 39.4, 33.8, 27.5, 19.0. $[\alpha]_D^{20}$ = - 1.69 (*c* 0.13 $CHCl_3$) for 94.5:5.5 er. HRMS (m/z, ESI): Calcd. for Chemical Formula: $C_{32}H_{32}NO_6^-$ $[M-H]^-$:526.2235, Found: 526.2235. HPLC analysis of the reaction product: Daicel Chiralpak IA, hexane/*iso*-propanol = 80: 20, 1.0 mL/min, λ= 325 nm, retention time: 10.96 min (major) and 8.12 min (minor).

(*S*)-4-(9-((1,3-dioxolan-2-yl)methyl)-2-chloro-7-(3,6-dihydro-2H-pyran-4-yl)-9H-fluoren-9-yl)butanoic acid (precursor to ***ent*-60**)

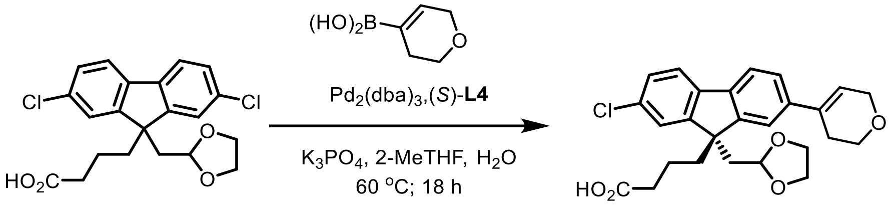

Under N_2 atmosphere, to a mixture of Pd_2dba_3 (3.76 mg, 0.00411 mmol) and (*S*)-**L4** (4.84 mg, 0.00822 mmol) was added 1 mL 2-MeTHF and the resulting solution was stirred for 20 min before use. Then the metal-ligand complex solution was added to a 7.2 mL 2-MeTHF solution of 3,6-Dihydro-2H-pyran-4-boronic acid pinacol ester (102 mg, 0.493 mmol), 4-(9-((1,3-dioxolan-2-yl)methyl)-2,7-dichloro-9H-fluoren-9-yl)butanoic acid (167 mg, 0.411 mmol) and K_3PO_4 (871 mg, 4.11 mmol), followed by 0.67mL H_2O. Then the resulting reaction mixture was stirred at 60 °C for 18 h. The reaction was quenched with water, neutralized to pH 3-5 with 1 M HCl and extracted with ethyl acetate (15 mL X 3) for three times. The combined organic phases were washed with brine, dried over Na_2SO_4 and concentrated in *vacuo*. The crude materials were purified by flash column chromatography with hexane and acetone (20:1-3:1) to give the desired products 98 mg in 53% yield as a white solid. ^{1}H NMR (500 MHz, $CDCl_3$) δ 7.63 – 7.57 (m, 2H), 7.40 – 7.35 (m, 3H), 7.31 (dd, J = 8.1, 1.9 Hz, 1H), 6.17–6.19 (m, 1H), 4.29–4.35 (m, 2H), 4.29 (t, J = 4.7 Hz, 1H), 3.95 (t, J = 5.5 Hz, 2H), 3.73–3.75 (m, 2H), 3.52–3.55 (m, 2H), 2.55–2.59 (m, 2H), 2.31 (t, J = 4.8 Hz, 2H), 2.06–2.10 (m, 4H), 0.91 – 0.85 (m, 2H). ^{13}C NMR (126 MHz, $CDCl_3$) δ 177.7, 151.2, 149.1, 139.8, 139.12, 139.05, 134.3, 132.9, 127.8, 124.3, 124.1, 122.7, 120.9, 119.9, 119.8, 102.2, 68.1, 66.0, 64.6, 64.5, 52.6, 43.8, 39.4, 33.6, 27.5, 18.8. $[\alpha]_D^{20}$ = - 10.9 (*c* 1.0 $CHCl_3$) for 95 : 5 er. HRMS (m/z, ESI): Calcd. for Chemical Formula: $C_{26}H_{26}ClO_5^-$[M-H]$^-$: 454.1474, Found: 453.1478. HPLC analysis of the reaction product: Daicel Chiralpak IA, hexane/*iso*-propanol = 95 : 5, 1.0 mL/min, λ= 318 nm, retention time: 20.44 min (major) and 24.34 min (minor).

(*S*)-4-(9-((1,3-dioxolan-2-yl)methyl)-2-(3,6-dihydro-2H-pyran-4-yl)-7-(6-methoxypyridin-3-yl)-9H-fluoren-9-yl)butanoic acid (*ent*-60)

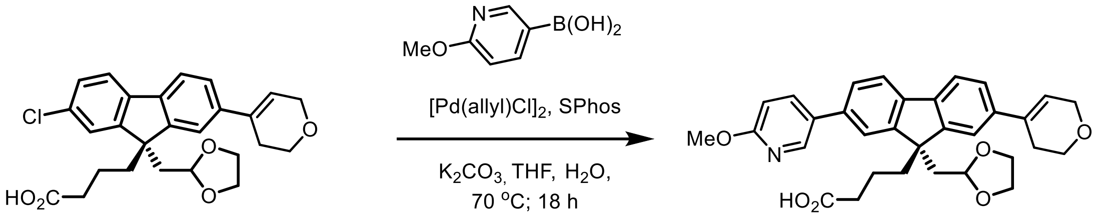

Under N_2 atmosphere, to a mixture of $[Pd(allyl)Cl]_2$ (3.2 mg, 0.0088 mmol) and SPhos (7.2 mg, 0.0176 mmol) was added 0.5 mL THF and the resulting solution was stirred for 20 min before use. Then the metal-ligand complex solution was added to a 2.5 mL THF solution of 2-Methoxy-5-pyridineboronic acid (54 mg, 0.352 mmol), (*S*)-4-(9-((1,3-dioxolan-2-yl)methyl)-2-chloro-7-(3,6-dihydro-2H-pyran-4-yl)-9H-fluoren-9-yl)butanoic acid (80 mg, 0.176 mmol) and K_2CO_3 (121 mg, 0.88 mmol), followed by 0.4 mL H_2O. Then the resulting reaction mixture was stirred at 70 °C for 18 h. The reaction was quenched with water, neutralized to pH 3-5 with 1 M HCl and extracted with ethyl acetate (30 mL X 3) for three times. The combined organic phases were washed with brine, dried over Na_2SO_4 and concentrated in *vacuo*. The crude materials were purified by flash column chromatography with hexane and acetone (20:1-1:1) to give the desired products 80 mg in 86% yield as a white solid. $[\alpha]_D^{20}$ = + 2.4 (*c* 1.0 $CHCl_3$) for 95:5 er. HRMS (m/z, ESI): Calcd. for Chemical Formula: $C_{32}H_{32}NO_6^-$ [M-H]$^-$:526.2235, Found: 526.2235. HPLC analysis of the reaction product: Daicel Chiralpak IA, hexane/*iso*-propanol = 80: 20, 1.0 mL/min, λ= 324 nm, retention time: 8.44 min (major) and 11.55min (minor).

Methyl (R)-3-amino-2'-(3,6-dihydro-2H-pyran-4-yl)-7'-(6-methoxypyridin-3-yl)spiro[cyclohexane-1,9'-fluoren]-3-ene-4-carboxylate (61)

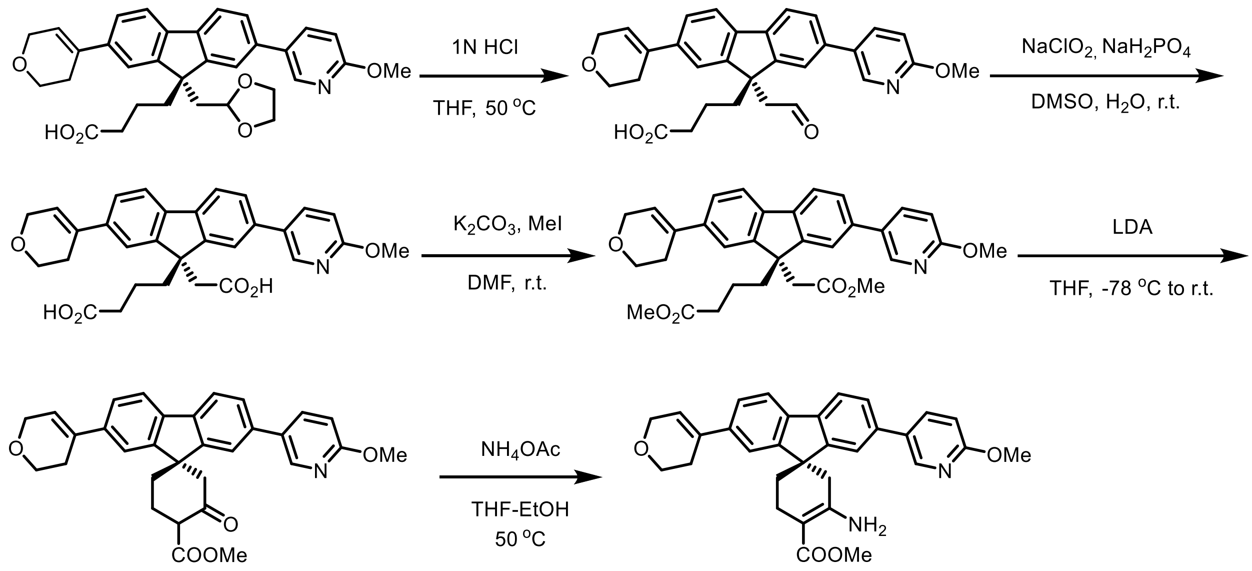

(*R*)-4-(9-((1,3-dioxolan-2-yl)methyl)-2-(3,6-dihydro-2H-pyran-4-yl)-7-(6-methoxypyridin-3-yl)-9H-fluoren-9-yl)butanoic acid (120 mg, 0.23 mmol) was dissolved in 1.0 mL THF and 1 mL 1M HCl was added. The reaction mixtures were stirred at 50 °C overnight and solvents were removed under reduced pressure. Water was added and the reaction solution was extracted with ethyl acetate (10 mL X 3) three times. The combined organic phases were washed with brine, dried over Na_2SO_4, and concentrated in *vacuo*. The crude product was directly subjected to the next step without further purification.

Under N_2 atmosphere, the above mixture and NaH_2PO_4 (115 mg, 0.96 mmol) were dissolved in DMSO (2 mL) and H_2O (0.7 mL). $NaClO_2$ (32.4 mg, 0.36 mmol) in H_2O (0.7 mL) was added dropwise into the above solution at room temperature[15]. After addition was completed, the resulting reaction mixture was stirred at room temperature overnight. The reaction was quenched with 1 M HCl solution to make pH=2, and extracted with ethyl acetate three times. The combined organic phases were washed with brine, dried over Na_2SO_4, and concentrated in *vacuo*. The crude mixture was directly subjected to the next step without further purification.

Under N_2 atmosphere, the above mixture and K_2CO_3 (138 mg, 1 mmol) were dissolved in DMF (3 mL). MeI (86 mg, 0.60 mmol) was added dropwise into the above solution at room temperature. After addition was completed,

the resulting reaction mixture was stirred at room temperature for 2 h. The reaction was quenched with saturated NH_4Cl solution and extracted with ethyl acetate three times. The combined organic phases were washed with brine, dried over Na_2SO_4, and concentrated in *vacuo*. The crude mixture was purified by column chromatography on silica gel (hexane: acetone = 20:1) to afford the product (40 mg, 33% yield over three steps) as a white solid.

Under N_2 atmosphere, methyl (R)-4-(2-(3,6-dihydro-2H-pyran-4-yl)-9-(2-methoxy-2-oxoethyl)-7-(6-methoxypyridin-3-yl)-9H-fluoren-9-yl)butanoate (40 mg, 0.076 mmol) was dissolved in THF (1 mL). LDA (2M, 76 μL, 0.152 mmol) was added dropwise into the above solution at -78 ^oC. After addition was completed, the resulting reaction mixture was allowed to warm to room temperature and stirred for 3 h[16]. The reaction was quenched with saturated NH_4Cl solution and extracted with ethyl acetate three times. The combined organic phases were washed with brine, dried over Na_2SO_4, and concentrated in *vacuo*. The crude mixture was directly subjected to the next step without further purification.

The above mixture and NH_4OAc (23 mg, 0.31 mmol) were dissolved in THF (0.5 mL) and EtOH (1 mL). The reaction mixture was stirred at 50 ^oC overnight[17]. The reaction was quenched with H_2O and extracted with ethyl acetate three times. The combined organic phases were washed with brine, dried over Na_2SO_4, and concentrated in *vacuo*. The crude mixture was purified by column chromatography on silica gel (hexane: ethyl acetate = 10:1-5:1) to afford the target compound (14 mg, 37% yield over two steps) as a white solid. ^1H NMR (400 MHz, $CDCl_3$) δ 8.41 (d, J = 2.5 Hz, 1H), 7.83 – 7.76 (m, 2H), 7.72 (d, J = 7.9 Hz, 1H), 7.56 – 7.51 (m, 2H), 7.47 – 7.40 (m, 2H), 6.84 (d, J = 8.6 Hz, 1H), 6.14 – 6.16 (m, 1H), 4.34 – 4.38 (m 2H), 4.01 (d, J = 1.1 Hz, 3H), 3.96 (t, J = 5.4 Hz, 2H), 3.79 (s, 3H), 2.66 – 2.71 (m, 2H), 2.63 – 2.52 (m, 4H), 1.92 – 1.82 (m, 2H). ^{13}C NMR (101 MHz, $CDCl_3$) δ 170.5, 155.7, 152.1, 151.5, 140.1, 138.8, 138.4, 137.9, 134.4, 130.5, 126.5, 124.5, 122.8, 122.2, 120.6, 120.1, 120.0, 111.1, 91.5, 66.1, 64.6, 53.9, 51.0, 48.8, 39.5, 32.9, 27.6, 22.3. $[\alpha]_D^{20}$ = + 8.3 (*c* 1.0 $CHCl_3$) for 94.5:5.5 er. HRMS (m/z, ESI): Calcd. for Chemical Formula: $C_{31}H_{31}N_2O_4^+[M+H]^+$: 495.2278, Found: 495.2276. HPLC analysis of the reaction product: Daicel Chiralpak IA, hexane/*iso*-propanol = 90: 10, 1.0 mL/min, λ= 324 nm, retention time: 50.46 min (major) and 66.92 min (minor).

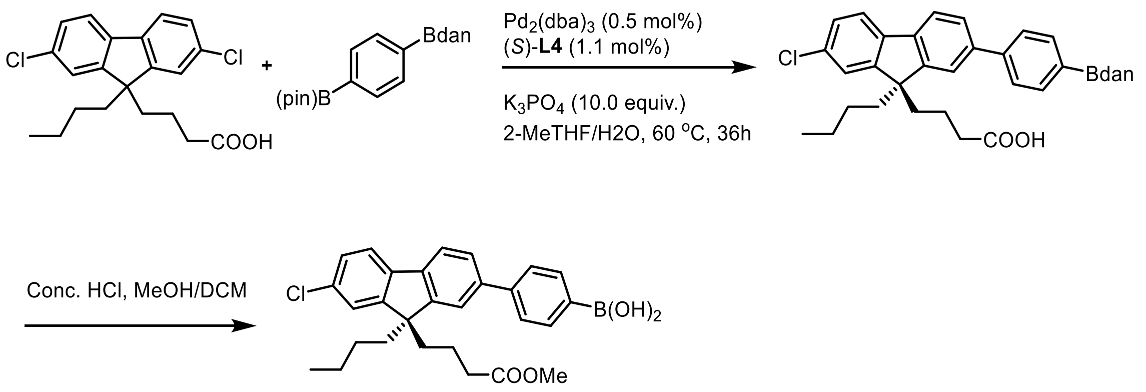

(*R*)-4-(2-(4-(1H-naphtho[1,8-de][1,3,2]diazaborinin-2(3H)-yl)phenyl)-9-butyl-7-chloro-9H-fluoren-9-yl)butanoic acid (62)

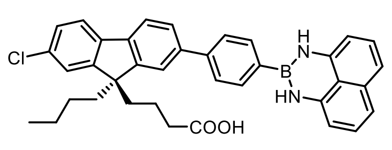

Under N_2 atmosphere, to a mixture of Pd_2dba_3 (4.6 mg, 0.005 mmol) and (*S*)-**L4** (6.4 mg, 0.011 mmol) was added 1.0 mL 2-MeTHF and the resulting solution was stirred for 20 min before use. Then metal-ligand complex solution was added to a 20 mL 2-MeTHF solution of 4-(9-butyl-2,7-dichloro-9H-fluoren-9-yl)butanoic acid **51** (377.6 mmol, 1.0 mmol), 2-(4-(4,4,5,5-tetramethyl-1,3,2-dioxaborolan-2-yl)phenyl)-2,3-dihydro-1H-naphtho[1,8-de][1,3,2]diazaborinine (0.407 g, 1.1 mmol) and K_3PO_4 (2.12 g, 10.0 mmol), followed by 1.5 mL H_2O. Then the resulting reaction mixture was stirred at 60 °C for 36 h. The reaction was quenched with water, neutralized to pH 3-5 with 1 M HCl and extracted with ethyl acetate for three times. The combined organic phases were washed with brine, dried over Na_2SO_4 and concentrated in *vacuo*. The crude materials were purified by flash column chromatography with hexane and ethyl acetate (6:1-1:1) to give the desired products 0.36 g in 61% yield as a brown solid. ^1H NMR (500 MHz, $CDCl_3$) δ 7.79 – 7.69 (m, 5H), 7.66 – 7.60 (m, 2H), 7.58-7.57 (m, 1H), 7.36 – 7.32 (m, 2H), 7.19 (t, *J* = 7.8 Hz, 2H), 7.11 (d, *J* = 8.2 Hz, 2H), 6.46 (d, *J* = 7.2 Hz, 2H), 6.08 (s, 2H), 2.16 – 1.98 (m, 6H), 1.12 (dt, *J* = 14.9, 7.4 Hz, 2H), 1.03 (dt, *J* = 10.8, 5.1 Hz, 2H), 0.73 – 0.64 (m, 5H). ^{13}C NMR (126 MHz, $CDCl_3$) δ 178.5, 152.1, 150.5, 143.1, 141.1, 140.1, 139.7, 139.3, 136.4, 133.1, 132.0, 127.7, 127.5, 127.0, 126.5, 123.3, 121.5, 121.0, 120.3, 117.9, 106.1, 55.2, 40.2, 39.4, 33.8, 25.8, 22.9, 19.2, 13.8. HRMS (m/z, ESI): Calcd. for Chemical Formula: $C_{37}H_{35}ClBN_2O_2^+$ $[M+H]^+$: 585.2475, Found: 585.2486.

(*R*)-(4-(9-butyl-7-chloro-9-(4-methoxy-4-oxobutyl)-9H-fluoren-2-yl)phenyl)boronic acid (63)

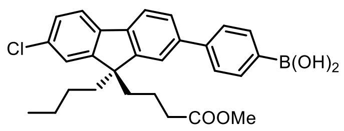

(*R*)-4-(2-(4-(1H-naphtho[1,8-de][1,3,2]diazaborinin-2(3H)-yl)phenyl)-9-butyl-7-chloro-9H-fluoren-9-yl)butanoic acid (30 mg) was dissolved in 0.75 mL DCM and 0.75 mL MeOH, then 20 µL conc. HCl was added. The reaction mixture was stirred at room temperature for 15 h[18]. Then the reaction was quenched by water and extracted with DCM (4 mL X 3) for three times. The combined organic phases were washed with brine, dried over Na_2SO_4 and concentrated in *vacuo*. The crude materials were purified by flash column chromatography to give the desired product as a white solid 16.3 mg in 67% yield. $[\alpha]_D^{20}$ = +3.20 (*c* 0.080 $CHCl_3$) for 97:3 er. ^{1}H NMR (500 MHz, Acetone) δ 8.01 (d, *J* = 8.2 Hz, 2H), 7.92 (d, *J* = 7.9 Hz, 1H), 7.87 (d, *J* = 8.1 Hz, 1H), 7.84 (d, *J* = 1.3 Hz, 1H), 7.77 (d, *J* = 8.2 Hz, 2H), 7.74 (dd, *J* = 7.9, 1.7 Hz, 1H), 7.55 (d, *J* = 1.9 Hz, 1H), 7.42 (dd, *J* = 8.1, 1.9 Hz, 1H), 3.50 (s, 3H), 2.28 – 2.09 (m, 6H), 1.12 (dd, *J* = 14.8, 7.4 Hz, 2H), 0.99 – 0.92 (m, 2H), 0.70 – 0.61 (m, 5H). ^{13}C NMR (126 MHz, Acetone) δ 172.8, 152.7, 150.8, 142.8, 140.5, 139.8, 139.5, 134.8, 127.3, 126.3, 126.1, 123.3, 121.5, 121.2, 120.4, 50.5, 39.8, 38.9, 33.4, 25.9, 22.7, 19.5, 13.2. HRMS (m/z, ESI): Calcd. for Chemical Formula: $C_{28}H_{31}BClO_4^+$ $[M+H]^+$: 477.1998, Found: 477.2008. HPLC analysis of the reaction product: Daicel Chiralpak IA, hexane/*iso*-propanol = 97: 3, 1.0 mL/min, λ= 333 nm, retention time: 14.24 min (major) and 14.94 min (minor).

4,4'-((9*R*,9'*R*)-1,4-phenylenebis(9-butyl-7-chloro-9H-fluorene-2,9-diyl))dibutyric acid (64)

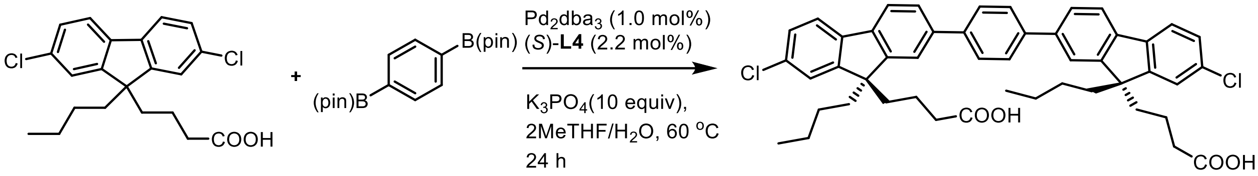

Under N_2 atmosphere, to a mixture of Pd_2dba_3 (3.0 mg, 0.0033 mmol) and (*S*)-**L4** (4.0 mg, 0.0066 mmol) was added 1.0 mL 2-MeTHF and the resulting solution was stirred for 20 min before use. Then the metal-ligand complex solution was added to a 19.0 mL 2-MeTHF solution of 4-(9-butyl-2,7-dichloro-9H-fluoren-9-yl)butanoic acid **51** (377.3 mg, 1.0 mmol), 1,4-bis(4,4,5,5-tetramethyl-1,3,2-dioxaborolan-2-yl)benzene (110.0 mg, 0.33 mmol) and K_3PO_4 (2.123 g, 10.0 mmol), followed by 1.65 mL H_2O. Then the resulting reaction mixture was stirred at 60 °C for 36 h. The reaction was quenched with water, neutralized to pH 3-5 with 1 M HCl and extracted with ethyl acetate (3 mL X 3) for three times. The combined organic phases were washed with brine, dried over Na_2SO_4 and concentrated in *vacuo*. Then the crude product was dissolved in 1.0 mL EtOH again and 10 μL conc. H_2SO_4 was added and the resulting reaction mixture was heated at 70 °C for 12 h. The reaction was quenched with water and extracted with ethyl acetate (2 mL X 3) for three times. The combined organic phases were washed with brine, dried over Na_2SO_4 and concentrated in *vacuo*. The crude materials were purified by flash column chromatography with hexane and ethyl acetate (6:1-1:1) to give the desired products 112.1 mg in 42% yield as a white solid, $[\alpha]_D^{20}$ = -76.00 (*c* 0.010 $CHCl_3$), 99:1 er. Diethyl ester product: ^1H NMR (500 MHz, $CDCl_3$) δ 7.79 (s, 4H), 7.77 (d, *J* = 7.9 Hz, 2H), 7.70 – 7.65 (m, 4H), 7.61 (d, *J* = 1.2 Hz, 2H), 7.39 – 7.32 (m, 4H), 4.09 – 4.02 (m, 4H), 2.14 – 1.99 (m, 12H), 1.20 (t, *J* = 7.1 Hz, 6H), 1.14-1.12 (m, 4H), 1.09 – 1.01 (m, 4H), 0.74-0.67 (m, 10H). ^{13}C NMR (126 MHz, $CDCl_3$) δ 173.3, 152.3, 150.6, 140.3, 140.0, 139.5, 139.4, 133.0, 127.6, 127.5, 126.3, 123.3, 121.4, 120.9, 120.2, 60.2, 55.2, 40.2, 39.6, 34.4, 25.9, 23.0, 19.5, 14.2, 13.8. MS (m/z, ESI): Calcd. for Chemical Formula: $C_{52}H_{56}Cl_2O_4^-$ $[M-H]^-$: 837.3448, Found: 837.3440. HPLC analysis of the dicarboxylic acid, prepared through hydrolysis of the above diethyl ester product under aqueous basic conditions (NaOH in water-THF): Daicel Chiralpak IH, hexane/iso-propanol =98: 2, 1.0 mL/min, λ = 329 nm, retention time: 35.09 min (major) and 48.22 min (minor).

(*meso*)-4,4'-((9*R*,9'*S*)-1,4-phenylenebis(9-butyl-7-chloro-9H-fluorene-2,9-diyl))dibutyric acid (*meso*-64)

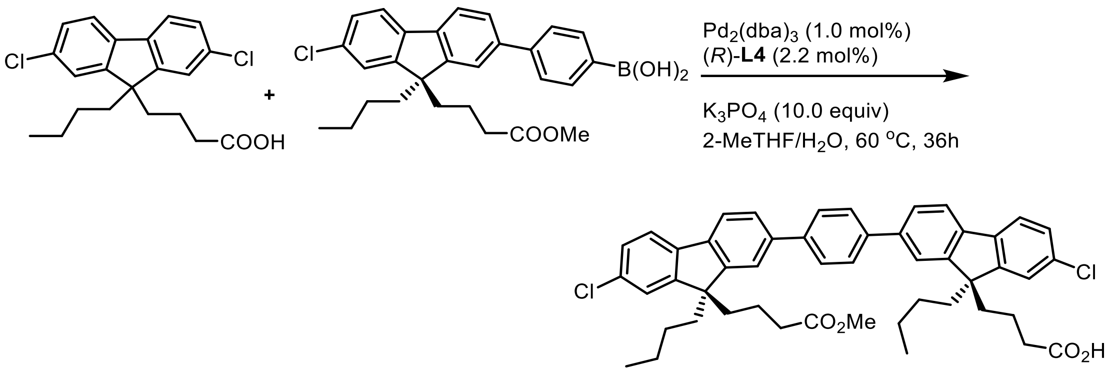

Under N_2 atmosphere, to a mixture of Pd_2dba_3 (0.3 mg, 0.00033 mmol) and (*R*)-**L4** (0.43 mg, 0.0073 mmol) was added 0.3 mL 2-MeTHF and the resulting solution was stirred for 20 min before use. Then the metal-ligand complex solution was added to a 0.3 mL 2-MeTHF solution of (*R*)-(4-(9-butyl-7-chloro-9-(4-methoxy-4-oxobutyl)-9H-fluoren-2-yl)phenyl)boronic acid (13.7 mg, 0.033 mmol), 4-(9-butyl-2,7-dichloro-9H-fluoren-9-yl)butanoic acid (37.7 mg, 0.1 mmol) and K_3PO_4 (77.3 mg, 3.3 mmol), followed by 0.055 mL H_2O. Then the resulting reaction mixture was stirred at 60 °C for 36 h. The reaction was quenched with water, neutralized to pH 3-5 with 1 M HCl and extracted with ethyl acetate (3 mL X 3) for three times. The combined organic phases were washed with brine, dried over Na_2SO_4 and concentrated in *vacuo*. The crude materials were purified by flash column chromatography with hexane and ethyl acetate (6:1-1:1) to give the desired products 26.2 mg in 34% yield as a white solid. Mono methyl ester product: ^{1}H NMR (500 MHz, $CDCl_3$) δ 7.80 – 7.74 (m, 6H), 7.66 (dd, *J* = 8.5, 4.6 Hz, 4H), 7.61 (s, 2H), 7.36 (dq, *J* = 4.1, 2.0 Hz, 4H), 3.59 (s, 3H), 2.15 – 2.00 (m, 12H), 1.15 – 1.10 (m, 4H), 1.07 – 1.02 (m, 4H), 0.74 – 0.66 (m, 10H). ^{13}C NMR (126 MHz, $CDCl_3$) δ 177.4, 173.7, 152.2, 152.1, 150.6, 150.5, 140.3, 140.0, 139.4, 139.3, 133.1, 127.6, 127.5, 126.3, 126.3, 123.3, 121.4, 120.9, 120.2, 55.2, 51.4, 40.2, 39.6, 39.4, 34.1, 33.7, 31.9, 29.4, 25.9, 23.0, 19.5, 19.2, 13.8. MS (m/z, ESI): Calcd. for Chemical Formula: $C_{49}H_{50}Cl_2O_4Na^+$ $[M+Na]^+$:795.2978, Found: 795.2974. HPLC analysis of the *meso*-dicarboxylic acid, prepared through hydrolysis of the above methyl ester product under aqueous basic conditions (NaOH in water-THF): Daicel Chiralpak IH, hexane/iso-propanol =98: 2,1.0 mL/min, λ = 329 nm, retention time: 32.9 min.

(*R*)-4-(9-butyl-2-chloro-7-(4-cyanophenyl)-9H-fluoren-9-yl)butanoic acid (precursor to **65**)

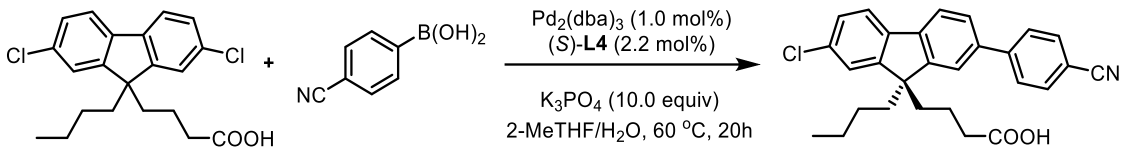

Under N_2 atmosphere, to a mixture of Pd_2dba_3 (2.3 mg, 0.0025 mmol) and (*S*)-**L4** (3.2 mg, 0.0055 mmol) was added 1.0 mL 2-MeTHF and the resulting solution was stirred for 20 min before use. Then metal-ligand complex solution was added to a 3 mL 2-MeTHF solution of 4-(9-butyl-2,7-dichloro-9H-fluoren-9-yl)butanoic acid **51** (188.8 mmol, 0.5 mmol), (4-cyanophenyl)boronic acid (162 mg, 1.1 mmol) and K_3PO_4 (1.01 g, 10.0 mmol), followed by 0.4 mL H_2O. Then the resulting reaction mixture was stirred at 60 °C for 20 h. The reaction was quenched with water, neutralized to pH 3-5 with 1 M HCl and extracted with ethyl acetate for three times. The combined organic phases were washed with brine, dried over Na_2SO_4 and concentrated in *vacuo*. The crude materials were purified by flash column chromatography with hexane and ethyl acetate (6:1-1:1) to give the desired products 0.132 g in 60% yield as a white solid. $[\alpha]_D^{20}$ = +3.60 (*c* 1.0 $CHCl_3$) for 95.5:4.5 er. ^1H NMR (400 MHz, $CDCl_3$) δ 7.77 – 7.69 (m, 5H), 7.67 – 7.63 (m, 1H), 7.57 (dd, J = 7.9, 1.7 Hz, 1H), 7.53 – 7.49 (m, 1H), 7.34 (d, J = 7.5 Hz, 2H), 2.15 – 2.02 (m, 4H), 1.96 –2.01 (m, 2H), 1.12 – 1.04 (m, 2H), 0.94 – 0.98 (m, 2H), 0.68 (t, J = 7.3 Hz, 3H), 0.61 (t, J = 7.3 Hz, 2H). ^{13}C NMR (101 MHz, $CDCl_3$) δ 178.2, 152.1, 150.7, 145.8, 140.7, 138.9, 138.4, 133.6, 132.6, 127.8, 127.7, 126.7, 123.3, 121.6, 121.2, 120.5, 119.0, 110.8, 55.3, 40.2, 39.3, 33.6, 25.8, 22.9, 19.0, 13.8. HRMS (m/z, ESI): Calcd. for Chemical Formula: $C_{28}H_{25}ClNO_2^-$ [M-H]$^-$: 442.1579, Found: 442.1572. HPLC analysis of the reaction product: Daicel Chiralpak IA, hexane/*iso*-propanol = 97: 3, 1.0 mL/min, λ= 322 nm, retention time: 13.85min (major) and 15.68 min (minor).

(*R*)-4-(9-butyl-7-chloro-9-(4-hydroxybutyl)-9H-fluoren-2-yl)benzonitrile (precursor to **65**)

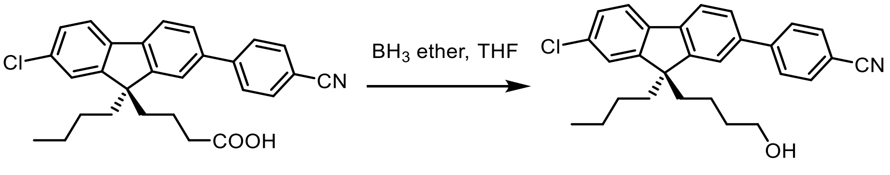

Under N_2 atmosphere, to a solution of (*R*)-4-(9-butyl-7-chloro-9-(4-hydroxybutyl)-9H-fluoren-2-yl)benzonitrile (60 mg, 15 mmol) in anhydrous 1.0 mL THF was added BH_3-ether solution (67 µL, 0.134 mmol, 2 mol/L) dropwise at -78 °C. After addition was completed, the reaction mixture was stirred at this temperature for 20 min.

Then the reaction was transferred to -15 °C and stirred for 12 h[19]. The reaction was quenched with water and extracted with ethyl acetate for three times. The combined organic phases were washed with brine, dried over Na_2SO_4 and concentrated in *vacuo*. The crude materials were purified by flash column chromatography with hexane and ethyl acetate (6:1-1:1) to give the desired products 42 mg in 73% yield as a white solid. ^{1}H NMR (400 MHz, $CDCl_3$) δ 7.77 – 7.72 (m, 5H), 7.66 – 7.63 (m, 1H), 7.58 (dd, J = 7.9, 1.7 Hz, 1H), 7.52 (dd, J = 1.7, 0.6 Hz, 1H), 7.36 – 7.32 (m, 2H), 3.45 – 3.39 (m, 2H), 2.09 – 1.97 (m, 4H), 1.38 – 1.34 (m, 2H), 1.10 (q, J = 7.4 Hz, 2H), 0.76 – 0.66 (m, 5H), 0.65 – 0.57 (m, 2H). ^{13}C NMR (126 MHz, $CDCl_3$) δ 152.8, 151.4, 146.0, 141.0, 139.1, 138.5, 133.8, 132.8, 128.0, 127.7, 126.7, 123.5, 121.6, 121.3, 120.6, 119.2, 111.0, 62.8, 55.7, 40.4, 40.3, 33.2, 26.1, 23.2, 20.4, 14.0.

(*R*)-4-(9-butyl-7-chloro-9-(4-(2-(2-methoxyethoxy)ethoxy)butyl)-9H-fluoren-2-yl)benzonitrile (65)

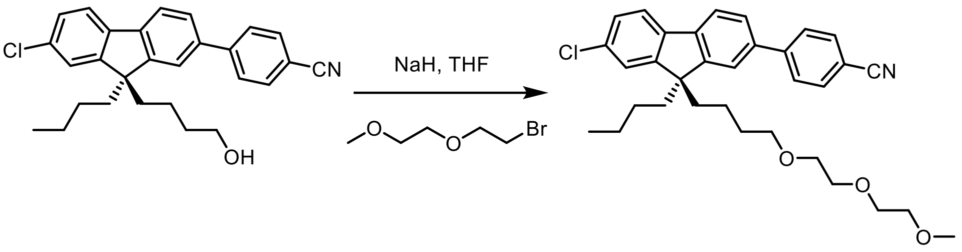

Under N_2 atmosphere, to a solution of (*R*)-4-(9-butyl-7-chloro-9-(4-(2-(2-methoxyethoxy)ethoxy)butyl)-9H-fluoren-2-yl)benzonitrile (15 mg, 0.035 mmol) in anhydrous 1.0 mL THF was added NaH (2 mg, 0.052 mmol) at 0 °C. After addition was completed, the reaction mixture was stirred at room temperature for 1 hour. Then 1-bromo-2-(2-methoxyethoxy)ethane (9.6 mg, 0.052 mmol)was added dropwise at room temperature and the reaction mixture was heated to 45 °C and stirred for 24 h. The reaction was quenched with water and extracted with ethyl acetate for three times. The combined organic phases were washed with brine, dried over Na_2SO_4 and concentrated in *vacuo*. The crude materials were purified by flash column chromatography with hexane and ethyl acetate (6:1-1:1) to give the desired products 10 mg in 54% yield as a white solid. $[\alpha]_D^{20}$ = +32 (*c* 0.6 $CHCl_3$) for 95:5 er. ^{1}H NMR (400 MHz, $CDCl_3$) δ 7.77 – 7.72 (m, 5H), 7.64 (d, J = 8.7 Hz, 1H), 7.58 (dd, J = 7.9, 1.7 Hz, 1H), 7.52 (dd, J = 1.7, 0.7 Hz, 1H), 7.35 – 7.31 (m, 2H), 3.56 – 3.50 (m, 4H), 3.50 – 3.46 (m, 2H), 3.45 – 3.42 (m, 2H), 3.34 (s, 3H), 3.22 (t, J = 6.7 Hz, 2H), 1.97 - 2.04 (m, 4H), 1.37 (dt, J = 15.2, 6.9 Hz, 2H), 1.09 (q, J = 7.4 Hz, 2H), 0.68 (t, J = 7.3 Hz, 5H), 0.61 (t, J = 7.4 Hz, 2H). ^{13}C NMR (126 MHz, $CDCl_3$) δ 152.9, 151.5, 146.1, 141.0, 139.1, 138.5, 133.7, 132.8, 128.0, 127.6, 126.7, 123.6, 121.7,

121.3, 120.6, 119.2, 111.0, 72.1, 71.2, 70.7, 70.7, 70.2, 59.2, 55.7, 40.4, 40.3, 30.0, 26.1, 23.2, 20.7, 14.0. HRMS (m/z, ESI): Calcd. for Chemical Formula: $C_{33}H_{39}ClNO_3^+$ $[M+H]^+$: 536.2613, Found: 523.2611. HPLC analysis of the reaction product: Daicel Chiralpak IA, hexane/*iso*-propanol = 99: 1, 0.6 mL/min, λ= 322 nm, retention time: 35.54 min (major) and 33.84 min (minor).

References

[1] Park, Y.; Jeon, I.; Shin, S.; Min, J.; Lee, P. H. *J. Org. Chem.* **2013**, *78*, 10209.

[2] Fleckenstein, C. A.; Plenio, H. *Chem. Eur. J.* **2007**, *13*, 2701.

[3] Chen, J.-J.; Onogi, S.; Hsieh, Y.-C.; Hsiao, C.-C.; Higashibayashi, S.; Sakurai, H.; Wu, Y.-T. *Adv. Synth. Catal.* **2012**, *354*, 1551.

[4] Voortman, T. P.; Chiechi, R. C. *ACS Appl. Mater. Interfaces* **2015**, *7*, 28006.

[5] Gaylord, B. S.; Bartholomew, G. P.; Baldocchi, R. A.; Hong, J. W.; Huisman, W. H.; Liang, Y.; Nguyen, T.; Tran, L. P.; Wheeler, J. M.; Plamer, A. C. V.; Uckert, F. P., WO 2011091086 A1, 28 July 2011.

[6] Bihovsky, R.; Levinson, B. L.; Loewi, R. C.; Erhardt, P. W.; Polokoff, M. A. *J. Med. Chem.* **1995**, *38*, 2119.

[7] Sivets, G. G. *Carbohydrate Research* **2020**, *488*, 107901.

[8] a) Ostermann, N.; Ruedisser, S.; Ehrhardt, C.; Breitenstein, W.; Marzinzik, A.; Jacoby, E.; Vangrevelinghe, E.; Ottl, J.; Klumpp, M.; Hartwieg, J. C.; Cumin, F.; Hassiepen, U.; Trappe, J.; Sedrani, R.; Geisse, S.; Gerhartz, B.; Richert, P.; Francotte, E.; Wagner, T.; Kromer, M.; Kosaka, T.; Webb, R. L.; Rigel, D. F.; Maibaum, J.; Baeschlin, D. K. *J. Med. Chem.* **2013**, 56, 2196; b) Cochran, B. M. *Synlett*. **2016**, *27*, 245.

[9] Jepsen, T. H.; Larsen, M.; Jørgensen, M.; Solanko, K. A.; Bond, A. D.; Kadziola, A.; Nielsen, M. B. *Eur. J. Org. Chem.* **2011**, 53.

[10] Yang, F.; Zhou, B. H.; Chen, P.; Zou, D.; Luo, Q. N.; Ren, W. Z.; Li, L. L.; Fan, L. M.; Li, J. *Molecules*, **2018**, *23*, 1922.

[11] Gormisky, P. E.; White, M. C. *J. Am. Chem. Soc.* **2011**, *133*, 12584.

[12] Trujillo, John I.; Gopalan, Aravamudan S. *Tetrahedron Lett.* **1993**, *34*, 7355.

[13] Ishiyama, T.; Ishida, K.; Miyaura, N. *Tetrahedron* **2001**, *57*, 9813.

[14] Maiti, D.; Fors, B. P.; Henderson, J. L.; Nakamura, Y.; Buchwald, S. L. *Chem. Sci.* **2011**, *2*, 57.

[15] Dalcanale, E.; Montanari, F. *J. Org. Chem.* **1986**, *51*, 567.

[16] Schepens, W.; Haver, D. V.; Vandewalle, M.; Bouillon, R.; Verstuyf, A.; De Clercq, P. J. *Org. Lett.* **2006**, *8*, 4247.

[17] Litvic, M.; Filipan, M.; Pogorelic, I.; Cepanec, I. *Green Chem.* **2005**, *7*, 771.

[18] Noguchi, H.; Shioda, T.; Chou, C.-M.; Suginome, M. *Org. Lett.* **2008**, *10*, 377.

[19] Yoon, N. M.; Pak, C. S.; Brown, H. C.; Krishnamurthy, S.; Stocky, T. P. *J. Org. Chem* **1973**, *38*, 2786.

NMR spectra

^{1}H NMR (500 MHz, $CDCl_3$)

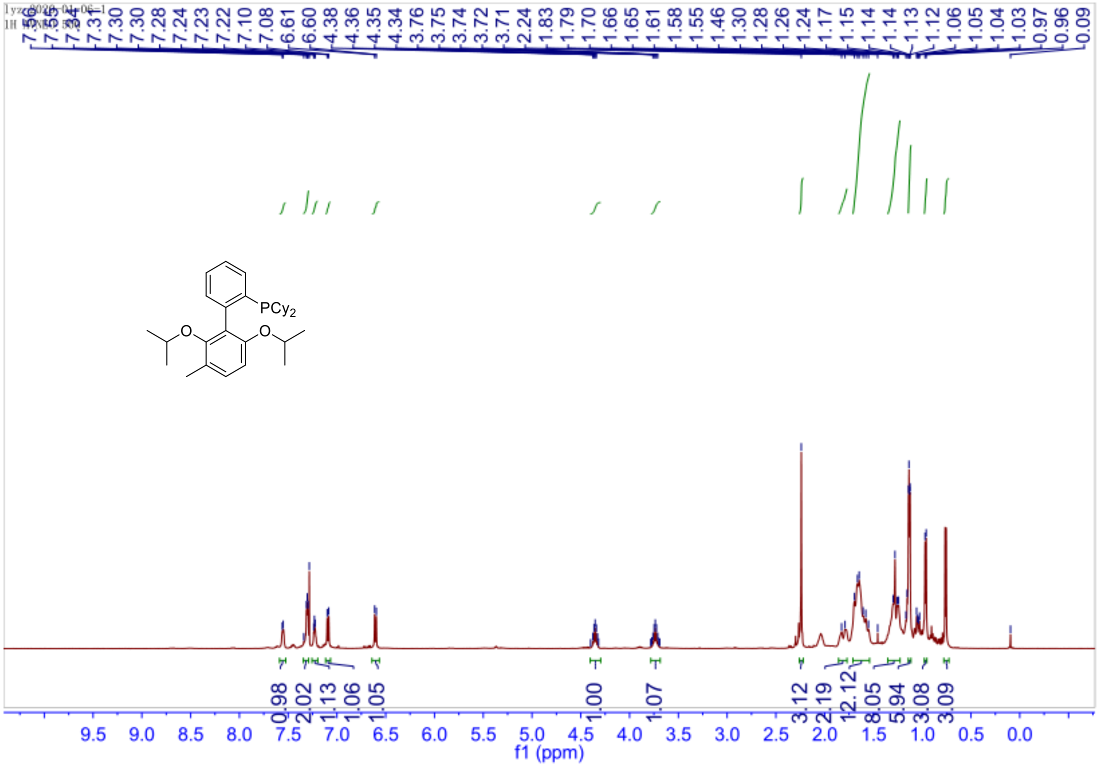

^{13}C NMR (126 MHz, $CDCl_3$)

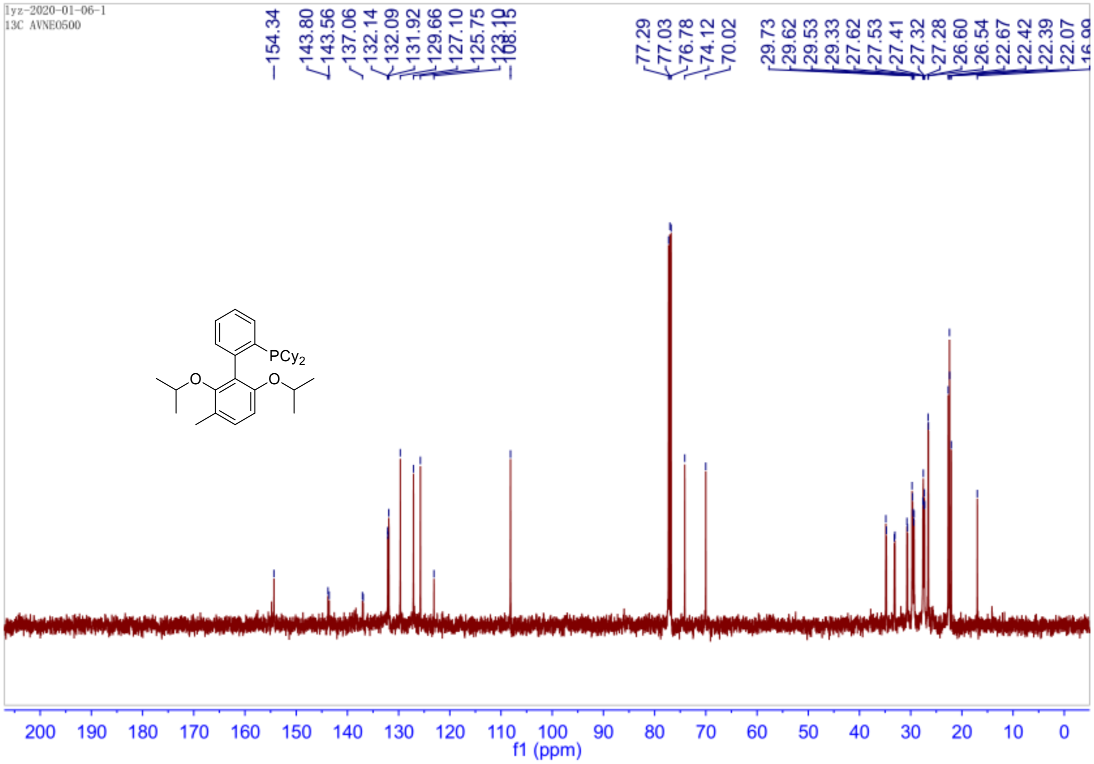

^{31}P NMR (202 MHz, $CDCl_3$)

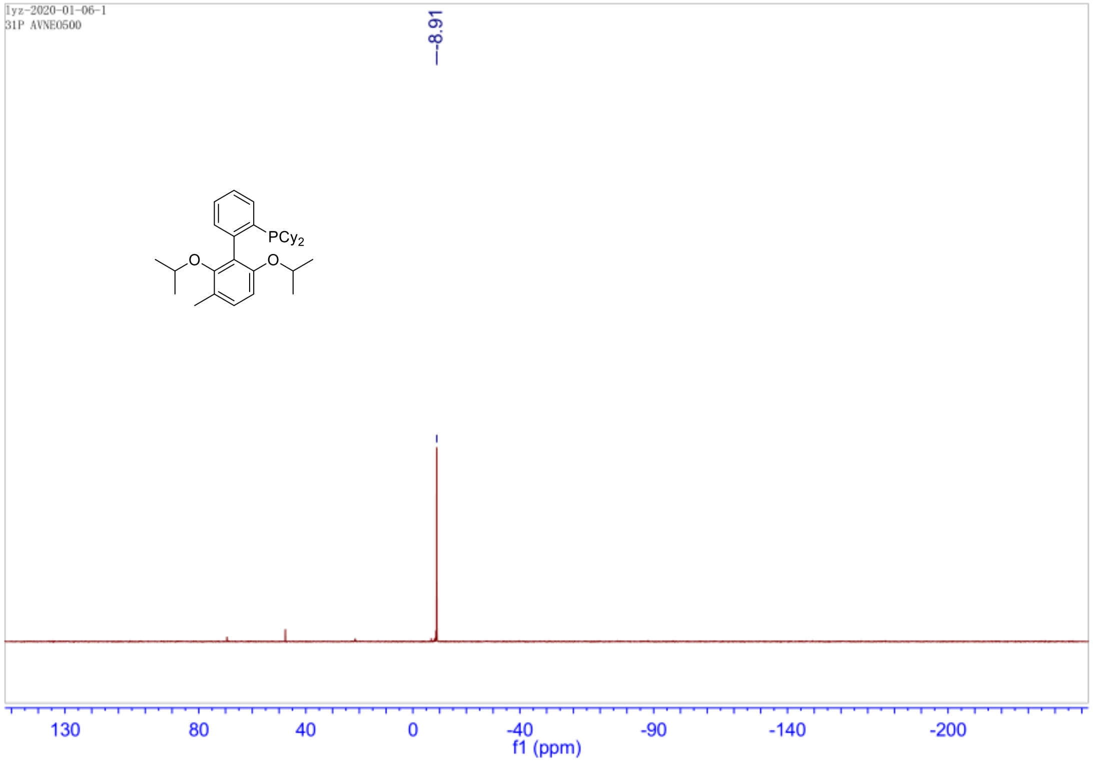

^{1}H NMR (500 MHz, $CDCl_3$)

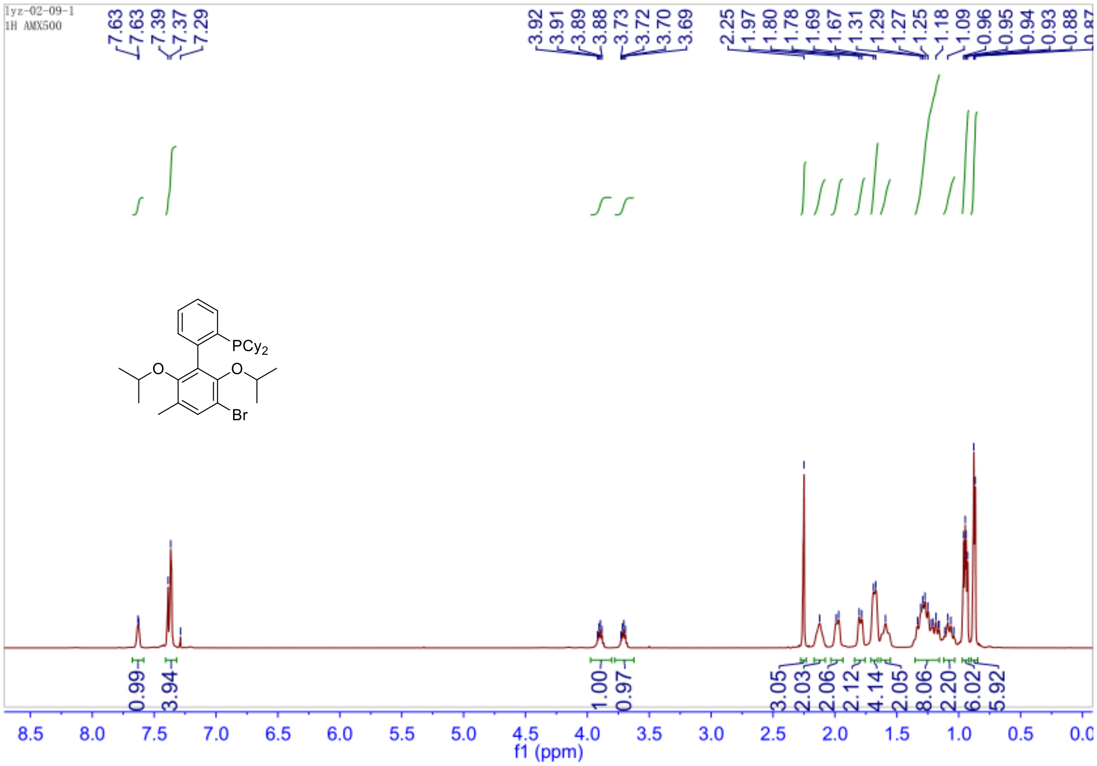

^{13}C NMR (126 MHz, $CDCl_3$)

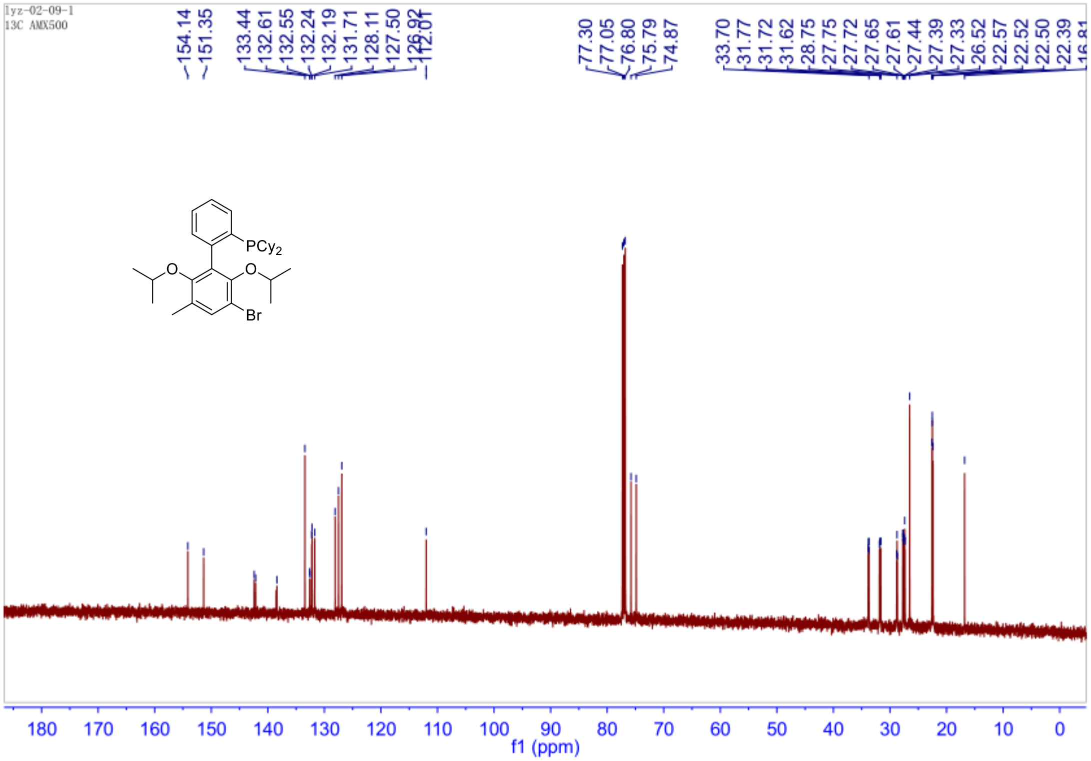

^{31}P NMR (202 MHz, $CDCl_3$)

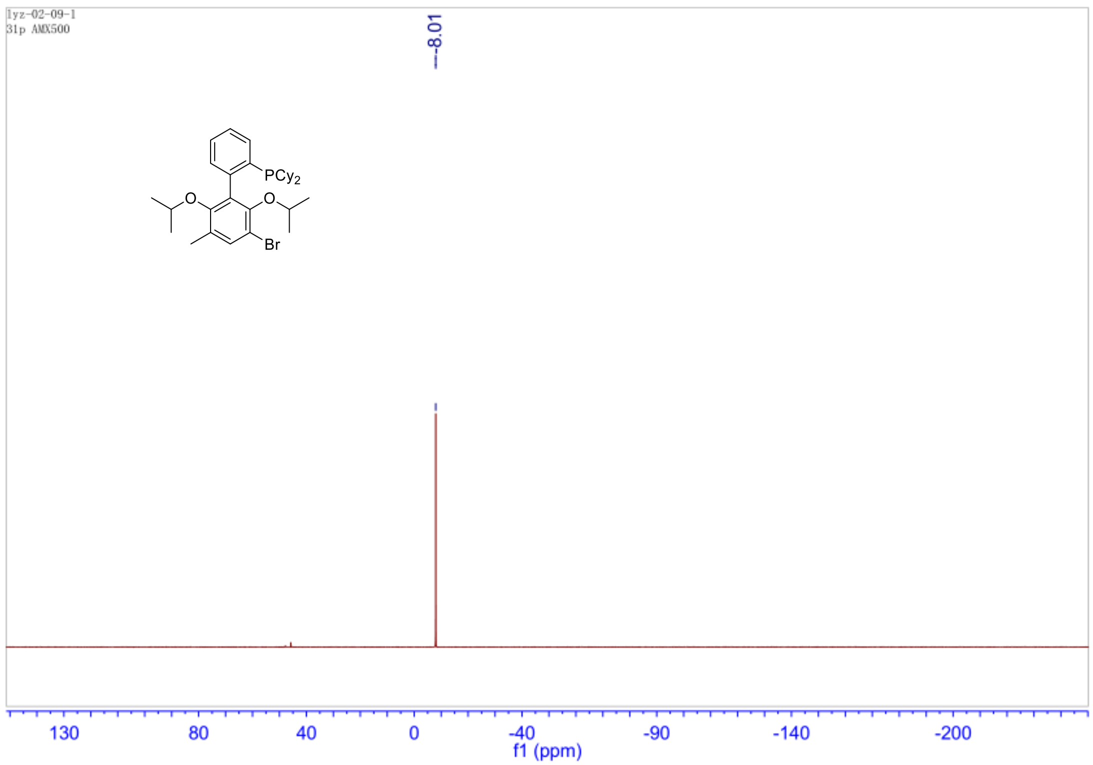

(*R, S_S*)-**L2**: ^{1}H NMR (500 MHz, $CDCl_3$)

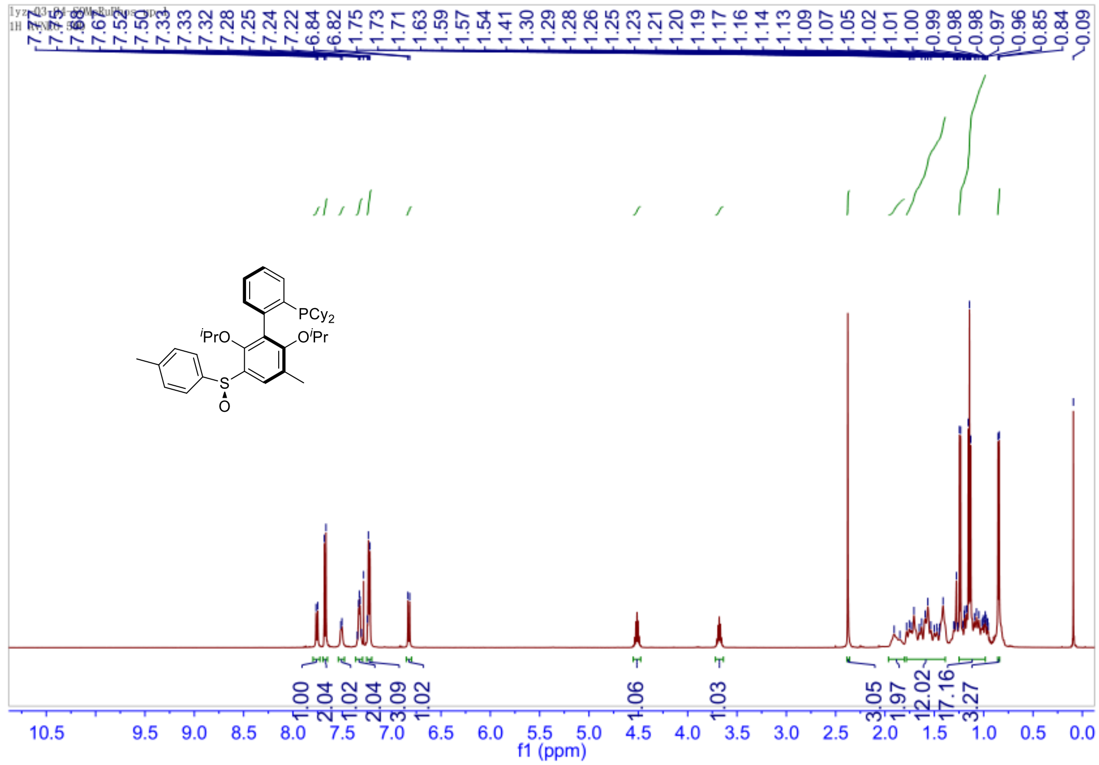

^{13}C NMR (126 MHz, $CDCl_3$)

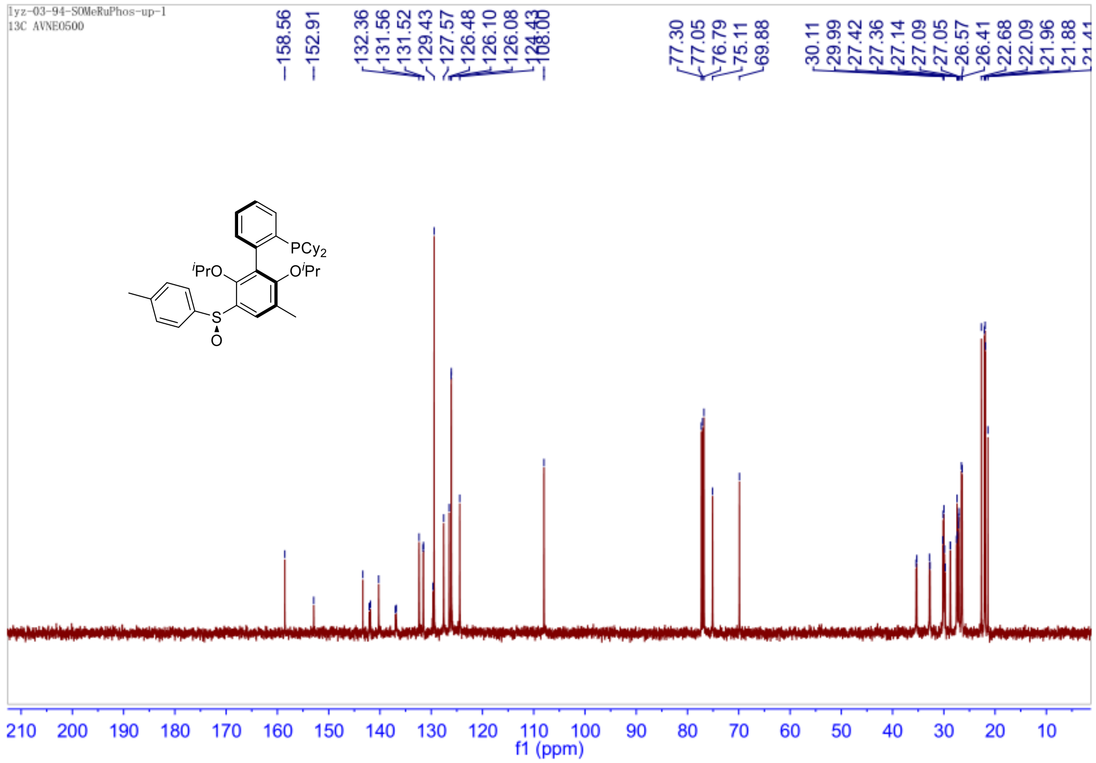

^{31}P NMR (202 MHz, $CDCl_3$)

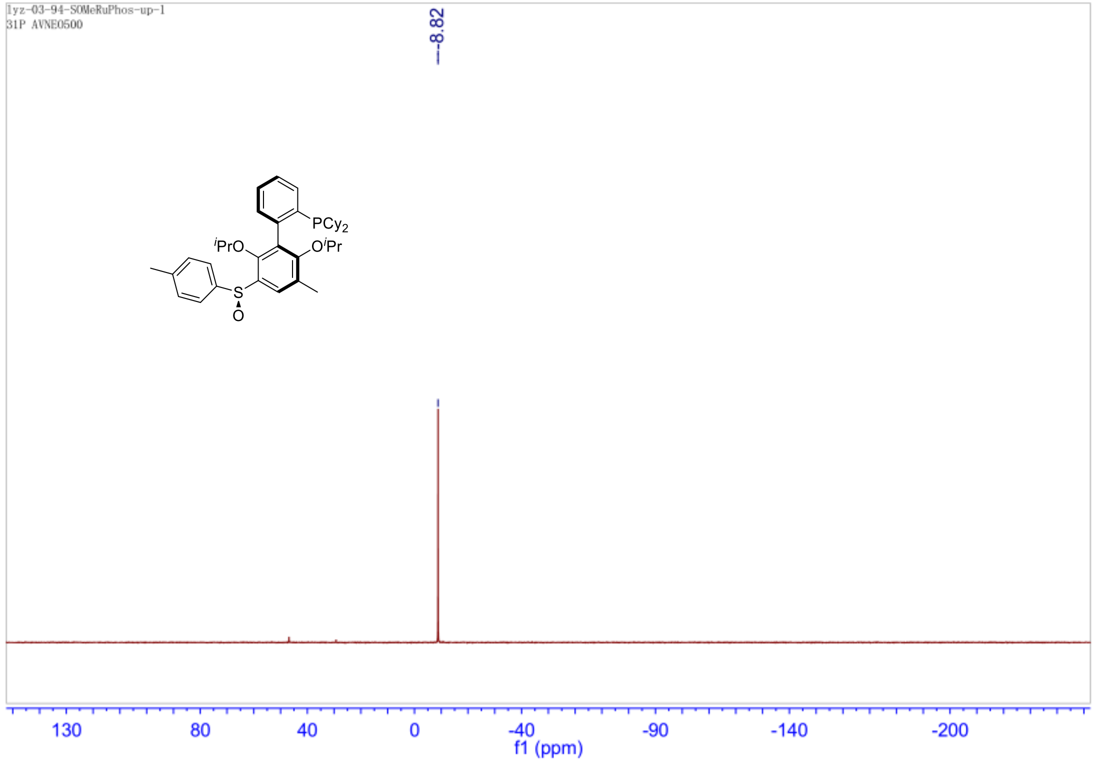

(*S*, S_S)-**L2**: ^{1}H NMR (500 MHz, CDCl$_3$)

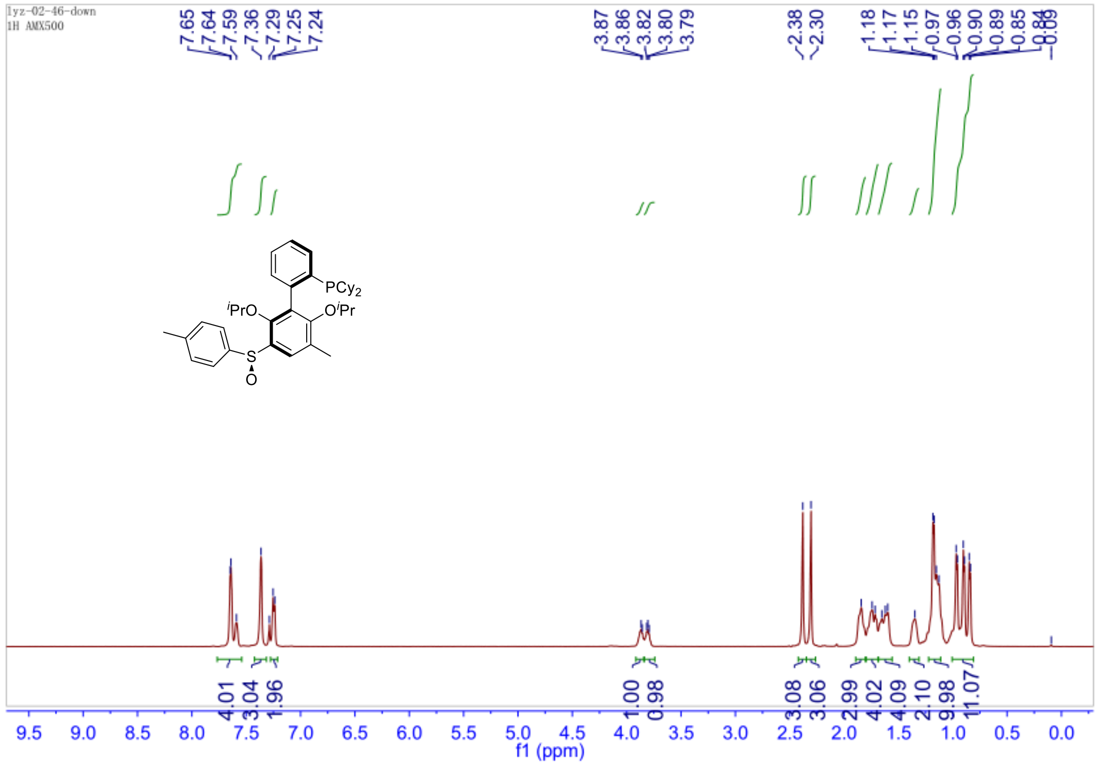

^{13}C NMR (126 MHz, CDCl$_3$)

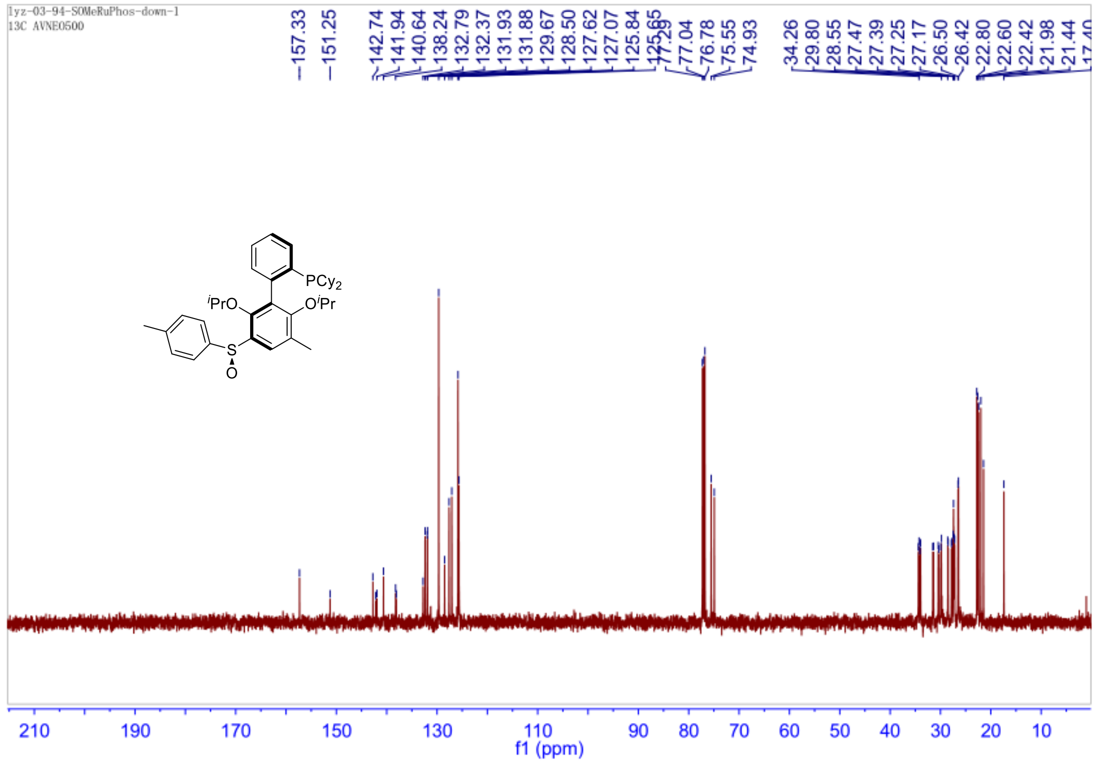

^{31}P NMR (202 MHz, $CDCl_3$)

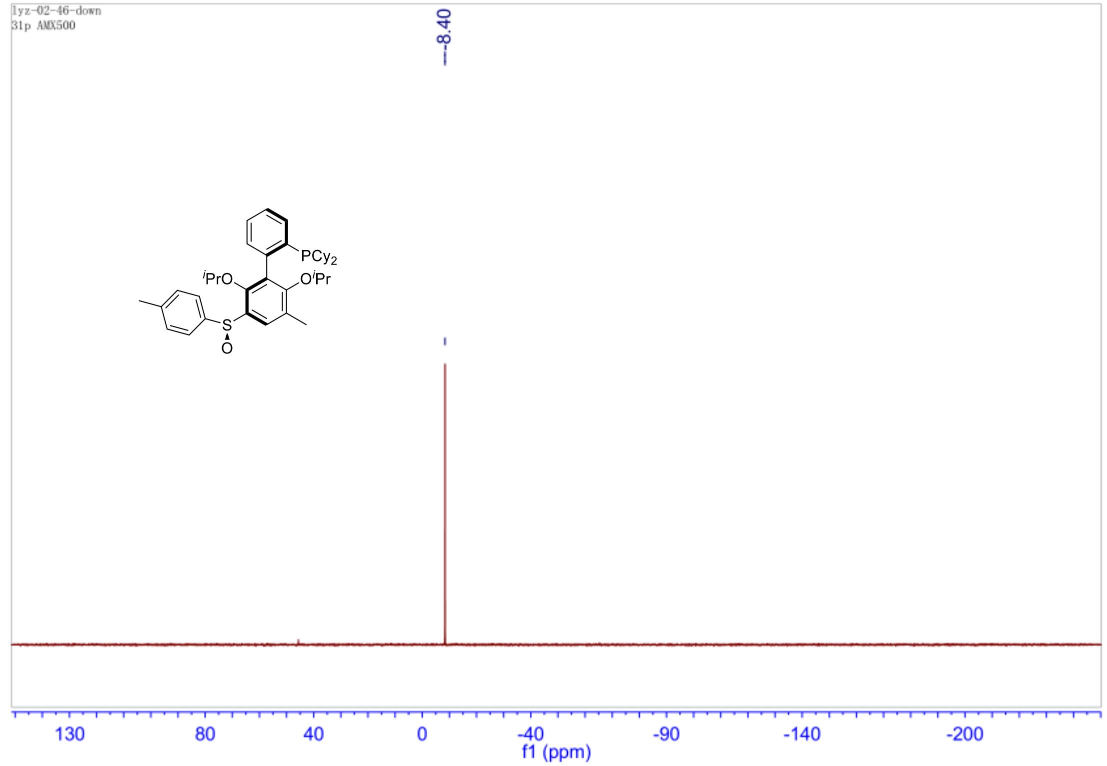

^{1}H NMR (500 MHz, $CDCl_3$)

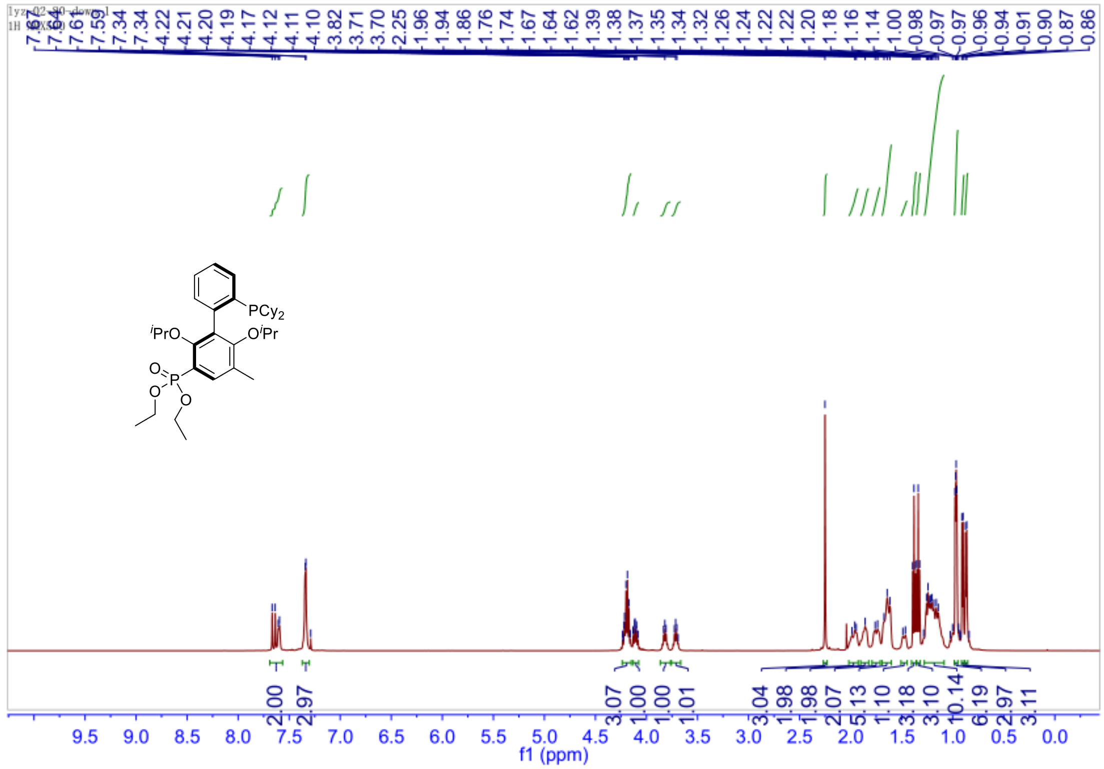

^{13}C NMR (126 MHz, $CDCl_3$)

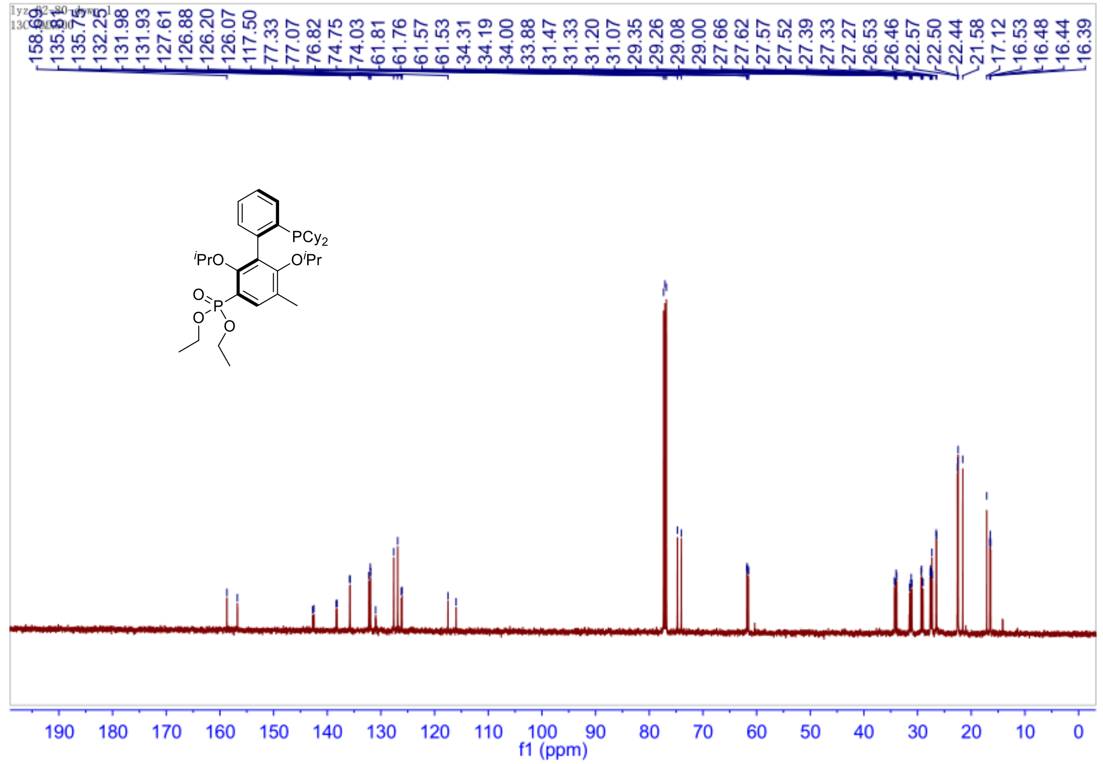

^{31}P NMR (202 MHz, $CDCl_3$)

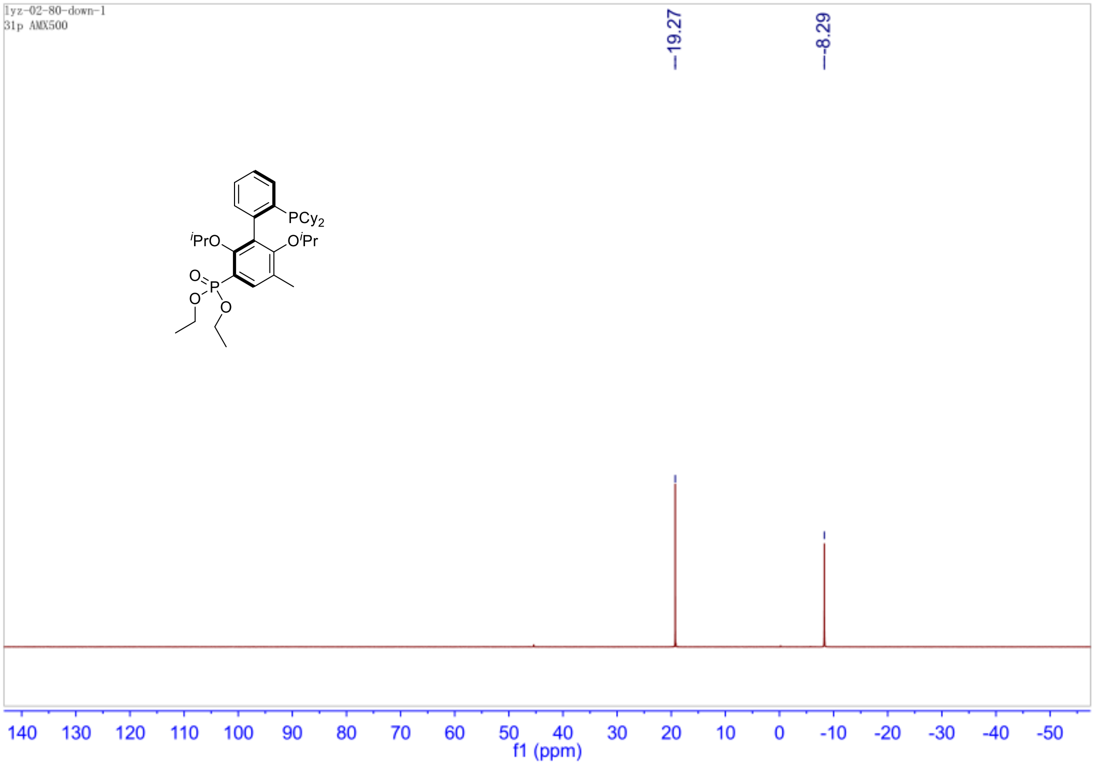

^{1}H NMR (500 MHz, $CDCl_3$) of (*S*)-**L4**

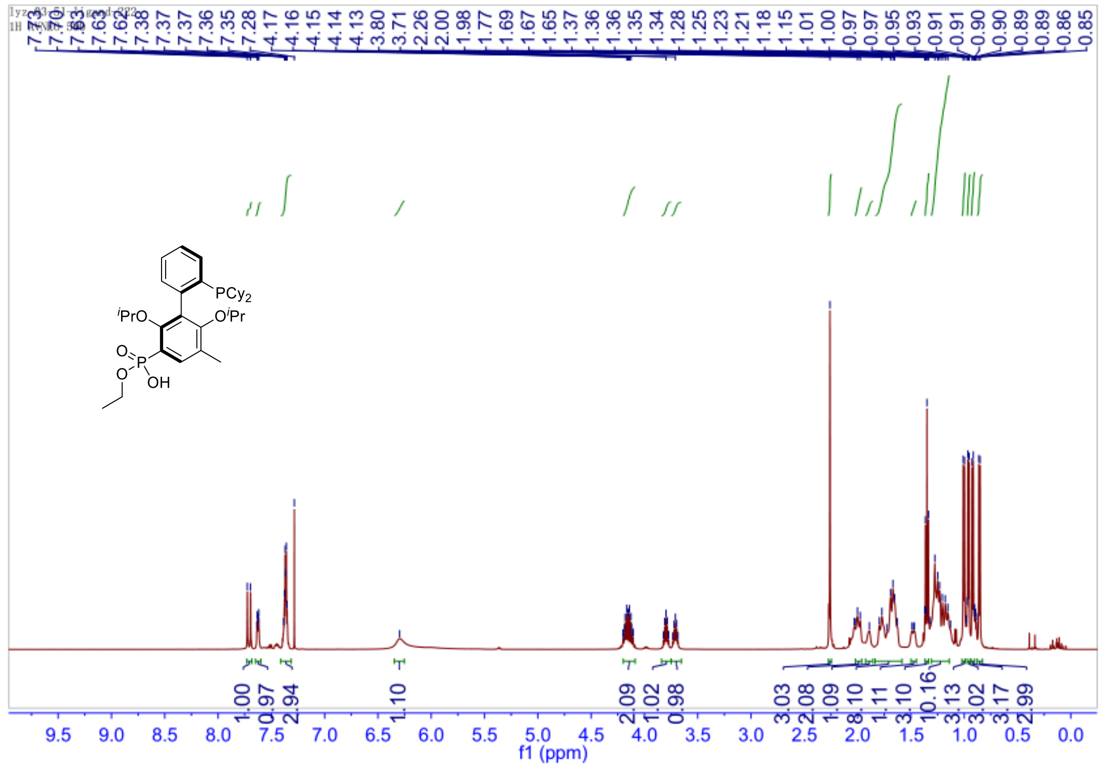

^{13}C NMR (126 MHz, $CDCl_3$) of (*S*)-**L4**

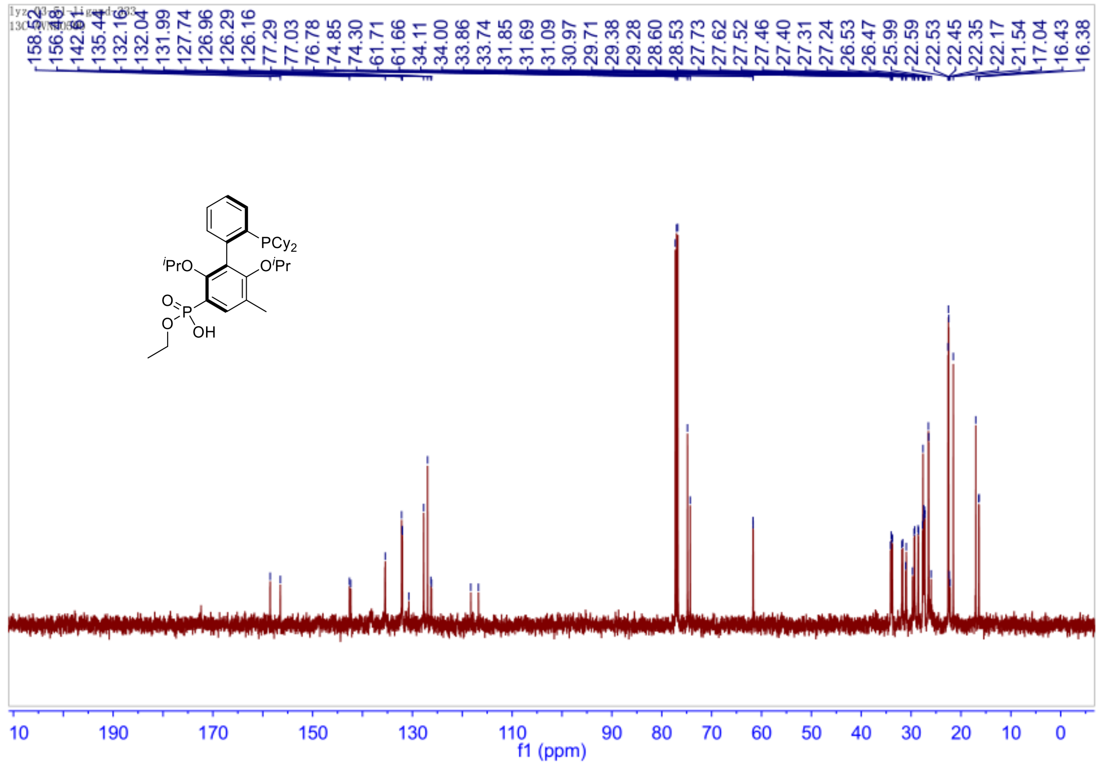

^{31}P NMR (202 MHz, $CDCl_3$) of (*S*)-**L4**

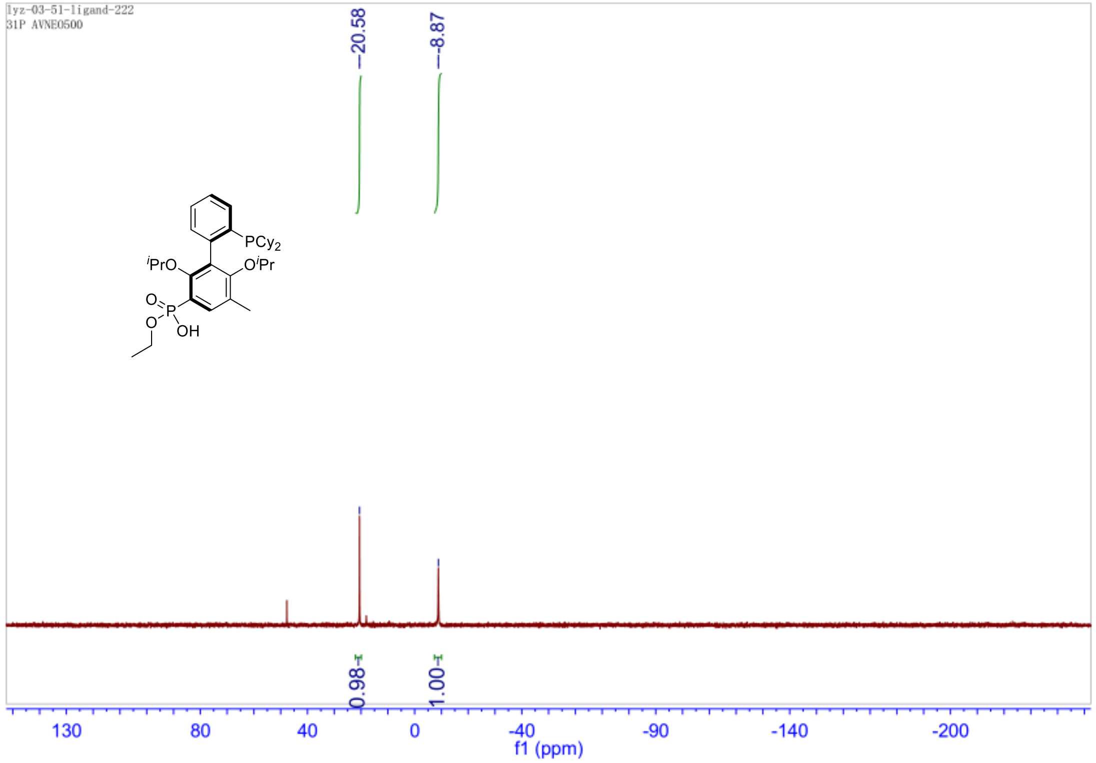

^{1}H NMR (500 MHz, $CDCl_3$)

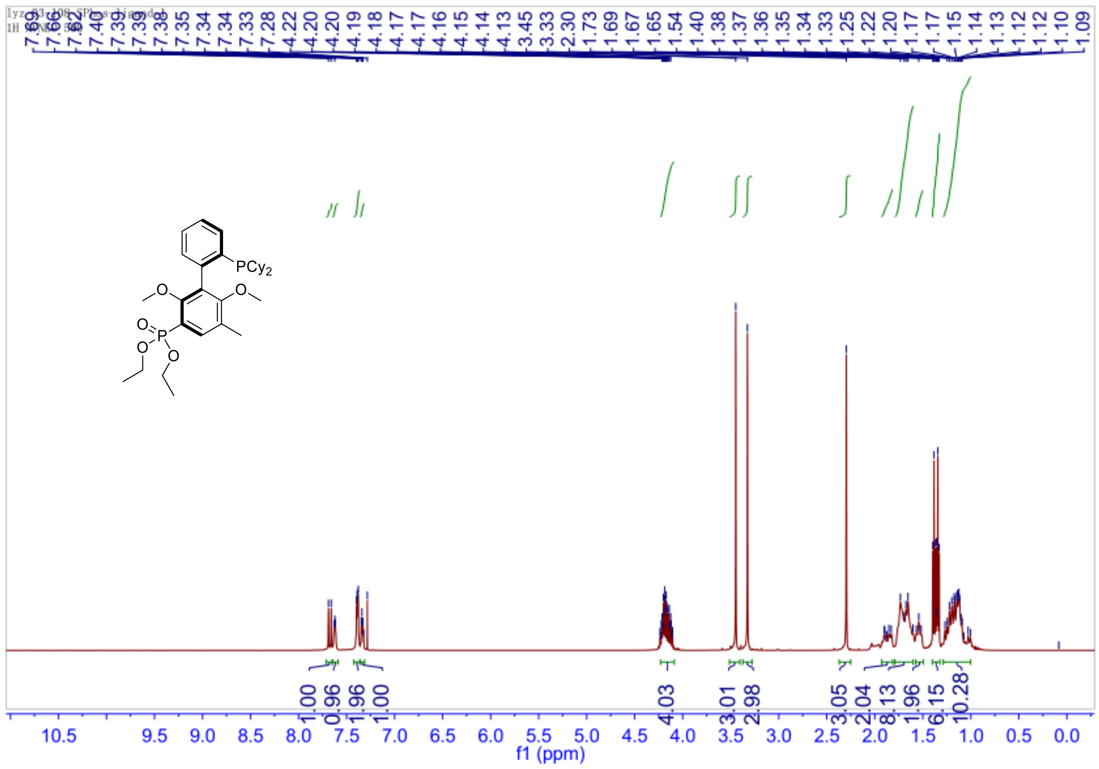

^{13}C NMR (126 MHz, $CDCl_3$)

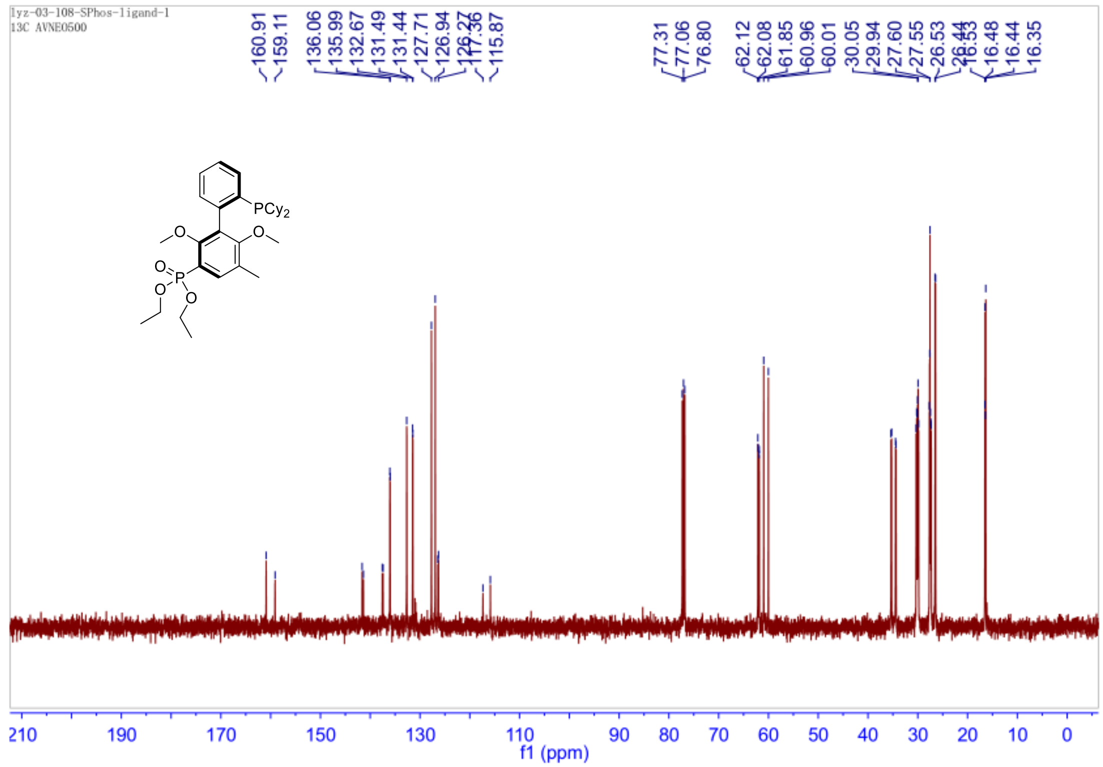

^{31}P NMR (202 MHz, $CDCl_3$)

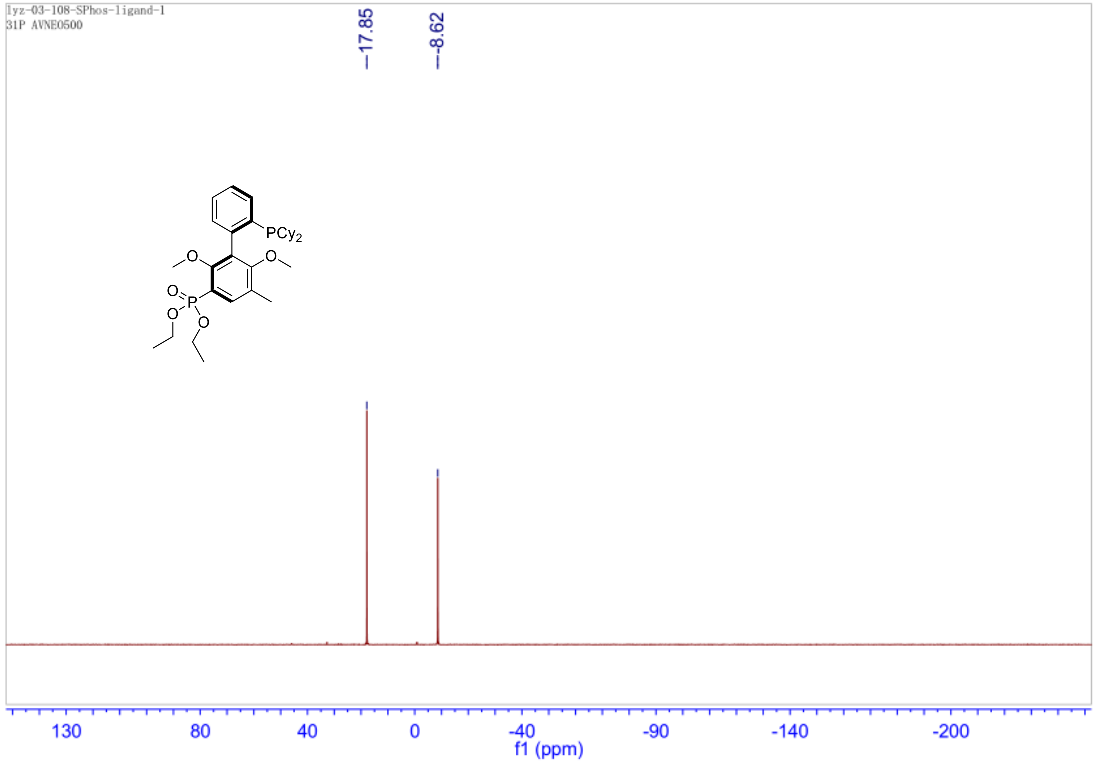

^{1}H NMR (500 MHz, $CDCl_3$) of (*S*)-**L5**

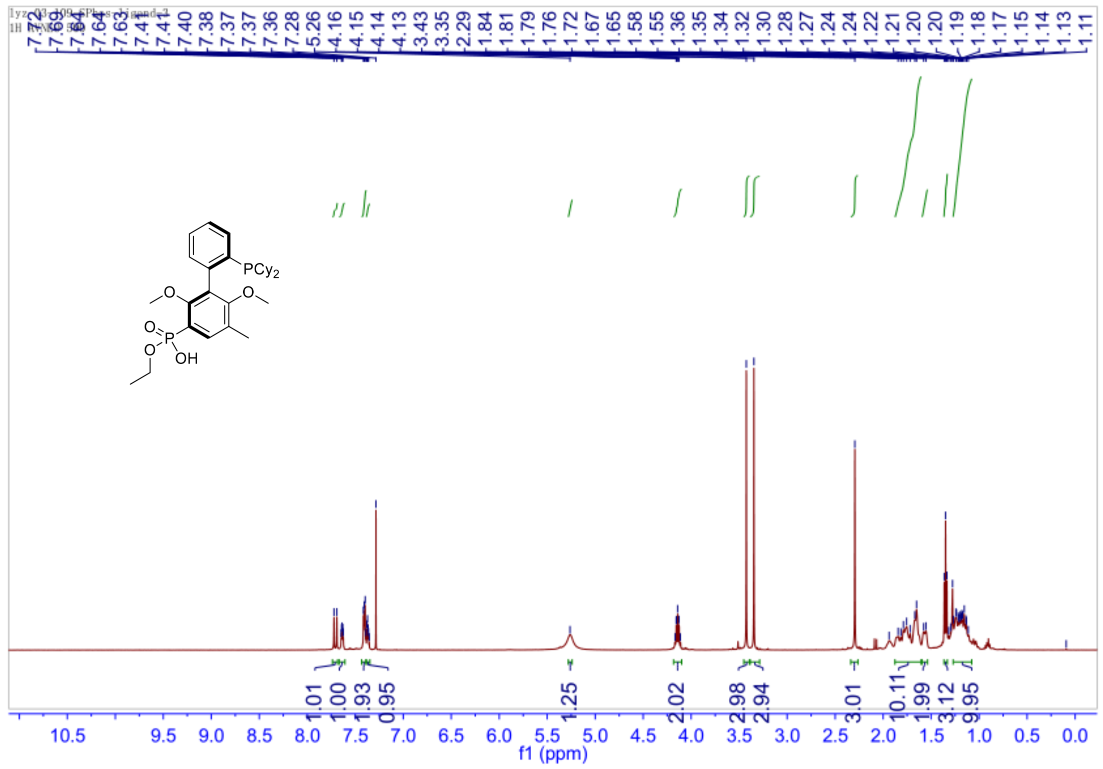

^{13}C NMR (126 MHz, $CDCl_3$) of (*S*)- **L5**

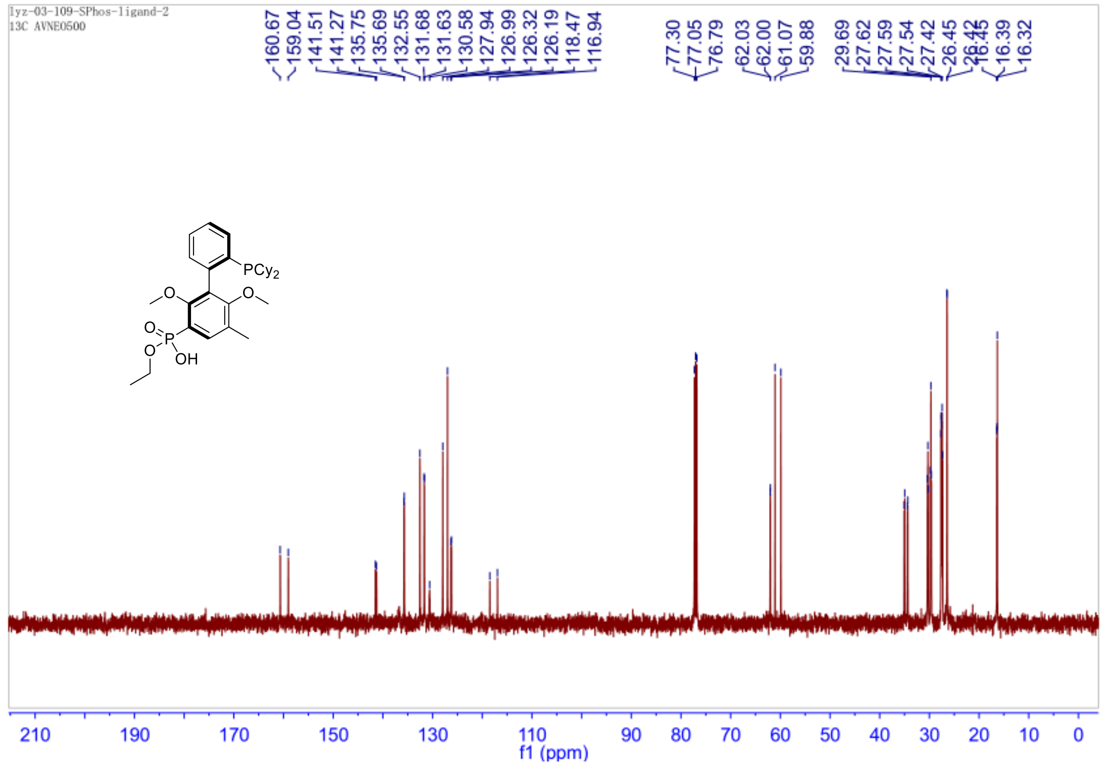

^{31}P NMR (202 MHz, $CDCl_3$) of (*S*)-**L5**

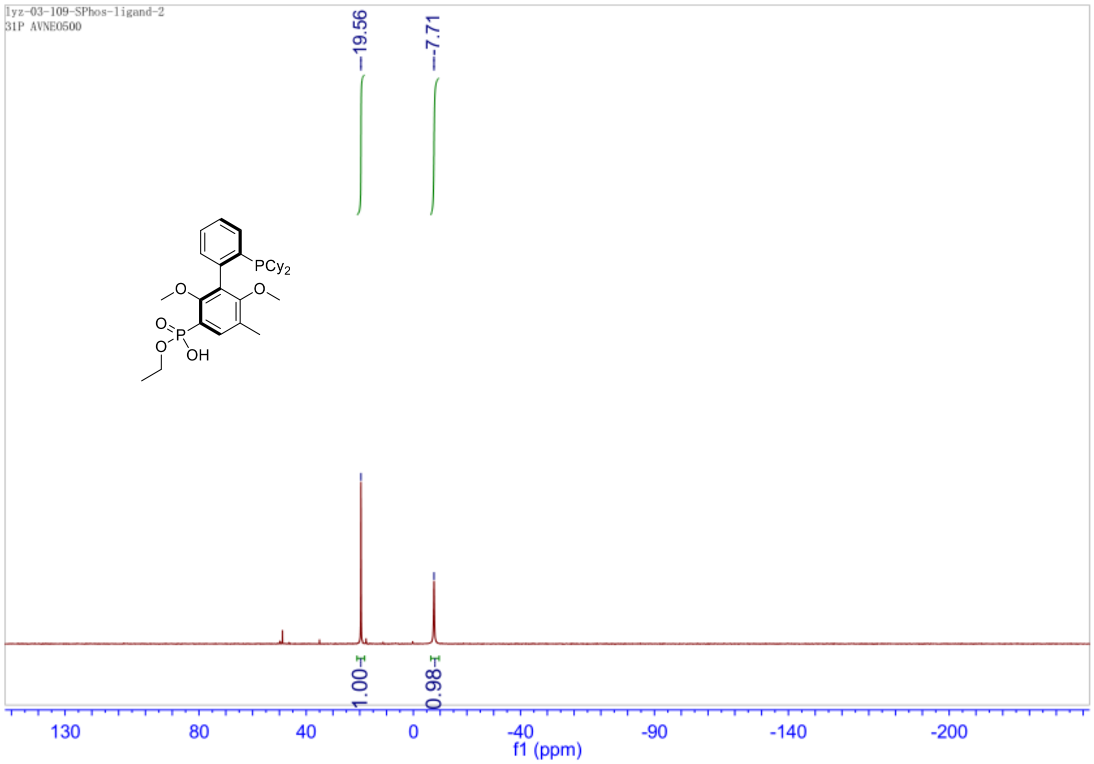

^{1}H NMR (500 MHz, $CDCl_3$)

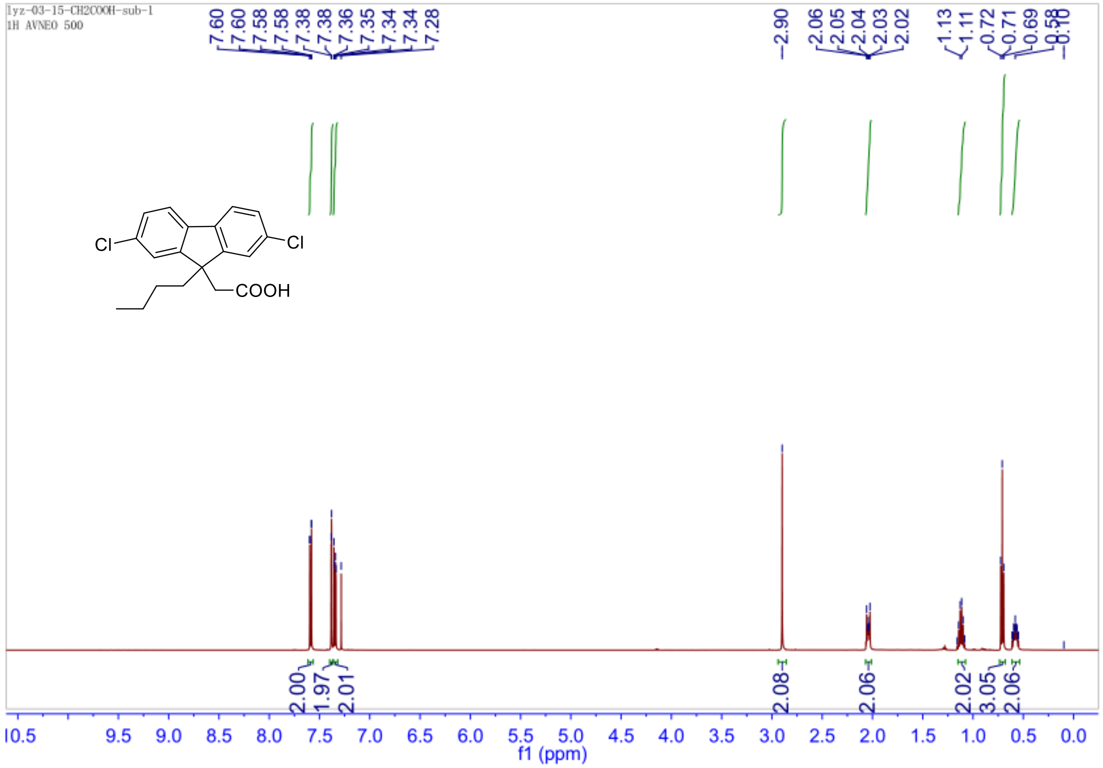

^{13}C NMR (126 MHz, $CDCl_3$)

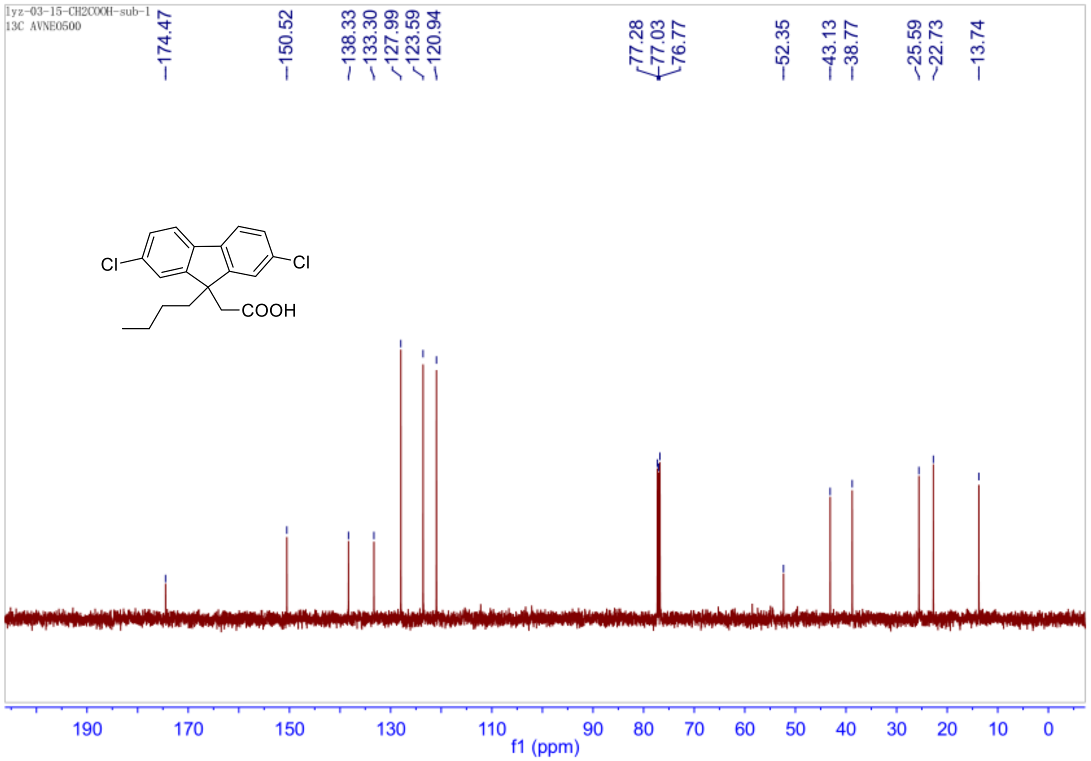

^{1}H NMR (300 MHz, $CDCl_3$)

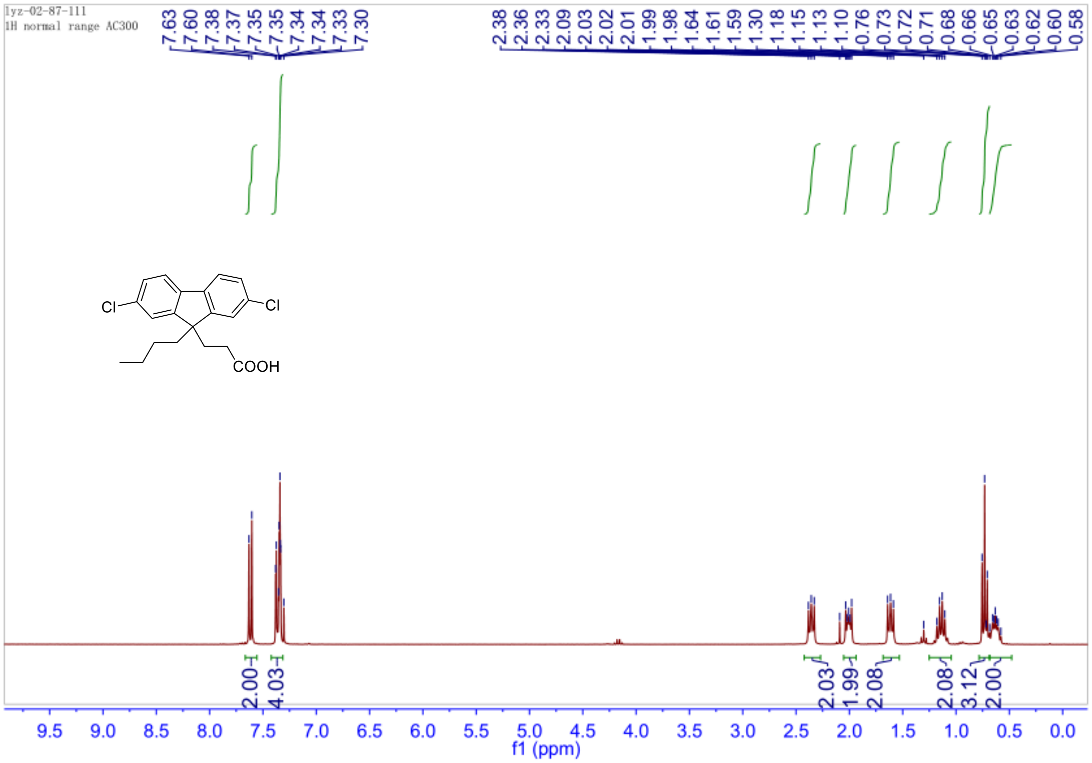

^{13}C NMR (75 MHz, $CDCl_3$)

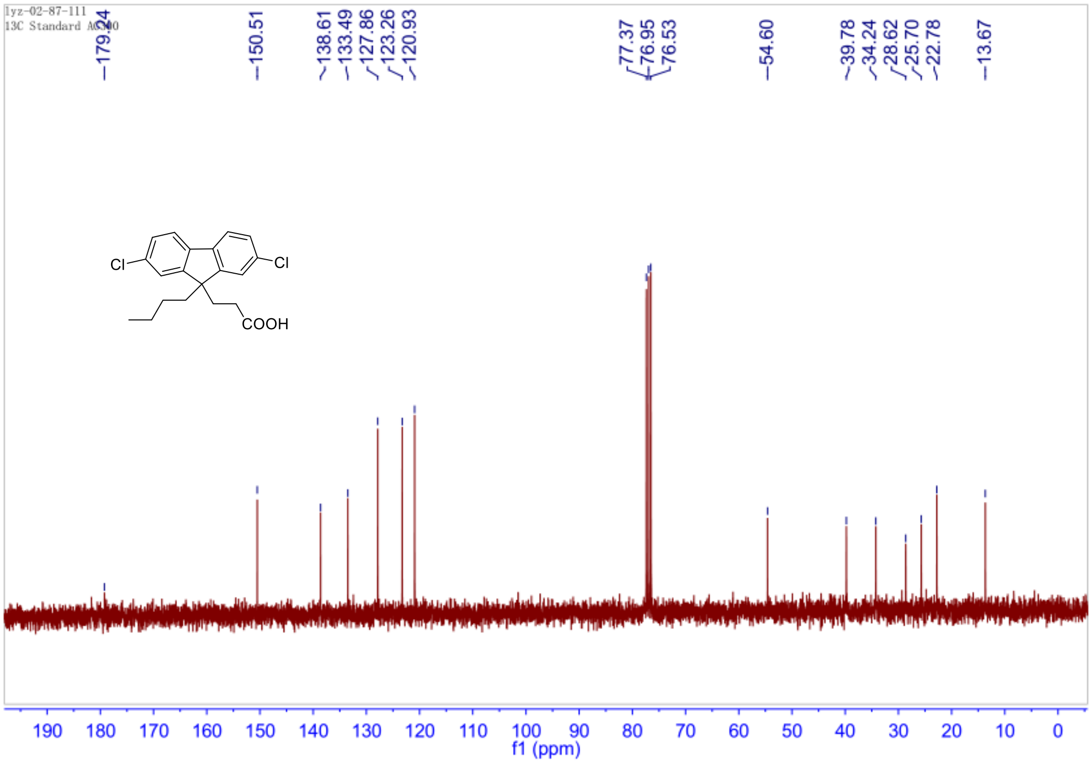

^{1}H NMR (500 MHz, $CDCl_3$)-**51**

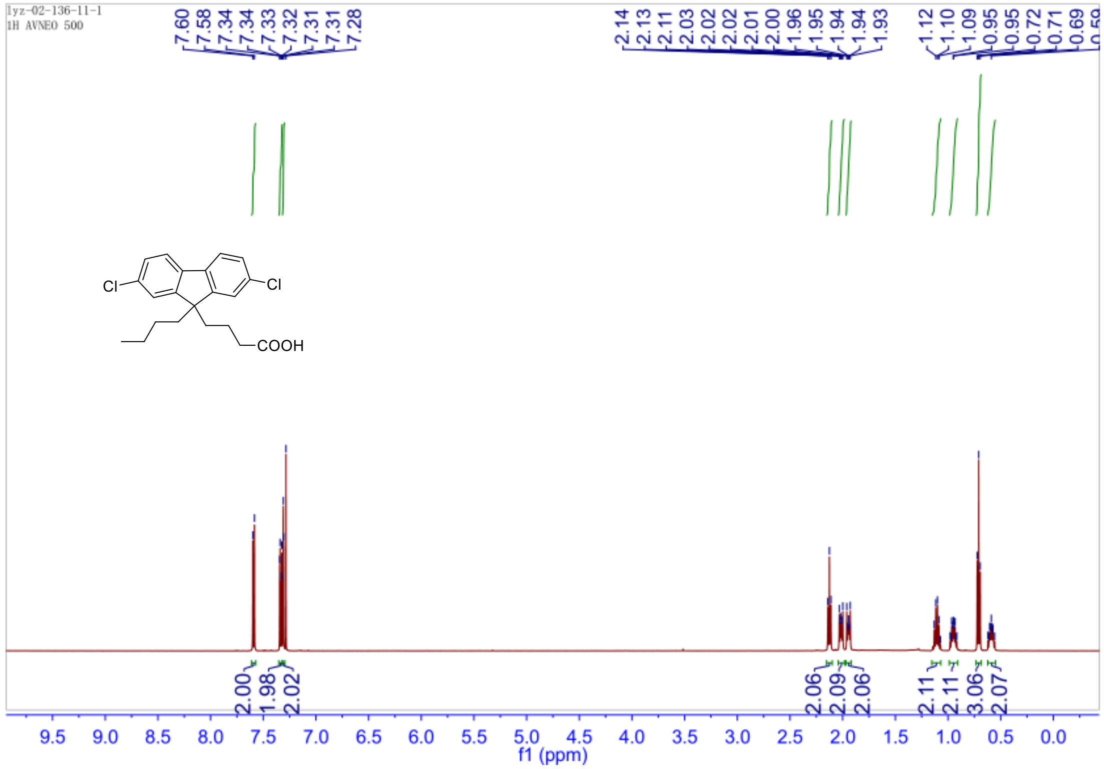

^{13}C NMR (126 MHz, $CDCl_3$)-**51**

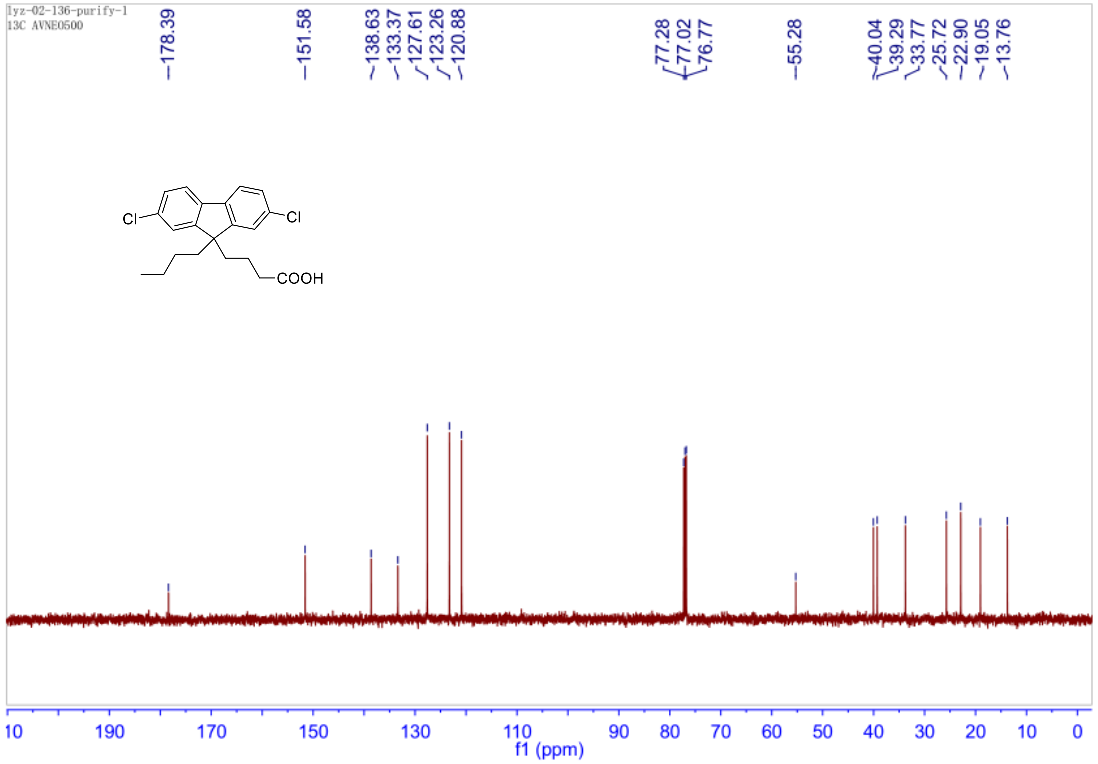

^{1}H NMR (500 MHz, $CDCl_3$)-**53**

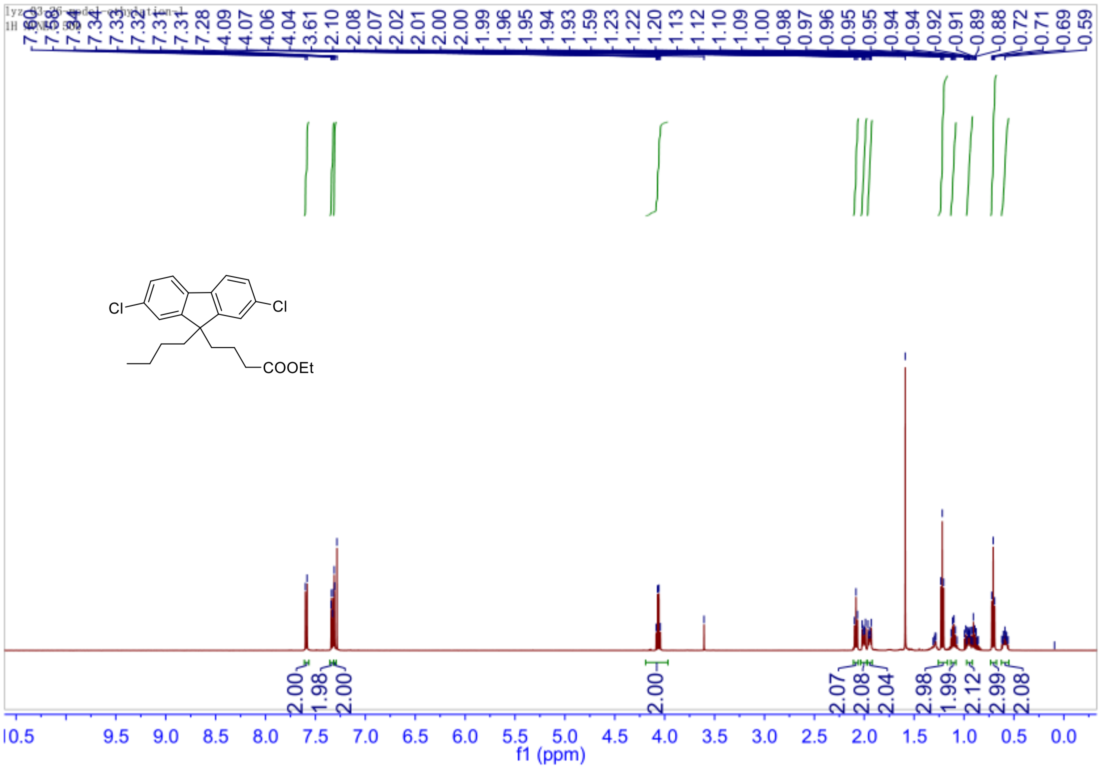

^{13}C NMR (126 MHz, $CDCl_3$)-**53**

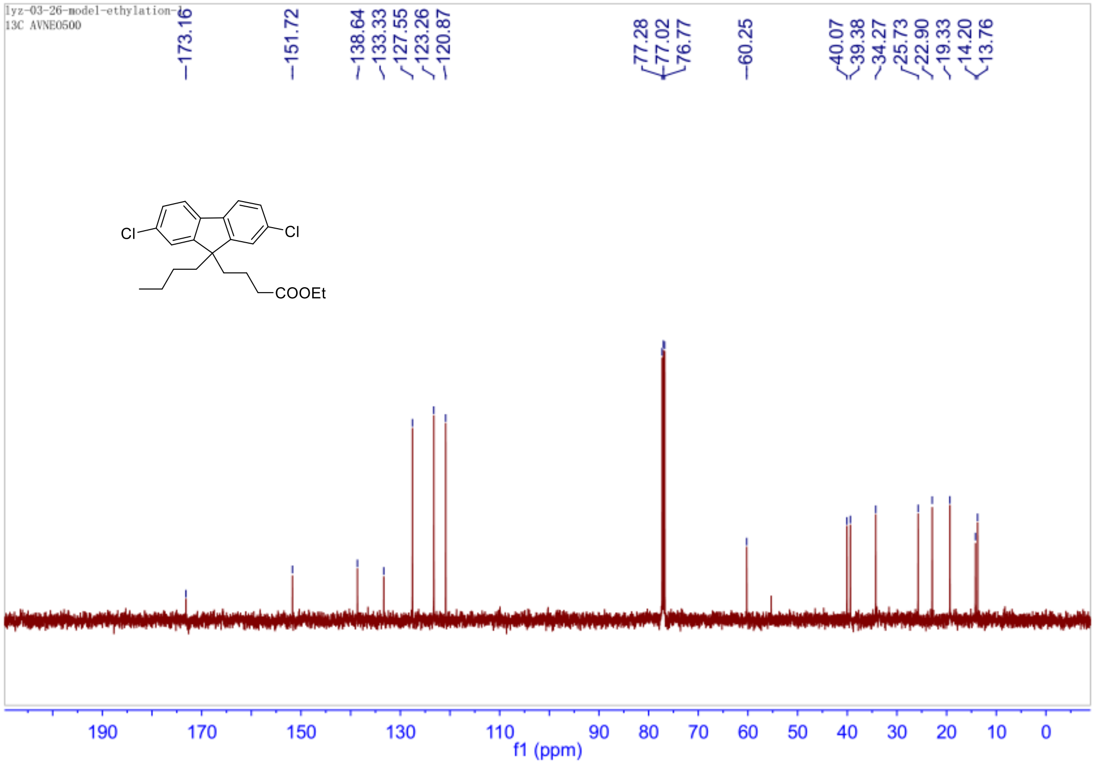

^{1}H NMR (500 MHz, $CDCl_3$)

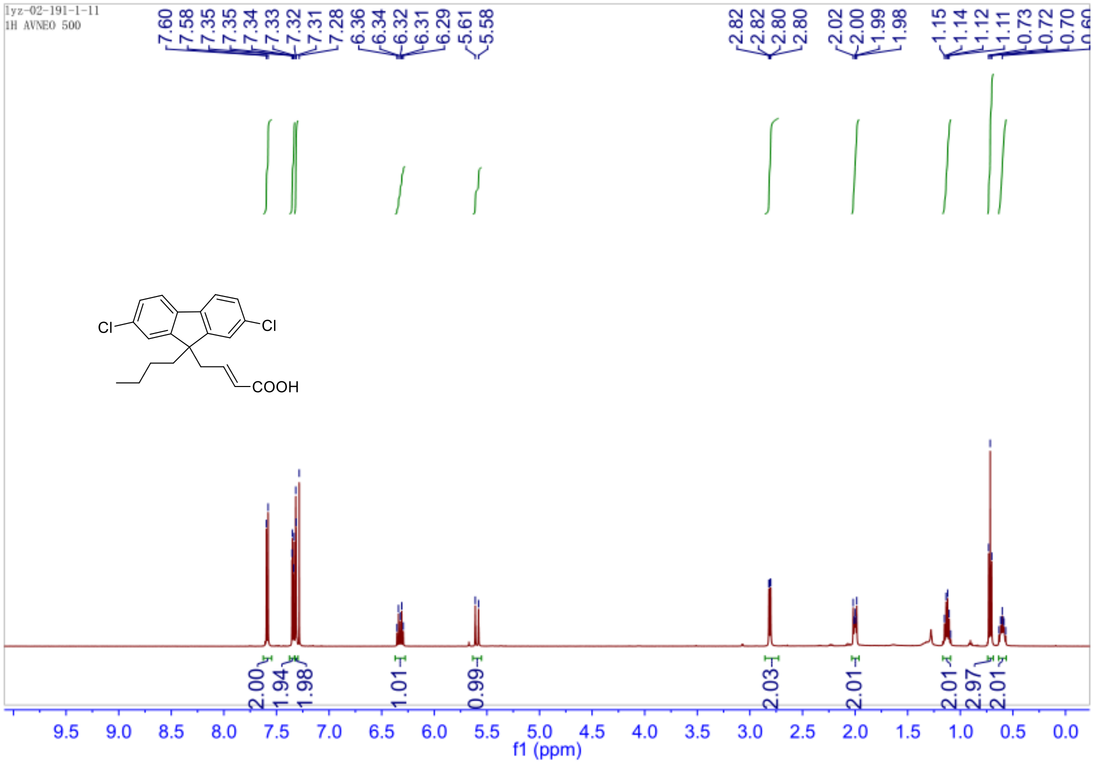

^{13}C NMR (126 MHz, $CDCl_3$)

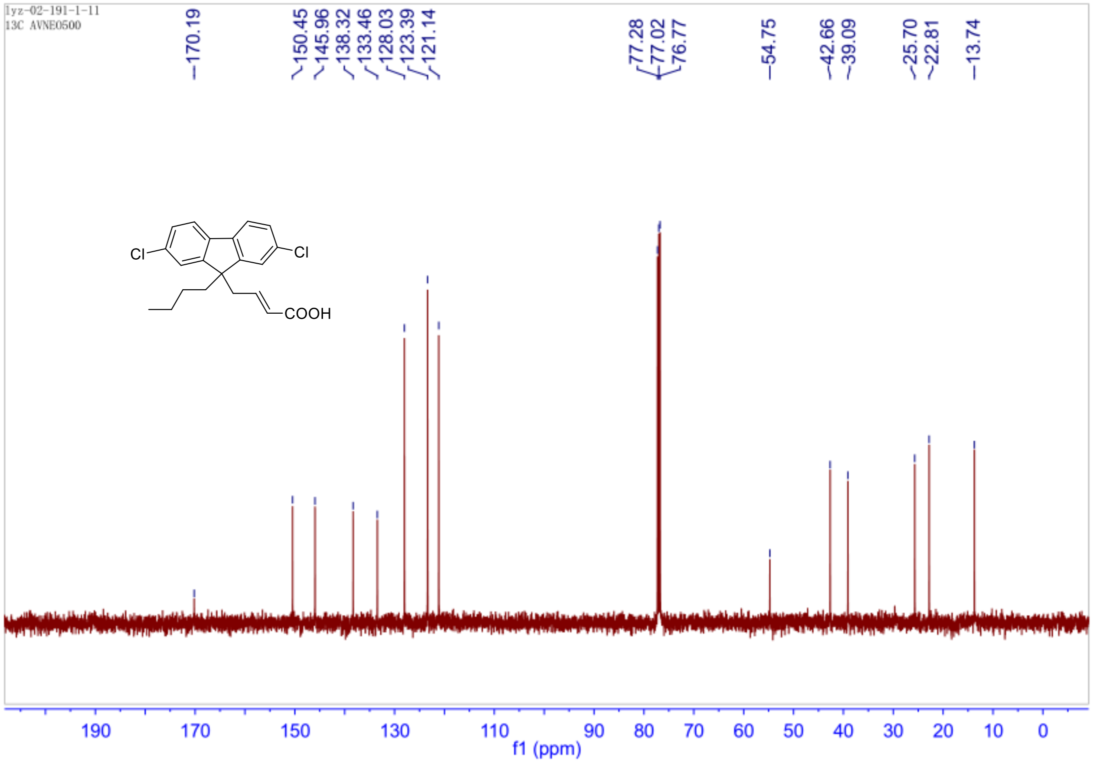

^{1}H NMR (500 MHz, $CDCl_3$)

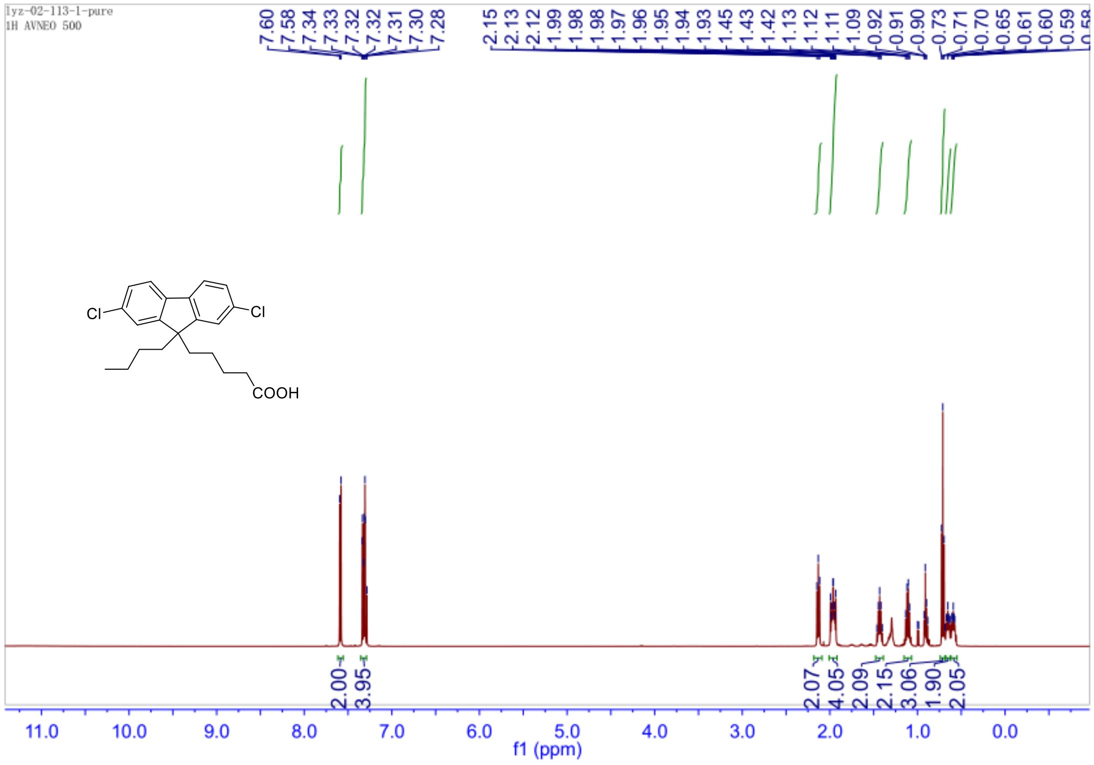

^{13}C NMR (126 MHz, $CDCl_3$)

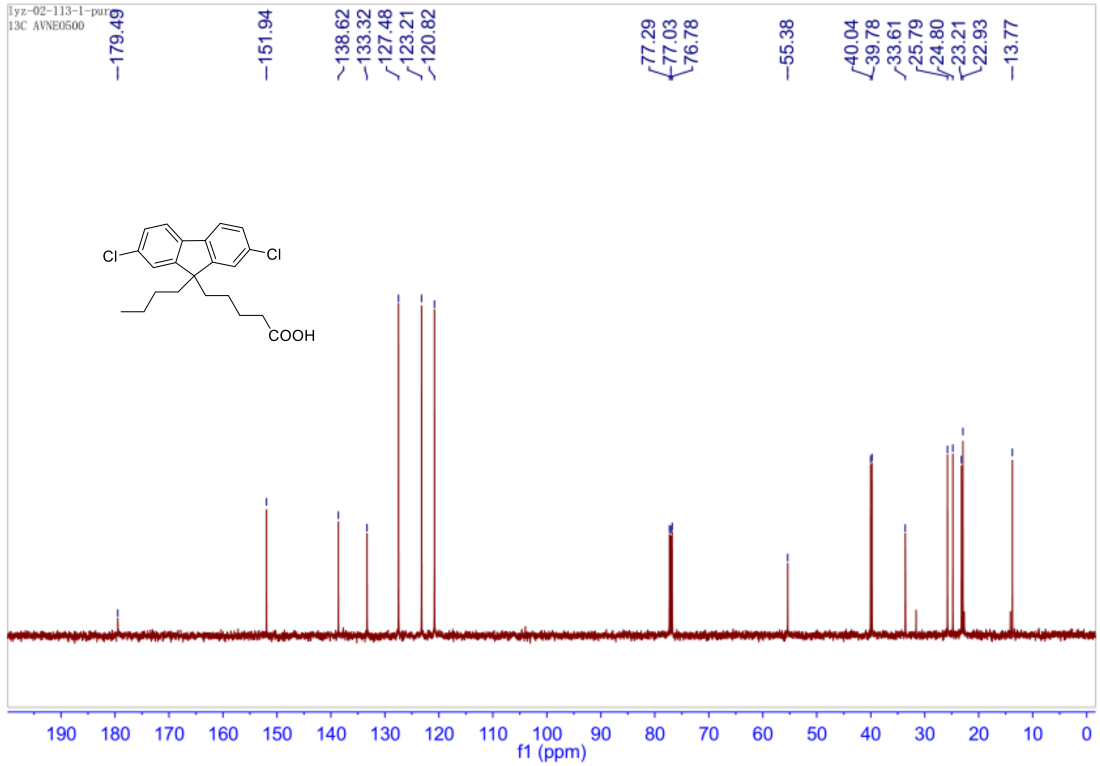

^{1}H NMR (300 MHz, $CDCl_3$)

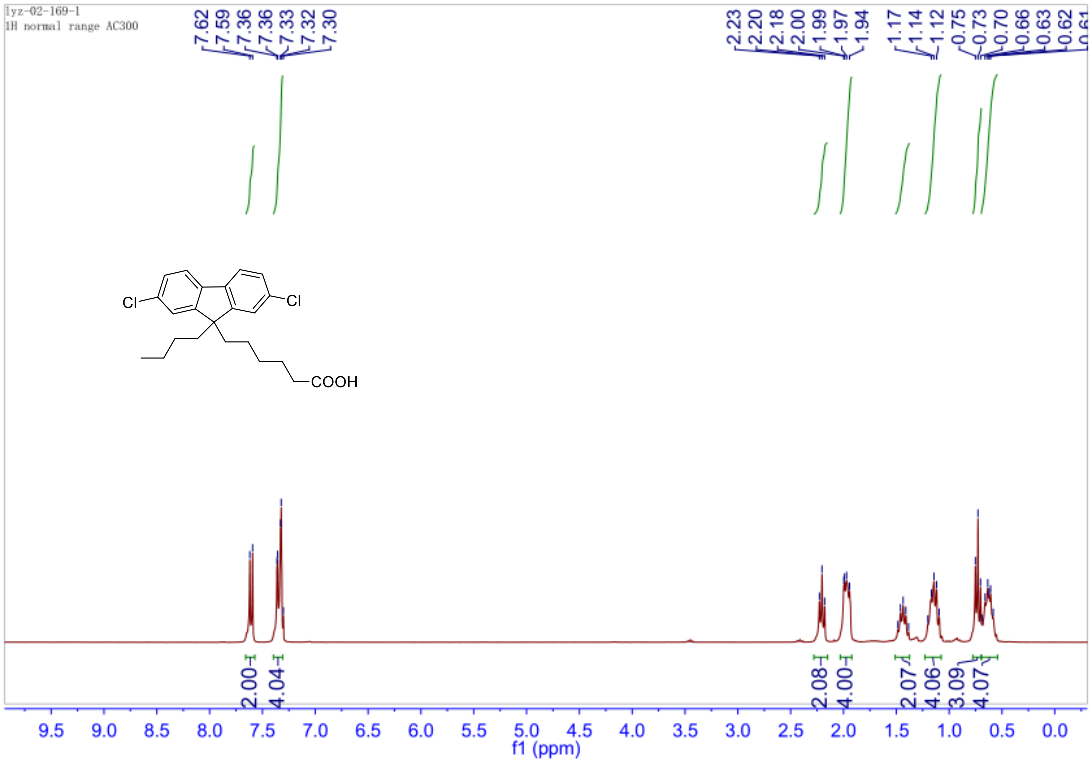

^{13}C NMR (75 MHz, $CDCl_3$)

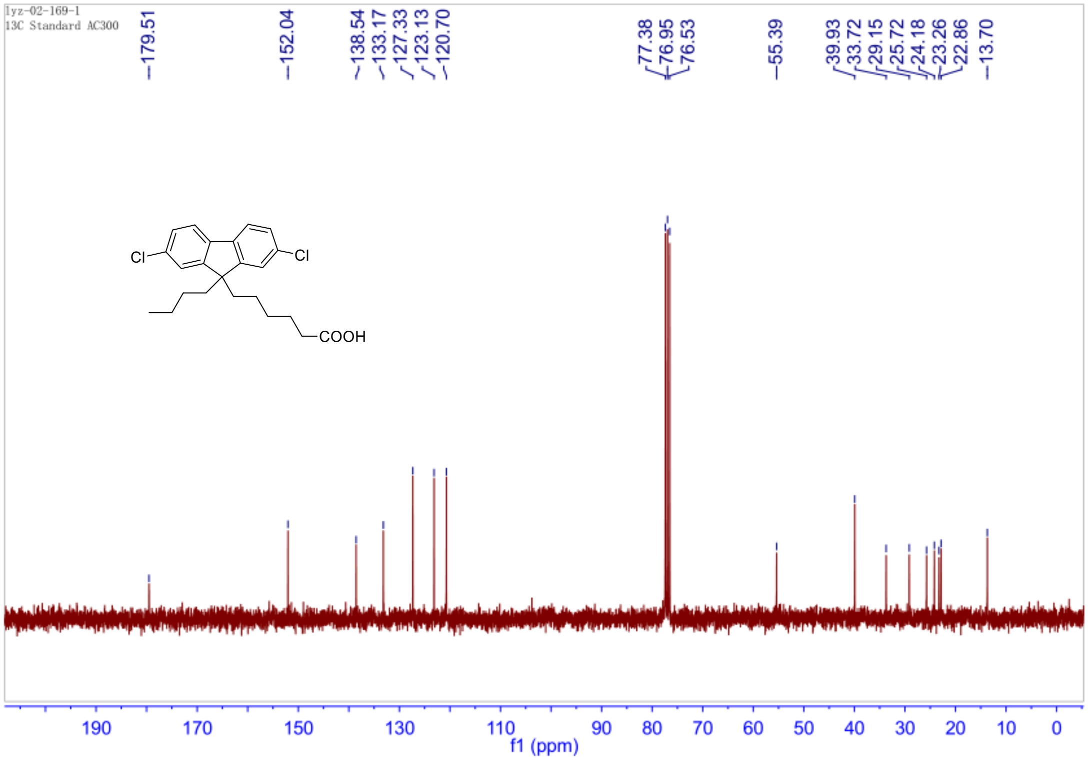

^{1}H NMR (500 MHz, $CDCl_3$)

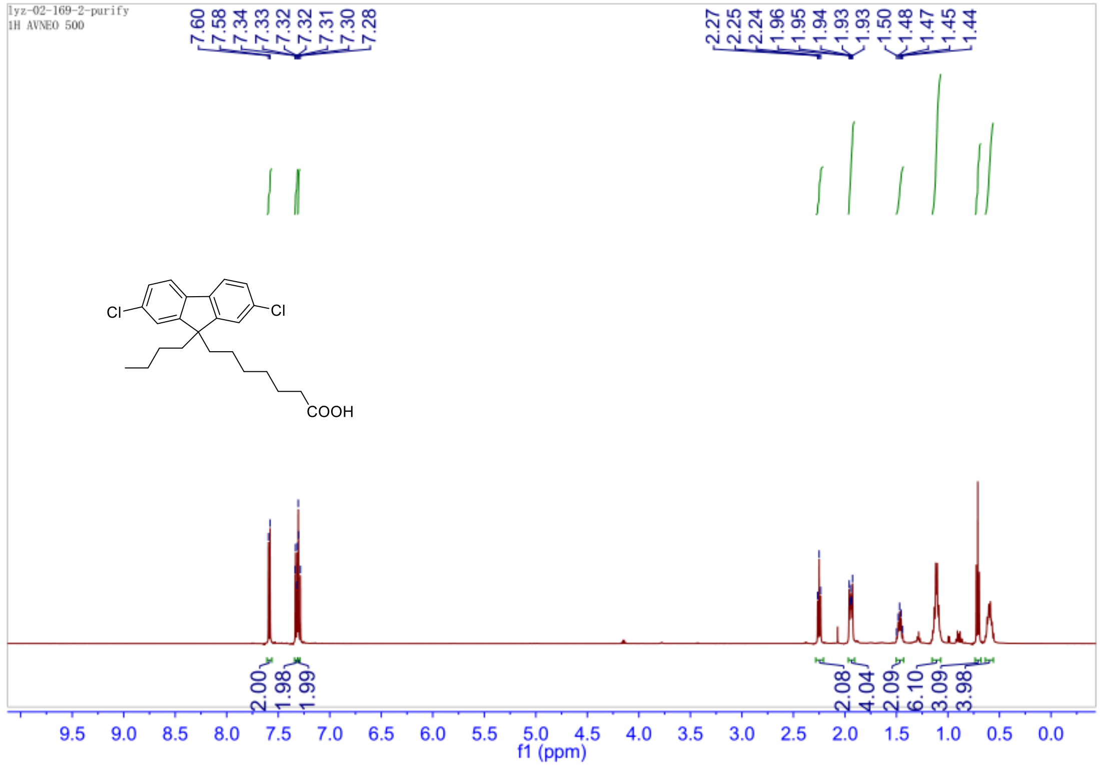

^{13}C NMR (126 MHz, $CDCl_3$)

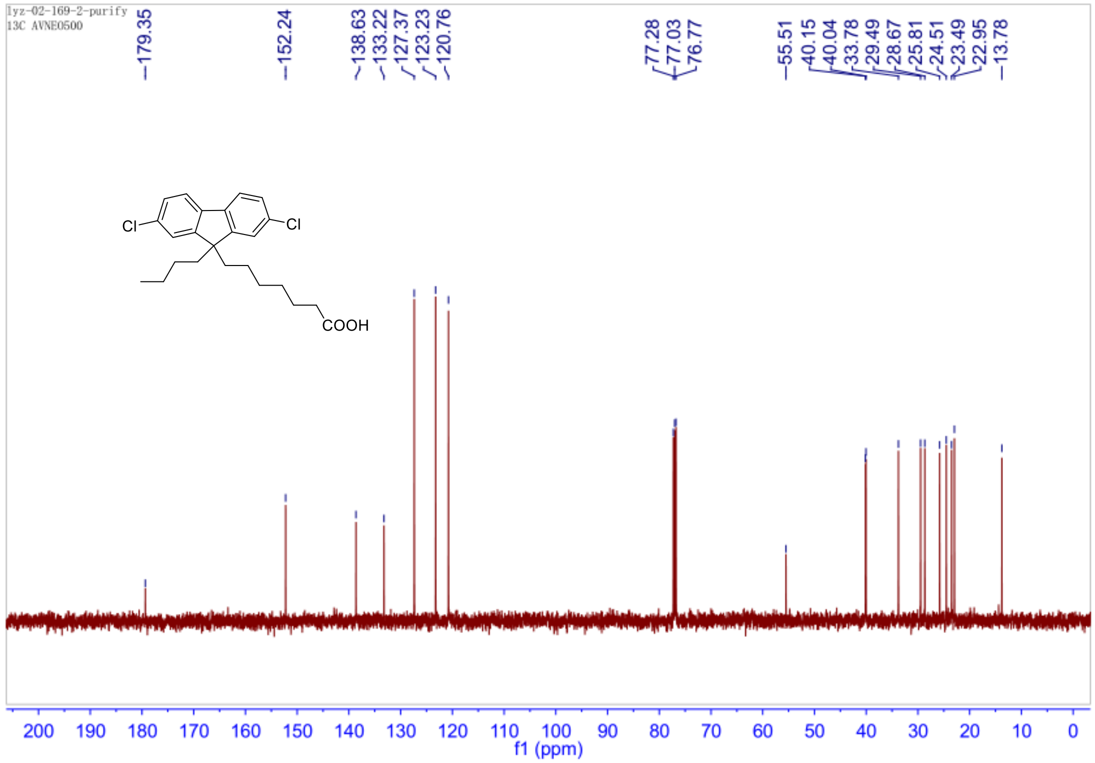

^1H NMR (500 MHz, $CDCl_3$)

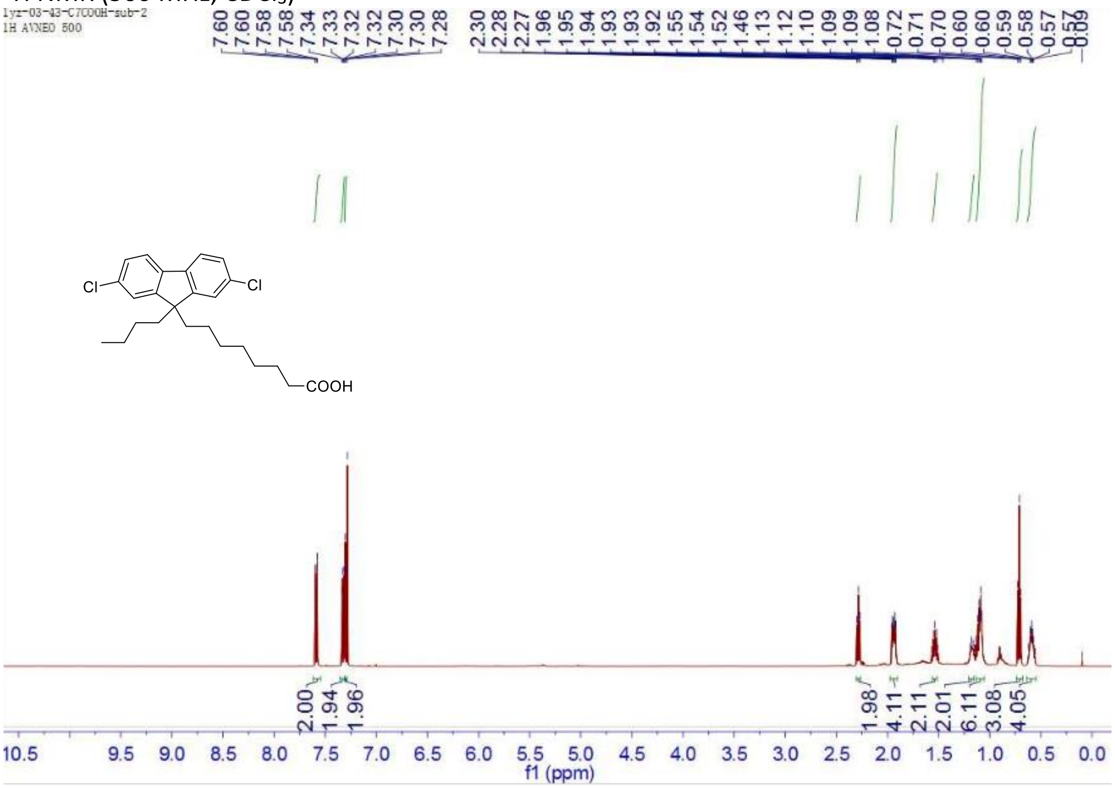

^{13}C NMR (126 MHz, $CDCl_3$)

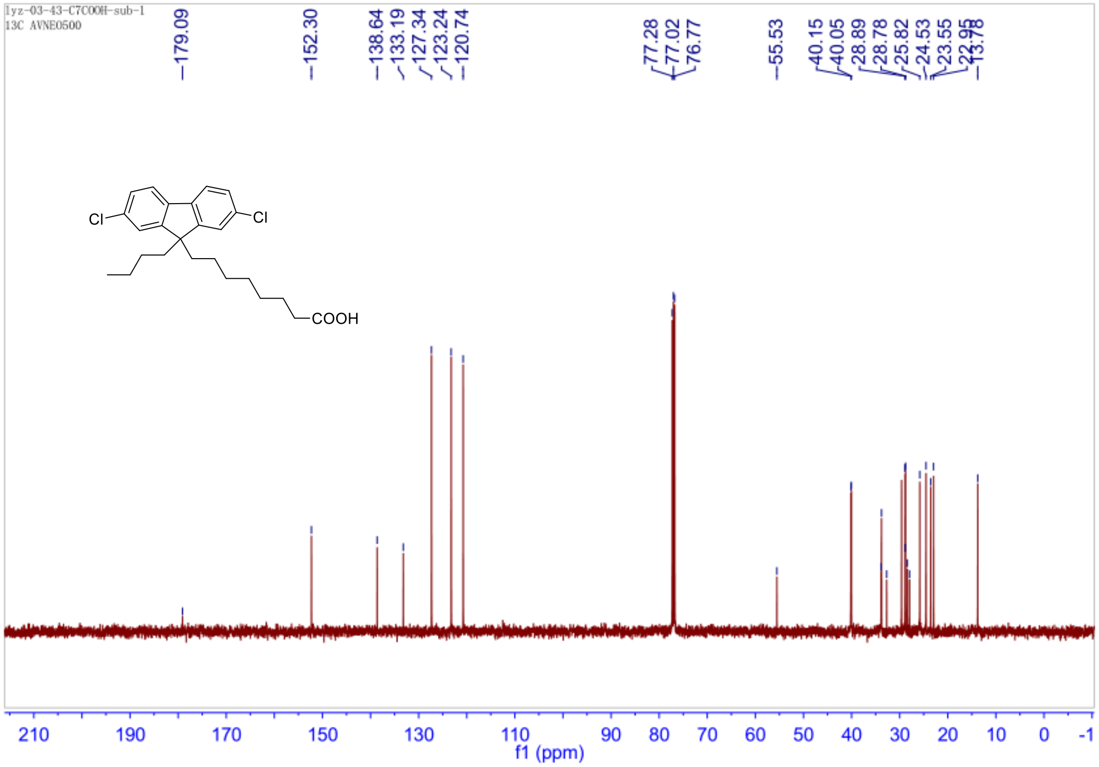

^{1}H NMR (500 MHz, $CDCl_3$)

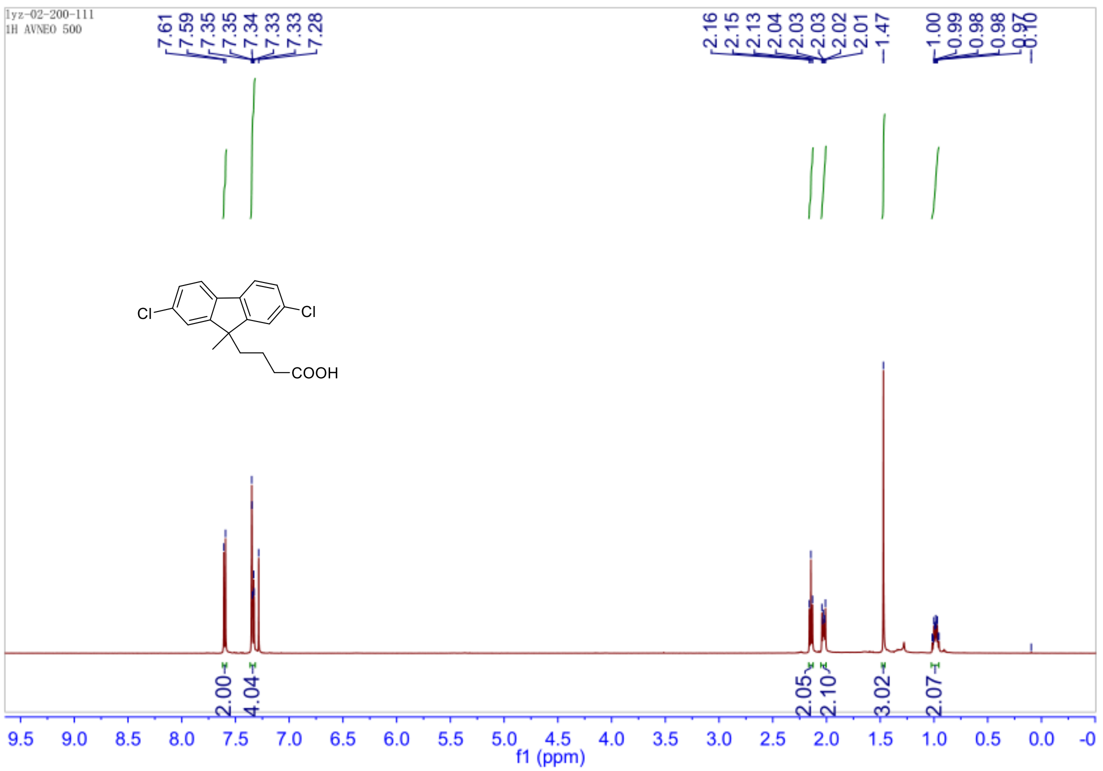

^{13}C NMR (126 MHz, $CDCl_3$)

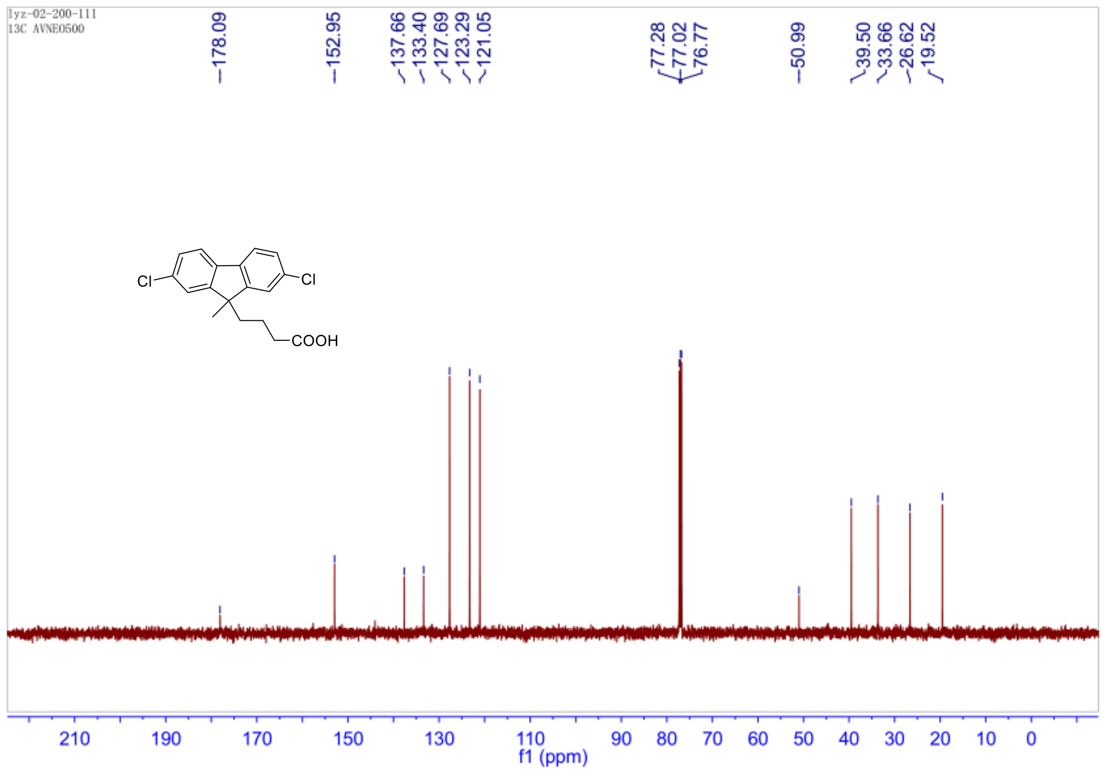

^{1}H NMR (500 MHz, $CDCl_3$)

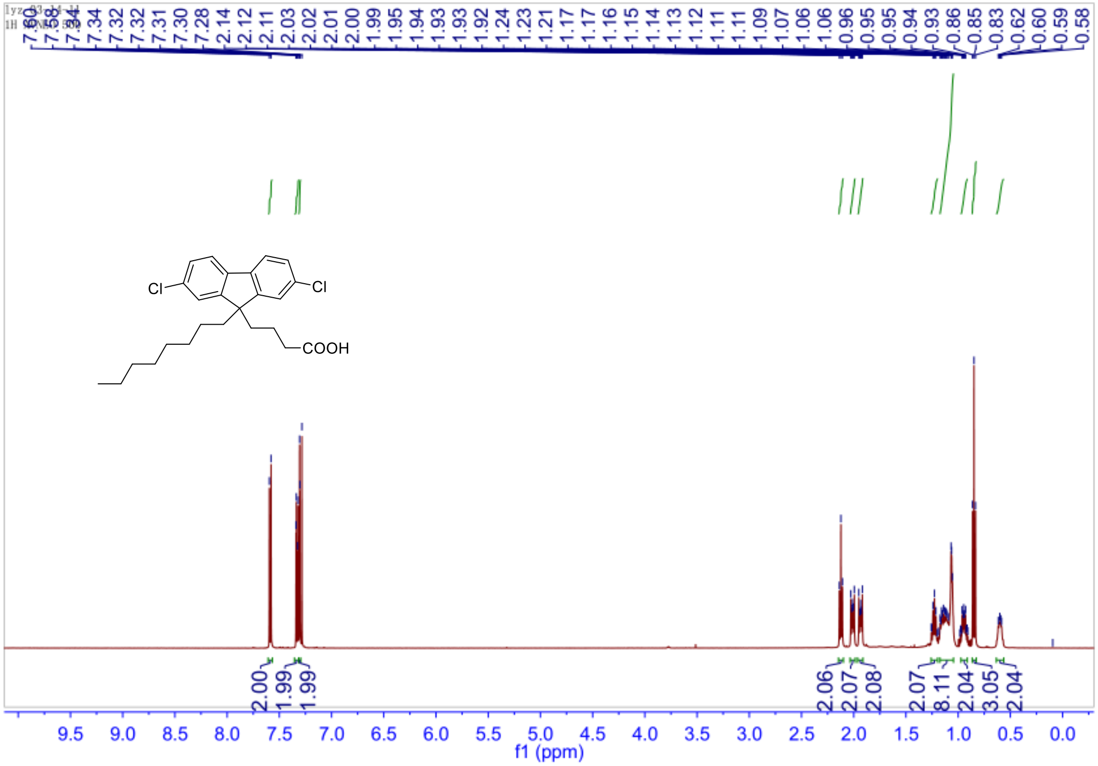

^{13}C NMR (126 MHz, $CDCl_3$)

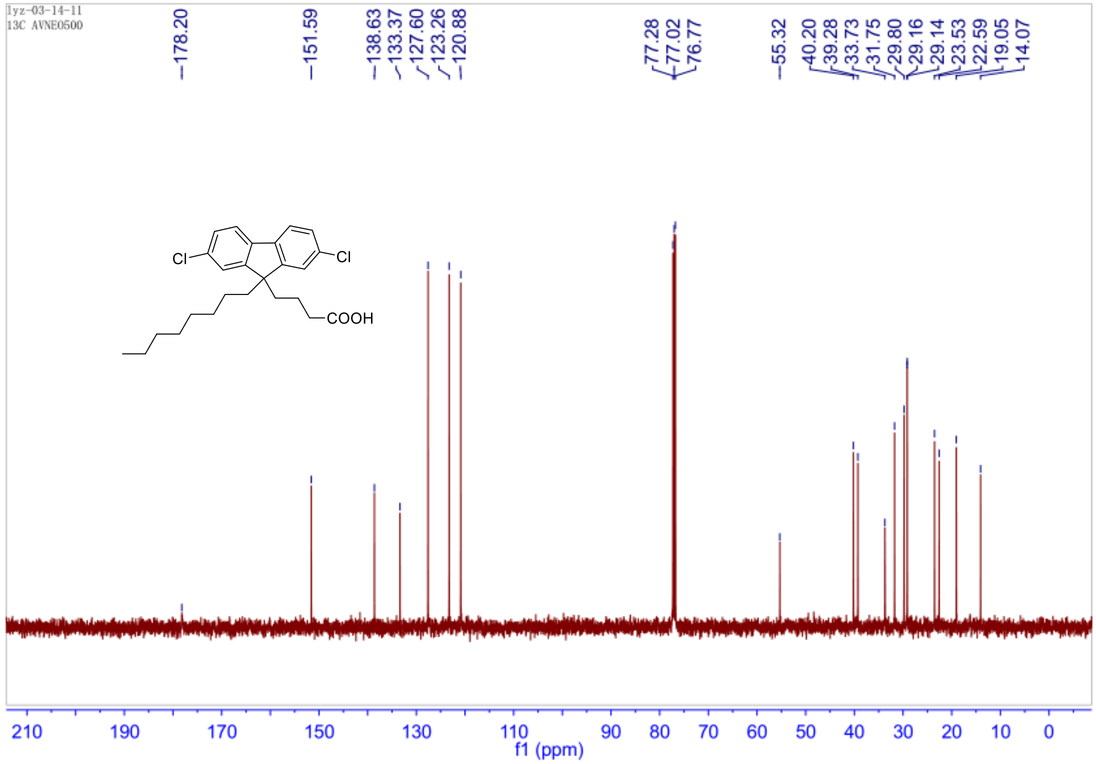

^{1}H NMR (500 MHz, $CDCl_3$)

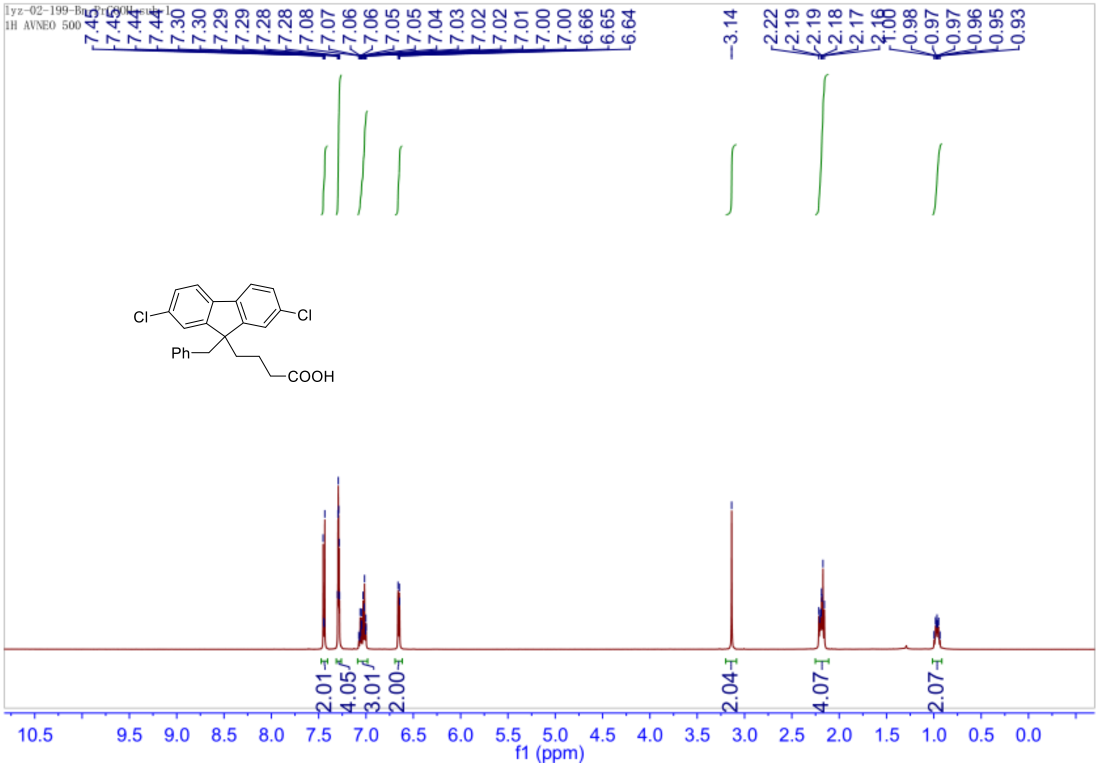

^{13}C NMR (126 MHz, $CDCl_3$)

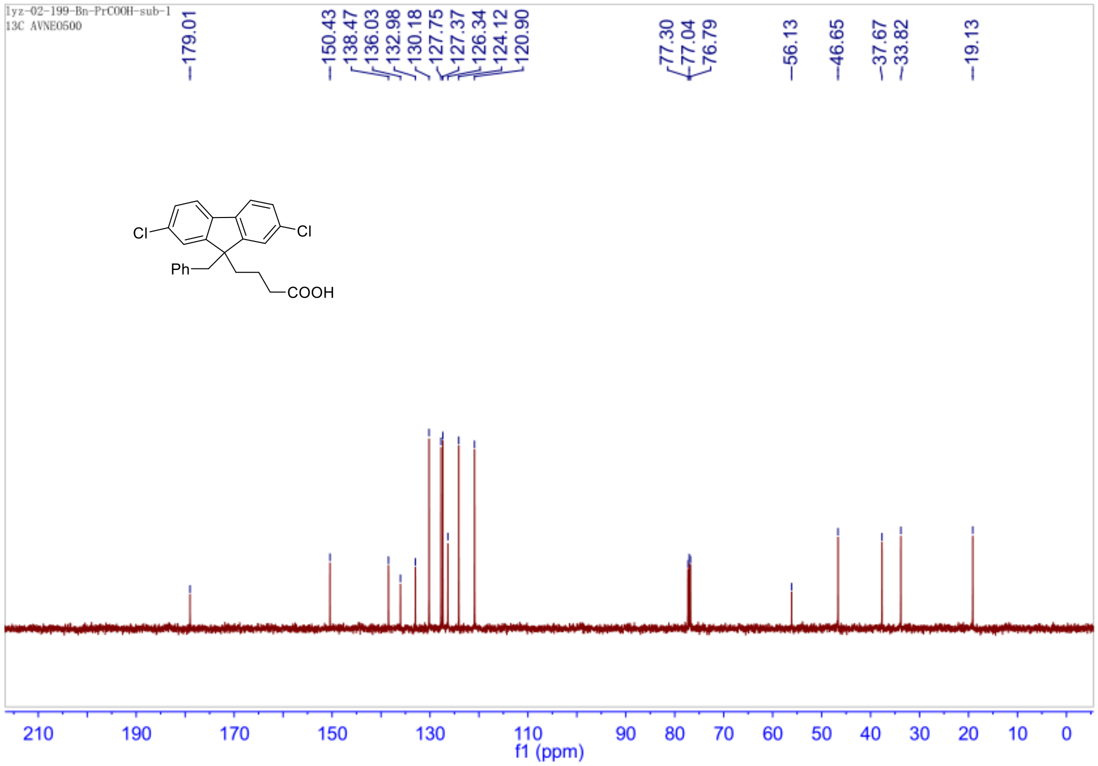

^{1}H NMR (500 MHz, $CDCl_3$)

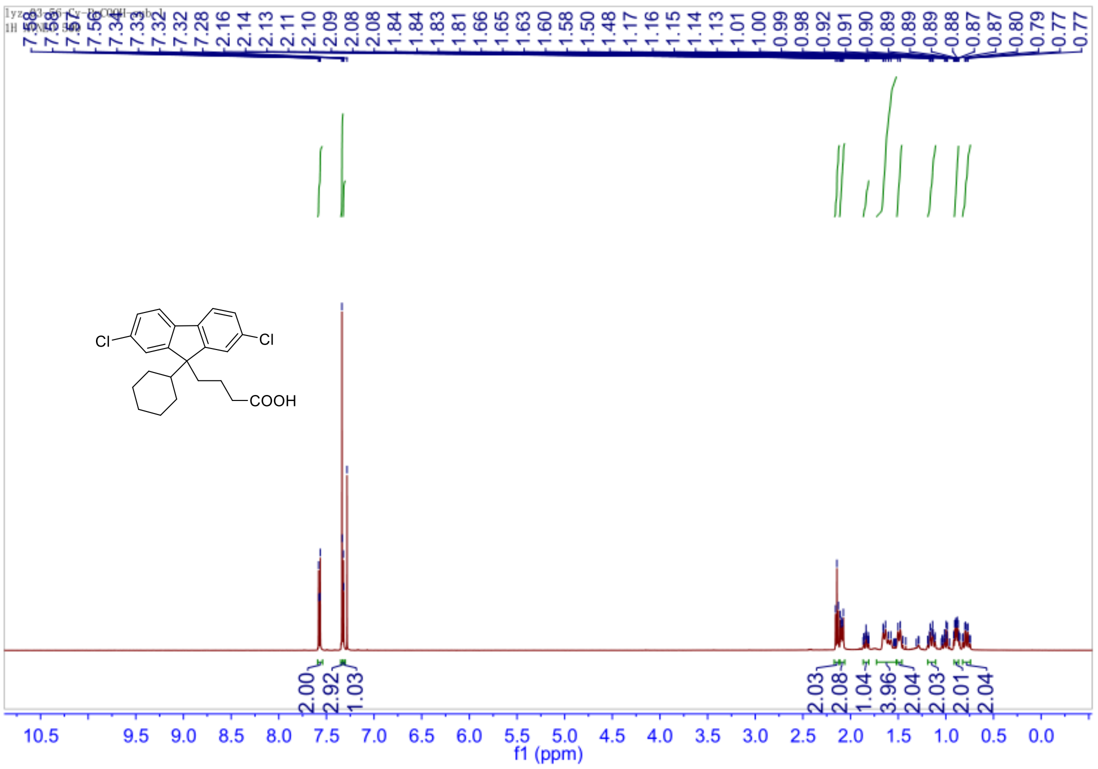

^{13}C NMR (126 MHz, $CDCl_3$)

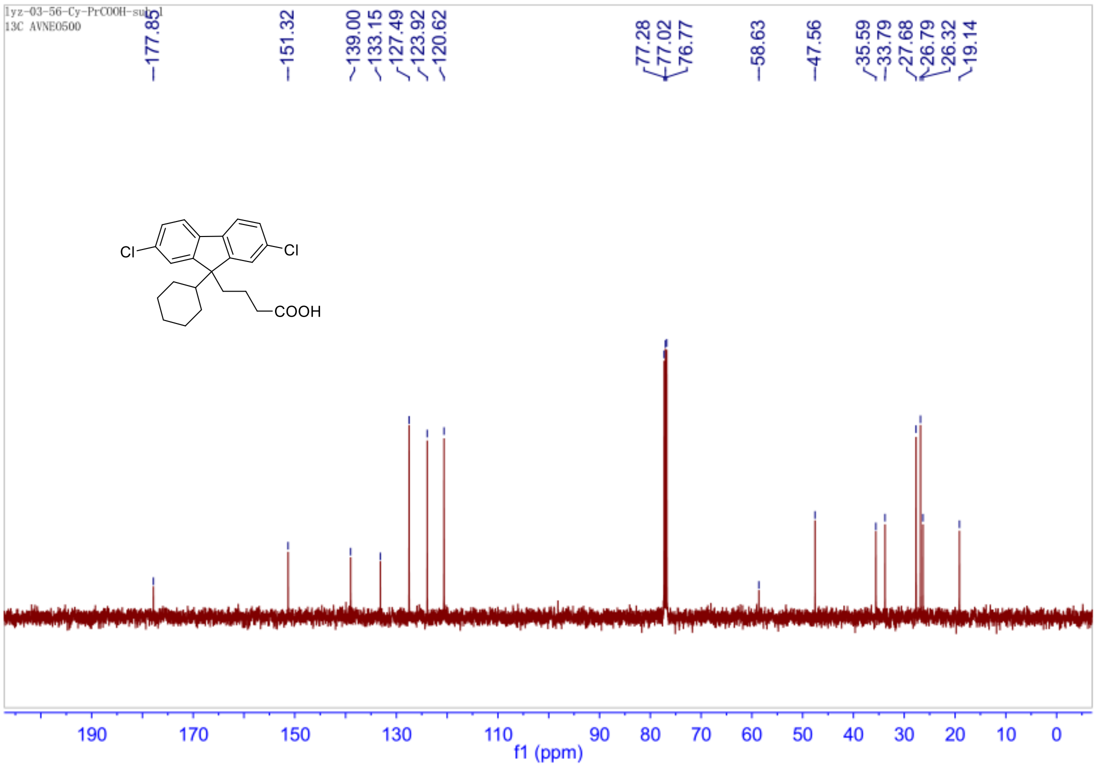

^{1}H NMR (500 MHz, $CDCl_3$)

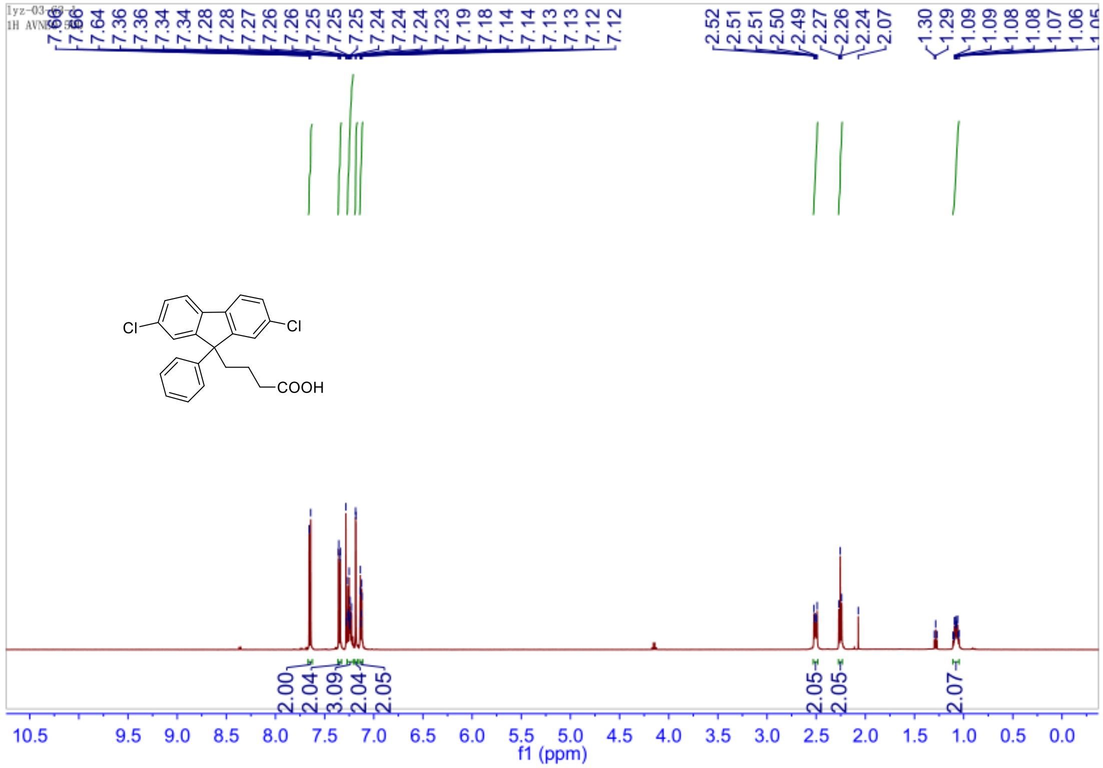

^{13}C NMR (126 MHz, $CDCl_3$)

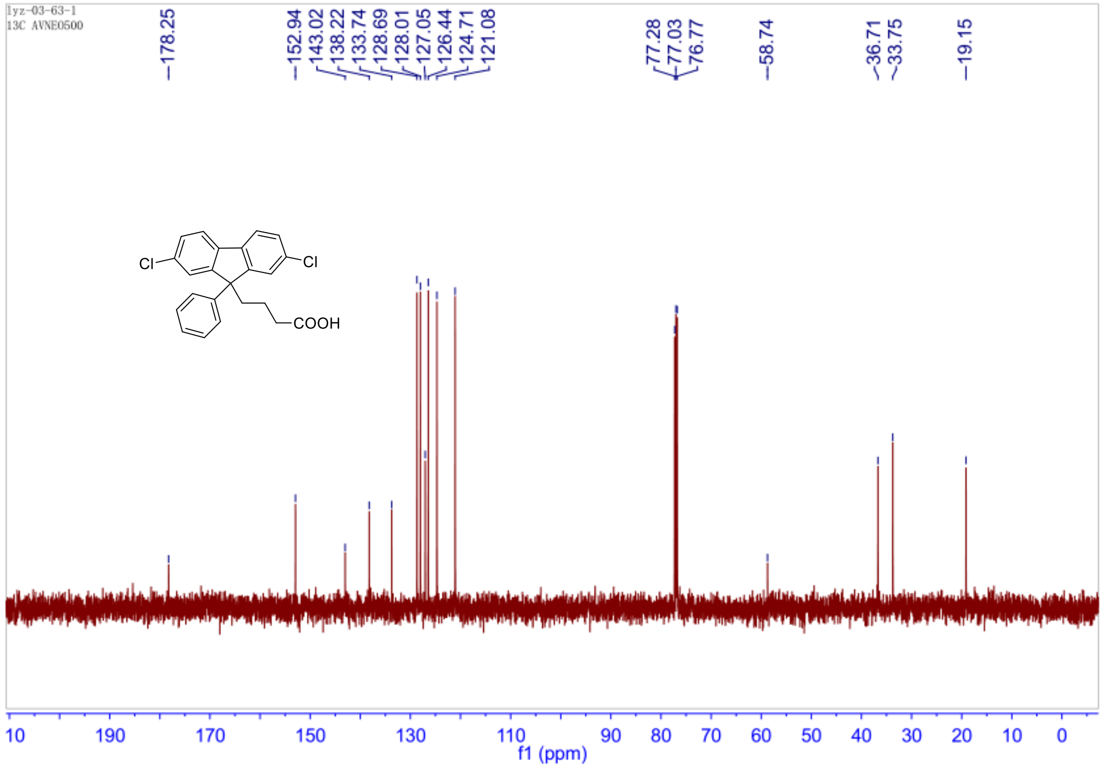

^{1}H NMR (500 MHz, $CDCl_3$)

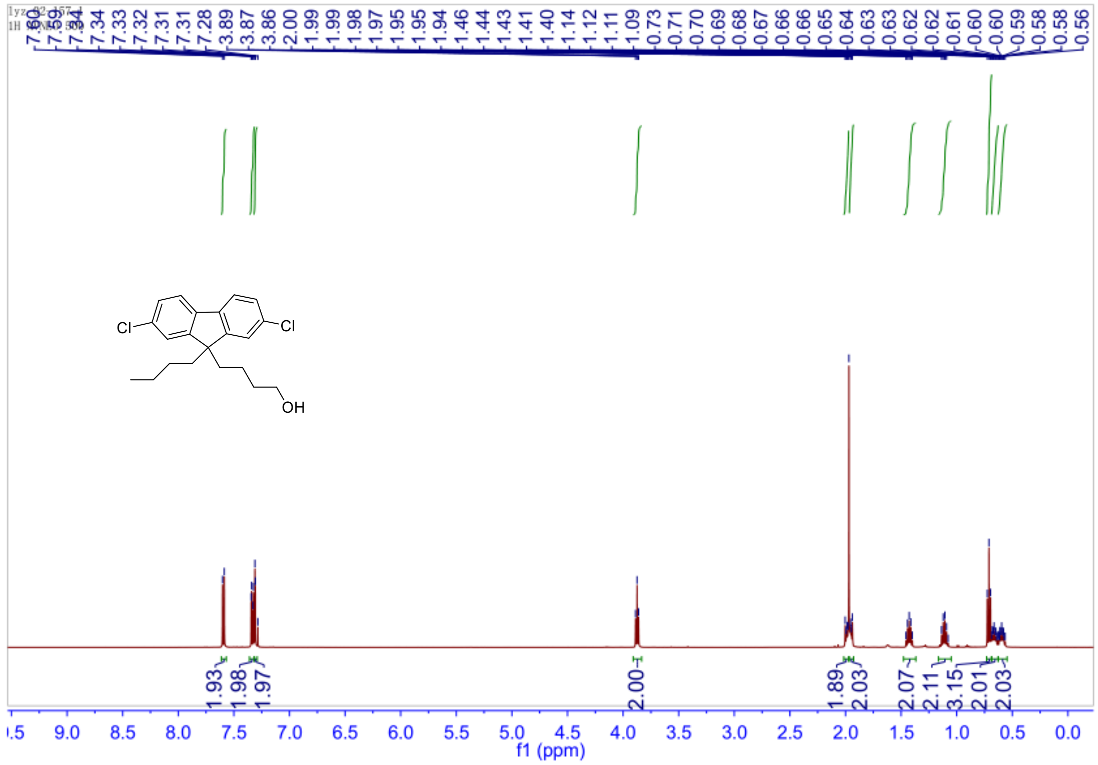

^{13}C NMR (126 MHz, $CDCl_3$)

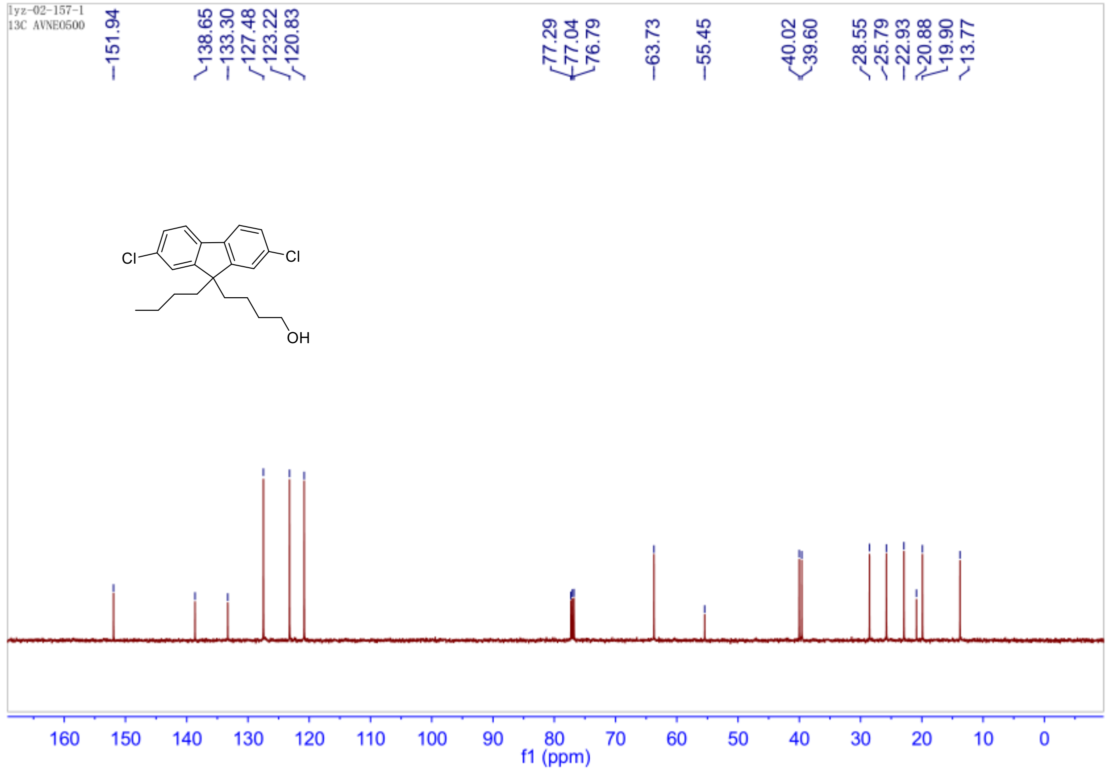

^{1}H NMR (300 MHz, $CDCl_3$)

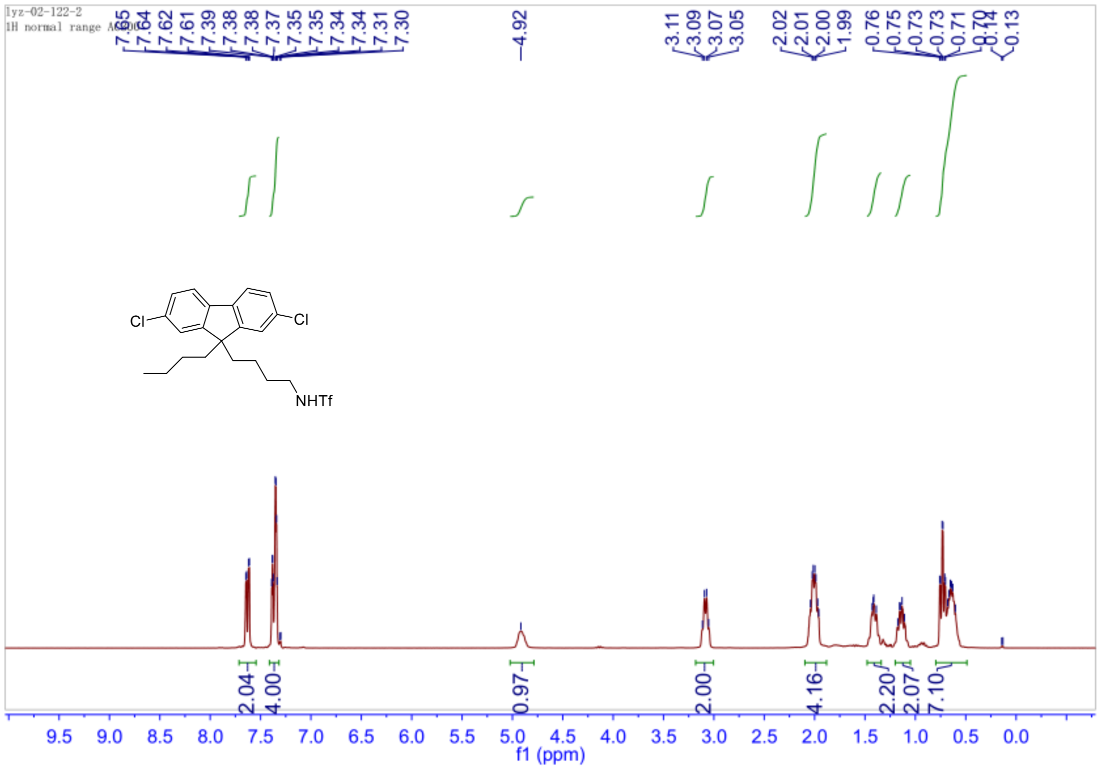

^{13}C NMR (75 MHz, $CDCl_3$)

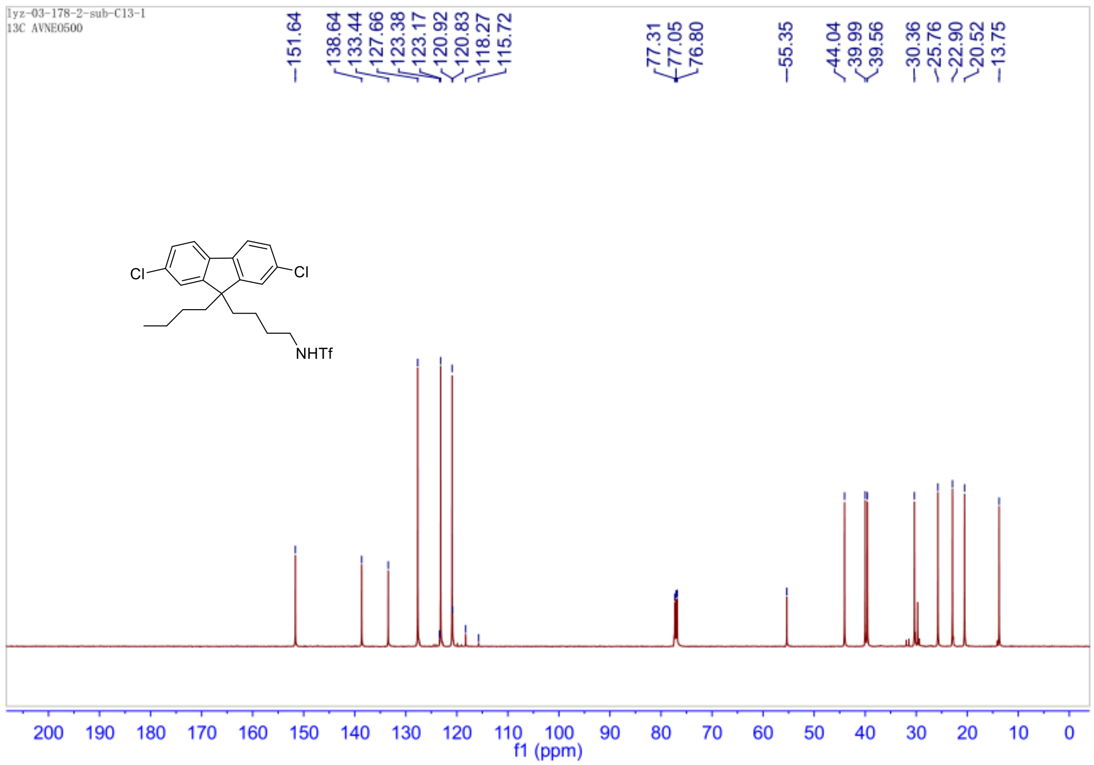

^{19}F NMR (471 MHz, $CDCl_3$)

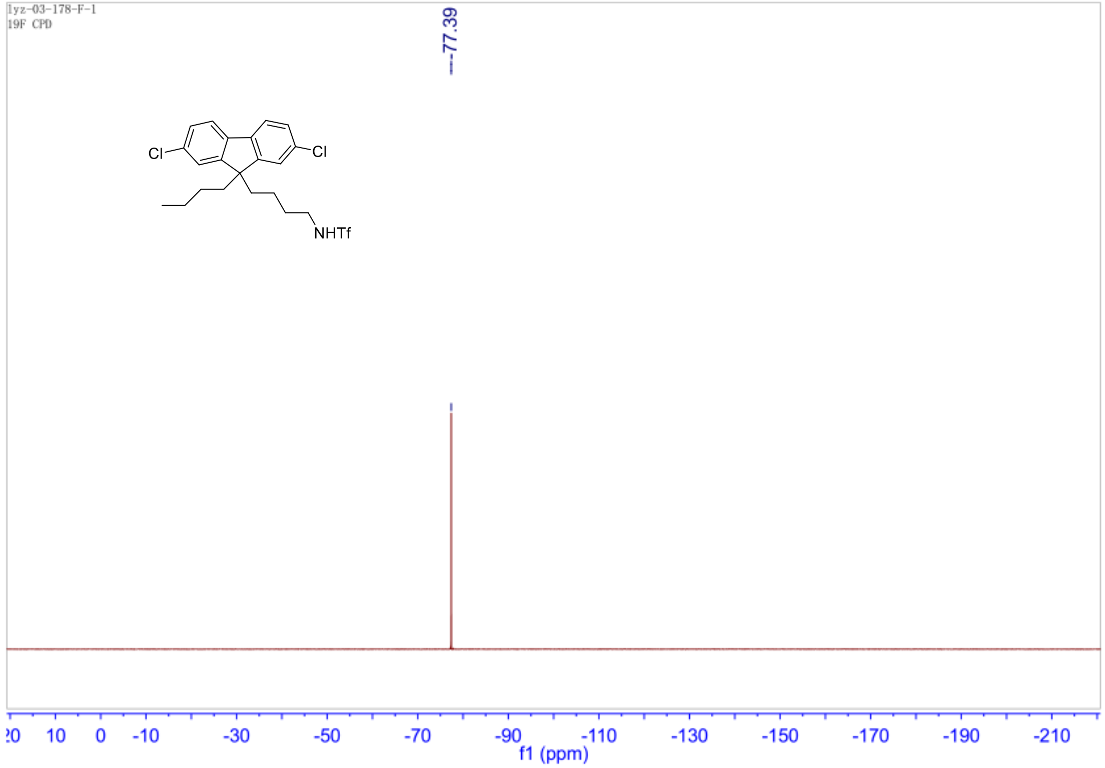

^{1}H NMR (500 MHz, $CDCl_3$)

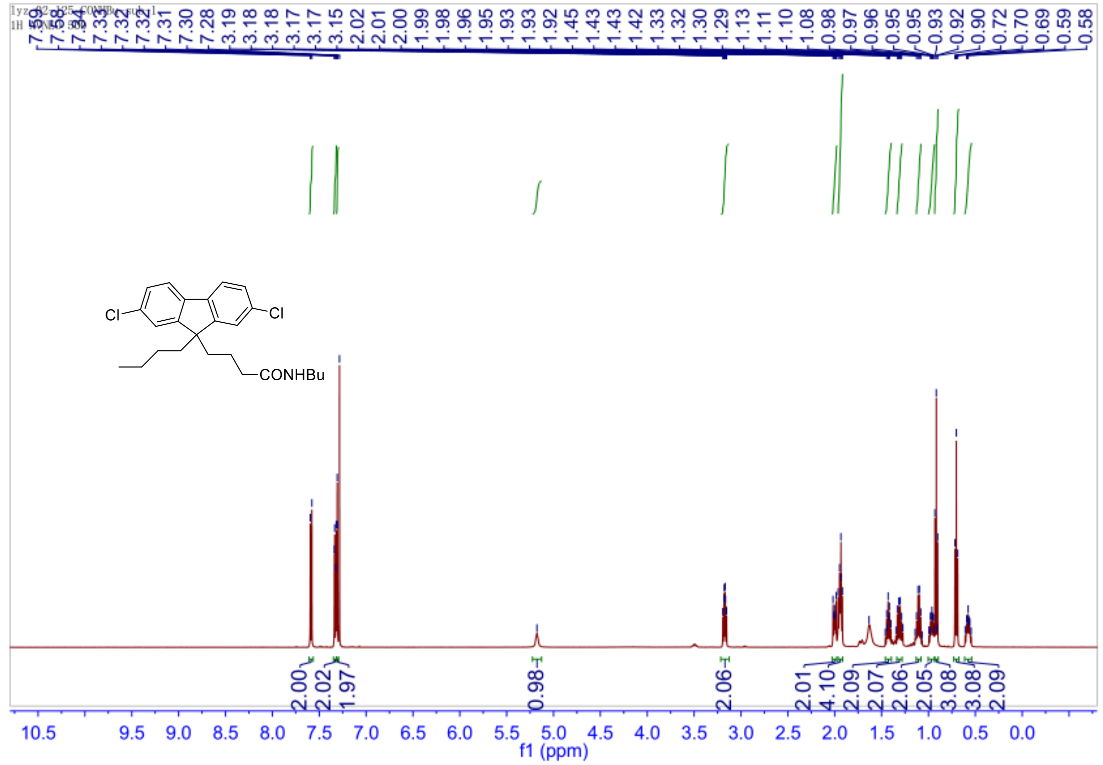

^{13}C NMR (126 MHz, $CDCl_3$)

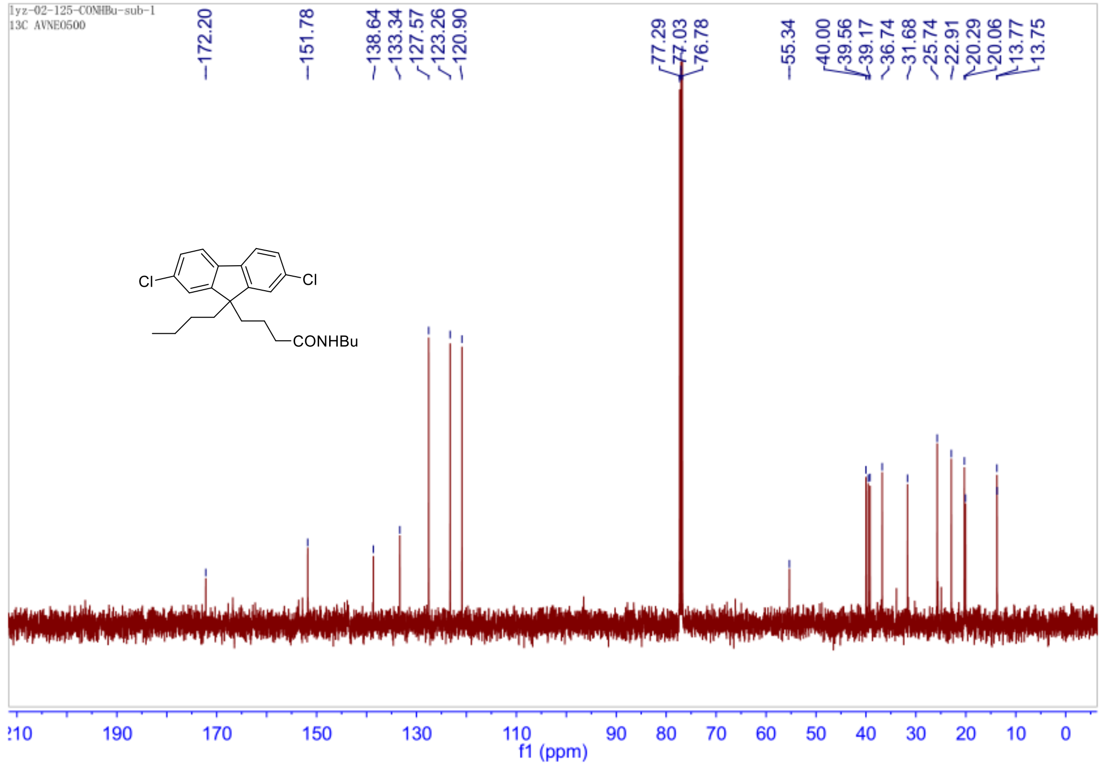

^{1}H NMR (500 MHz, $CDCl_3$)

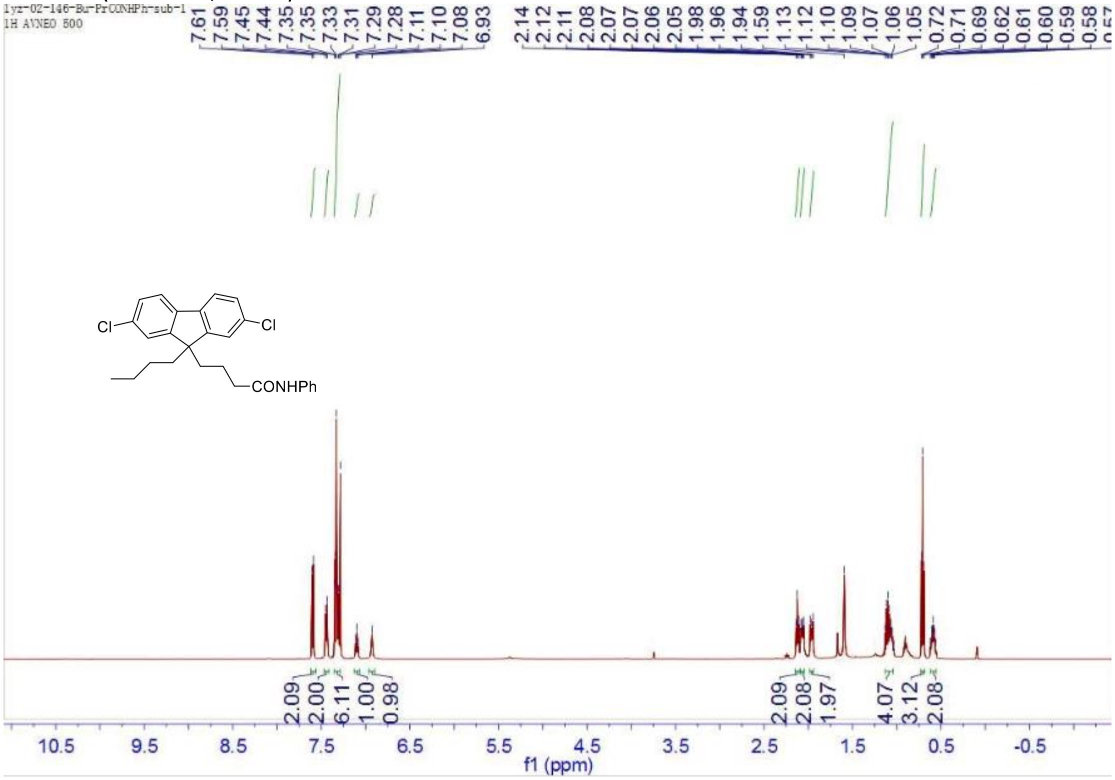

^{13}C NMR (126 MHz, $CDCl_3$)

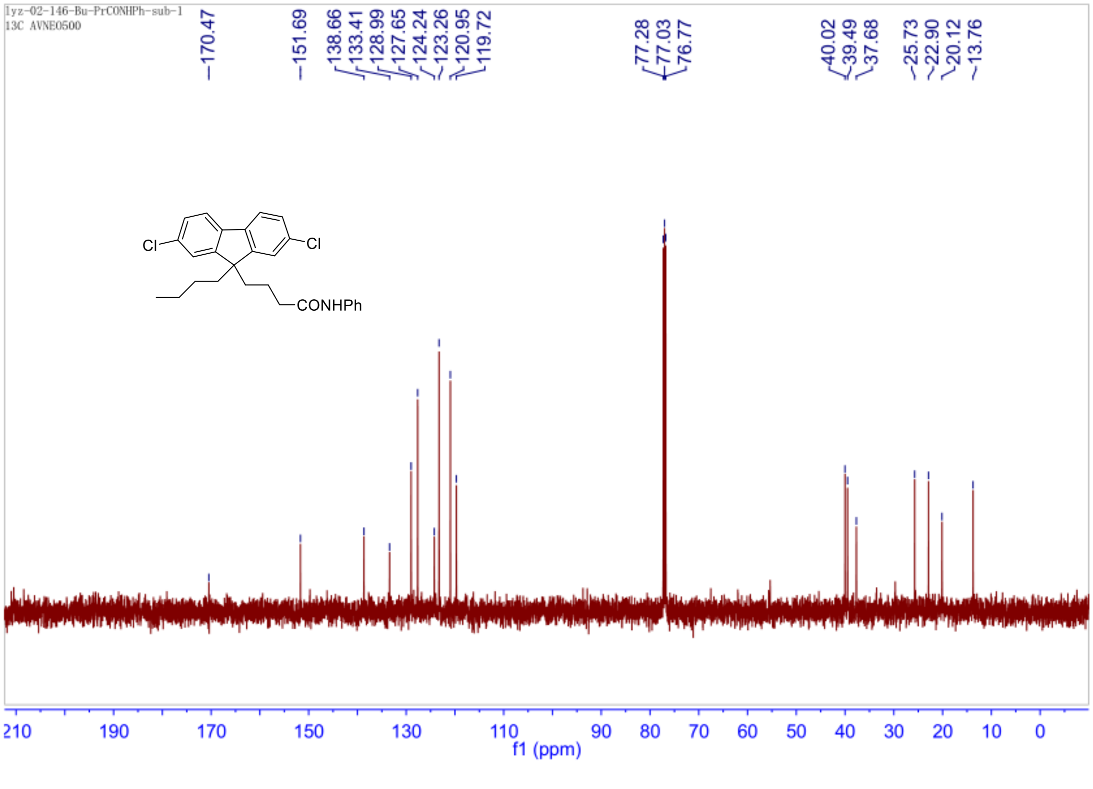

^{1}H NMR (500 MHz, $CDCl_3$)

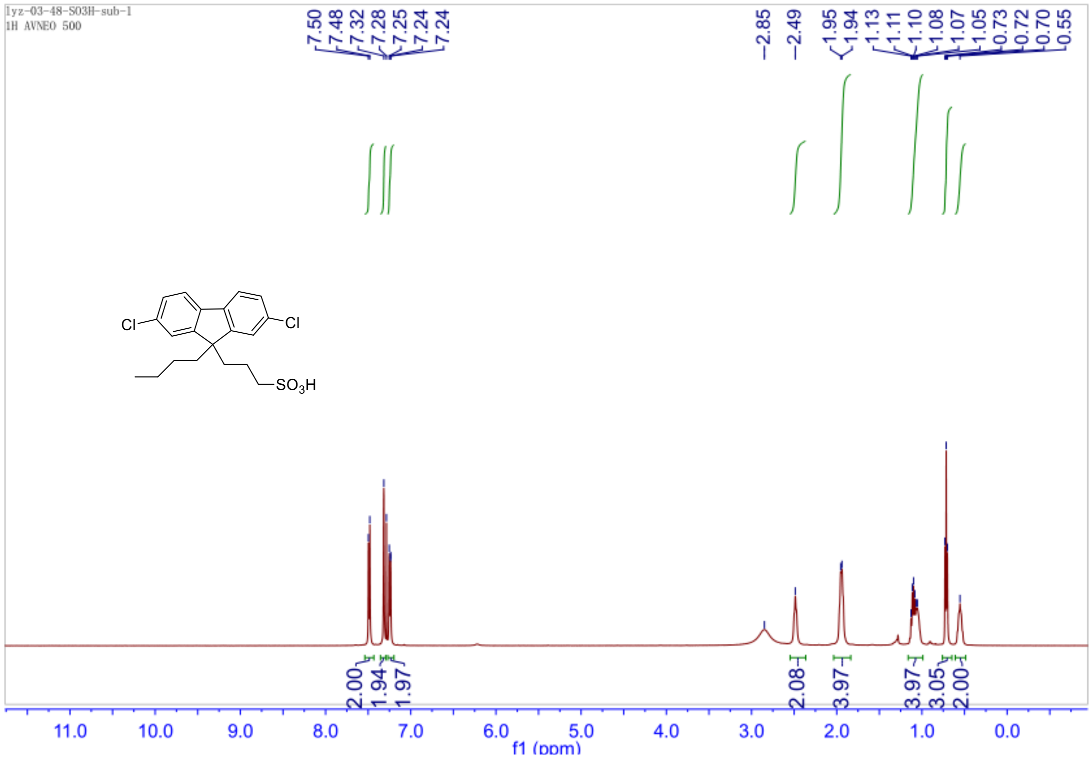

^{13}C NMR (126 MHz, $CDCl_3$)

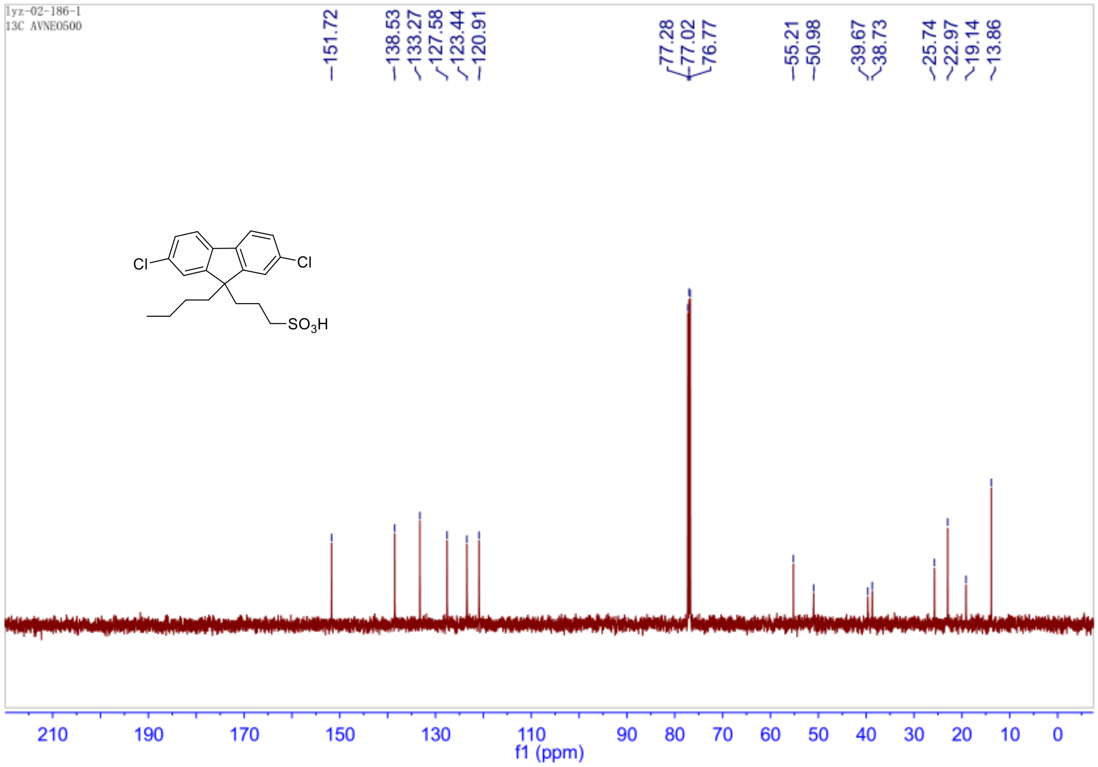

^{1}H NMR (500 MHz, $CDCl_3$)

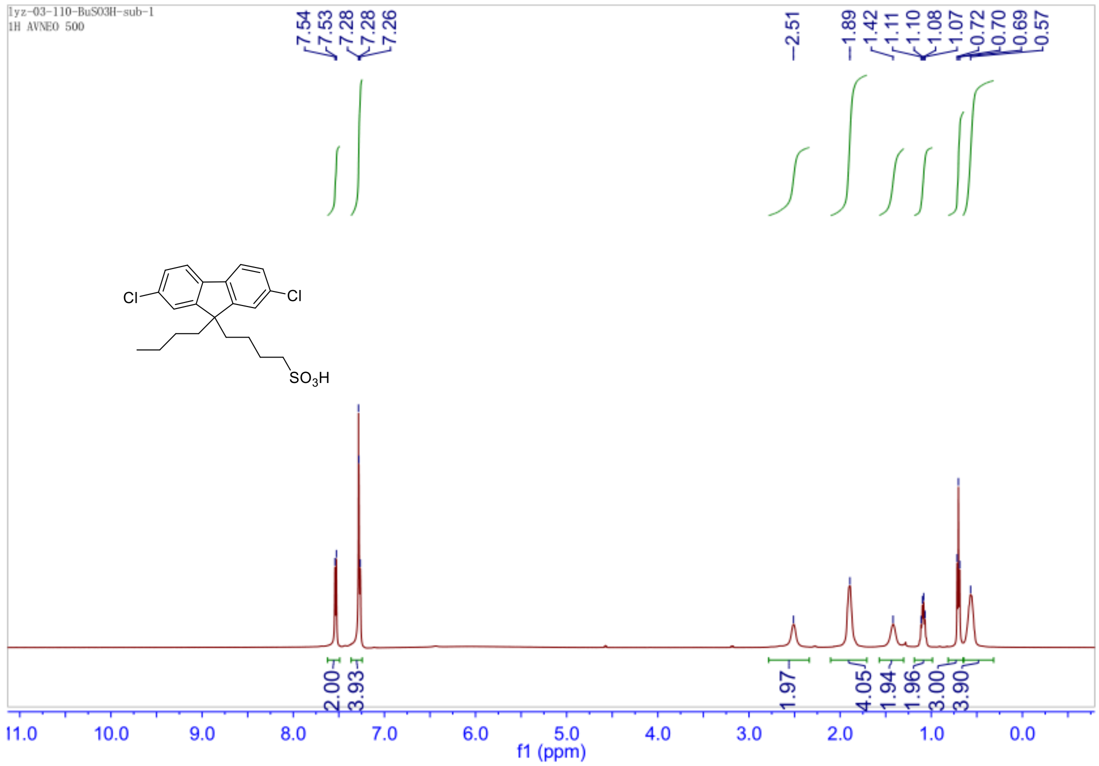

^{13}C NMR (126 MHz, $CDCl_3$)

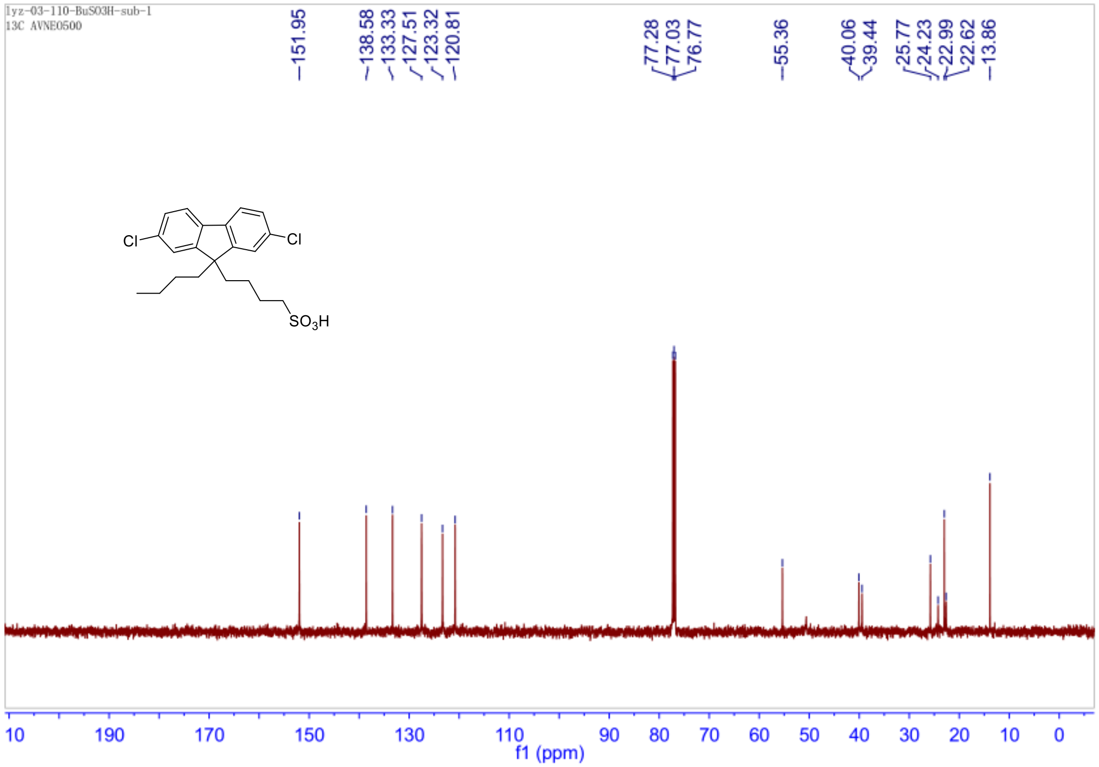

^{1}H NMR (500 MHz, $CDCl_3$)

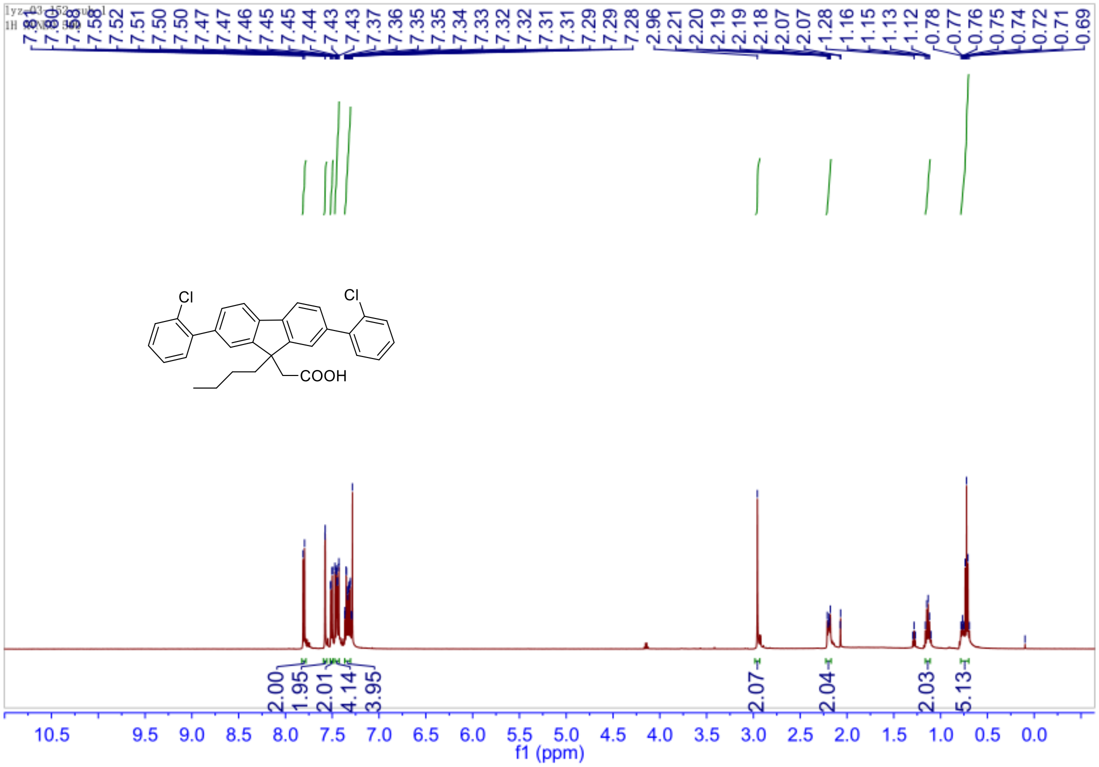

^{13}C NMR (126 MHz, $CDCl_3$)

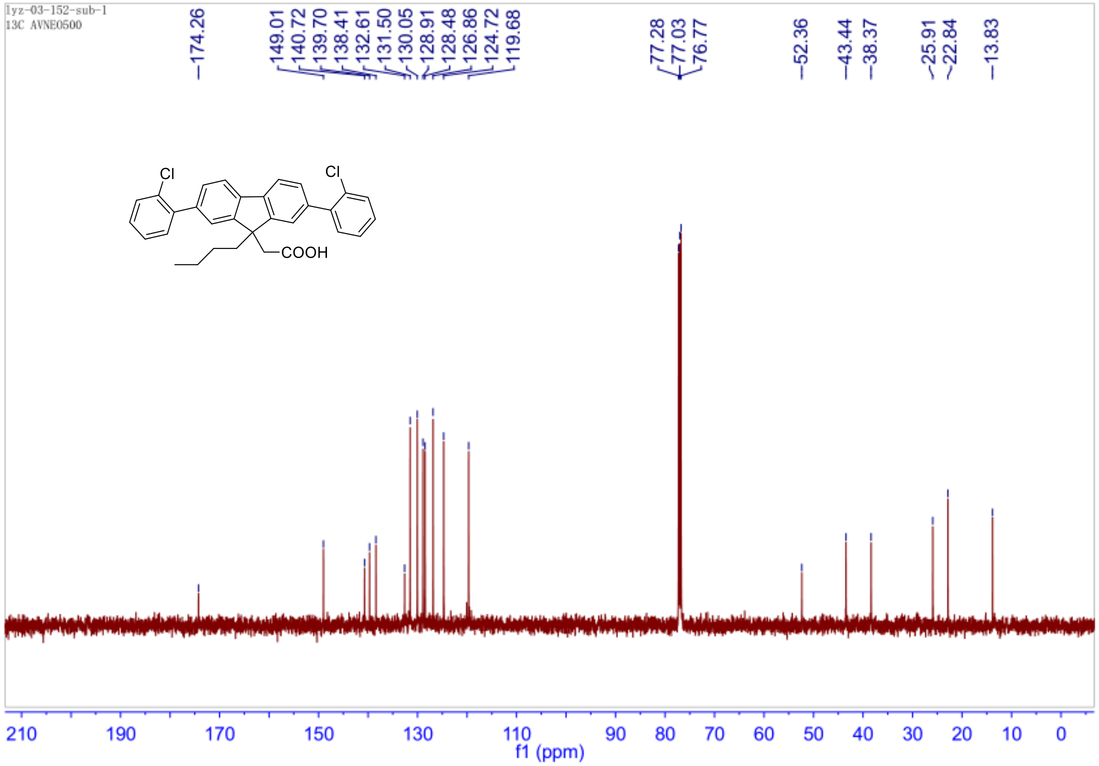

^{1}H NMR (500 MHz, $CDCl_3$)

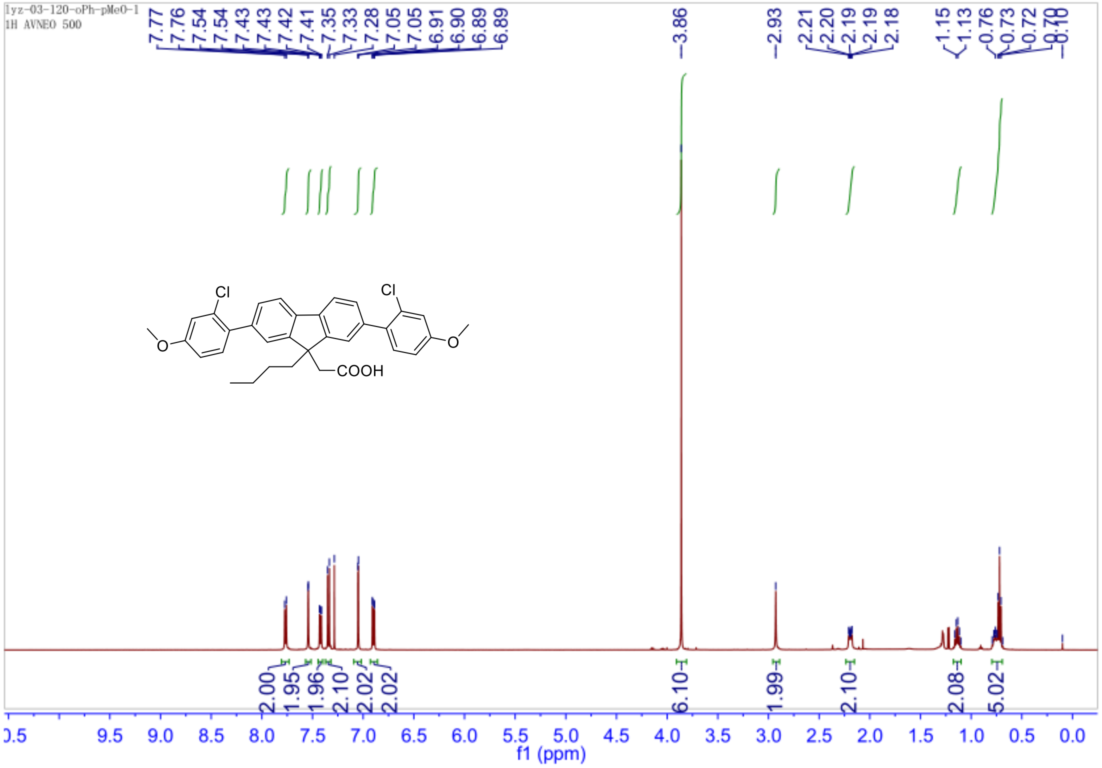

^{13}C NMR (126 MHz, $CDCl_3$)

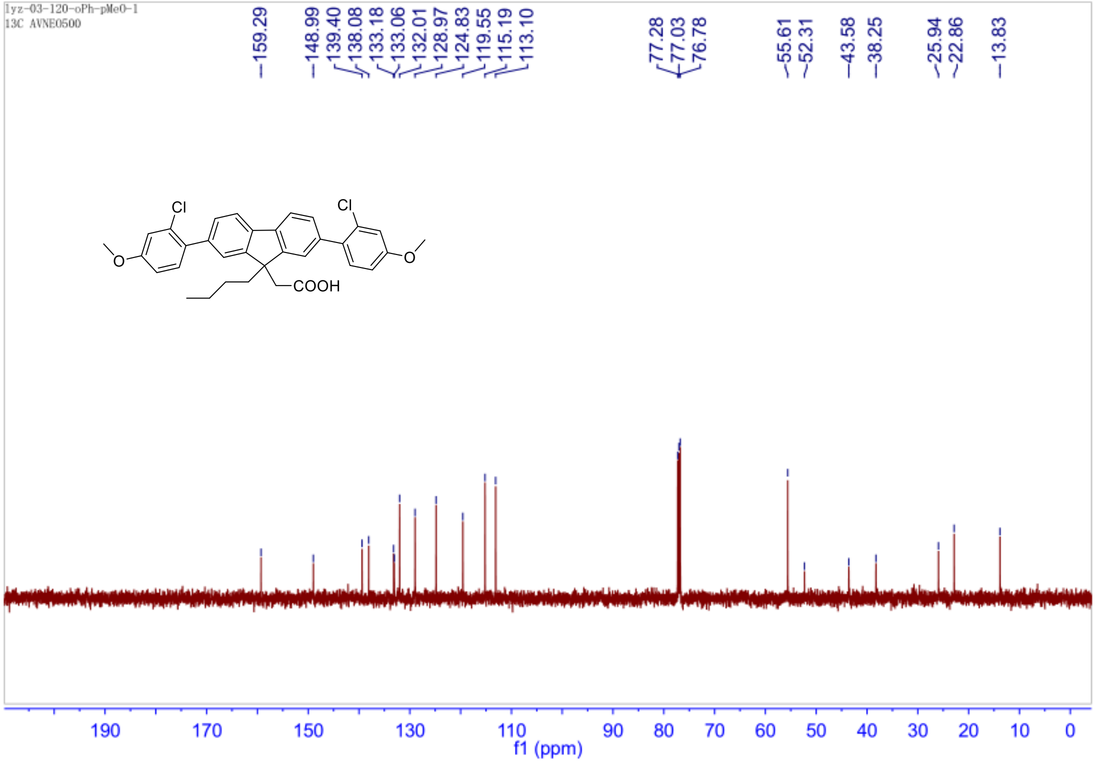

^{1}H NMR (500 MHz, $CDCl_3$)

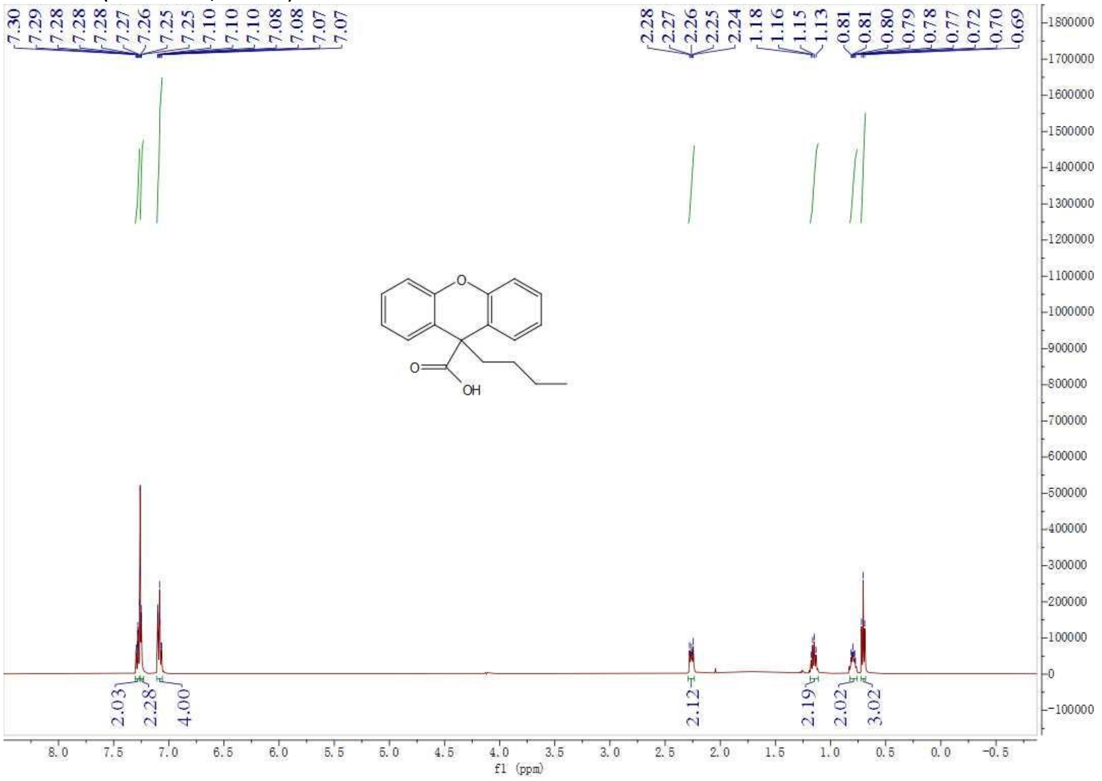

^{13}C NMR (126 MHz, $CDCl_3$)

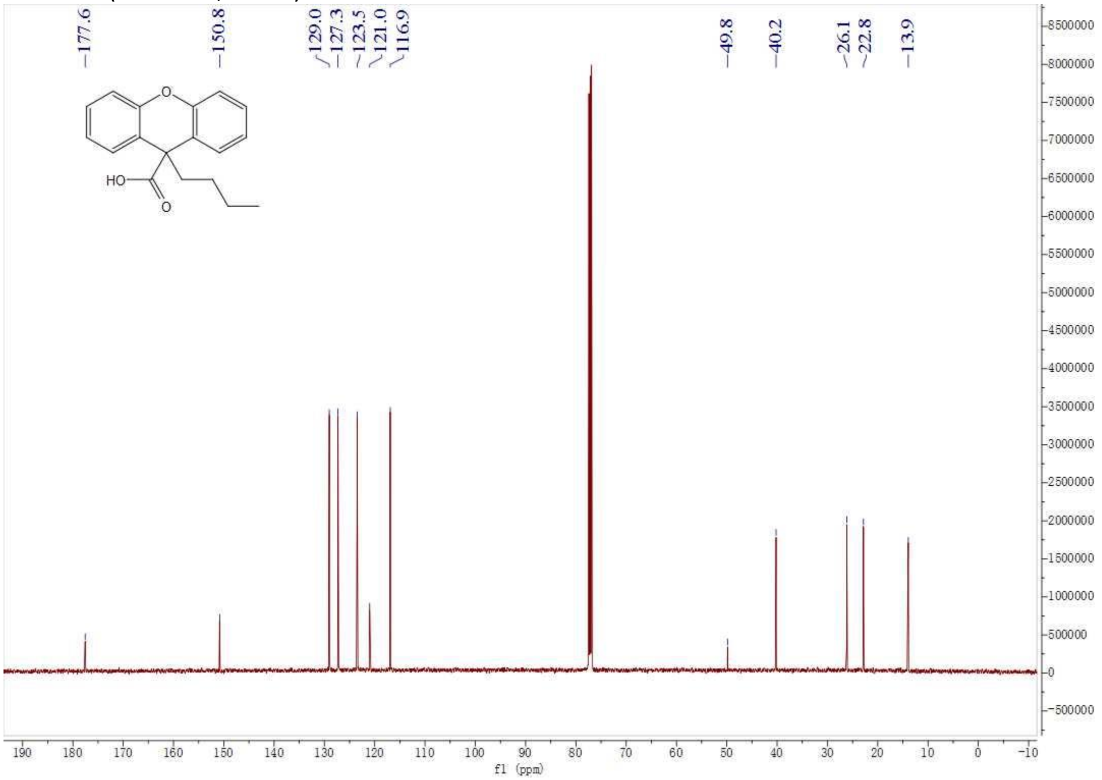

^{1}H NMR (400 MHz, $CDCl_3$)

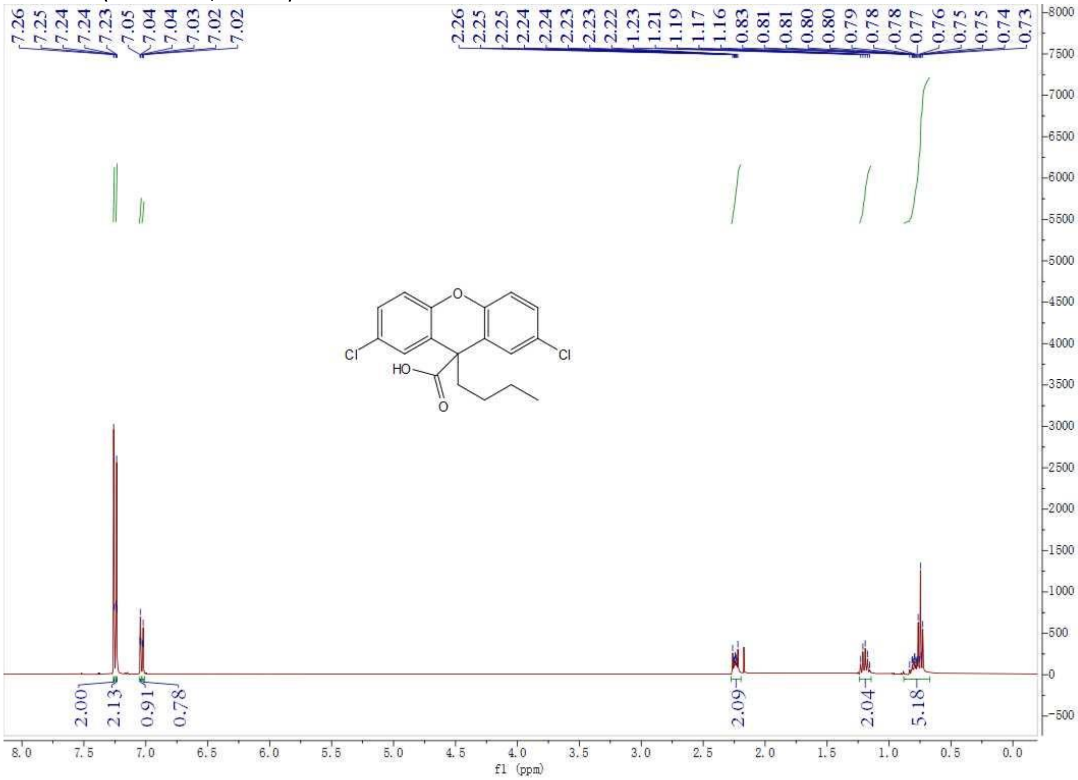

^{13}C NMR (126 MHz, $CDCl_3$)

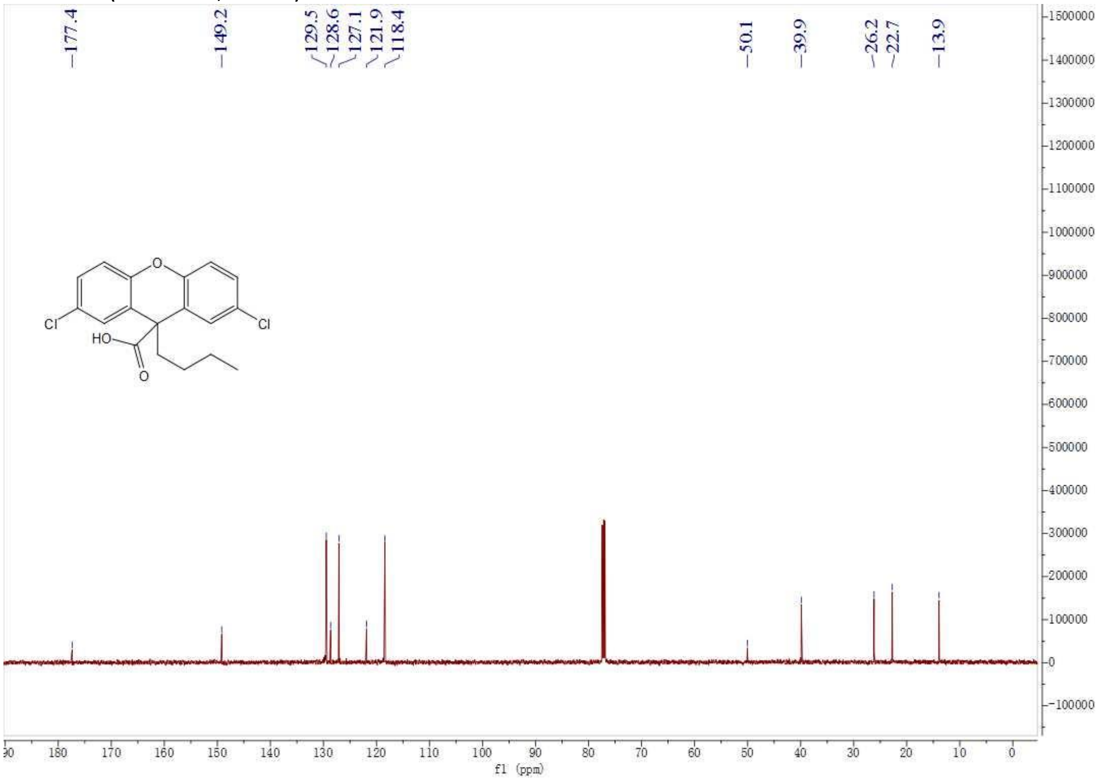

^{1}H NMR (500 MHz, $CDCl_3$)

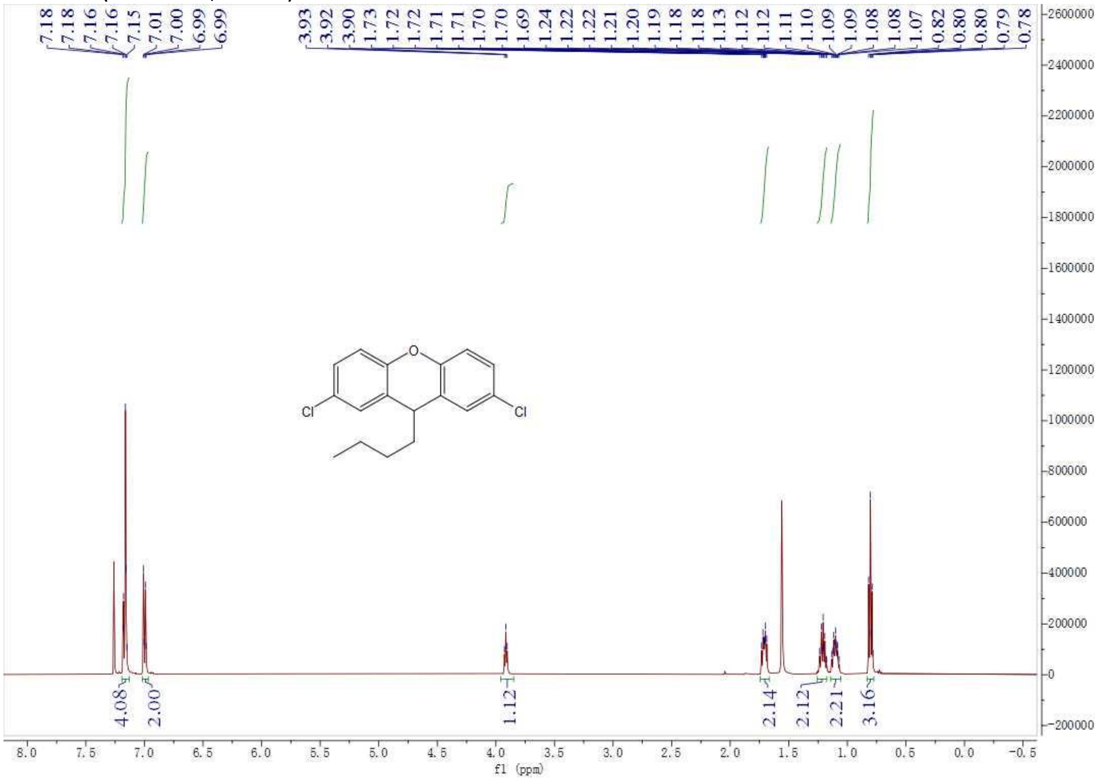

^{13}C NMR (126 MHz, $CDCl_3$)

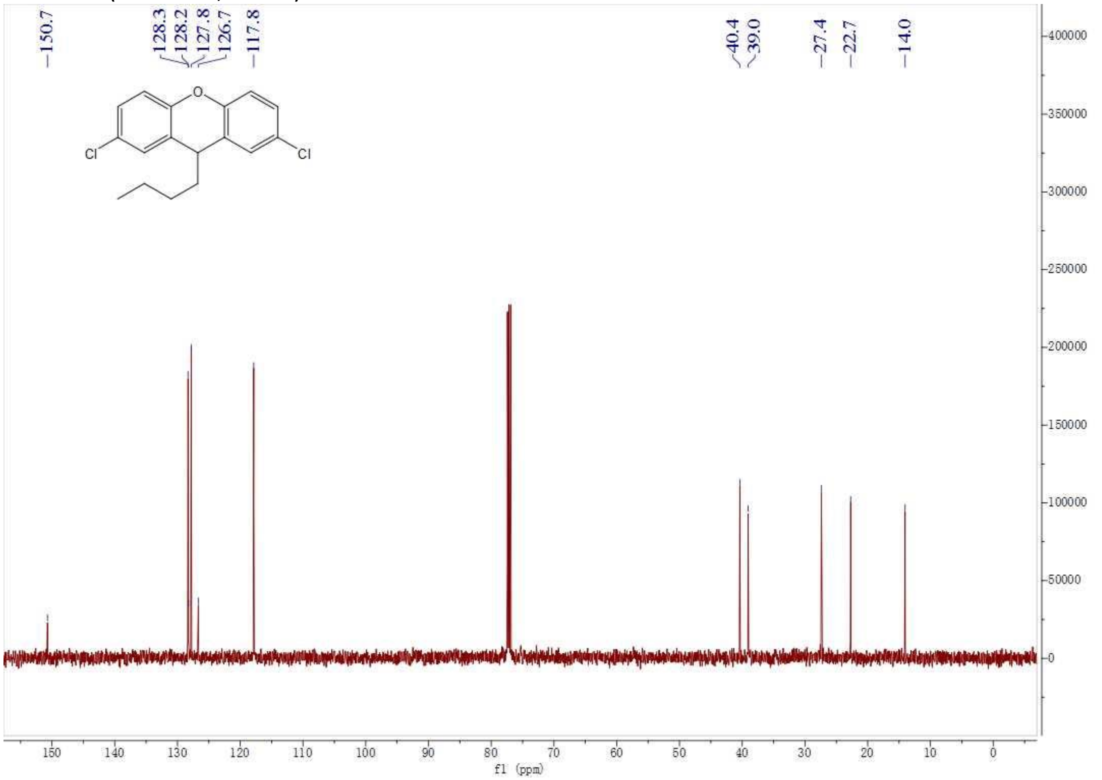

^{1}H NMR (400 MHz, $CDCl_3$)

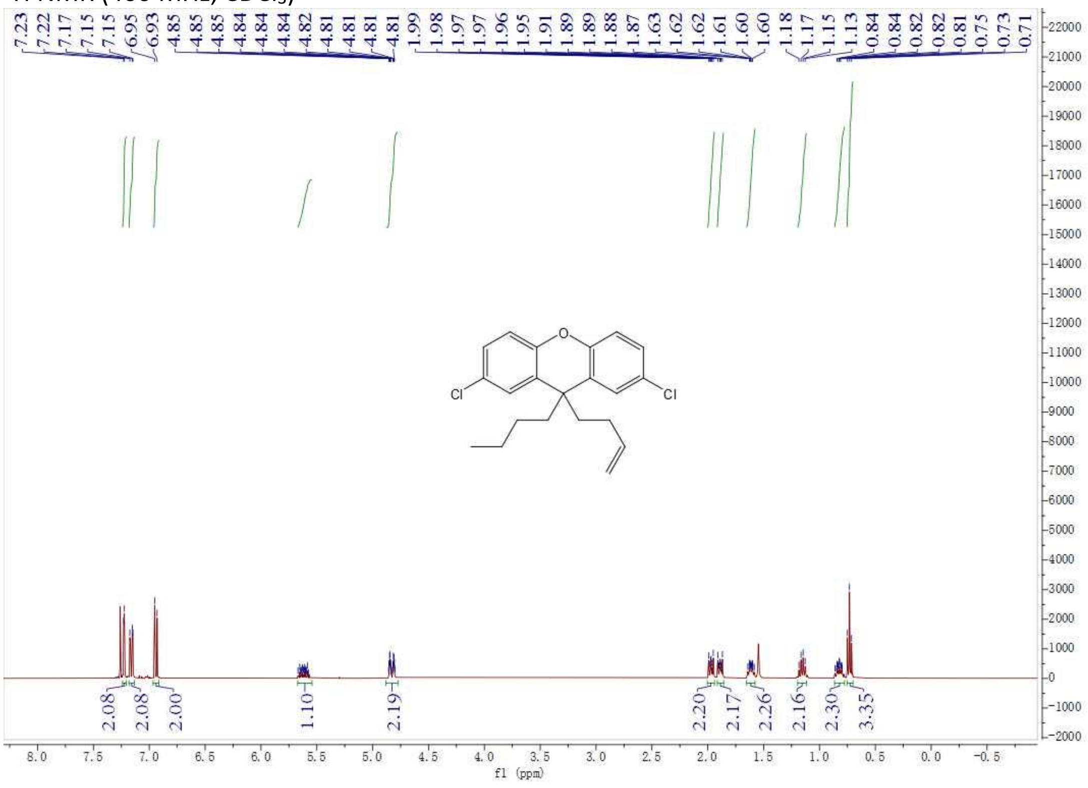

^{13}C NMR (126 MHz, $CDCl_3$)

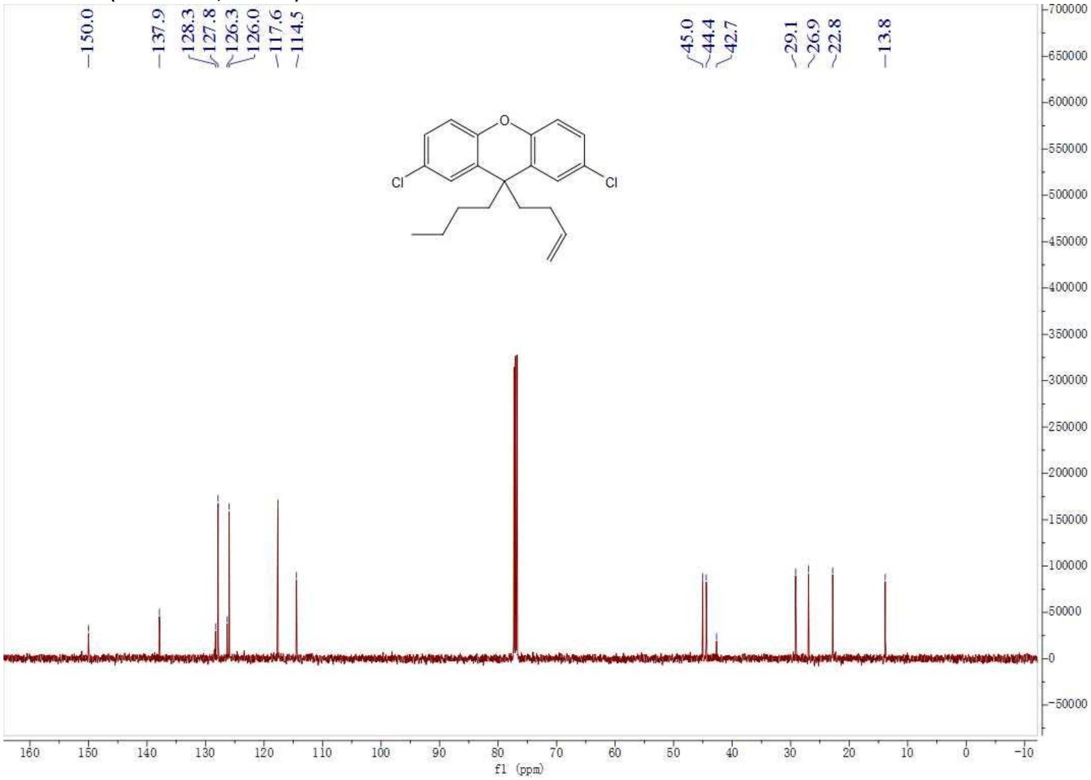

^{1}H NMR (500 MHz, $CDCl_3$)

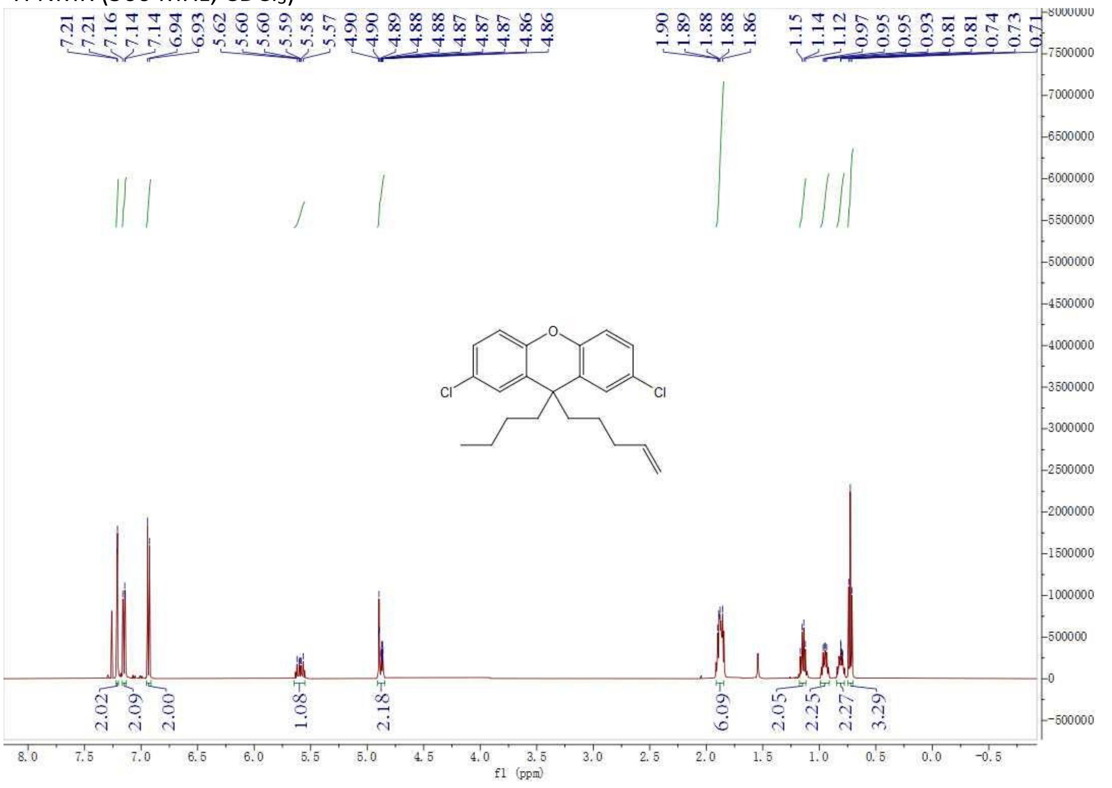

^{13}C NMR (126 MHz, $CDCl_3$)

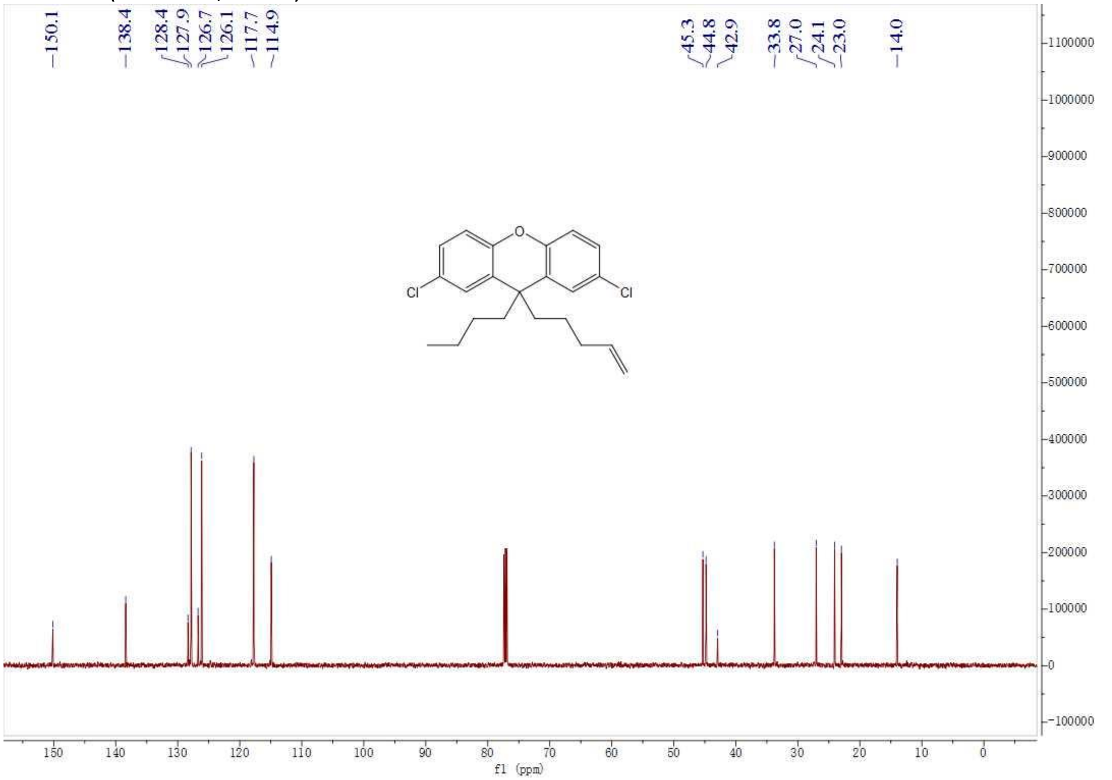

^{1}H NMR (500 MHz, $CDCl_3$)

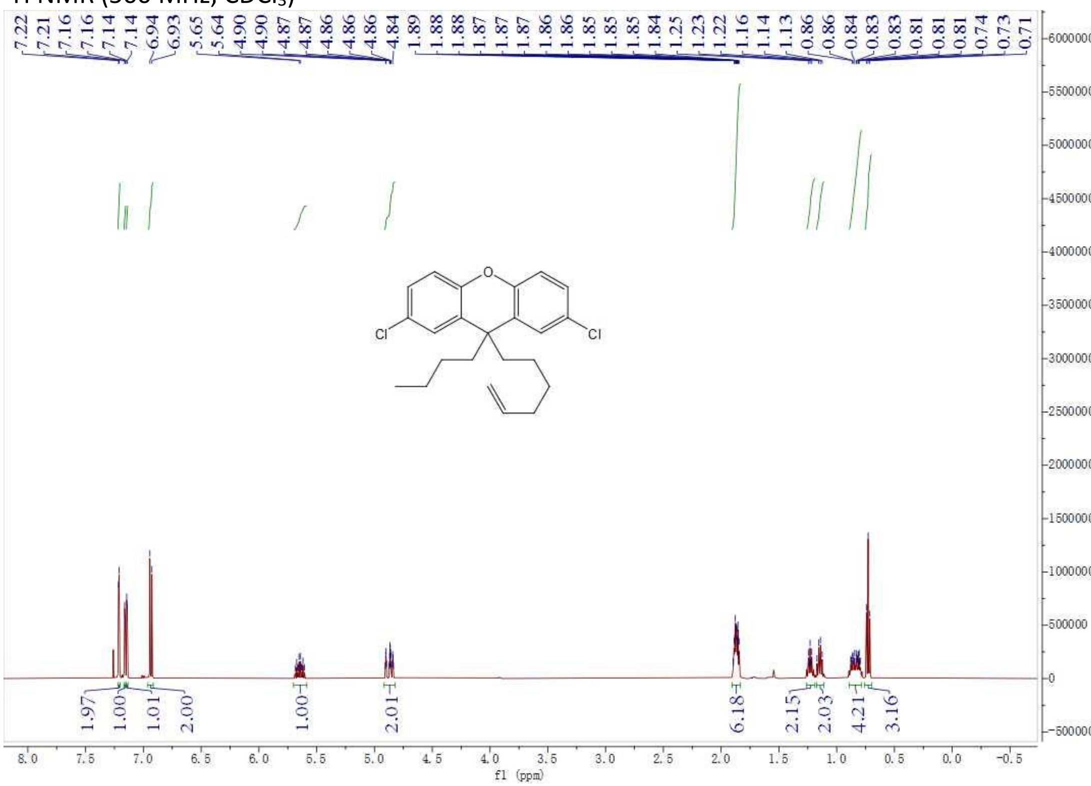

^{13}C NMR (126 MHz, $CDCl_3$)

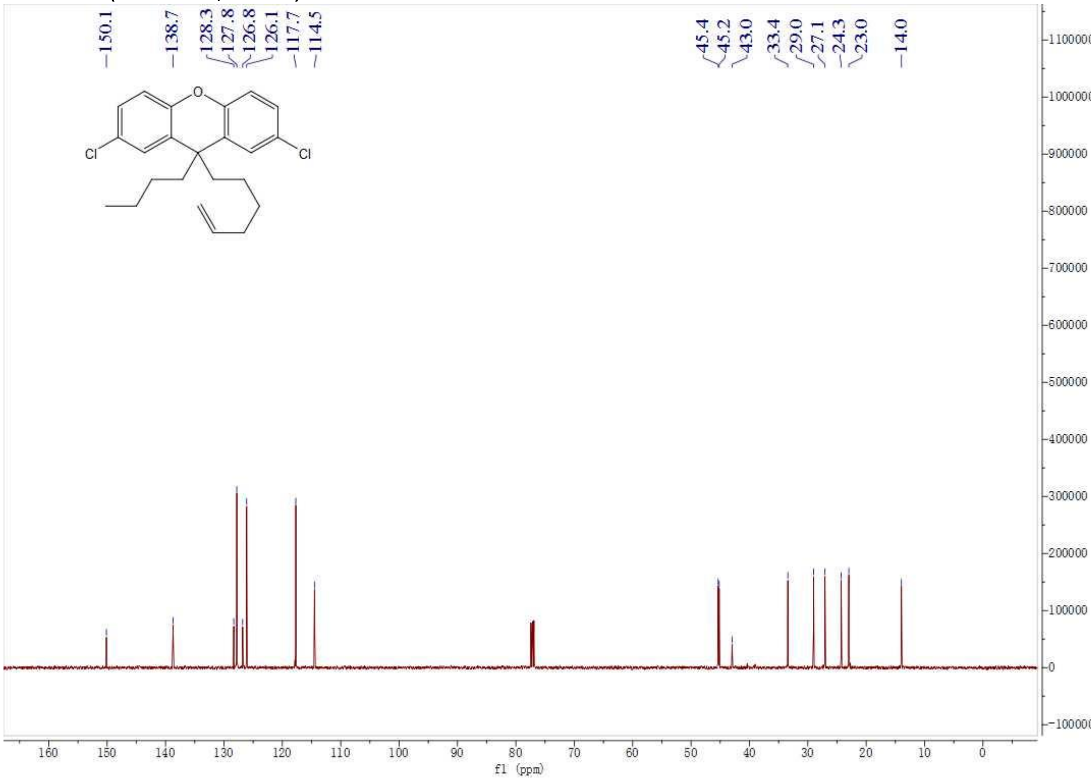

^{1}H NMR (500 MHz, $CDCl_3$)

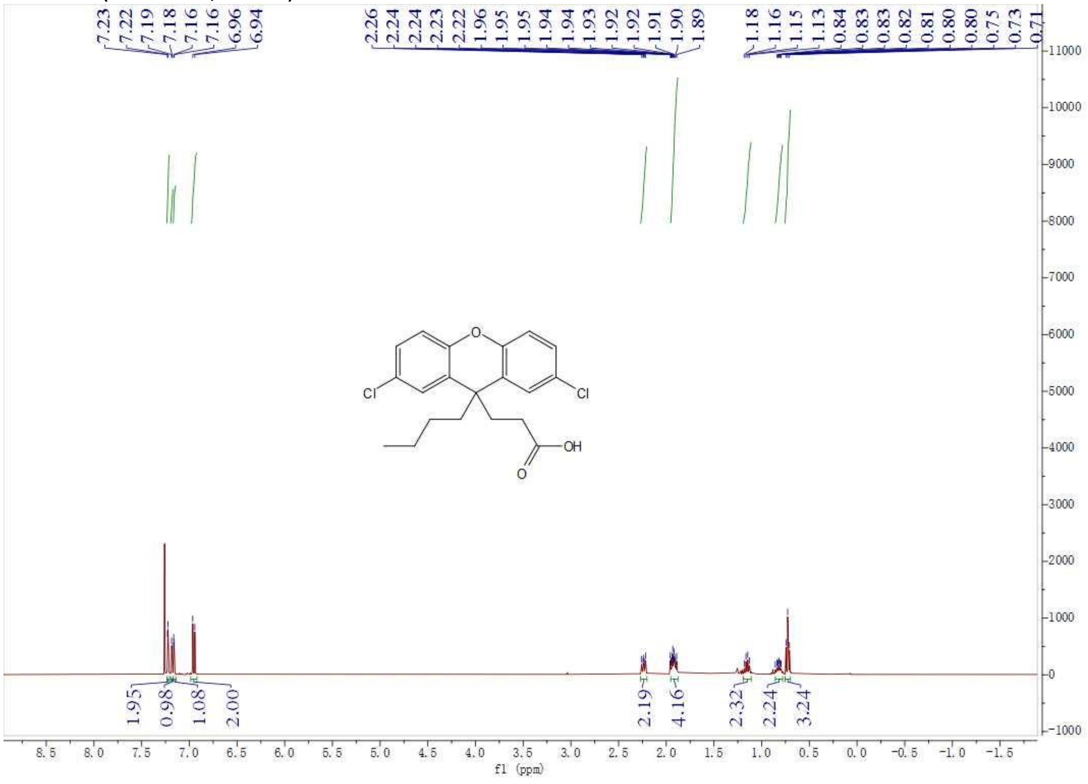

^{13}C NMR (126 MHz, $CDCl_3$)

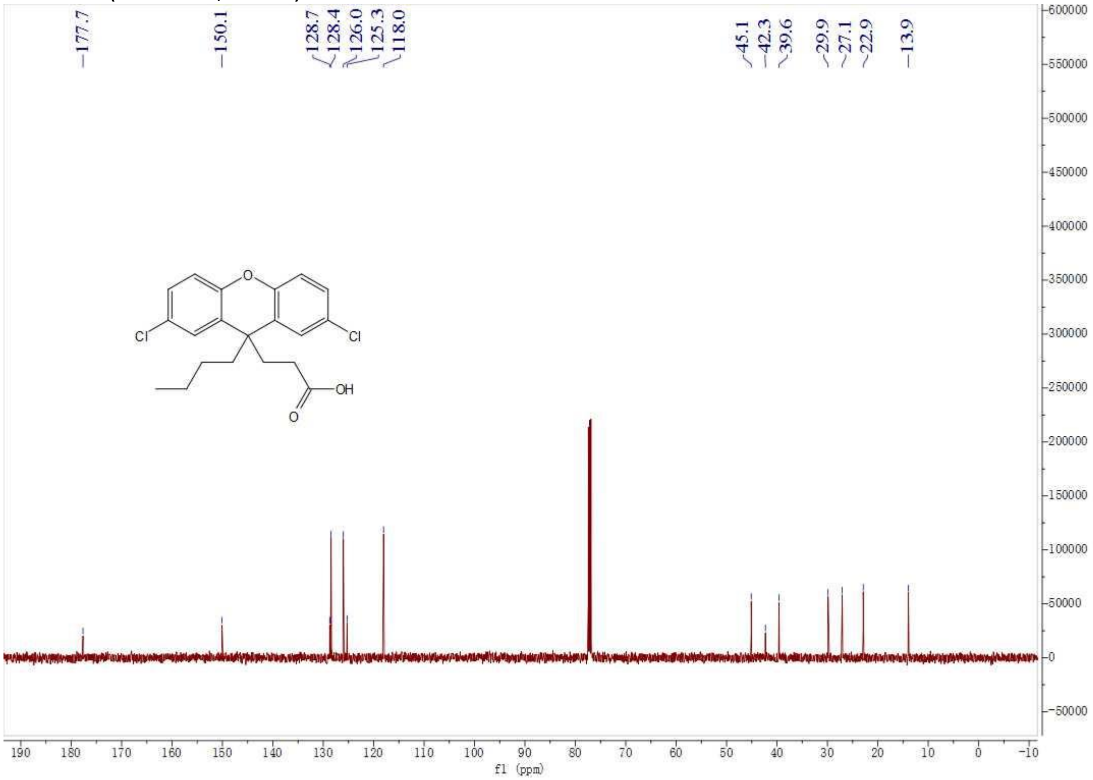

^{1}H NMR (500 MHz, $CDCl_3$)

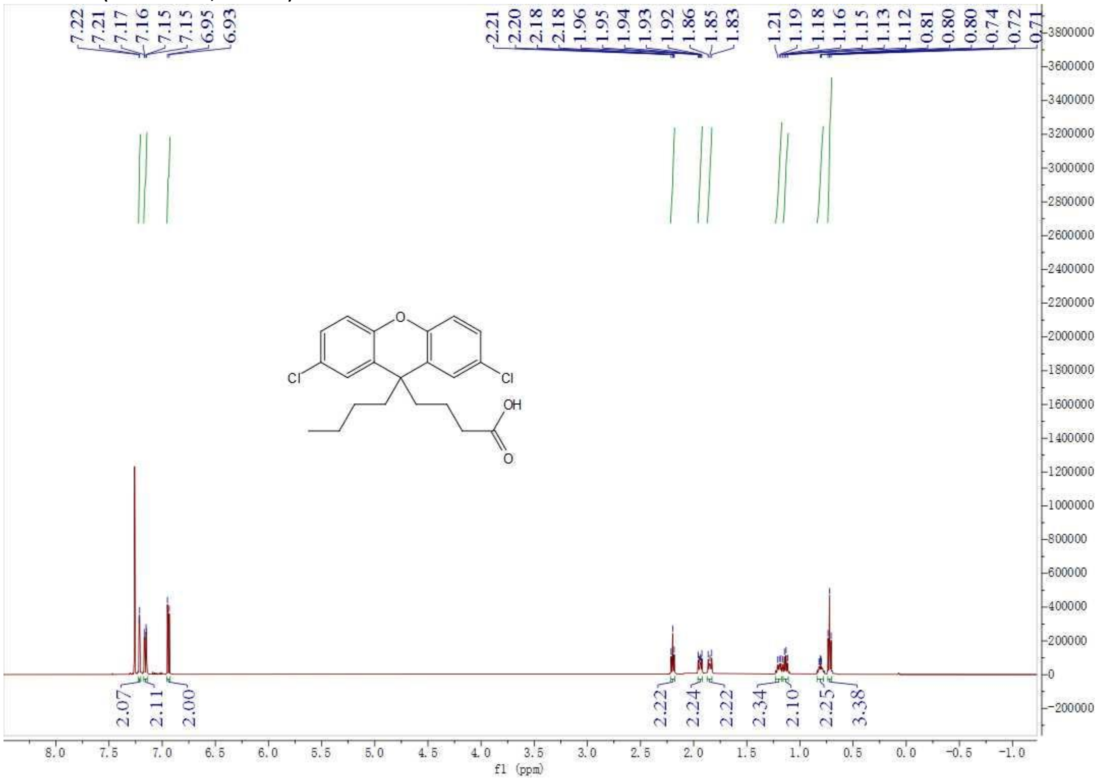

^{13}C NMR (126 MHz, $CDCl_3$)

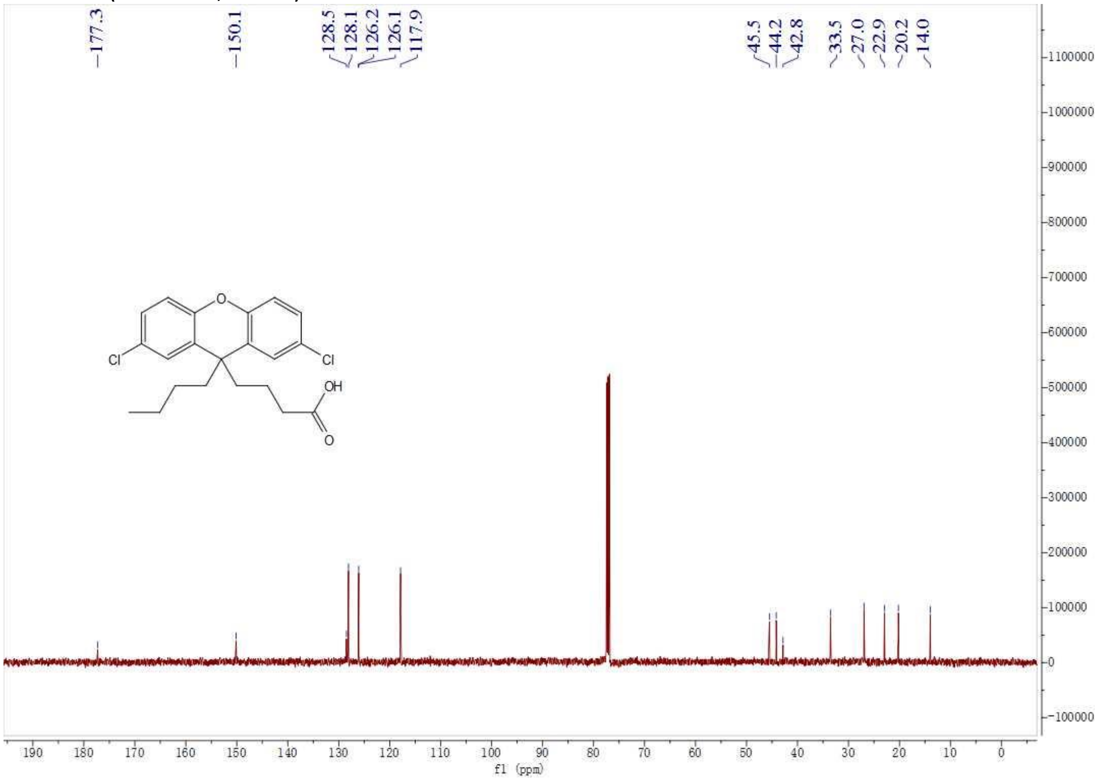

^{1}H NMR (500 MHz, $CDCl_3$)

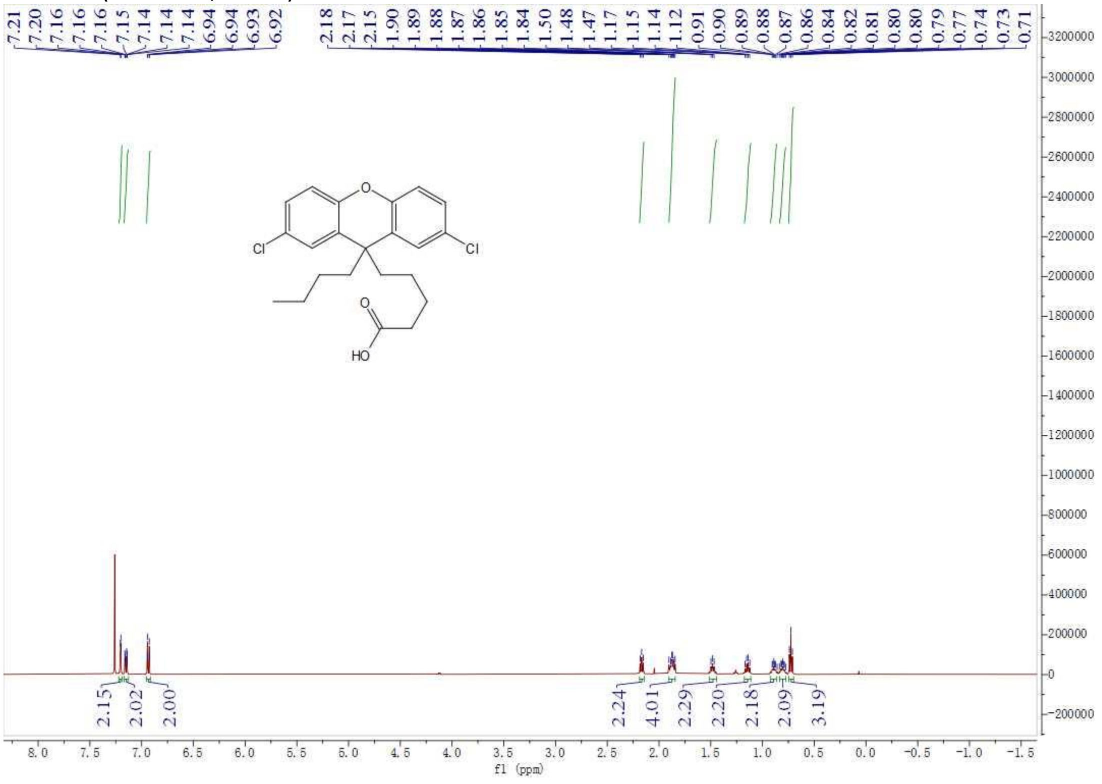

^{13}C NMR (126 MHz, $CDCl_3$)

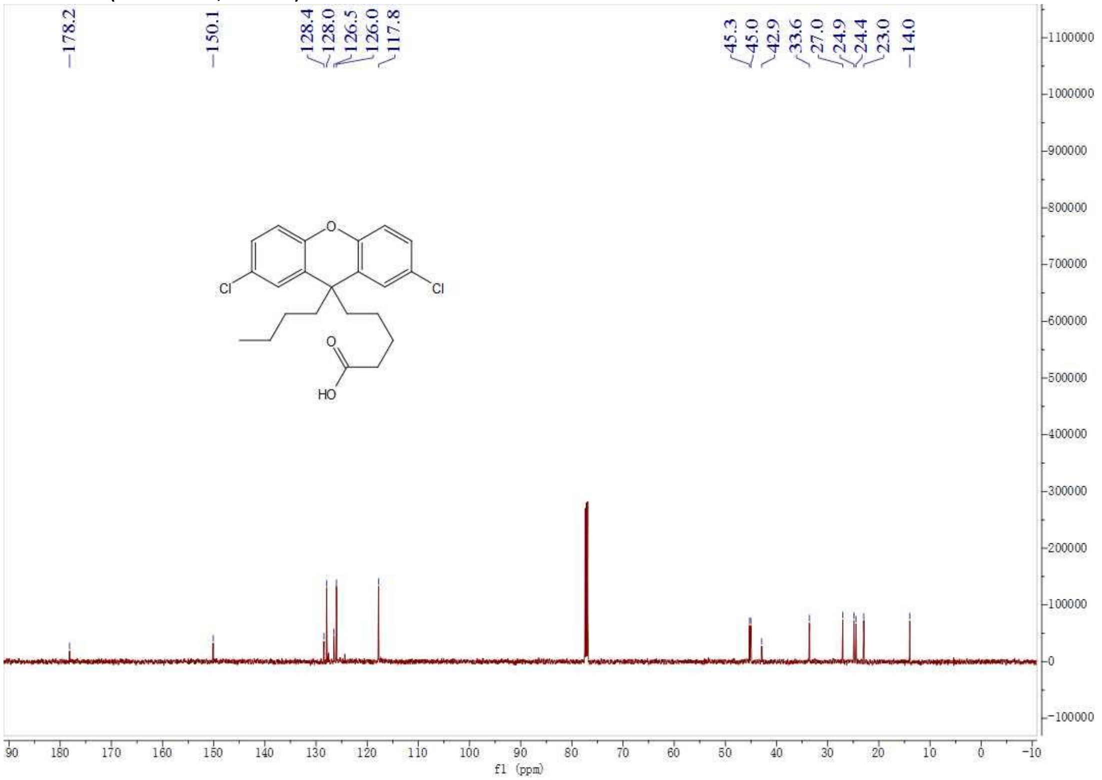

^{1}H NMR (500 MHz, $CDCl_3$)

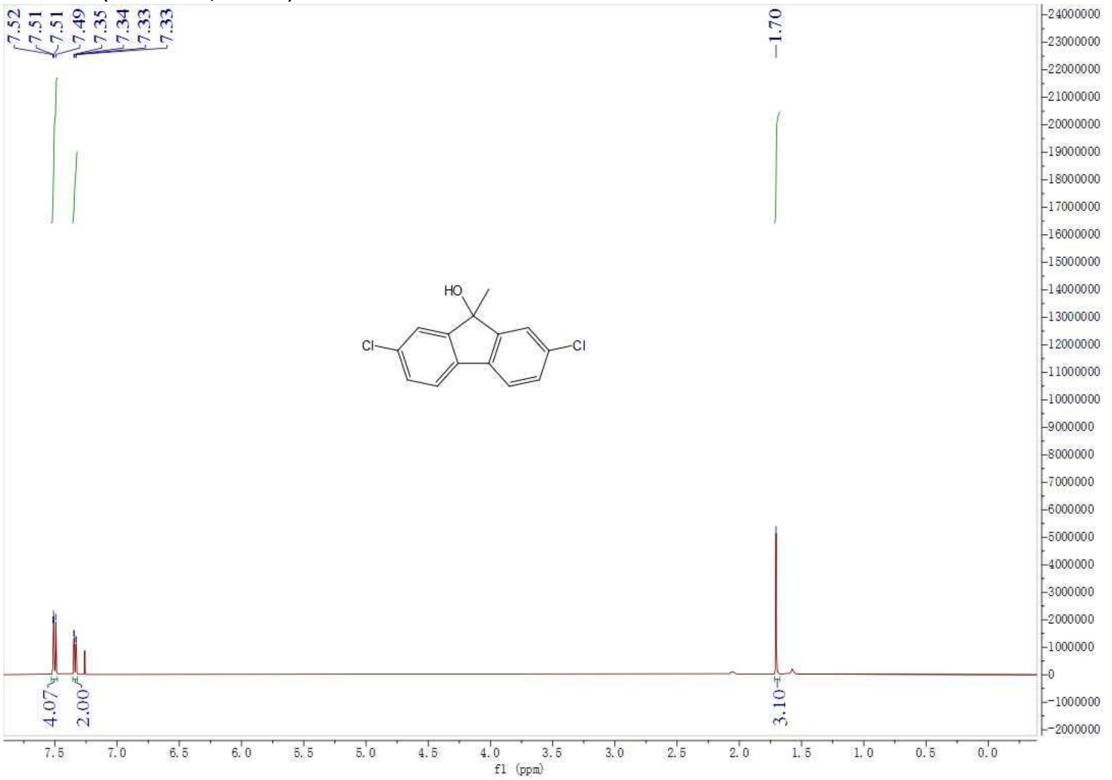

^{13}C NMR (126 MHz, $CDCl_3$)

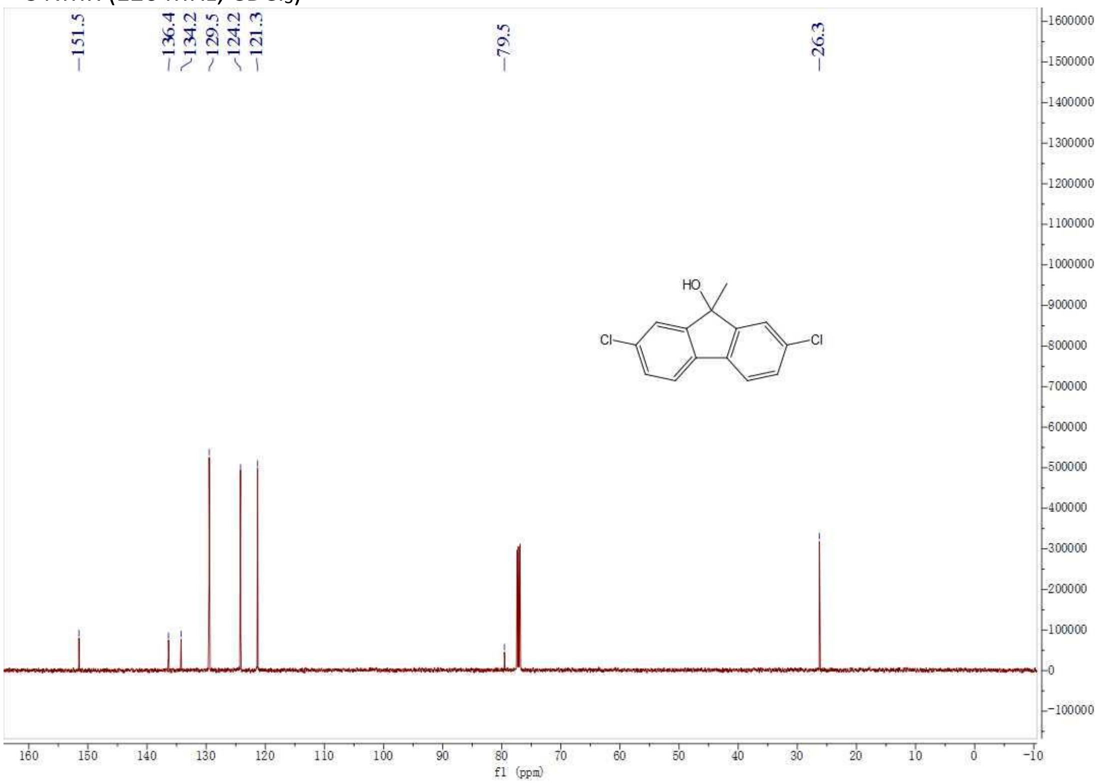

^{1}H NMR (500 MHz, $CDCl_3$)

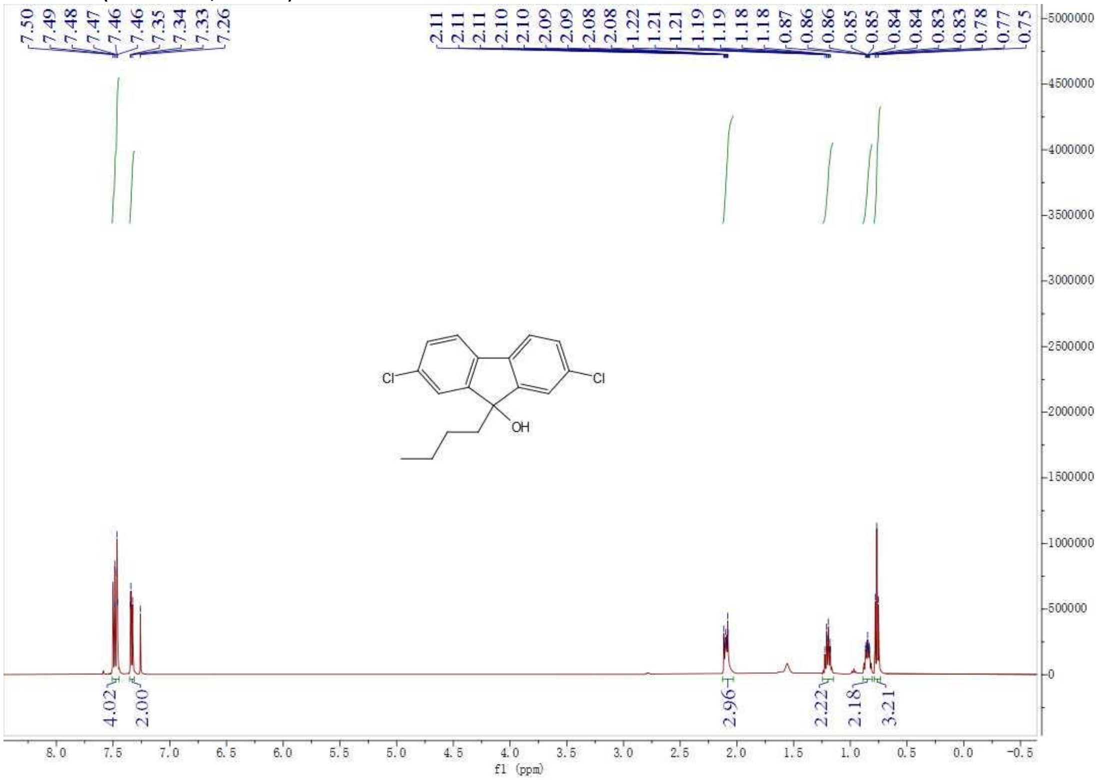

^{13}C NMR (126 MHz, $CDCl_3$)

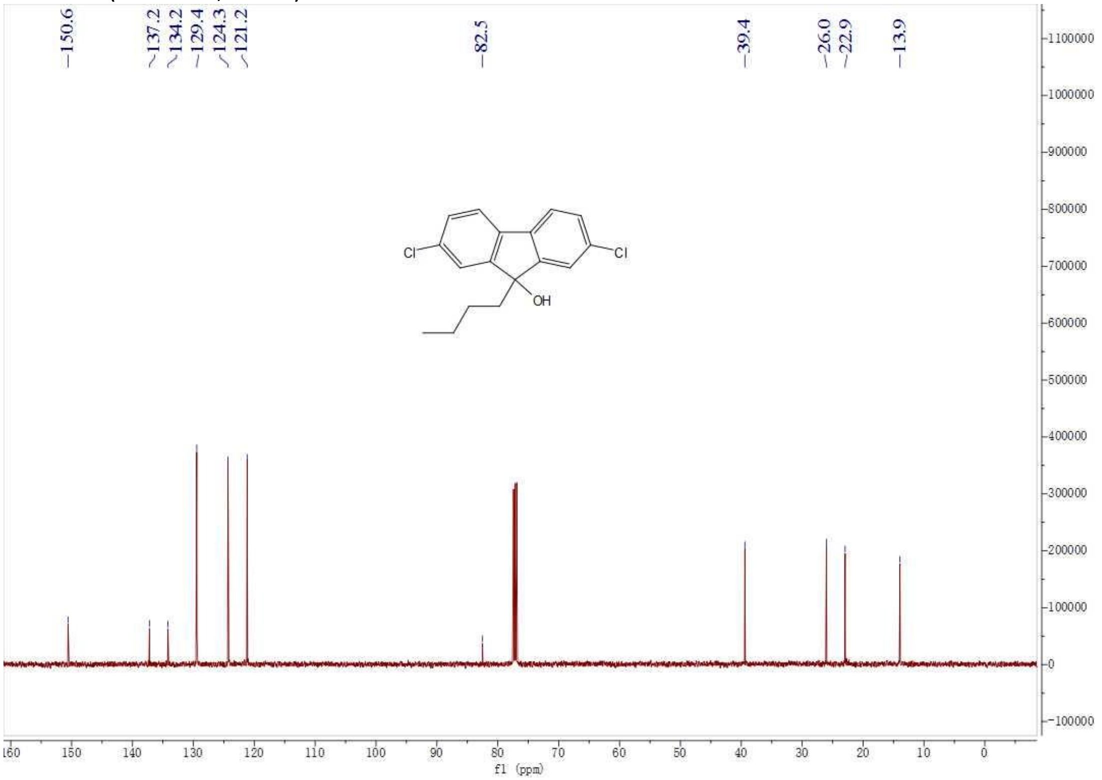

^1H NMR (500 MHz, $CDCl_3$)

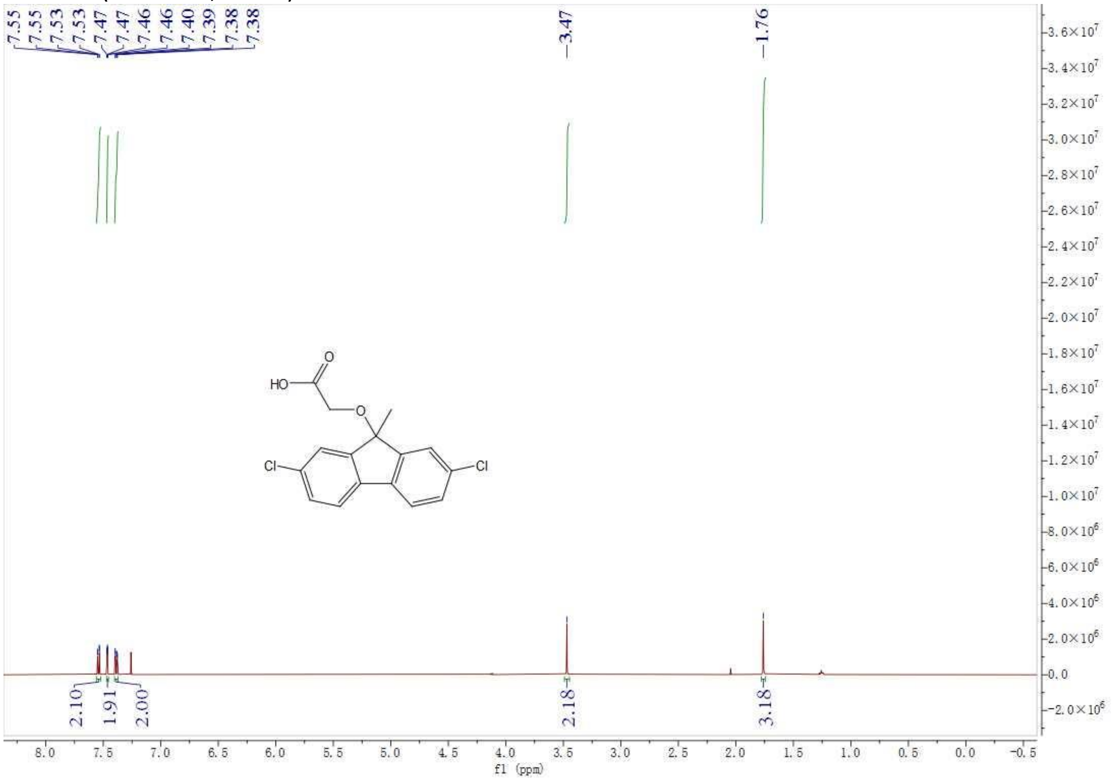

^{13}C NMR (126 MHz, $CDCl_3$)

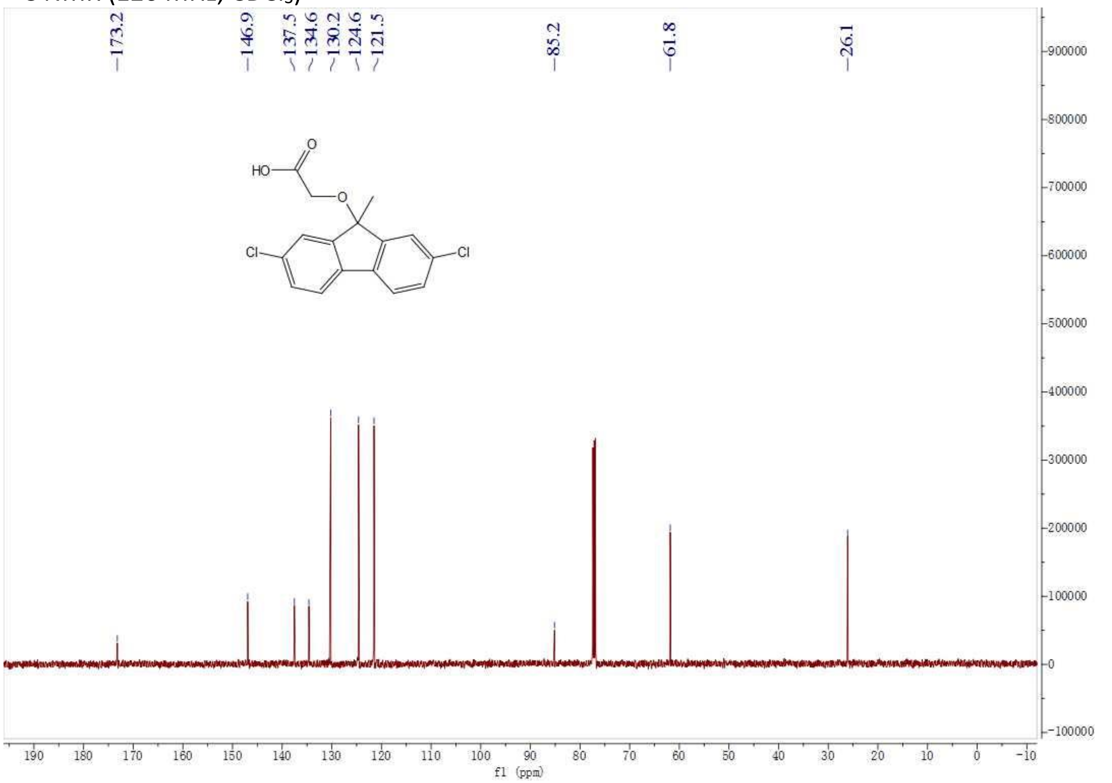

^{1}H NMR (500 MHz, $CDCl_3$)

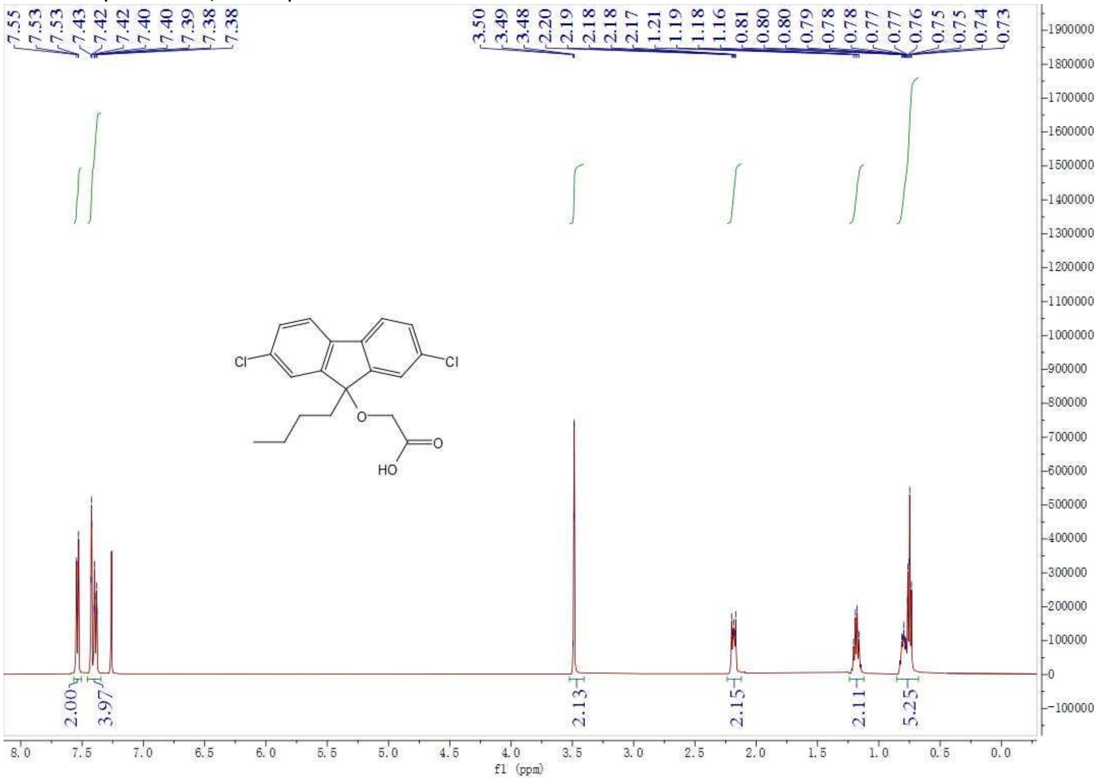

^{13}C NMR (126 MHz, $CDCl_3$)

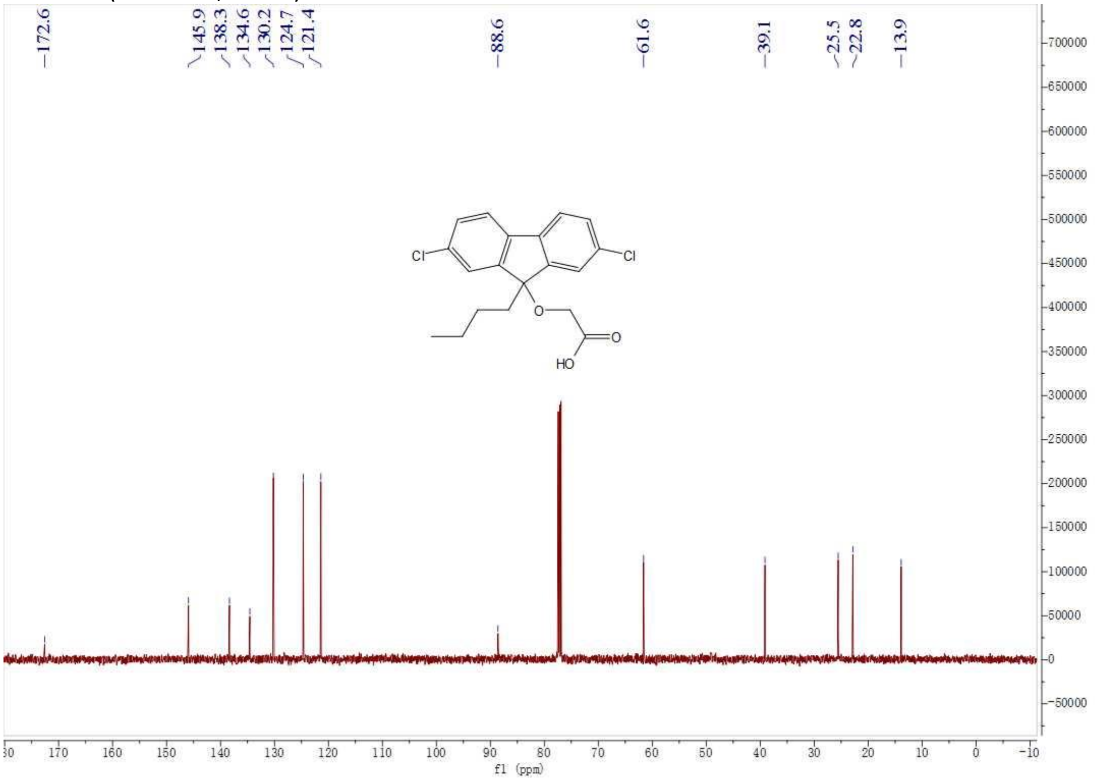

^{1}H NMR (500 MHz, $CDCl_3$)-**57**

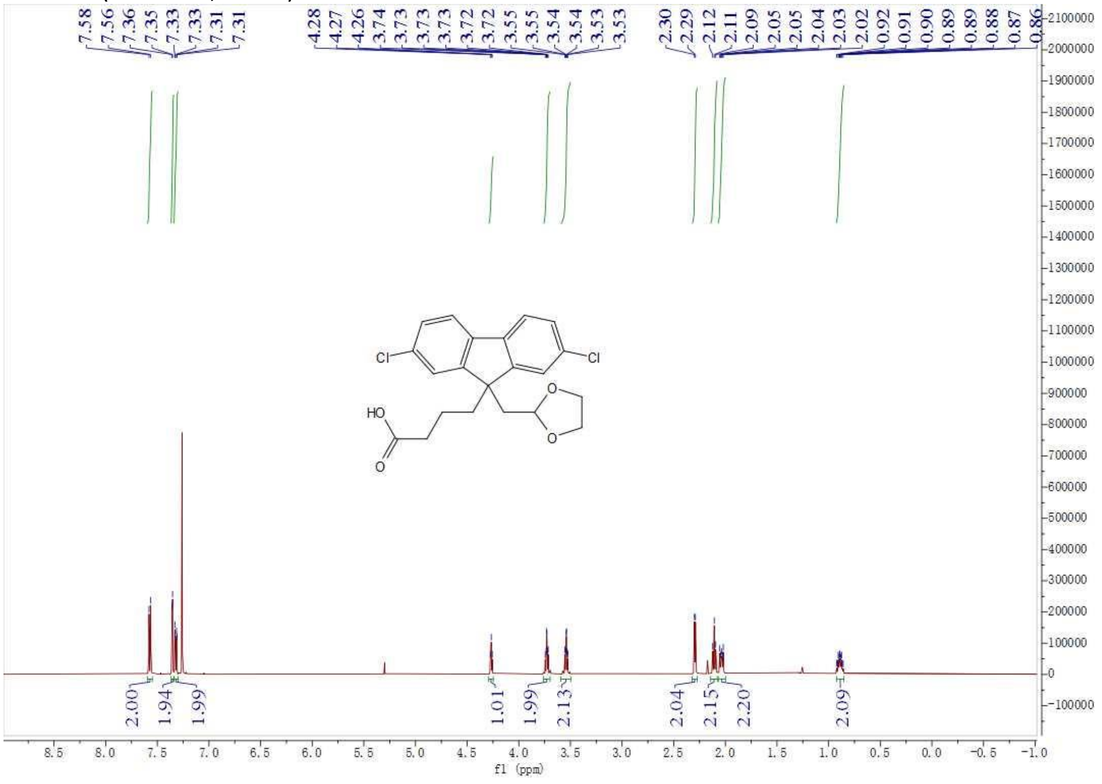

^{13}C NMR (101 MHz, $CDCl_3$)-**57**

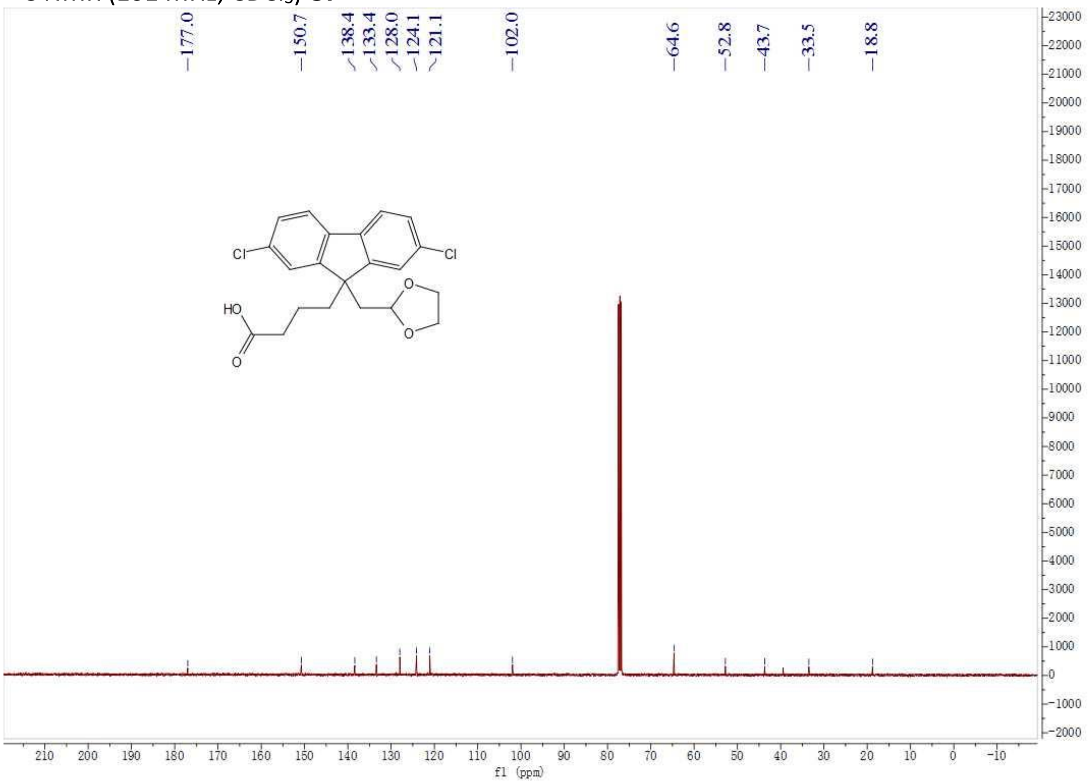

^{1}H NMR (500 MHz, $CDCl_3$)-**1**

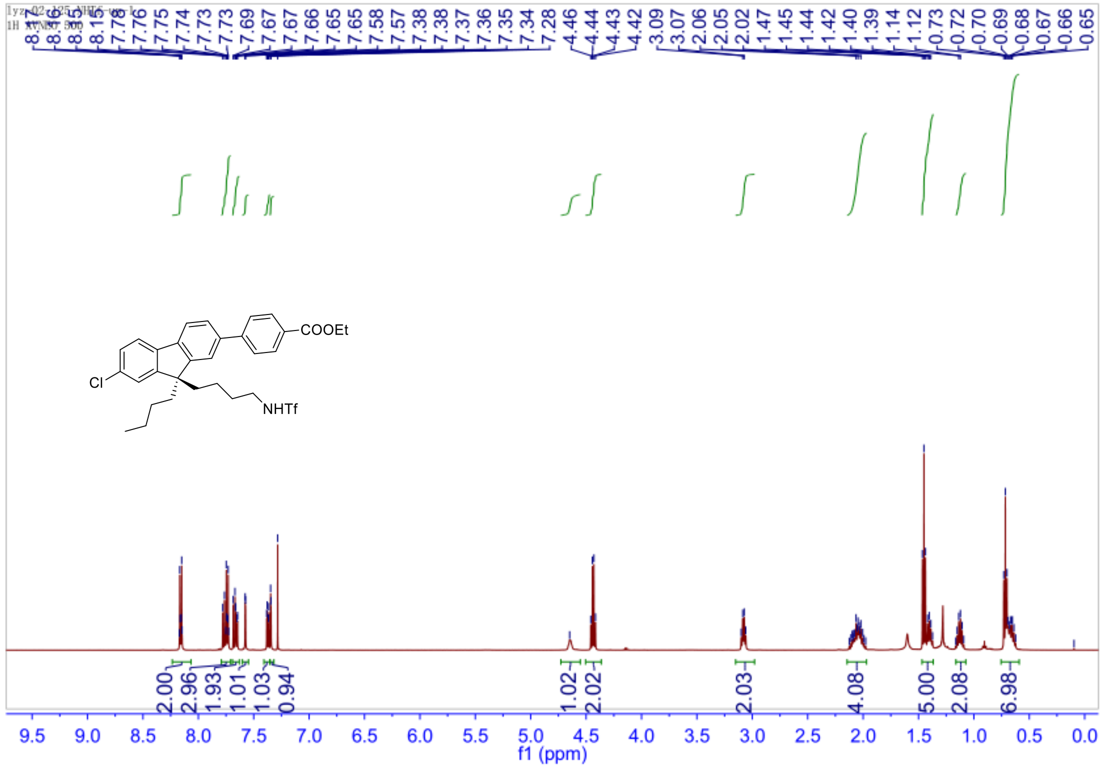

^{13}C NMR (126 MHz, $CDCl_3$)-**1**

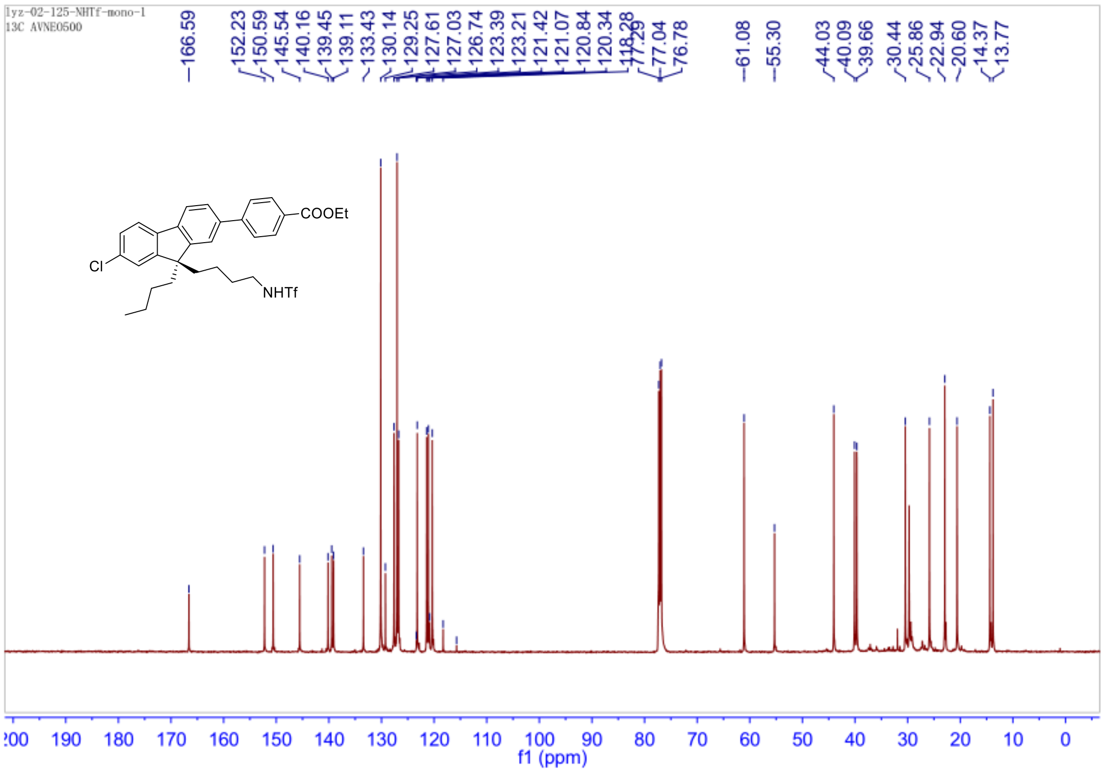

^{19}F NMR (471 MHz, $CDCl_3$)-**1**

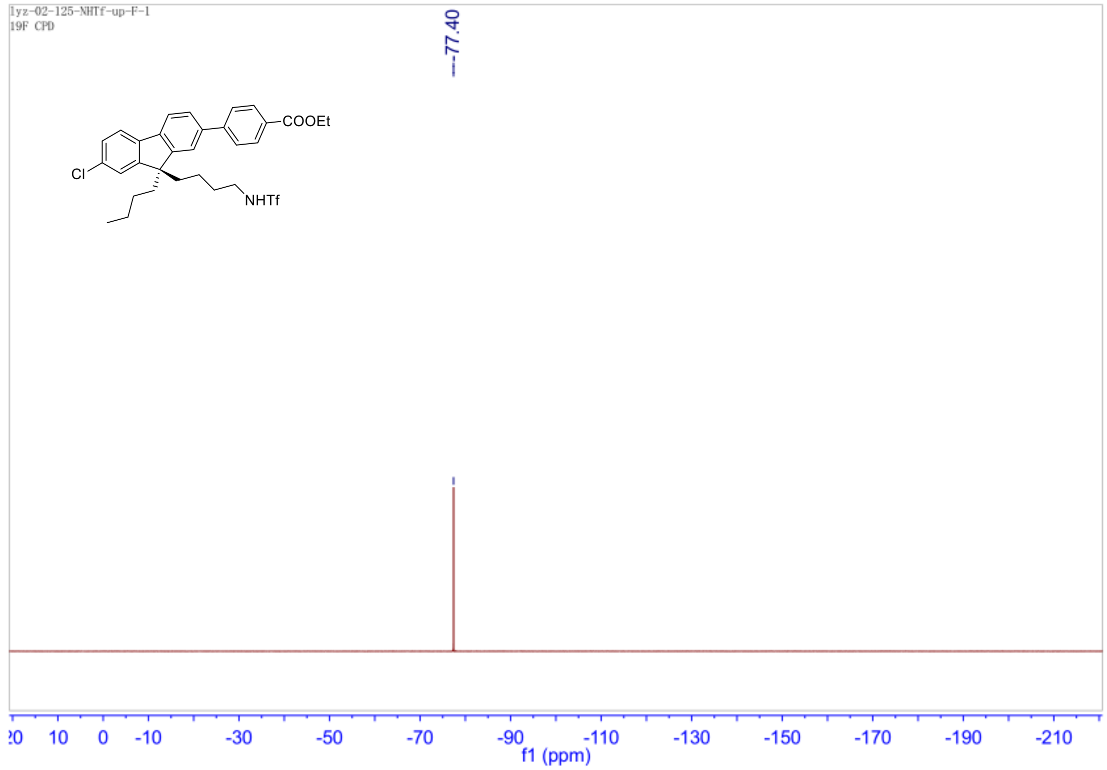

^{1}H NMR (500 MHz, $CDCl_3$) **-2**

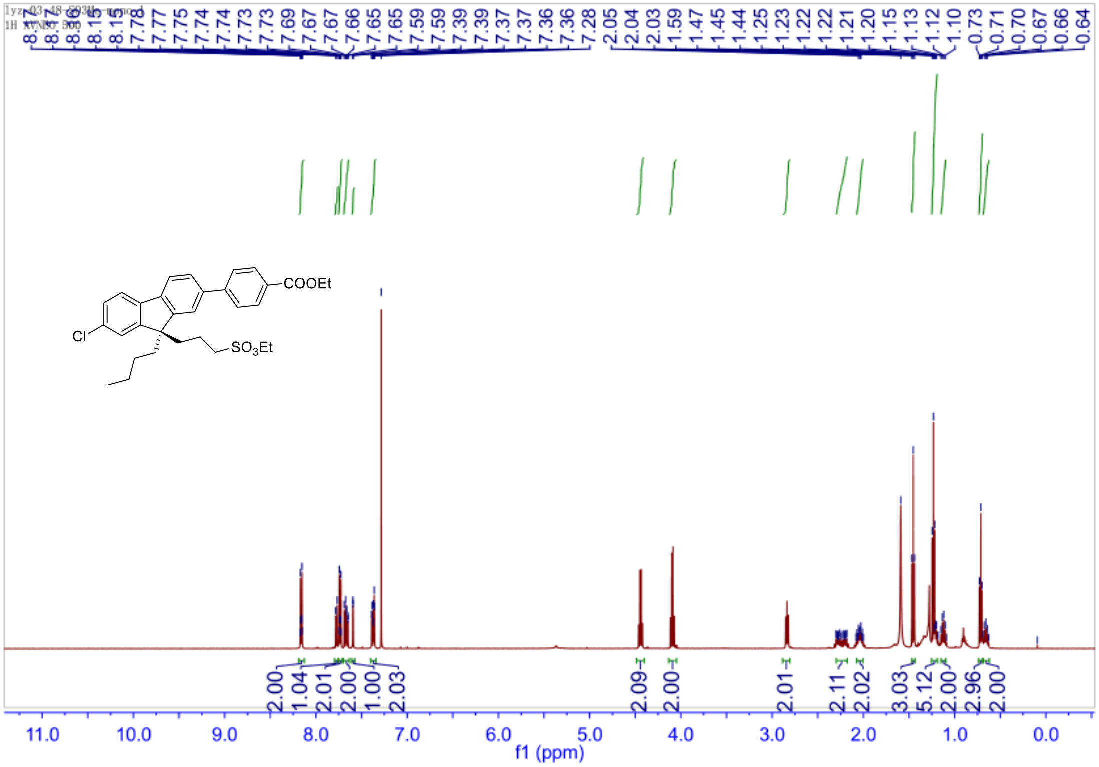

^{13}C NMR (126 MHz, $CDCl_3$) **-2**

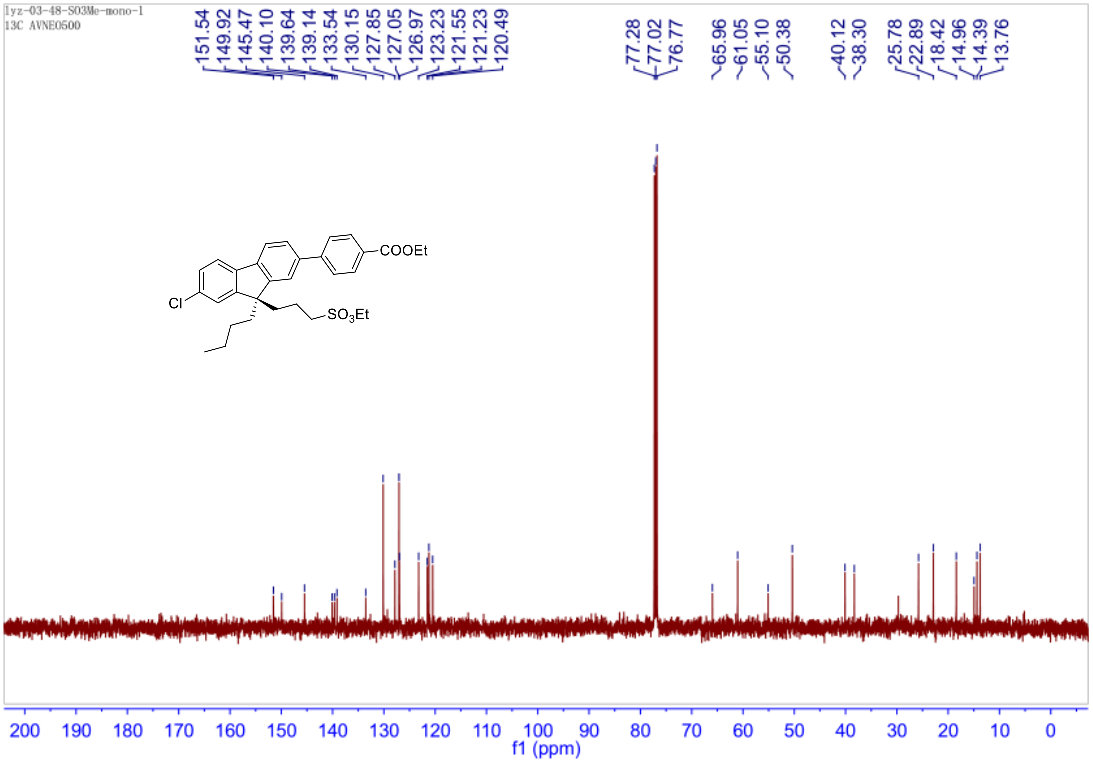

^{1}H NMR (500 MHz, $CDCl_3$) **-3**

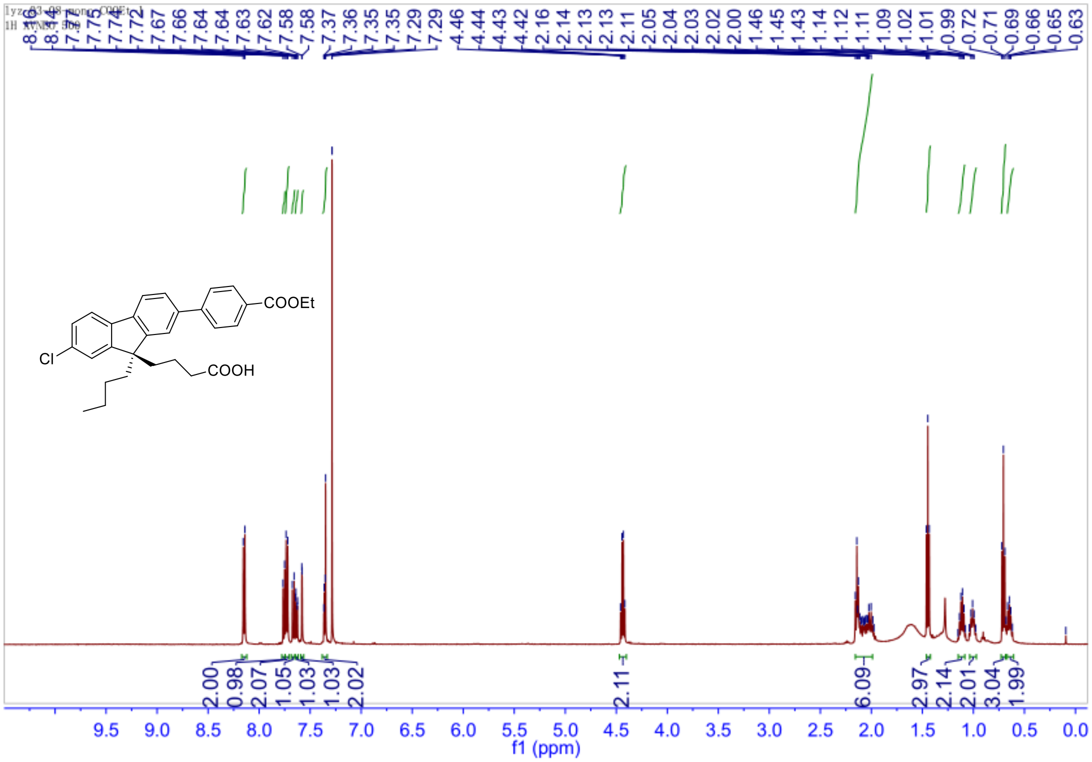

^{13}C NMR (126 MHz, $CDCl_3$) **-3**

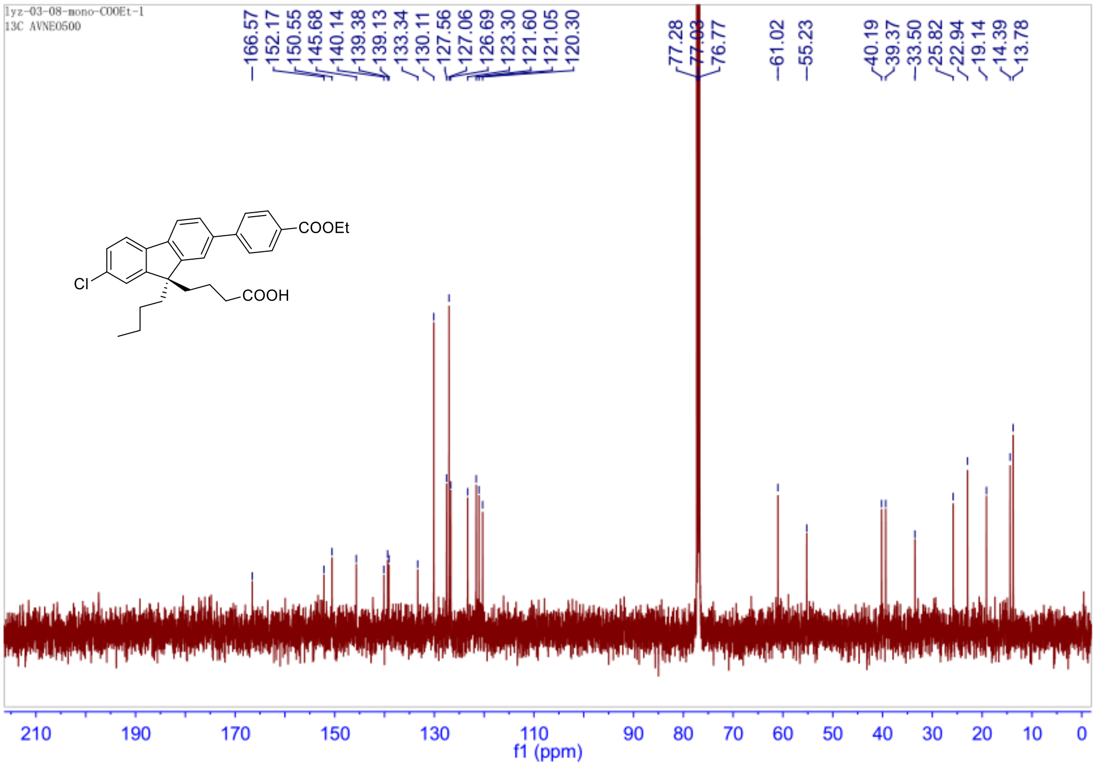

^{1}H NMR (500 MHz, $CDCl_3$) **-4**

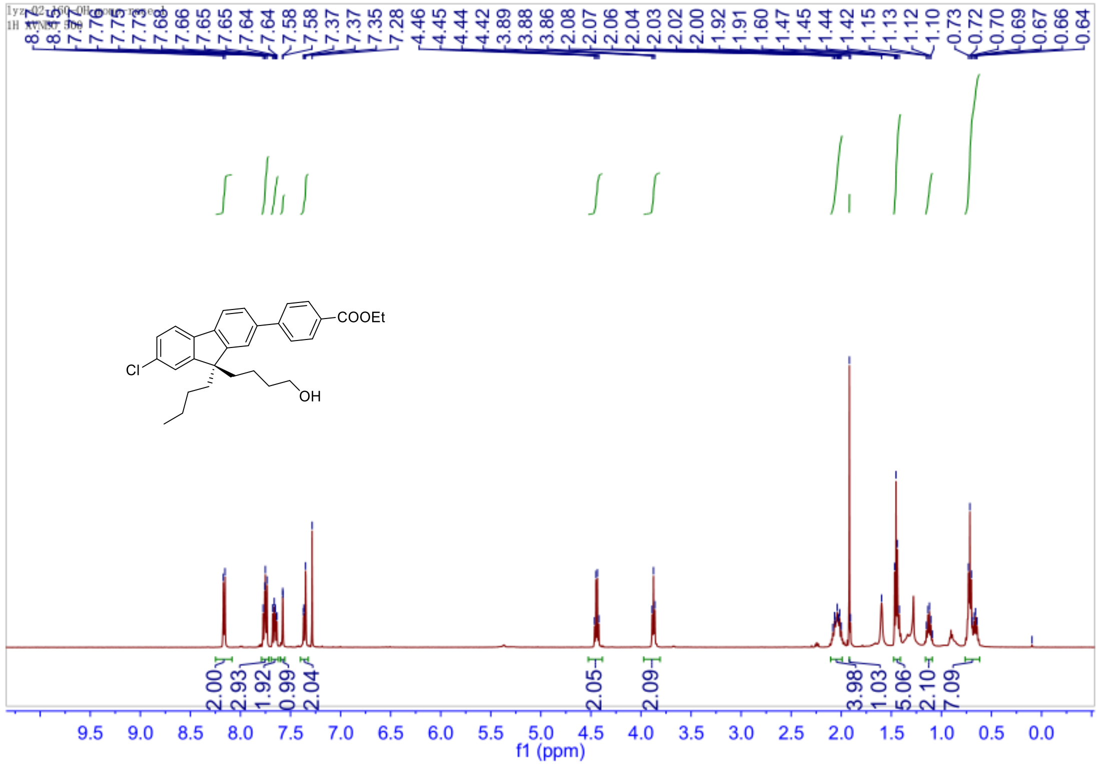

^{13}C NMR (126 MHz, $CDCl_3$)-**4**

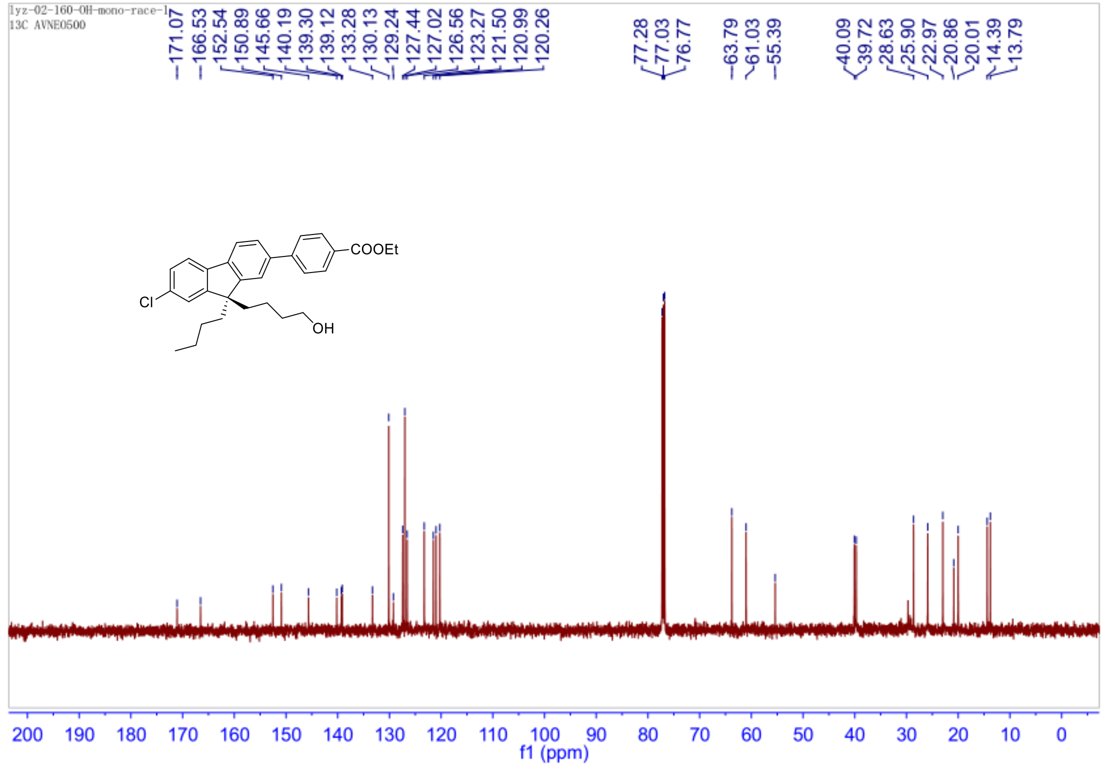

^{1}H NMR (500 MHz, $CDCl_3$) **-5**

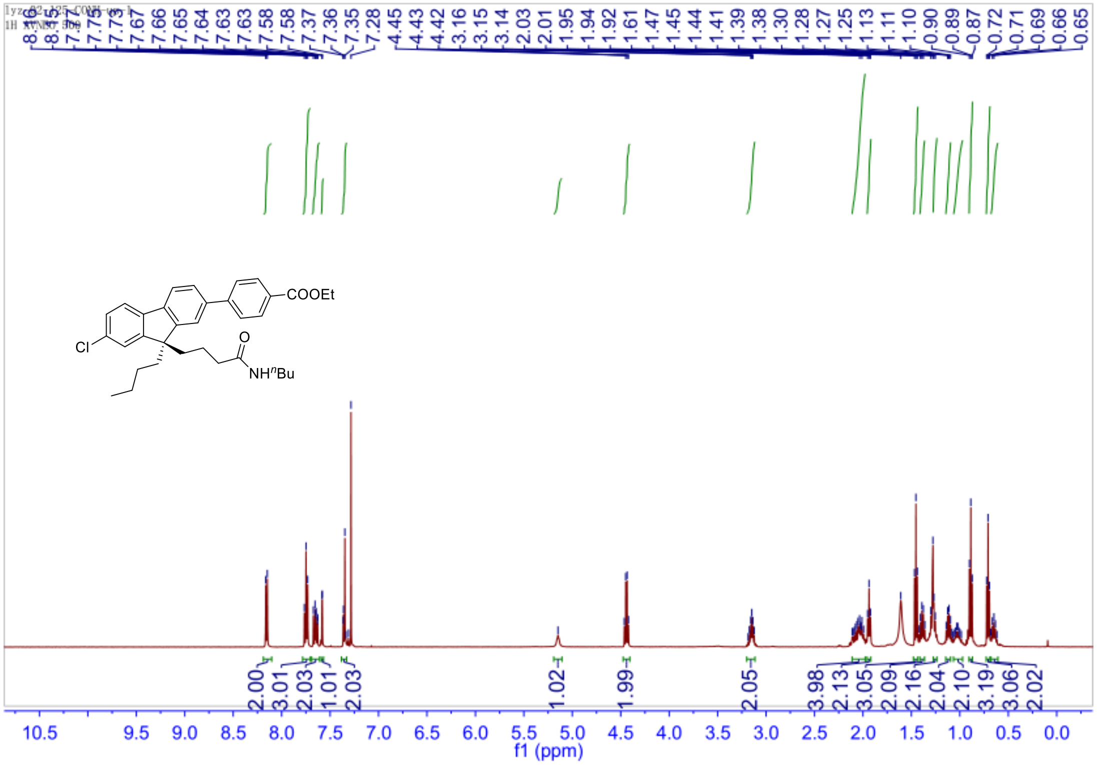

^{13}C NMR (126 MHz, $CDCl_3$) **-5**

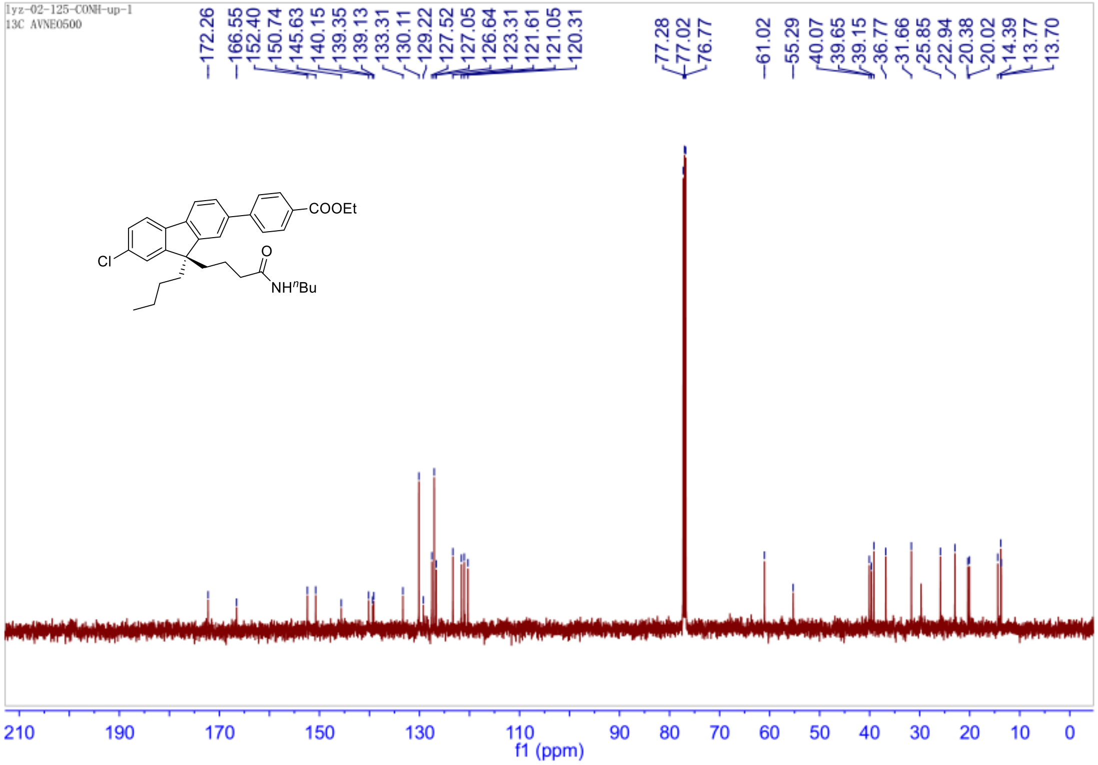

^{1}H NMR (500 MHz, $CDCl_3$) **-6**

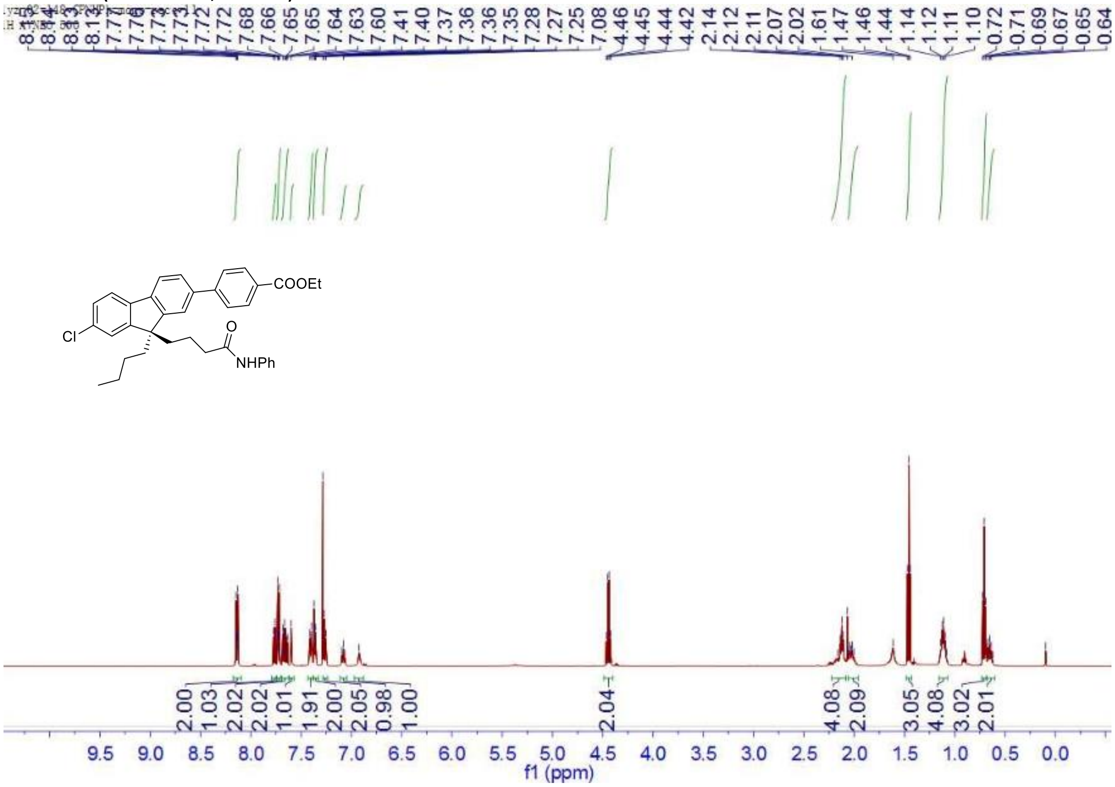

^{13}C NMR (126 MHz, $CDCl_3$) **-6**

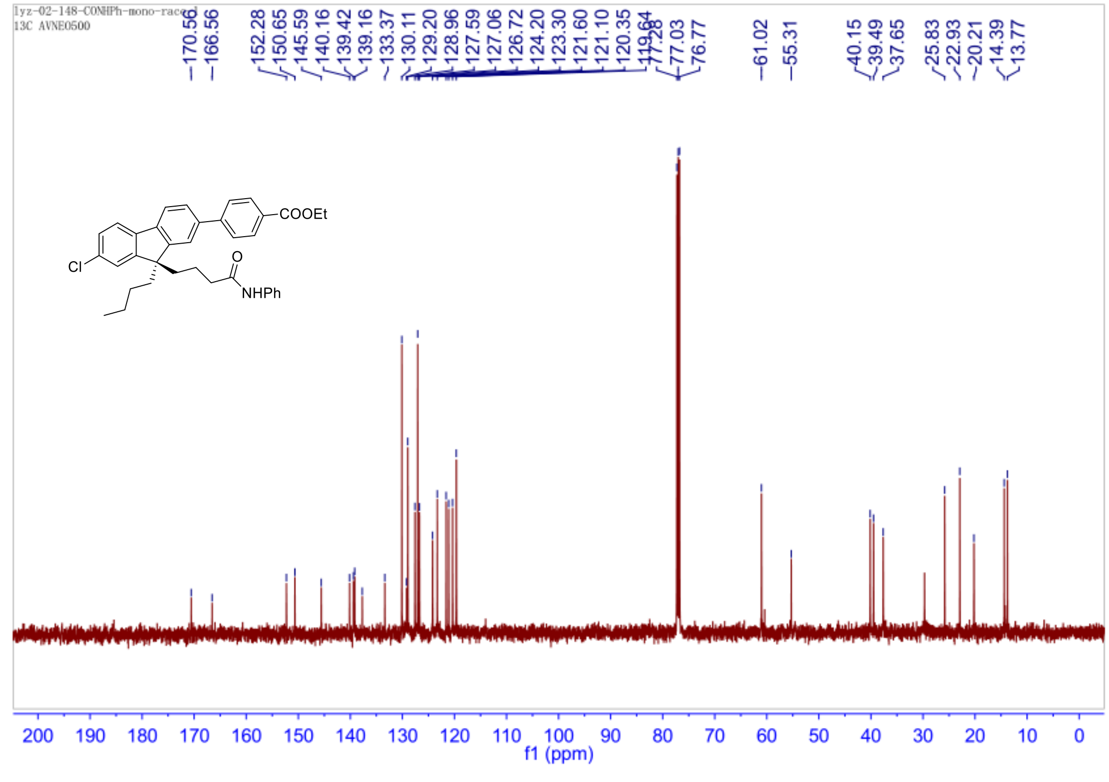

^1H NMR (500 MHz, $CDCl_3$) **-7**

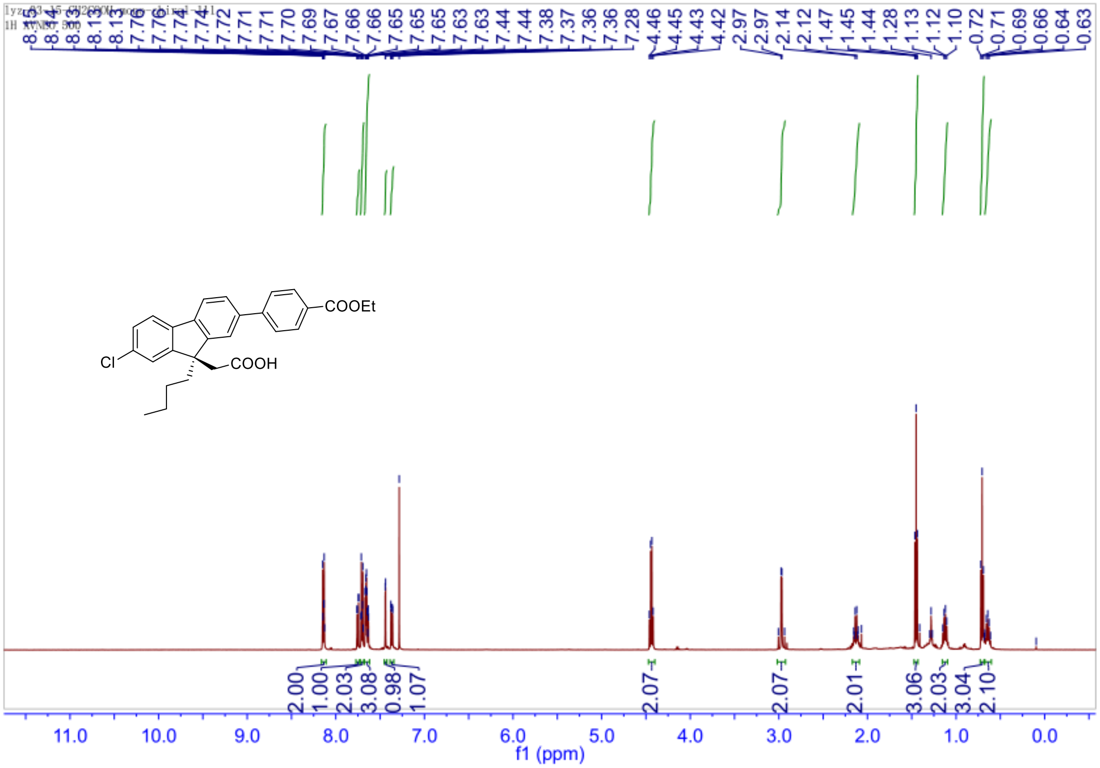

^{13}C NMR (126 MHz, $CDCl_3$) **-7**

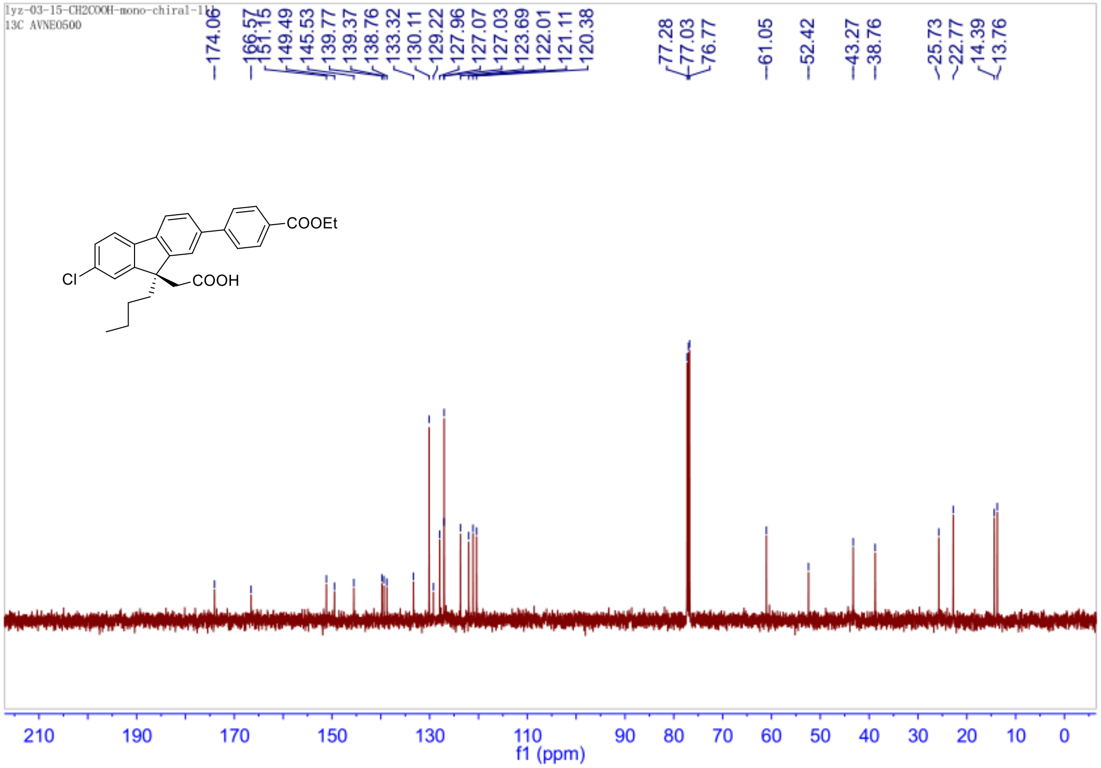

^{1}H NMR (500 MHz, $CDCl_3$) **-8**

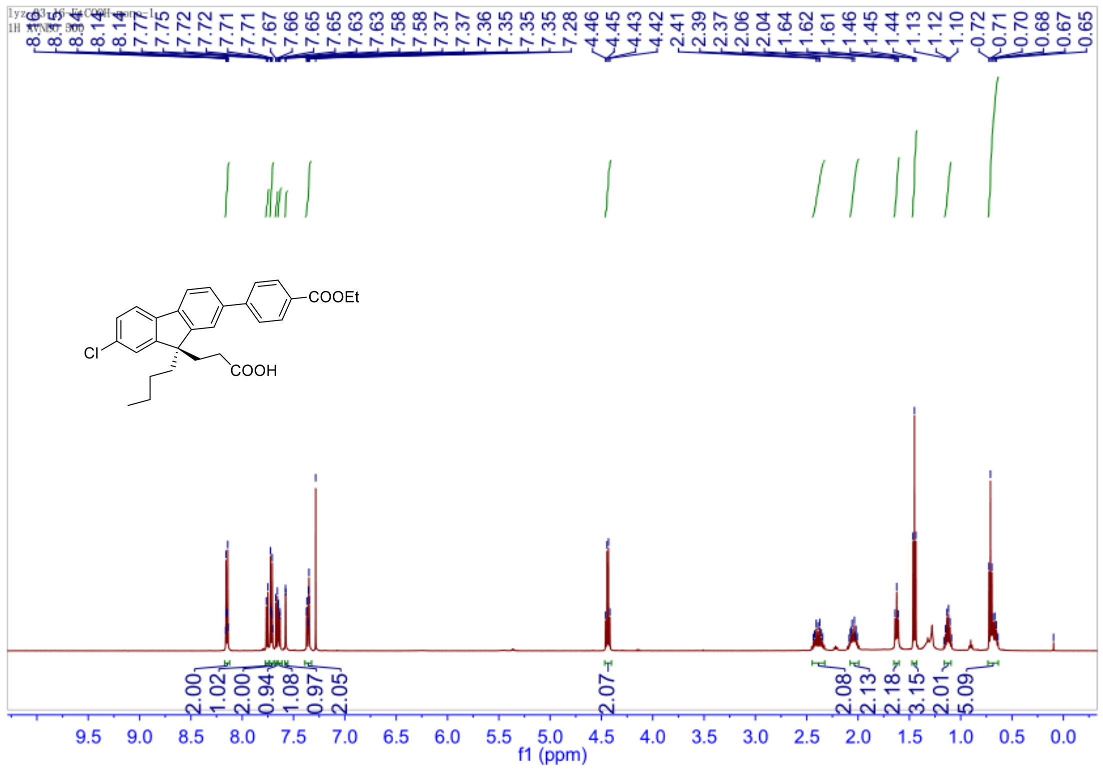

^{13}C NMR (126 MHz, $CDCl_3$) **-8**

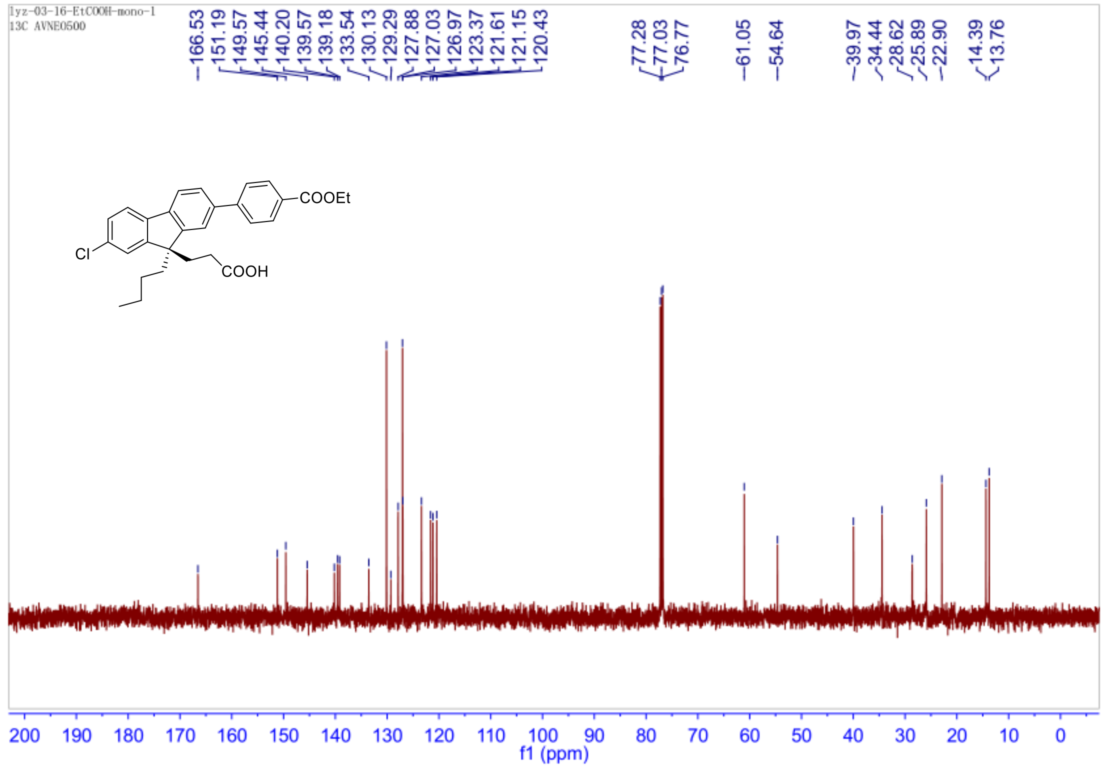

^{1}H NMR (300 MHz, $CDCl_3$) **-9**

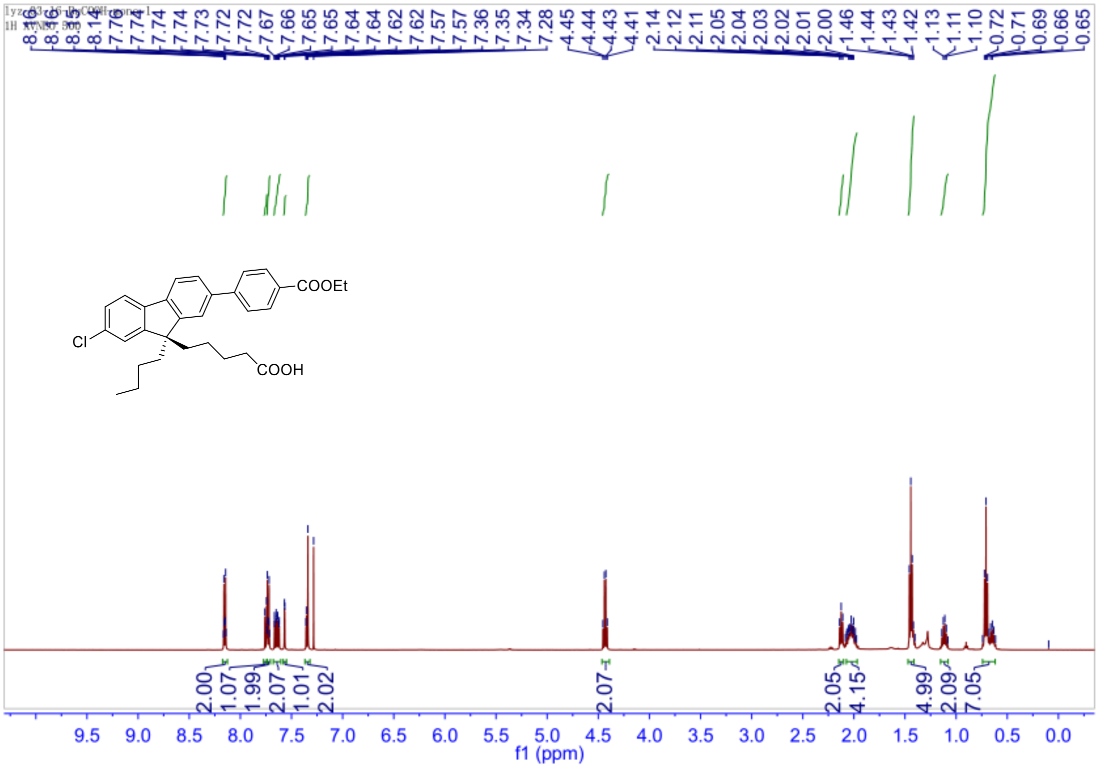

^{13}C NMR (75 MHz, $CDCl_3$) **-9**

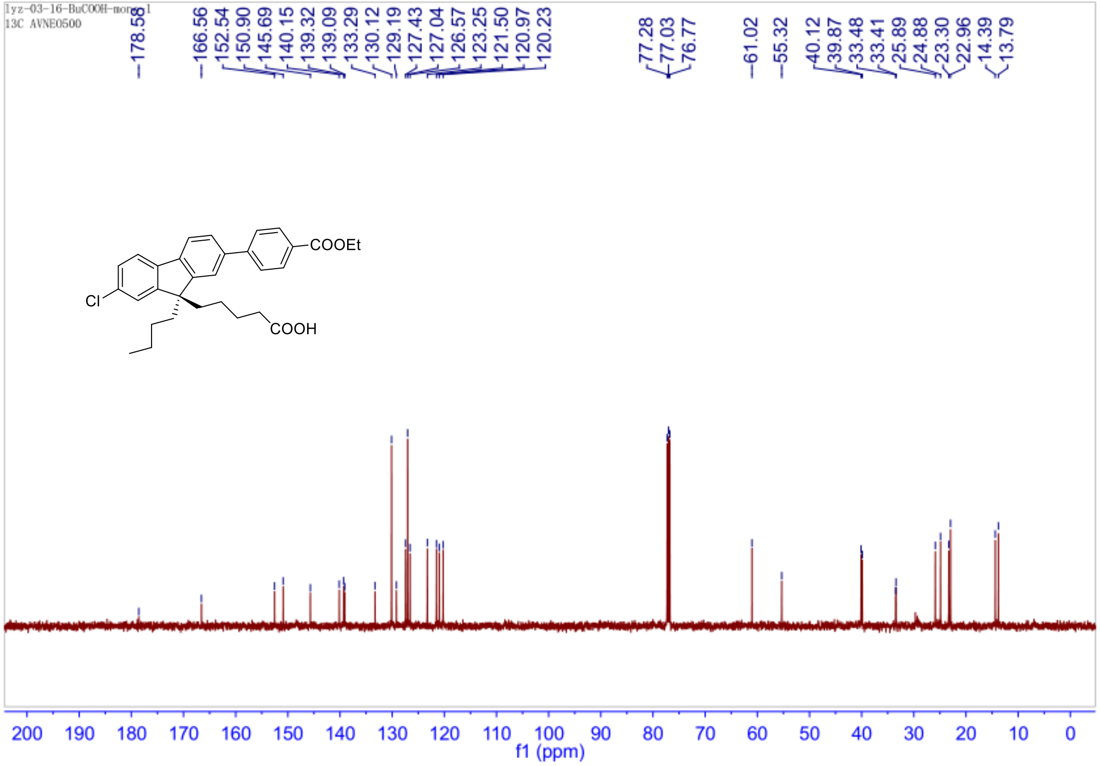

^{1}H NMR (500 MHz, $CDCl_3$) **-10**

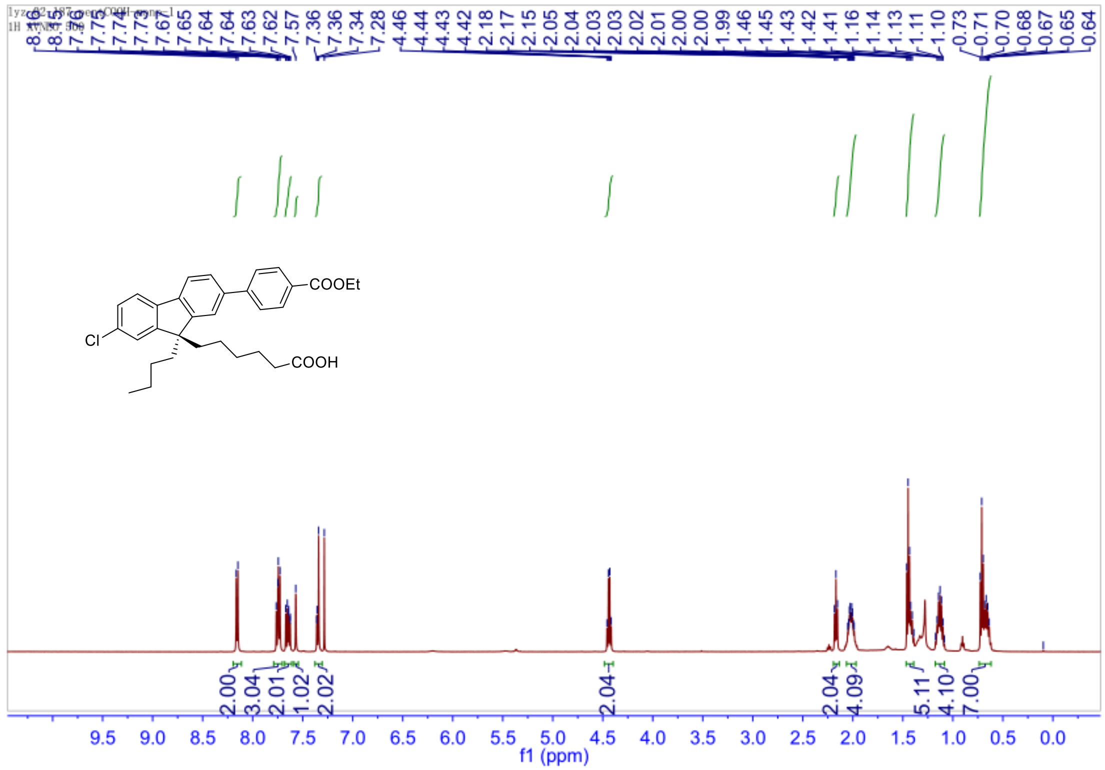

^{13}C NMR (126 MHz, $CDCl_3$) **-10**

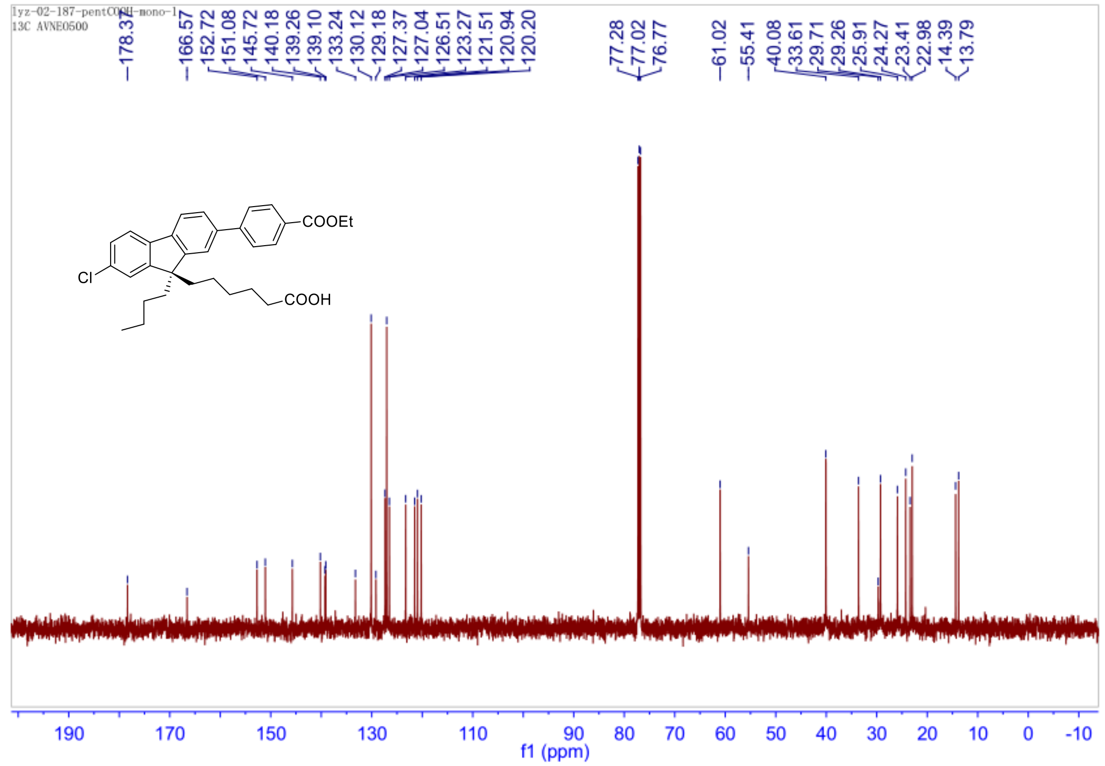

^{1}H NMR (500 MHz, $CDCl_3$) **-11**

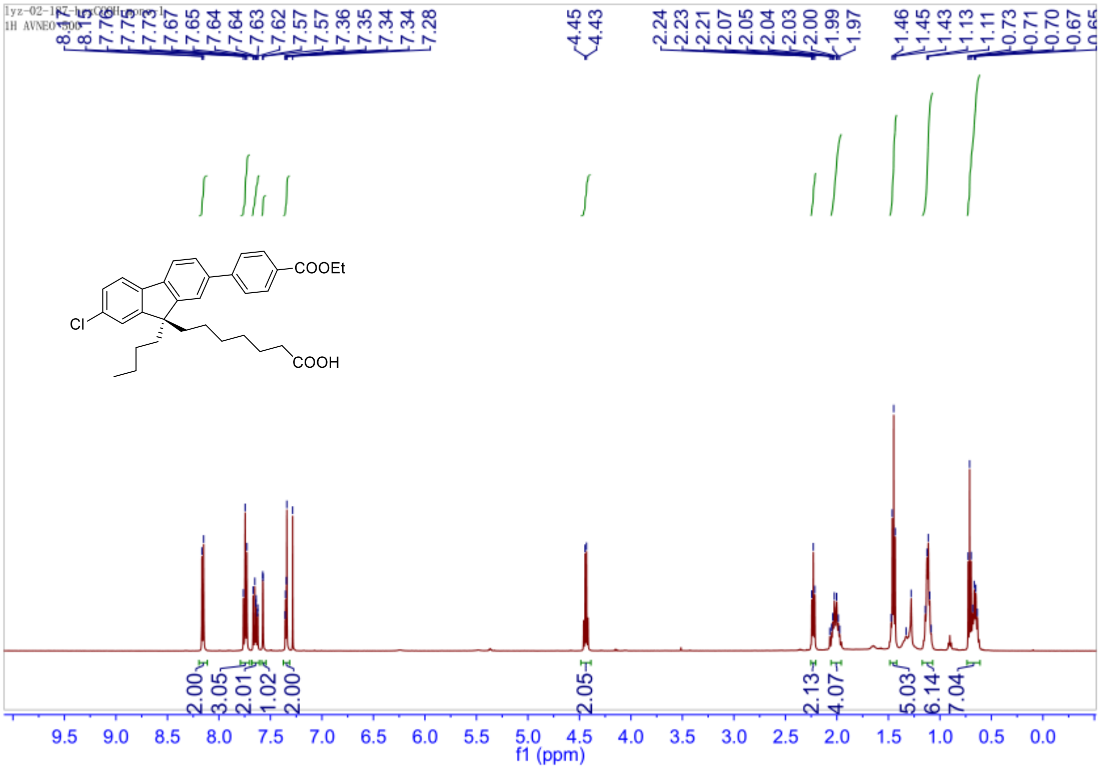

^{13}C NMR (126 MHz, $CDCl_3$) **-11**

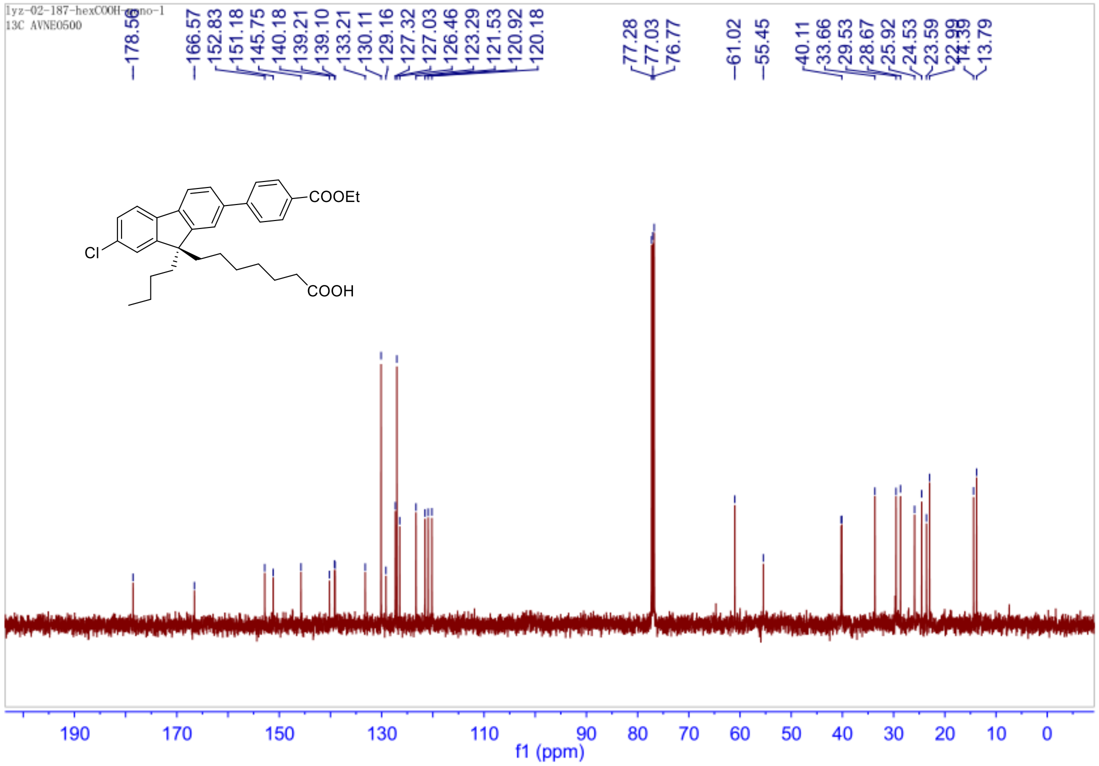

^{1}H NMR (500 MHz, $CDCl_3$) **-12**

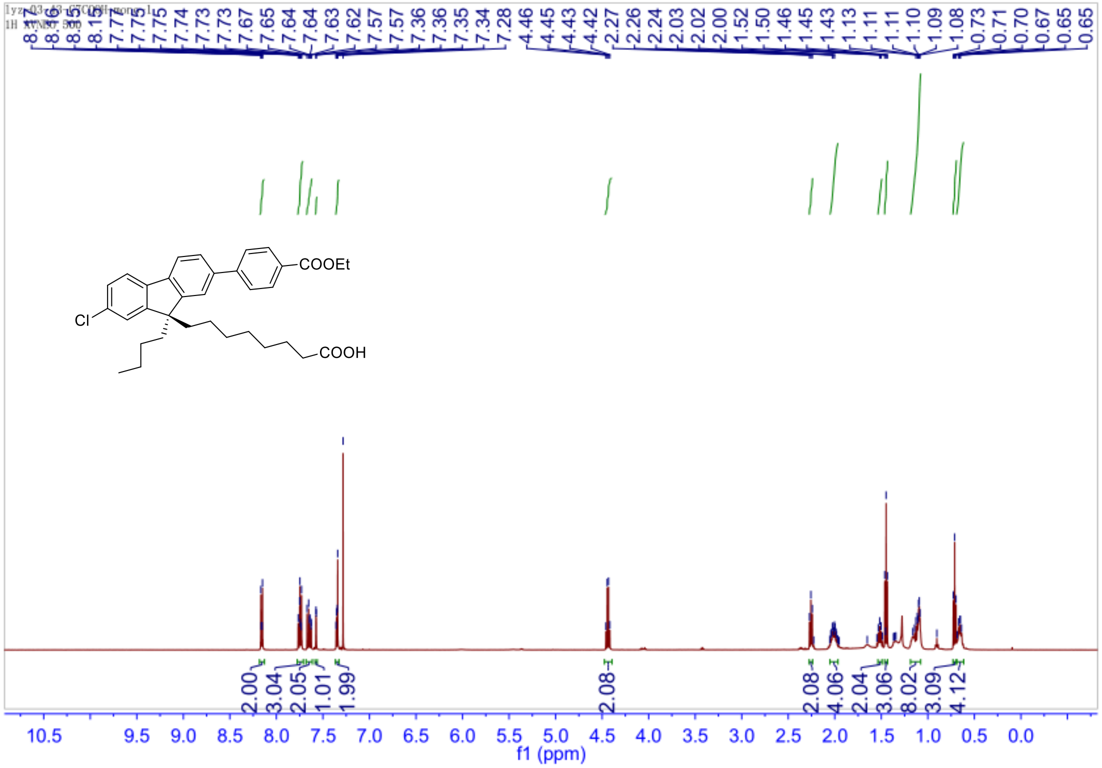

^{13}C NMR (126 MHz, $CDCl_3$) **-12**

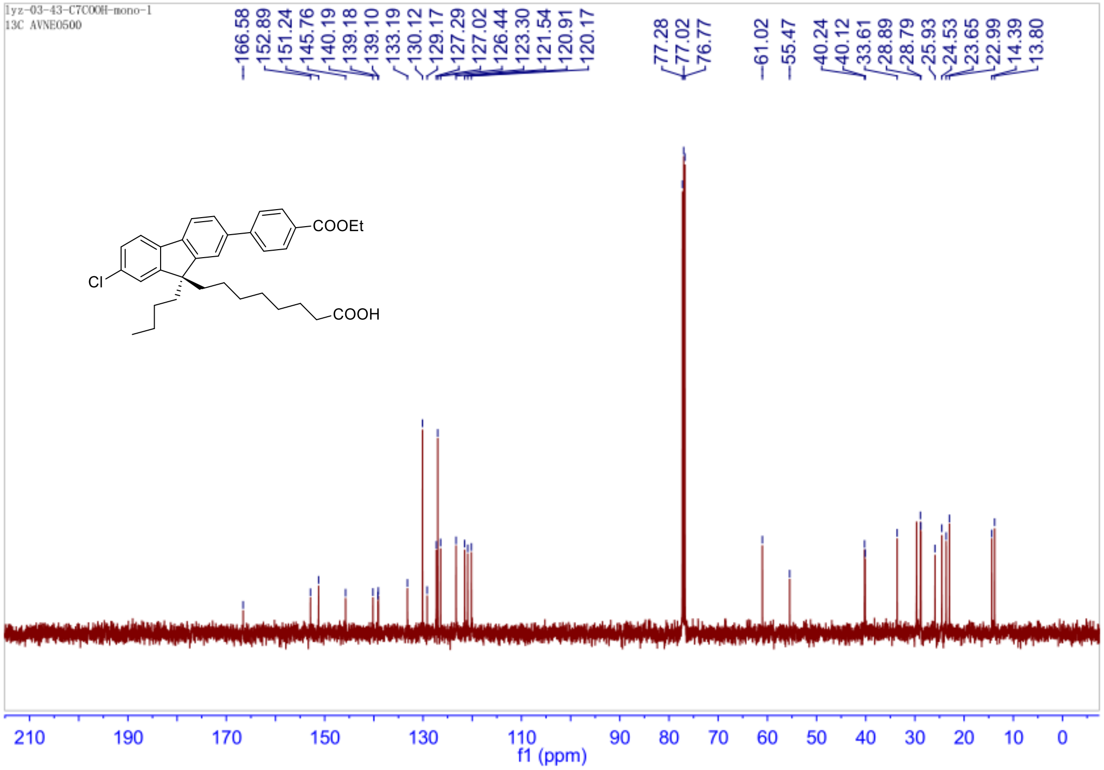

^{1}H NMR (500 MHz, $CDCl_3$) **-13**

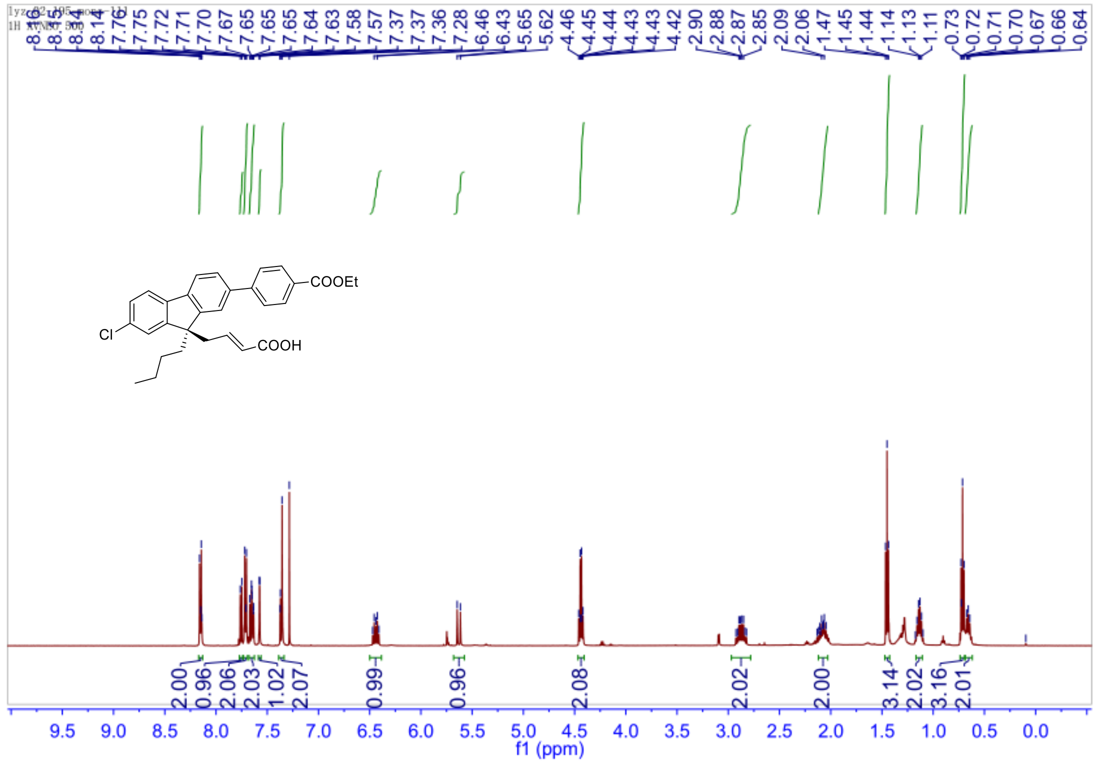

^{13}C NMR (126 MHz, $CDCl_3$) **-13**

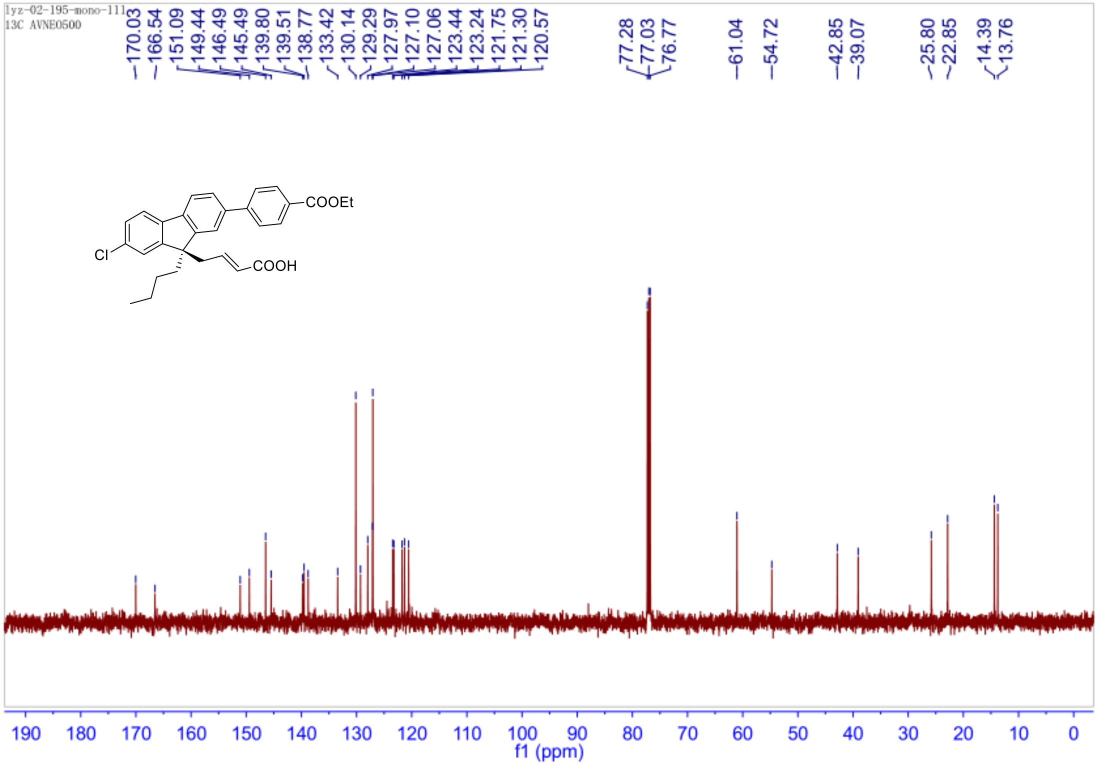

^{1}H NMR (500 MHz, $CDCl_3$) **-14**

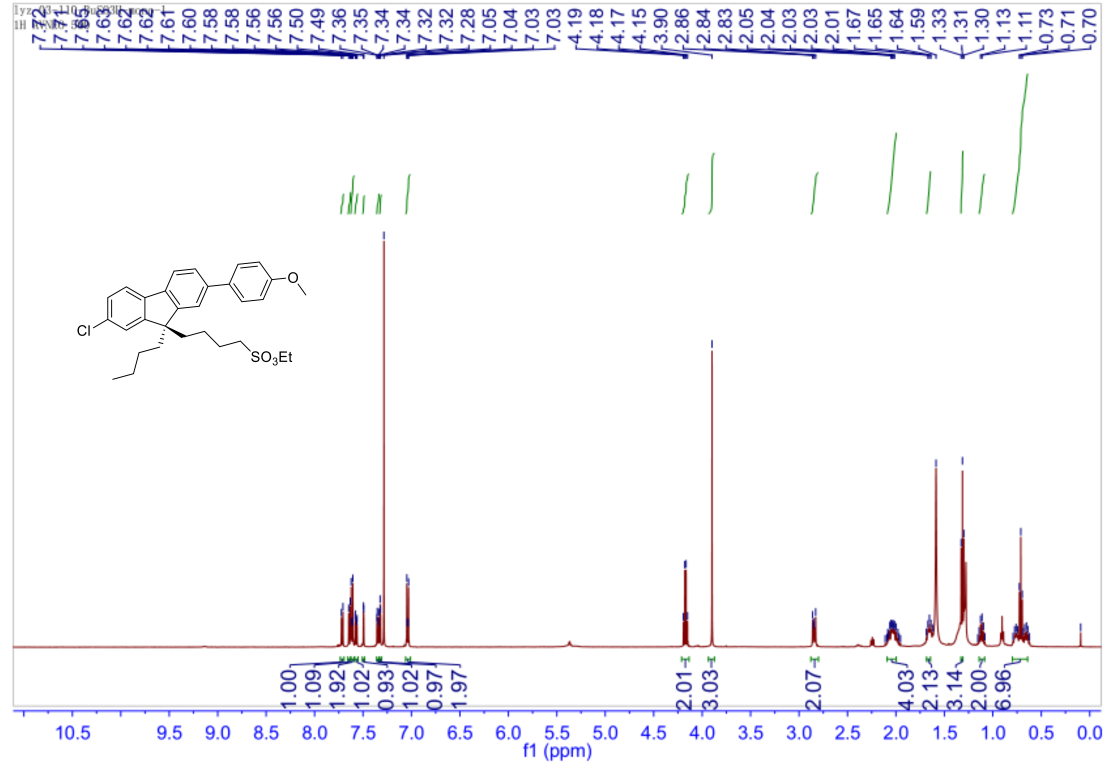

^{13}C NMR (126 MHz, $CDCl_3$) **-14**

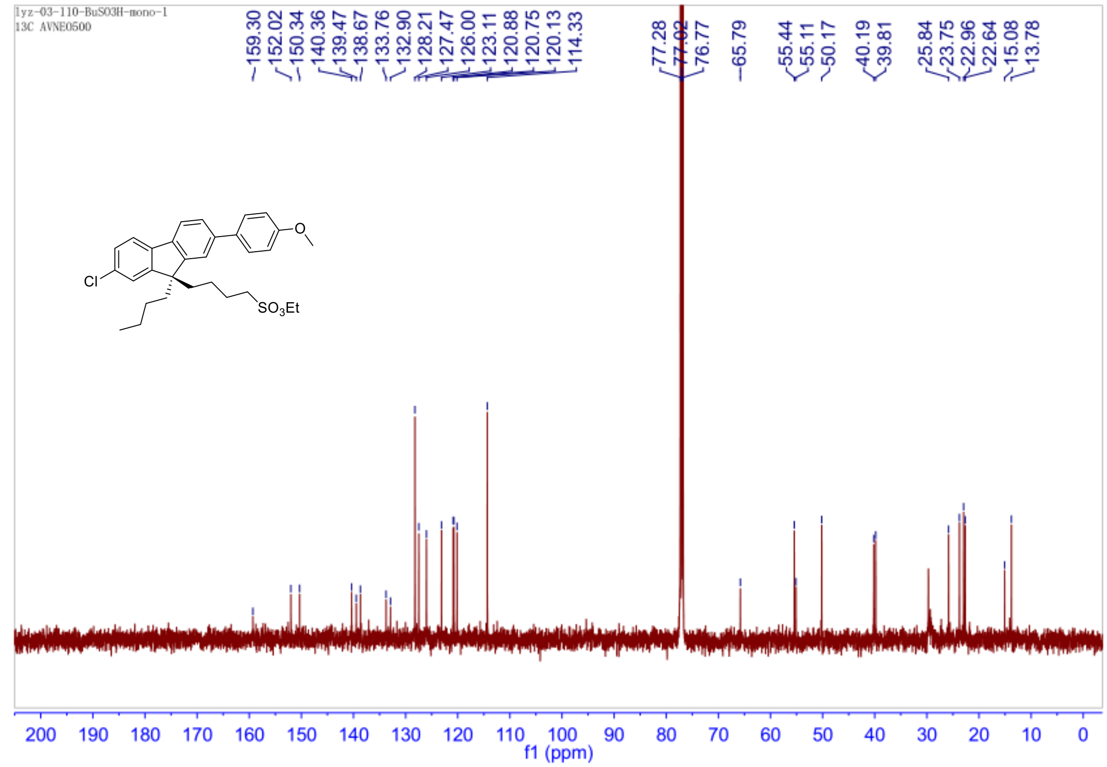

^{1}H NMR (500 MHz, $CDCl_3$) **-15**

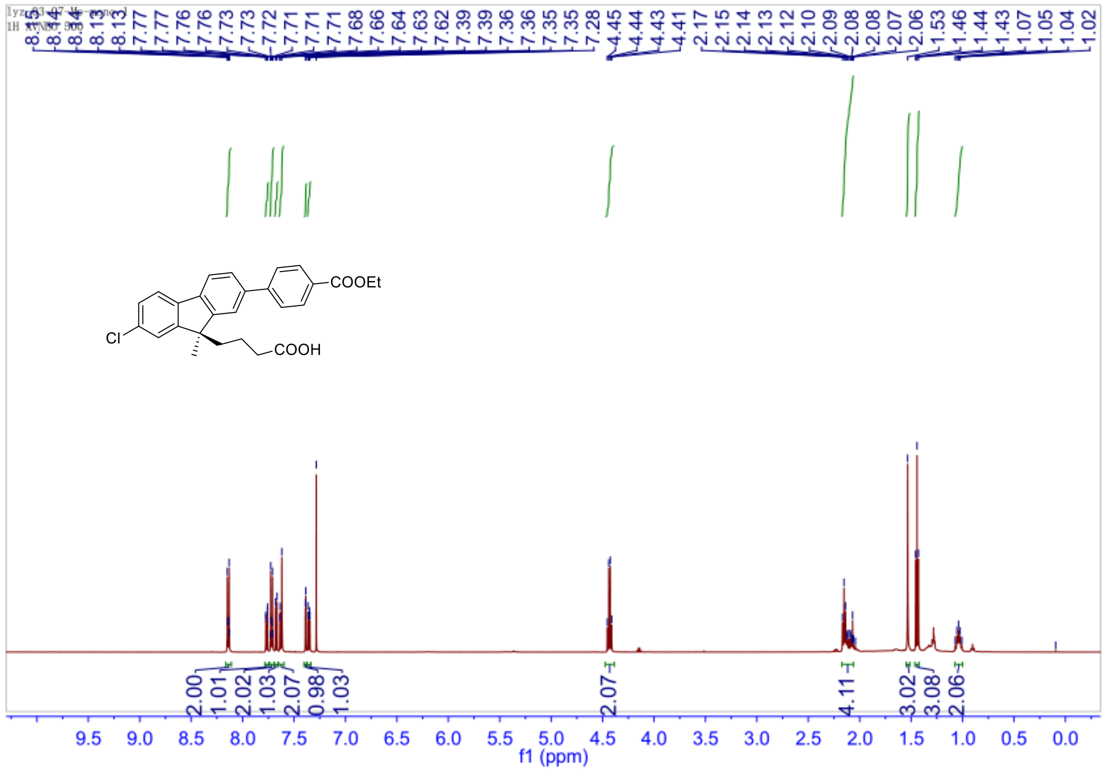

^{13}C NMR (126 MHz, $CDCl_3$) **-15**

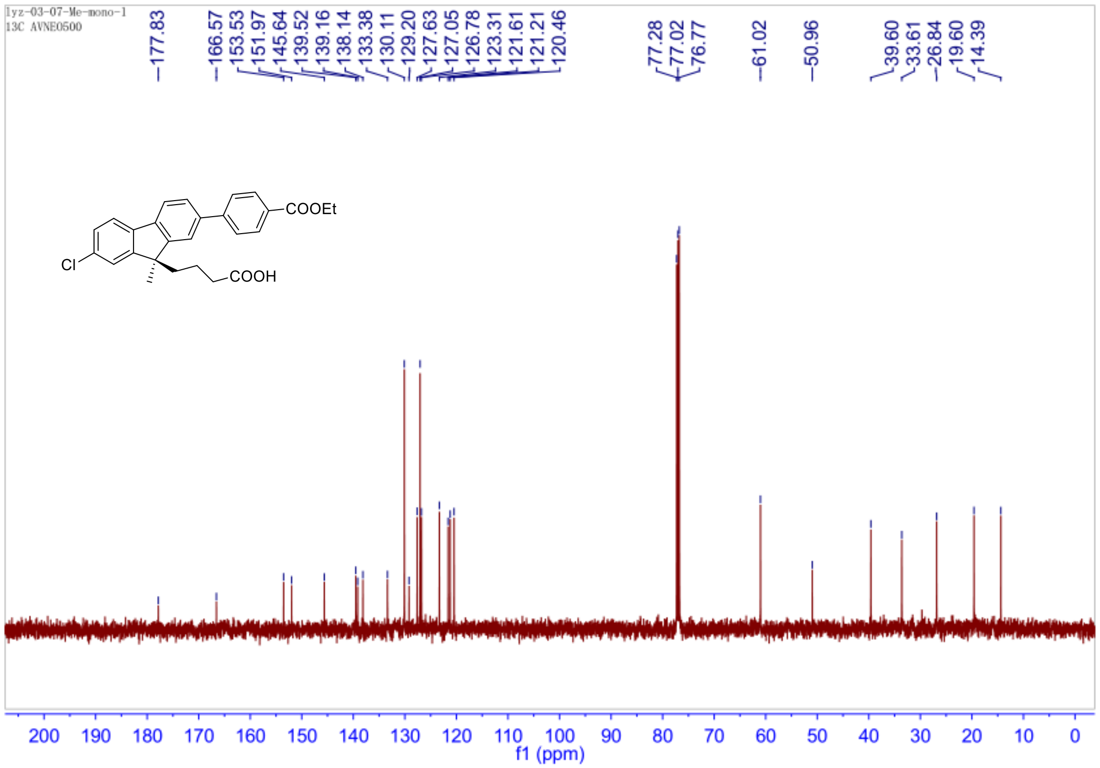

^{1}H NMR (500 MHz, $CDCl_3$) **-16**

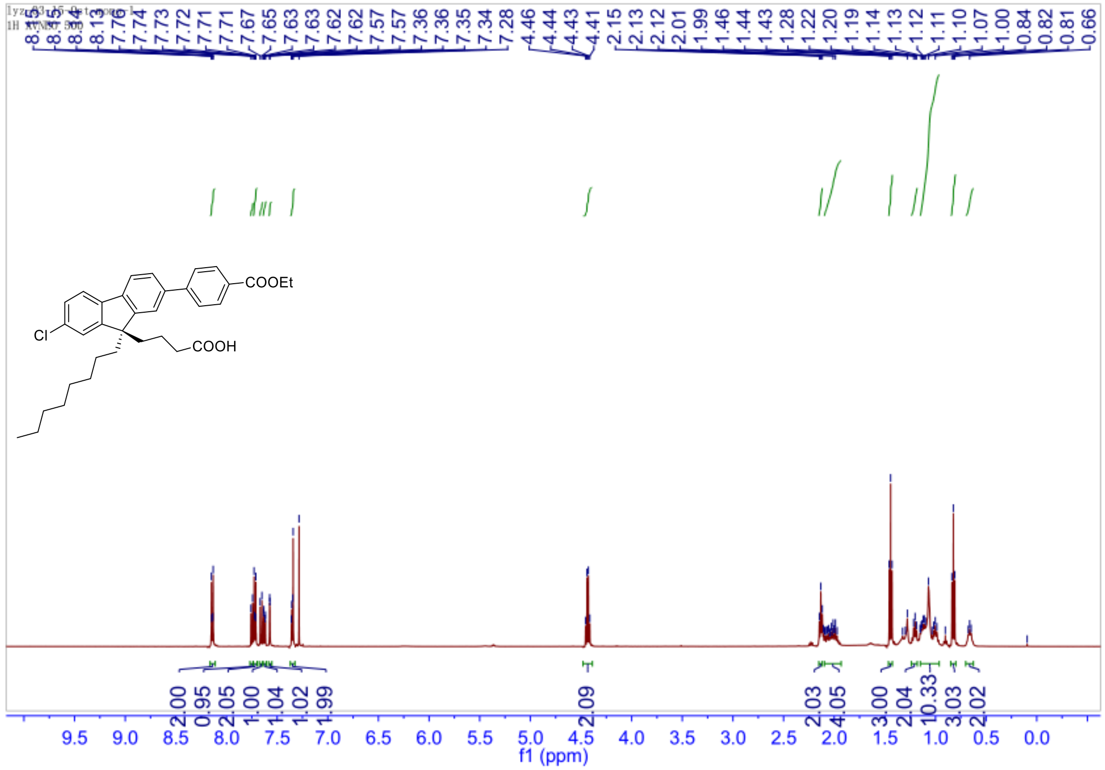

^{13}C NMR (126 MHz, $CDCl_3$) **-16**

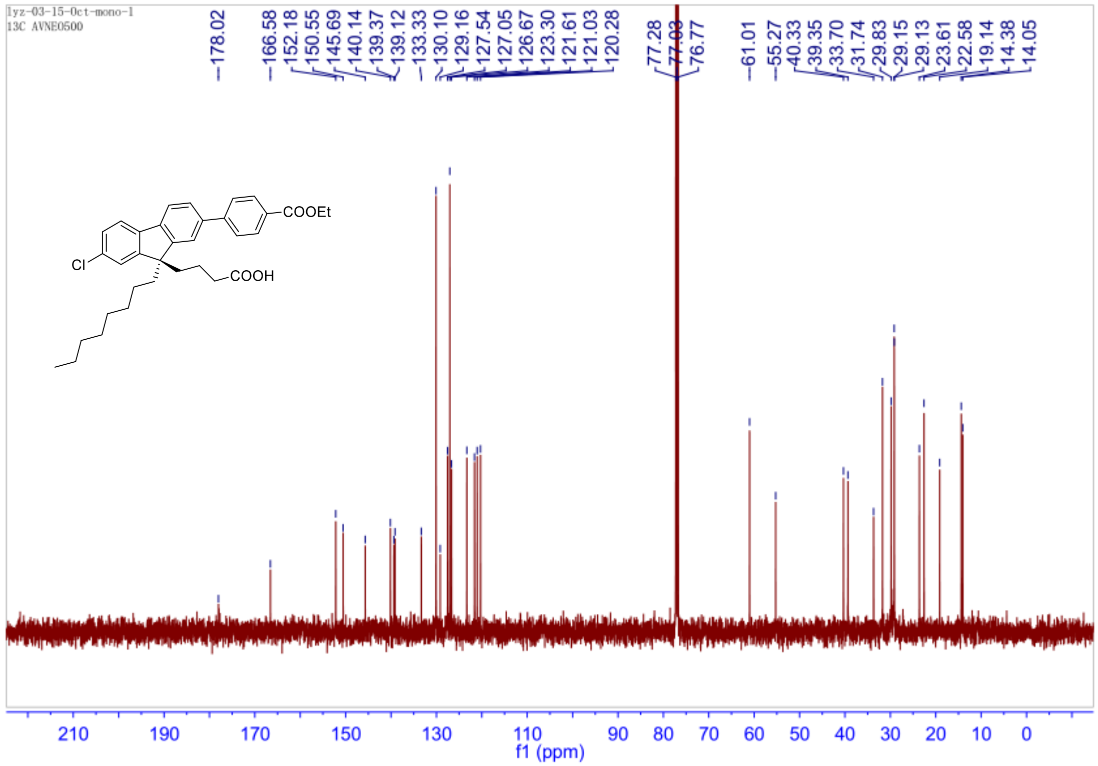

^{1}H NMR (500 MHz, $CDCl_3$) **-17**

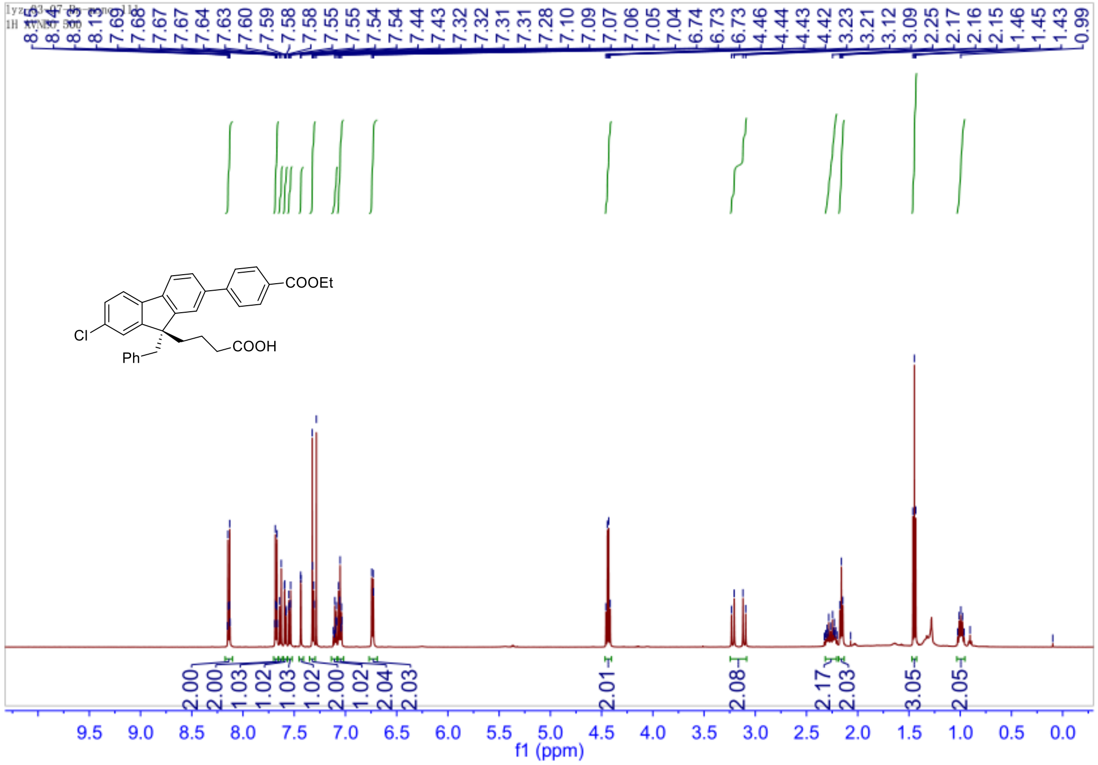

^{13}C NMR (126 MHz, $CDCl_3$) **-17**

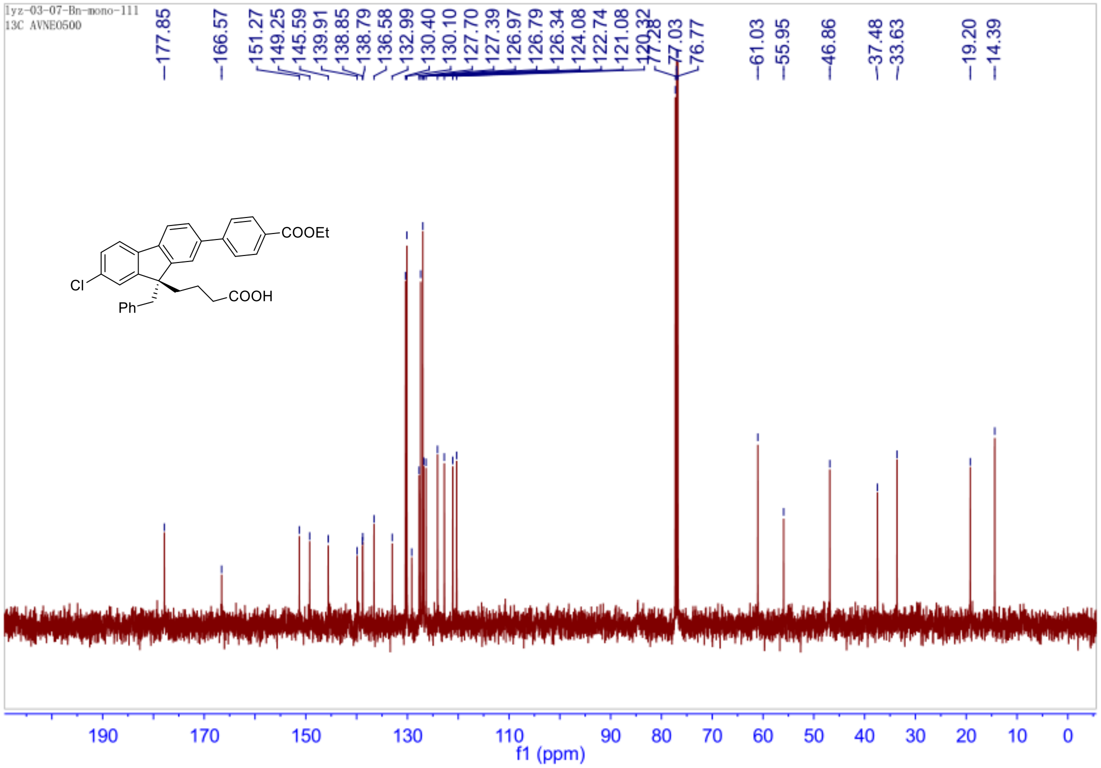

^{1}H NMR (500 MHz, $CDCl_3$) **-18**

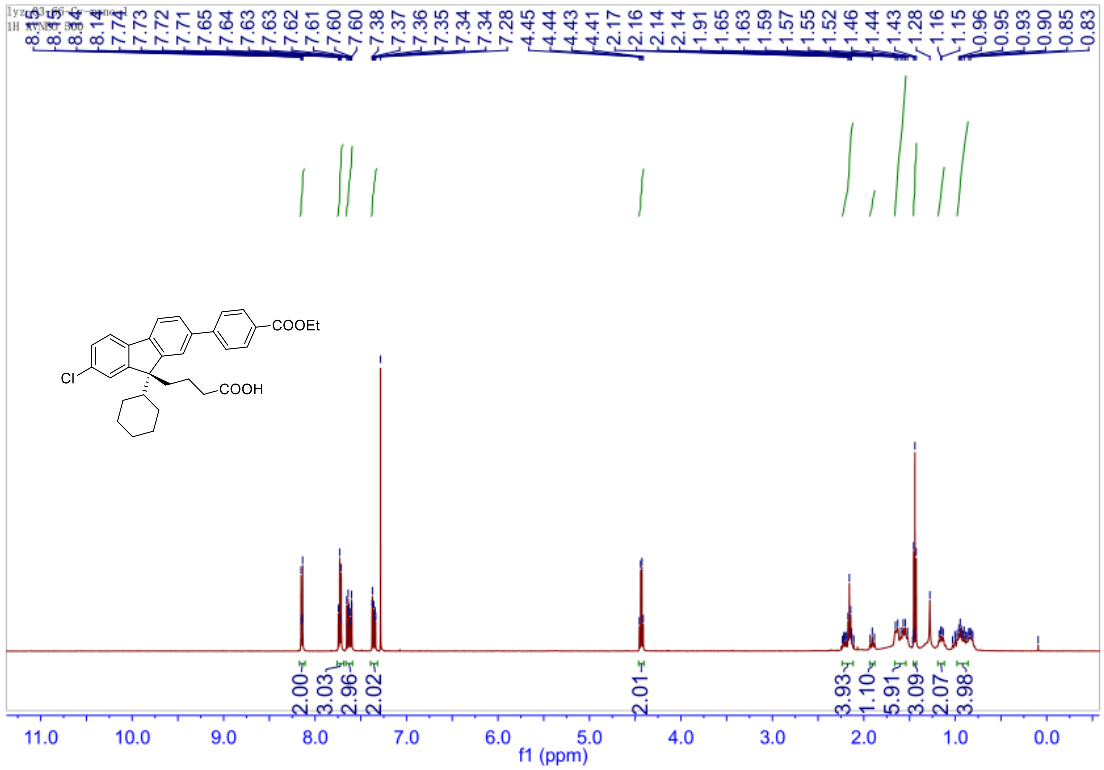

^{13}C NMR (126 MHz, $CDCl_3$) **-18**

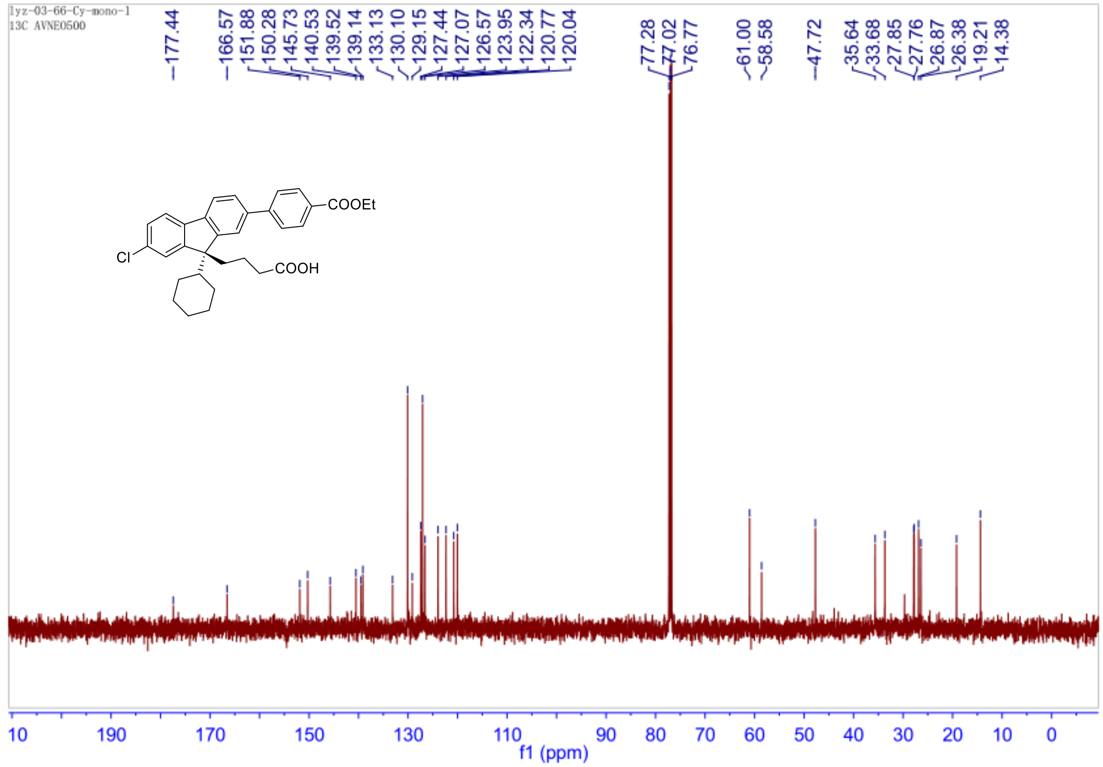

^{1}H NMR (500 MHz, $CDCl_3$) **-19**

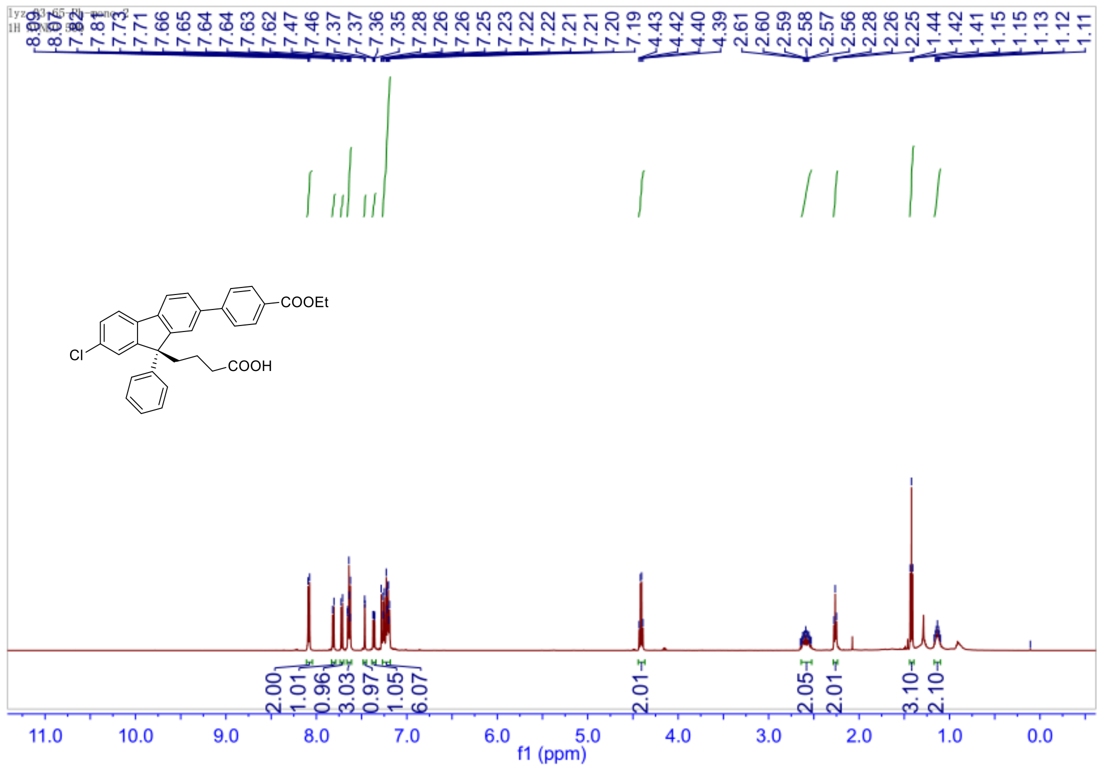

^{13}C NMR (126 MHz, $CDCl_3$) **-19**

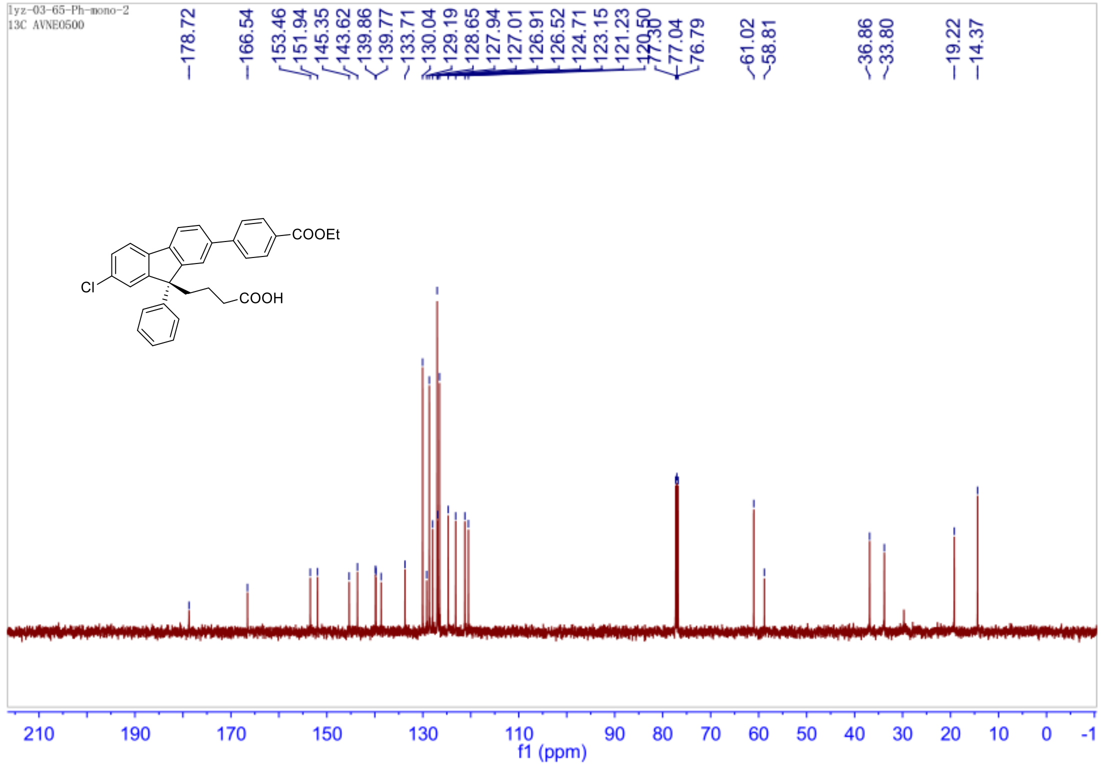

^{1}H NMR (500 MHz, $CDCl_3$)-**20**

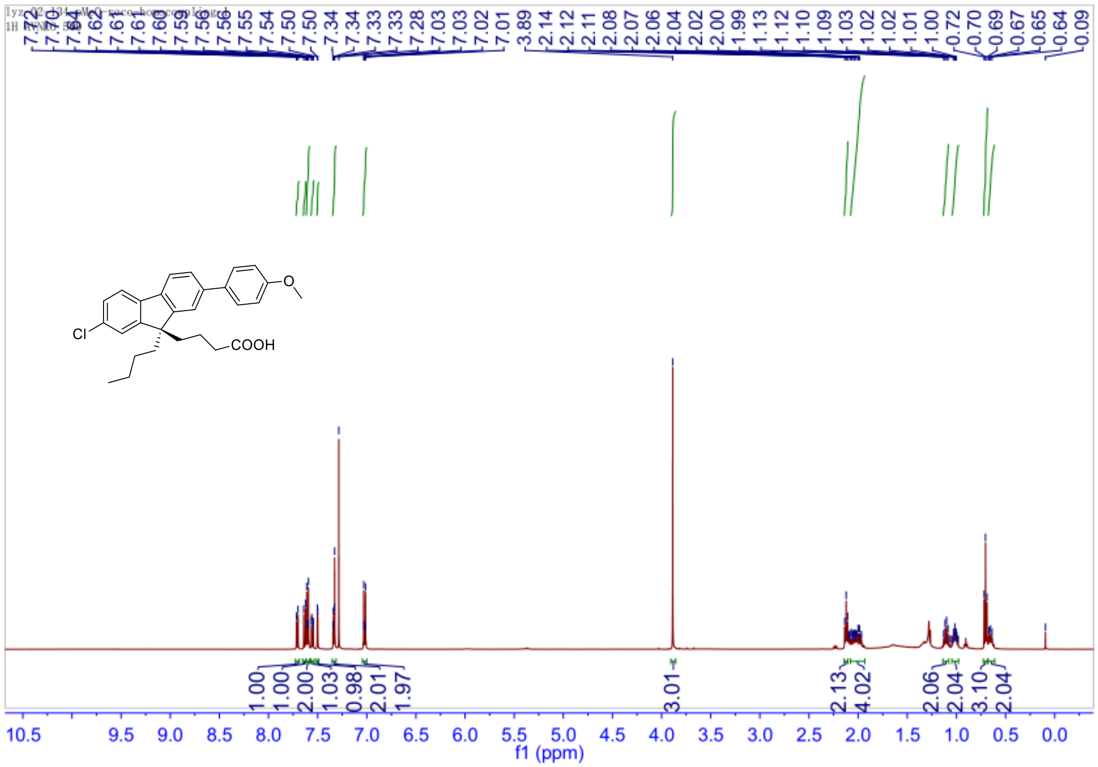

^{13}C NMR (126 MHz, $CDCl_3$)-**20**

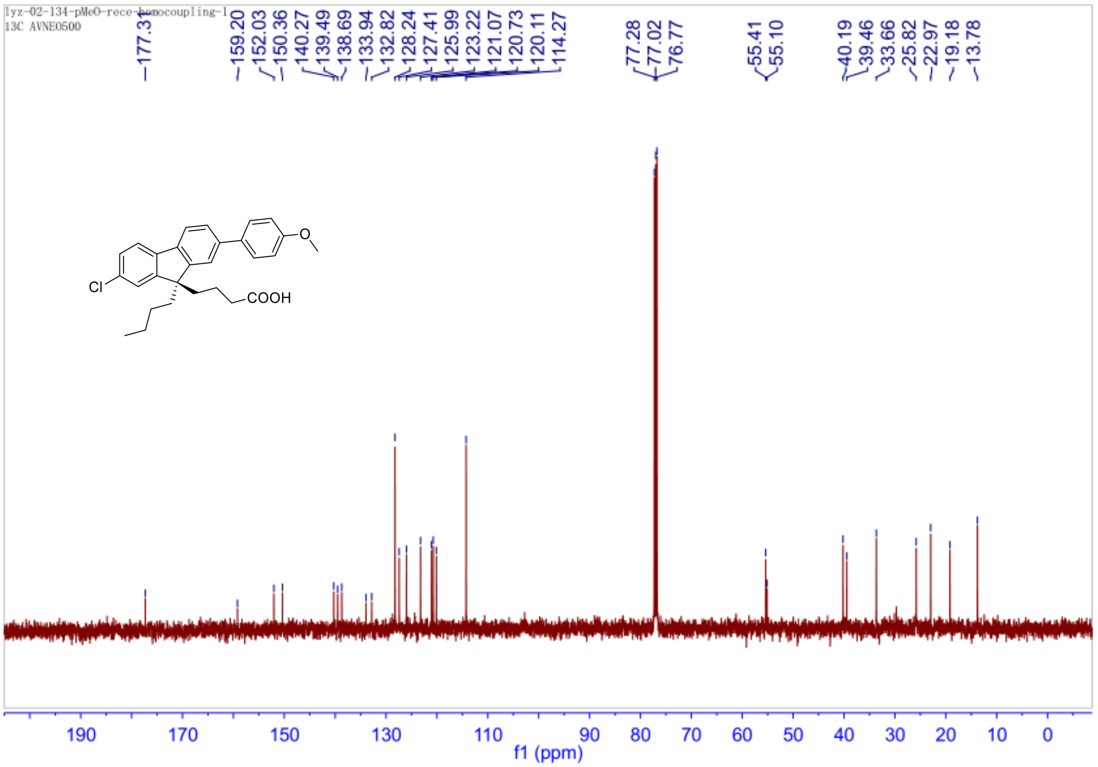

^{1}H NMR (500 MHz, $CDCl_3$)-**21**

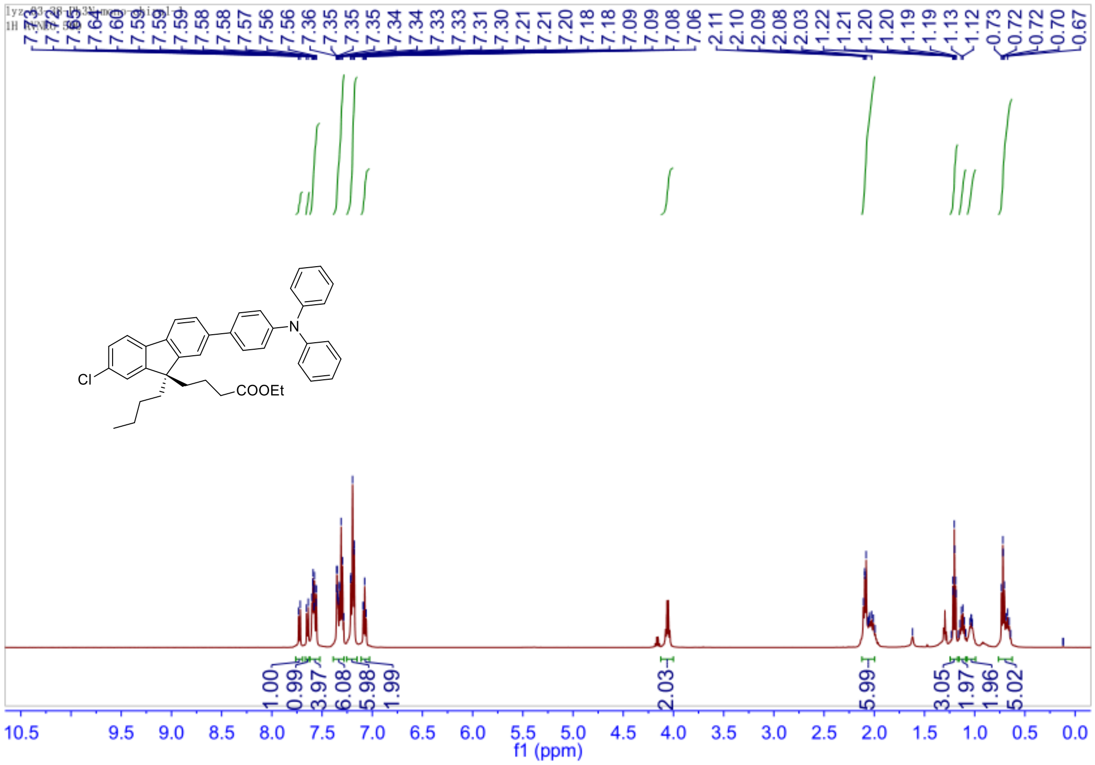

^{13}C NMR (126 MHz, $CDCl_3$)-**21**

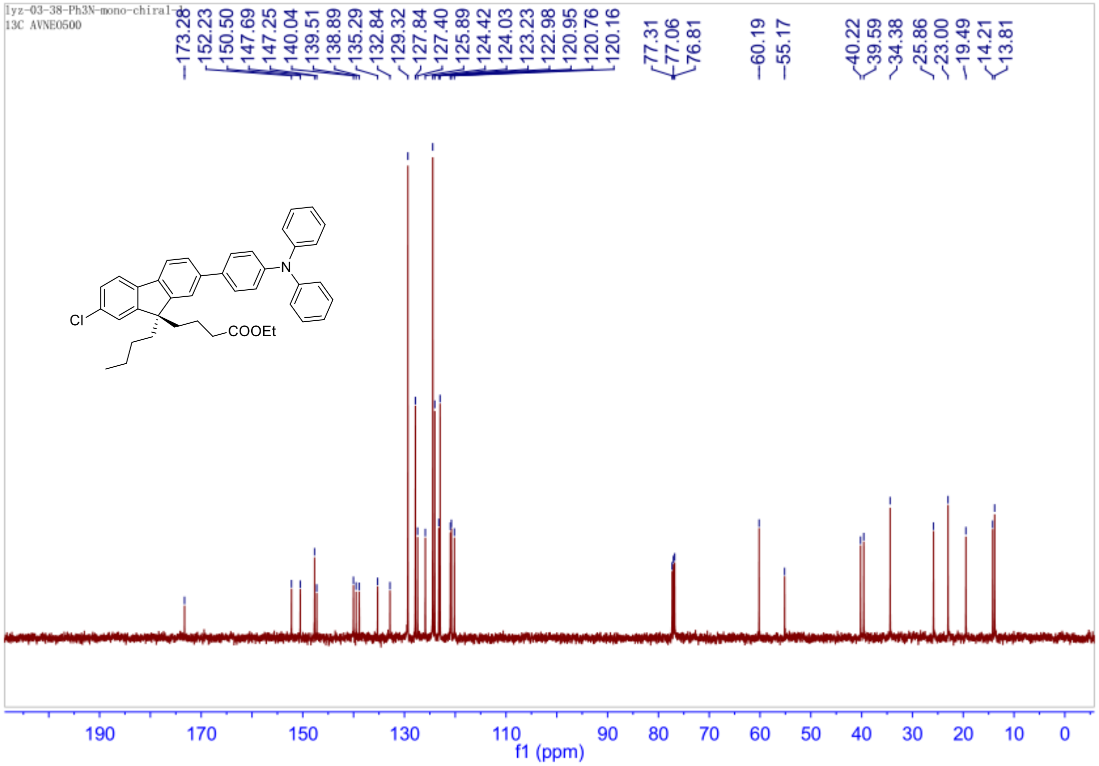

^{1}H NMR (500 MHz, $CDCl_3$)-**22**

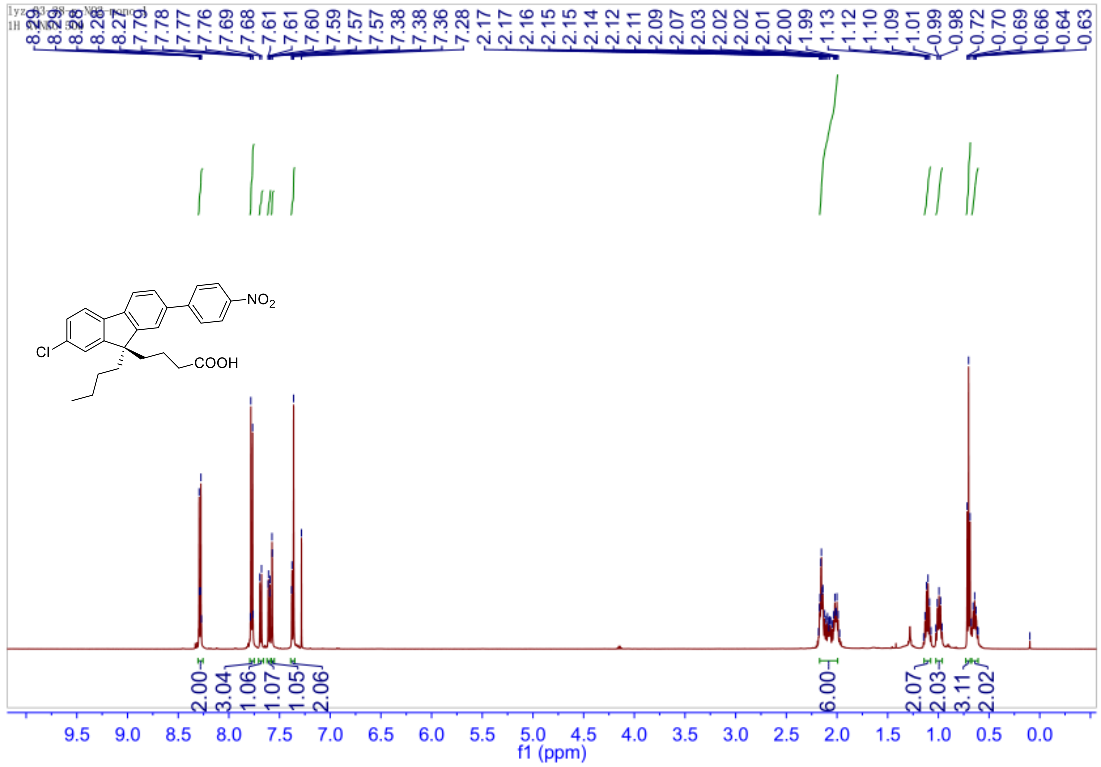

^{13}C NMR (126 MHz, $CDCl_3$)-**22**

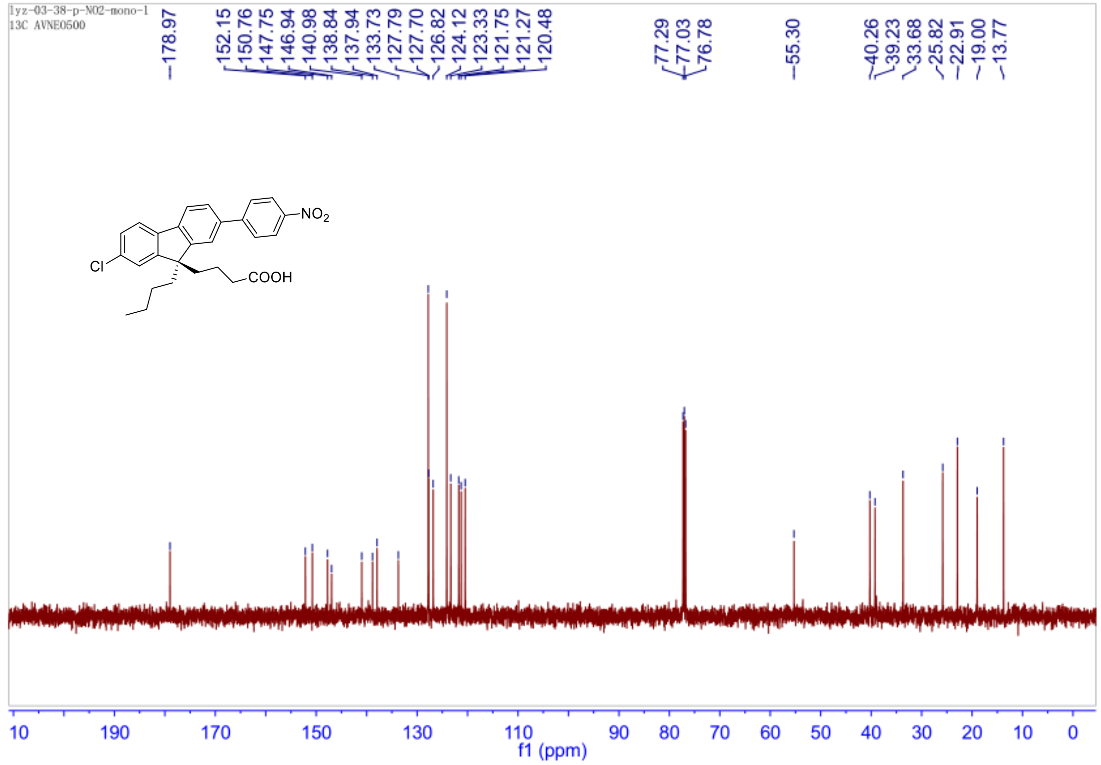

^{1}H NMR (500 MHz, $CDCl_3$)-**23**

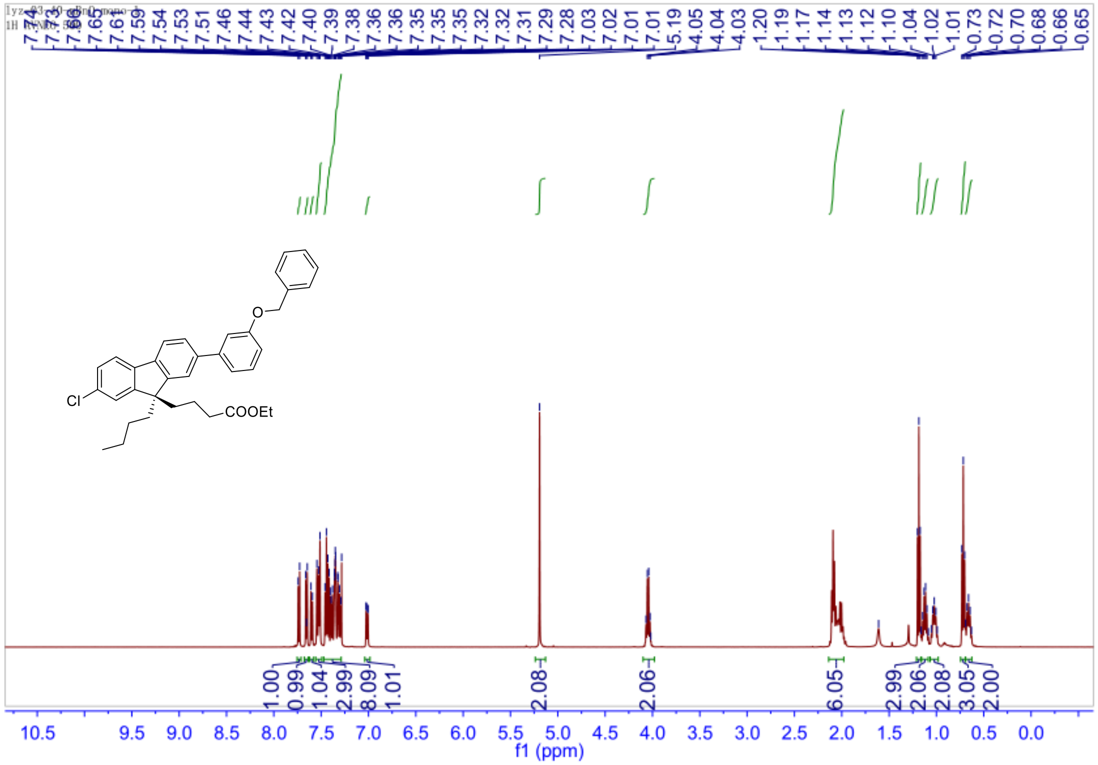

^{13}C NMR (126 MHz, $CDCl_3$)-**23**

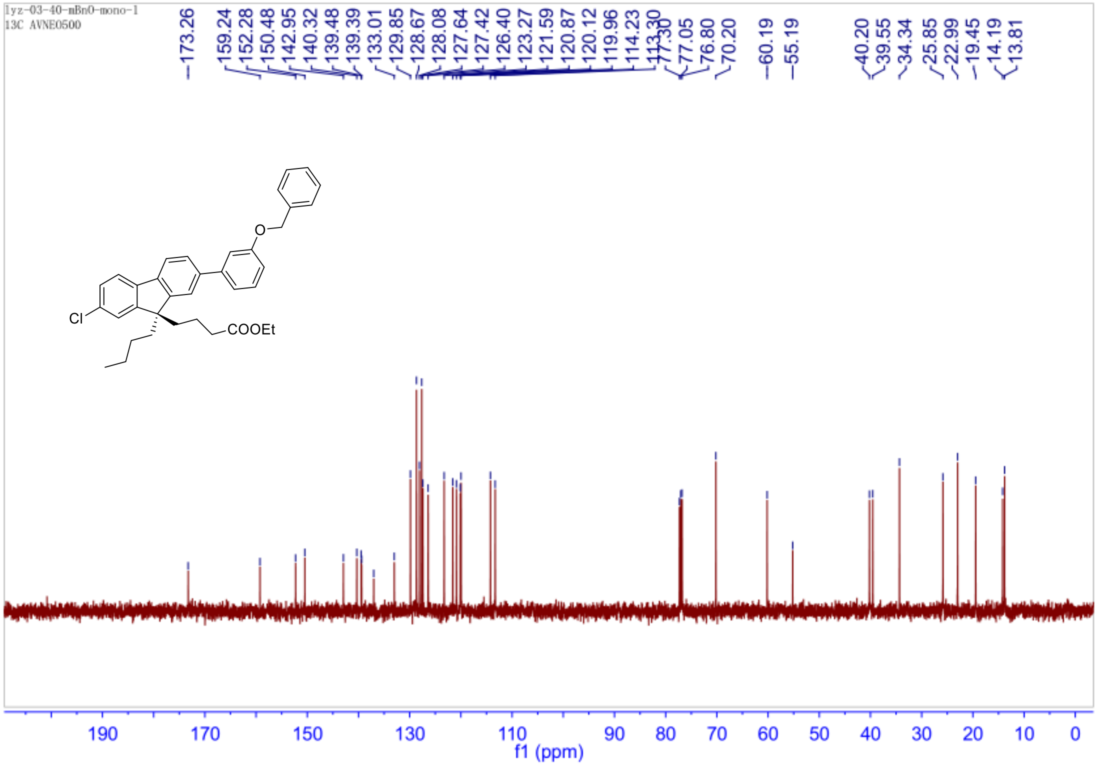

^{1}H NMR (500 MHz, CDCl$_3$)-**24**

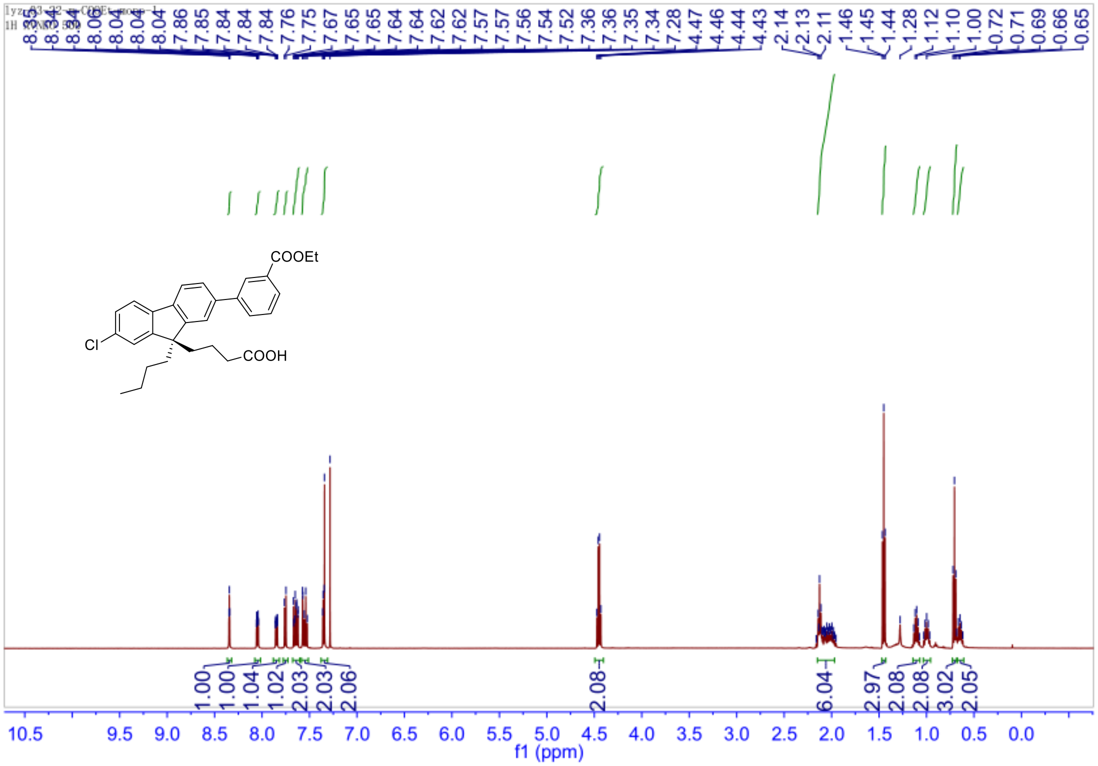

^{13}C NMR (126 MHz, CDCl$_3$)-**24**

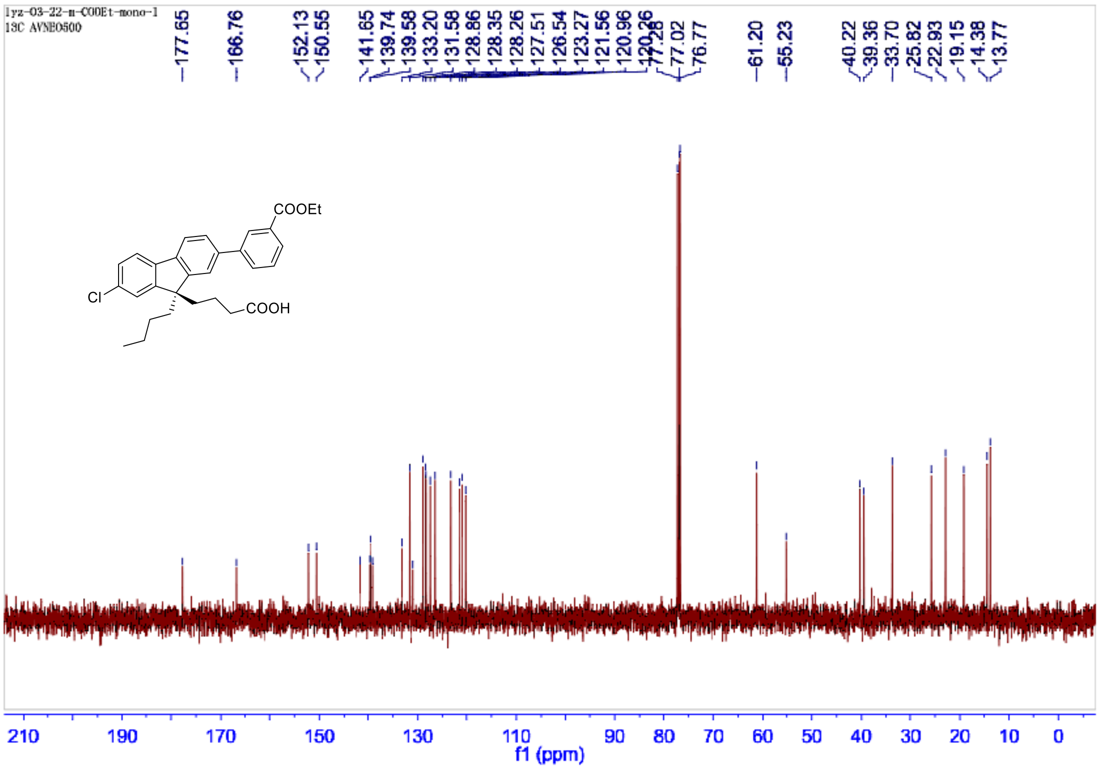

^{1}H NMR (500 MHz, $CDCl_3$)-**25**

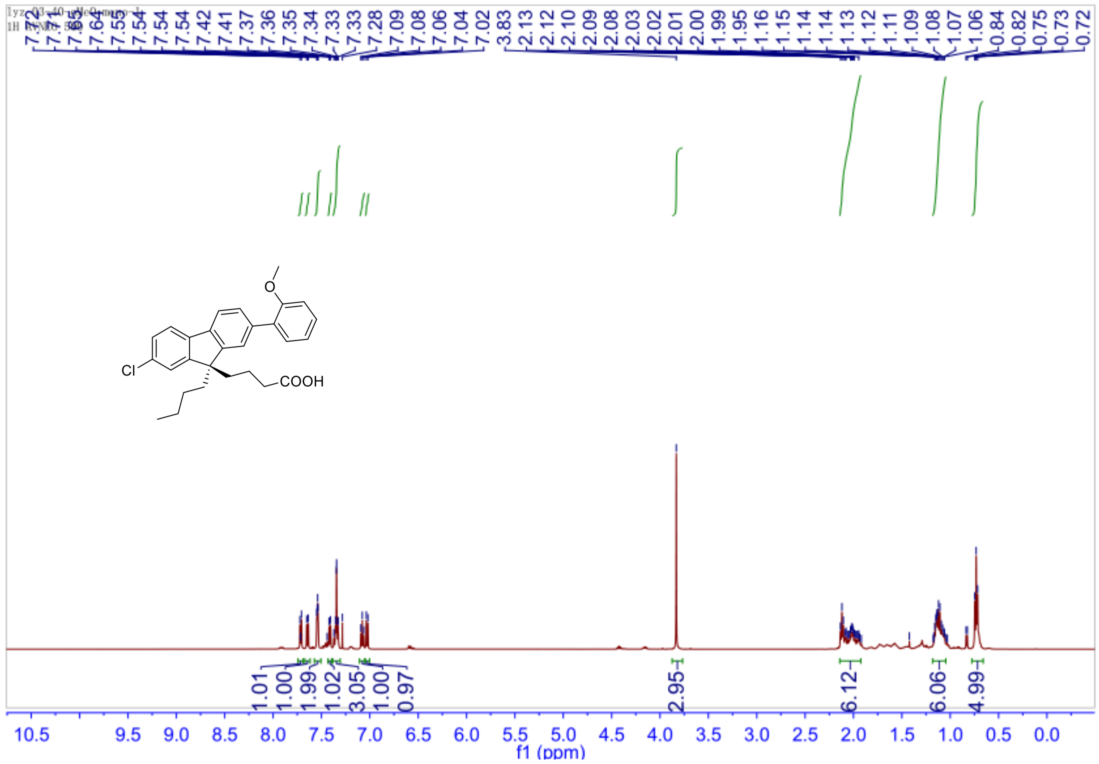

^{13}C NMR (126 MHz, $CDCl_3$)-**25**

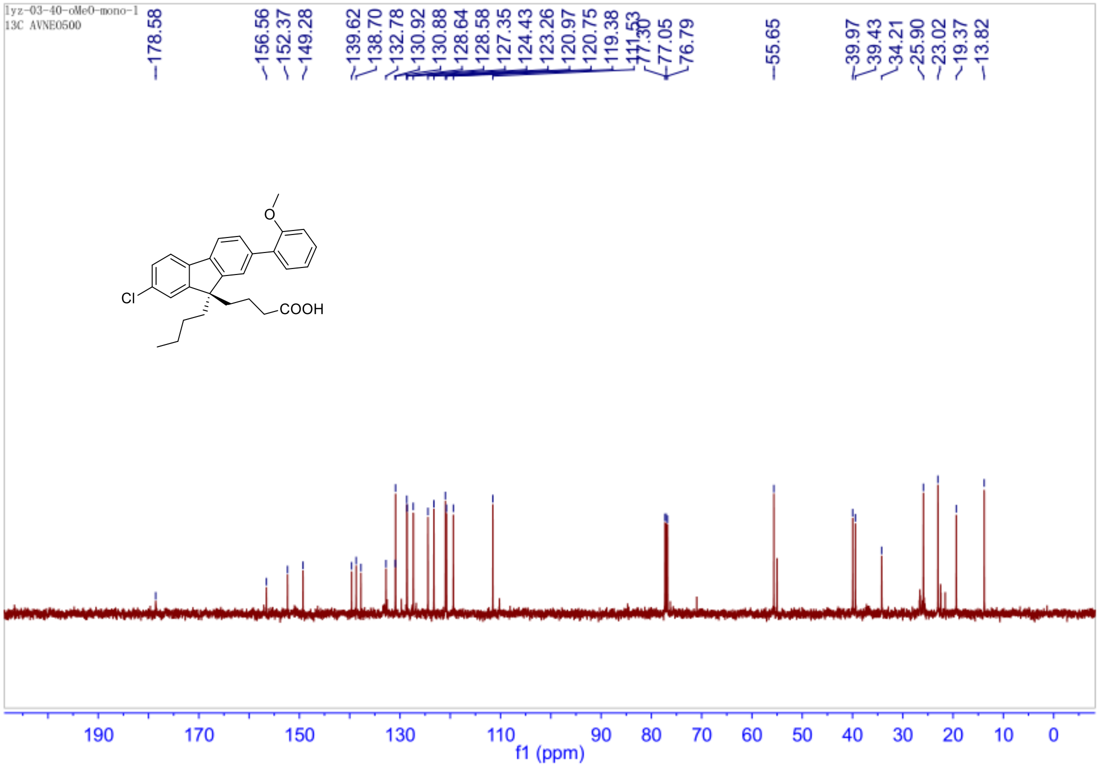

^{1}H NMR (500 MHz, $CDCl_3$)-**26**

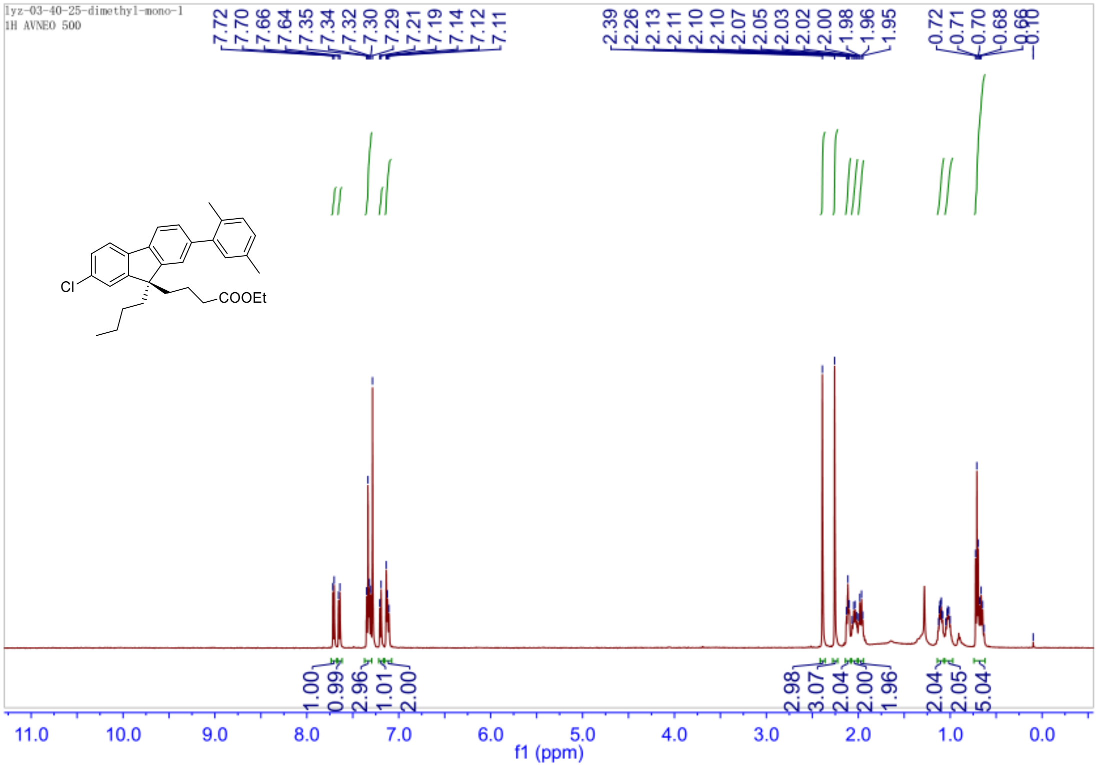

^{13}C NMR (126 MHz, $CDCl_3$)-**26**

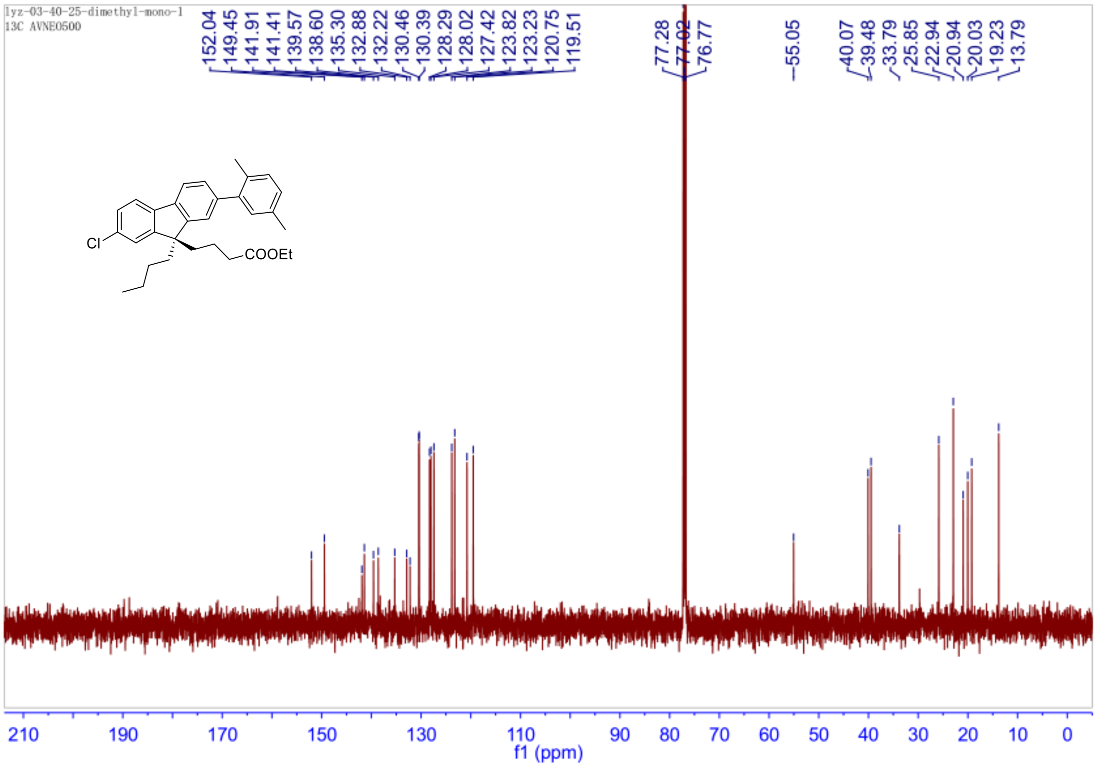

^{1}H NMR (500 MHz, $CDCl_3$)-**27**

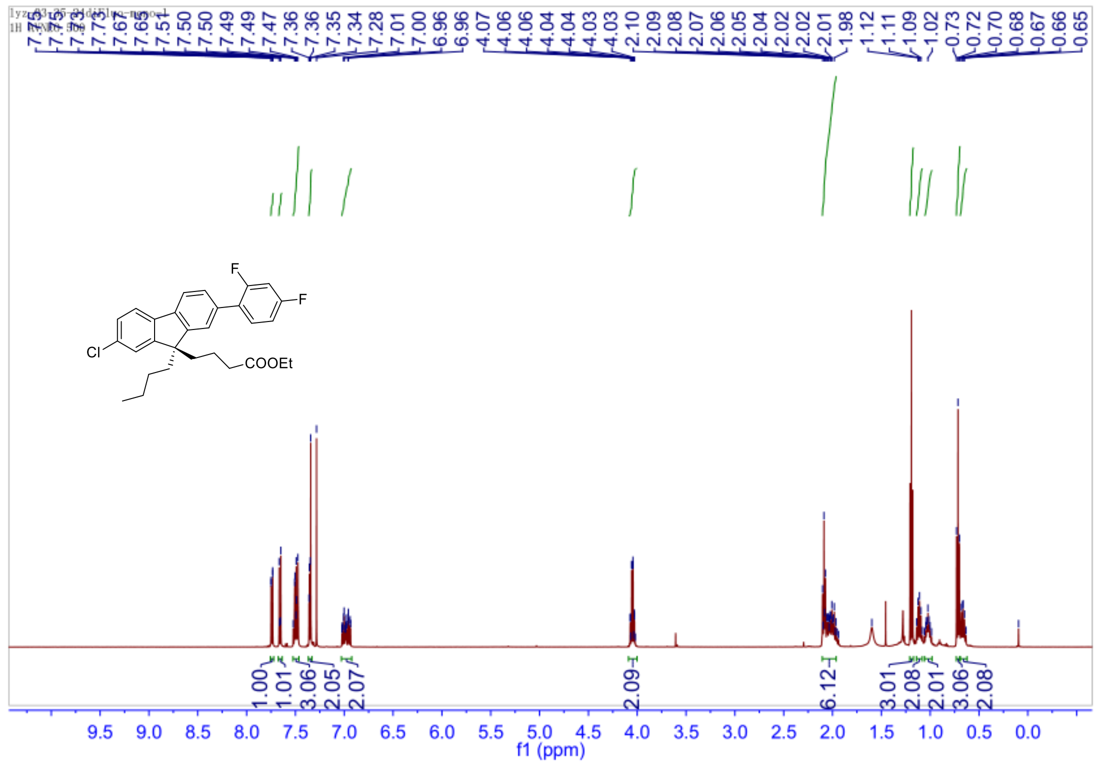

^{13}C NMR (126 MHz, $CDCl_3$)-**27**

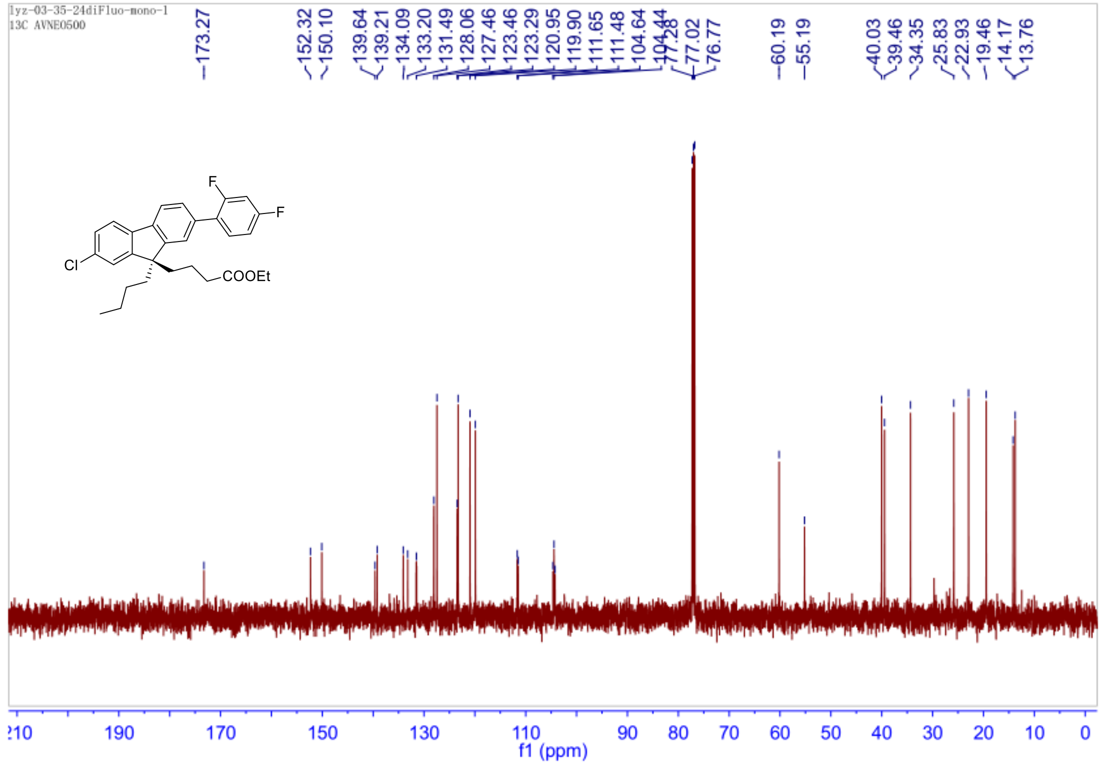

^{19}F NMR (471 MHz, $CDCl_3$)-**27**

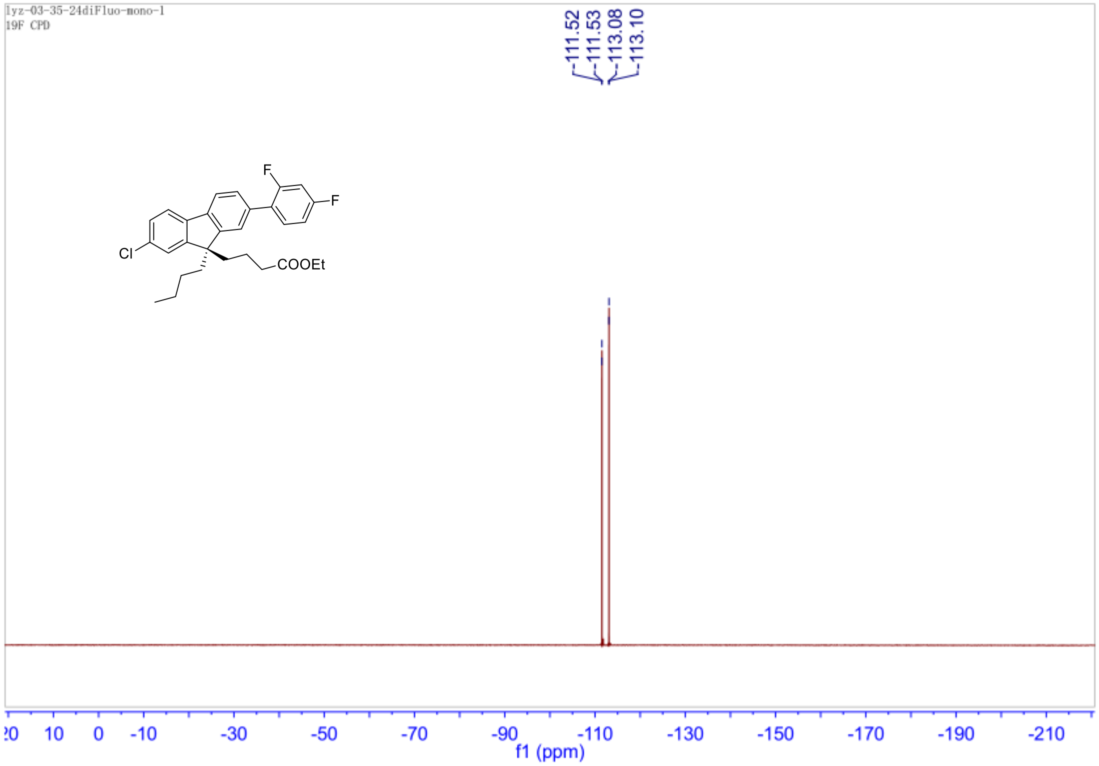

^{1}H NMR (500 MHz, $CDCl_3$)-**28**

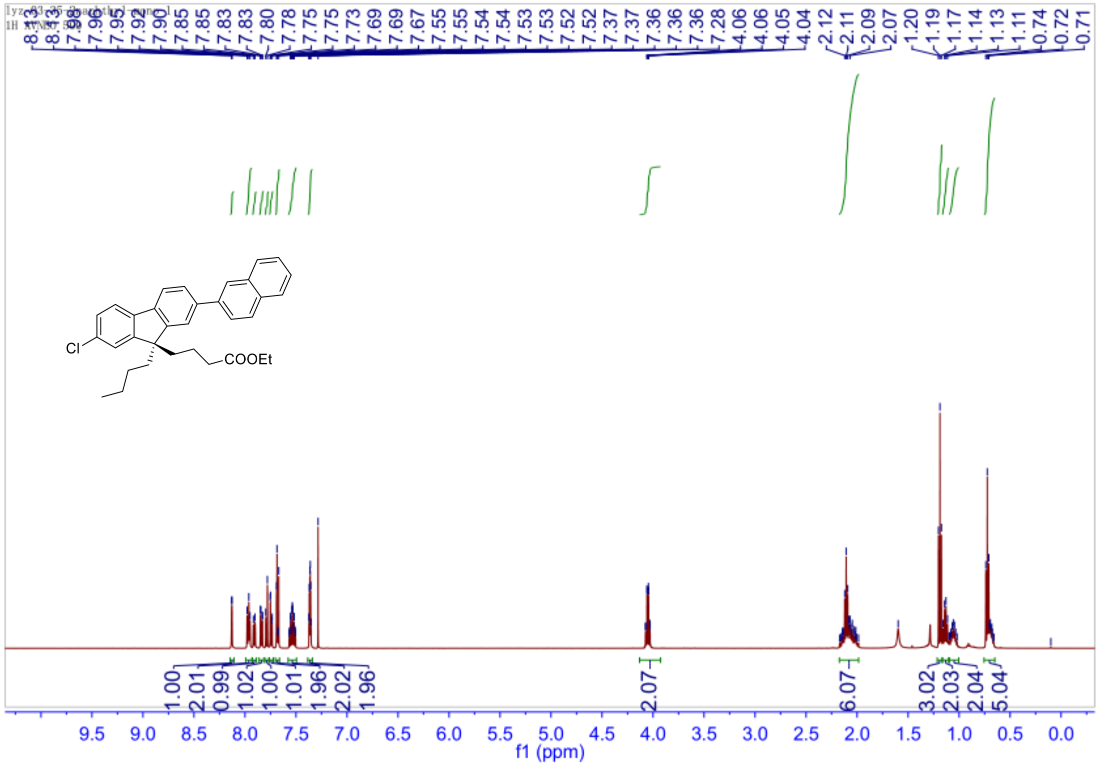

^{13}C NMR (126 MHz, $CDCl_3$)-**28**

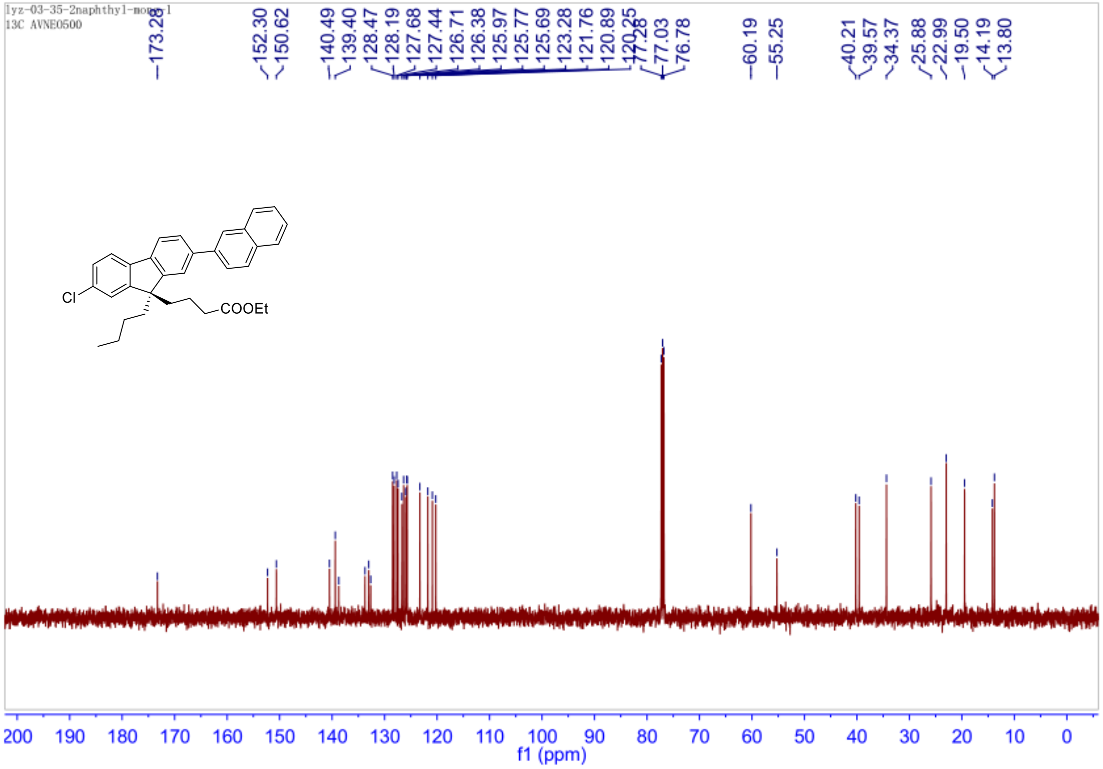

^{1}H NMR (500 MHz, $CDCl_3$)-**29**

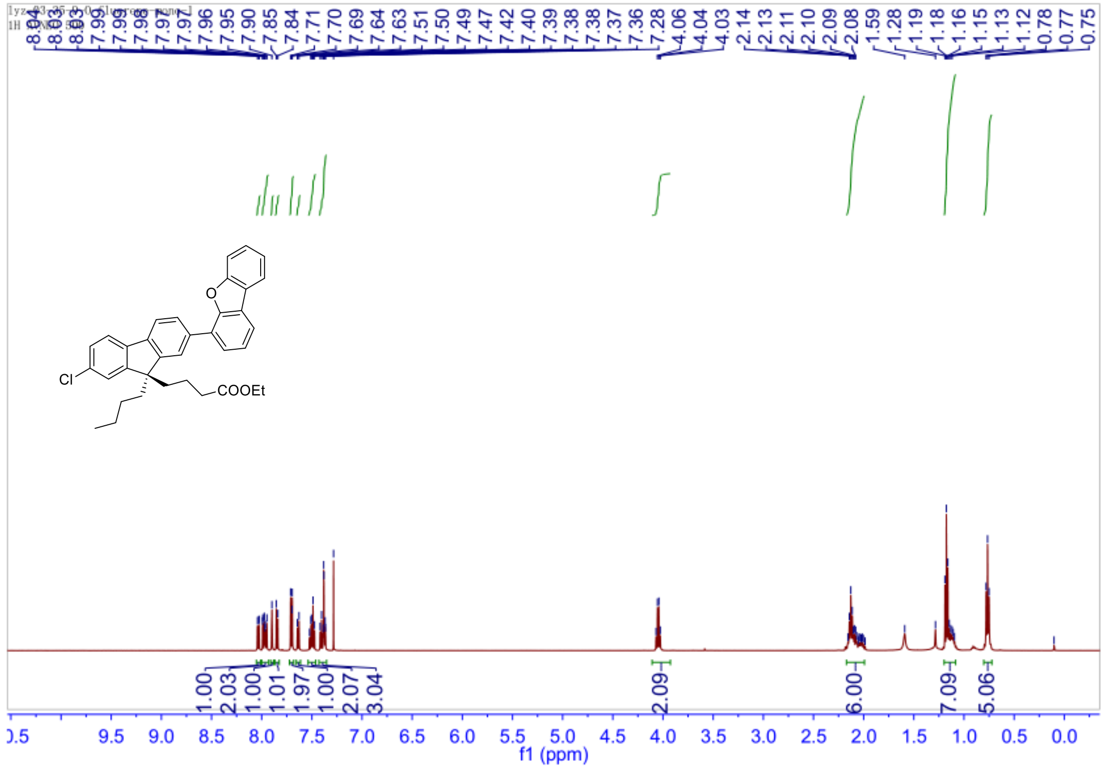

^{13}C NMR (126 MHz, $CDCl_3$)-**29**

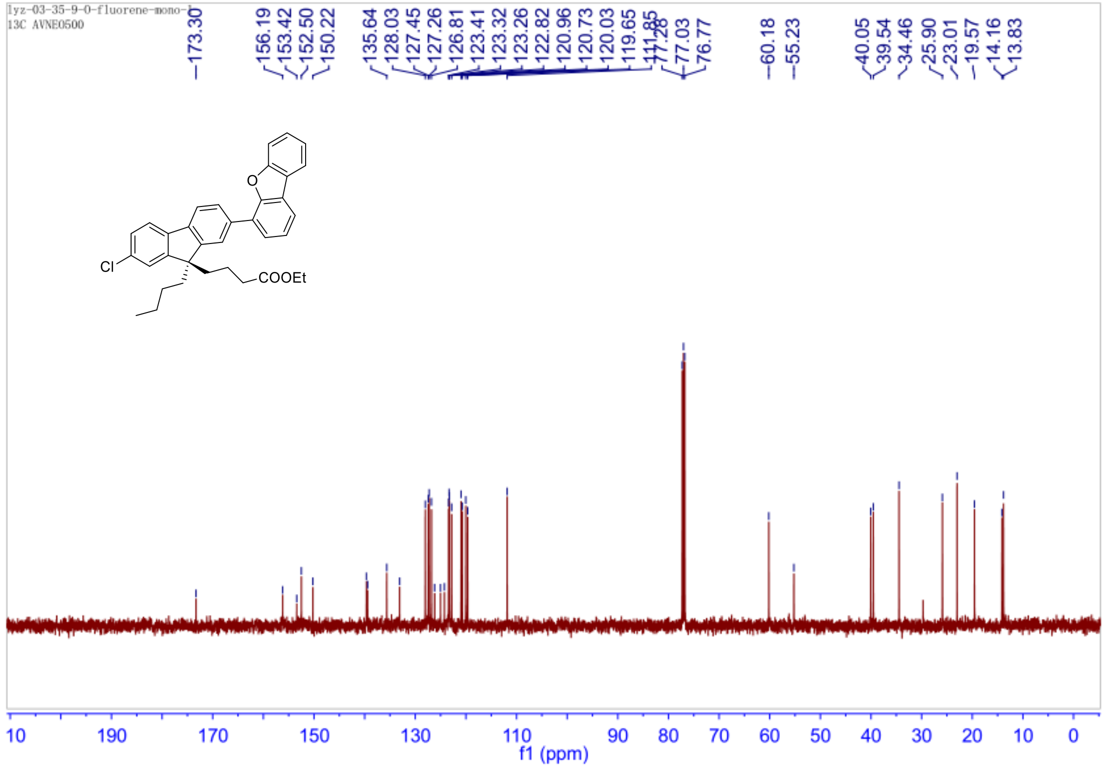

^{1}H NMR (500 MHz, $CDCl_3$)-**30**

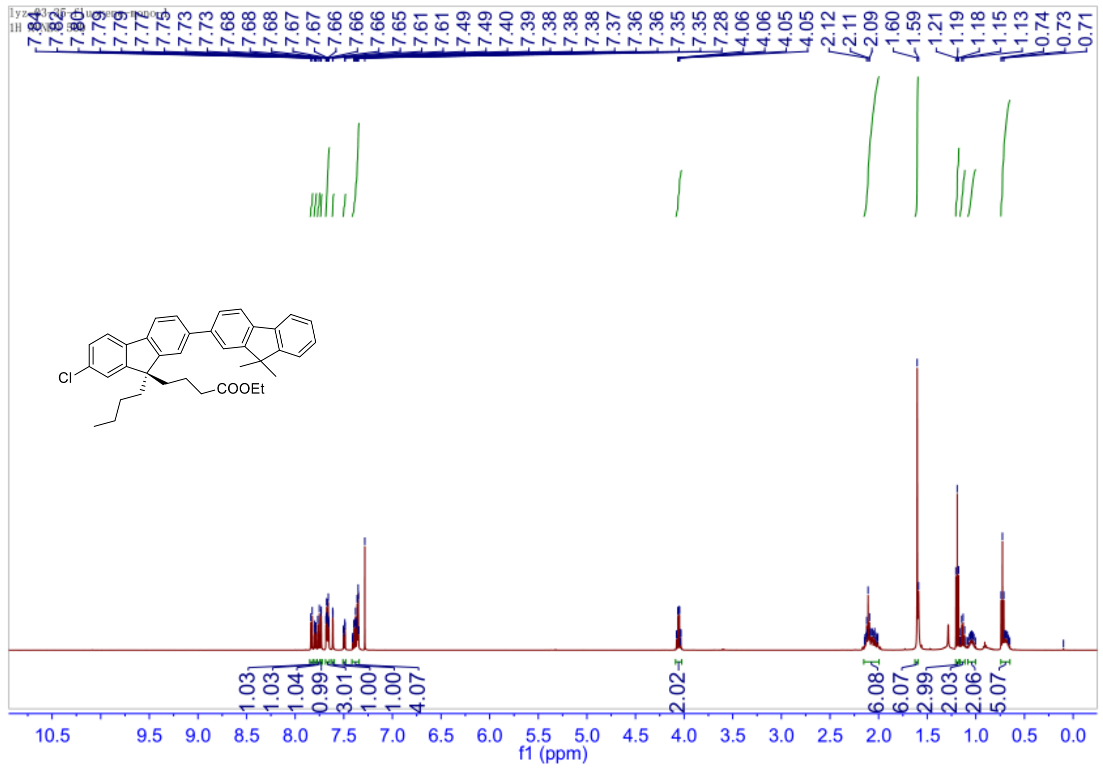

^{13}C NMR (126 MHz, $CDCl_3$)-**30**

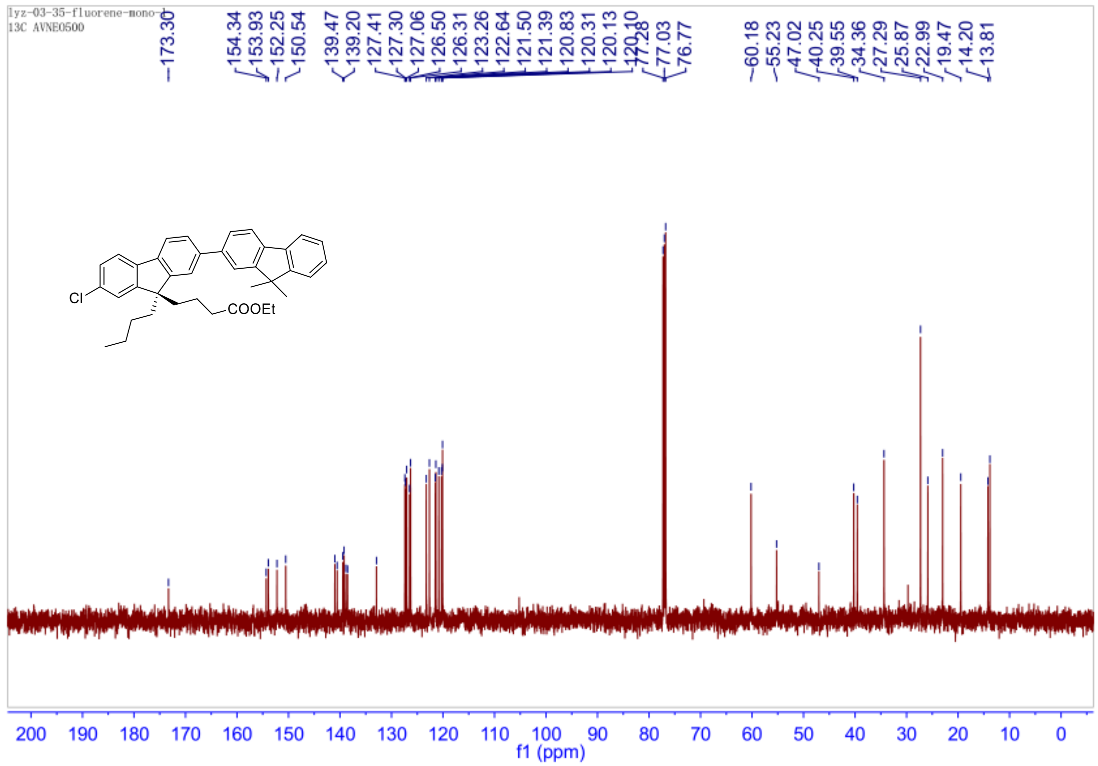

^{1}H NMR (500 MHz, $CDCl_3$)-**31**

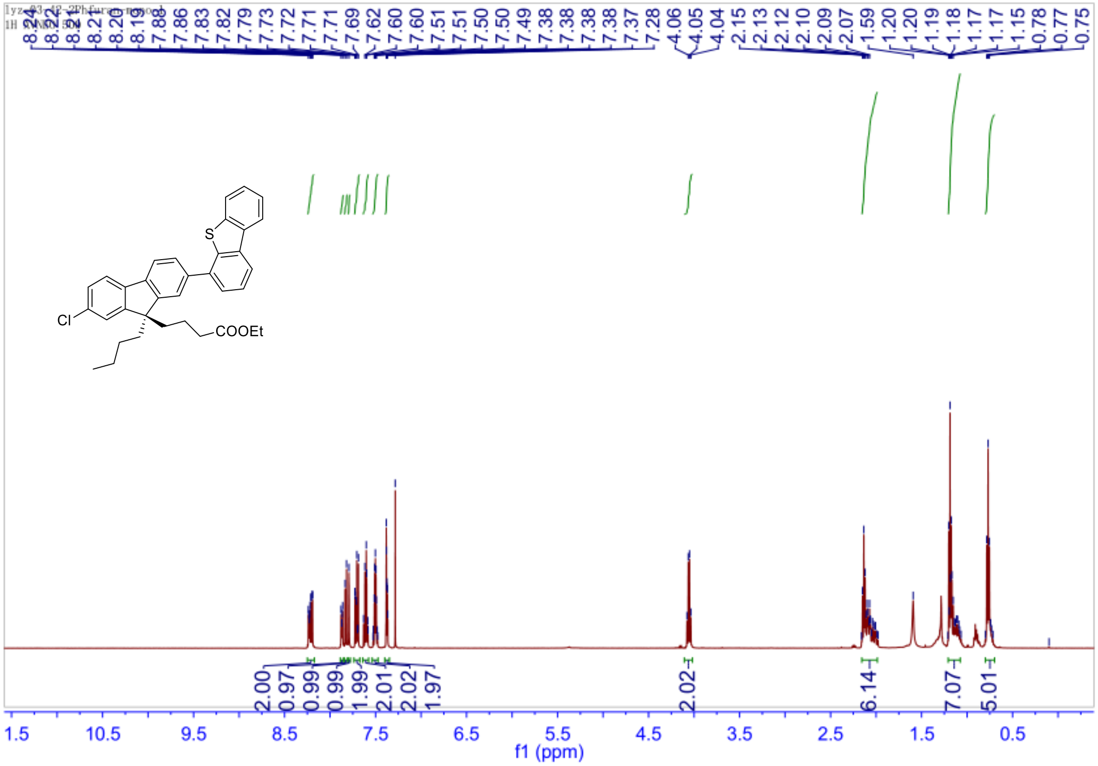

^{13}C NMR (126 MHz, $CDCl_3$)-**31**

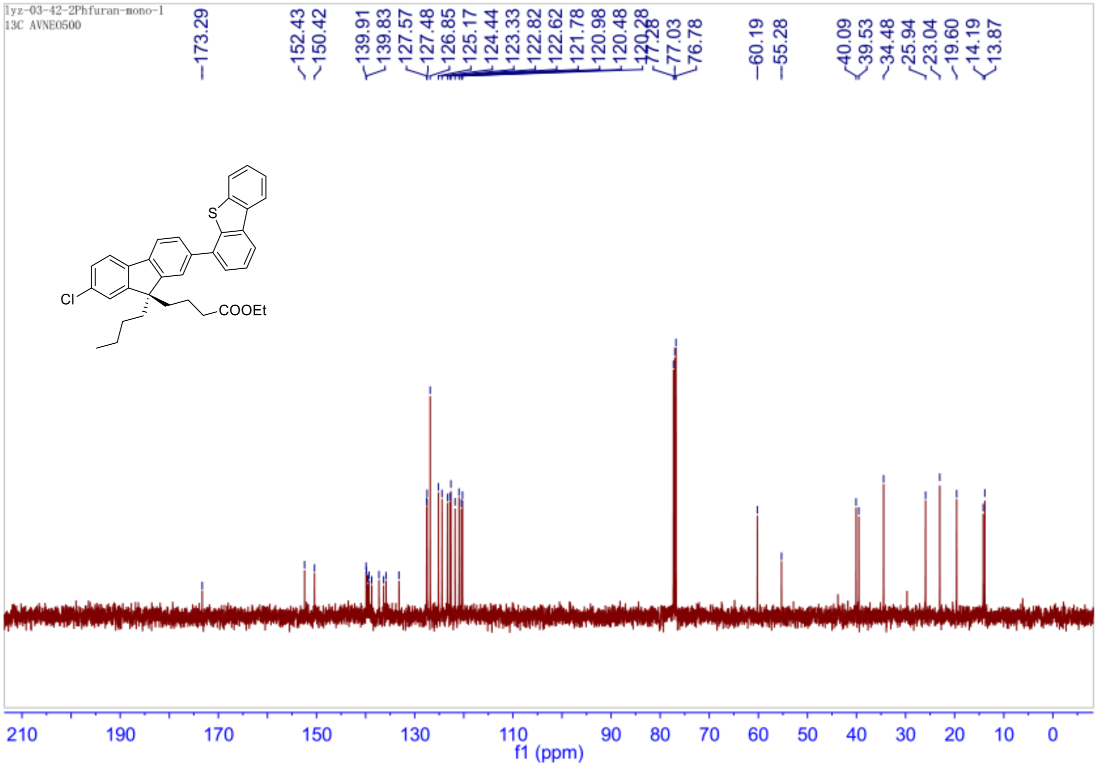

^{1}H NMR (500 MHz, $CDCl_3$)-**32**

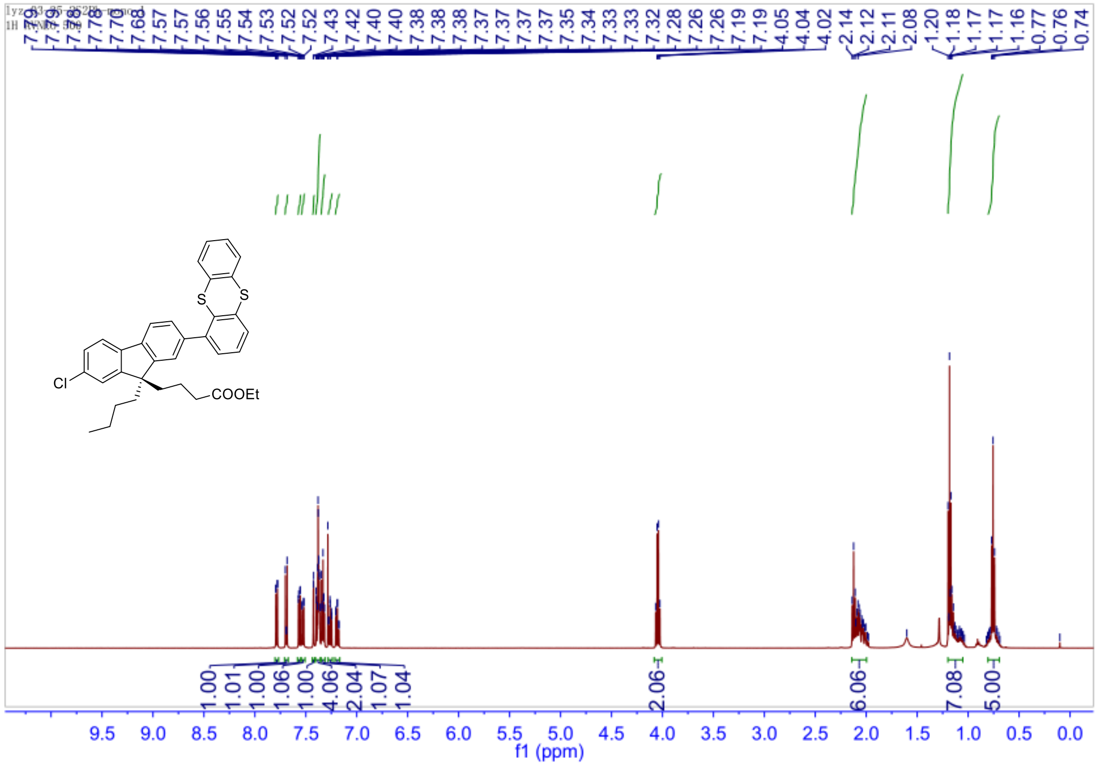

^{13}C NMR (126 MHz, $CDCl_3$)-**32**

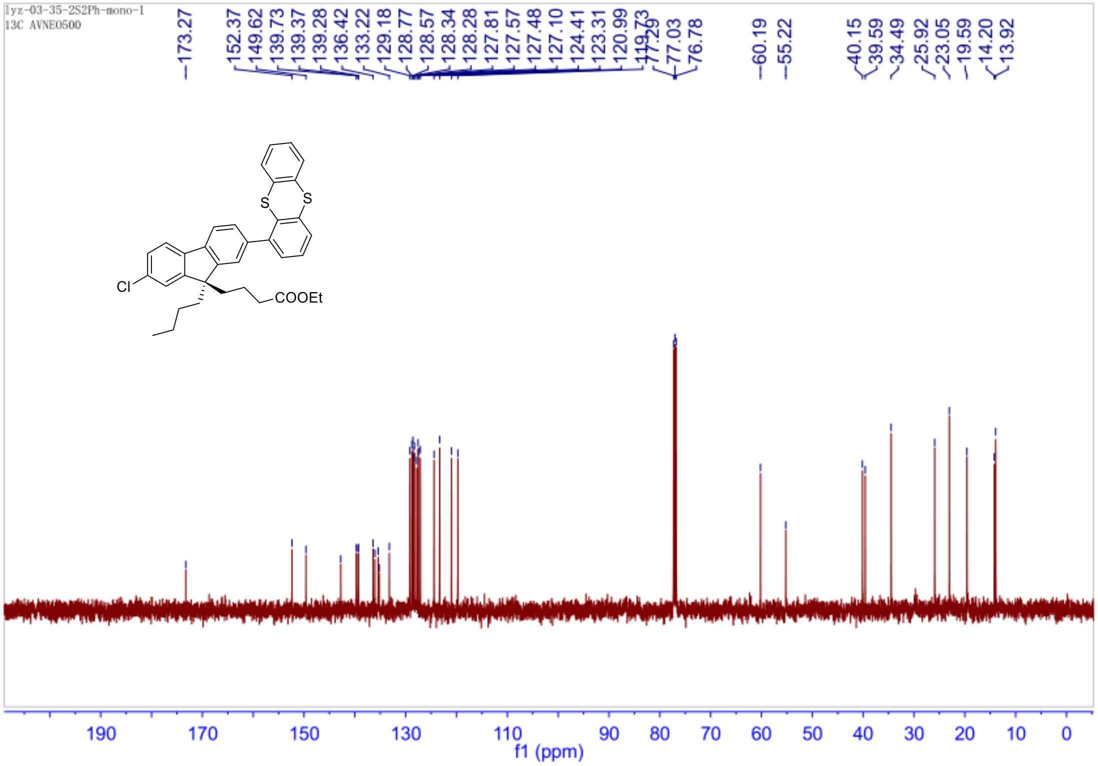

^1H NMR (500 MHz, $CDCl_3$)-**33**

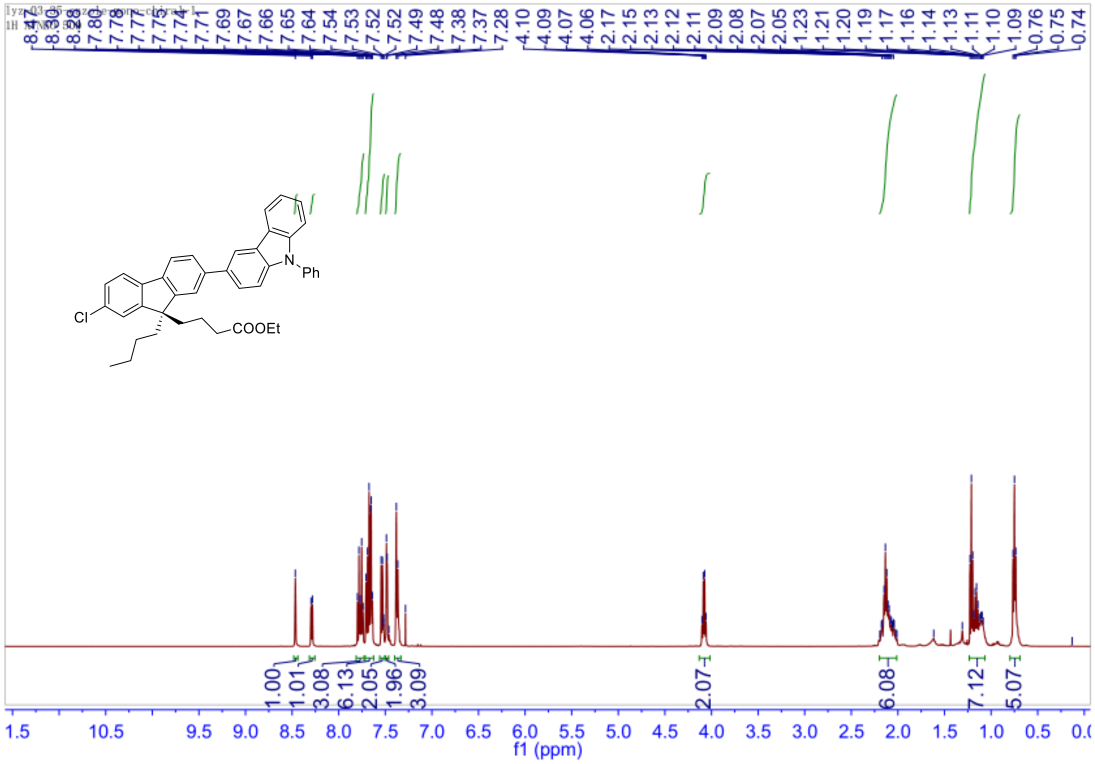

^{13}C NMR (126 MHz, $CDCl_3$)-**33**

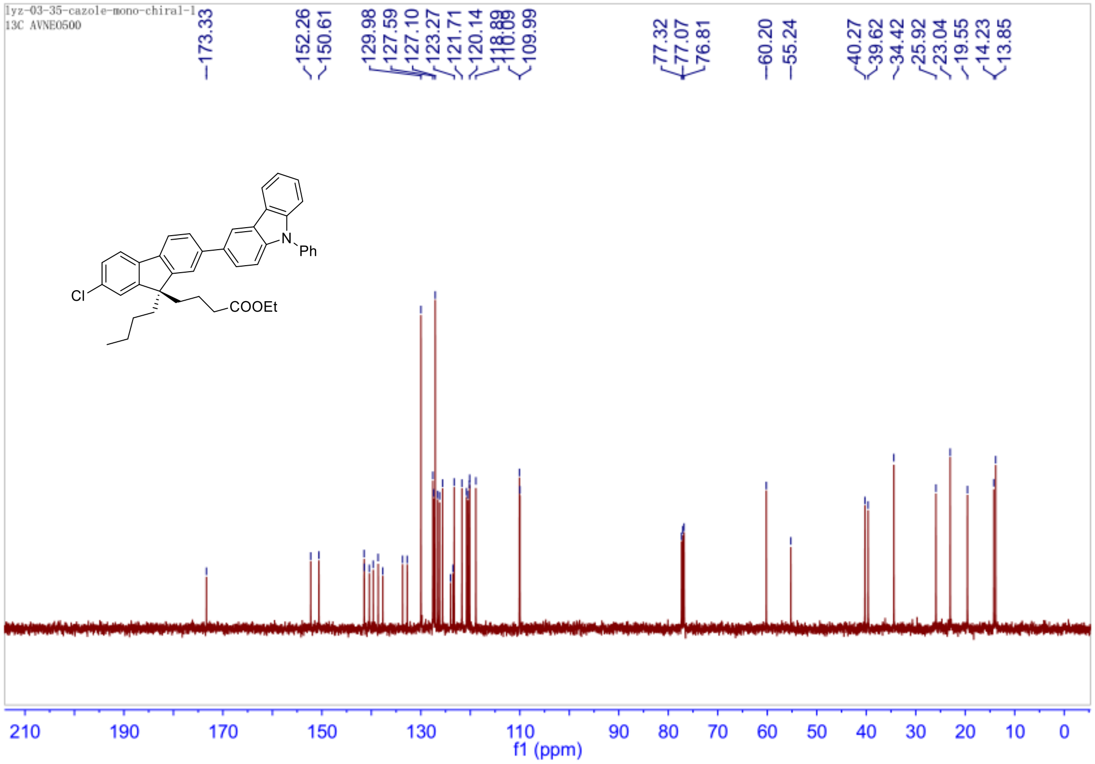

^{1}H NMR (500 MHz, $CDCl_3$)-**34**

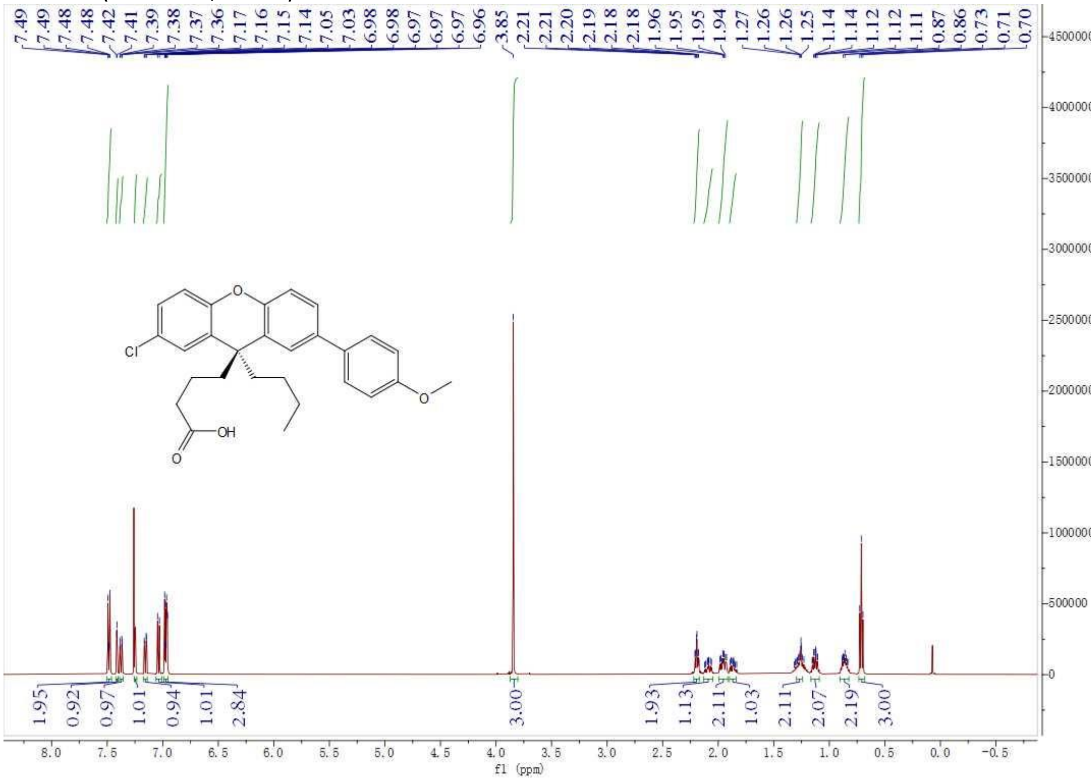

^{13}C NMR (126 MHz, $CDCl_3$)-**34**

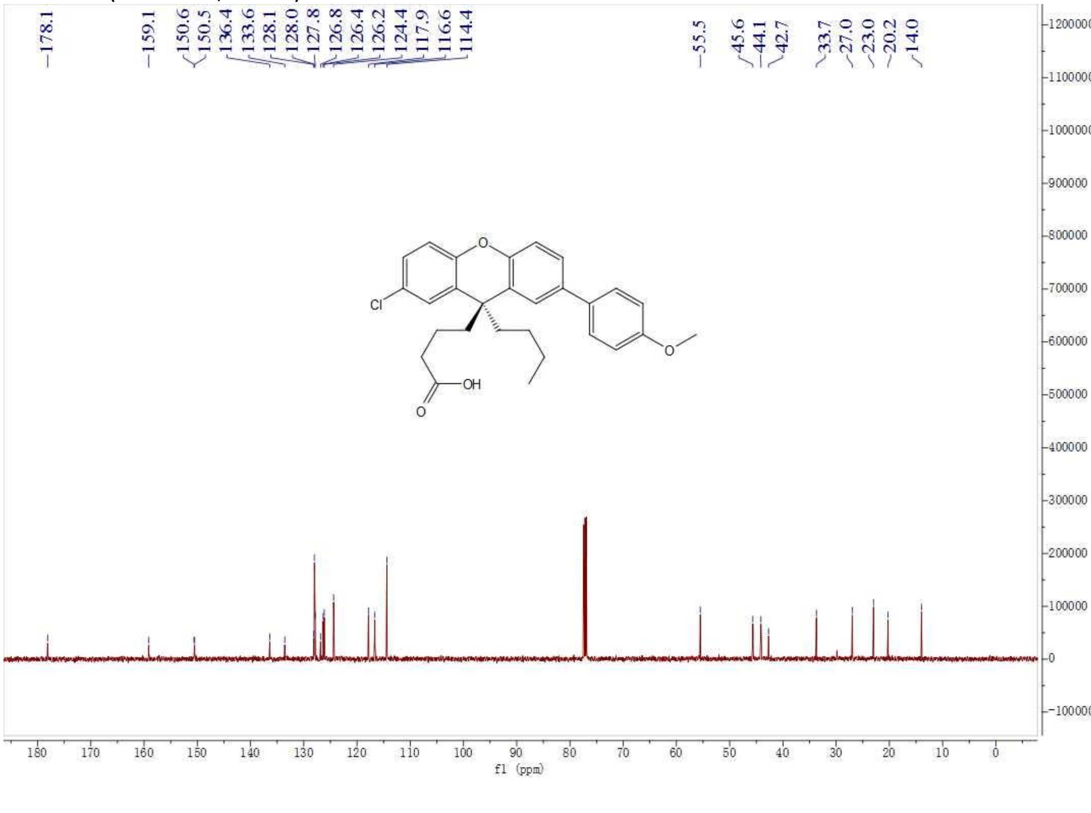

^{1}H NMR (500 MHz, $CDCl_3$)-**35**

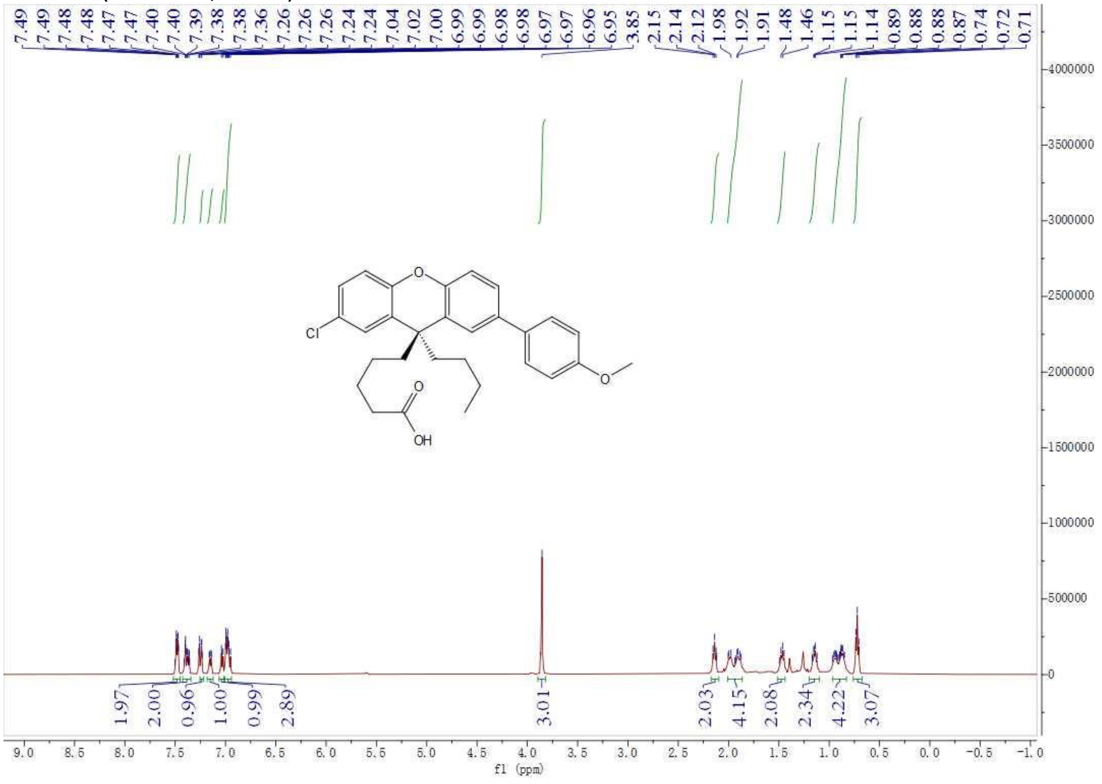

^{13}C NMR (126 MHz, $CDCl_3$)-**35**

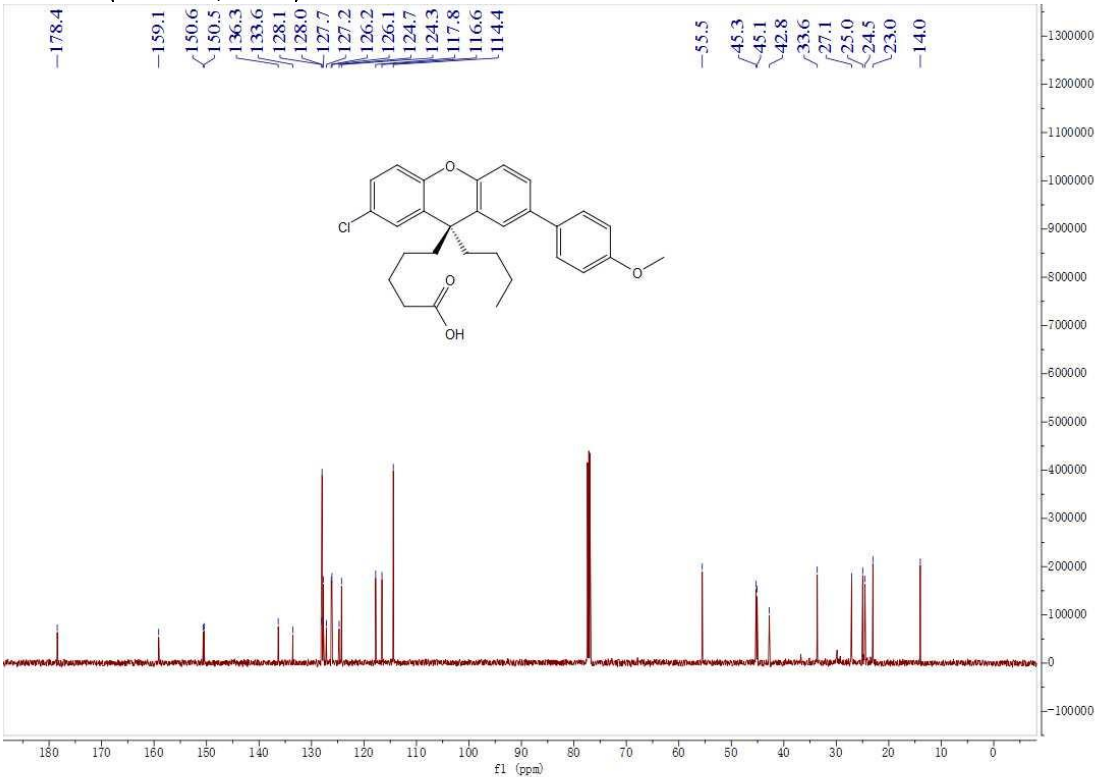

^{1}H NMR (500 MHz, $CDCl_3$)-**36**

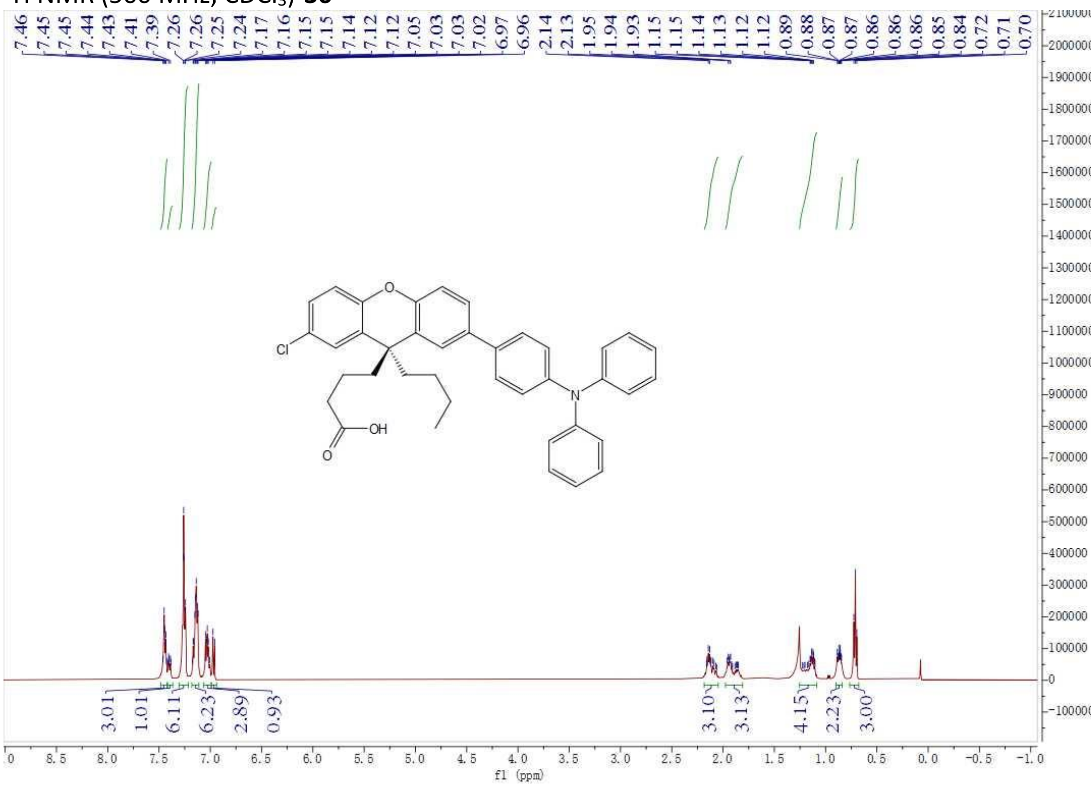

^{13}C NMR (126 MHz, $CDCl_3$)-**36**

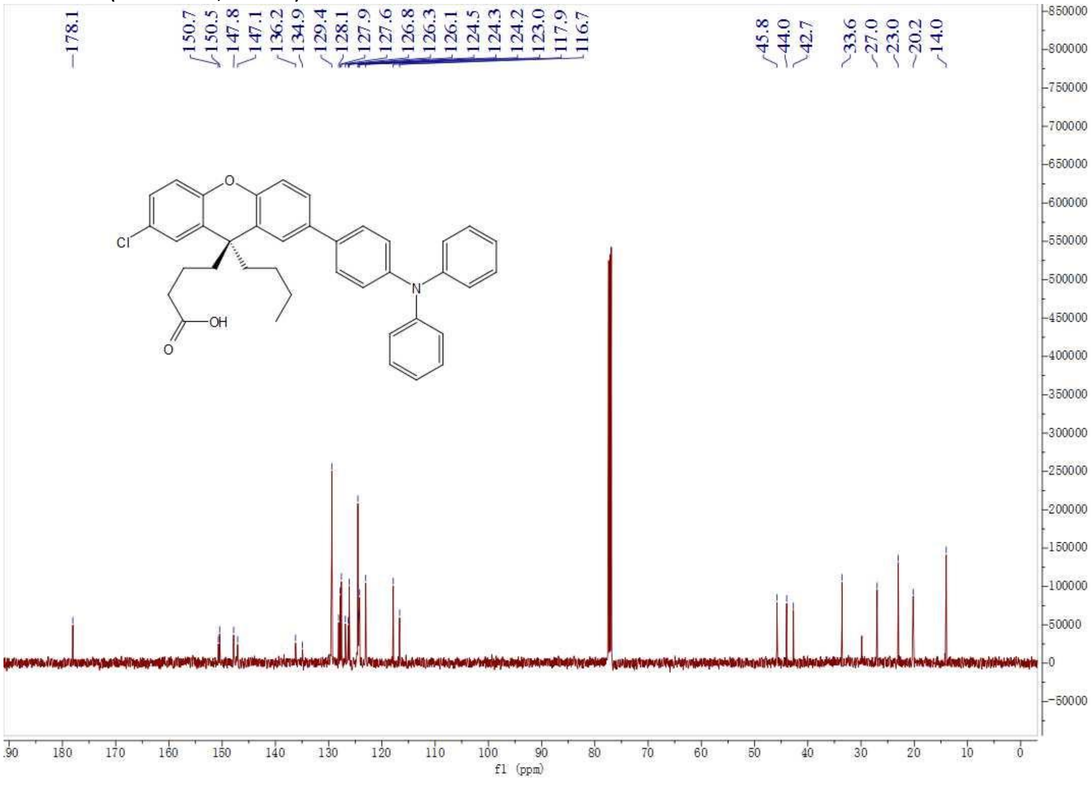

^{1}H NMR (500 MHz, $CDCl_3$)-**37**

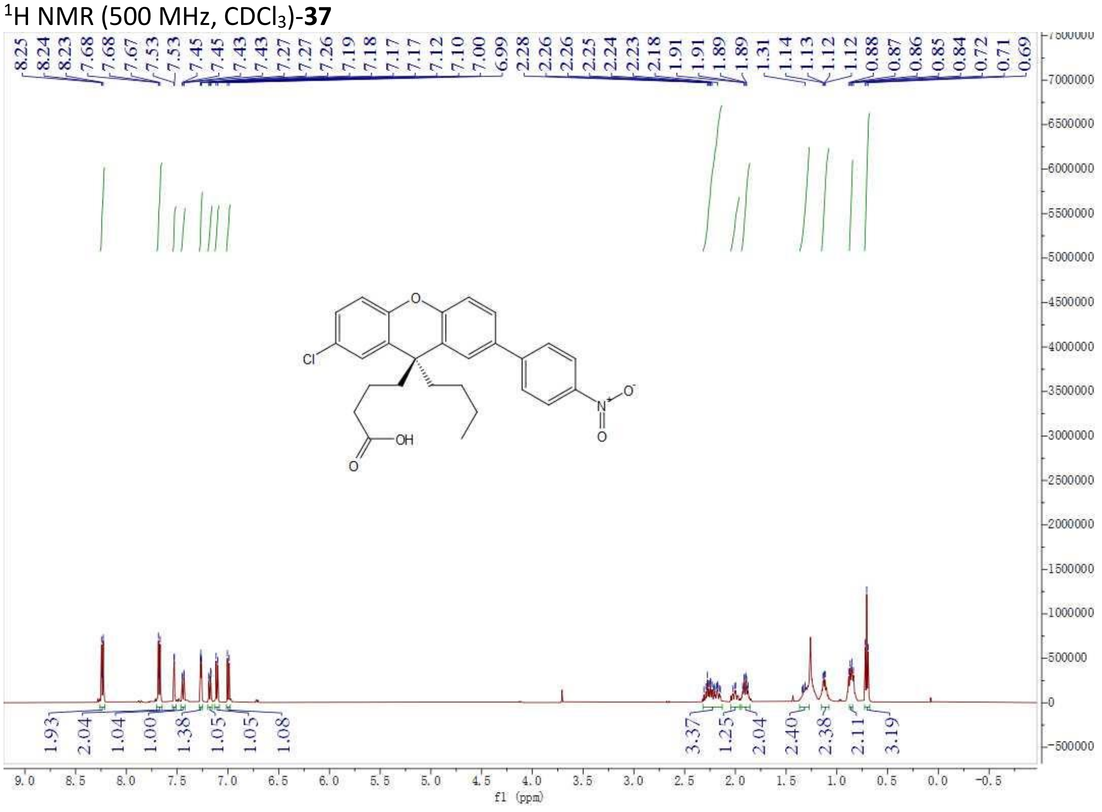

^{13}C NMR (126 MHz, $CDCl_3$)-**37**

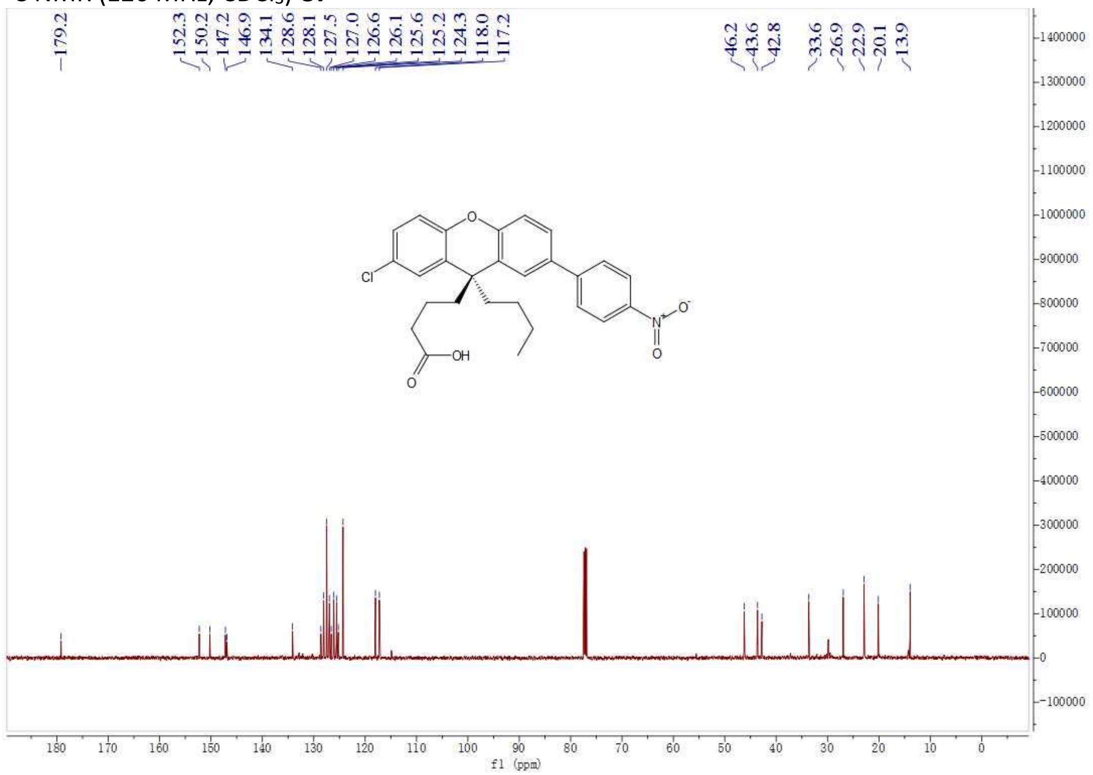

^{1}H NMR (500 MHz, $CDCl_3$)-**38**

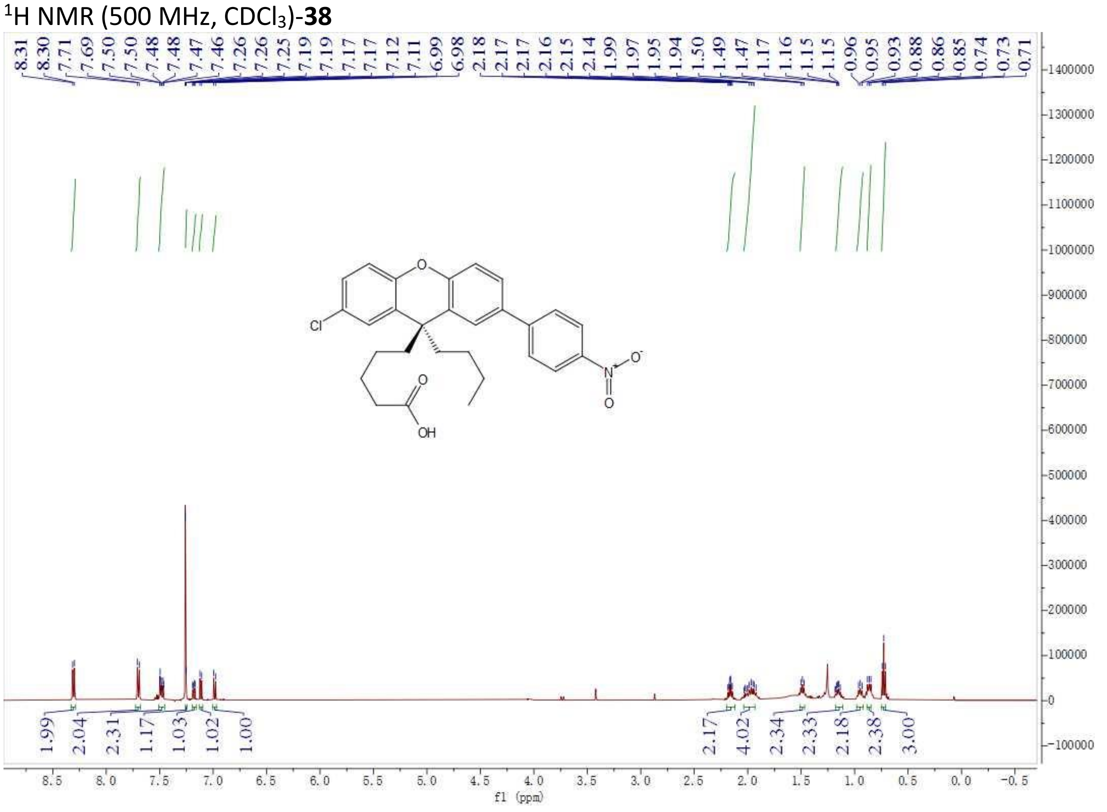

^{13}C NMR (126 MHz, $CDCl_3$)-**38**

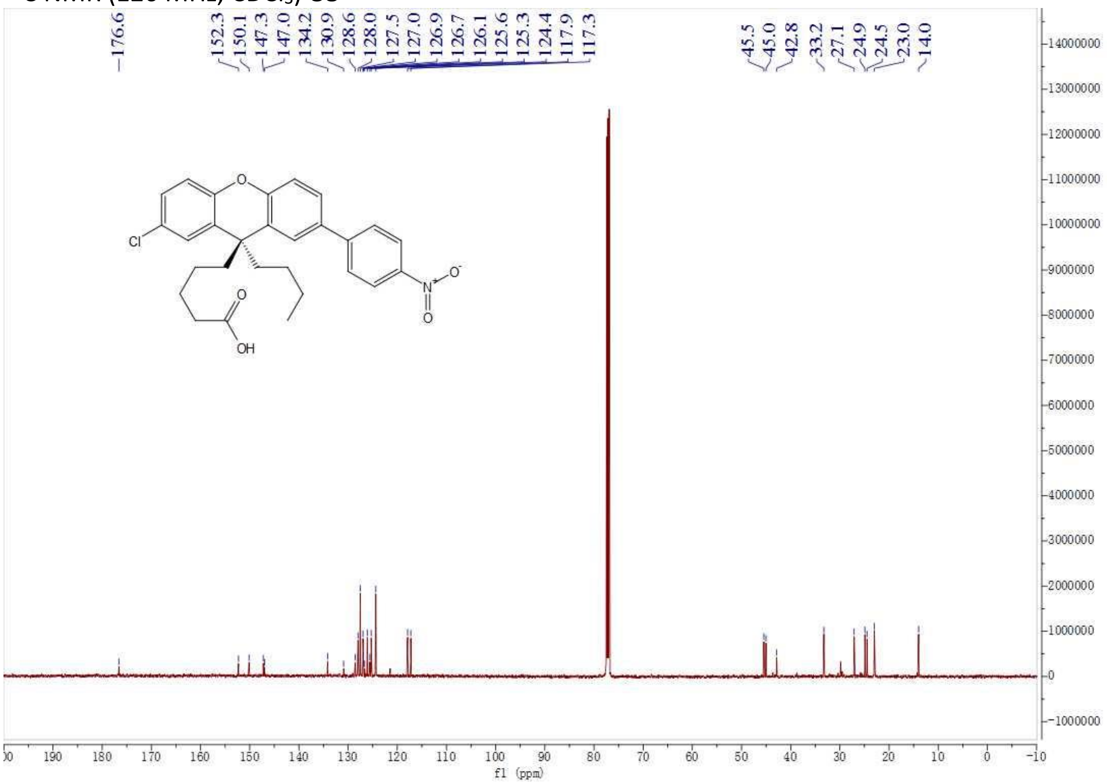

^{1}H NMR (500 MHz, $CDCl_3$)-**39**

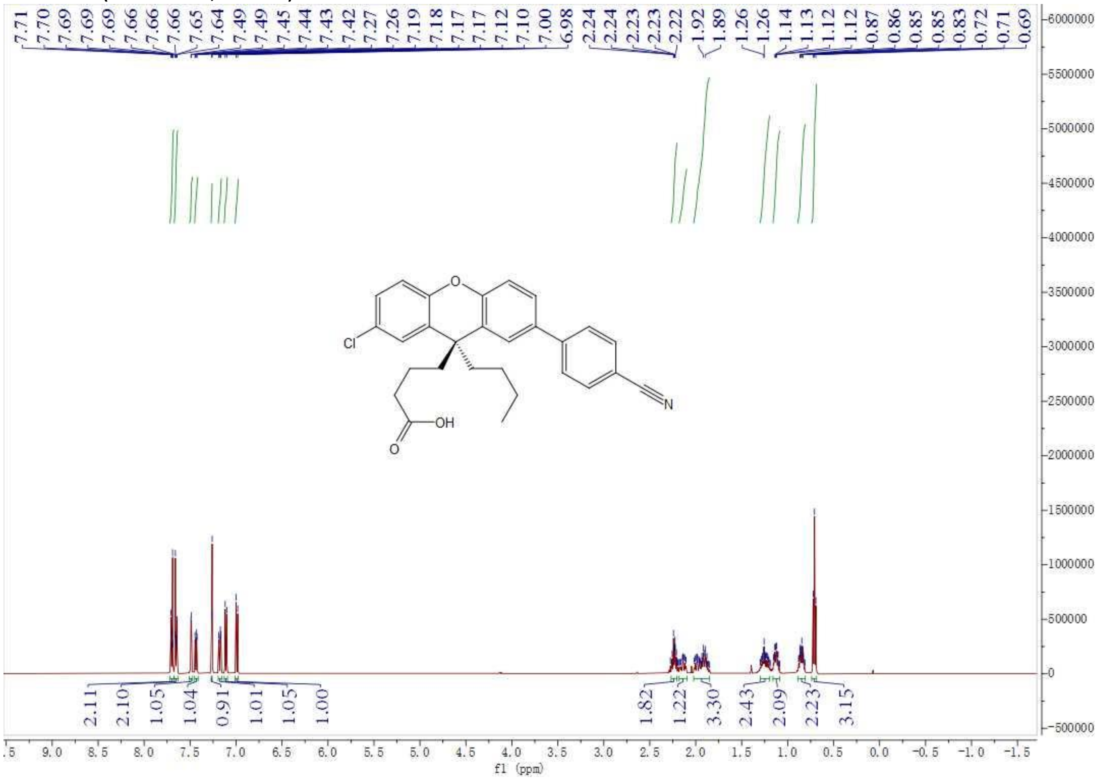

^{13}C NMR (126 MHz, $CDCl_3$)-**39**

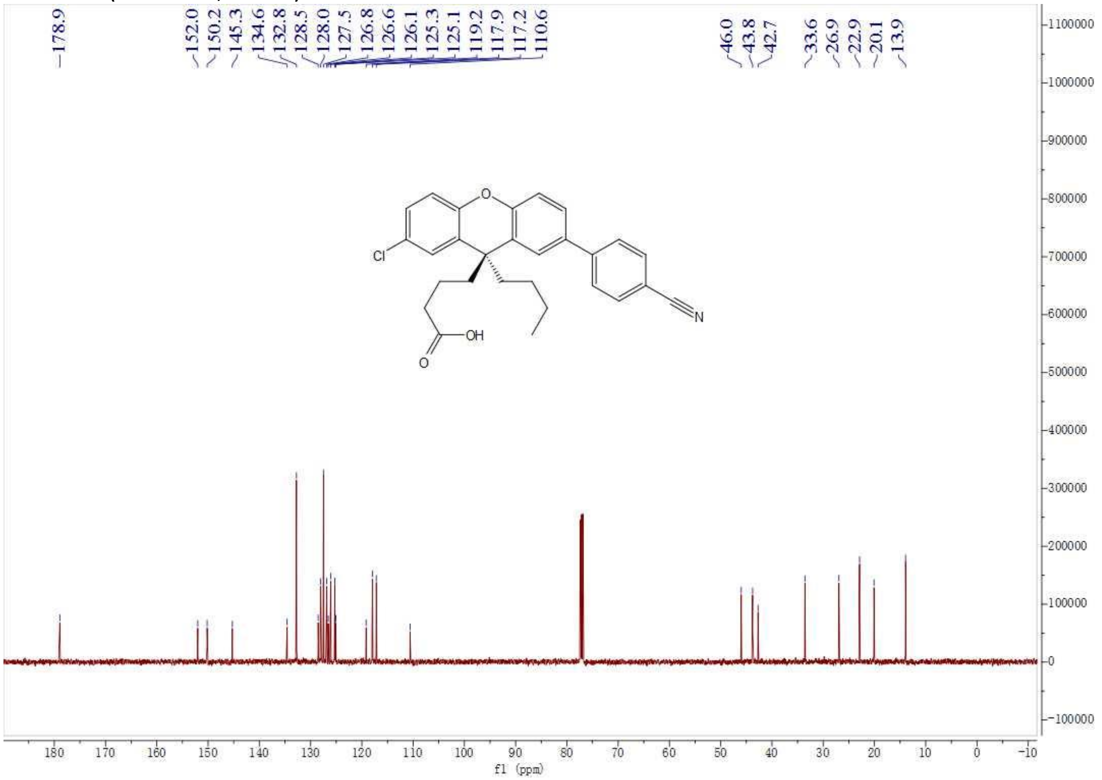

^{1}H NMR (500 MHz, $CDCl_3$)-**40**

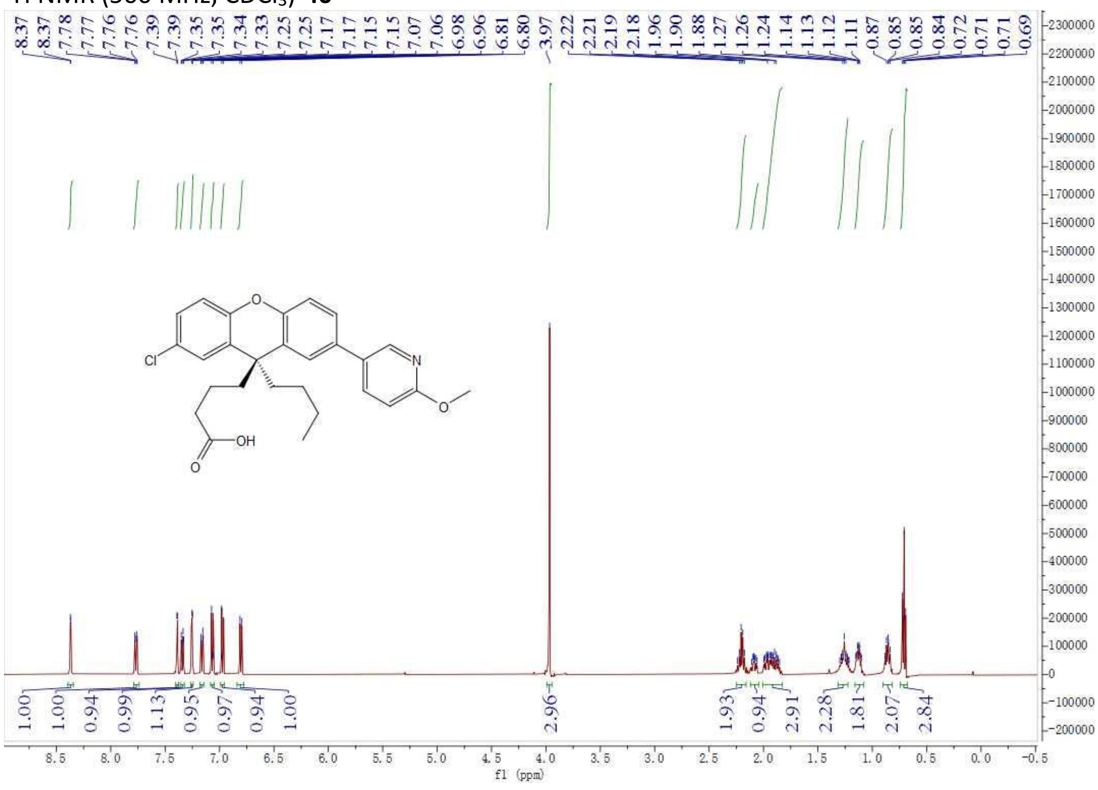

^{13}C NMR (101 MHz, $CDCl_3$)-**40**

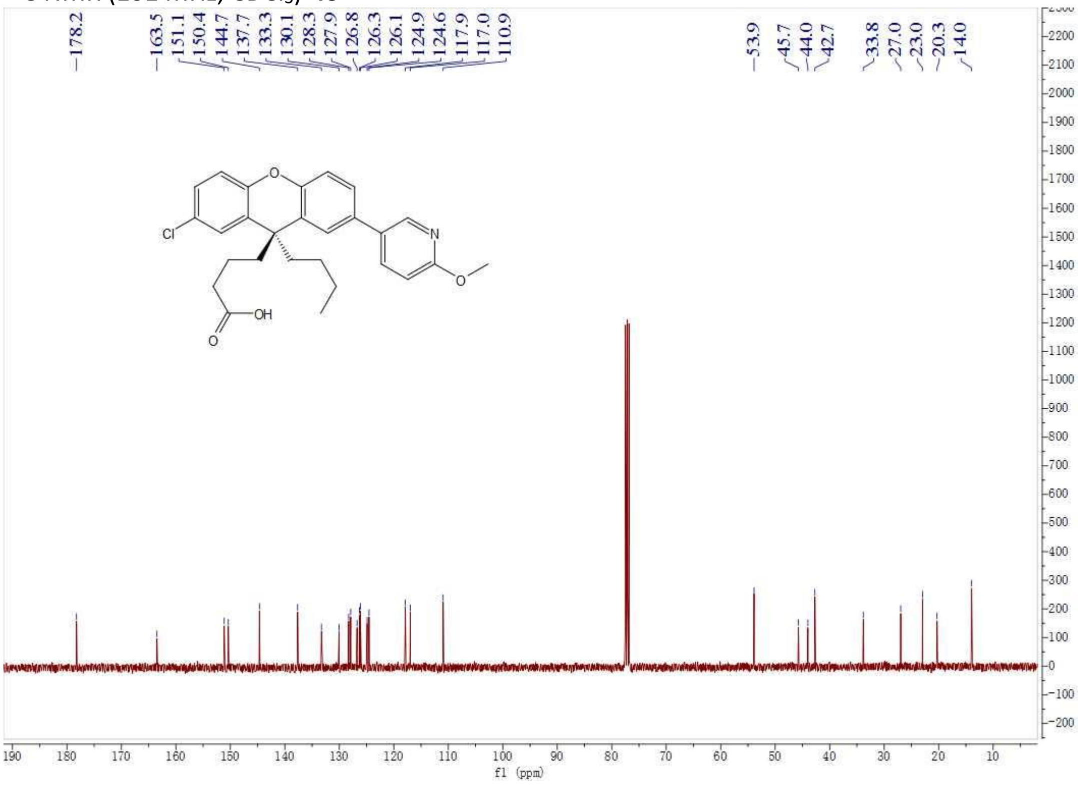

^{1}H NMR (400 MHz, $CDCl_3$)-**41**

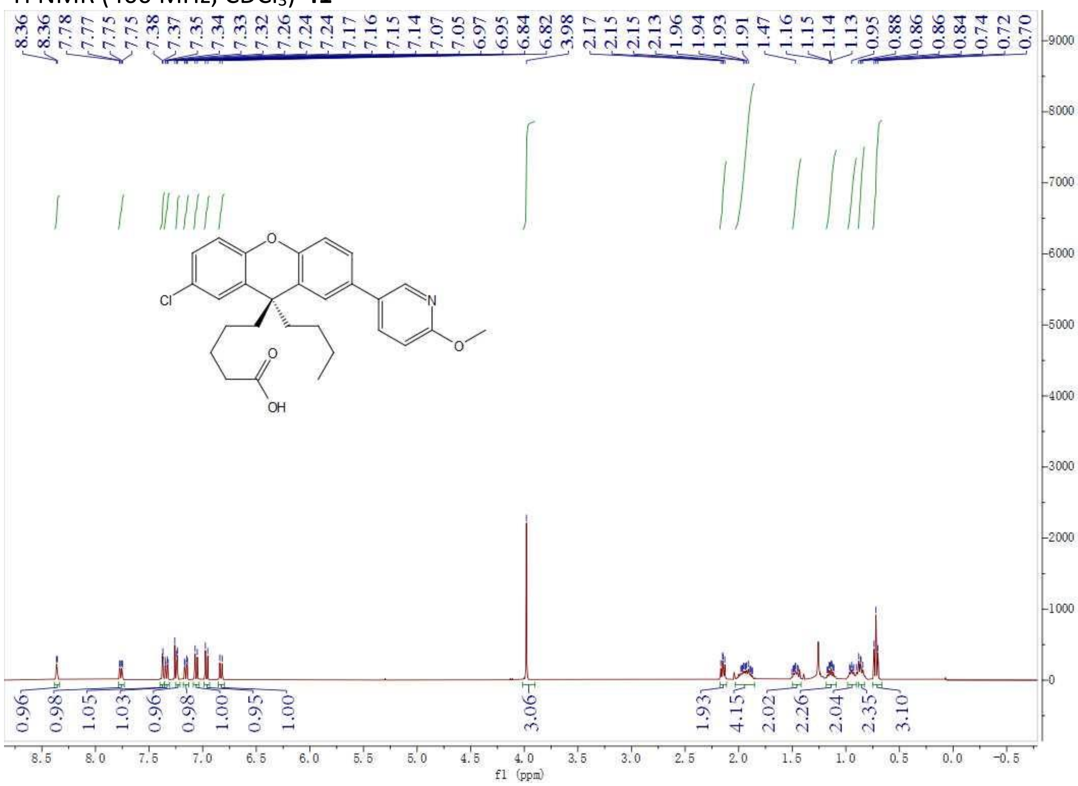

^{13}C NMR (126 MHz, $CDCl_3$)-**41**

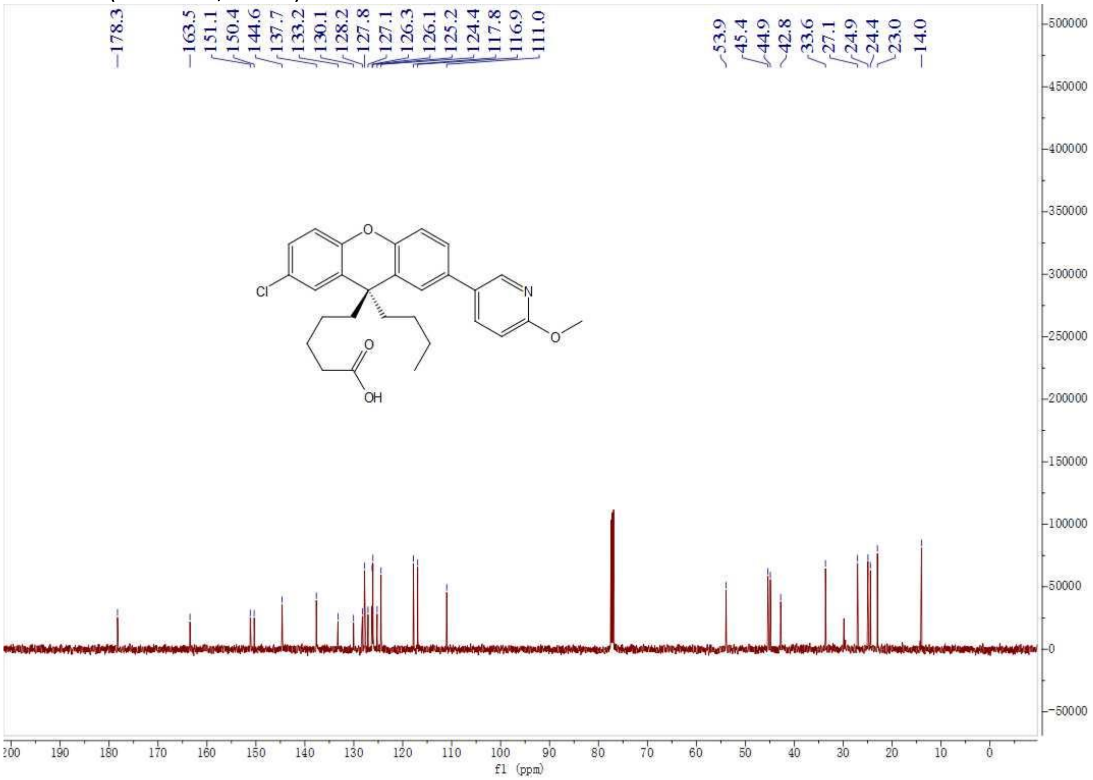

^{1}H NMR (500 MHz, $CDCl_3$)-**42**

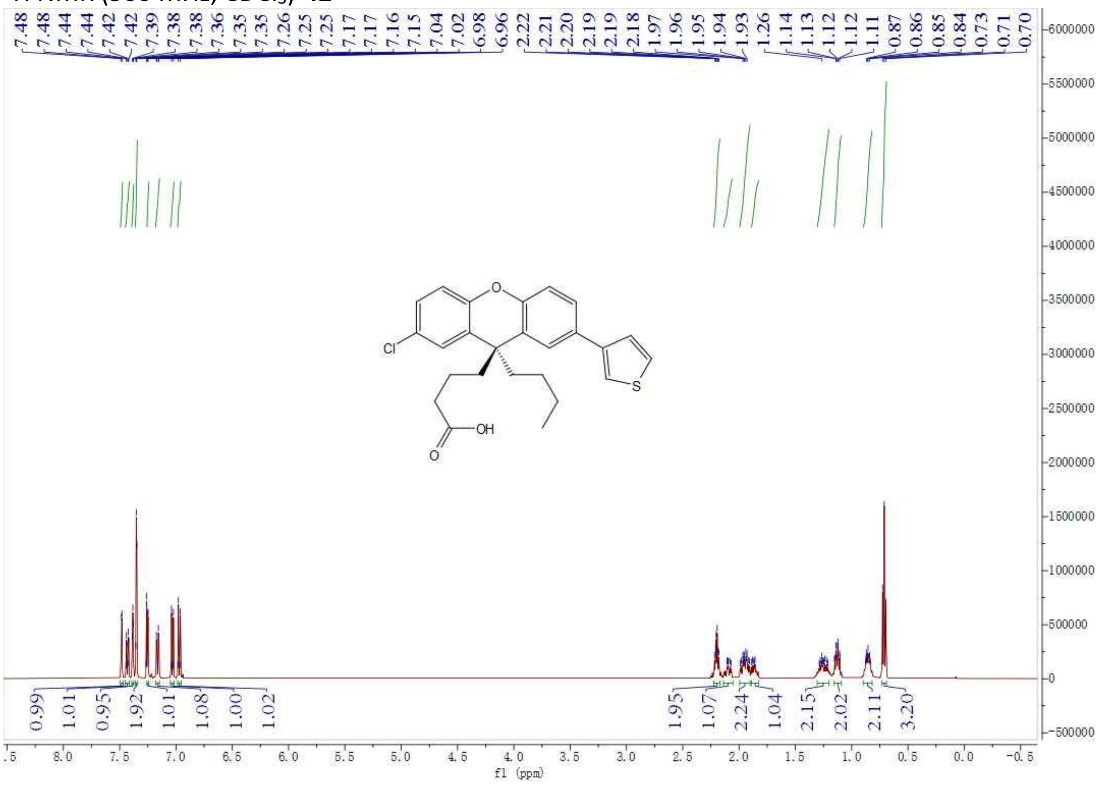

^{13}C NMR (126 MHz, $CDCl_3$)-**42**

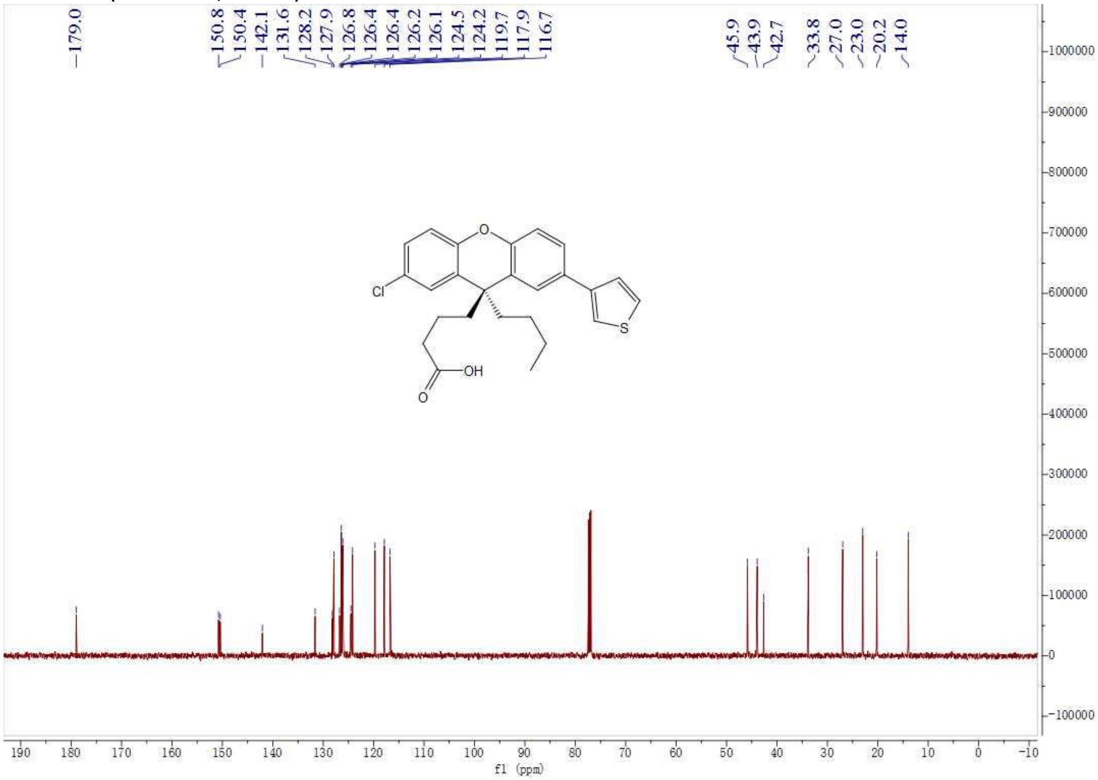

^1H NMR (500 MHz, $CDCl_3$)-**43**

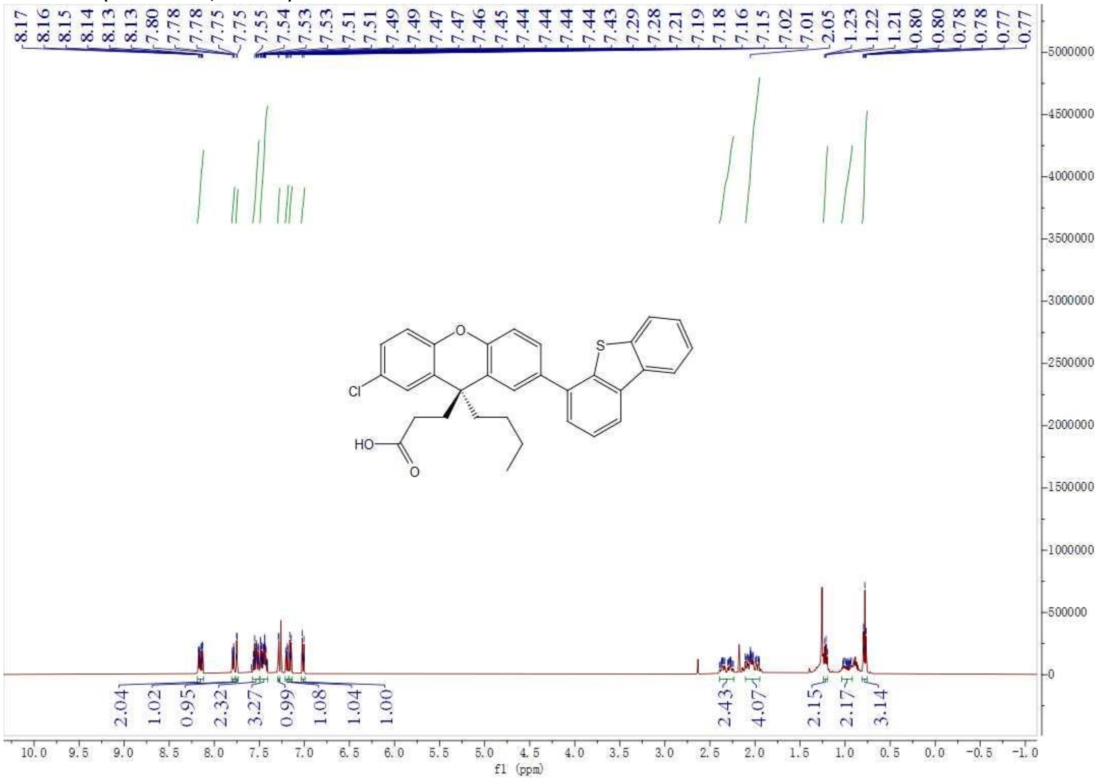

^{13}C NMR (126 MHz, $CDCl_3$)-**43**

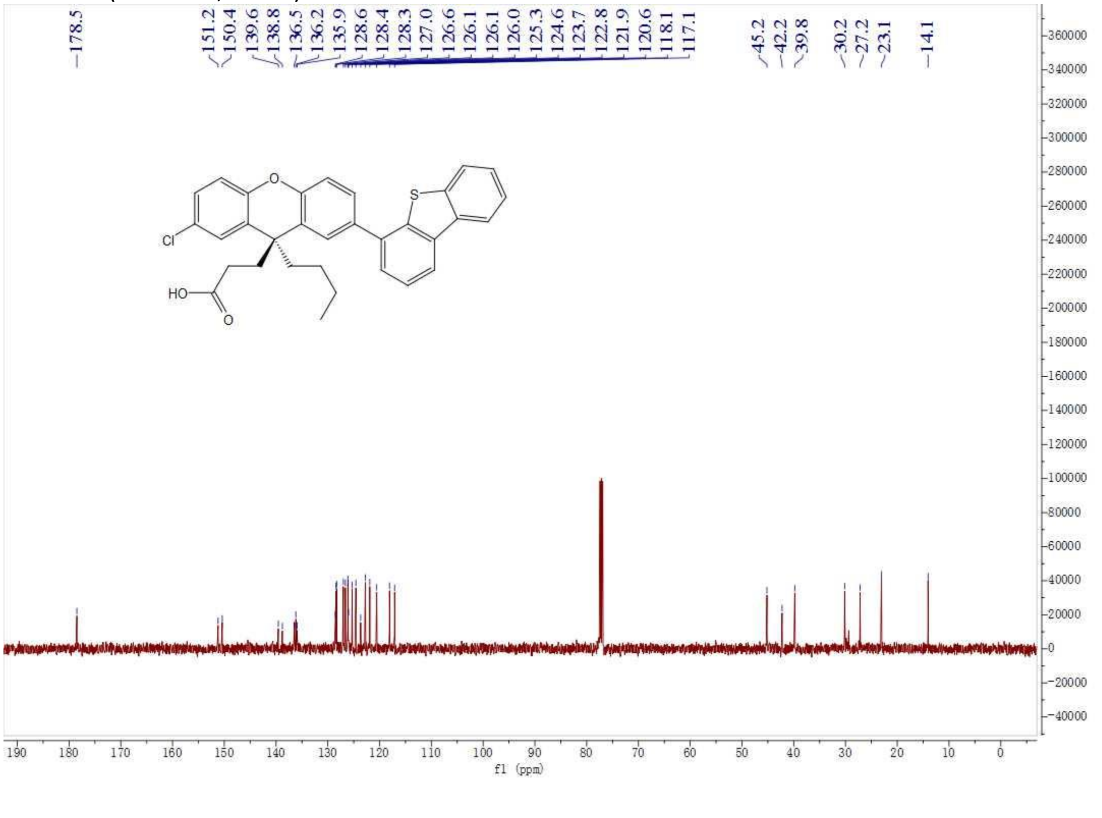

^{1}H NMR (500 MHz, $CDCl_3$)-**44**

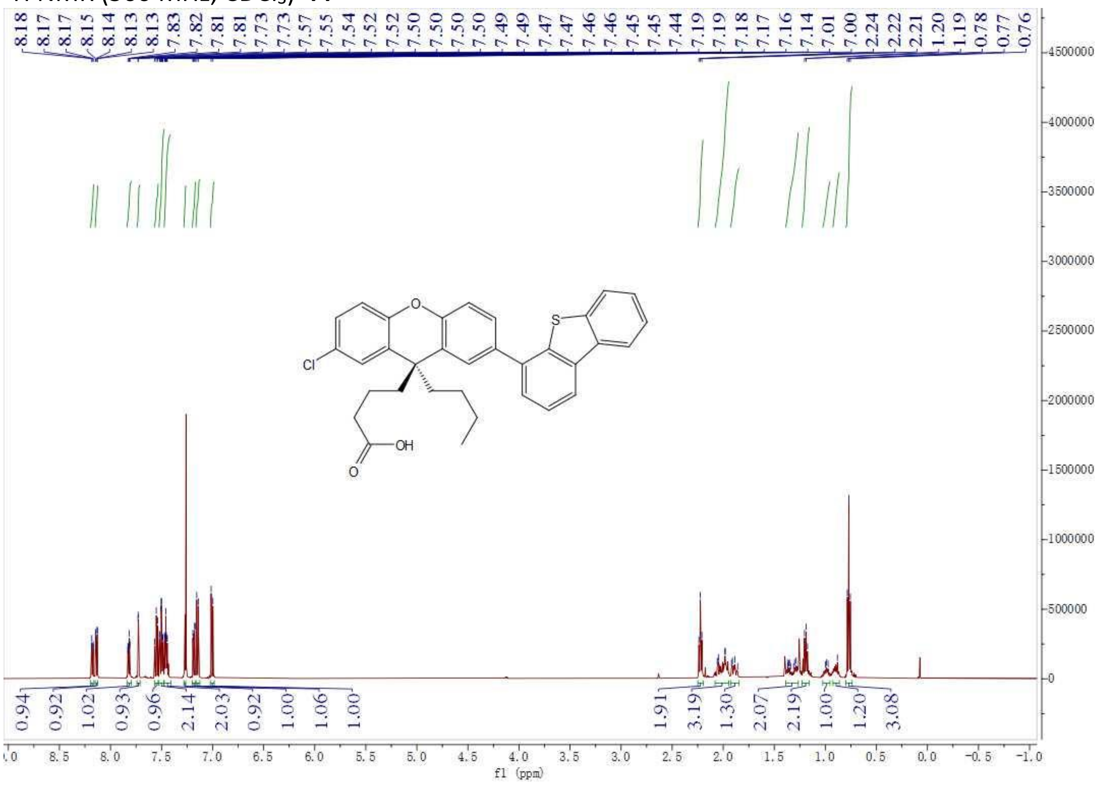

^{13}C NMR (126 MHz, $CDCl_3$)-**44**

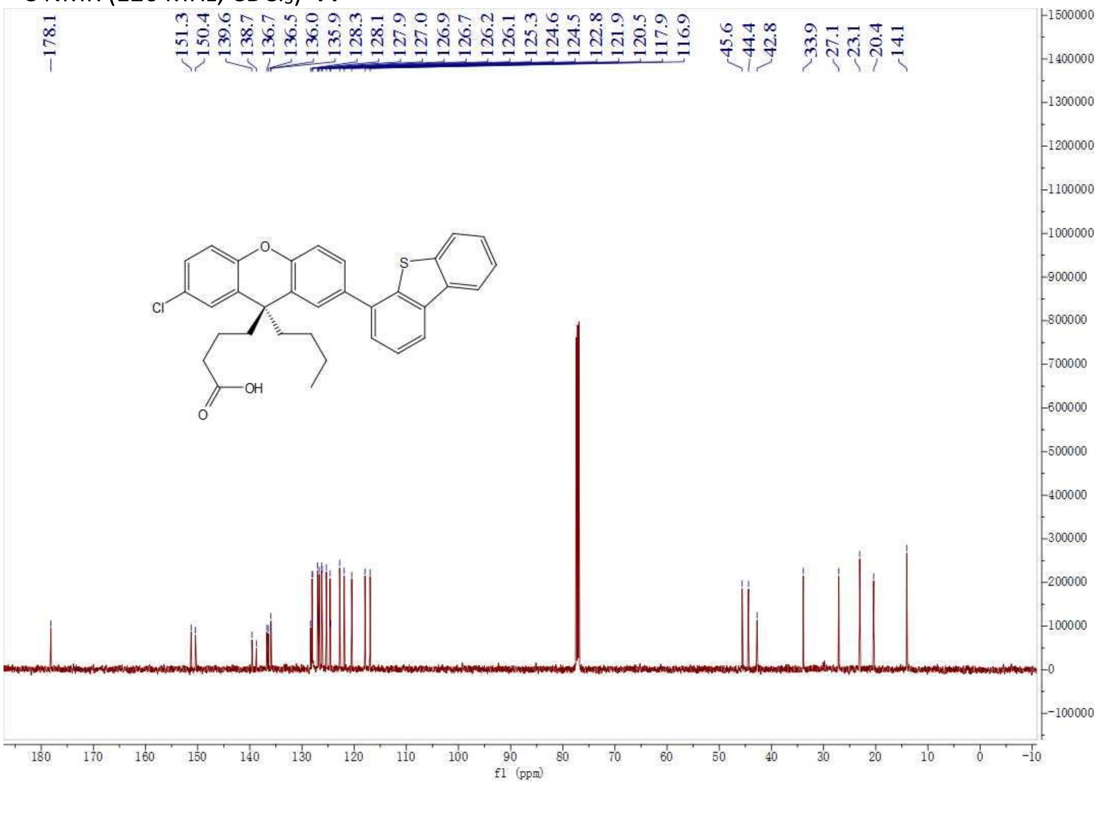

^{1}H NMR (400 MHz, $CDCl_3$)-**45**

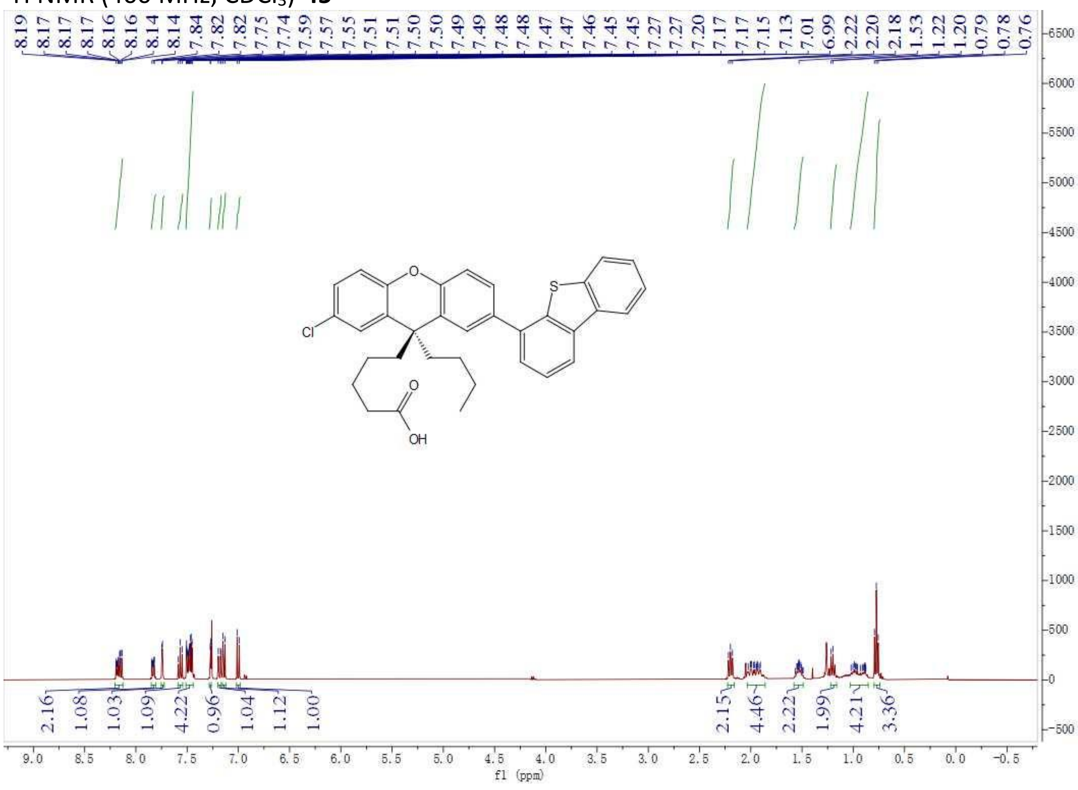

^{13}C NMR (126 MHz, $CDCl_3$)-**45**

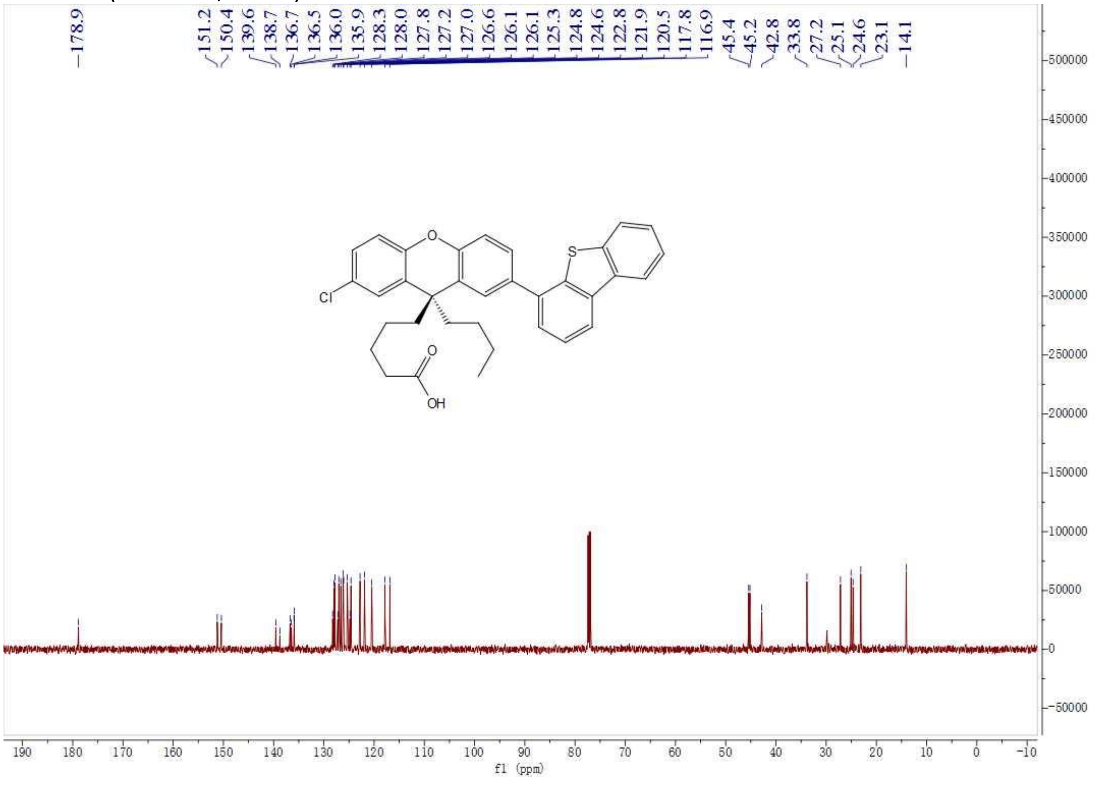

^{1}H NMR (500 MHz, $CDCl_3$)-**46**

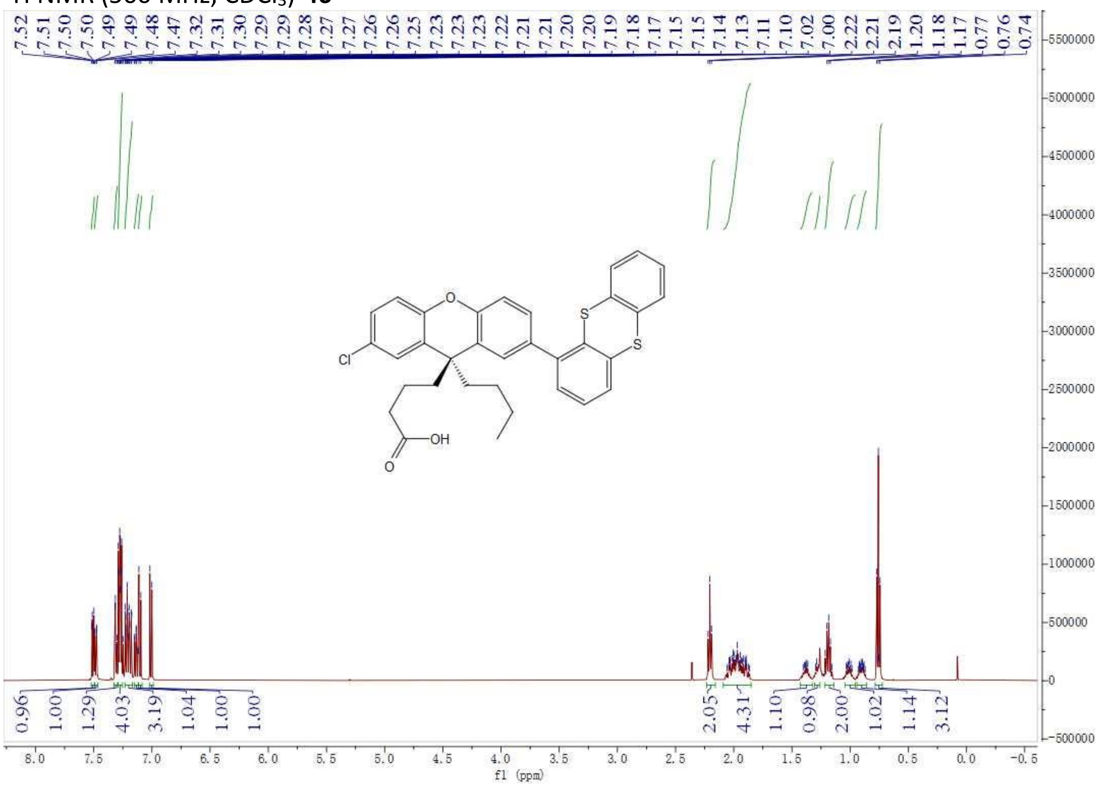

^{13}C NMR (126 MHz, $CDCl_3$)-**46**

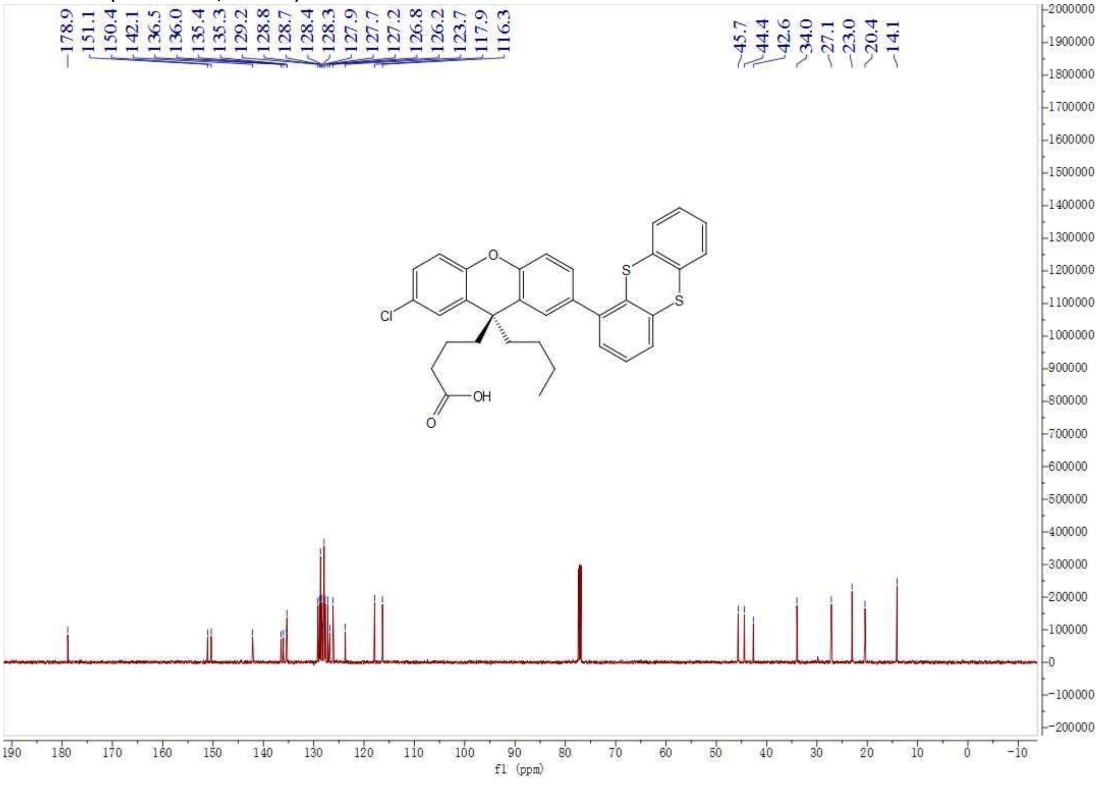

^{1}H NMR (500 MHz, $CDCl_3$)-**47**

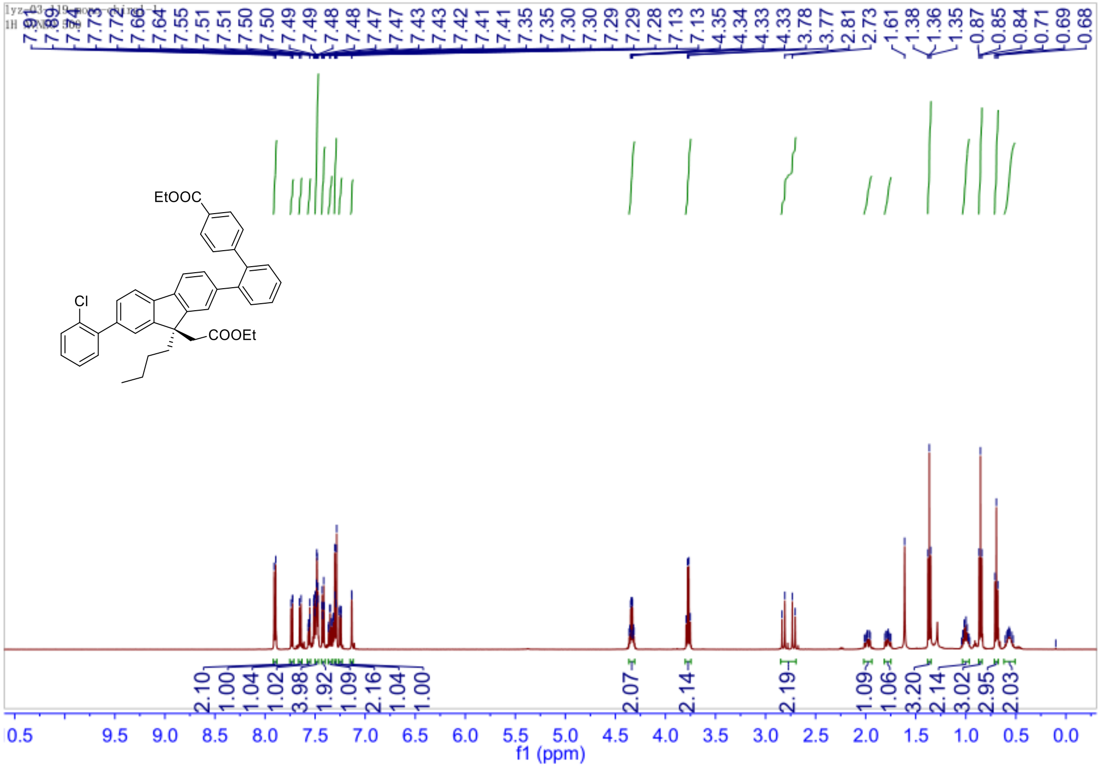

^{13}C NMR (126 MHz, $CDCl_3$)-**47**

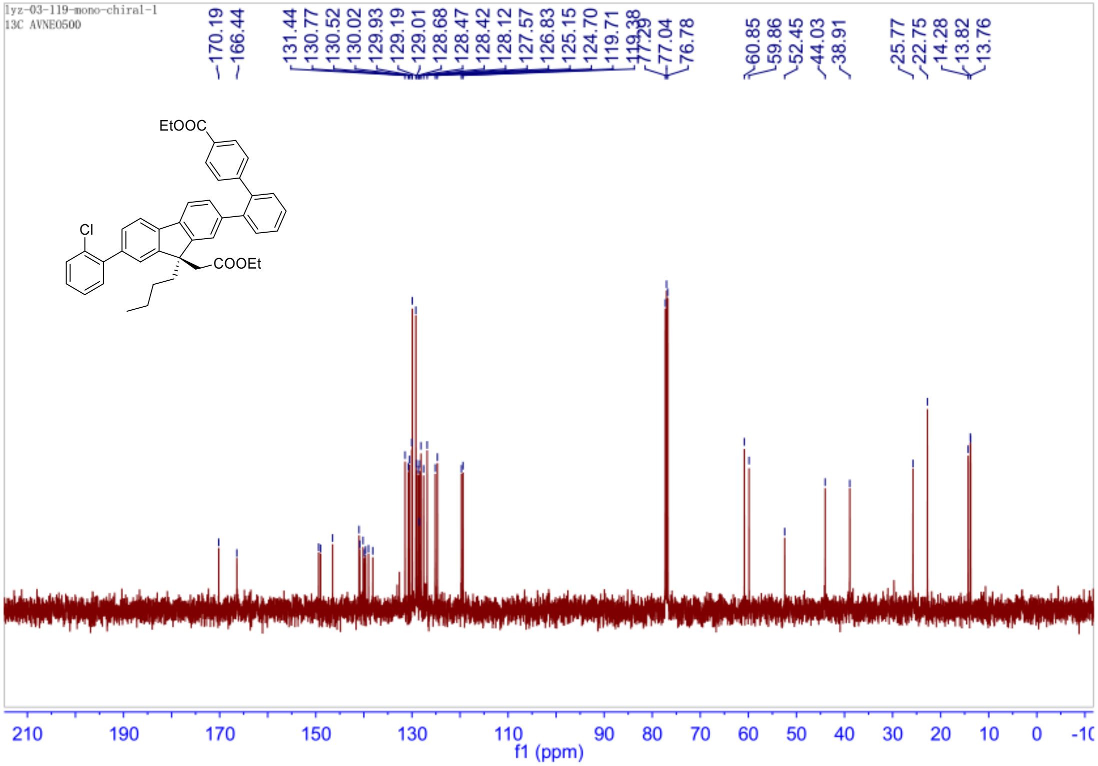

^{1}H NMR (500 MHz, $CDCl_3$)-**48**

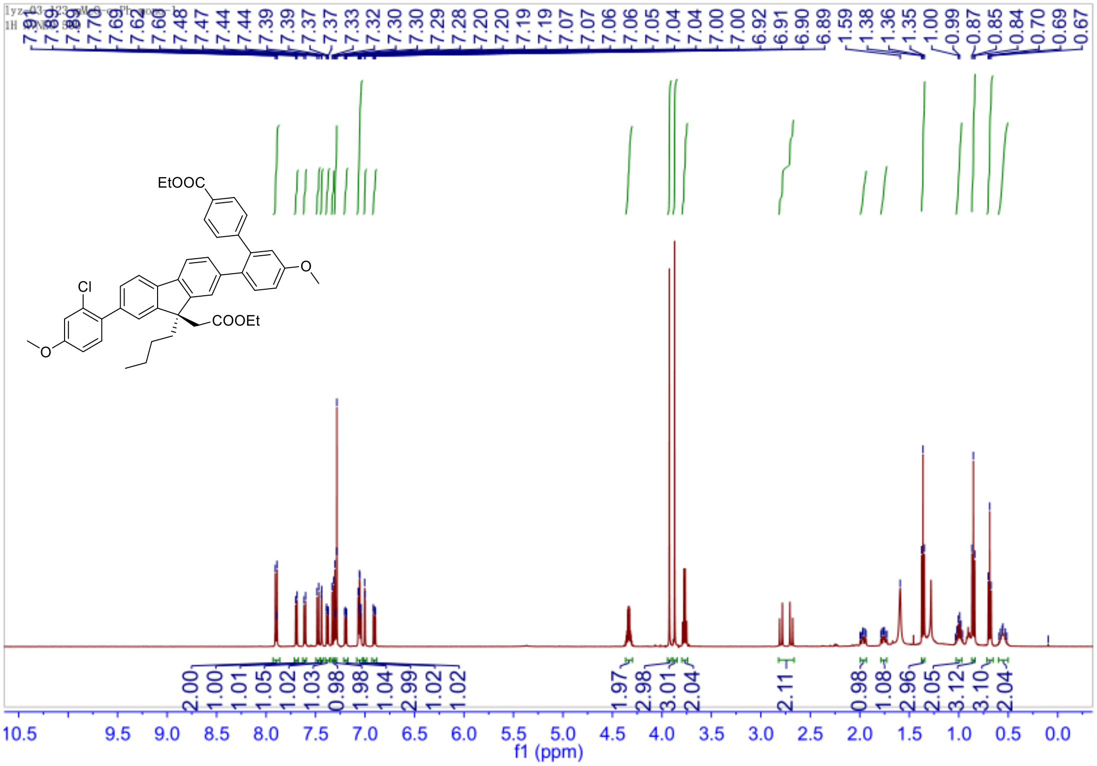

^{13}C NMR (126 MHz, $CDCl_3$)-**48**

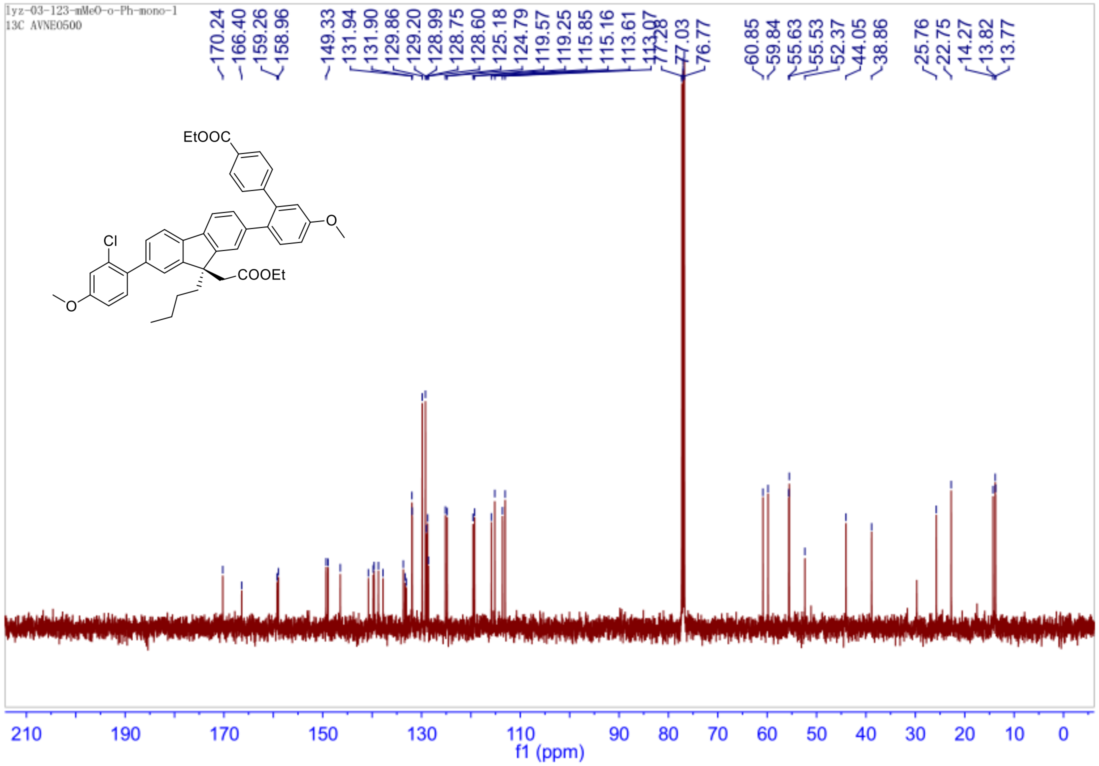

^{1}H NMR (400 MHz, $CDCl_3$)-**49**

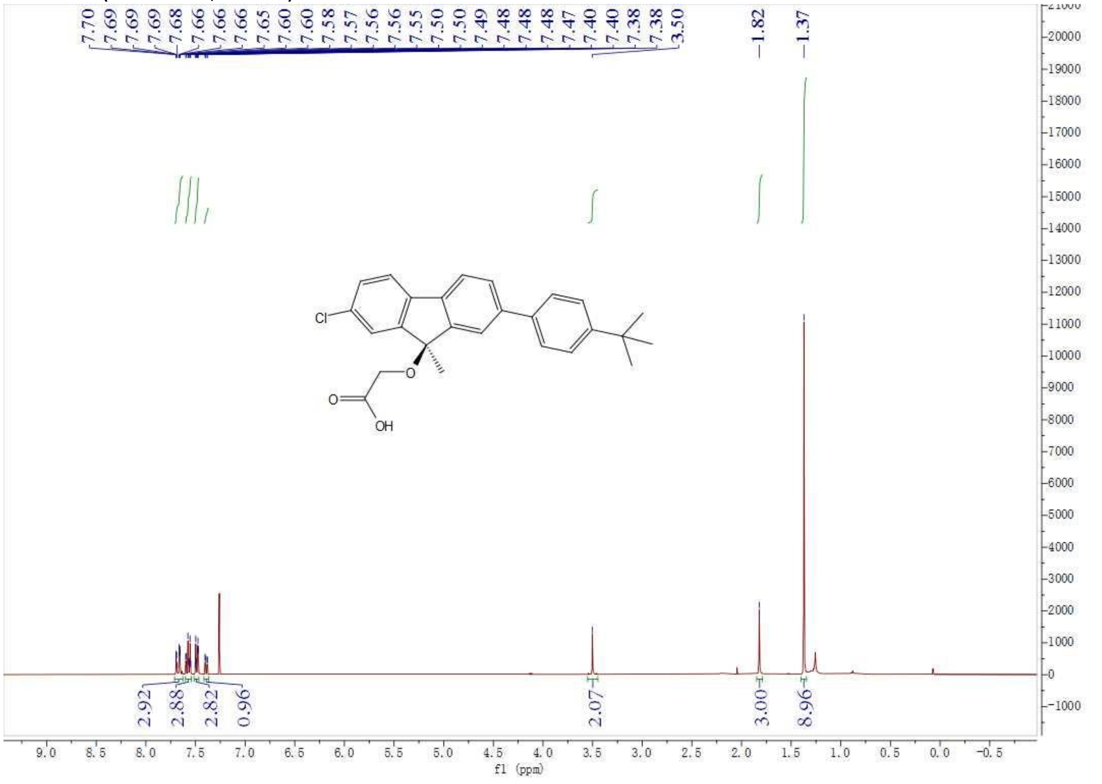

^{13}C NMR (126 MHz, $CDCl_3$)-**49**

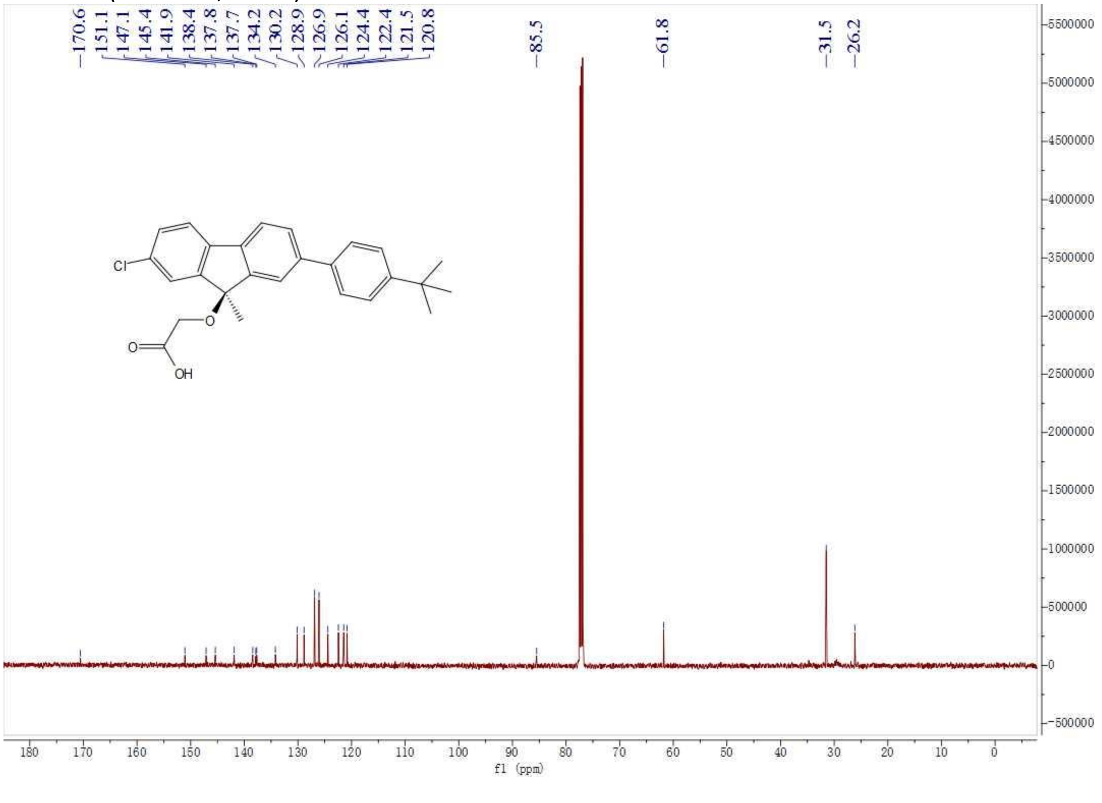

^{1}H NMR (500 MHz, $CDCl_3$)-**50**

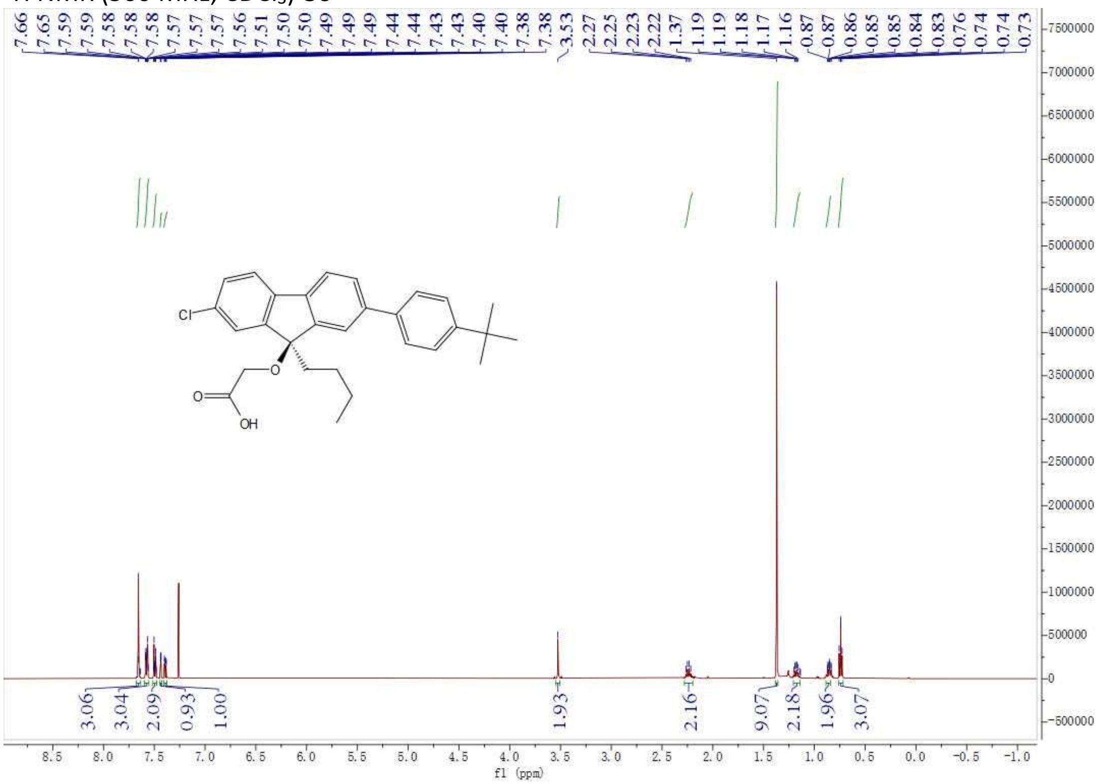

^{13}C NMR (126 MHz, $CDCl_3$)-**50**

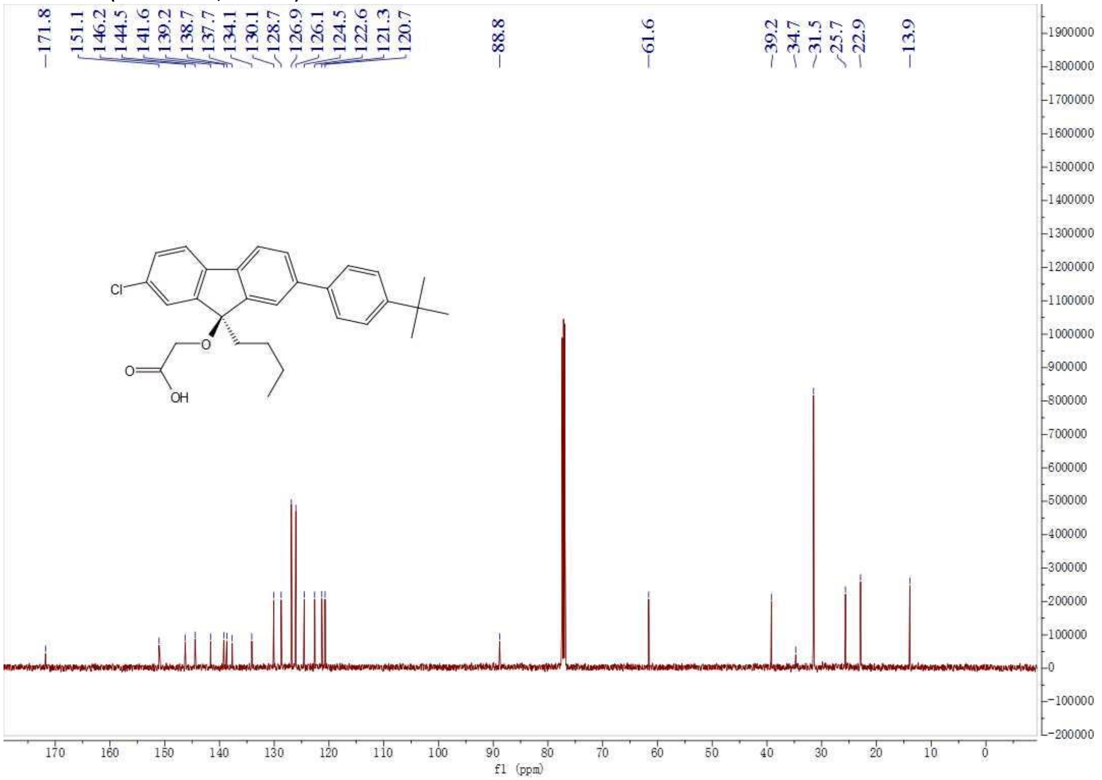

^{1}H NMR (500 MHz, $CDCl_3$)-**53** (coupling product of **53** from control experiment)

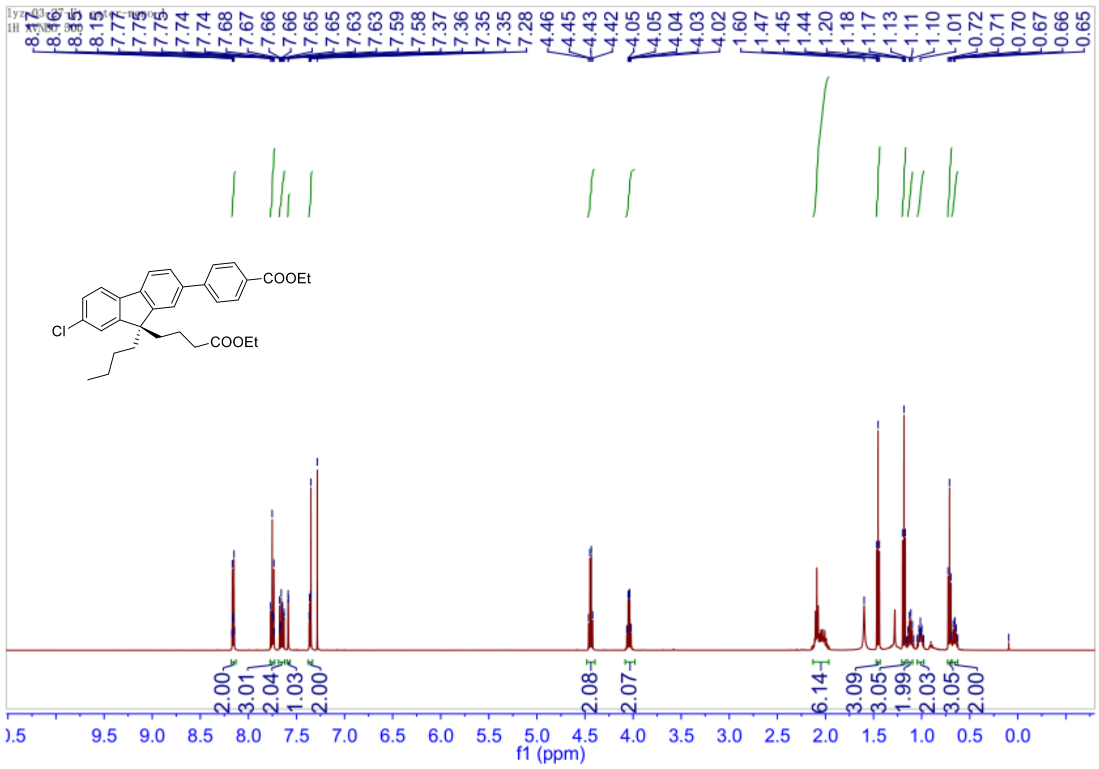

^{13}C NMR (126 MHz, $CDCl_3$)-**53** (coupling product of **53** from control experiment)

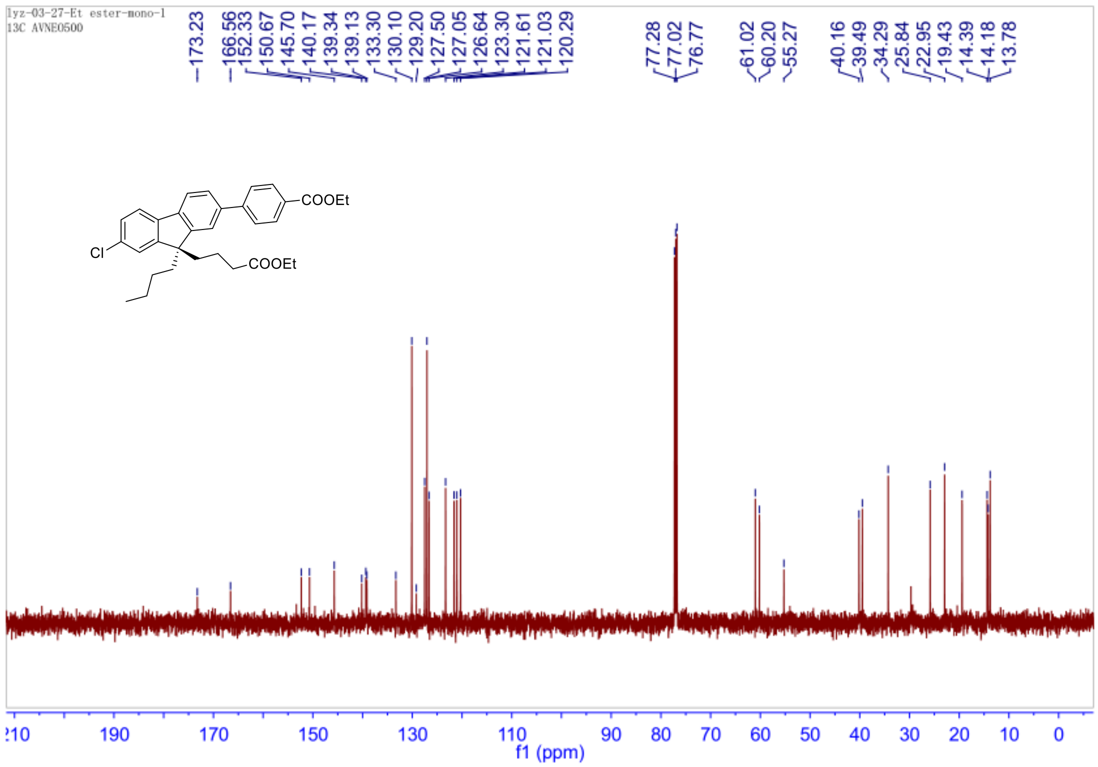

^{1}H NMR (500 MHz, $CDCl_3$)-**54**

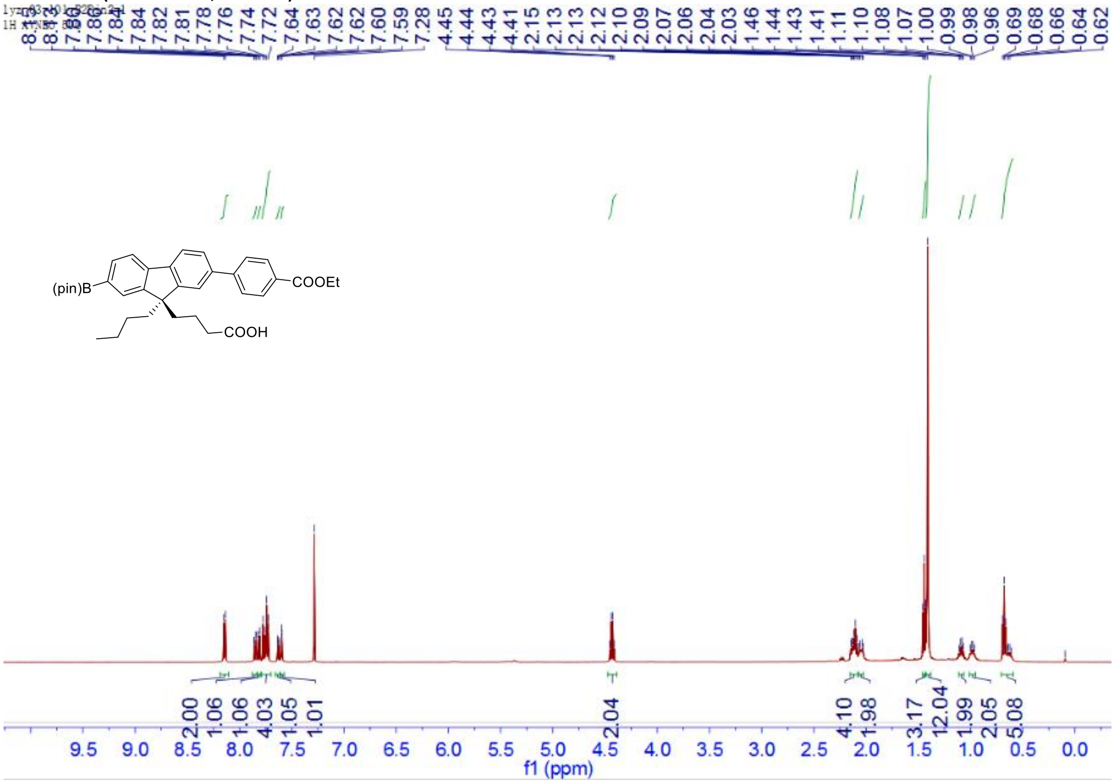

^{13}C NMR (126 MHz, $CDCl_3$)-**54**

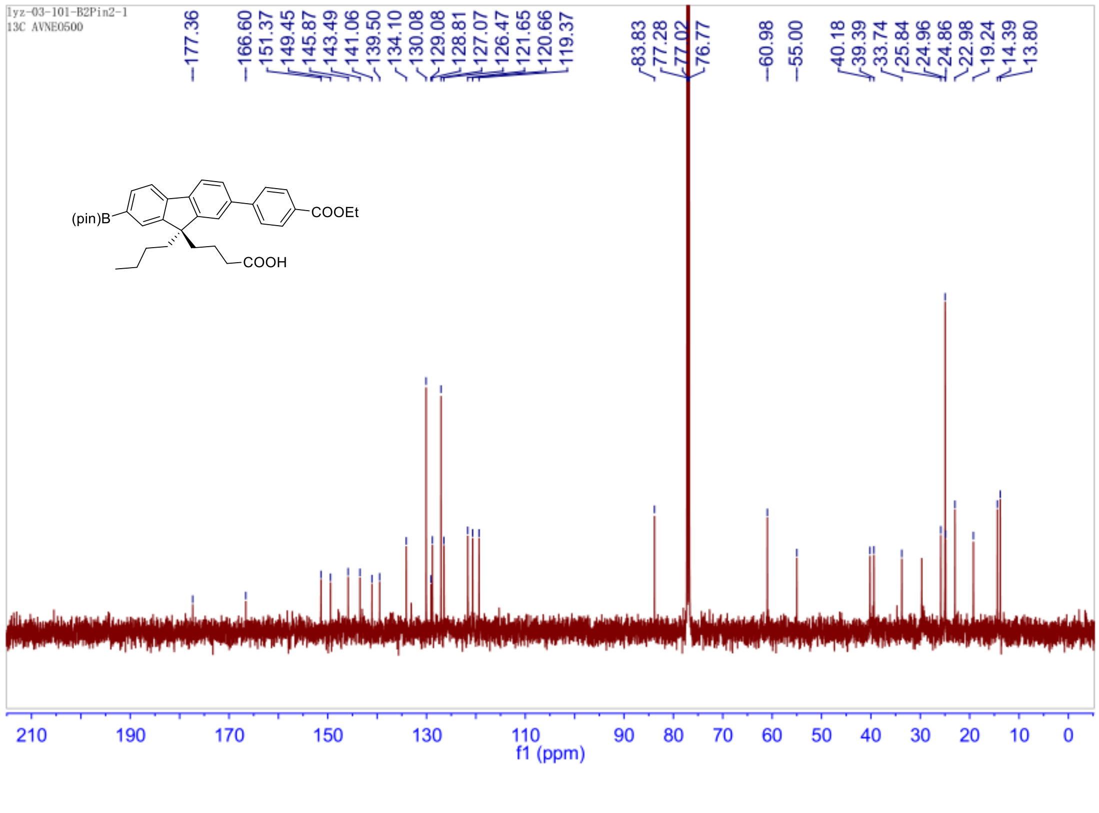

^{1}H NMR (500 MHz, $CDCl_3$)-**55**

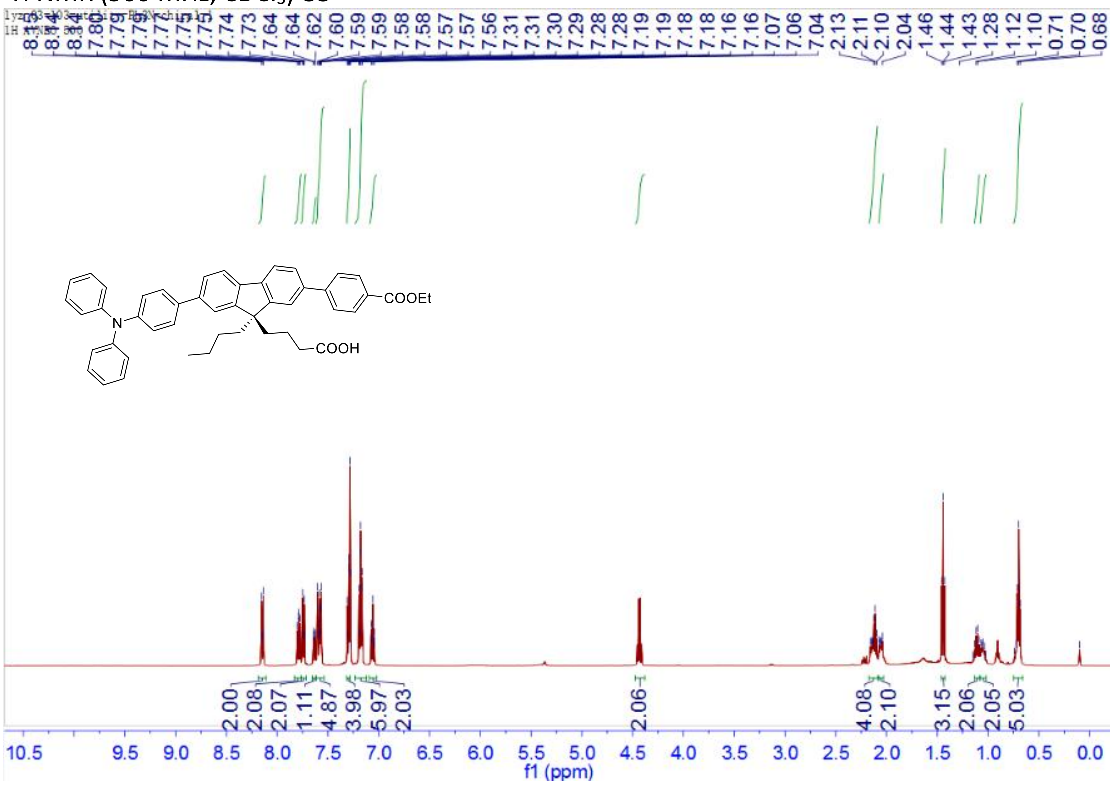

^{13}C NMR (126 MHz, $CDCl_3$)-**55**

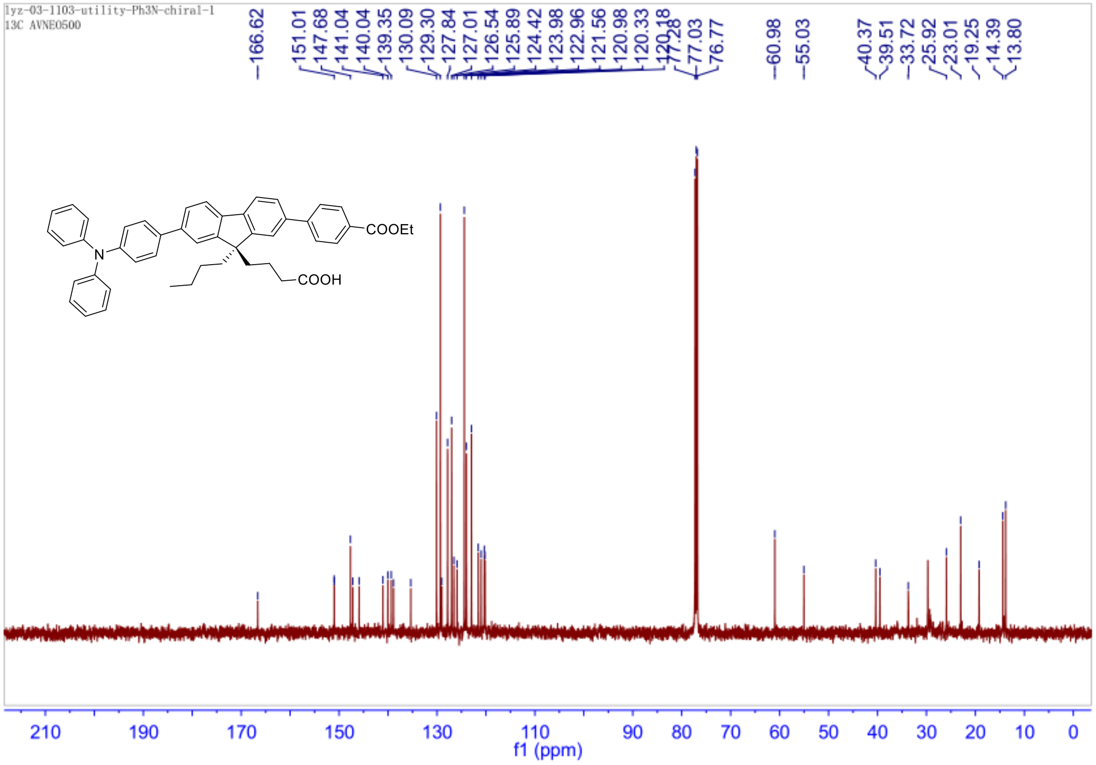

^{1}H NMR (500 MHz, $CDCl_3$)-**56**

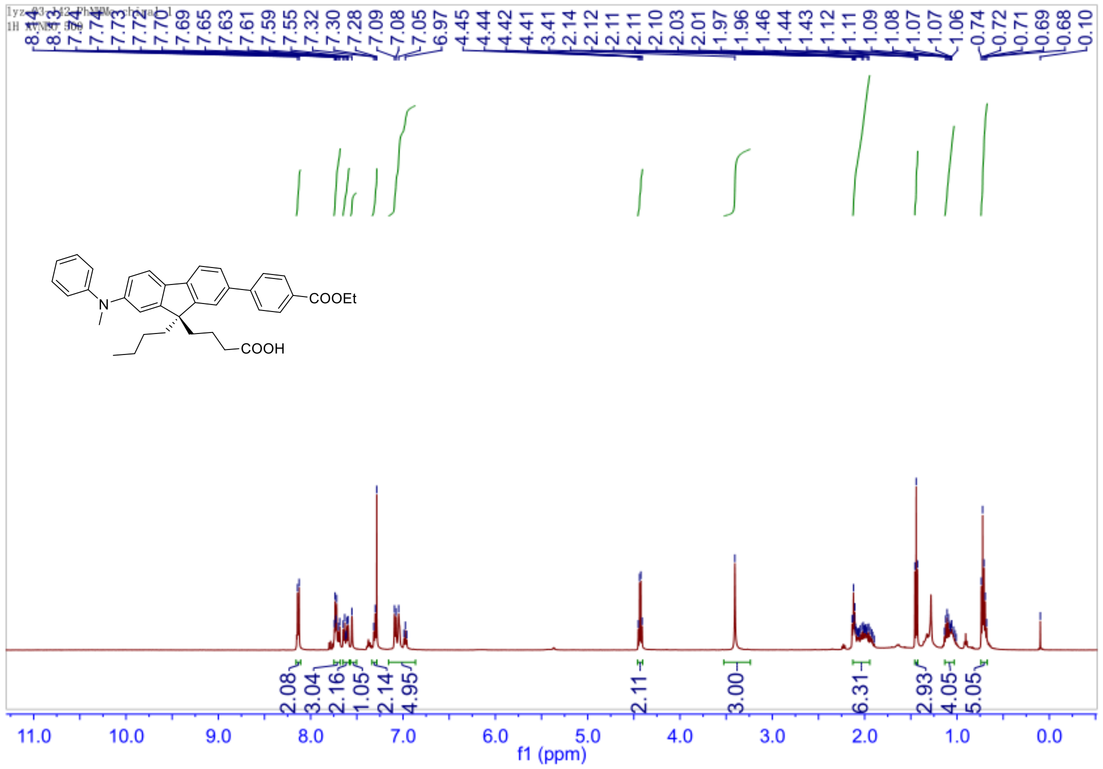

^{13}C NMR (126 MHz, $CDCl_3$)-**56**

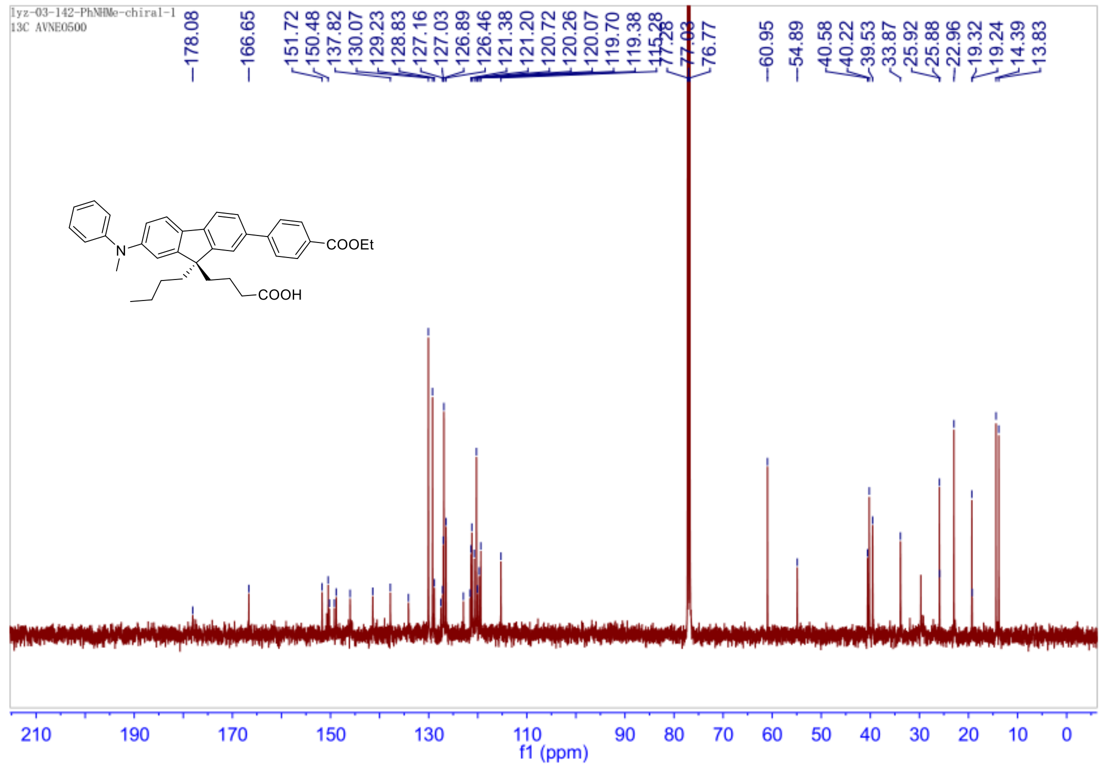

^{1}H NMR (400 MHz, $CDCl_3$)-precursor to **60**

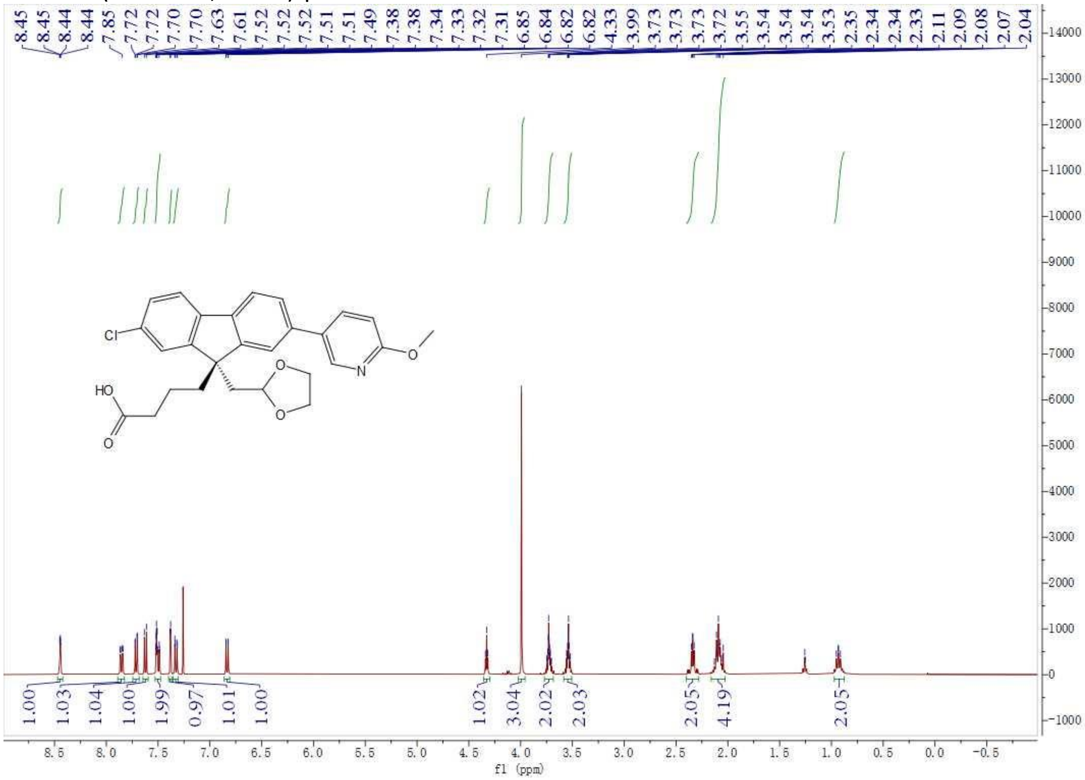

^{13}C NMR (101 MHz, $CDCl_3$)

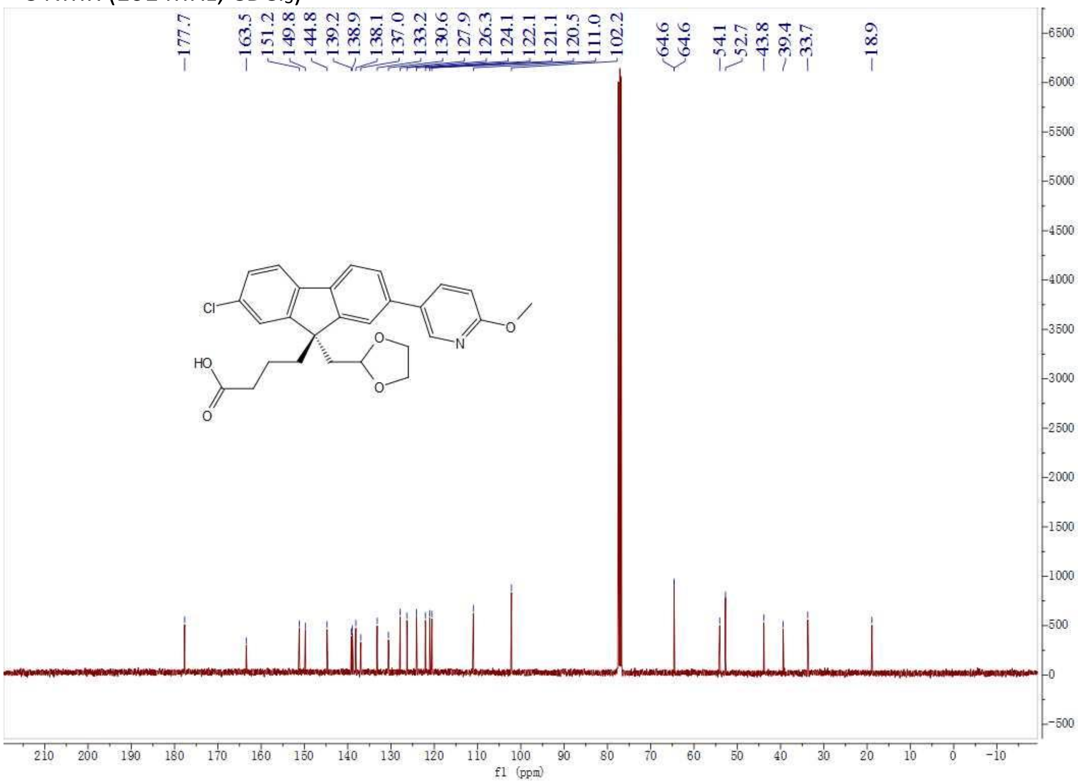

^{1}H NMR (400 MHz, $CDCl_3$)-**60**

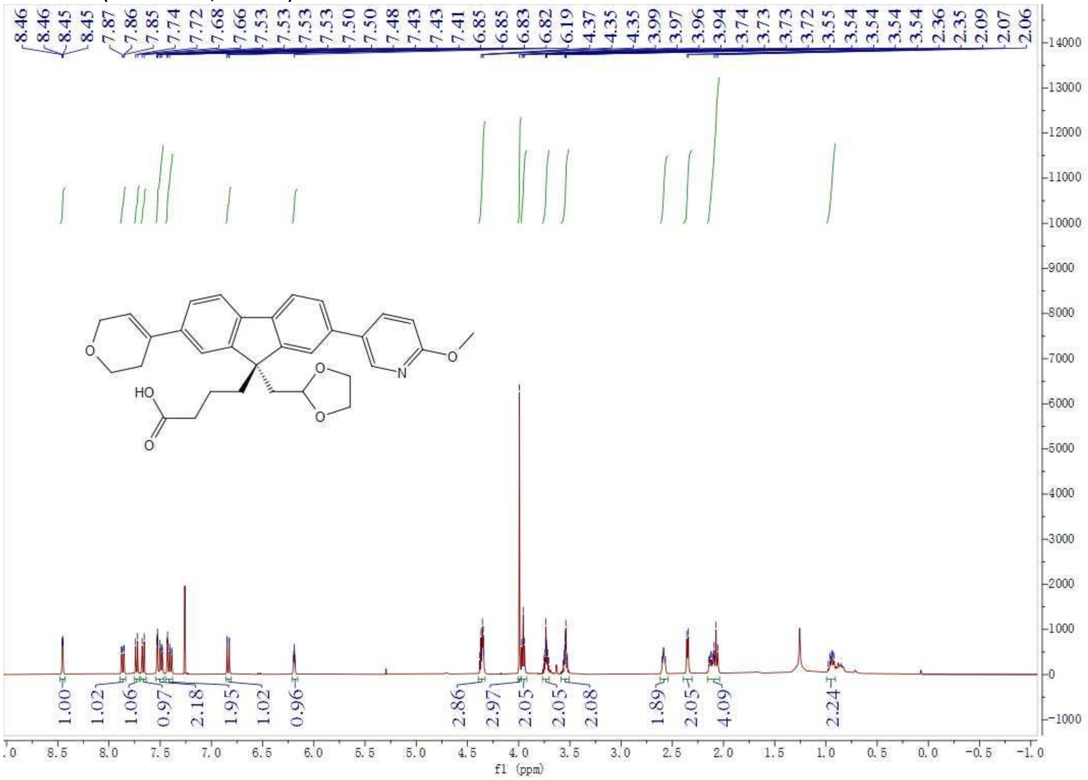

^{13}C NMR (101 MHz, $CDCl_3$)-**60**

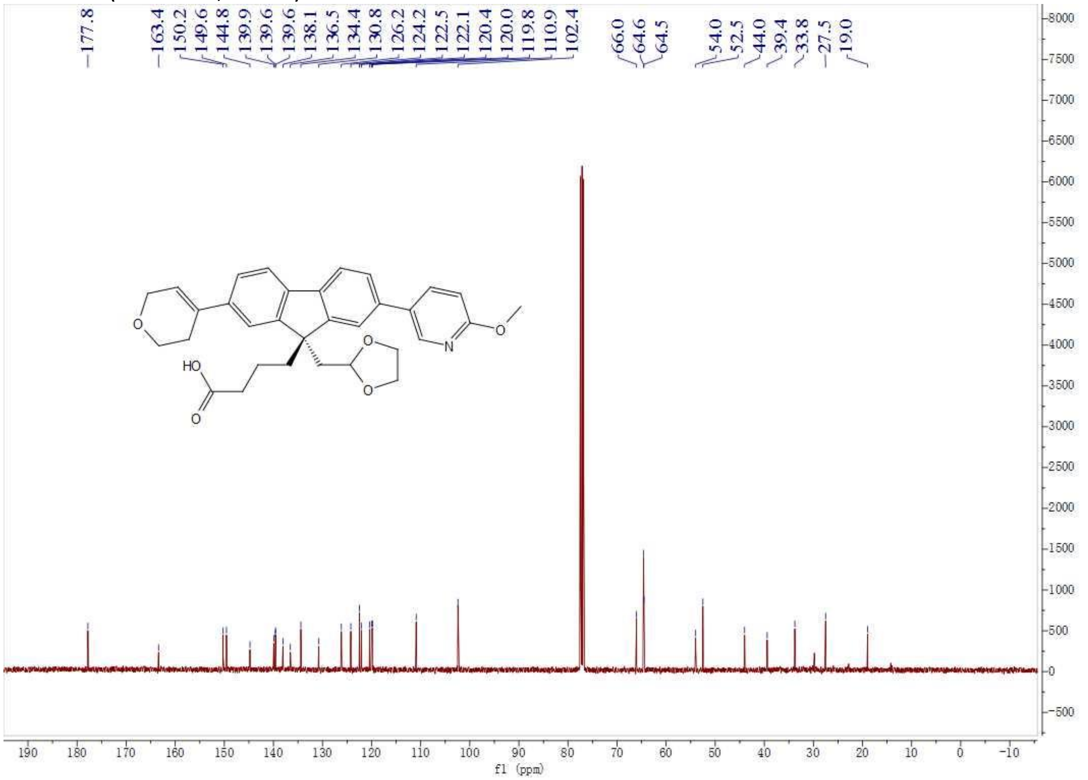

^{1}H NMR (500 MHz, $CDCl_3$)-precursor to ***ent*-60**

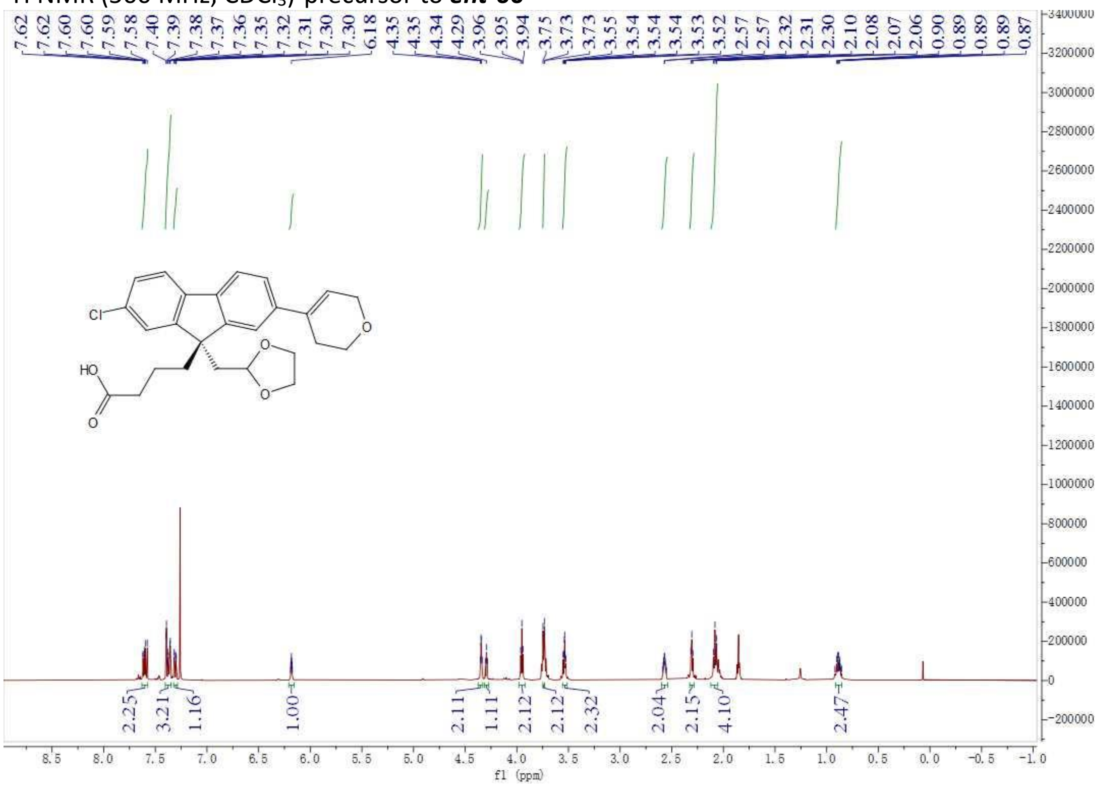

^{13}C NMR (126 MHz, $CDCl_3$)

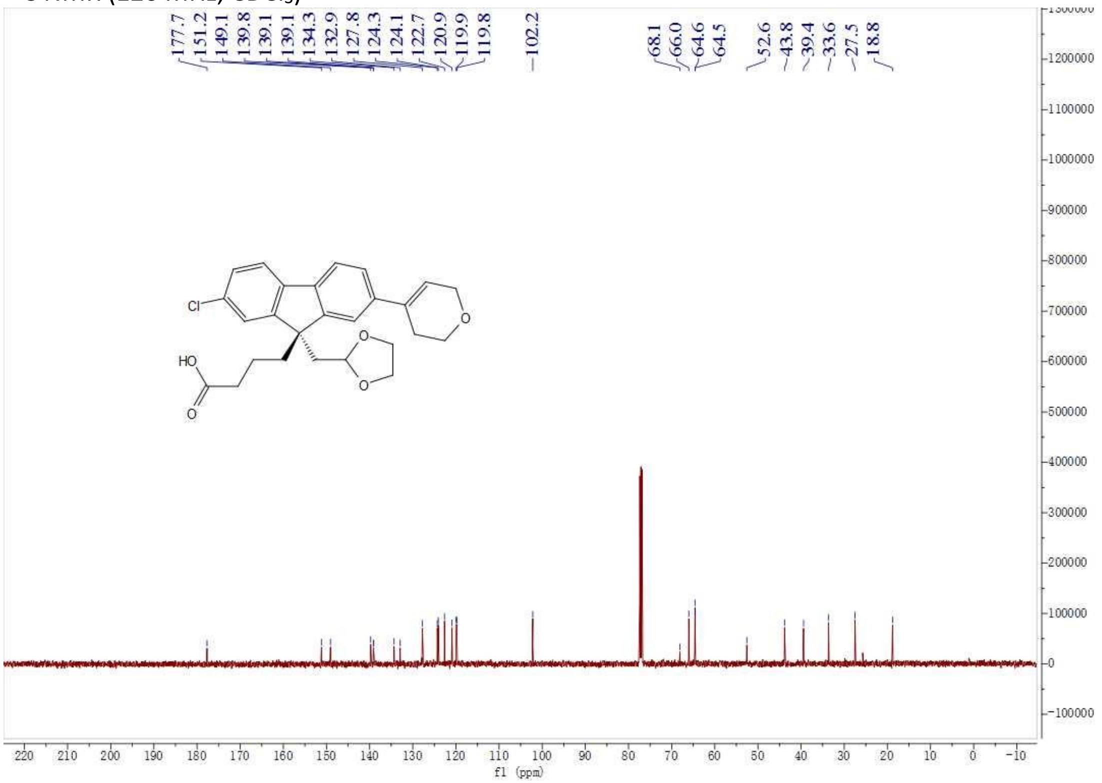

^1H NMR (400 MHz, $CDCl_3$)-**61**

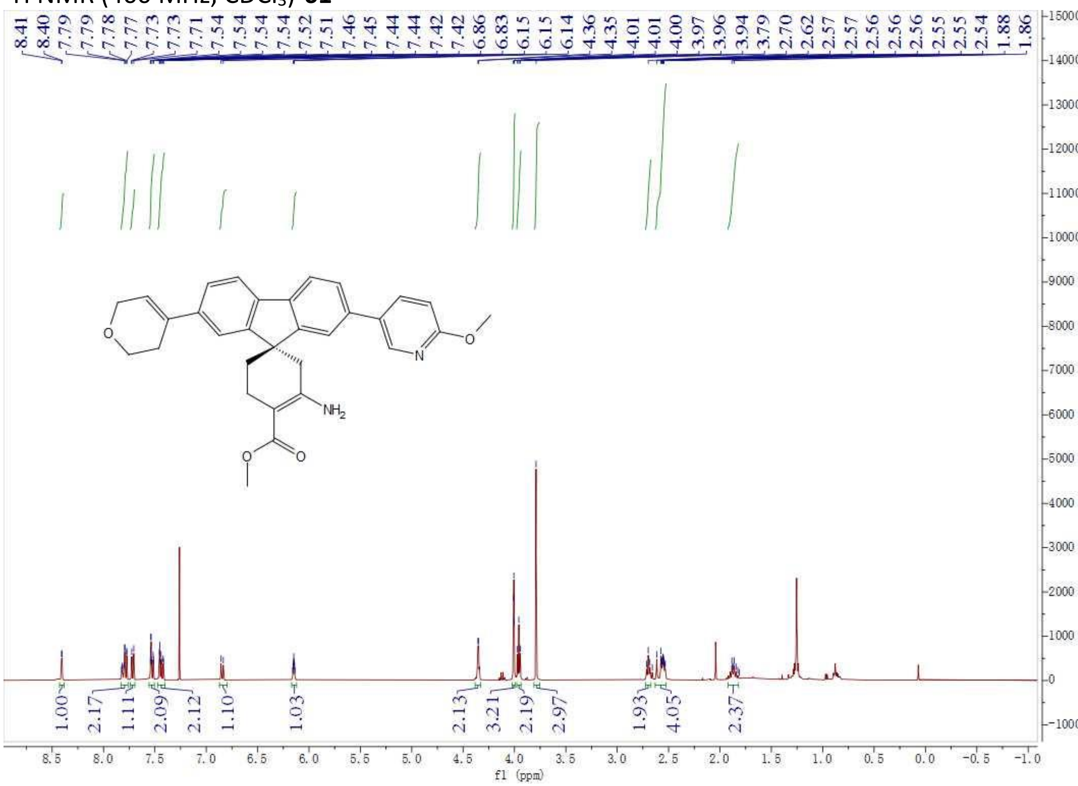

^{13}C NMR (101 MHz, $CDCl_3$)-**61**

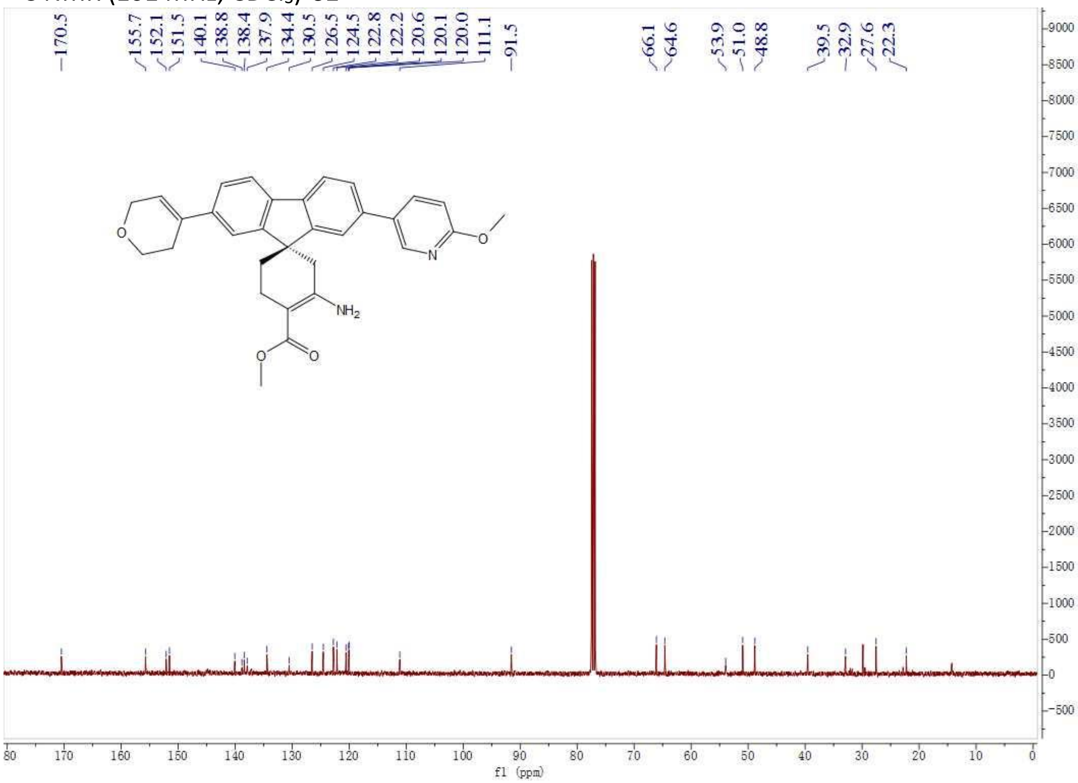

^{1}H NMR (500 MHz, $CDCl_3$)-**62**

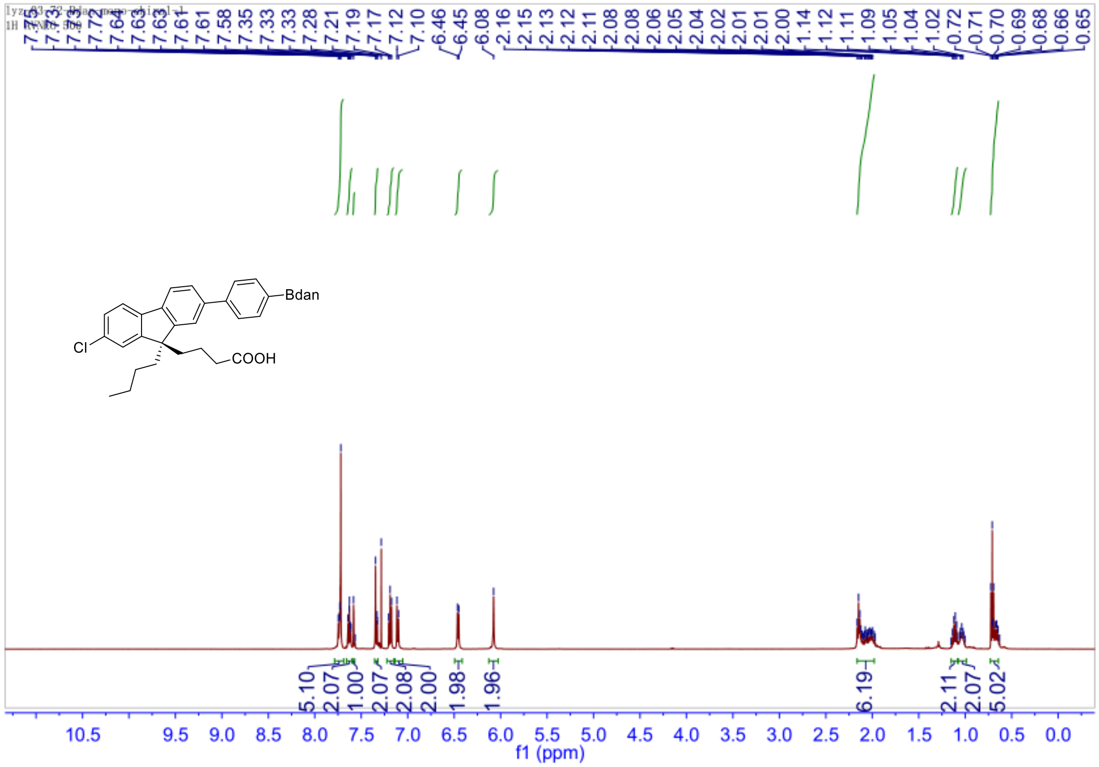

^{13}C NMR (126 MHz, $CDCl_3$)-**62**

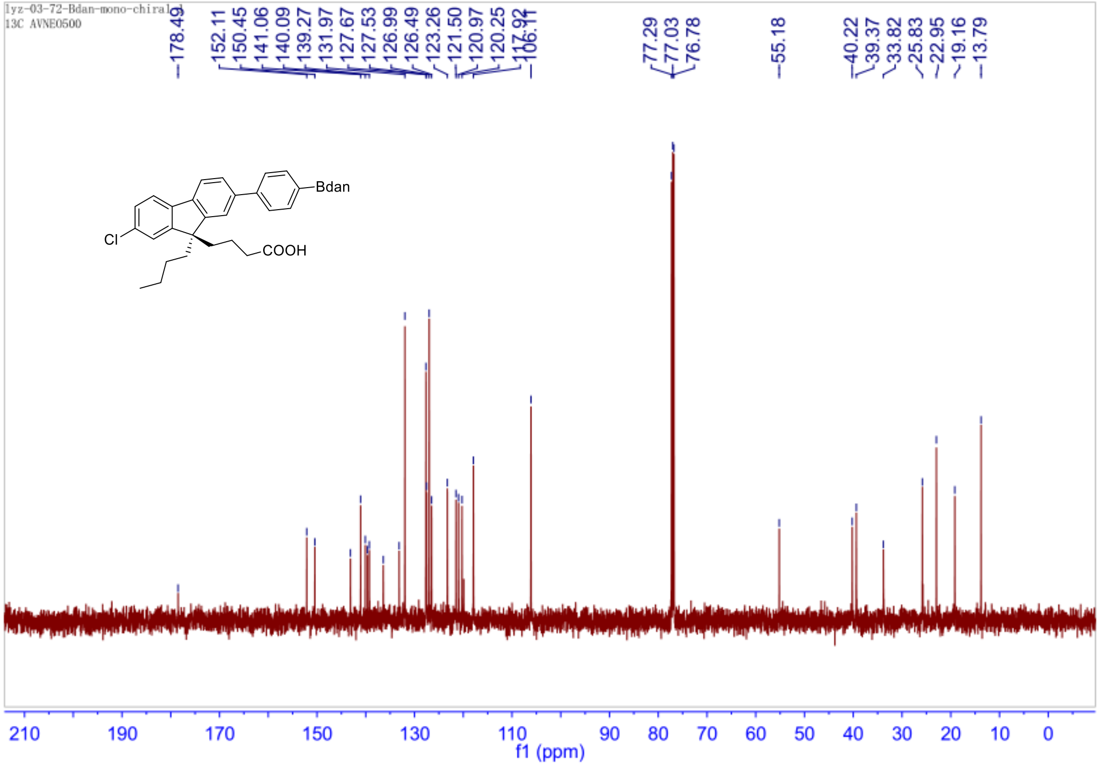

^{1}H NMR (500 MHz, d_6-Acetone) **-63**

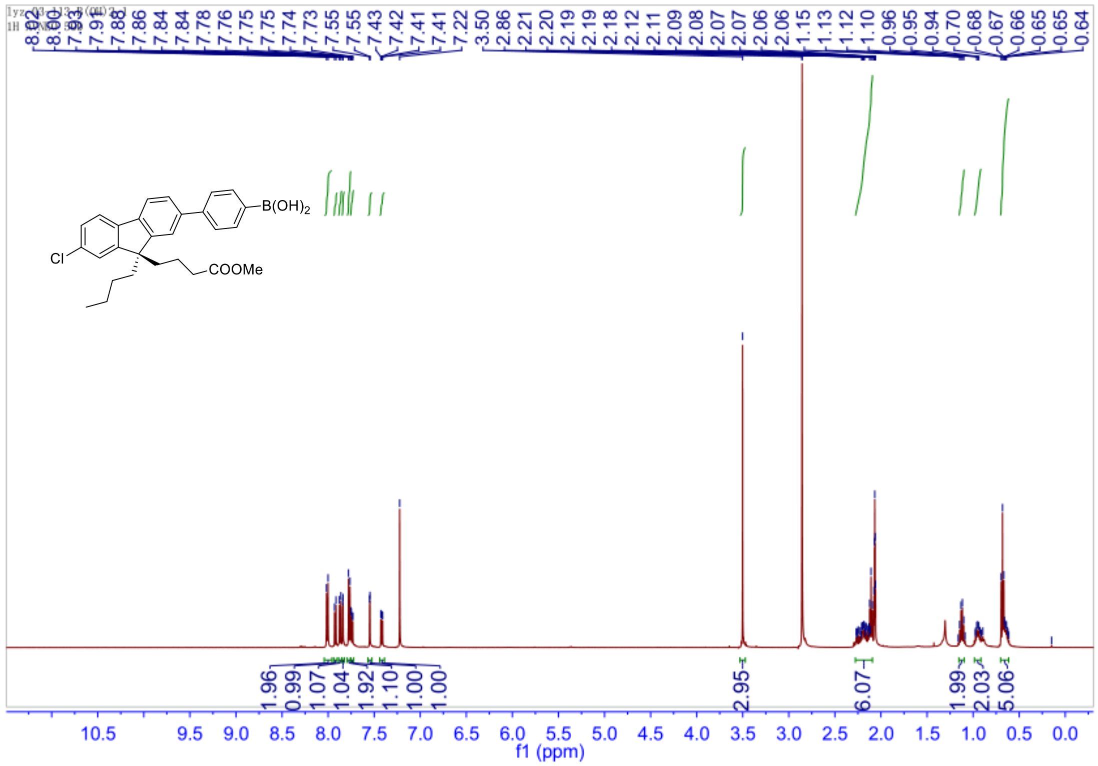

^{13}C NMR (126 MHz, d_6-Acetone) **-63**

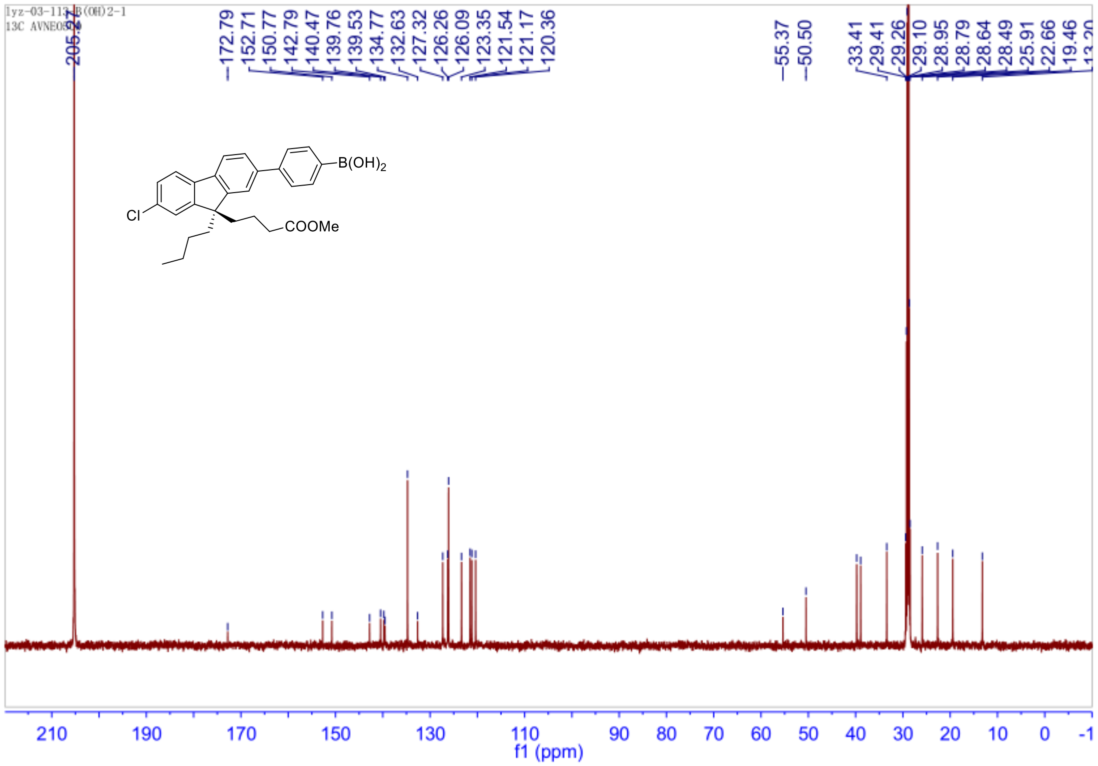

^{1}H NMR (500 MHz, $CDCl_3$)-**64**

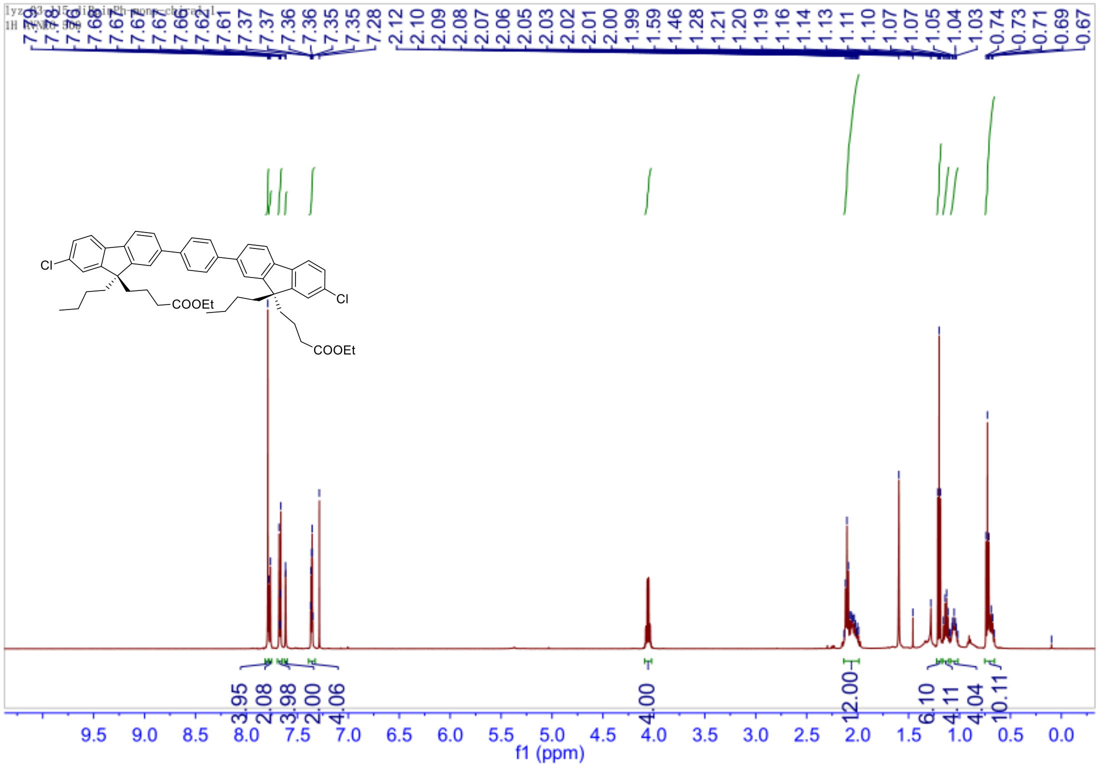

^{13}C NMR (126 MHz, $CDCl_3$)-**64**

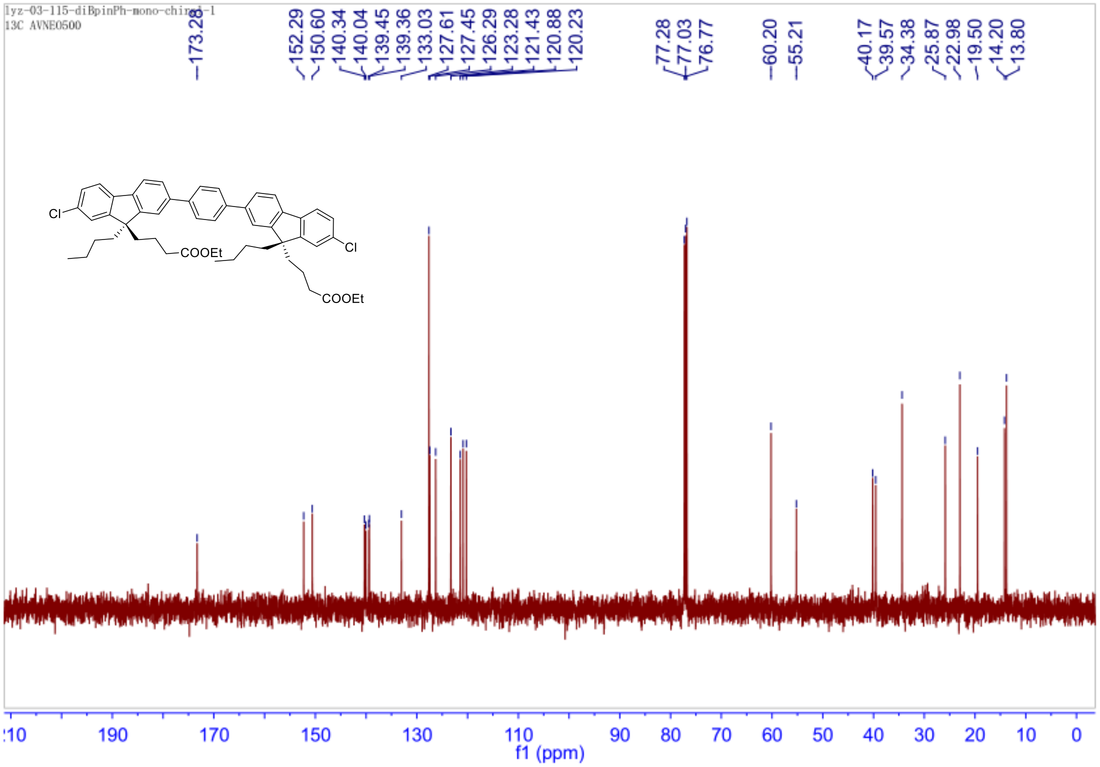

^{1}H NMR (500 MHz, $CDCl_3$)-*meso*-**64**

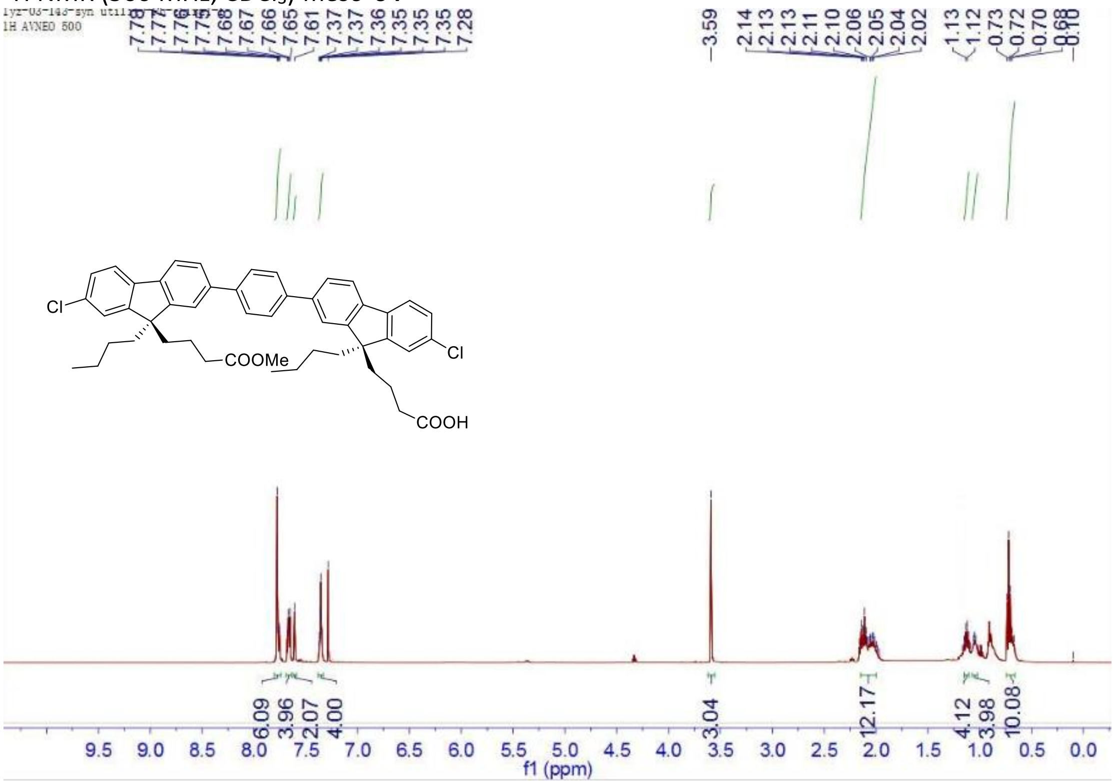

^{13}C NMR (126 MHz, $CDCl_3$) -*meso*-**64**

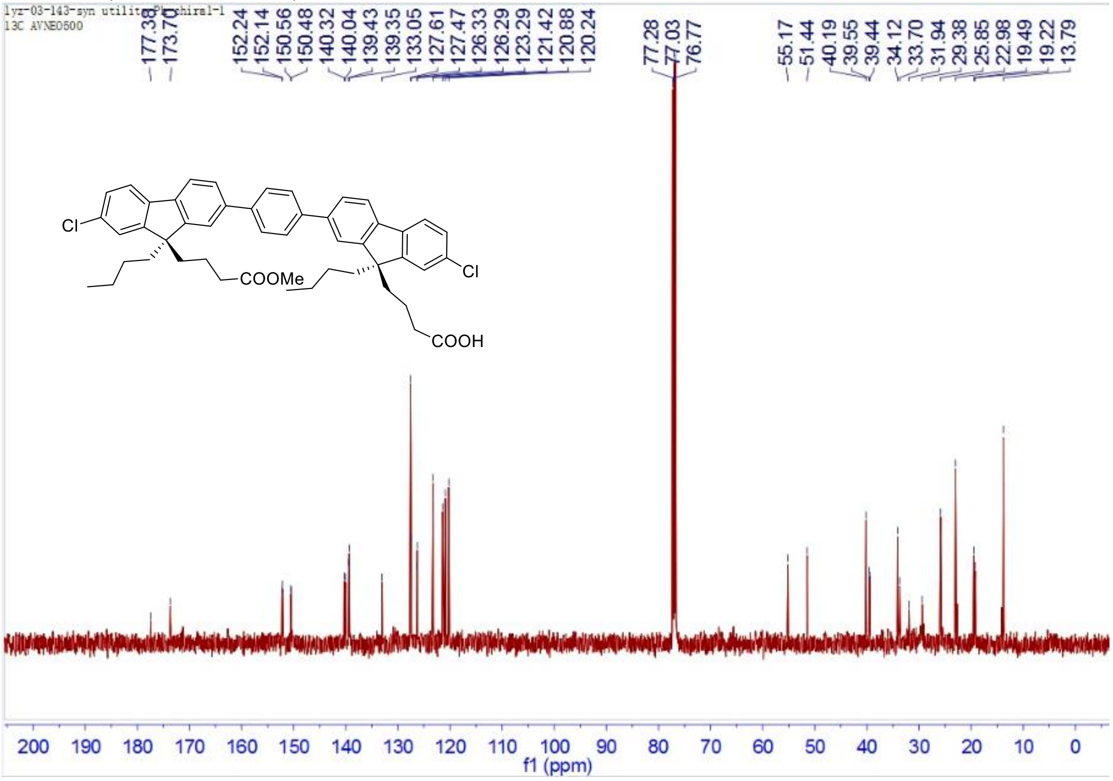

^{1}H NMR (400 MHz, $CDCl_3$)-precursor to **65**

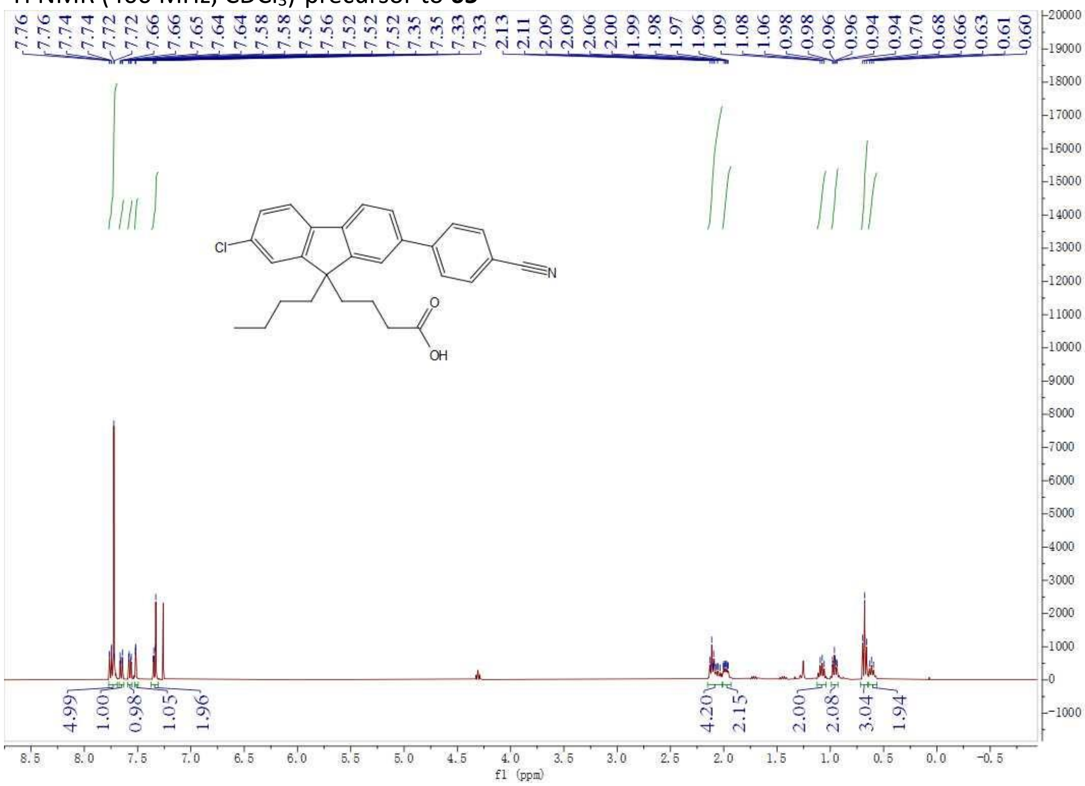

^{13}C NMR (101 MHz, $CDCl_3$)

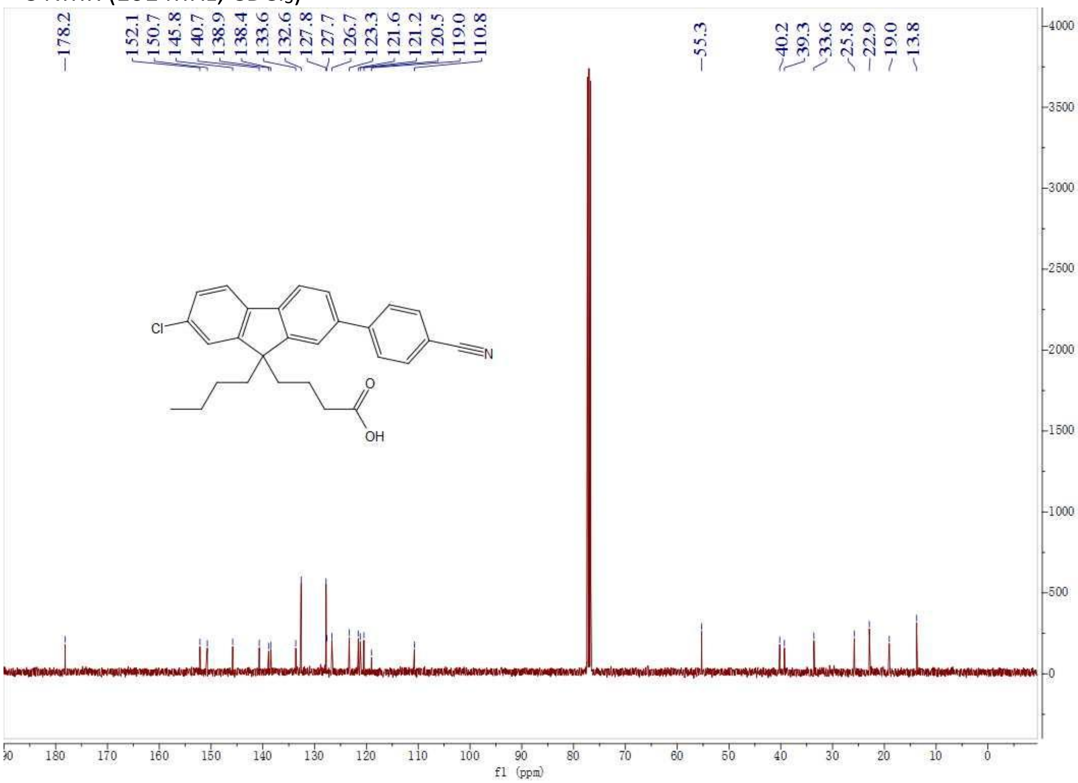

^{1}H NMR (400 MHz, $CDCl_3$)-precursor to **65**

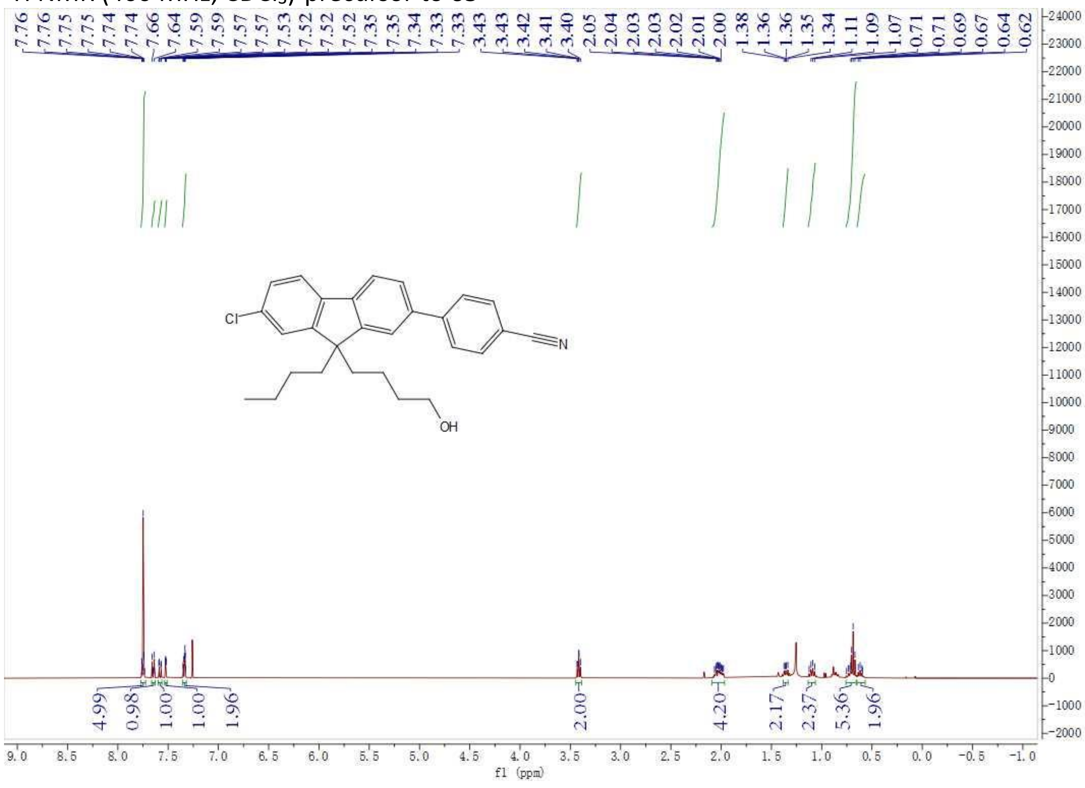

^{13}C NMR (126 MHz, $CDCl_3$)

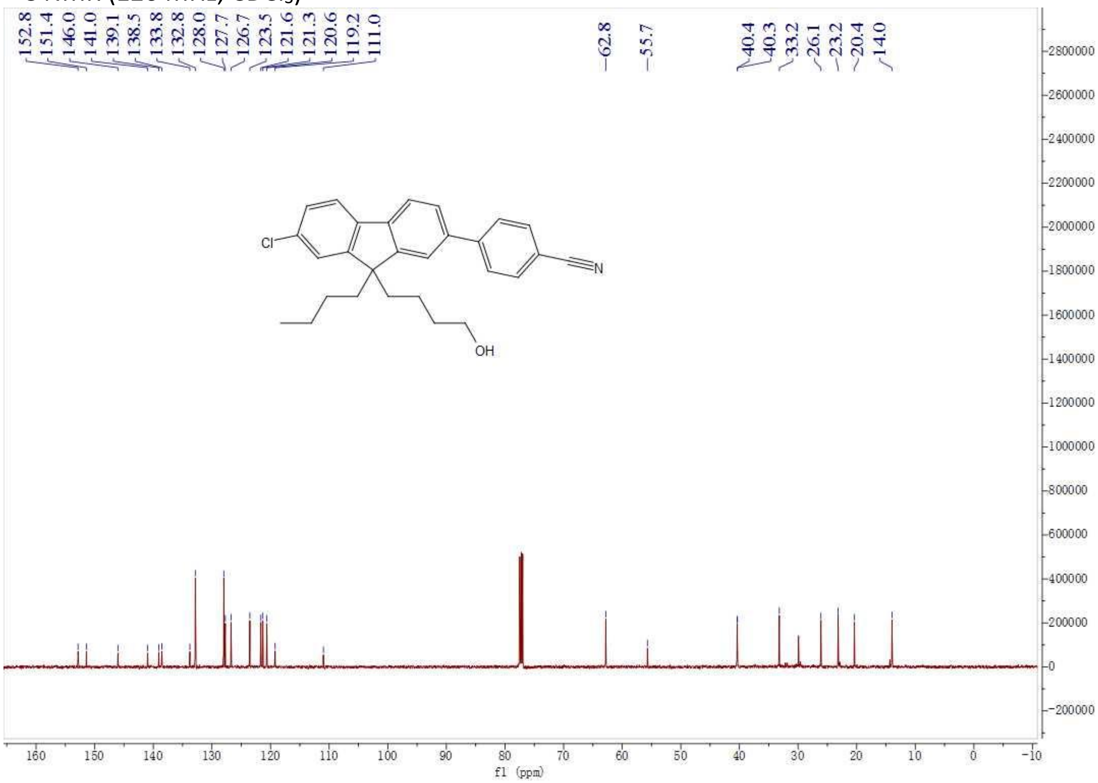

^{1}H NMR (400 MHz, $CDCl_3$)-**65**

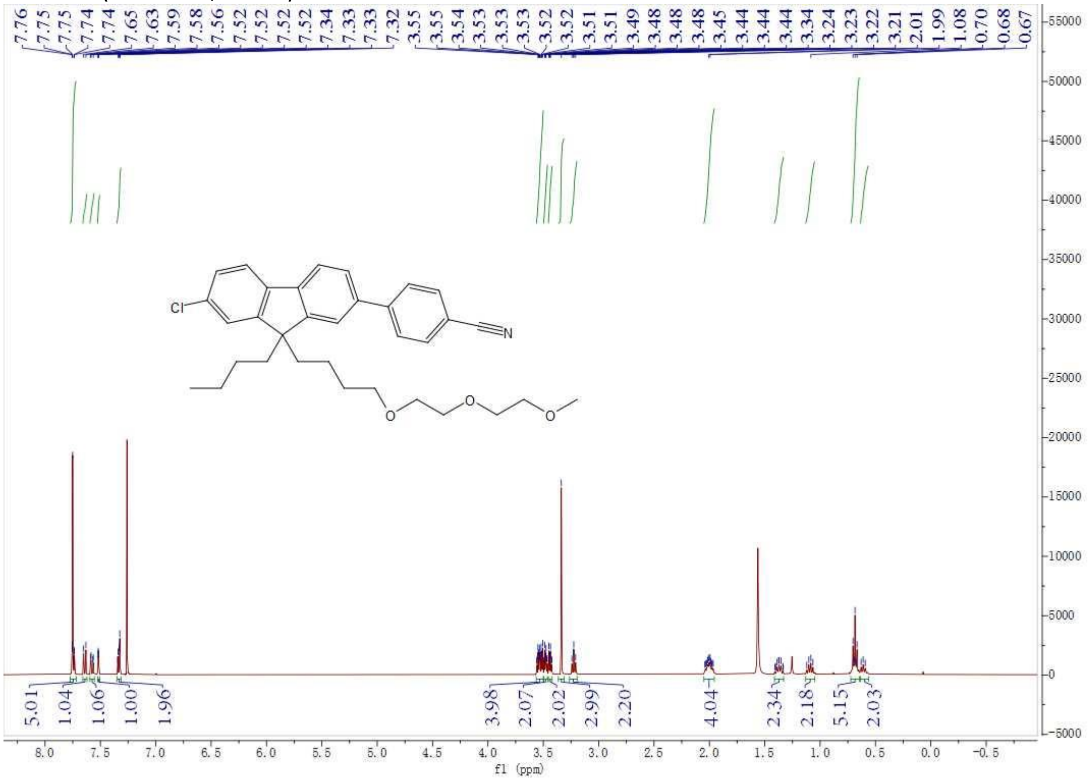

^{13}C NMR (126 MHz, $CDCl_3$)-**65**

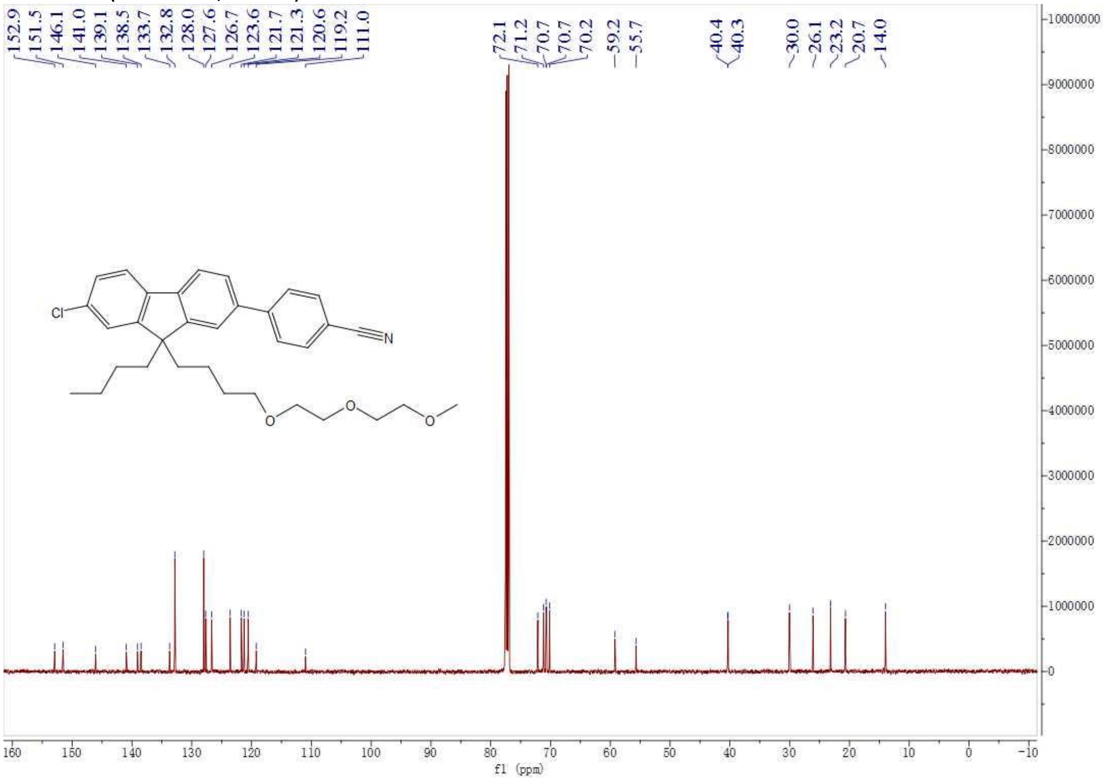

HPLC traces

L1

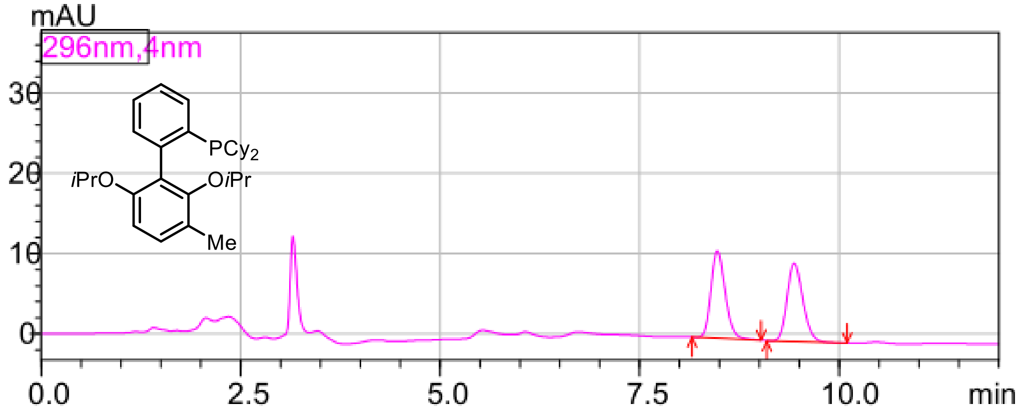

<Peak Table>

PDA Ch1 296nm

Peak#	Ret. Time	Area	Height	Mark	Area%	Peak Start	Peak End
1	8.474	134690	10903	M	50.241	8.155	9.024
2	9.438	133396	9728	M	49.759	9.093	10.101
Total		268086	20631		100.000		

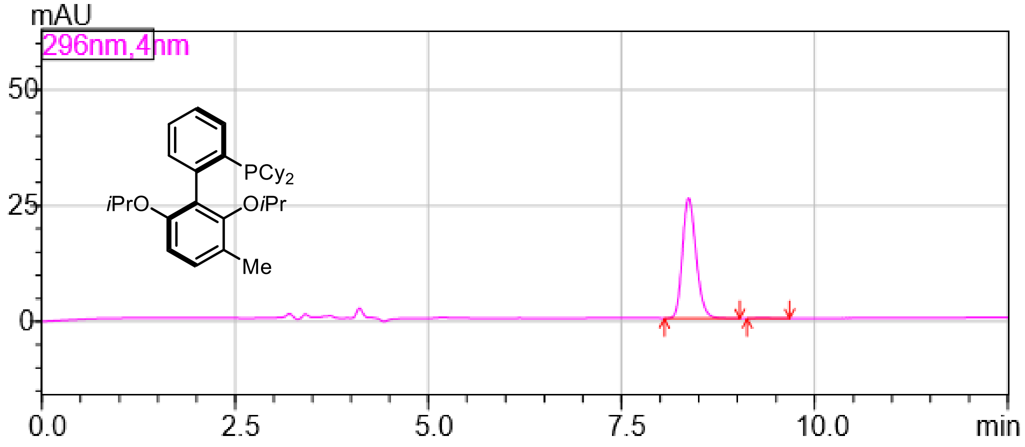

<Peak Table>

PDA Ch1 296nm

Peak#	Ret. Time	Area	Height	Mark	Area%	Peak Start	Peak End
1	8.366	320654	25988	M	99.752	8.053	9.029
2	9.348	797	65	M	0.248	9.125	9.675
Total		321451	26053		100.000		

SI-L3

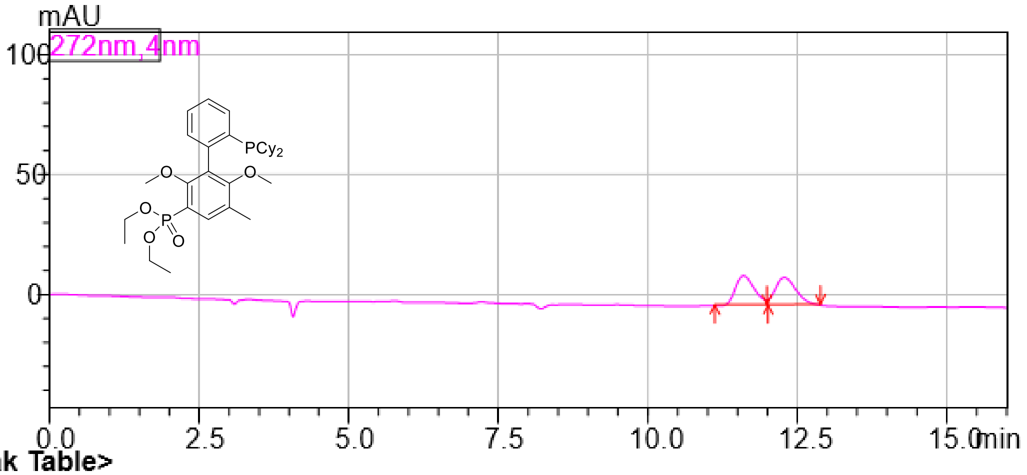

<Peak Table>

PDA Ch1 272nm

Peak#	Ret. Time	Area	Height	Mark	Area%	Peak Start	Peak End
1	11.601	261166	12079	M	49.549	11.120	11.995
2	12.282	265925	11362	M	50.451	12.011	12.885
Total		527091	23440		100.000		

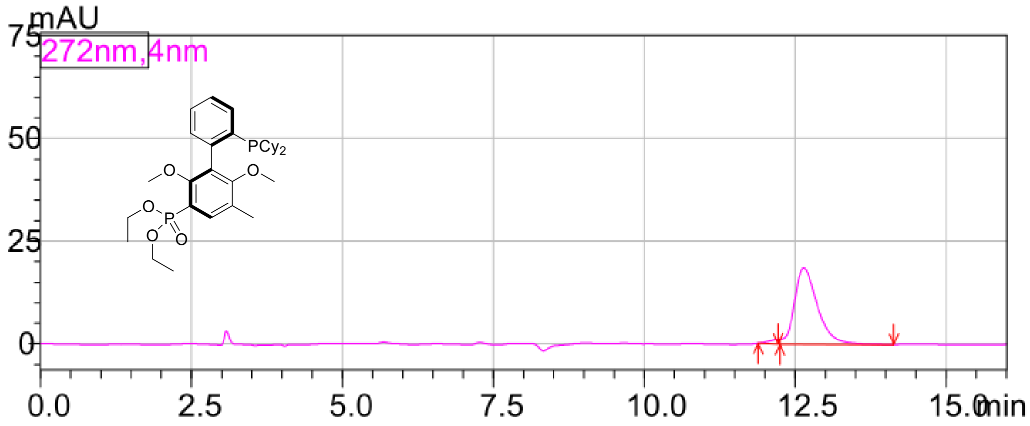

<Peak Table>

PDA Ch1 272nm

Peak#	Ret. Time	Area	Height	Mark	Area%	Peak Start	Peak End
1	11.885	8739	1195	M	1.392	11.685	11.888
2	12.265	619240	24283	M	98.608	11.931	13.696
Total		627979	25477		100.000		

1 (Table 1)

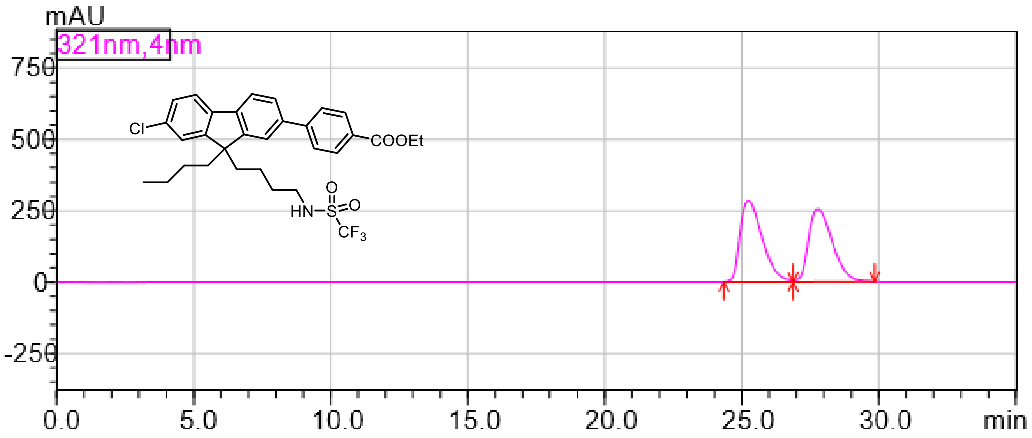

<Peak Table>

PDA Ch1 321nm

Peak#	Ret. Time	Area	Peak Start	Peak End	Height	Area%
1	25.242	16069281	24.347	26.875	283170	51.049
2	27.774	15409018	26.875	29.856	255538	48.951
Total		31478299			538707	100.000

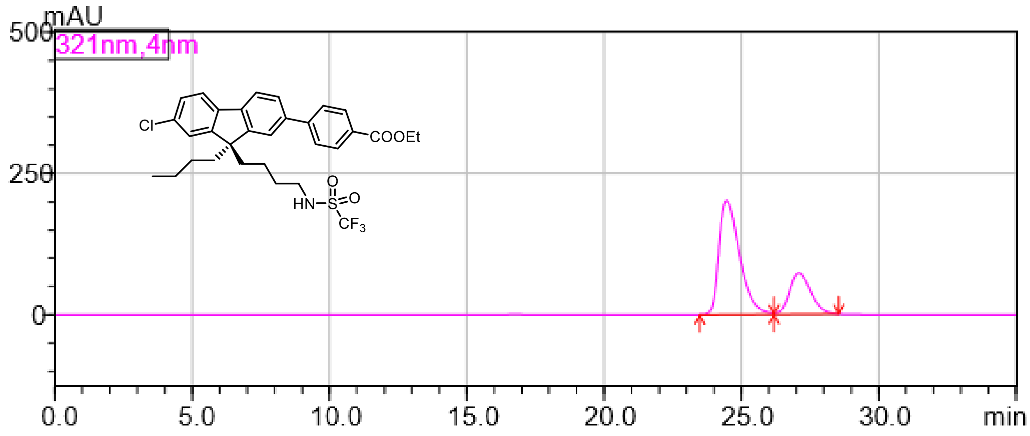

<Peak Table>

PDA Ch1 321nm

Peak#	Ret. Time	Area	Height	Peak Start	Peak End	Area%
1	24.466	10527717	201750	23.477	26.187	72.943
2	27.093	3905045	72832	26.187	28.549	27.057
Total		14432761	274582			100.000

4 (Table 1)

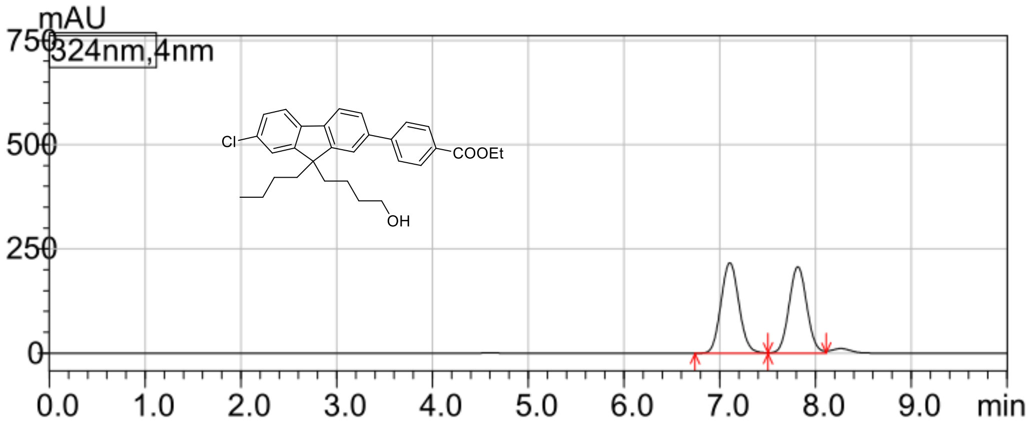

<Peak Table>

PDA Ch1 325nm

Peak#	Ret. Time	Area	Height	Mark	Area%	Peak Start	Peak End
1	7.105	2764300	213258	M	50.687	6.757	7.461
2	7.815	2689339	205076	M	49.313	7.541	8.181
Total		5453638	418334		100.000		

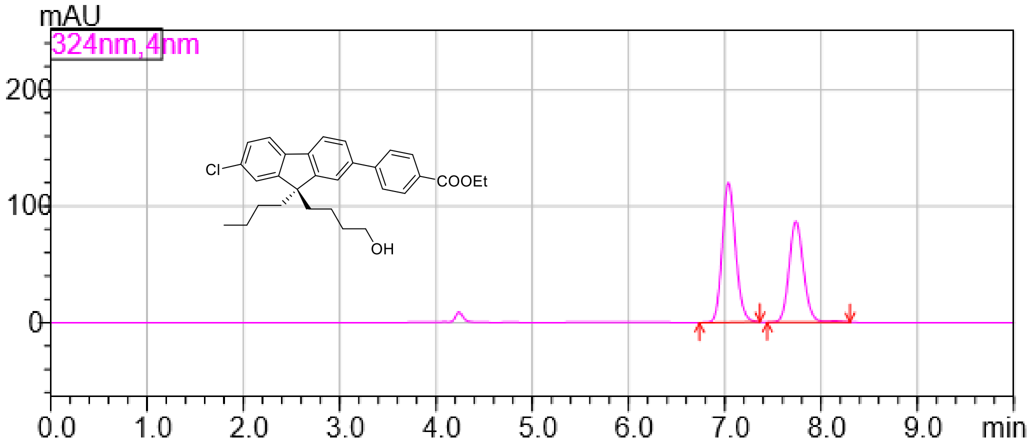

<Peak Table>

PDA Ch1 324nm

Peak#	Ret. Time	Area	Height	Mark	Area%	Peak Start	Peak End
1	7.038	1158012	119562	M	55.985	6.736	7.365
2	7.738	910414	86520	M	44.015	7.440	8.299
Total		2068426	206082		100.000		

5 (Table 1)

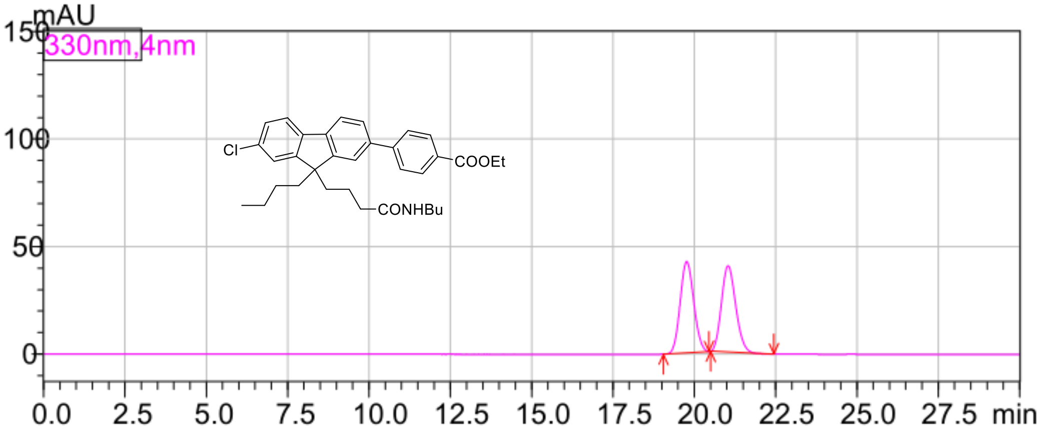

<Peak Table>

PDA Ch1 330nm

Peak#	Ret. Time	Area	Height	Mark	Area%	Peak Start	Peak End
1	19.765	1240436	42511	M	50.308	19.045	20.448
2	21.037	1225238	40109	M	49.692	20.507	22.443
Total		2465673	82620		100.000		

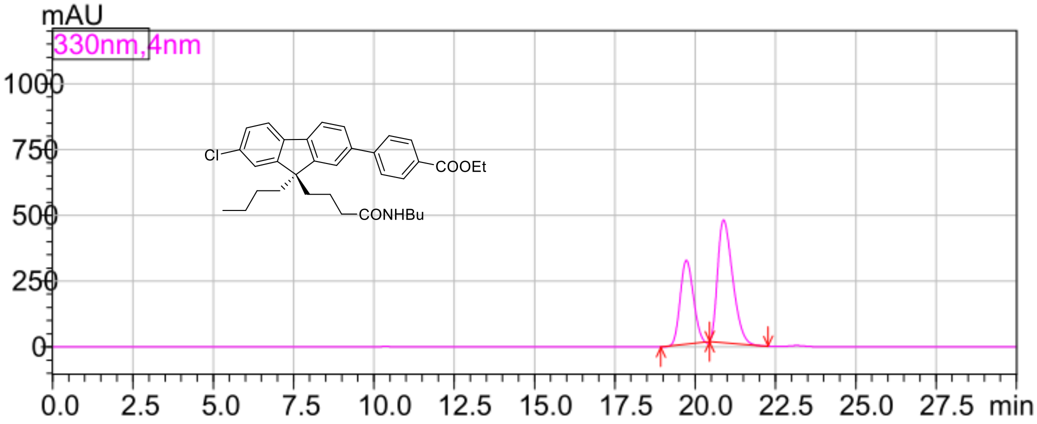

<Peak Table>

PDA Ch1 330nm

Peak#	Ret. Time	Area	Height	Mark	Area%	Peak Start	Peak End
1	19.727	8982205	318138	M	37.279	18.928	20.448
2	20.885	15112260	465848	M	62.721	20.448	22.267
Total		24094465	783986		100.000		

6 (Table 1)

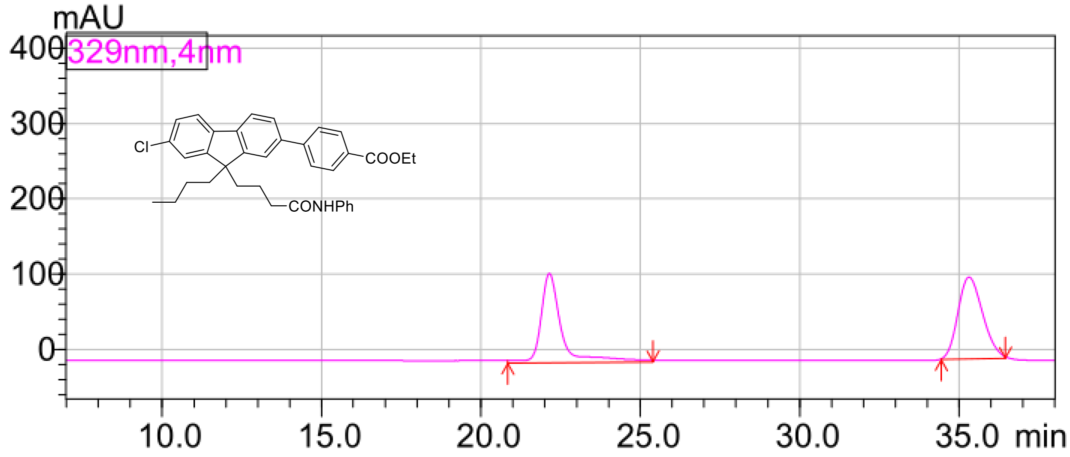

<Peak Table>

PDA Ch1 329nm

Peak#	Ret. Time	Area	Height	Area%	Peak Start	Peak End
1	22.142	5394166	118544	48.033	20.843	25.397
2	35.316	5835976	108458	51.967	34.443	36.453
Total		11230142	227002	100.000		

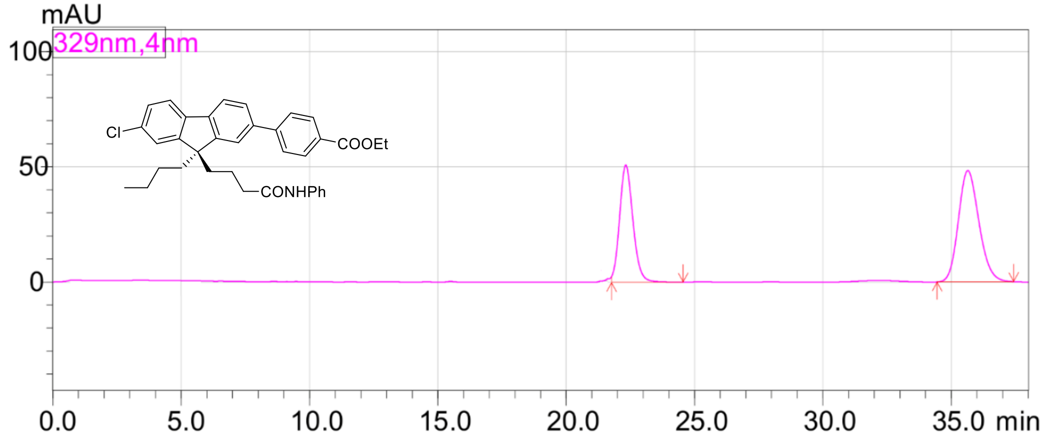

<Peak Table>

PDA Ch1 329nm

Peak#	Ret. Time	Area	Height	Area%	Peak Start	Peak End
1	22.320	1832972	50802	40.480	21.765	24.549
2	35.646	2695129	48339	59.520	34.453	37.429
Total		4528101	99141	100.000		

Substrate scope (Table 2 and Scheme 3)

7

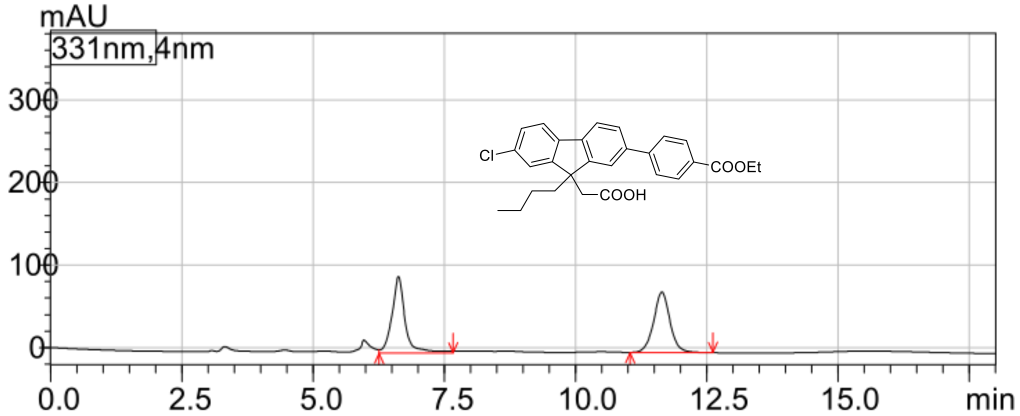

<Peak Table>

PDA Ch1 331nm

Peak#	Ret. Time	Area	Height	Mark	Area%	Peak Start	Peak End
1	6.626	1540227	91526	M	50.248	6.293	7.595
2	11.644	1525042	72447	M	49.752	11.147	12.165
Total		3065269	163972		100.000		

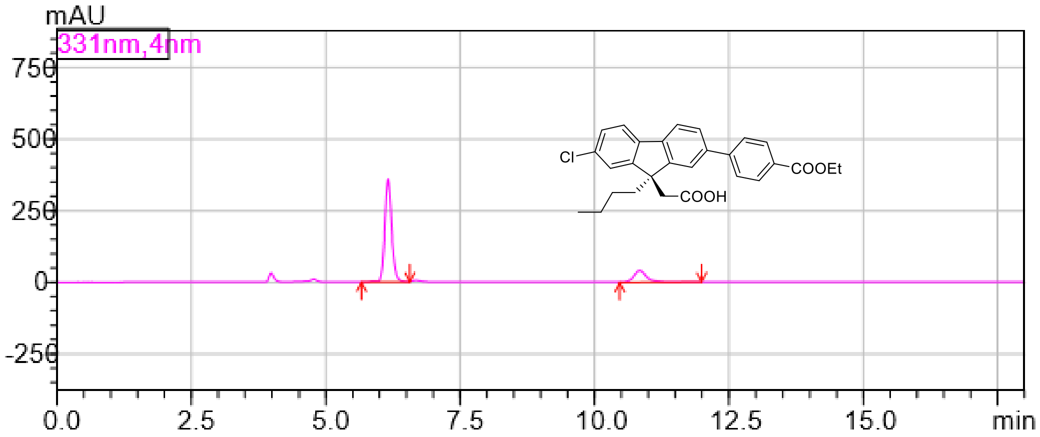

<Peak Table>

PDA Ch1 331nm

Peak#	Ret. Time	Area	Height	Mark	Area%	Peak Start	Peak End
1	6.157	3261362	359661	M	82.623	5.664	6.565
2	10.840	685925	40779	M	17.377	10.469	11.995
Total		3947287	400441		100.000		

8

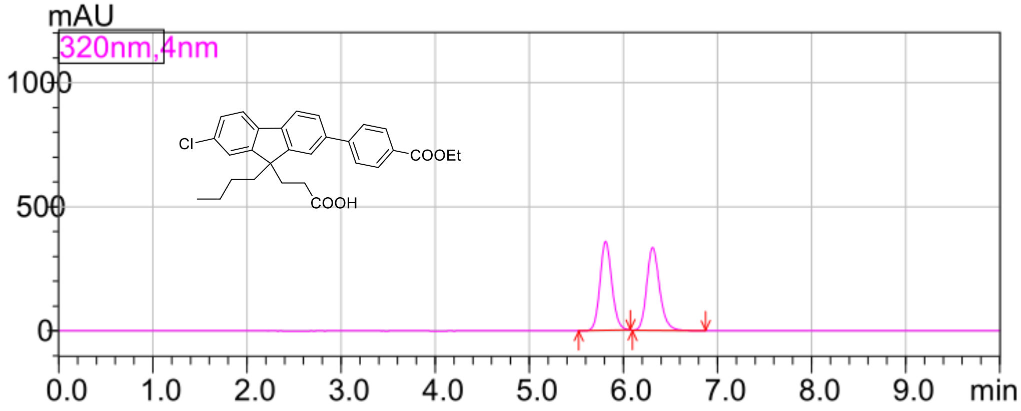

<Peak Table>

PDA Ch1 320nm

Peak#	Ret. Time	Area	Height	Mark	Area%	Peak Start	Peak End
1	5.811	3148595	358360	M	49.862	5.525	6.075
2	6.310	3166084	333331	M	50.138	6.096	6.875
Total		6314679	691691		100.000		

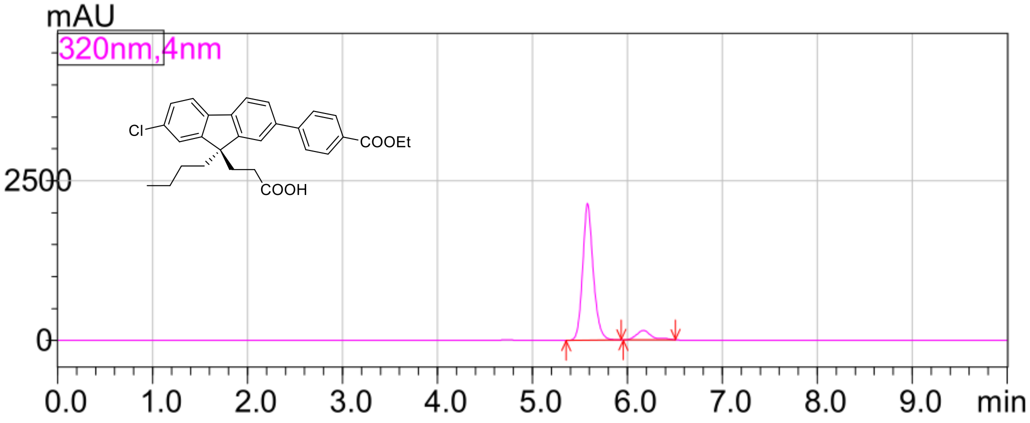

<Peak Table>

PDA Ch1 320nm

Peak#	Ret. Time	Area	Height	Mark	Area%	Peak Start	Peak End
1	5.580	16589232	2145980	M	90.595	5.355	5.936
2	6.169	1722235	150925	M	9.405	5.957	6.507
Total		18311467	2296905		100.000		

3

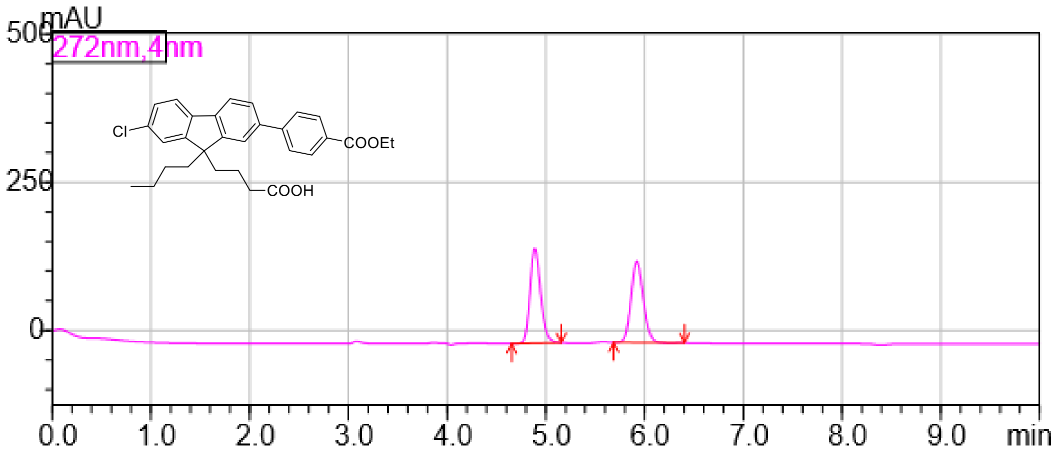

<Peak Table>

PDA Ch1 272nm

Peak#	Ret. Time	Area	Height	Mark	Area%	Peak Start	Peak End
1	4.889	1195937	160467	M	50.337	4.651	5.157
2	5.921	1179907	136881	M	49.663	5.685	6.405
Total		2375844	297348		100.000		

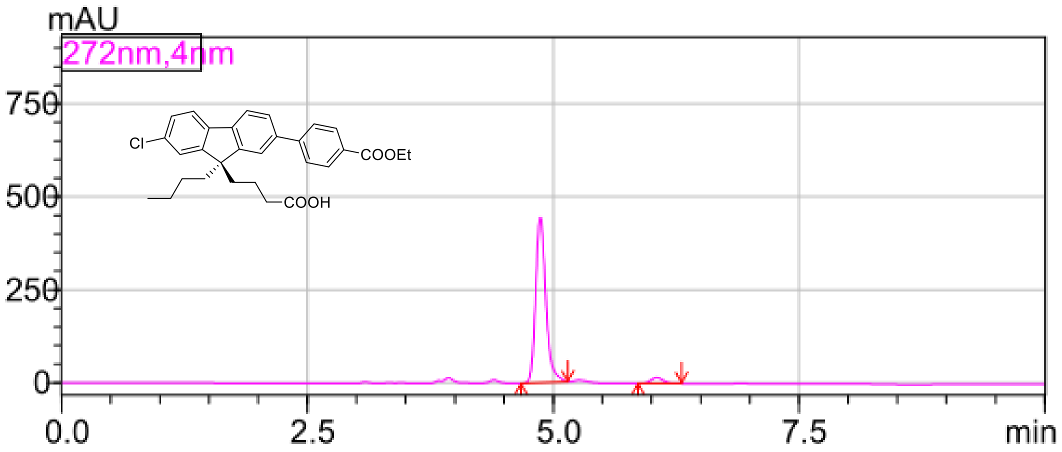

<Peak Table>

PDA Ch1 272nm

Peak#	Ret. Time	Area	Height	Area%	Peak Start	Peak End
1	4.868	3205662	444990	96.019	4.672	5.147
2	6.053	132915	15550	3.981	5.861	6.304
Total		3338577	460540	100.000		

9

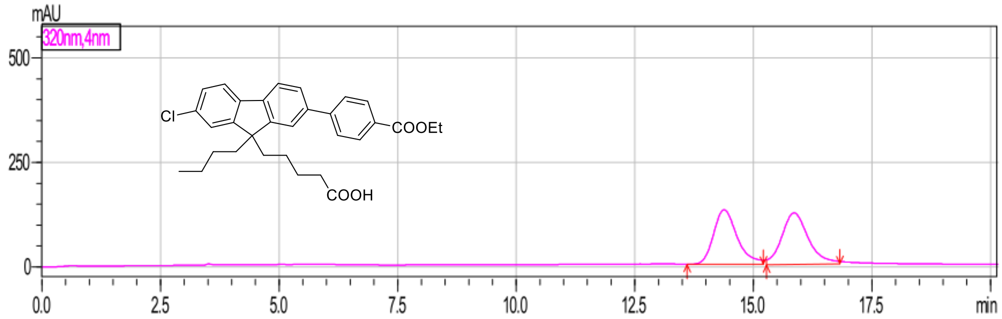

<Peak Table>

PDA Ch1 320nm

Peak#	Ret. Time	Area	Mark	Area%
1	14.382	4571832		48.735
2	15.857	4809141	M	51.265
Total		9380973		100.000

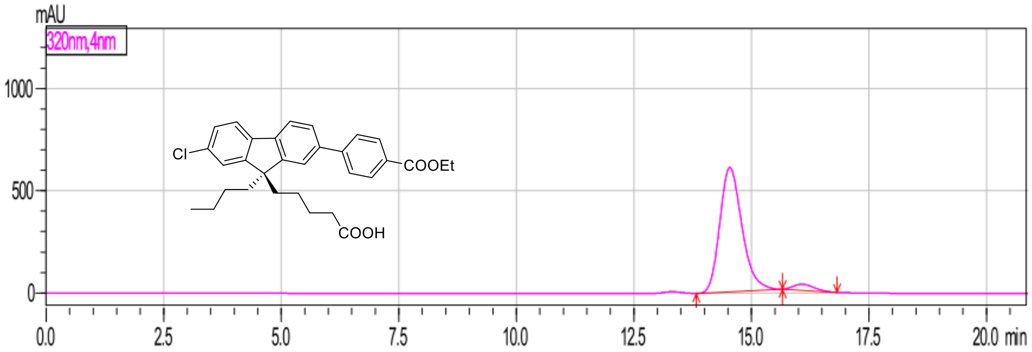

<Peak Table>

PDA Ch1 320nm

Peak#	Ret. Time	Area	Unit	Area%
1	14.540	19987622		95.620
2	16.074	915481		4.380
Total		20903103		100.000

10

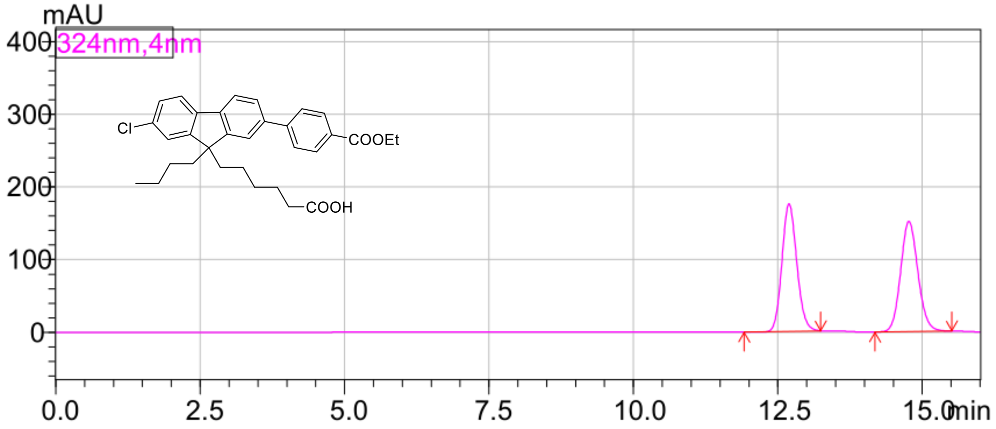

<Peak Table>

PDA Ch1 324nm

Peak#	Ret. Time	Area	Height	Area%	Peak Start	Peak End
1	12.693	3134531	175640	49.763	11.915	13.243
2	14.769	3164340	151761	50.237	14.181	15.509
Total		6298870	327401	100.000		

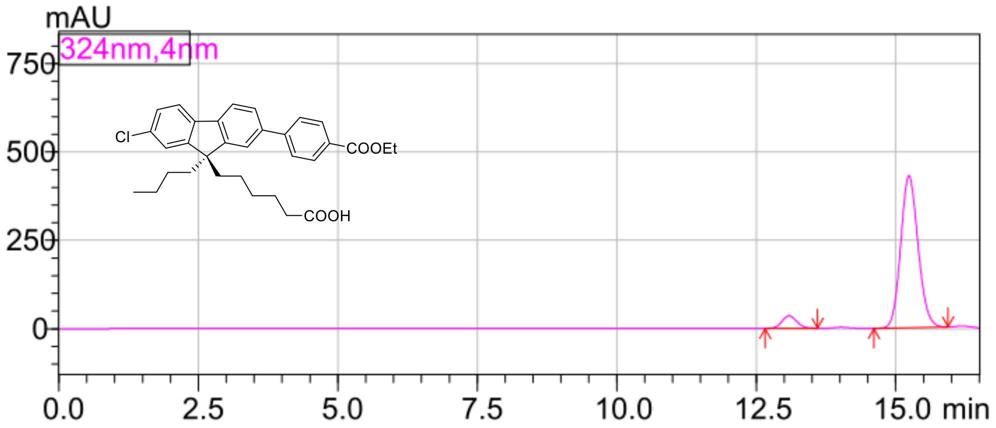

<Peak Table>

PDA Ch1 324nm

Peak#	Ret. Time	Area	Height	Area%	Peak Start	Peak End
1	13.086	664913	35466	6.645	12.656	13.595
2	15.236	9342030	430358	93.355	14.608	15.936
Total		10006943	465823	100.000		

11

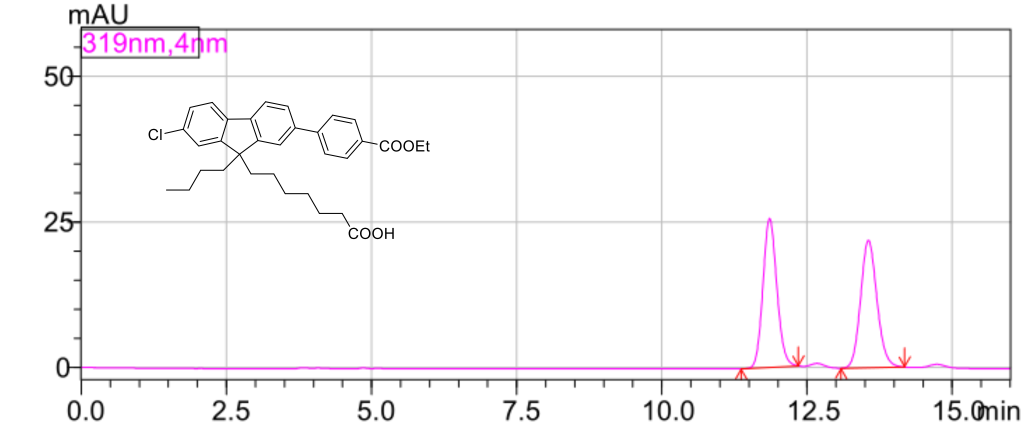

<Peak Table>

PDA Ch1 319nm

Peak#	Ret. Time	Height	Mark	Area%	Area/Height	Peak Start	Peak End
1	11.855	25567	M	49.793	16.704	11.365	12.347
2	13.558	21908	M	50.207	19.655	13.088	14.181
Total		47476		100.000			

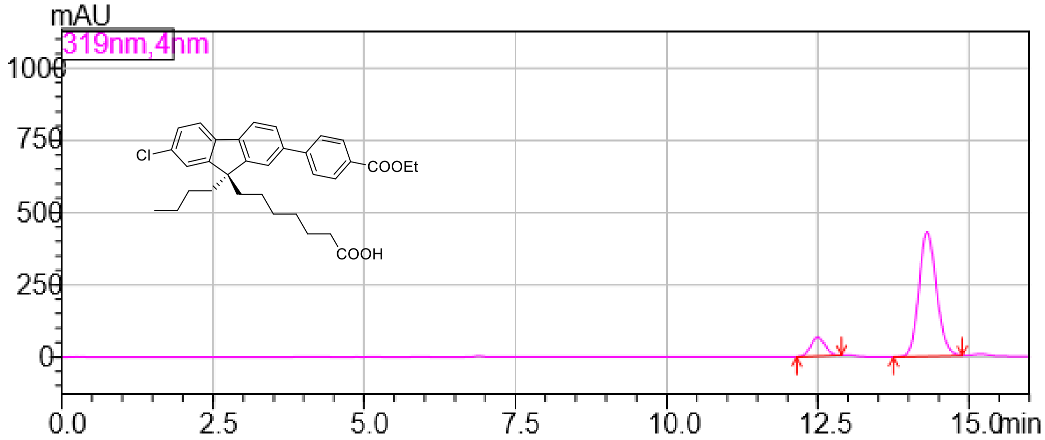

<Peak Table>

PDA Ch1 319nm

Peak#	Ret. Time	Area	Height	Area%	Peak Start	Peak End
1	12.501	1059047	64136	10.922	12.149	12.891
2	14.308	8637379	429041	89.078	13.749	14.885
Total		9696426	493176	100.000		

12

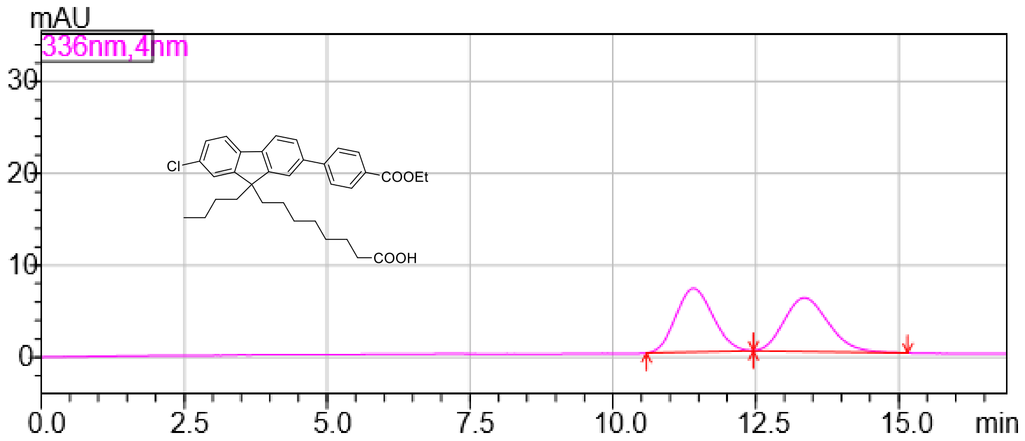

<Peak Table>

PDA Ch1 336nm

Peak#	Ret. Time	Area	Conc.	Area%	Height	Peak Start	Peak End
1	11.409	311432	0.000	49.856	6922	10.587	12.459
2	13.358	313228	0.000	50.144	5845	12.459	15.157
Total		624660		100.000	12768		

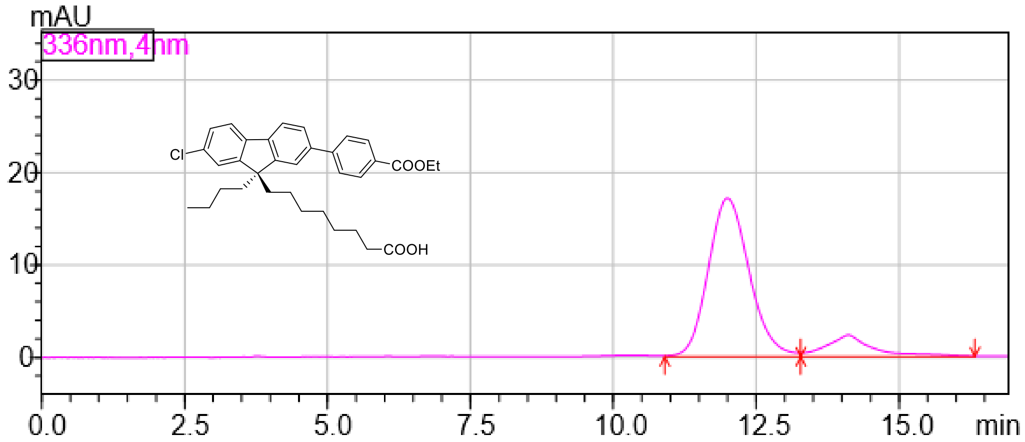

<Peak Table>

PDA Ch1 336nm

Peak#	Ret. Time	Unit	Mark	Area%	Height	Peak Start	Peak End
1	11.996		M	86.394	17179	10.896	13.280
2	14.115		M	13.606	2343	13.280	16.331
Total				100.000	19522		

13

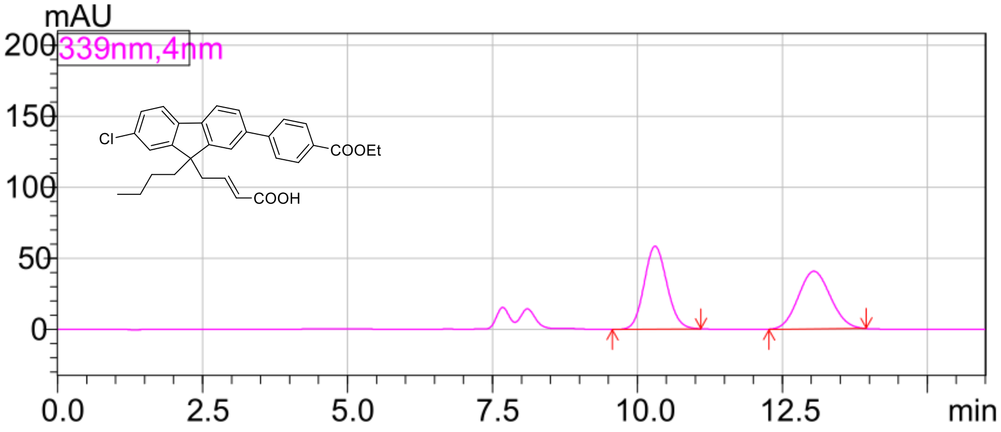

<Peak Table>

PDA Ch1 339nm

Peak#	Ret. Time	Area	Height	Area%	Peak Start	Peak End
1	10.304	1600515	58524	50.493	9.568	11.093
2	13.043	1569239	40609	49.507	12.267	13.947
Total		3169754	99133	100.000		

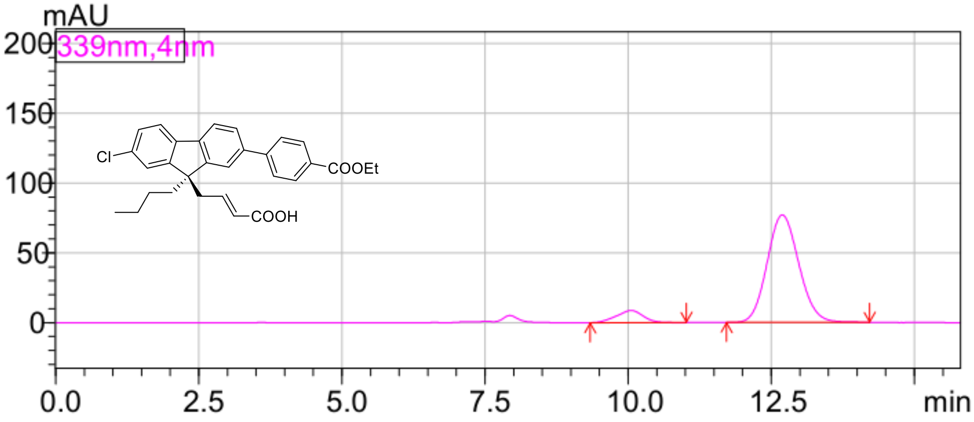

<Peak Table>

PDA Ch1 339nm

Peak#	Ret. Time	Height	Mark	Area%	Peak Start	Peak End
1	10.052	8652	M	8.638	9.339	11.019
2	12.692	77050	M	91.362	11.717	14.219
Total		85702		100.000		

2

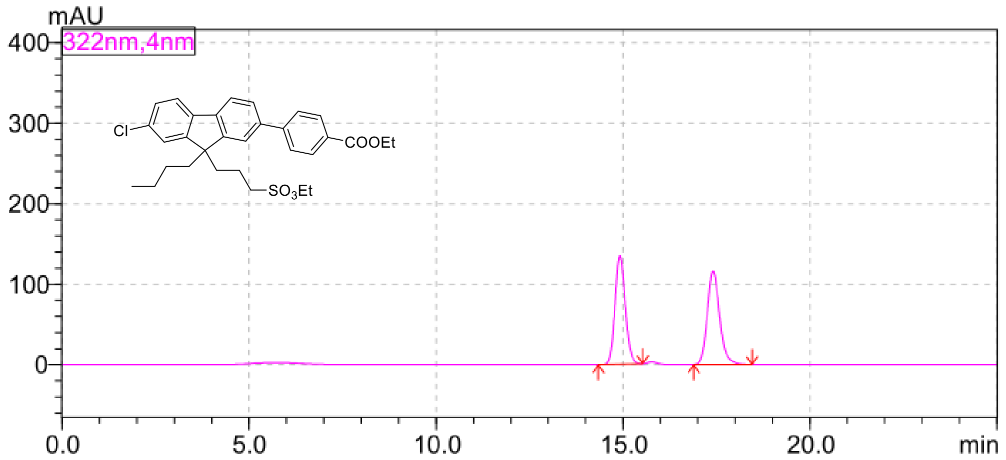

<Peak Table>

PDA Ch1 322nm

Peak#	Ret. Time	Area	Height	Area%	Peak Start	Peak End
1	14.918	2587026	134541	49.193	14.341	15.525
2	17.411	2671887	116080	50.807	16.896	18.453
Total		5258912	250621	100.000		

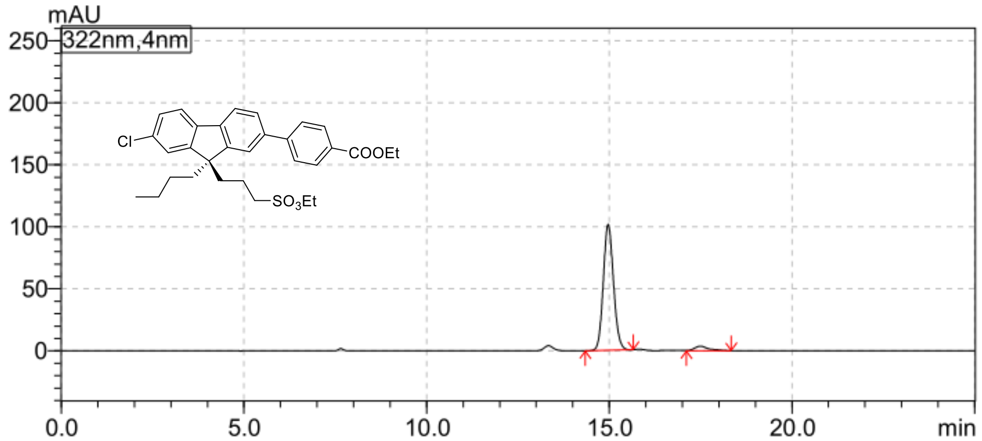

<Peak Table>

PDA Ch1 322nm

Peak#	Ret. Time	Area	Height	Mark	Area%	Peak Start	Peak End
1	14.968	1973528	101586	M	95.081	14.400	15.541
2	17.492	102100	3717	M	4.919	17.045	18.480
Total		2075628	105303		100.000		

14

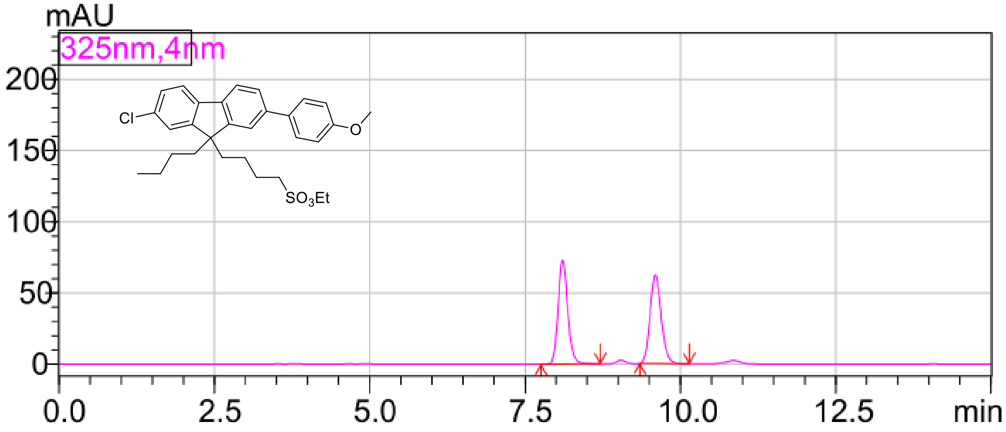

<Peak Table>

PDA Ch1 325nm

Peak#	Ret. Time	Area	Height	Area%	Peak Start	Peak End
1	8.104	788190	73055	50.293	7.755	8.709
2	9.596	779019	62088	49.707	9.344	10.144
Total		1567209	135143	100.000		

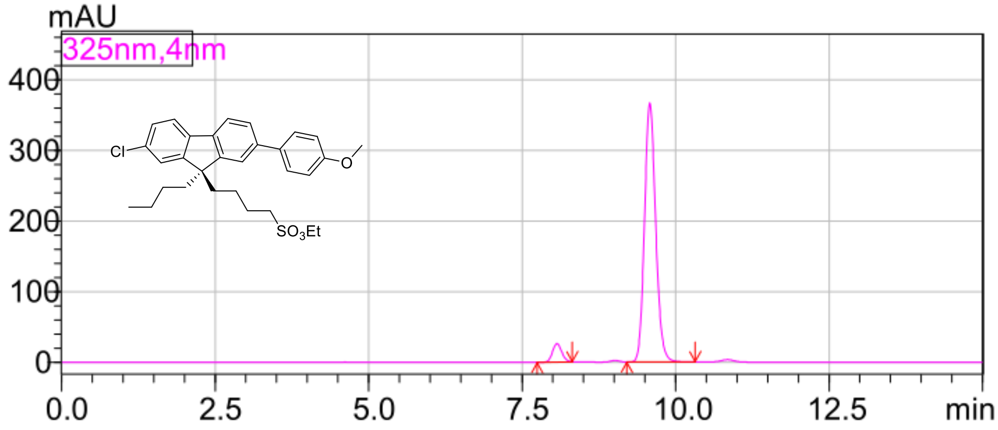

<Peak Table>

PDA Ch1 325nm

Peak#	Ret. Time	Area	Height	Conc.	Area%	Peak Start	Peak End
1	8.067	273386	26155	0.000	5.551	7.744	8.315
2	9.579	4651933	367155	0.000	94.449	9.205	10.320
Total		4925319	393310		100.000		

15

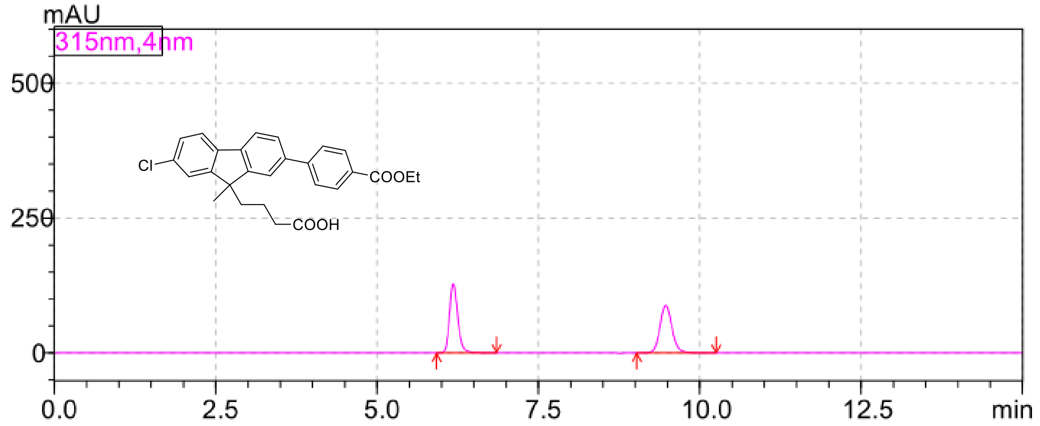

<Peak Table>

PDA Ch1 315nm

Peak#	Ret. Time	Area	Height	Mark	Area%	Peak Start	Peak End
1	6.180	1155997	128659	M	50.045	5.920	6.853
2	9.473	1153906	88102	M	49.955	9.024	10.256
Total		2309903	216761		100.000		

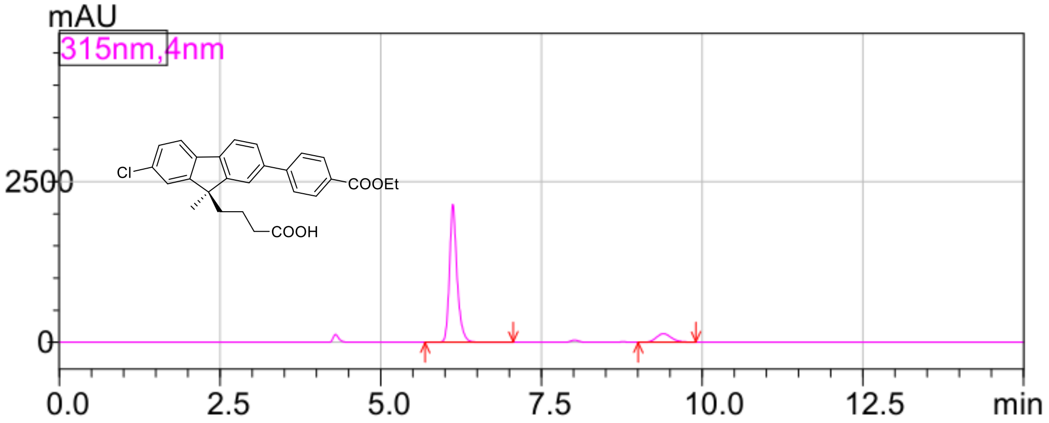

<Peak Table>

PDA Ch1 315nm

Peak#	Ret. Time	Area	Height	Mark	Area%	Peak Start	Peak End
1	6.121	17799005	2150253	M	88.882	5.691	7.061
2	9.397	2226353	133405	M	11.118	9.003	9.904
Total		20025357	2283658		100.000		

16

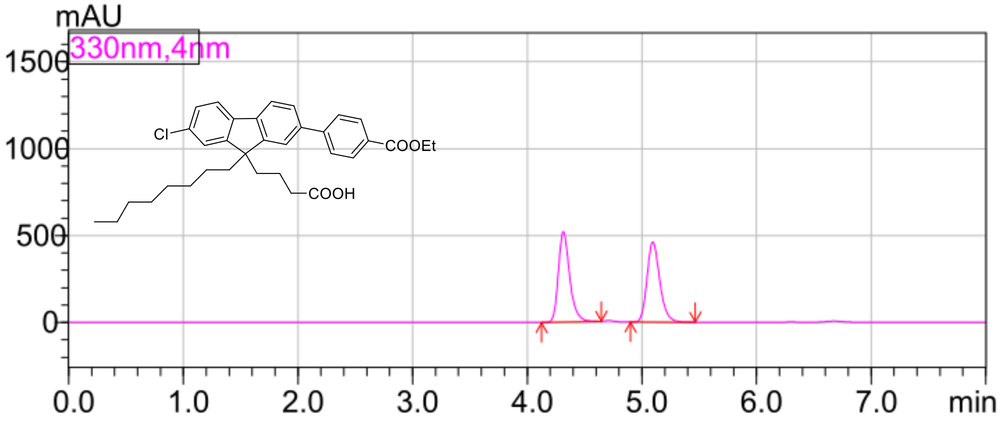

<Peak Table>

PDA Ch1 330nm

Peak#	Ret. Time	Area	Height	Mark	Area%	Peak Start	Peak End
1	4.315	3575747	520608	M	50.024	4.123	4.645
2	5.095	3572263	461260	M	49.976	4.901	5.467
Total		7148010	981868		100.000		

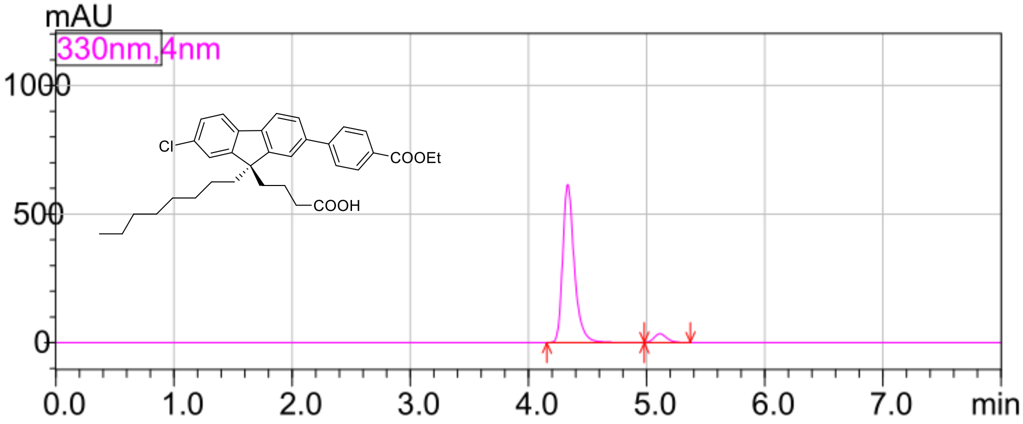

<Peak Table>

PDA Ch1 330nm

Peak#	Ret. Time	Area	Height	Mark	Area%	Peak Start	Peak End
1	4.332	4218194	615272	M	94.294	4.155	4.981
2	5.112	255263	33944	M	5.706	4.981	5.371
Total		4473457	649216		100.000		

17

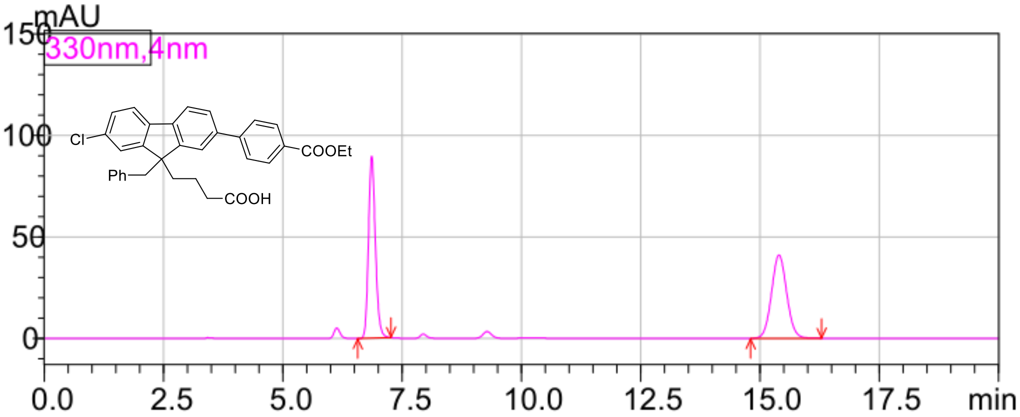

<Peak Table>

PDA Ch1 330nm

Peak#	Ret. Time	Area	Height	Mark	Area%	Peak Start	Peak End
1	6.861	903151	89618	M	49.791	6.565	7.264
2	15.397	910737	40979	M	50.209	14.805	16.288
Total		1813888	130597		100.000		

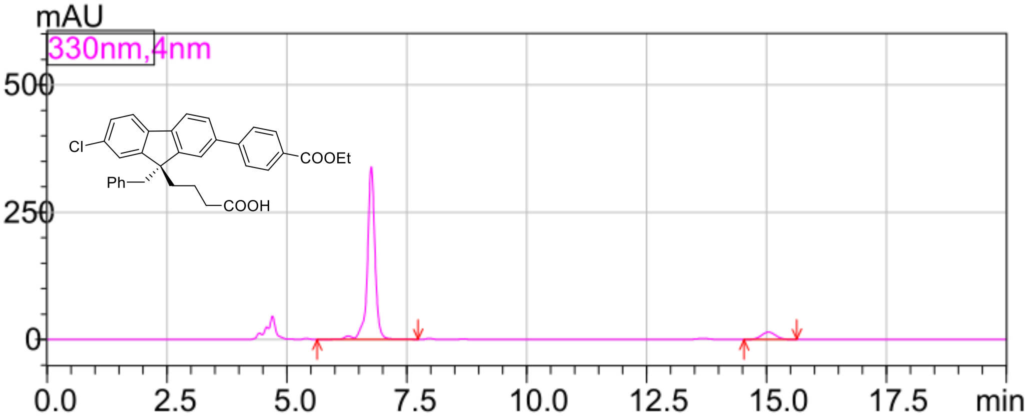

<Peak Table>

PDA Ch1 330nm

Peak#	Ret. Time	Area	Height	Mark	Area%	Peak Start	Peak End
1	6.758	3816870	339173	M	92.527	5.627	7.733
2	15.039	308283	14555	M	7.473	14.533	15.627
Total		4125154	353727		100.000		

18

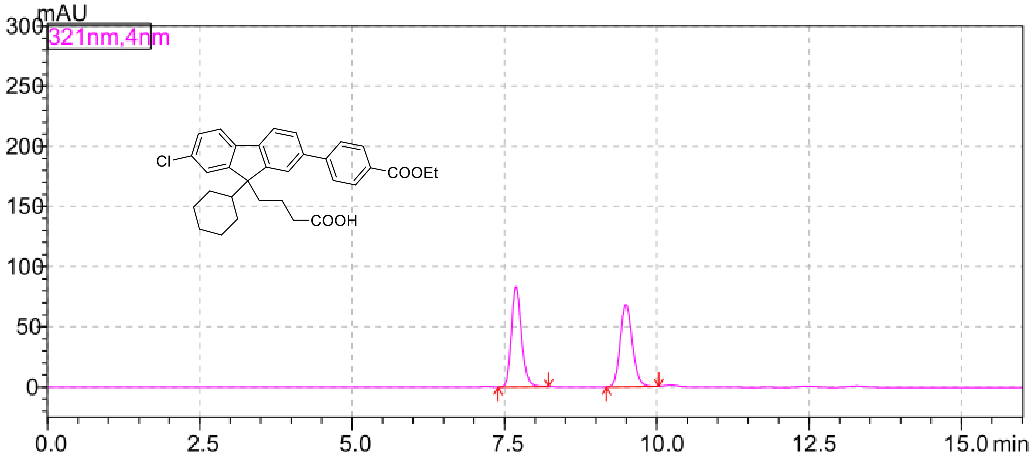

<Peak Table>

PDA Ch1 321nm

Peak#	Ret. Time	Area	Height	Area%	Peak Start	Peak End
1	7.688	967048	83194	50.450	7.392	8.224
2	9.493	949801	68318	49.550	9.173	10.032
Total		1916849	151512	100.000		

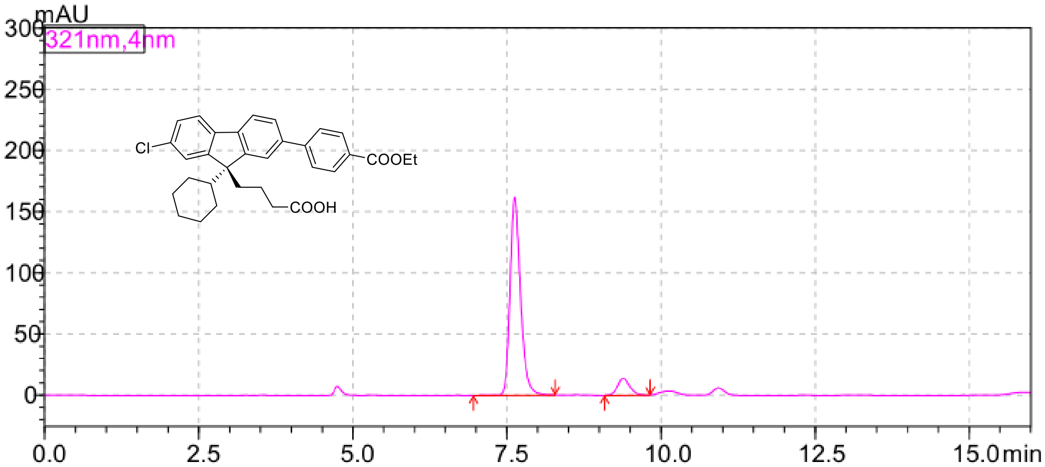

<Peak Table>

PDA Ch1 321nm

Peak#	Ret. Time	Area	Height	Area%	Peak Start	Peak End
1	7.626	1916651	161845	91.108	6.955	8.283
2	9.388	187064	13659	8.892	9.083	9.824
Total		2103715	175504	100.000		

19

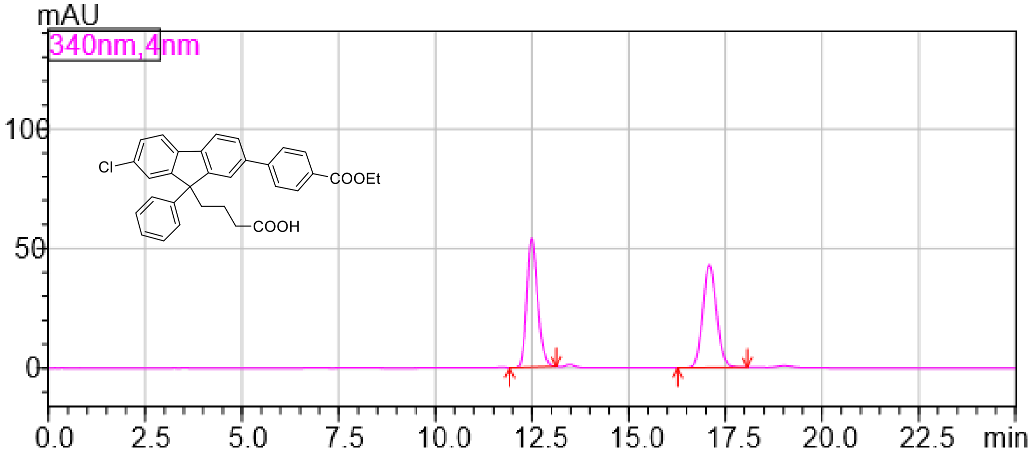

<Peak Table>

PDA Ch1 340nm

Peak#	Ret. Time	Area	Height	Mark	Area%	Peak Start	Peak End
1	12.491	1052688	53804	M	49.606	11.920	13.125
2	17.089	1069401	42927	M	50.394	16.267	18.069
Total		2122089	96731		100.000		

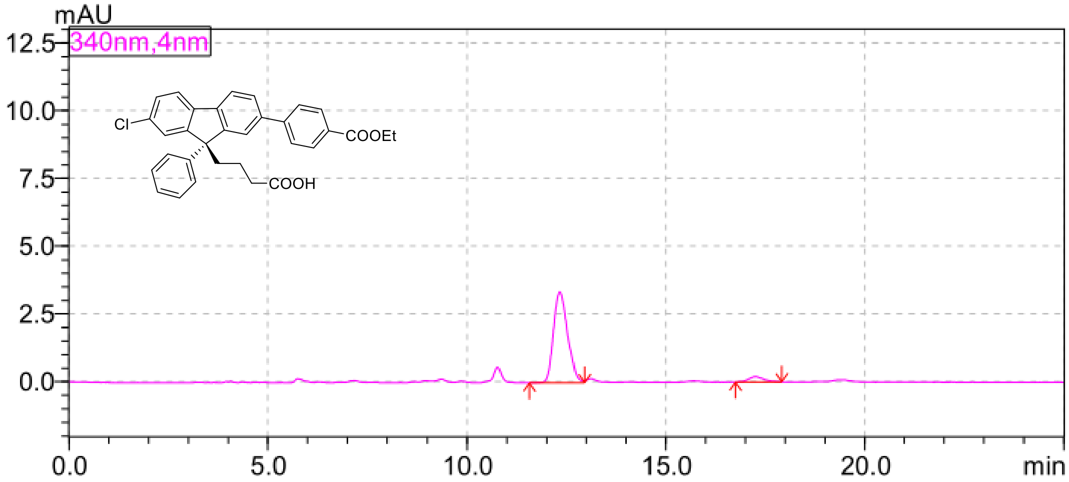

<Peak Table>

PDA Ch1 340nm

Peak#	Ret. Time	Area	Height	Area%	Peak Start	Peak End
1	12.334	84483	3355	94.387	11.568	12.955
2	17.251	5024	199	5.613	16.741	17.915
Total		89507	3553	100.000		

20

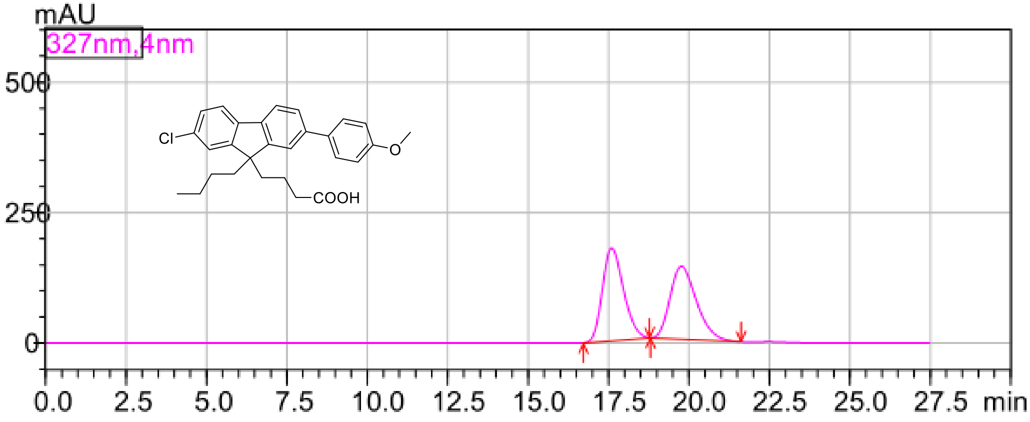

<Peak Table>

PDA Ch1 327nm

Peak#	Ret. Time	Area	Height	Mark	Area%	Peak Start	Peak End
1	17.597	8088778	177472	M	50.255	16.715	18.773
2	19.763	8006616	139737	M	49.745	18.805	21.621
Total		16095394	317209		100.000		

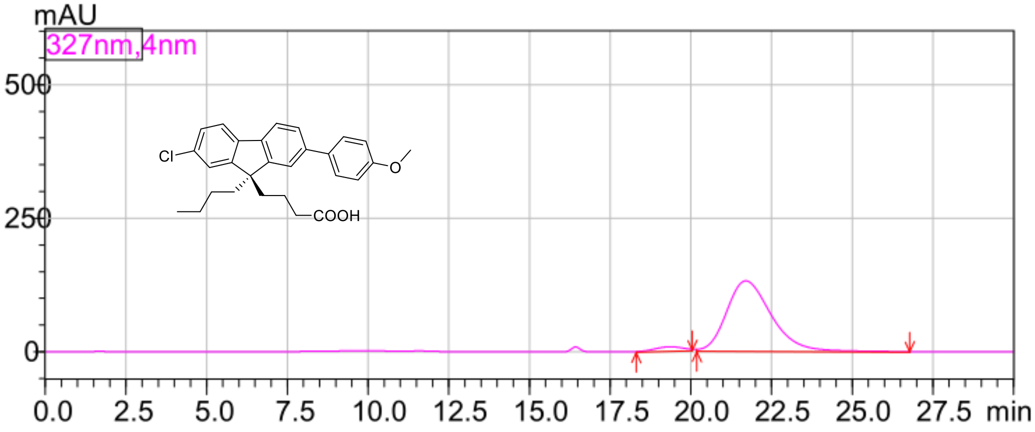

<Peak Table>

PDA Ch1 327nm

Peak#	Ret. Time	Area	Height	Mark	Area%	Peak Start	Peak End
1	19.363	553959	8652	M	4.073	18.309	20.048
2	21.704	13045579	132482	M	95.927	20.181	26.779
Total		13599537	141133		100.000		

21

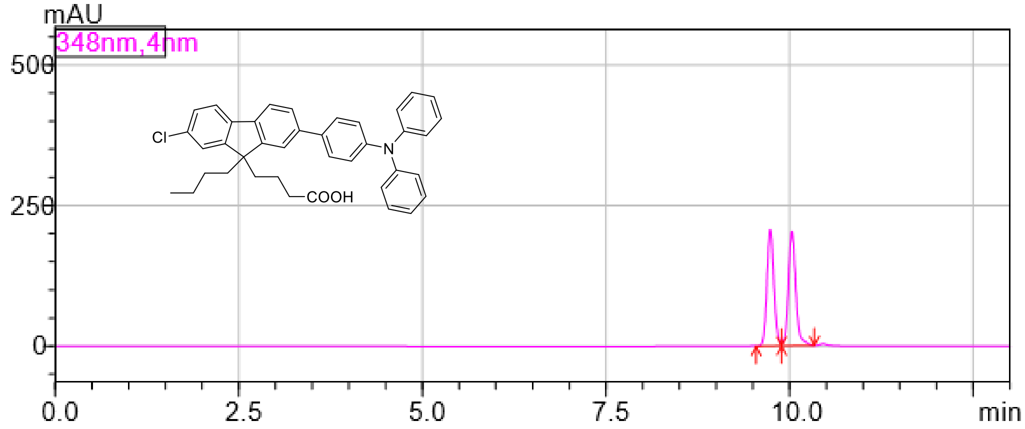

<Peak Table>

PDA Ch1 348nm

Peak#	Ret. Time	Area	Height	Peak Start	Peak End	Area%
1	9.736	1361527	206723	9.547	9.888	48.654
2	10.032	1436878	203282	9.888	10.336	51.346
Total		2798406	410005			100.000

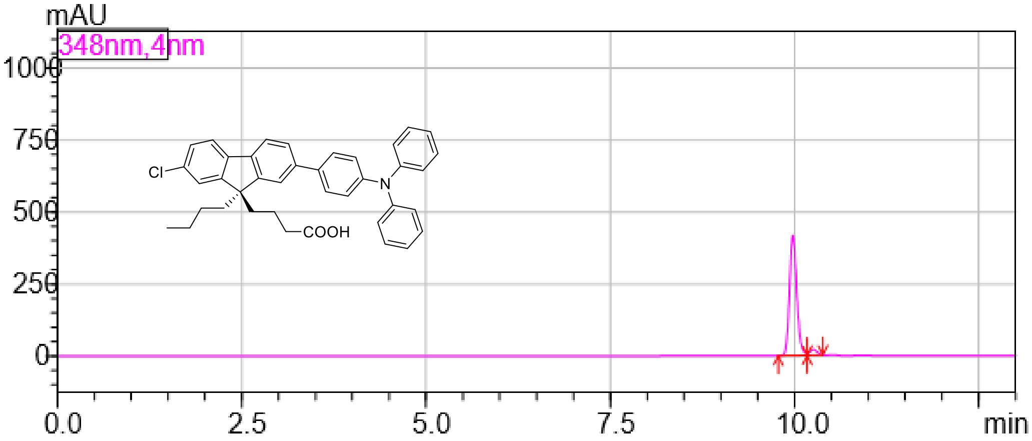

<Peak Table>

PDA Ch1 348nm

Peak#	Ret. Time	Area	Height	Peak Start	Peak End	Area%
1	9.977	2863714	416396	9.776	10.171	95.420
2	10.257	137452	19619	10.171	10.384	4.580
Total		3001166	436015			100.000

22

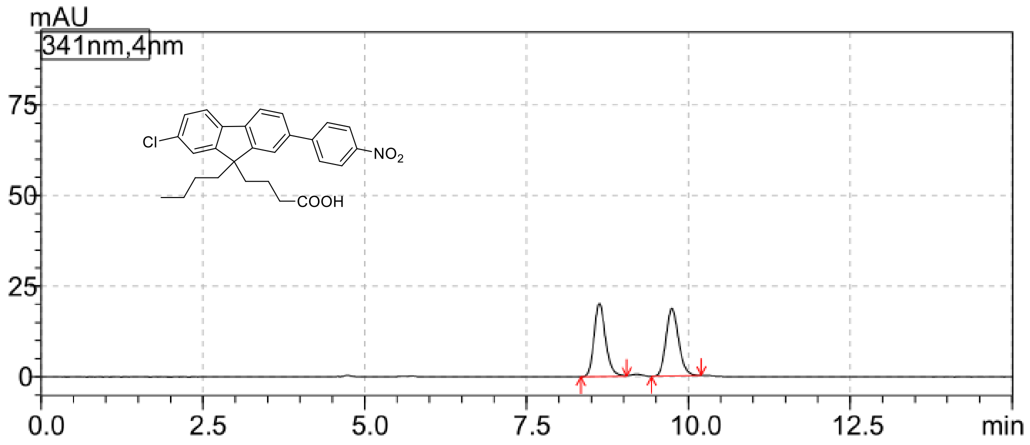

<Peak Table>

PDA Ch1 340nm

Peak#	Ret. Time	Area	Height	Mark	Area%	Peak Start	Peak End
1	8.624	247208	20228	M	49.076	8.352	8.992
2	9.744	256514	18866	M	50.924	9.435	10.224
Total		503722	39094		100.000		

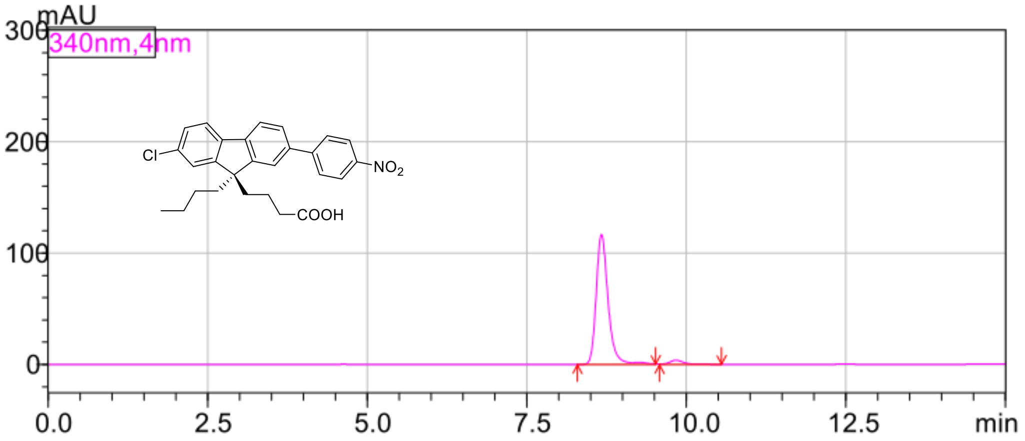

<Peak Table>

PDA Ch1 340nm

Peak#	Ret. Time	Area	Height	Mark	Area%	Peak Start	Peak End
1	8.669	1536823	117003	M	96.023	8.293	9.520
2	9.837	63650	3811	M	3.977	9.579	10.549
Total		1600473	120814		100.000		

23

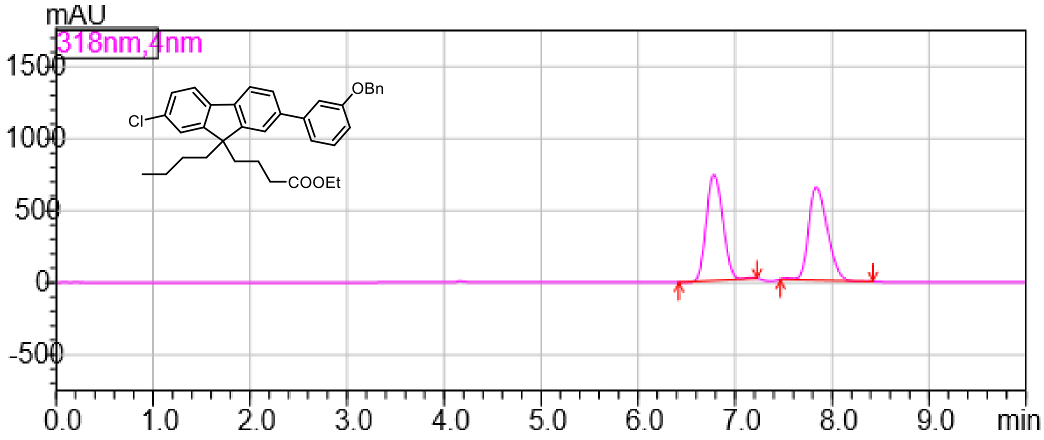

<Peak Table>

PDA Ch1 318nm

Peak#	Ret. Time	Area	Height	Peak Start	Peak End	Area%
1	6.782	8763723	735594	6.421	7.227	50.226
2	7.838	8684872	645717	7.467	8.421	49.774
Total		17448595	1381311			100.000

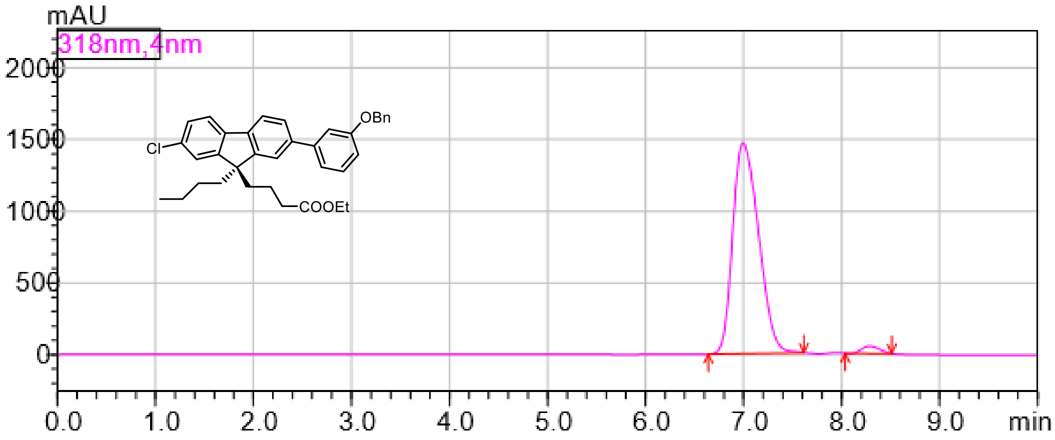

<Peak Table>

PDA Ch1 318nm

Peak#	Ret. Time	Area	Height	Peak Start	Peak End	Area%
1	6.997	26933869	1467169	6.640	7.616	97.678
2	8.287	640297	51242	8.037	8.512	2.322
Total		27574165	1518411			100.000

24

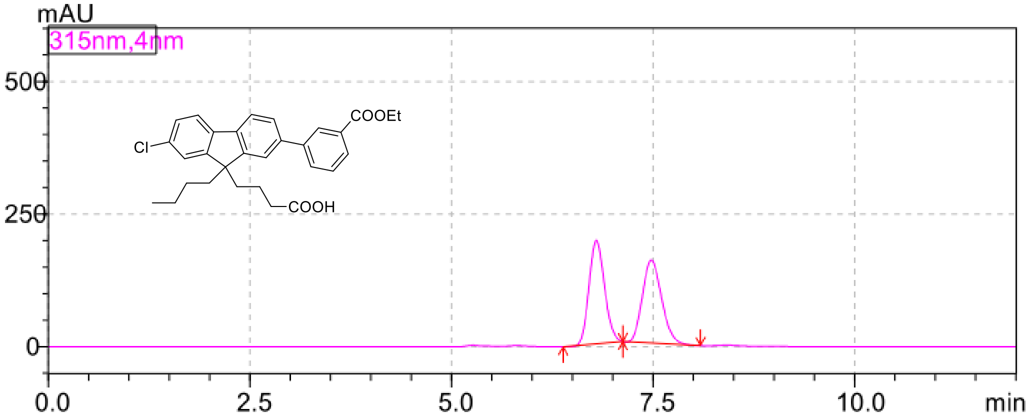

<Peak Table>

PDA Ch1 315nm

Peak#	Ret. Time	Area	Height	Mark	Area%	Peak Start	Peak End
1	6.797	2611192	195302	M	50.069	6.384	7.125
2	7.479	2604031	156414	M	49.931	7.125	8.091
Total		5215224	351716		100.000		

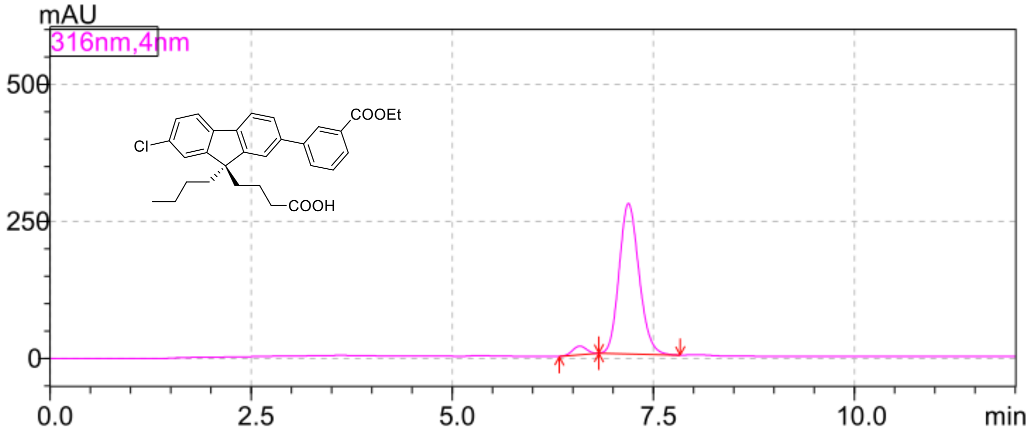

<Peak Table>

PDA Ch1 316nm

Peak#	Ret. Time	Area	Height	Mark	Area%	Peak Start	Peak End
1	6.585	186956	15590	M	3.800	6.331	6.821
2	7.190	4732592	274482	M	96.200	6.821	7.835
Total		4919548	290072		100.000		

25

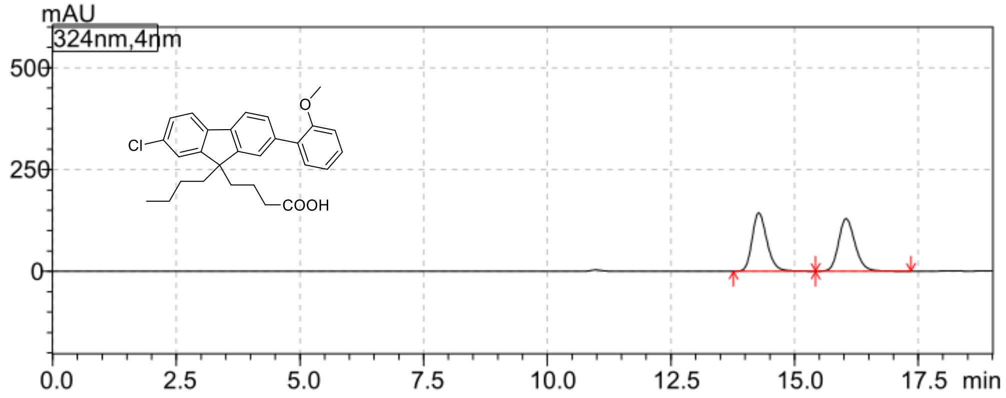

<Peak Table>

PDA Ch1 324nm

Peak#	Ret. Time	Area	Height	Mark	Area%	Peak Start	Peak End
1	14.275	2982301	143459	M	49.944	13.691	15.328
2	16.040	2988939	128994	M	50.056	15.365	17.259
Total		5971240	272453		100.000		

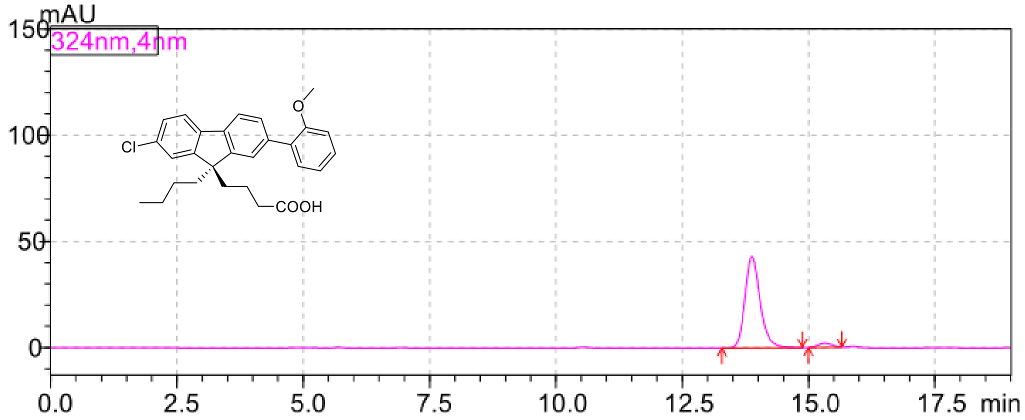

<Peak Table>

PDA Ch1 324nm

Peak#	Ret. Time	Area	Height	Mark	Area%	Peak Start	Peak End
1	13.876	896974	43068	M	96.278	13.285	14.880
2	15.321	34680	1824	M	3.722	14.992	15.659
Total		931655	44892		100.000		

26

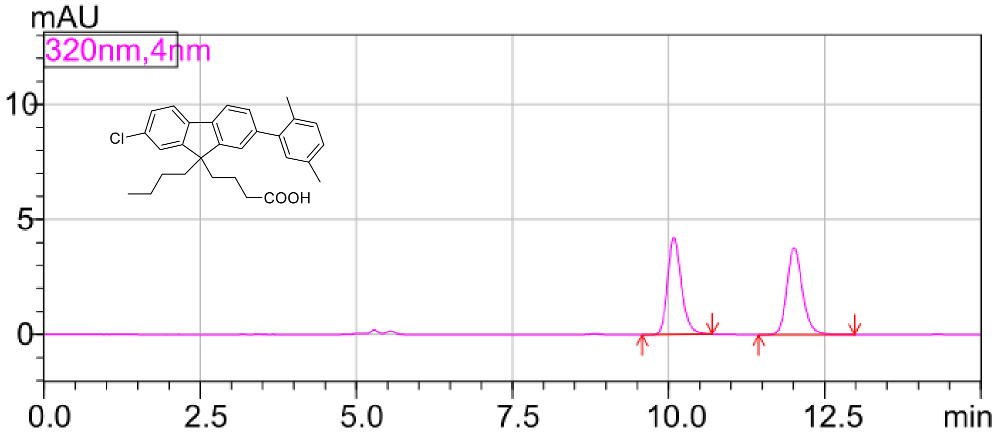

<Peak Table>

PDA Ch1 320nm

Peak#	Ret. Time	Area	Height	Area%	Peak Start	Peak End
1	10.085	65184	4242	49.705	9.579	10.699
2	12.011	65957	3790	50.295	11.445	12.981
Total		131141	8032	100.000		

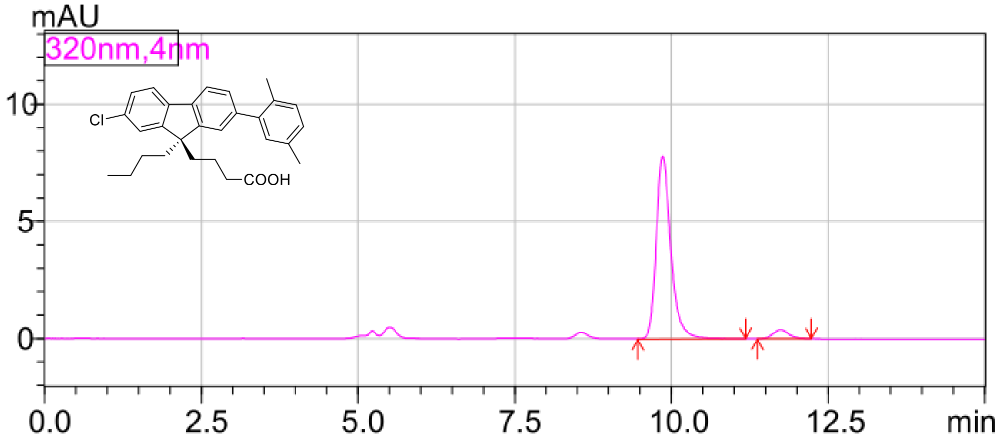

<Peak Table>

PDA Ch1 320nm

Peak#	Ret. Time	Area	Height	Area%	Peak Start	Peak End
1	9.862	126730	7809	94.842	9.467	11.184
2	11.746	6892	388	5.158	11.371	12.235
Total		133621	8197	100.000		

27

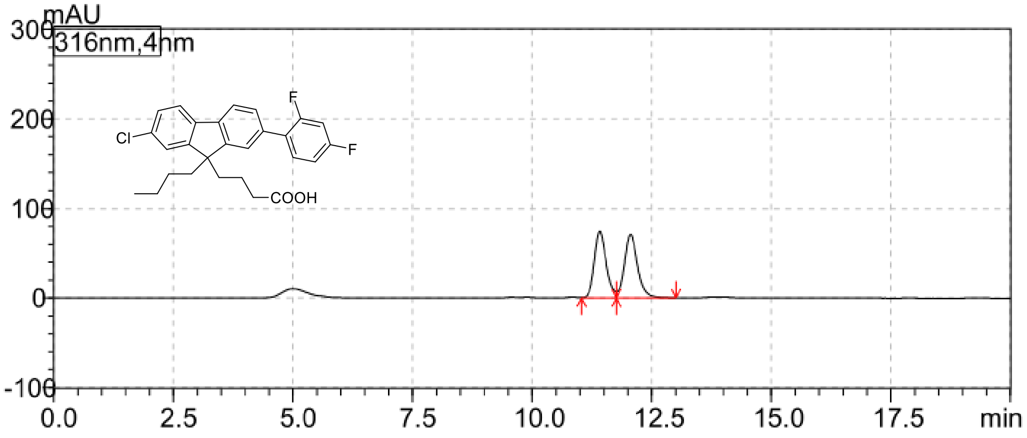

<Peak Table>

PDA Ch1 316nm

Peak#	Ret. Time	Area	Height	Mark	Area%	Peak Start	Peak End
1	11.412	1135981	70656	M	50.071	11.019	11.760
2	12.056	1132760	66036	M	49.929	11.760	12.811
Total		2268742	136692		100.000		

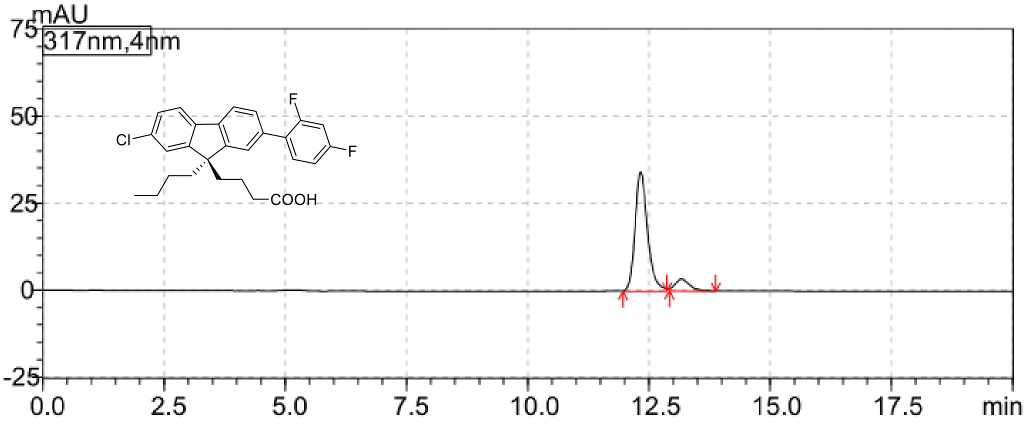

<Peak Table>

PDA Ch1 316nm

Peak#	Ret. Time	Area	Height	Mark	Area%	Peak Start	Peak End
1	12.328	606322	34836	M	92.982	11.680	12.928
2	13.174	45762	2794	M	7.018	12.971	13.632
Total		652085	37630		100.000		

28

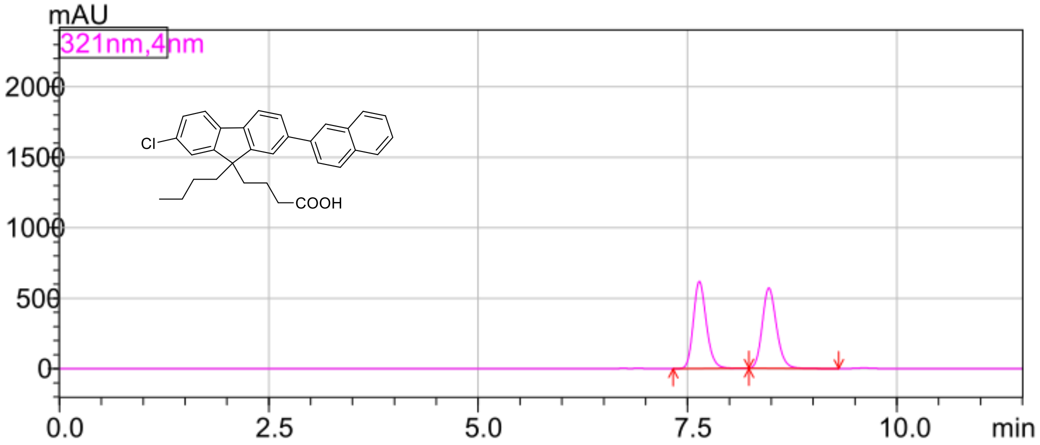

<Peak Table>

PDA Ch1 321nm

Peak#	Ret. Time	Area	Height	Mark	Area%	Peak Start	Peak End
1	7.641	6650777	618104	M	49.850	7.328	8.235
2	8.472	6690701	569043	M	50.150	8.235	9.307
Total		13341478	1187148		100.000		

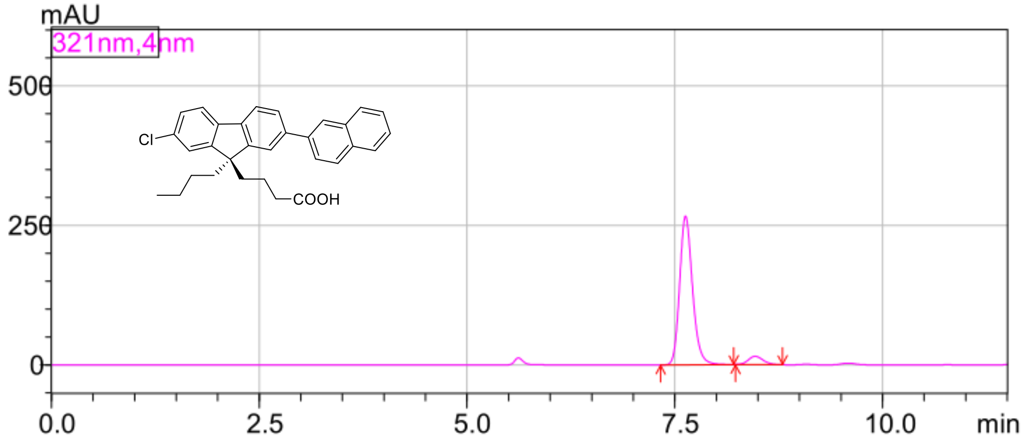

<Peak Table>

PDA Ch1 321nm

Peak#	Ret. Time	Area	Height	Mark	Area%	Peak Start	Peak End
1	7.629	2860174	266404	M	94.007	7.328	8.208
2	8.467	182351	14803	M	5.993	8.229	8.795
Total		3042525	281207		100.000		

29

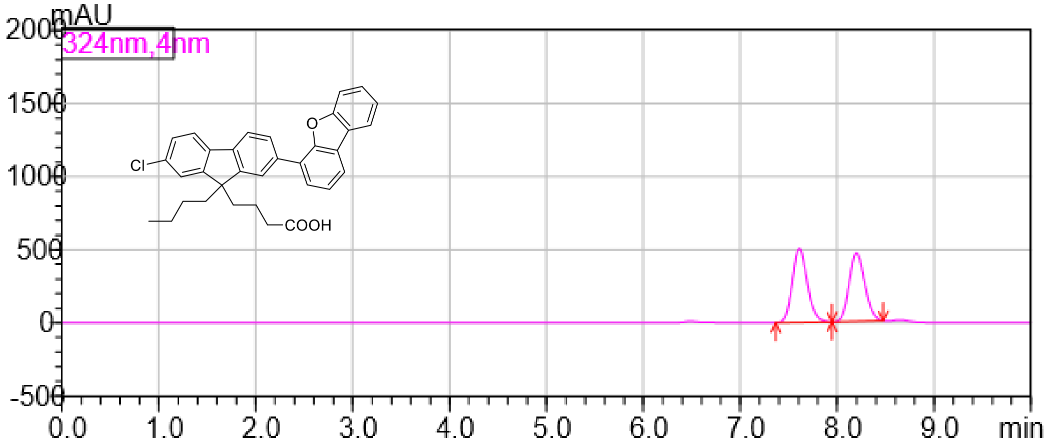

<Peak Table>

PDA Ch1 324nm

Peak#	Ret. Time	Area	Height	Mark	Area%	Peak Start	Peak End
1	7.608	5462436	504995	M	50.922	7.365	7.947
2	8.199	5264578	467558	M	49.078	7.947	8.475
Total		10727014	972553		100.000		

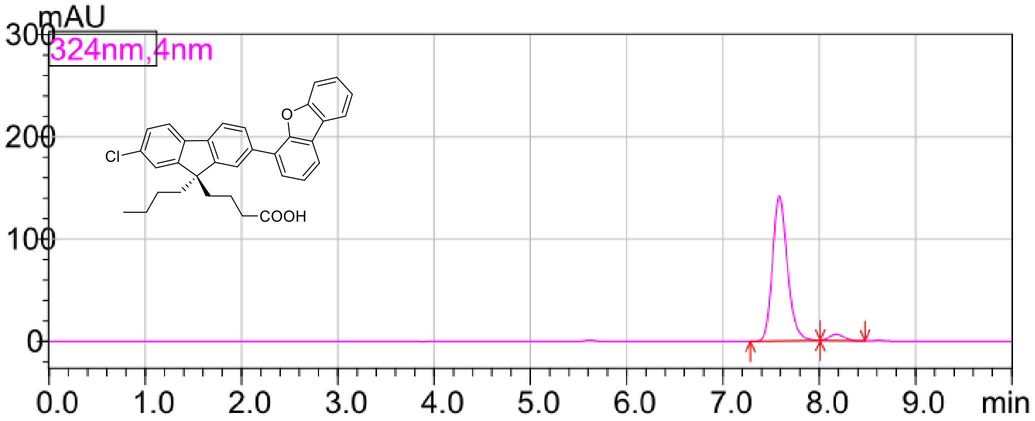

<Peak Table>

PDA Ch1 324nm

Peak#	Ret. Time	Area	Height	Mark	Area%	Peak Start	Peak End
1	7.585	1528908	141835	M	95.937	7.285	8.011
2	8.176	64744	6234	M	4.063	8.011	8.475
Total		1593652	148069		100.000		

30

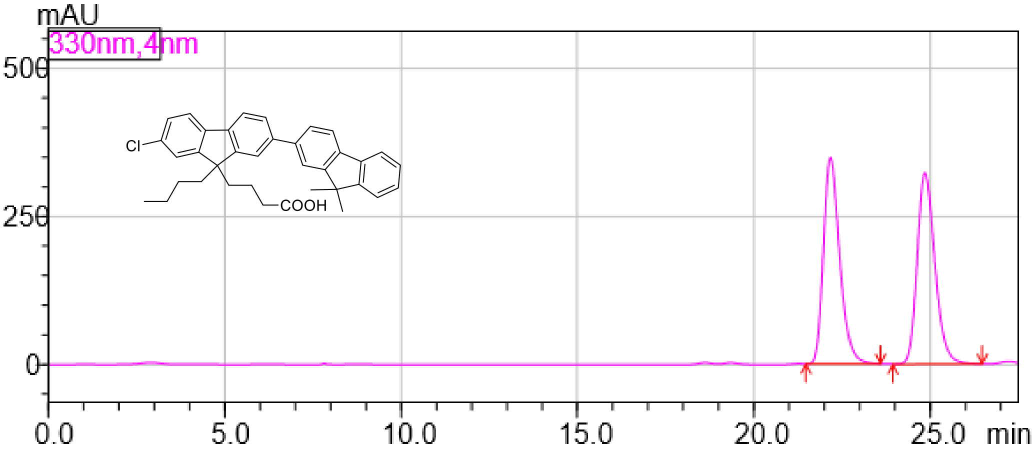

<Peak Table>

PDA Ch1 330nm

Peak#	Ret. Time	Area	Height	Mark	Area%	Peak Start	Peak End
1	22.168	11172774	347968	M	49.811	21.472	23.589
2	24.851	11257343	322486	M	50.189	23.941	26.469
Total		22430117	670454		100.000		

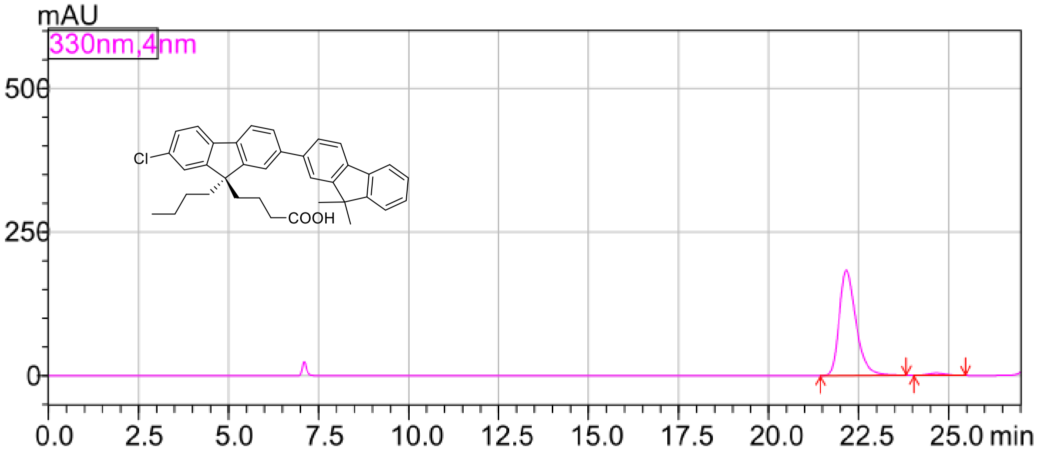

<Peak Table>

PDA Ch1 330nm

Peak#	Ret. Time	Area	Height	Mark	Area%	Peak Start	Peak End
1	22.160	5934338	183594	M	97.988	21.440	23.824
2	24.657	121821	3695	M	2.012	24.037	25.467
Total		6056159	187289		100.000		

31

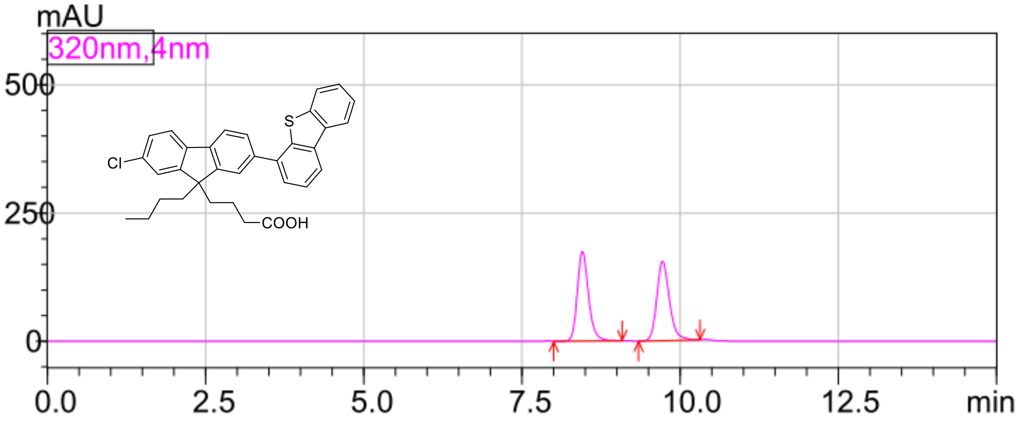

<Peak Table>

PDA Ch1 320nm

Peak#	Ret. Time	Area	Height	Mark	Area%	Peak Start	Peak End
1	8.454	2150008	174394	M	50.057	8.000	9.083
2	9.722	2145135	154725	M	49.943	9.344	10.315
Total		4295142	329119		100.000		

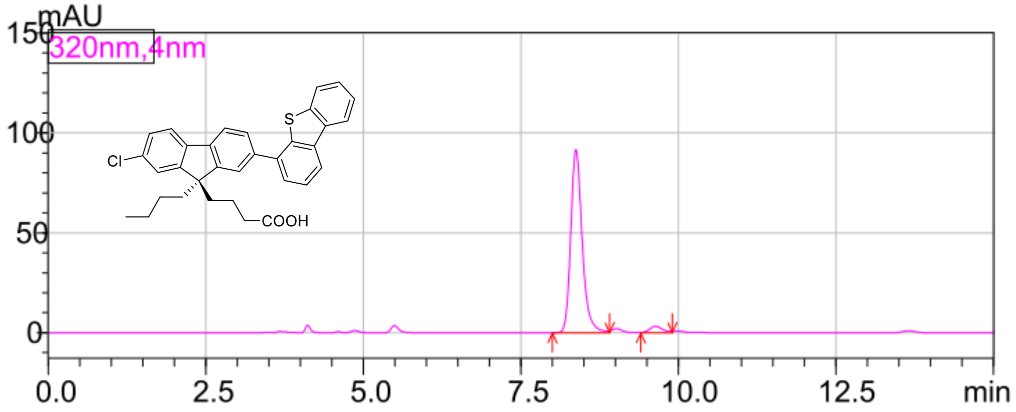

<Peak Table>

PDA Ch1 320nm

Peak#	Ret. Time	Area	Height	Mark	Area%	Peak Start	Peak End
1	8.373	1161882	91670	M	96.007	8.000	8.907
2	9.638	48320	3275	M	3.993	9.403	9.904
Total		1210202	94945		100.000		

32

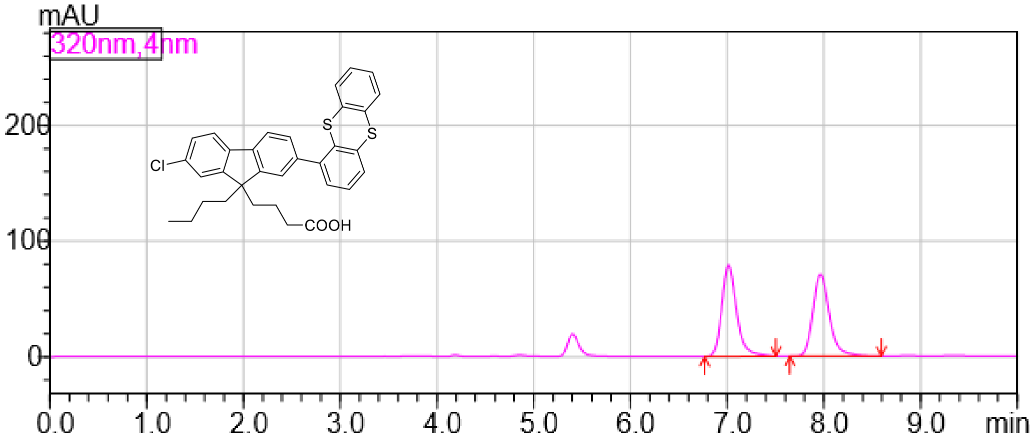

<Peak Table>

PDA Ch1 320nm

Peak#	Ret. Time	Area	Height	Peak Start	Peak End	Area%
1	7.013	852808	79008	6.768	7.504	50.289
2	7.964	843016	70841	7.648	8.592	49.711
Total		1695824	149849			100.000

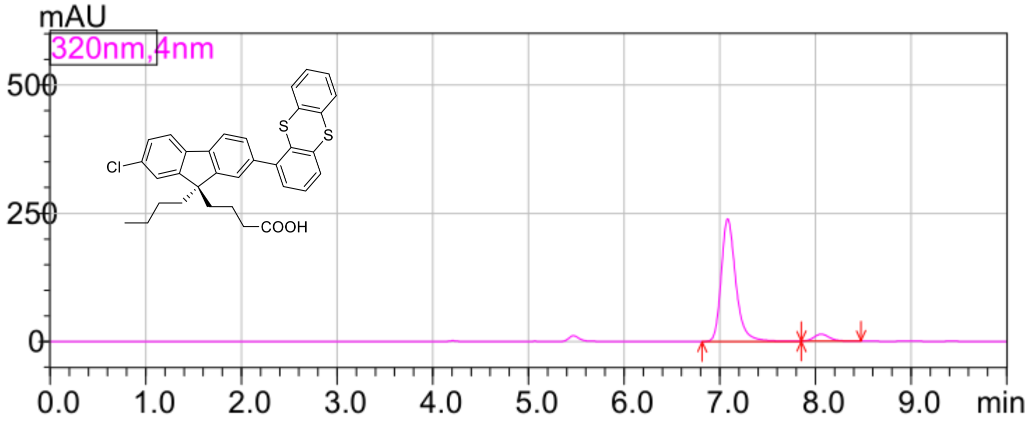

<Peak Table>

PDA Ch1 320nm

Peak#	Ret. Time	Area	Height	Mark	Area%	Peak Start	Peak End
1	7.082	2550028	239479	M	94.269	6.816	7.851
2	8.060	155032	13654	M	5.731	7.851	8.475
Total		2705060	253133		100.000		

33

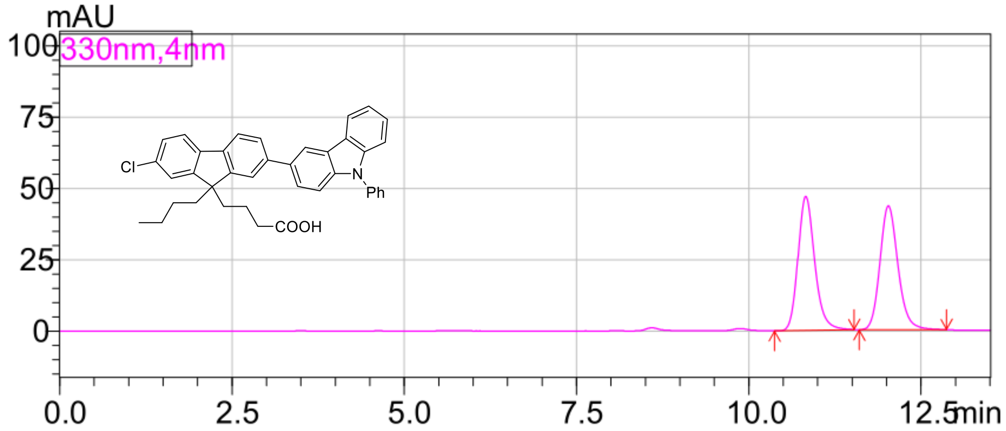

<Peak Table>

PDA Ch1 330nm

Peak#	Ret. Time	Area	Height	Mark	Area%	Peak Start	Peak End
1	10.824	792380	47029	M	49.866	10.373	11.531
2	12.026	796636	43460	M	50.134	11.600	12.869
Total		1589016	90489		100.000		

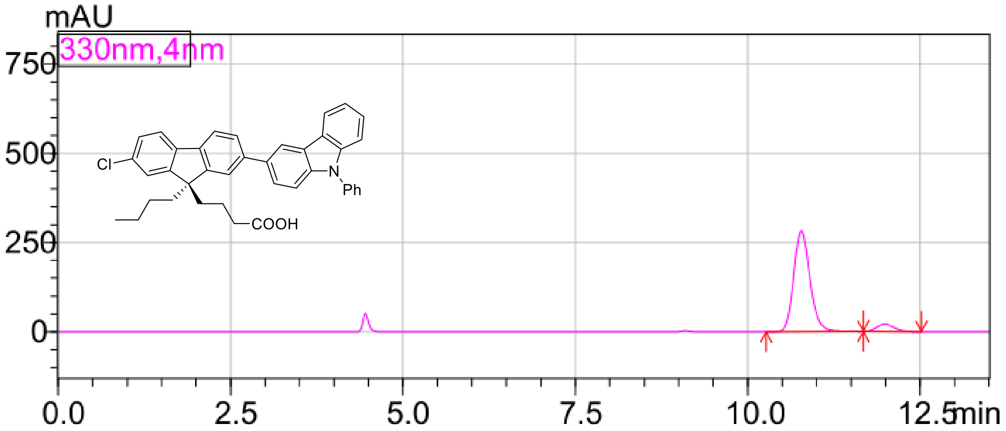

<Peak Table>

PDA Ch1 330nm

Peak#	Ret. Time	Area	Height	Mark	Area%	Peak Start	Peak End
1	10.775	4590288	282862	M	92.689	10.267	11.675
2	11.988	362090	20630	M	7.311	11.675	12.517
Total		4952378	303492		100.000		

34

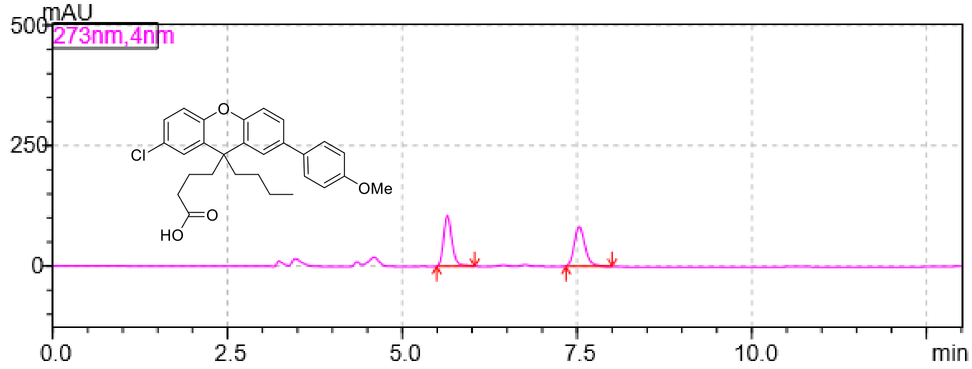

<Peak Table>

PDA Ch1 273nm

Peak#	Ret. Time	Area	Height	Area%	Peak Start	Peak End
1	5.645	906091	105748	50.371	5.493	6.037
2	7.532	892761	82420	49.629	7.344	8.000
Total		1798853	188167	100.000		

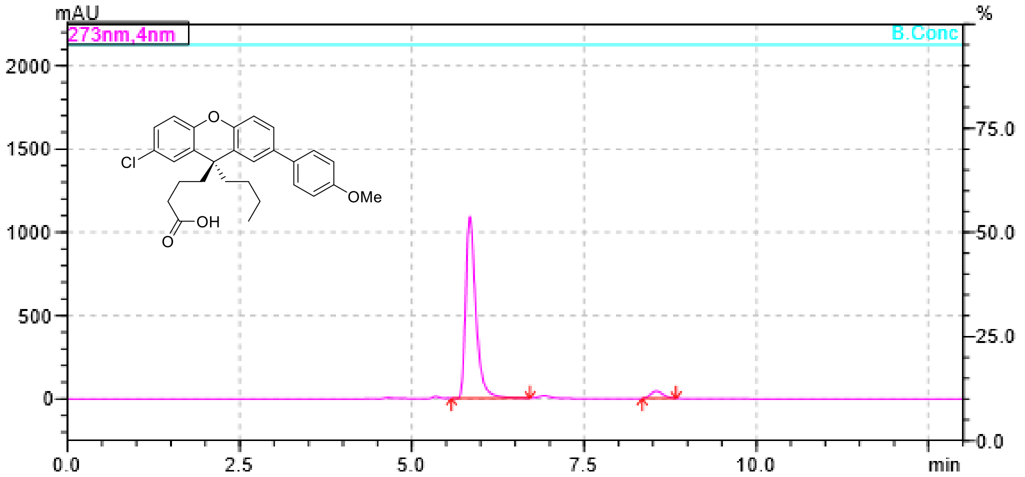

<Peak Table>

PDA Ch1 273nm

Peak#	Ret. Time	Area	Height	Area%	Peak Start	Peak End
1	5.840	11259551	1092140	95.297	5.573	6.715
2	8.549	555671	43655	4.703	8.341	8.832
Total		11815222	1135795	100.000		

***ent*-34**

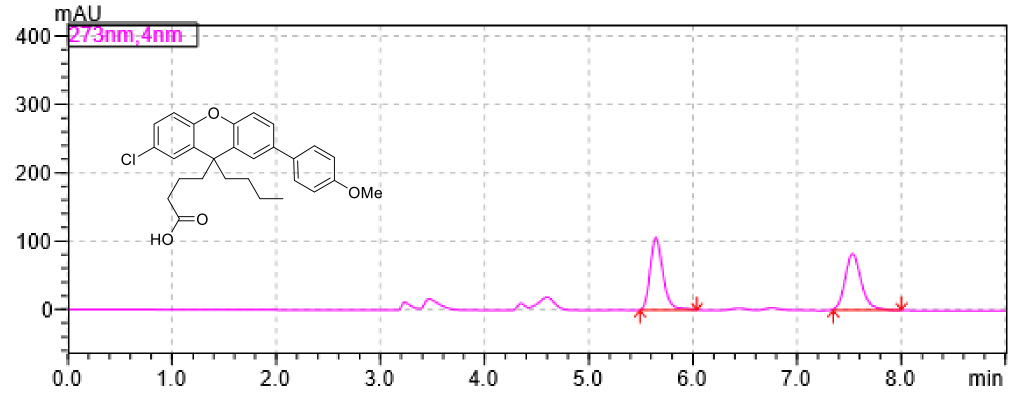

<Peak Table>

PDA Ch1 273nm

Peak#	Ret. Time	Area	Height	Area%	Peak Start	Peak End
1	5.645	906091	105748	50.371	5.493	6.037
2	7.532	892761	82420	49.629	7.344	8.000
Total		1798853	188167	100.000		

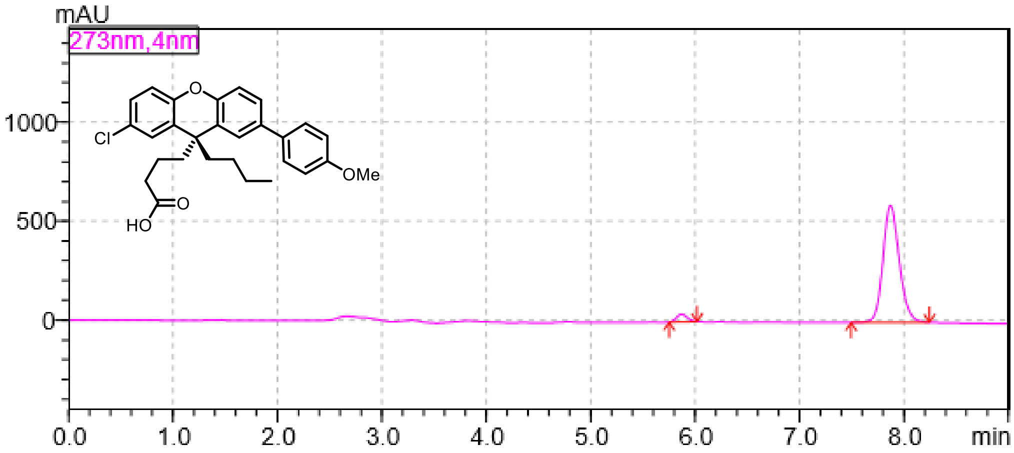

<Peak Table>

PDA Ch1 273nm

Peak#	Ret. Time	Area	Height	Area%	Peak Start	Peak End
1	5.870	269620	37049	4.025	5.749	6.016
2	7.871	6428568	590825	95.975	7.493	8.245
Total		6698188	627874	100.000		

35

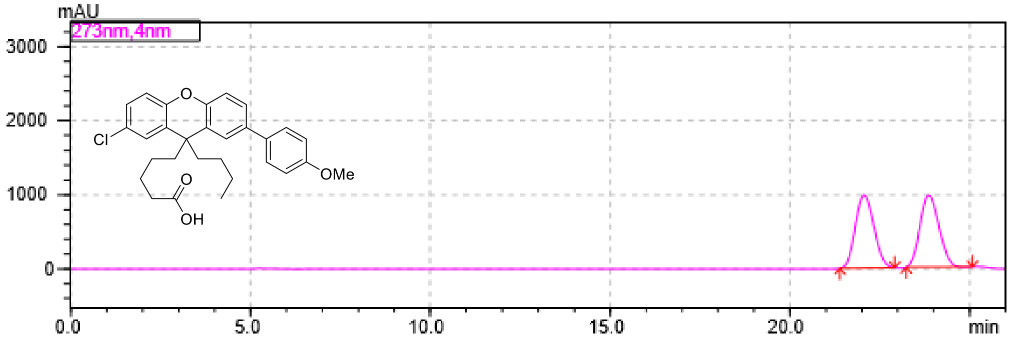

<Peak Table>

PDA Ch1 273nm

Peak#	Ret. Time	Area	Height	Mark	Peak Start	Peak End	Area%
1	22.063	35610635	988909	M	21.387	22.912	49.999
2	23.862	35612259	968627	M	23.232	25.077	50.001
Total		71222894	1957536				100.000

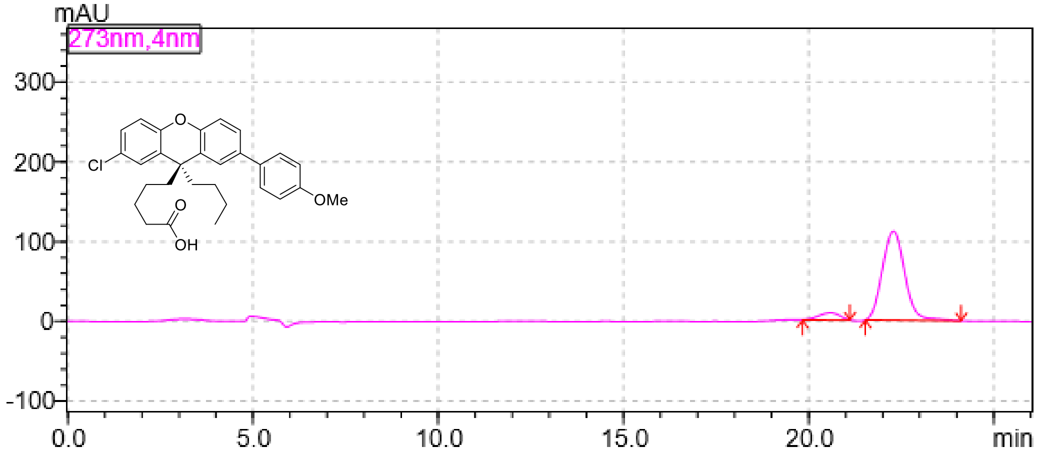

<Peak Table>

PDA Ch1 273nm

Peak#	Ret. Time	Area	Height	Mark	Peak Start	Peak End	Area%
1	20.587	344742	8950	M	19.829	21.115	6.826
2	22.291	4705984	111632	M	21.531	24.112	93.174
Total		5050725	120583				100.000

36

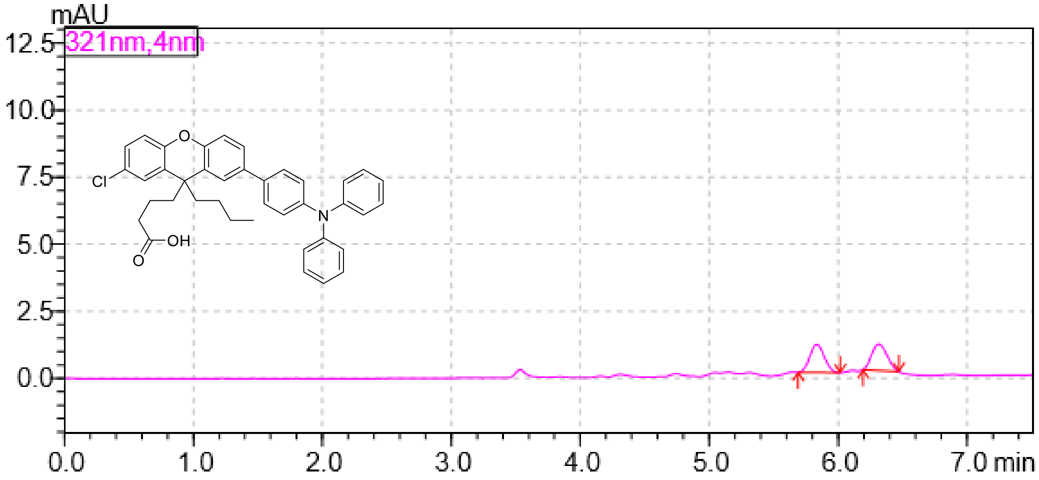

<Peak Table>

PDA Ch1 321nm

Peak#	Ret. Time	Area	Height	Area%	Peak Start	Peak End
1	5.832	8160	1037	49.895	5.691	6.016
2	6.315	8194	986	50.105	6.192	6.469
Total		16354	2023	100.000		

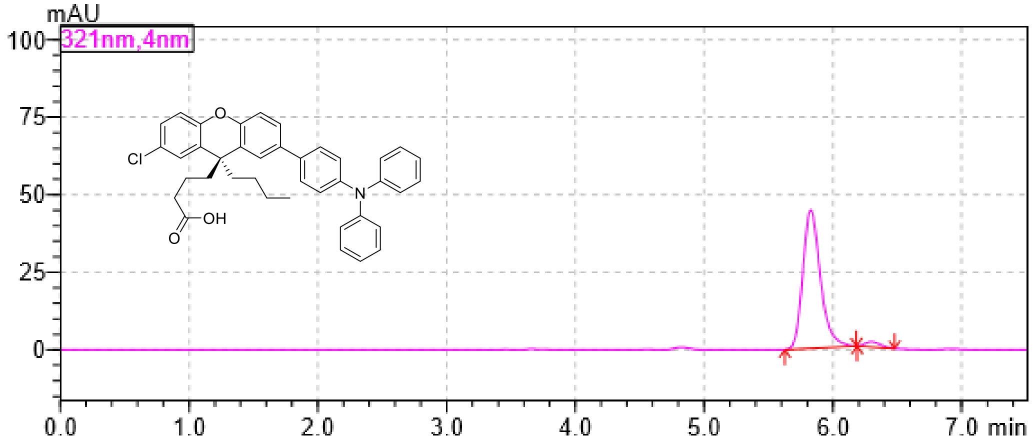

<Peak Table>

PDA Ch1 321nm

Peak#	Ret. Time	Area	Height	Area%	Peak Start	Peak End
1	5.827	425862	44585	96.856	5.627	6.181
2	6.301	13826	1698	3.144	6.187	6.480
Total		439688	46283	100.000		

37

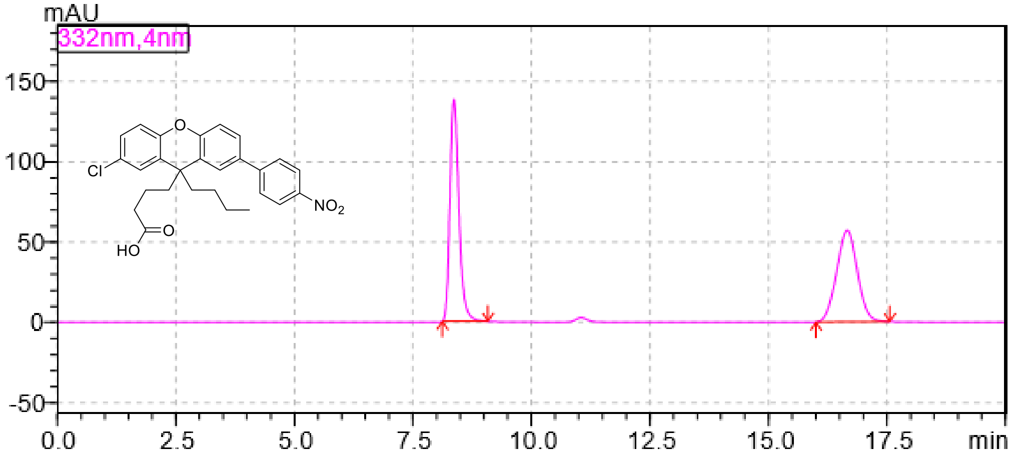

<Peak Table>

PDA Ch1 332nm

Peak#	Ret. Time	Area	Height	Area%	Peak Start	Peak End
1	8.367	1805772	138326	50.888	8.123	9.088
2	16.664	1742738	57134	49.112	16.005	17.563
Total		3548510	195461	100.000		

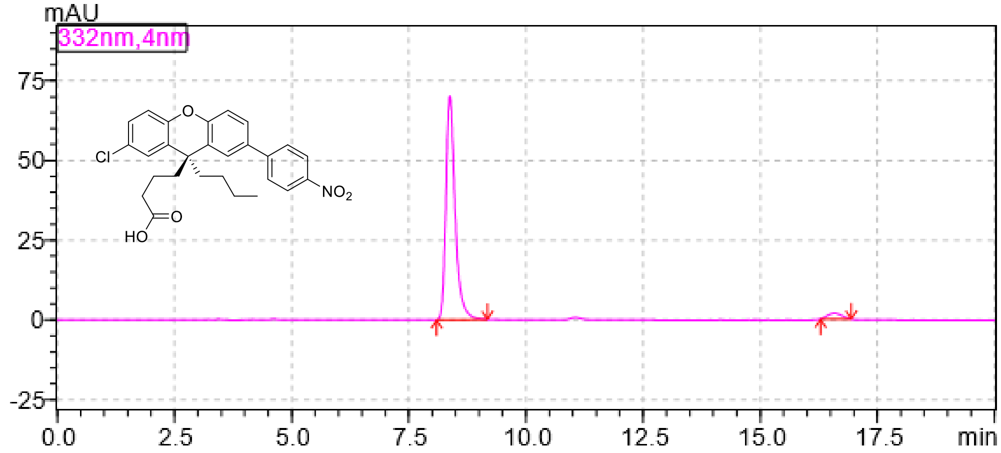

<Peak Table>

PDA Ch1 332nm

Peak#	Ret. Time	Area	Height	Area%	Peak Start	Peak End
1	8.376	959272	70145	96.188	8.096	9.173
2	16.583	38012	1825	3.812	16.283	16.944
Total		997284	71970	100.000		

38

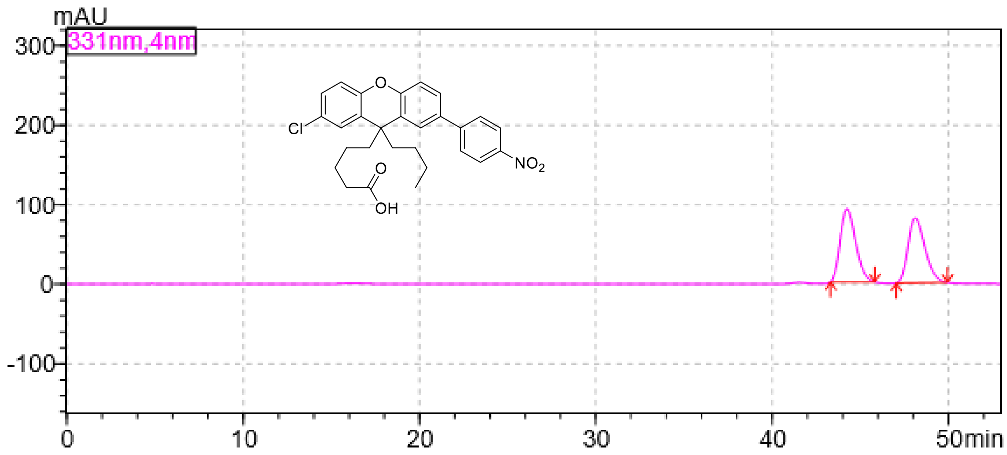

<Peak Table>

PDA Ch1 331nm

Peak#	Ret. Time	Area	Height	Area%	Peak Start	Peak End
1	44.249	5620073	91863	50.756	43.312	45.824
2	48.120	5452641	81630	49.244	47.029	49.941
Total		11072714	173493	100.000		

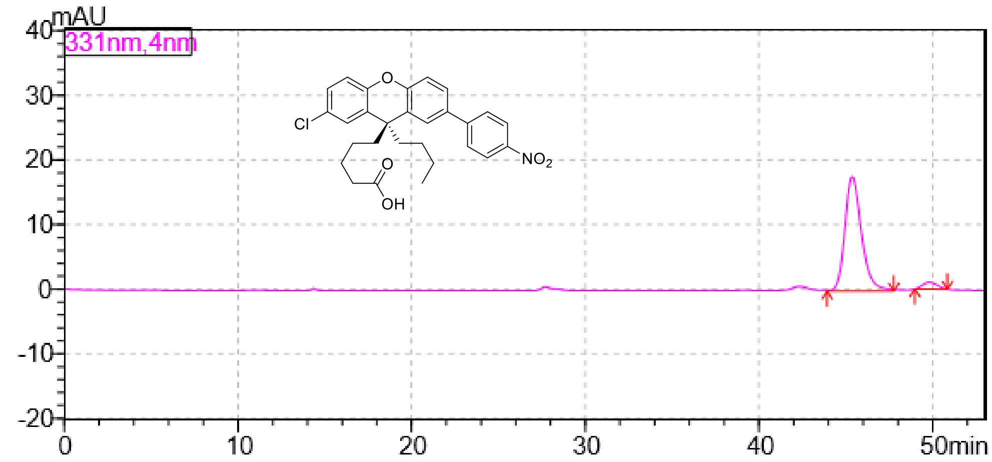

<Peak Table>

PDA Ch1 331nm

Peak#	Ret. Time	Area	Height	Area%	Peak Start	Peak End
1	45.372	1182867	17700	95.000	43.931	47.765
2	49.796	62259	1064	5.000	48.987	50.859
Total		1245127	18764	100.000		

39

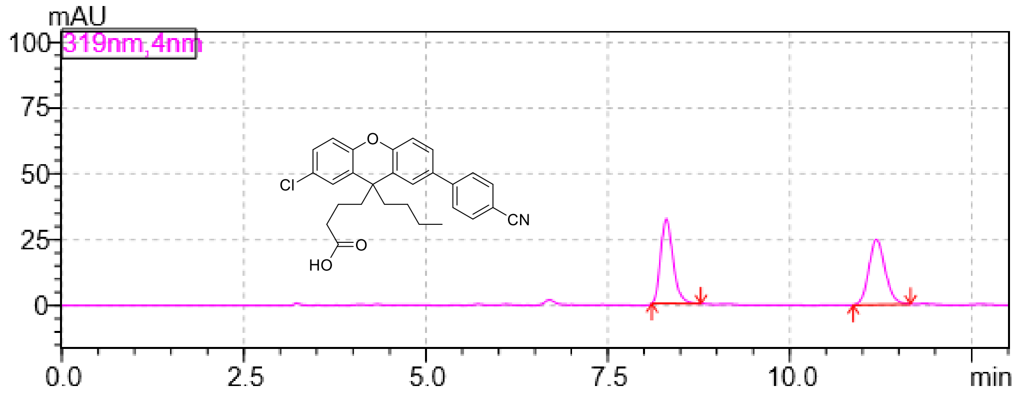

<Peak Table>

PDA Ch1 319nm

Peak#	Ret. Time	Area	Height	Mark	Area%	Peak Start	Peak End
1	8.303	383604	32162	M	49.719	8.107	8.779
2	11.192	387939	24761	M	50.281	10.869	11.659
Total		771543	56923		100.000		

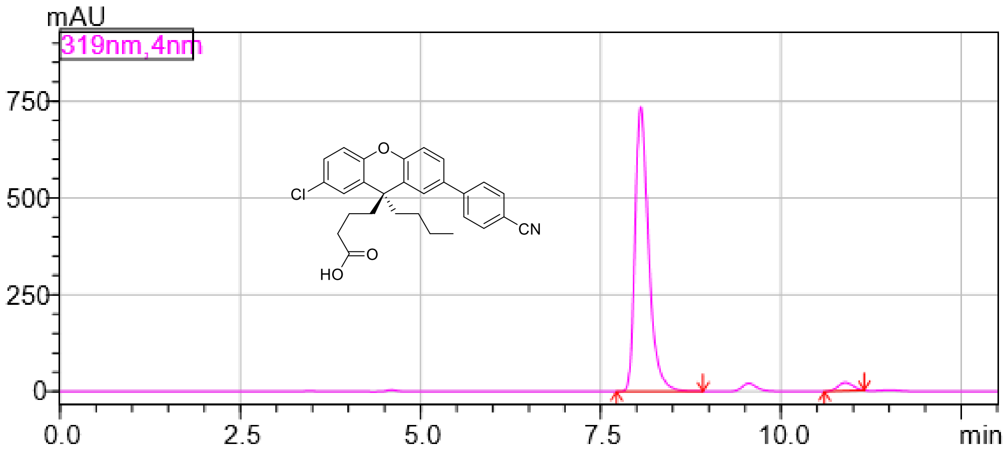

<Peak Table>

PDA Ch1 319nm

Peak#	Ret. Time	Area	Height	Area%	Peak Start	Peak End
1	8.054	9594456	736404	96.844	7.717	8.917
2	10.893	312700	21266	3.156	10.603	11.152
Total		9907156	757670	100.000		

40

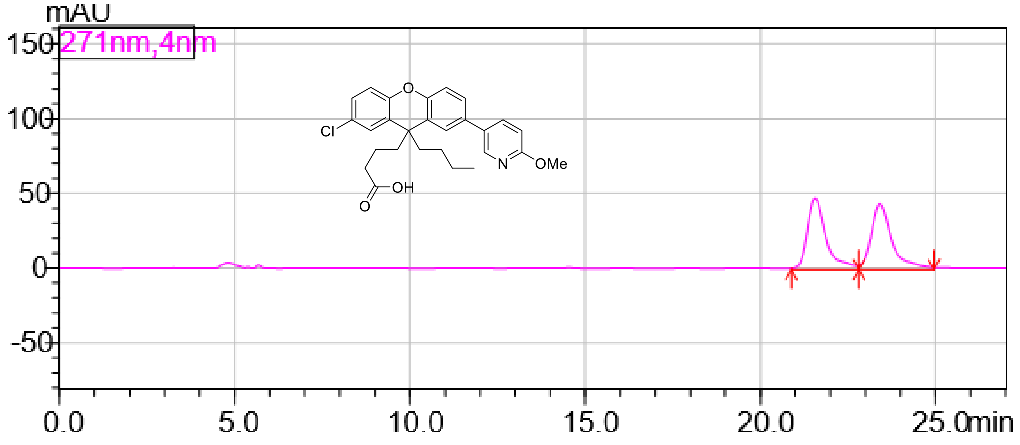

<Peak Table>

PDA Ch1 271nm

Peak#	Ret. Time	Area	Height	Mark	Peak Start	Peak End	Area%
1	21.563	1792694	47725	M	20.885	22.811	49.368
2	23.413	1838617	44044	M	22.811	24.955	50.632
Total		3631312	91769				100.000

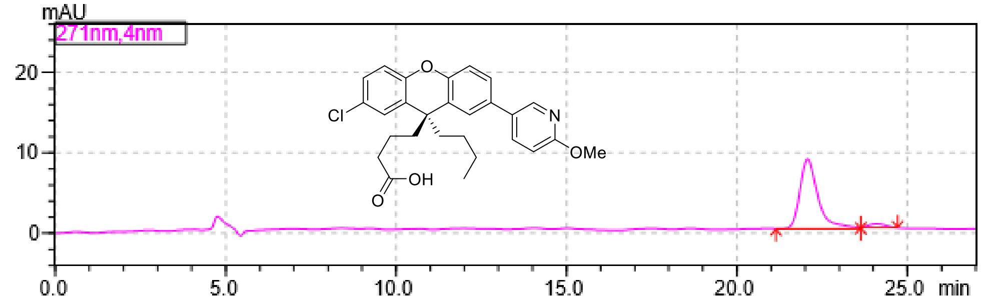

<Peak Table>

PDA Ch1 271nm

Peak#	Ret. Time	Area	Area%	Mark	Peak Start	Peak End	Height
1	22.070	323283	95.226	M	21.141	23.632	8669
2	24.070	16207	4.774	M	23.632	24.709	468
Total		339490	100.000				9138

41

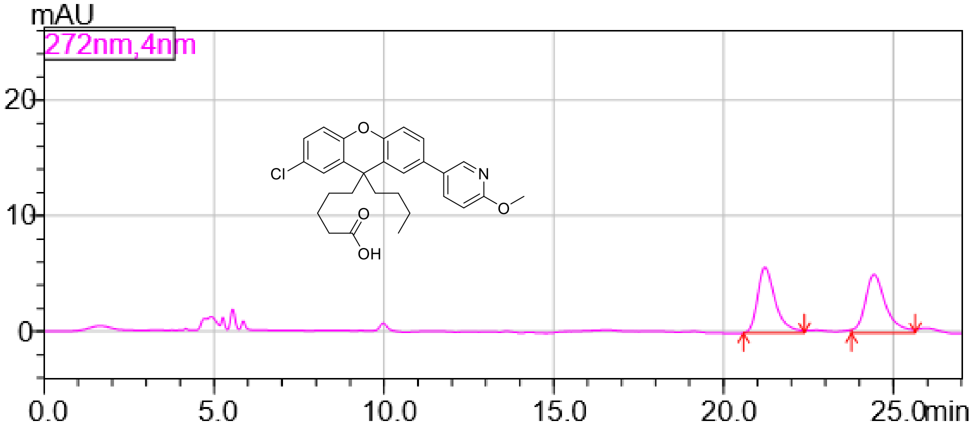

<Peak Table>

PDA Ch1 272nm

Peak#	Ret. Time	Area	Height	Area%	Peak Start	Peak End
1	21.220	208727	5681	49.098	20.587	22.373
2	24.428	216395	5064	50.902	23.771	25.643
Total		425122	10746	100.000		

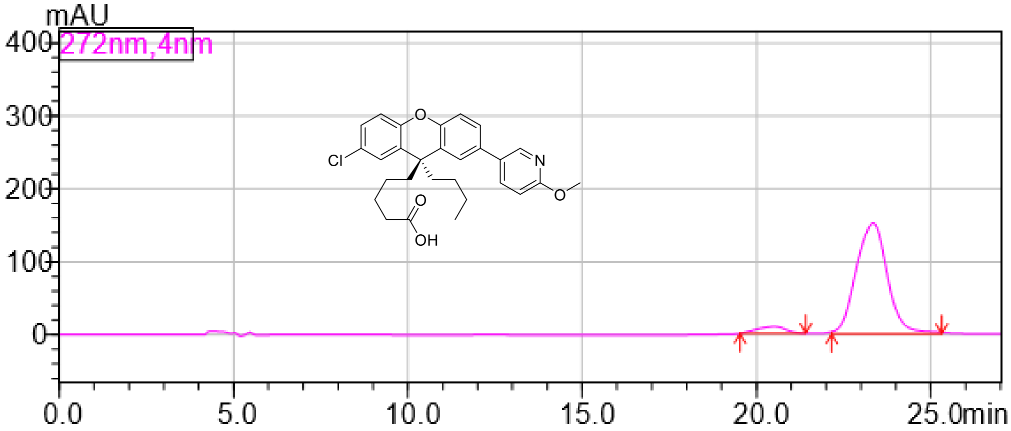

<Peak Table>

PDA Ch1 272nm

Peak#	Ret. Time	Area	Height	Area%	Peak Start	Peak End
1	20.509	560134	9627	5.340	19.461	21.477
2	23.345	9929382	153512	94.660	22.016	26.123
Total		10489516	163140	100.000		

42

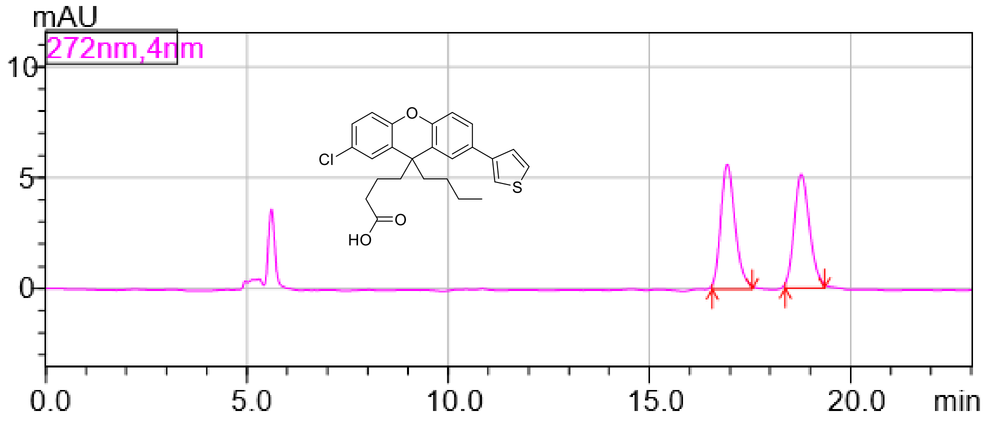

<Peak Table>

PDA Ch1 272nm

Peak#	Ret. Time	Area	Height	Area%	Peak Start	Peak End
1	16.934	141070	5629	50.695	16.565	17.552
2	18.775	137203	5147	49.305	18.379	19.349
Total		278273	10776	100.000		

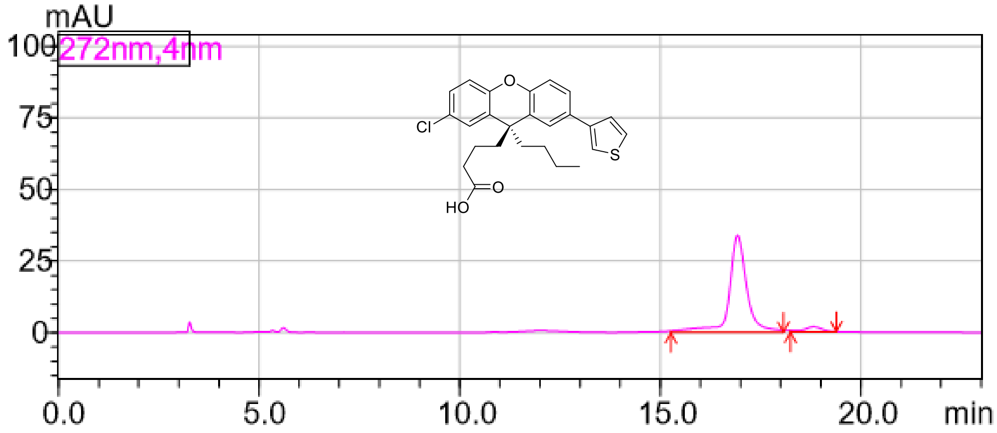

<Peak Table>

PDA Ch1 272nm

Peak#	Ret. Time	Area	Height	Area%	Peak Start	Peak End
1	16.925	1029787	33772	95.132	15.259	18.069
2	18.822	52697	1732	4.868	18.240	19.387
Total		1082484	35503	100.000		

43

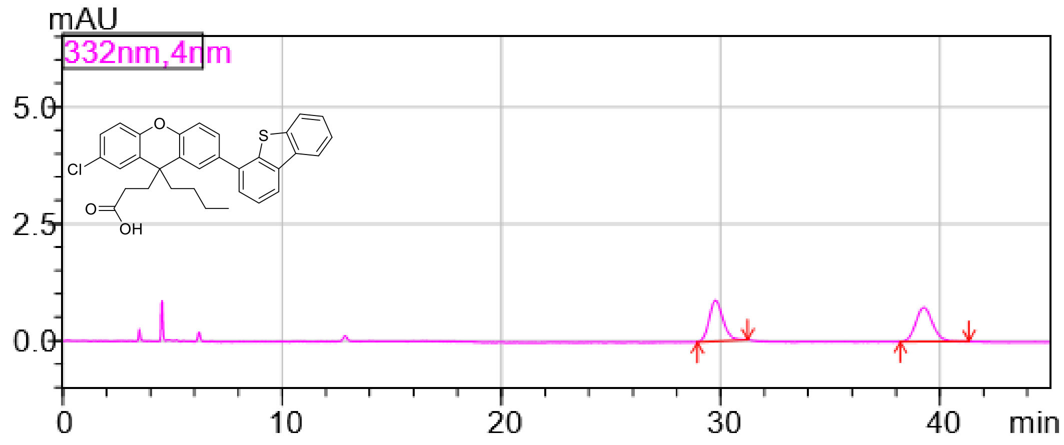

<Peak Table>

PDA Ch1 332nm

Peak#	Ret. Time	Area	Height	Area%	Peak Start	Peak End
1	29.779	38675	877	49.664	28.939	31.243
2	39.274	39198	733	50.336	38.197	41.323
Total		77873	1610	100.000		

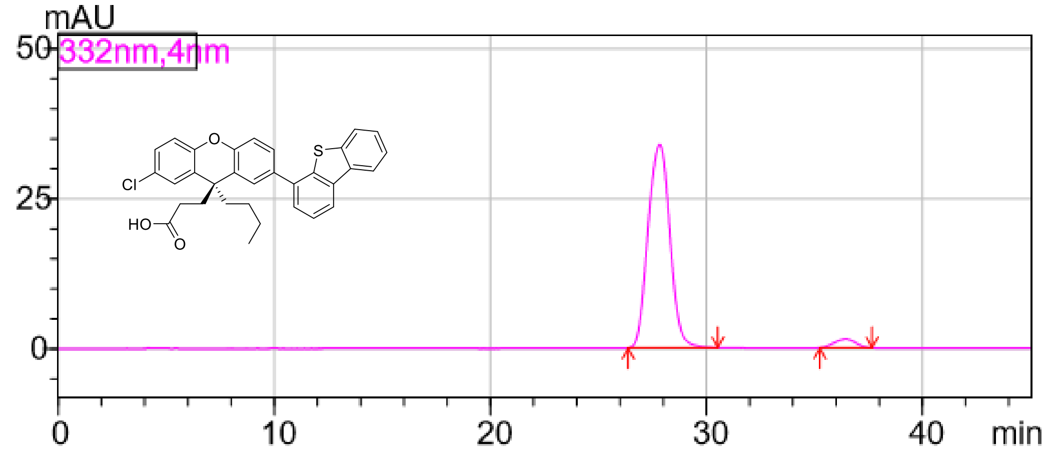

<Peak Table>

PDA Ch1 332nm

Peak#	Ret. Time	Area	Height	Area%	Peak Start	Peak End
1	27.837	2384424	33873	95.965	26.373	30.523
2	36.433	100261	1439	4.035	35.253	37.675
Total		2484685	35312	100.000		

44

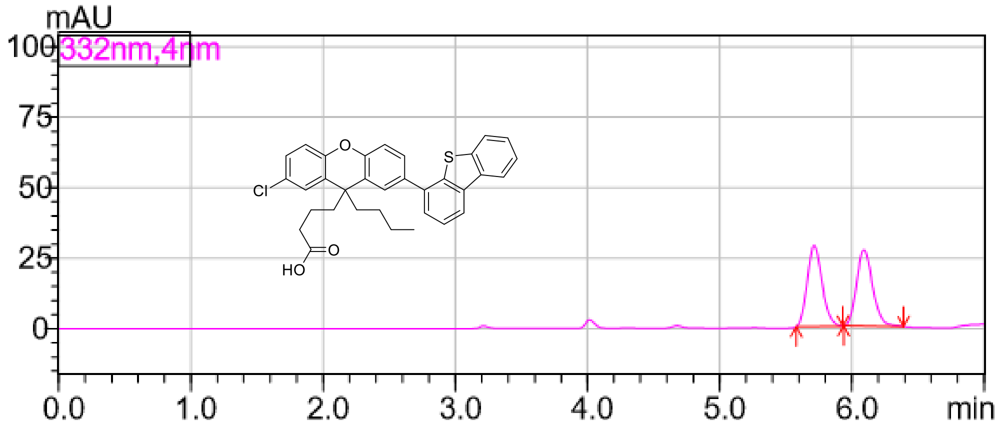

<Peak Table>

PDA Ch1 332nm

Peak#	Ret. Time	Area	Height	Mark	Area%	Peak Start	Peak End
1	5.714	223782	28565	M	49.626	5.579	5.931
2	6.091	227152	26973	M	50.374	5.936	6.389
Total		450934	55538		100.000		

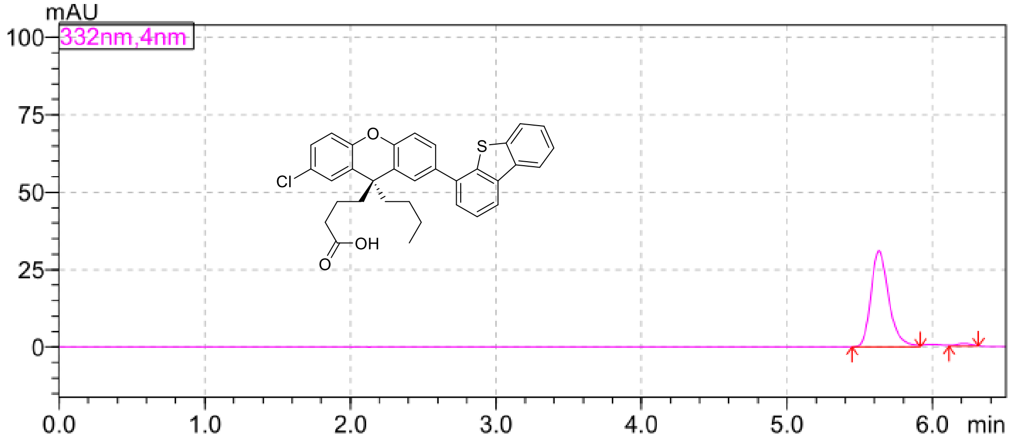

<Peak Table>

PDA Ch1 332nm

Peak#	Ret. Time	Area	Height	Area%	Peak Start	Peak End
1	5.631	267341	31103	97.628	5.445	5.915
2	6.217	6494	828	2.372	6.112	6.315
Total		273836	31932	100.000		

45

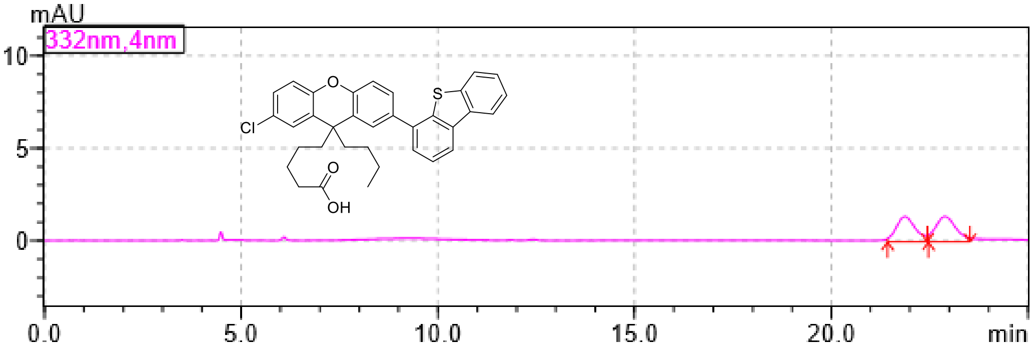

<Peak Table>

PDA Ch1 332nm

Peak#	Ret. Time	Area	Height	Area%	Peak Start	Peak End
1	21.871	47644	1380	48.400	21.424	22.443
2	22.883	50795	1383	51.600	22.464	23.531
Total		98439	2764	100.000		

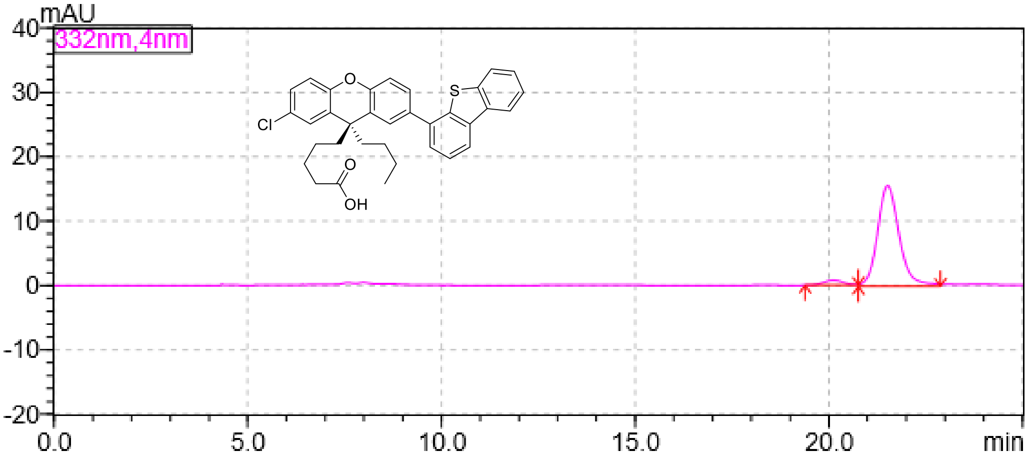

<Peak Table>

PDA Ch1 332nm

Peak#	Ret. Time	Area	Height	Area%	Peak Start	Peak End
1	20.118	28206	784	4.413	19.387	20.763
2	21.517	610994	15668	95.587	20.763	22.880
Total		639200	16452	100.000		

46

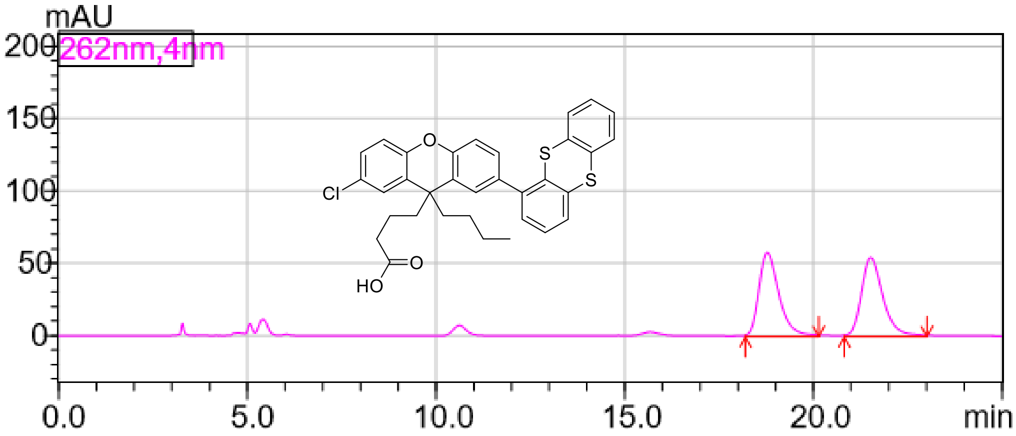

<Peak Table>

PDA Ch1 262nm

Peak#	Ret. Time	Area	Height	Area%	Peak Start	Peak End
1	18.778	2161985	58068	49.635	18.203	20.139
2	21.519	2193740	54571	50.365	20.821	23.003
Total		4355725	112639	100.000		

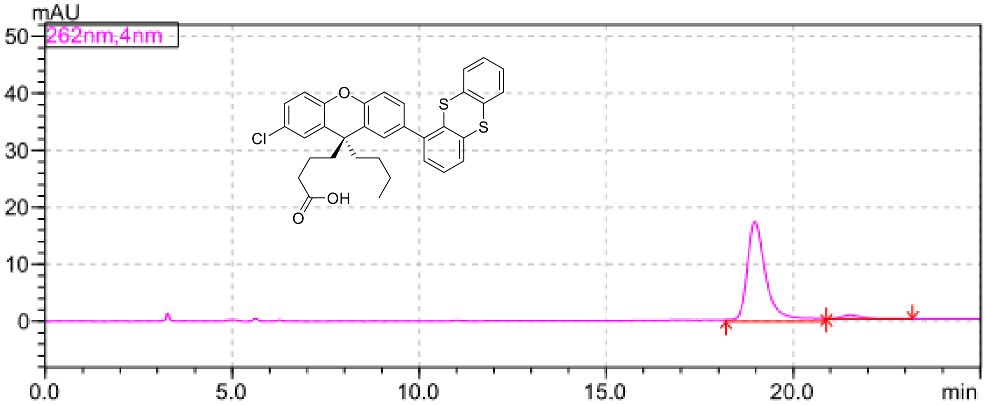

<Peak Table>

PDA Ch1 262nm

Peak#	Ret. Time	Area	Height	Area%	Peak Start	Peak End
1	18.978	626442	17572	96.934	18.203	20.885
2	21.523	19814	622	3.066	20.885	23.195
Total		646256	18194	100.000		

47

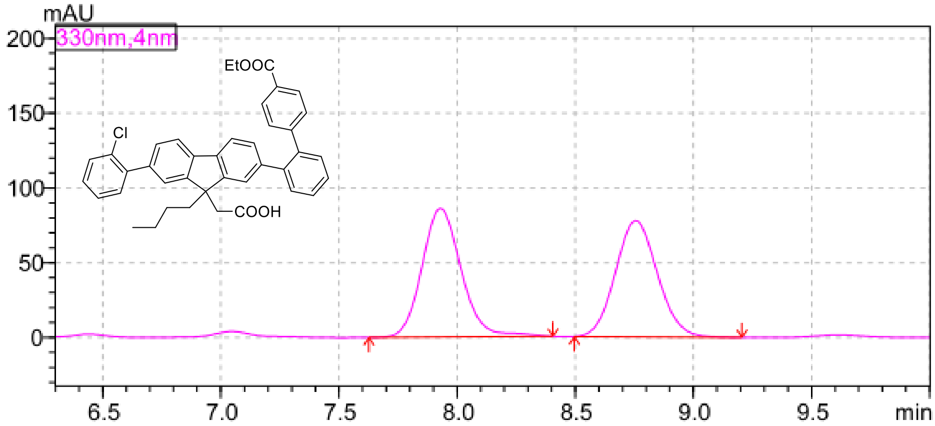

<Peak Table>

PDA Ch1 330nm

Peak#	Ret. Time	Area	Height	Area%	Peak Start	Peak End
1	7.930	998985	86103	50.894	7.627	8.405
2	8.756	963903	77726	49.106	8.496	9.205
Total		1962888	163829	100.000		

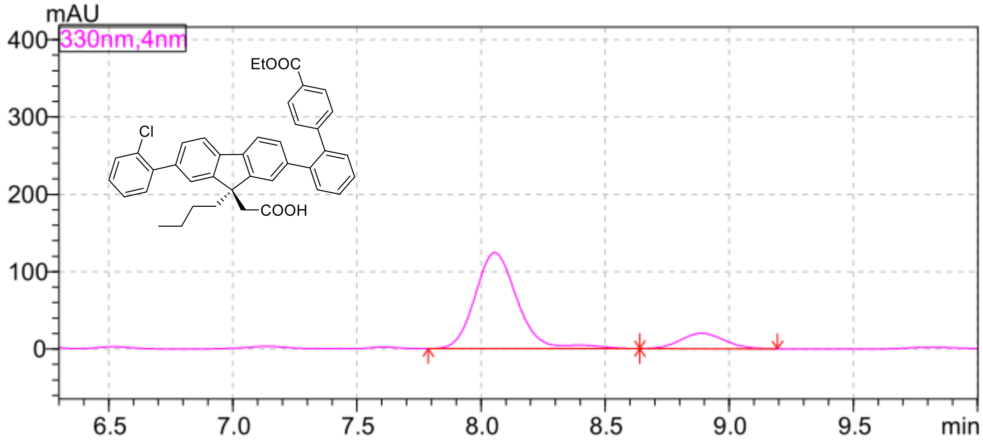

<Peak Table>

PDA Ch1 330nm

Peak#	Ret. Time	Area	Height	Area%	Peak Start	Peak End
1	8.055	1471903	124104	85.718	7.787	8.640
2	8.889	245242	19903	14.282	8.640	9.195
Total		1717145	144007	100.000		

48

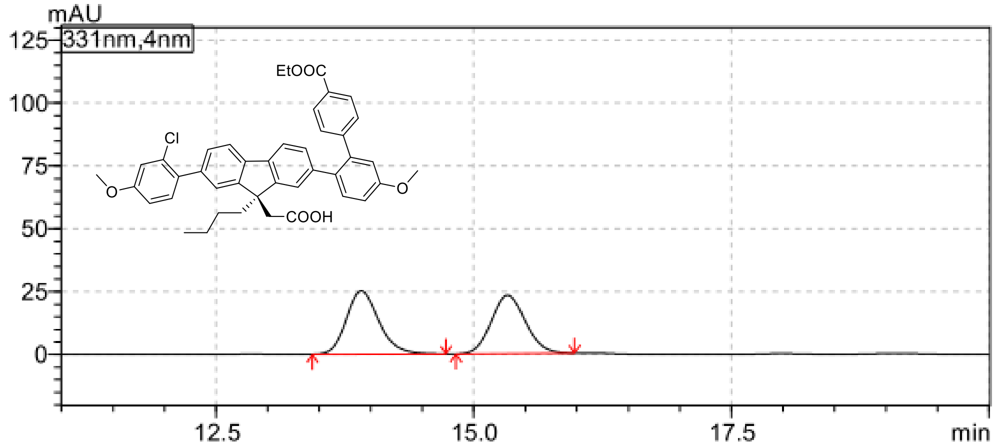

<Peak Table>

PDA Ch1 350nm

Peak#	Ret. Time	Area	Height	Peak Start	Peak End	Area%
1	13.909	118715	5406	13.403	14.688	50.200
2	15.331	117769	5019	14.731	16.315	49.800
Total		236484	10424			100.000

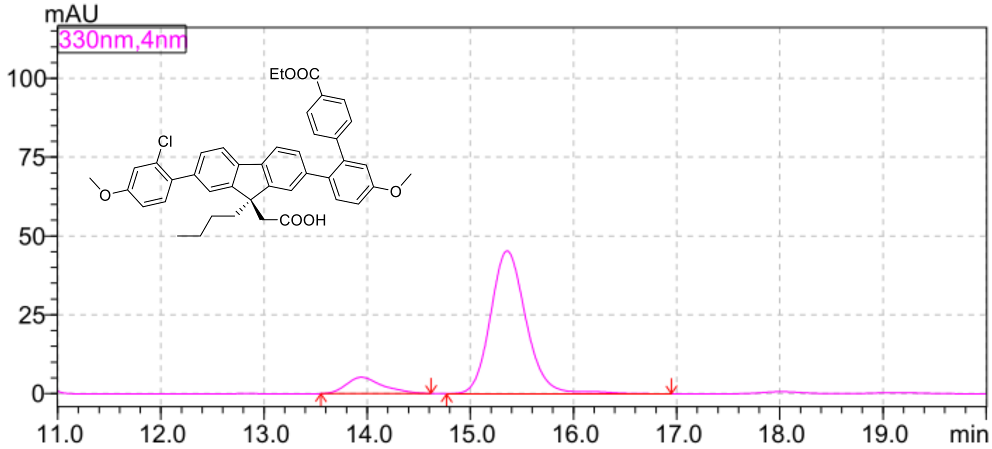

<Peak Table>

PDA Ch1 330nm

Peak#	Ret. Time	Area	Height	Area%	Peak Start	Peak End
1	13.942	128004	5203	10.457	13.552	14.619
2	15.356	1096155	45395	89.543	14.768	16.949
Total		1224159	50598	100.000		

49

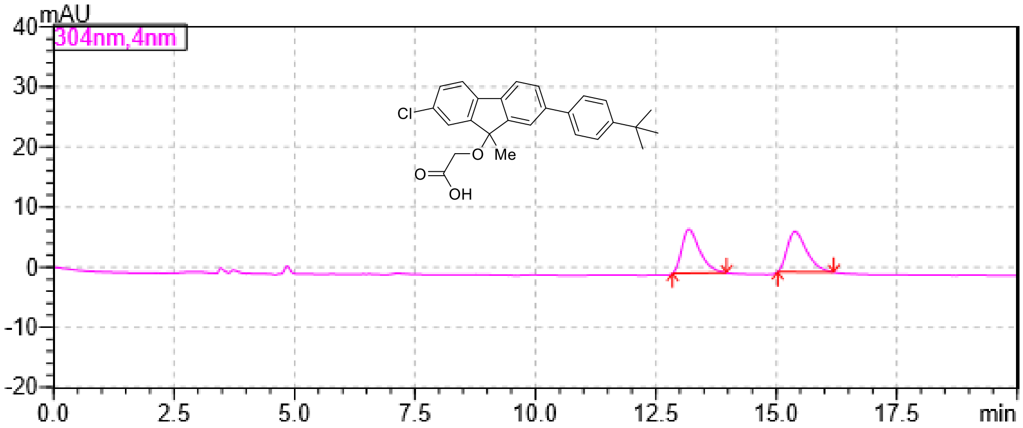

<Peak Table>

PDA Ch1 304nm

Peak#	Ret. Time	Area	Height	Area%	Peak Start	Peak End
1	13.189	188773	7292	49.783	12.848	13.963
2	15.390	190421	6739	50.217	15.029	16.197
Total		379194	14031	100.000		

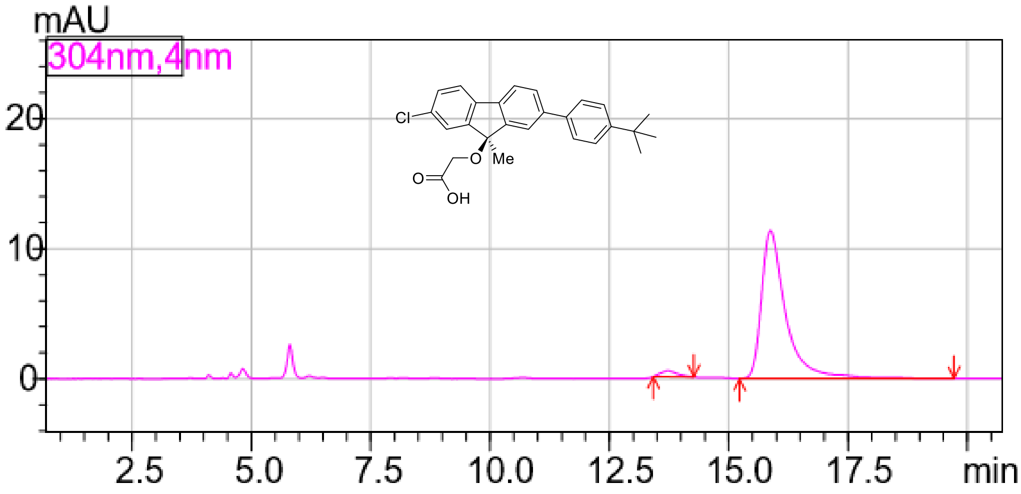

<Peak Table>

PDA Ch1 304nm

Peak#	Ret. Time	Area	Height	Area%	Peak Start	Peak End
1	13.728	7209	362	1.734	13.483	14.133
2	15.875	408642	11397	98.266	15.227	19.717
Total		415851	11758	100.000		

50

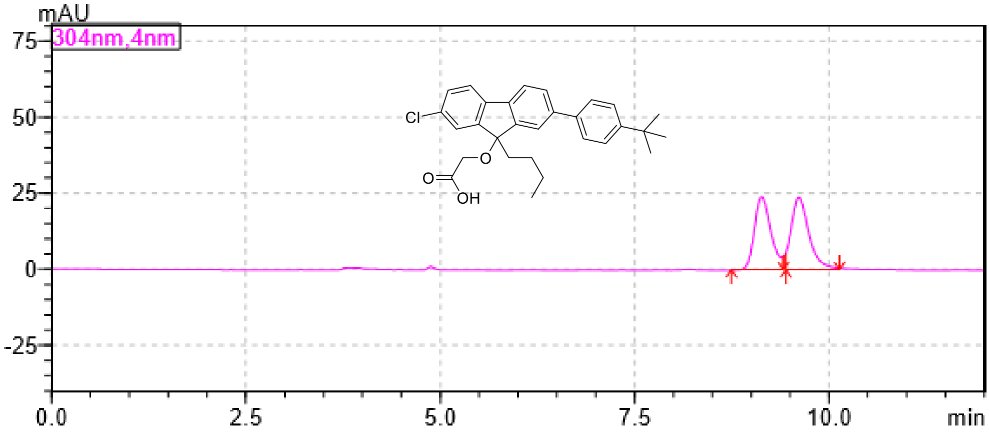

<Peak Table>

PDA Ch1 304nm

Peak#	Ret. Time	Area	Height	Area%	Peak Start	Peak End
1	9.137	353184	24037	48.369	8.747	9.413
2	9.615	377004	23760	51.631	9.445	10.139
Total		730188	47797	100.000		

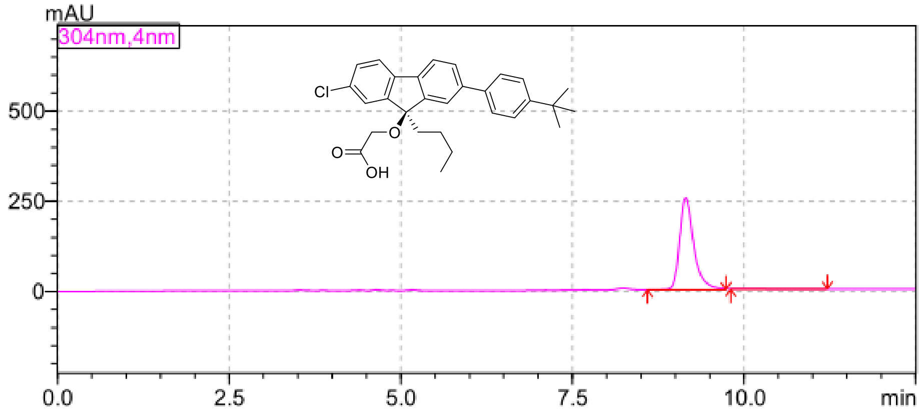

<Peak Table>

PDA Ch1 304nm

Peak#	Ret. Time	Area	Height	Area%	Peak Start	Peak End
1	9.150	3682060	254991	99.201	8.592	9.739
2	10.175	29653	1196	0.799	9.808	11.216
Total		3711713	256188	100.000		

Control experiment (product of reaction using **53**)

Racemic marker

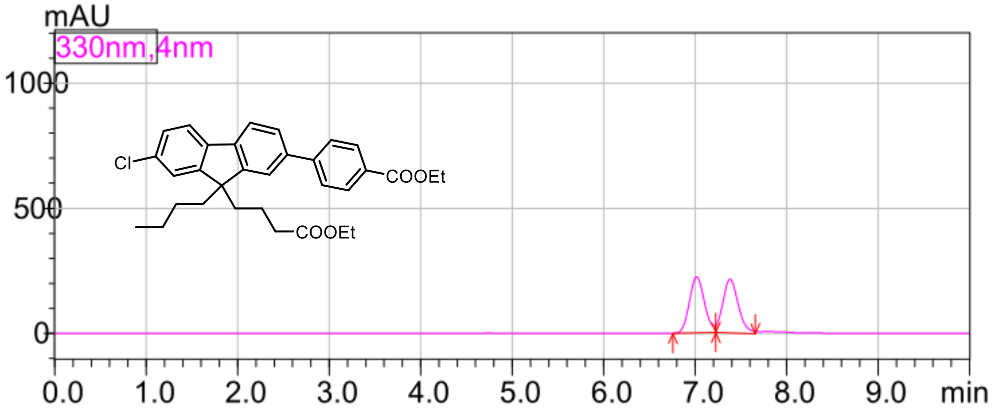

<Peak Table>

PDA Ch1 330nm

Peak#	Ret. Time	Area	Height	Mark	Area%	Peak Start	Peak End
1	7.016	2551333	222426	M	50.105	6.757	7.227
2	7.382	2540657	215334	M	49.895	7.227	7.659
Total		5091990	437761		100.000		

HPLC trace of the coupling product of ethyl ester **53** under the optimized reaction conditions. It was found that the product was racemic.

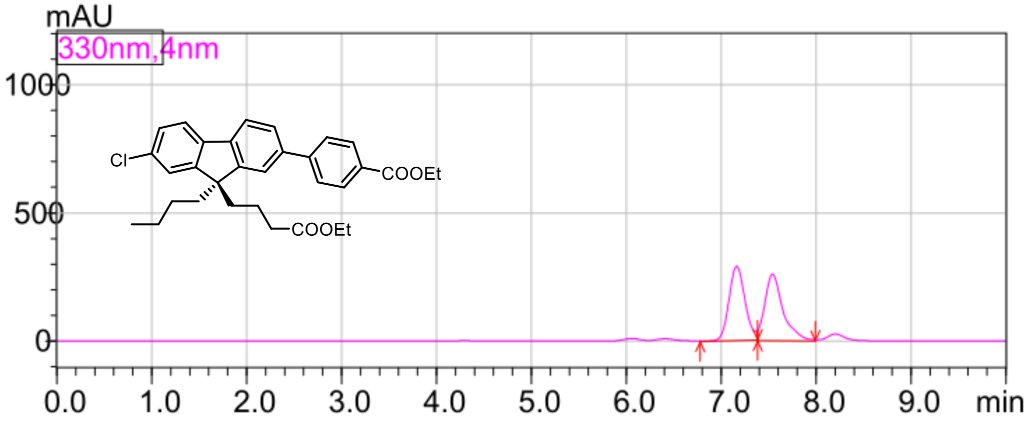

<Peak Table>

PDA Ch1 330nm

Peak#	Ret. Time	Area	Height	Mark	Area%	Peak Start	Peak End
1	7.162	3434882	290805	M	49.686	6.779	7.381
2	7.540	3478269	259984	M	50.314	7.381	7.989
Total		6913151	550788		100.000		

Synthetic applications (Scheme 4)

54

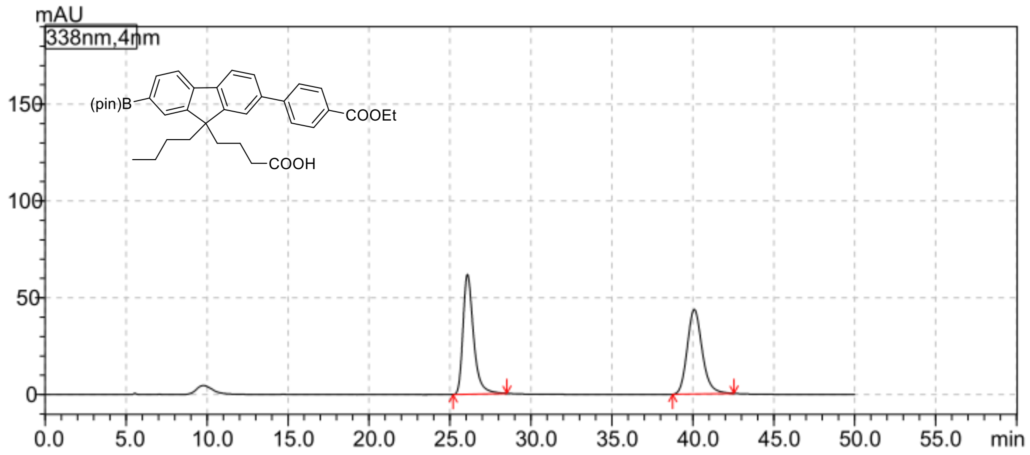

<Peak Table>

PDA Ch1 338nm

Peak#	Ret. Time	Area	Height	Area%	Peak Start	Peak End
1	26.081	2953745	62178	50.553	24.635	33.344
2	40.091	2889143	43921	49.447	37.915	43.856
Total		5842887	106100	100.000		

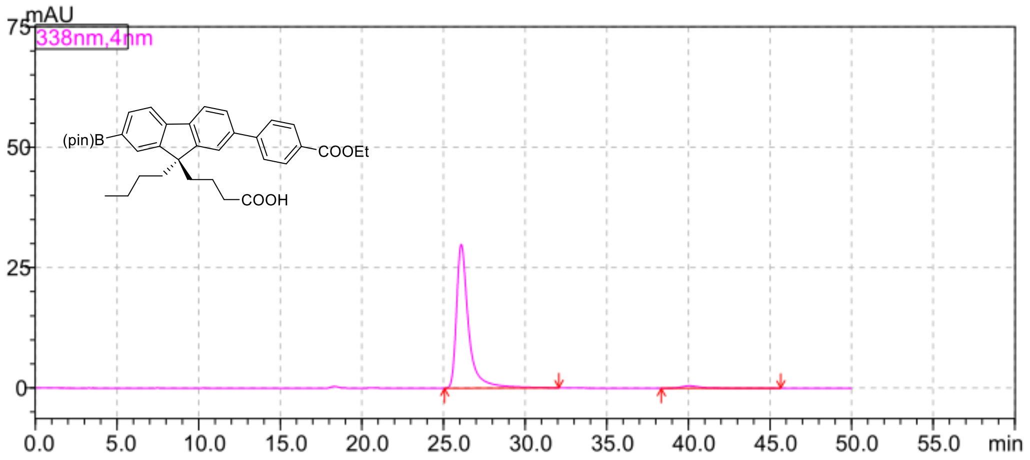

<Peak Table>

PDA Ch1 338nm

Peak#	Ret. Time	Area	Height	Area%	Peak Start	Peak End
1	26.090	1545538	29868	97.144	25.061	32.069
2	40.057	45439	521	2.856	38.336	45.664
Total		1590977	30389	100.000		

55

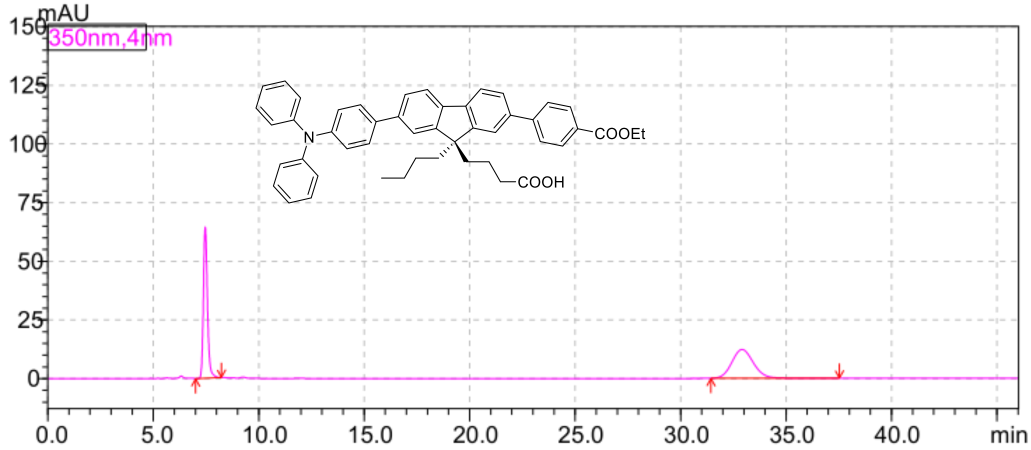

<Peak Table>

PDA Ch1 350nm

Peak#	Ret. Time	Area	Height	Area%	Peak Start	Peak End
1	7.463	865327	64355	49.596	7.003	8.224
2	32.907	879408	12317	50.404	31.429	37.531
Total		1744735	76673	100.000		

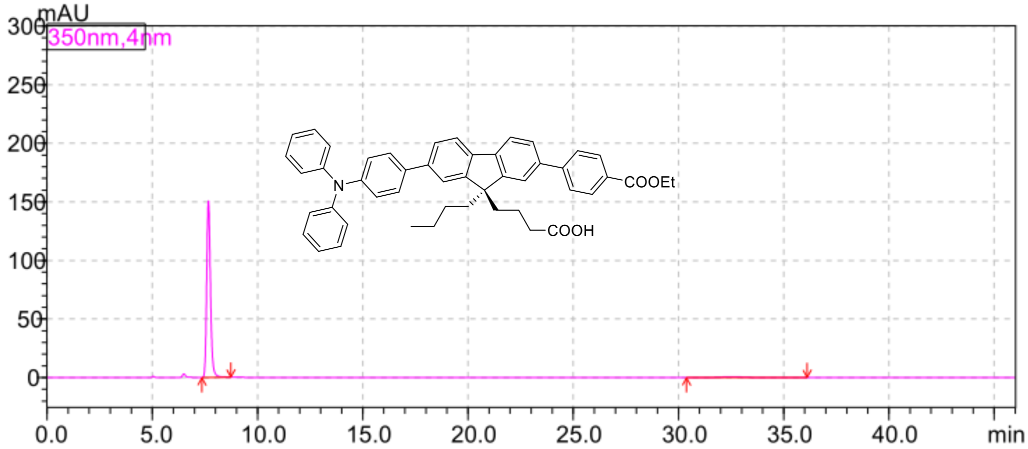

<Peak Table>

PDA Ch1 350nm

Peak#	Ret. Time	Area	Mark	Area%	Peak Start	Peak End
1	7.664	1891075	M	97.552	7.349	8.736
2	32.507	47449	M	2.448	30.373	36.107
Total		1938524		100.000		

56

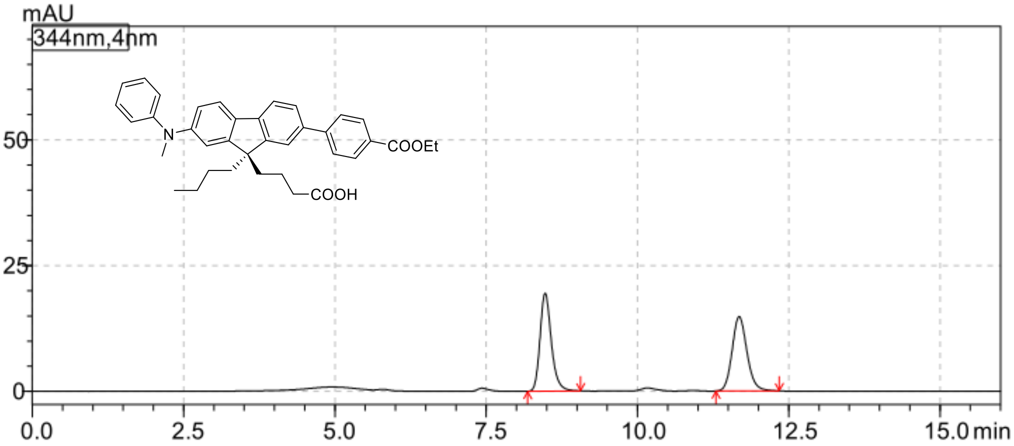

<Peak Table>

PDA Ch1 344nm

Peak#	Ret. Time	Area	Height	Peak Start	Peak End	Area%
1	8.474	255586	19511	8.027	9.195	49.952
2	11.680	256074	14844	11.168	12.528	50.048
Total		511659	34354			100.000

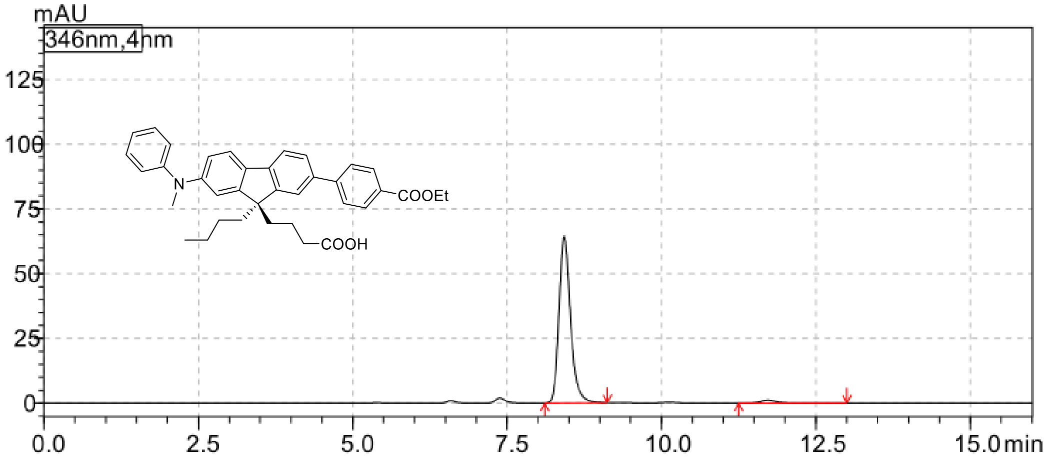

<Peak Table>

PDA Ch1 346nm

Peak#	Ret. Time	Area	Height	Peak Start	Peak End	Area%
1	8.423	840279	64403	8.085	9.307	97.332
2	11.724	23032	1158	11.221	12.971	2.668
Total		863311	65561			100.000

Precursor to **60**

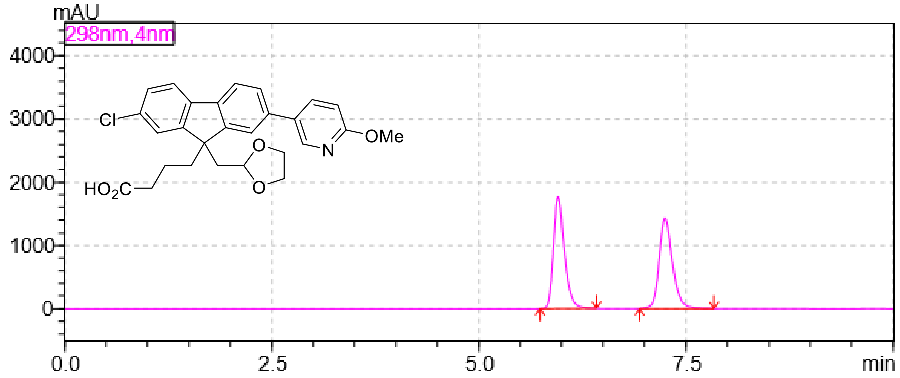

<Peak Table>

PDA Ch1 298nm

Peak#	Ret. Time	Area	Height	Peak Start	Peak End	Area%
1	5.957	16224547	1768166	5.739	6.421	50.350
2	7.247	15998750	1428746	6.939	7.840	49.650
Total		32223297	3196912			100.000

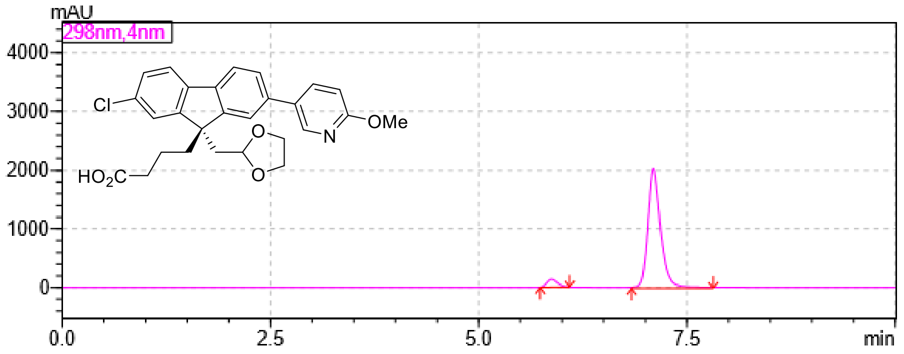

<Peak Table>

PDA Ch1 298nm

Peak#	Ret. Time	Area	Height	Peak Start	Peak End	Area%
1	5.875	1245832	139877	5.739	6.091	5.328
2	7.096	22137992	2046523	6.832	7.813	94.672
Total		23383823	2186400			100.000

Precursor to ***ent*-60**

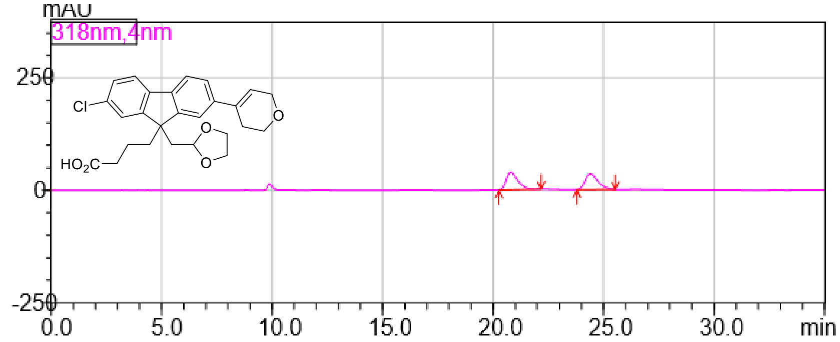

<Peak Table>

PDA Ch1 318nm

Peak#	Ret. Time	Area	Peak Start	Peak End	Area%	Height
1	20.792	1409390	20.251	22.165	49.818	38788
2	24.398	1419696	23.792	25.531	50.182	34637
Total		2829086			100.000	73425

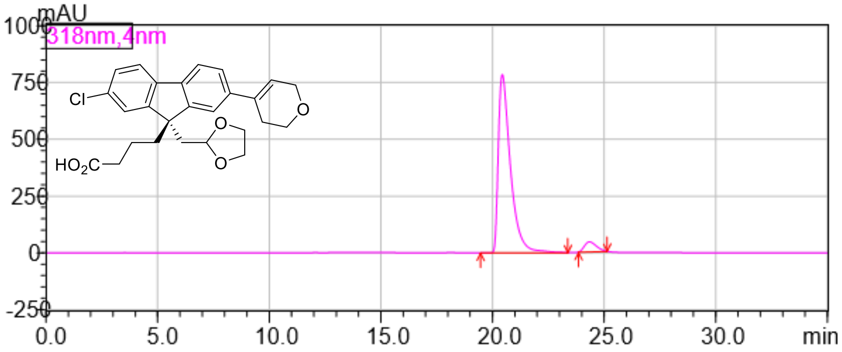

<Peak Table>

PDA Ch1 318nm

Peak#	Ret. Time	Area	Height	Peak Start	Peak End	Area%
1	20.438	29970030	786189	19.461	23.376	94.832
2	24.344	1633227	43861	23.851	25.136	5.168
Total		31603257	830051			100.000

60

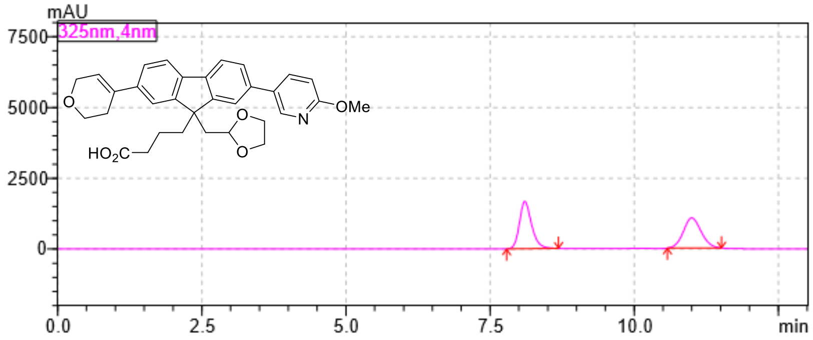

<Peak Table>

PDA Ch1 325nm

Peak#	Ret. Time	Area	Height	Peak Start	Peak End	Area%
1	8.102	24589901	1674022	7.787	8.688	51.668
2	10.999	23002148	1067279	10.576	11.515	48.332
Total		47592049	2741301			100.000

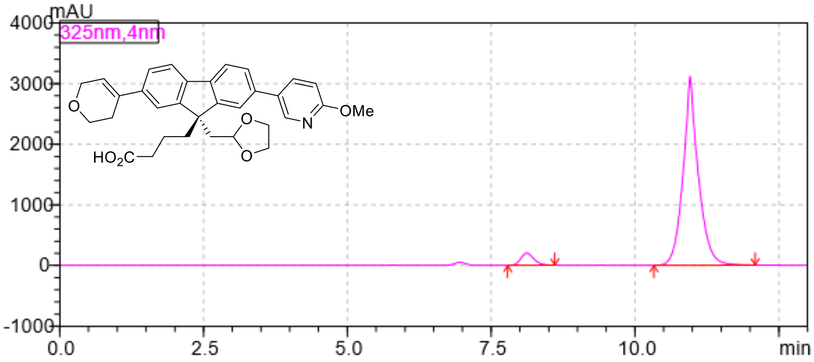

<Peak Table>

PDA Ch1 325nm

Peak#	Ret. Time	Area	Height	Peak Start	Peak End	Area%
1	8.119	3222891	201242	7.787	8.608	5.334
2	10.958	57195559	3113170	10.331	12.091	94.666
Total		60418449	3314412			100.000

***ent*-60**

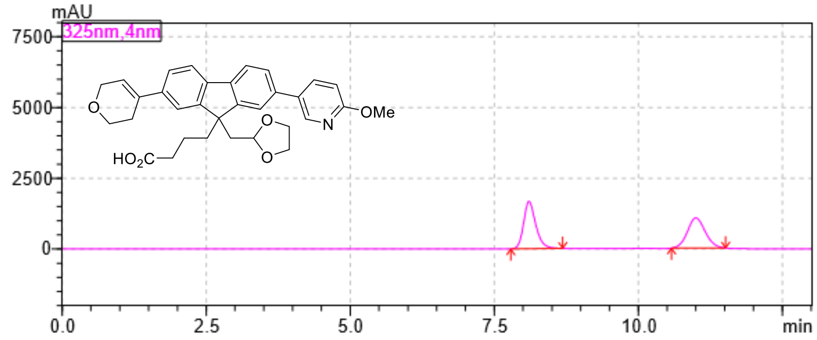

<Peak Table>

PDA Ch1 325nm

Peak#	Ret. Time	Area	Height	Peak Start	Peak End	Area%
1	8.102	24589901	1674022	7.787	8.688	51.668
2	10.999	23002148	1067279	10.576	11.515	48.332
Total		47592049	2741301			100.000

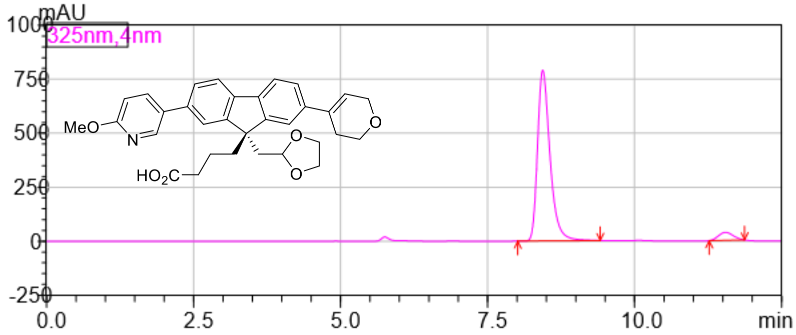

<Peak Table>

PDA Ch1 325nm

Peak#	Ret. Time	Area	Height	Peak Start	Peak End	Area%
1	8.438	11654532	790526	8.011	9.413	94.759
2	11.546	644599	36927	11.269	11.872	5.241
Total		12299131	827453			100.000

61

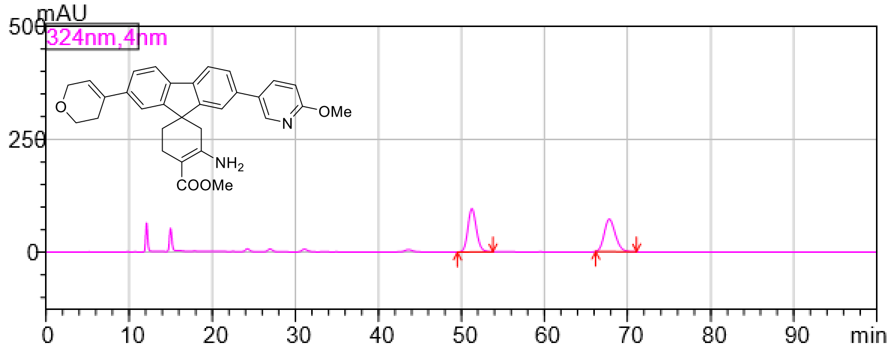

<Peak Table>

PDA Ch1 324nm

Peak#	Ret. Time	Area	Height	Peak Start	Peak End	Area%
1	51.267	6882299	96063	49.515	53.803	50.667
2	67.828	6701199	72095	66.197	71.109	49.333
Total		13583498	168157			100.000

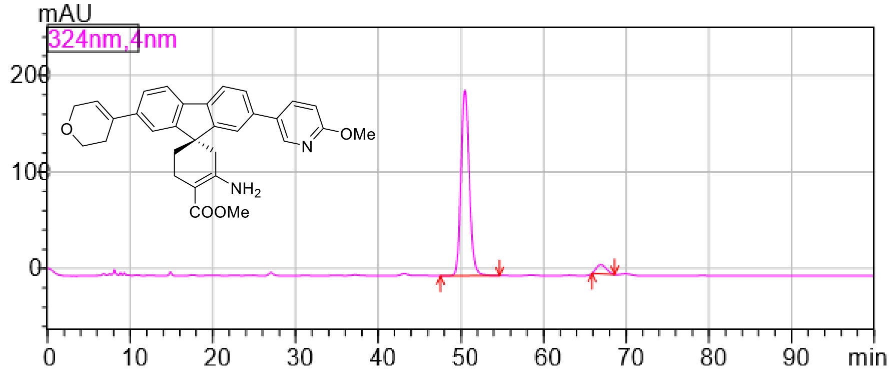

<Peak Table>

PDA Ch1 324nm

Peak#	Ret. Time	Area	Peak Start	Peak End	Area%
1	50.460	13723428	47.504	54.683	94.581
2	66.924	786333	65.872	68.619	5.419
Total		14509761			100.000

63

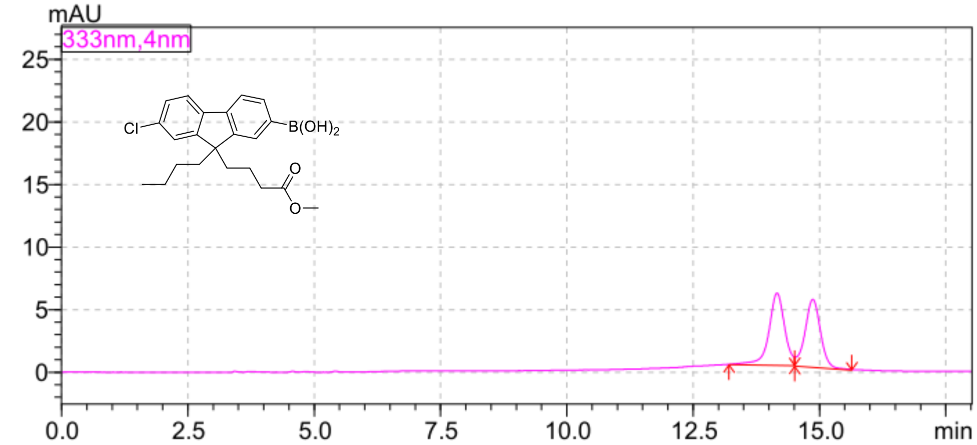

<Peak Table>

PDA Ch1 333nm

Peak#	Ret. Time	Area	Mark	Area%	Peak Start	Peak End
1	14.160	125568	M	51.789	13.211	14.512
2	14.869	116893	M	48.211	14.512	15.643
Total		242461		100.000		

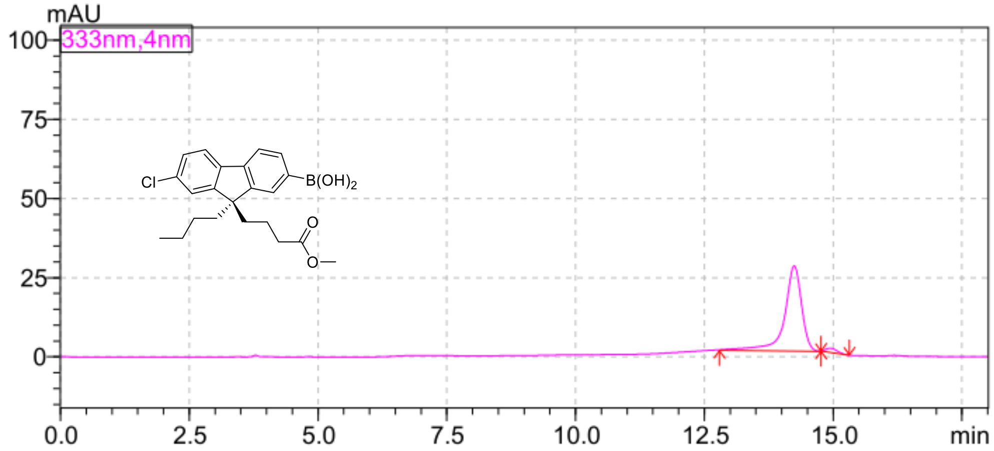

<Peak Table>

PDA Ch1 333nm

Peak#	Ret. Time	Area	Height	Mark	Area%	Peak Start	Peak End
1	14.244	621650	26912	M	97.002	12.789	14.763
2	14.943	19211	1263	M	2.998	14.763	15.312
Total		640861	28175		100.000		

64

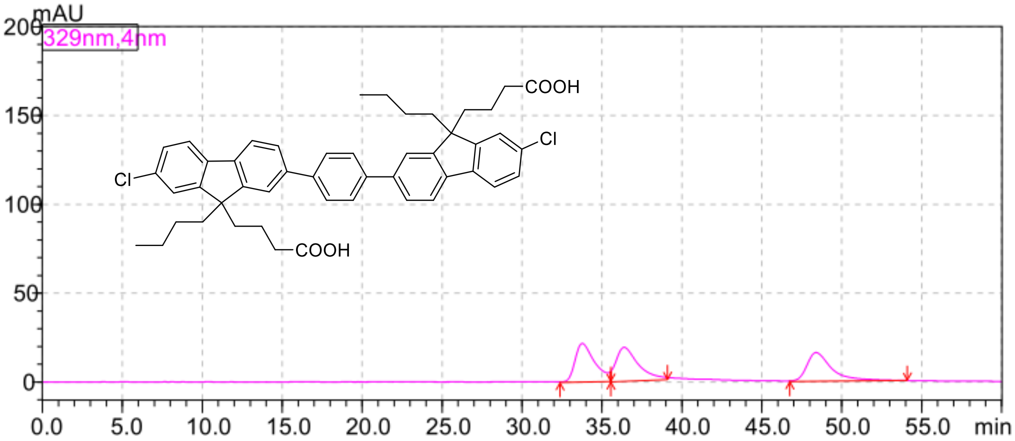

<Peak Table>

PDA Ch1 329nm

Peak#	Ret. Time	Area	Height	Peak Start	Peak End	Area%
1	33.770	1874907	21692	32.379	35.552	34.153
2	36.389	1866352	19072	35.552	39.099	33.997
3	48.390	1748519	16102	46.747	54.091	31.850
Total		5489778	56866			100.000

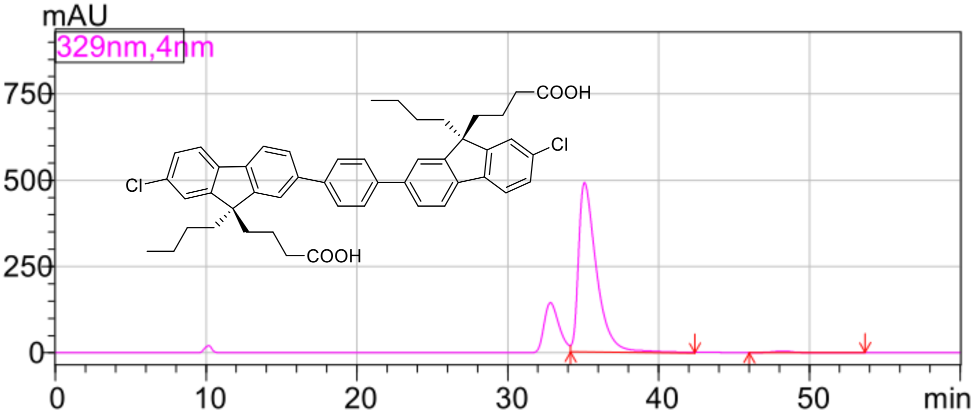

<Peak Table>

PDA Ch1 329nm

Peak#	Ret. Time	Area	Height	Peak Start	Peak End	Area%
1	35.092	40983994	490819	34.165	42.395	99.048
2	48.223	393805	4241	46.016	53.685	0.952
Total		41377798	495060			100.000

meso-**64**

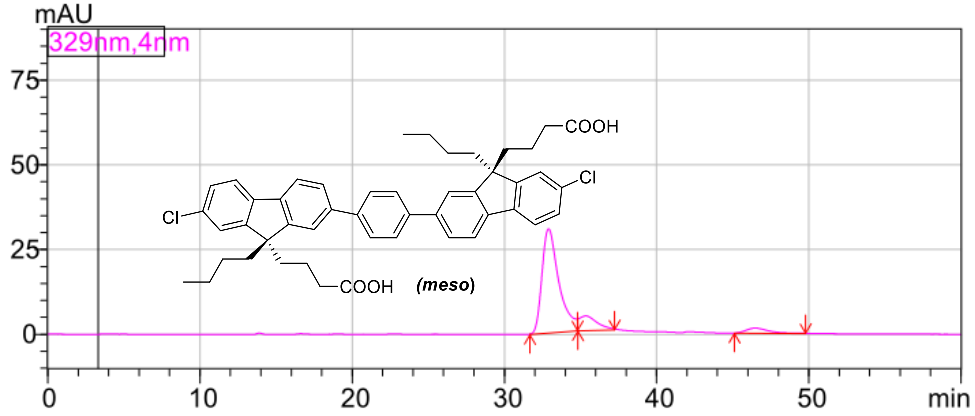

<Peak Table>

PDA Ch1 329nm

Peak#	Ret. Time	Area	Peak Start	Peak End	Area%
1	32.906	2387781	31.669	34.795	82.632
2	35.330	351513	34.837	37.232	12.165
3	46.473	150356	45.104	49.792	5.203
Total		2889650			100.000

Precursor to **65**

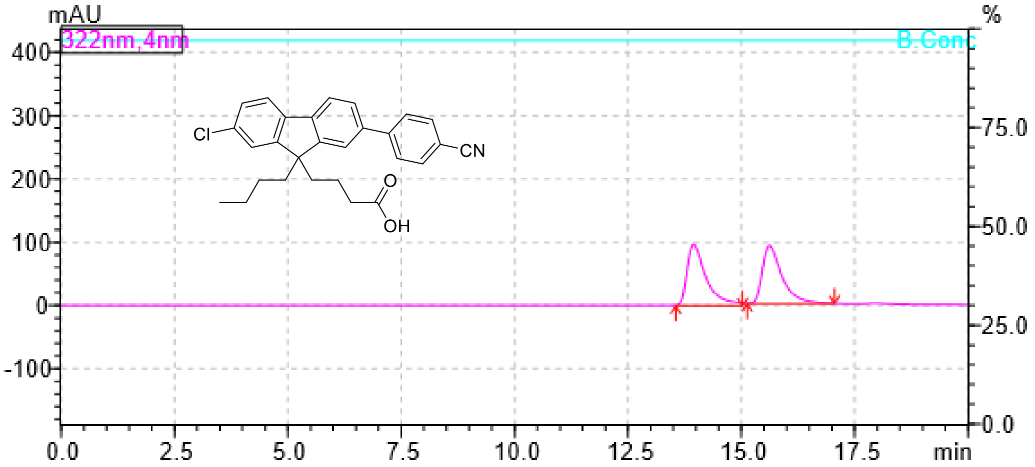

<Peak Table>

PDA Ch1 322nm

Peak#	Ret. Time	Area	Height	Area%	Peak Start	Peak End
1	13.947	2889213	97370	50.854	13.552	15.024
2	15.620	2792205	91985	49.146	15.125	17.056
Total		5681418	189354	100.000		

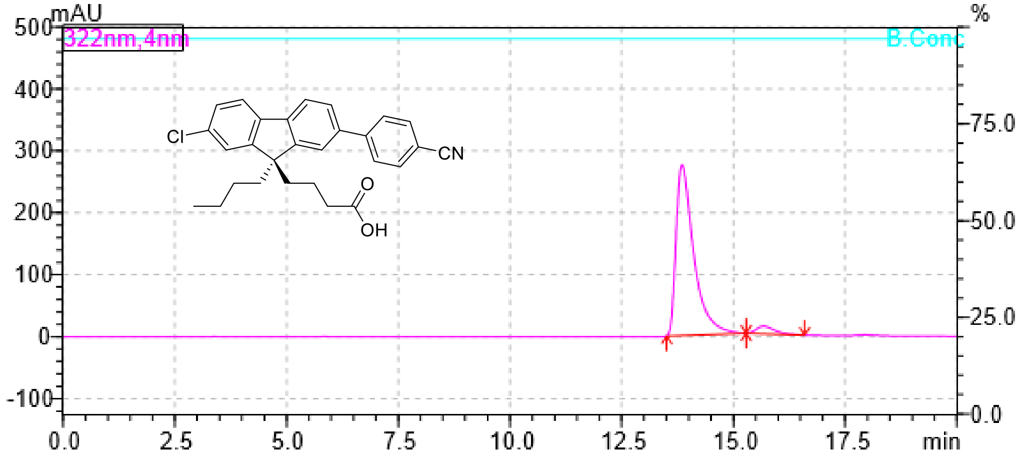

<Peak Table>

PDA Ch1 322nm

Peak#	Ret. Time	Area	Height	Area%	Peak Start	Peak End
1	13.853	7587079	275446	95.639	13.504	15.280
2	15.676	345967	12101	4.361	15.280	16.597
Total		7933046	287546	100.000		

65

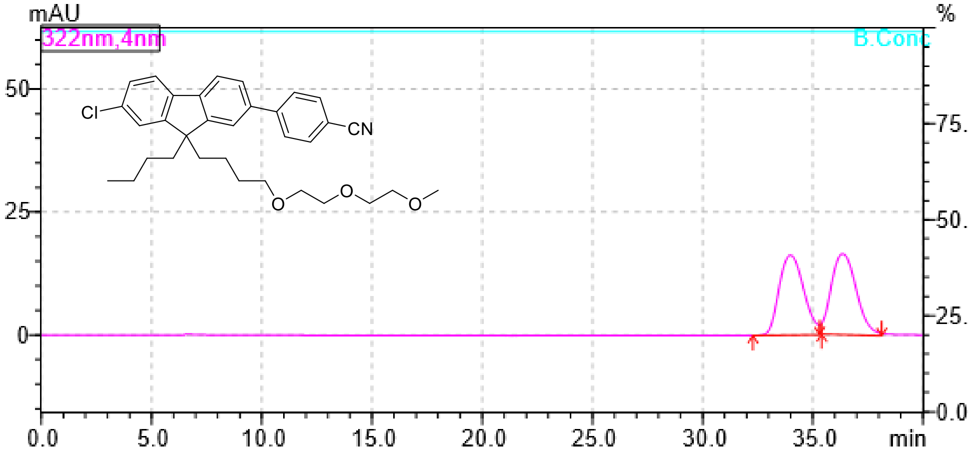

<Peak Table>

PDA Ch1 322nm

Peak#	Ret. Time	Area	Height	Area%	Peak Start	Peak End
1	33.994	1251976	16176	49.189	32.288	35.312
2	36.363	1293281	16395	50.811	35.413	38.139
Total		2545257	32572	100.000		

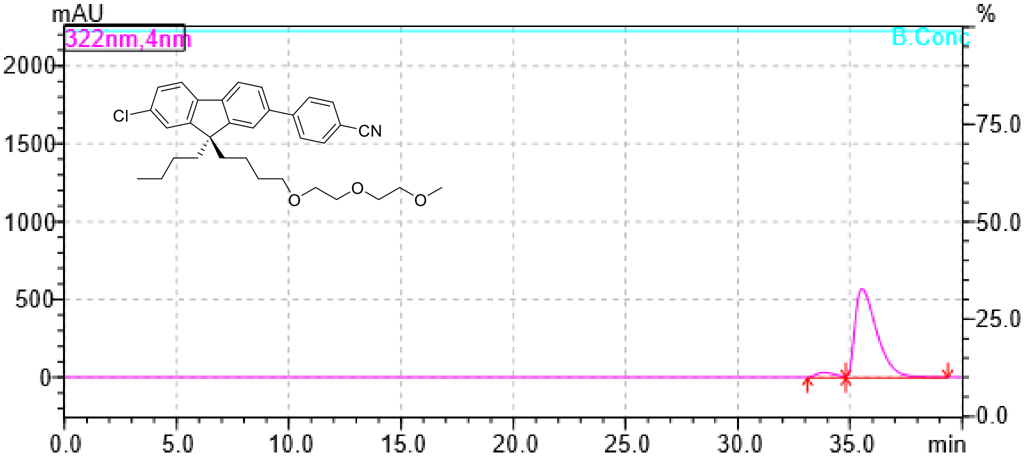

<Peak Table>

PDA Ch1 322nm

Peak#	Ret. Time	Area	Height	Area%	Peak Start	Peak End
1	33.844	2018165	33524	4.906	33.093	34.811
2	35.541	39122132	569940	95.094	34.811	39.349
Total		41140297	603464	100.000		

X-ray crystallography data

Compound (S_S, *S*)-**L2**

The .cif data file is attached as a separate document. CCDC Deposition Number **2054849.**

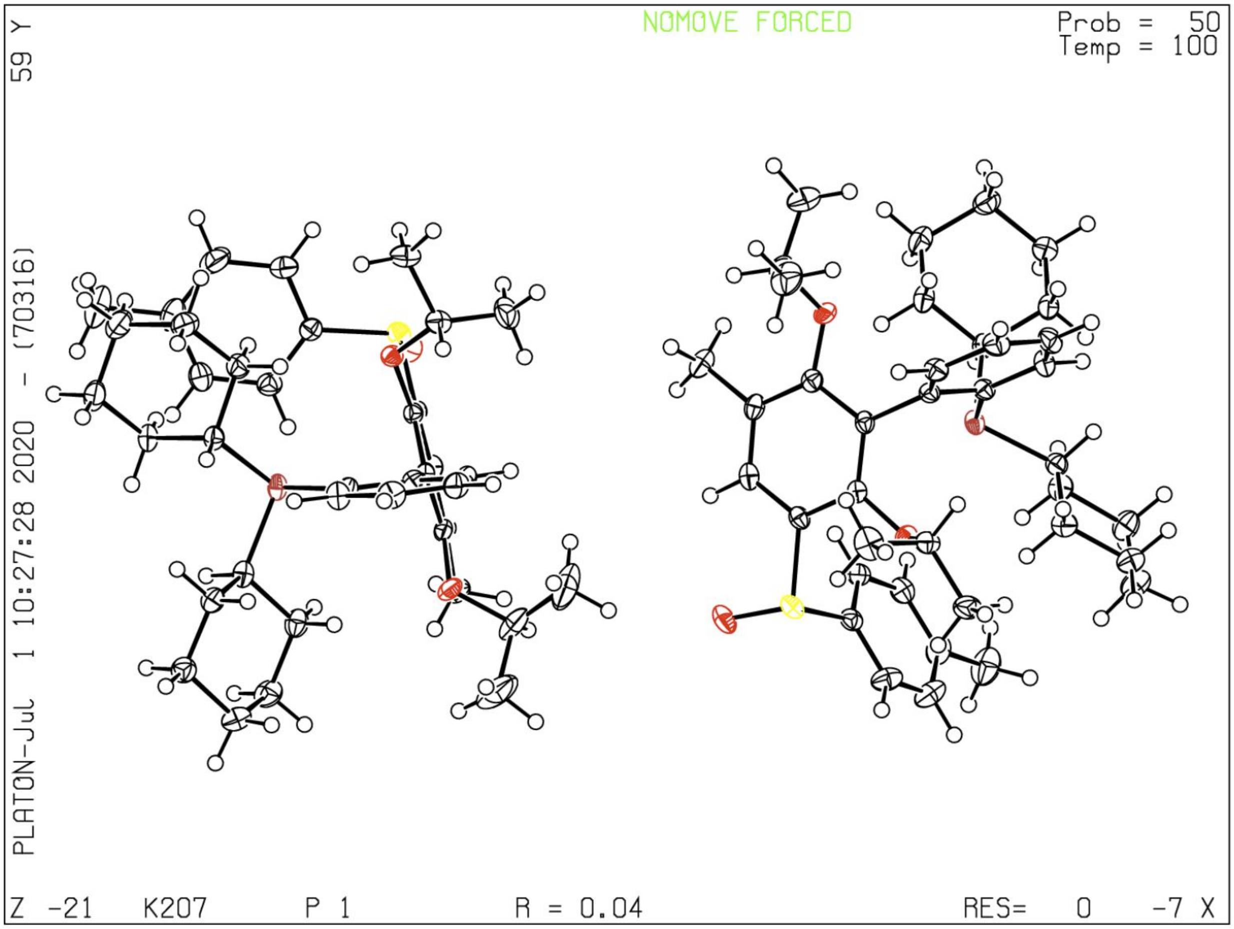

Compound **37**

The .cif data file is attached as a separate document. CCDC Deposition Number **2054848.**

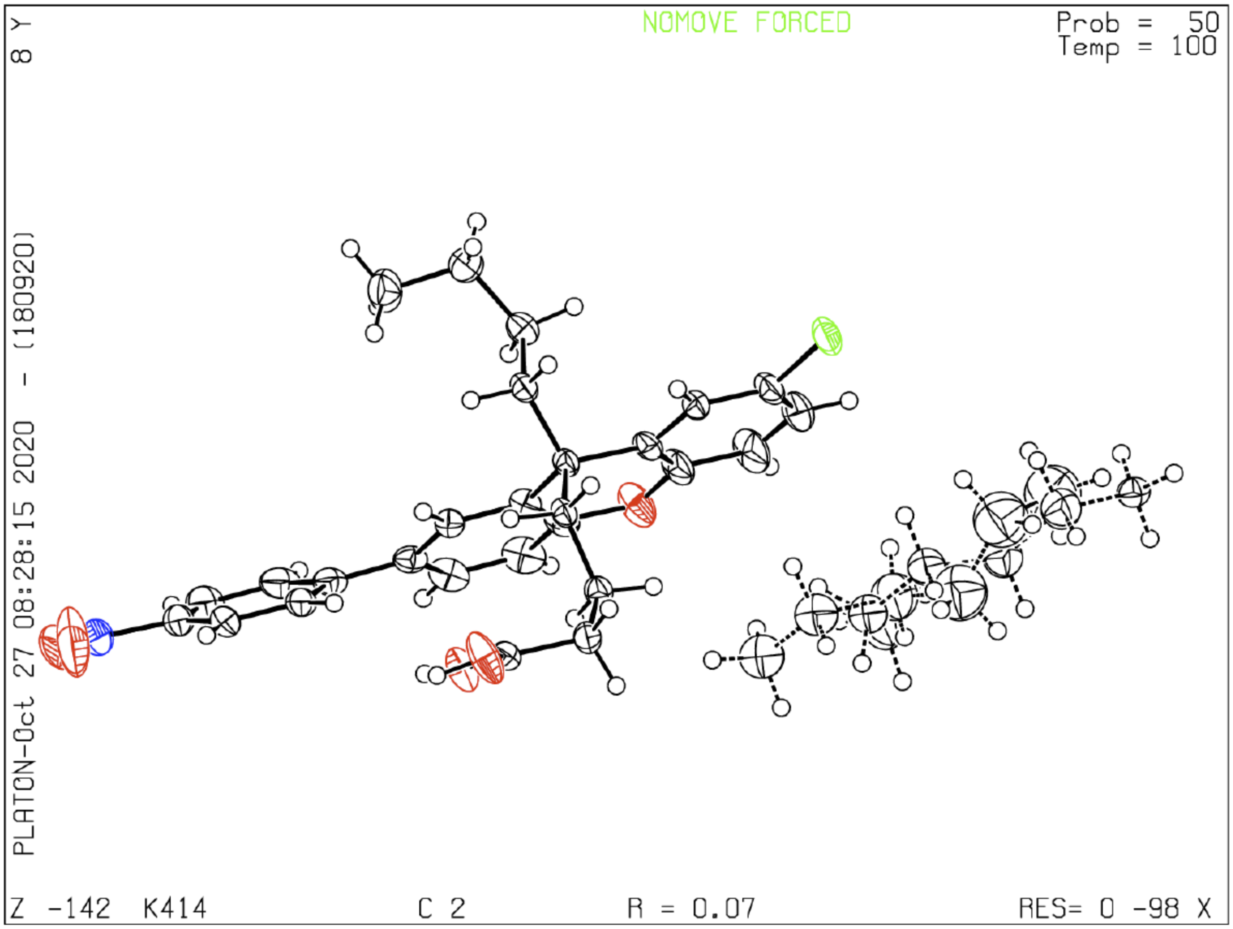

Supplement: Supplementary file 1 — ja1c12345_si_001.pdf [file ja1c12345_si_001.pdf]
